# Supplementary material for: Gaps and drivers of global marine animal biodiversity from the surface to abyss
Source: Nat Commun. 2026 May 27;17:4553. doi: 10.1038/s41467-026-73613-z (PMC13216614; doi:10.1038/s41467-026-73613-z)
Supplement: Supplementary file 1 — Supplementary Information [file 41467_2026_73613_MOESM1_ESM.pdf]

Supplementary Information

Gaps and drivers of global marine animal biodiversity from the surface to abyss

Hanieh Saeedi[1,2]*

[1]Senckenberg Research Institute and Natural History Museum, Senckenberg Data and Modelling Centre and Department of Marine Zoology, Geobiodiversity Informatics, Senckenberganlage 25, 60325 Frankfurt am Main, Germany

[2]Goethe University Frankfurt. Department 15 - Life Sciences, Institute for Ecology, Evolution and Diversity, 60438 Frankfurt am Main, Germany

*Corresponding author: hanieh.saeedi@senckenberg.de

Contents

Supplementary Table 1 3
Supplementary Table 2 4
Supplementary Table 3 4
Supplementary Table 4 5
Supplementary Figure 1 9
Supplementary Figure 3 9
Supplementary Figure 4 10
Supplementary Figure 5 11
Supplementary Figure 6 12
Supplementary Figure 7 13
Supplementary Figure 8 14
Supplementary Figure 9 15
Supplementary Figure 10. 16
Supplementary Figure 11. 17
Supplementary Table 5 18
Supplementary Table 6 18
Supplementary Table 7 18
Supplementary Table 8 19
Supplementary Table 9 19
Supplementary Table 10 19
Supplementary Note 1 20

Supplementary Table 1. The dataset's citations were initially extracted from the Global Biodiversity Information Facility (GBIF). When the data were removed due to quality control, the citation remained in this table.

**Ocean/Sea**	**Citation**
Arctic Ocean	GBIF.org (22 March 2024) GBIF Occurrence Download https://doi.org/10.15468/dl.z6367w ; and GBIF.org (04 April 2024) GBIF Occurrence Download https://doi.org/10.15468/dl.yjky25 ; and GBIF.org (04 April 2024) GBIF Occurrence Download https://doi.org/10.15468/dl.x3mbxr
North-West Pacific Ocean	GBIF.org (27 March 2024) GBIF Occurrence Download https://doi.org/10.15468/dl.w4c3k4
South-West Pacific Ocean	GBIF.org (03 April 2024) GBIF Occurrence Download https://doi.org/10.15468/dl.g98d3t
Indian Ocean	GBIF.org (11 April 2024) GBIF Occurrence Download https://doi.org/10.15468/dl.gbndjc
North Atlantic Ocean	GBIF.org (13 April 2024) GBIF Occurrence Download https://doi.org/10.15468/dl.es2r8w
South Atlantic Ocean	GBIF.org (06 April 2024) GBIF Occurrence Download https://doi.org/10.15468/dl.zb45ax
North-East Pacific Ocean	GBIF.org (11 April 2024) GBIF Occurrence Download https://doi.org/10.15468/dl.xzzzf2
South-East Pacific Ocean	GBIF.org (05 April 2024) GBIF Occurrence Download https://doi.org/10.15468/dl.ycw6ah
Southern Ocean	GBIF.org (22 March 2024) GBIF Occurrence Download https://doi.org/10.15468/dl.jcww6k
Caspian Sea	GBIF.org (07 April 2024) GBIF Occurrence Download https://doi.org/10.15468/dl.yxbqm8
Baltic Sea	GBIF.org (09 April 2024) GBIF Occurrence Download https://doi.org/10.15468/dl.udj9cs
Mediterranean Sea	GBIF.org (07 April 2024) GBIF Occurrence Download https://doi.org/10.15468/dl.sgd26v

Supplementary Table 2. The metric results of the number of occurrence records (NumRec) and species counts (NumSpe) per 5° latitudinal bands,10 m (for shallow water and mesopelagic), and 100 m depth intervals (for deep). The global number of occurrence records and species counts with no depth information were 3,359,550 and 51,900, respectively. Global depth zone includes all depths (0 - 11,000 m), shallow (0 - 200 m), mesopelagic (<200 - 500 m), and deep (> 500 m).

Total			Latitude						Depth					
Depth Zone	Occurrence records	Species counts	Max-NumRec	Min-NumRec	Avg-NumRec	Max-NumSpe	Min-NumSpe	Avg-NumSpe	Max-NumRec	Min-NumRec	Avg-NumRec	Max-NumSpe	Min-NumSpe	Avg-NumSpe
Global	47,995,228	184,141	7,566,285	28,649	1,411,624	38,304	494	16,924	32,452,583	2	440,323	118,621	1	4,855
Shallow	37,942,959	131,980	7,402,048	7,056	1,115,969	25,317	271	10,657	9,842,428	71,863	1,806,808	70,125	7,700	24,841
mesopelagic	3,971,269	49,128	650,912	2,391	116,802	7,275	141	3,689	218,058	42,204	128,105	32,981	5,791	23,185
Deep	2,721,450	51,342	556,993	9,251	80,043	8,735	411	4,270	332,389	2	26,422	20,955	1	2,139

Supplementary Table 3. The metric results of the number of occurrence records (NumRec) and species counts (NumSpe) per 5° latitudinal bands (Latitude) and 100 m depth intervals (Depth) for seven taxa. Only the occurrence records with depth information are included in this table.

Total			Latitude						Depth					
Taxon	Occurrence records	Species counts	Max-NumRec	Min-NumRec	Avg-NumRec	Max-NumSpe	Min-NumSpe	Avg-NumSpe	Max-NumRec	Min-NumRec	Avg-NumRec	Max-NumSpe	Min-NumSpe	Avg-NumSpe
Annelida	3,749,866	13,231	1,096,821	1,808	110,290	2,990	57	1,272	2,861,093	1	39,472	9,280	1	431
Arthropoda	8,607,914	42,759	1,140,982	6,989	253,174	7,885	241	3,657	6,470,758	1	84,391	24,889	1	1,214
Chordata	25,773,808	35,996	4,024,194	715	758,053	10,810	11	4,323	17,920,224	1	289,593	27,571	1	1,228
Cnidaria	2,848,954	14,442	374,554	1,769	83793	3,468	28	1,506	1,519,641	1	32,374	8,680	1	595
Echinodermata	1,285,694	10,901	224,046	1,090	37,815	2,350	12	1,138	706,398	1	13,975	5,737	1	520
Mollusca	3,310,239	42,445	714,954	689	97,360	8,761	31	3,376	2,156,188	1	39,882	27,871	1	1,163
Porifera	920,923	11,793	171,135	121	27,086	1,956	7	805	297,889	1	13,543	6,819	1	401

Supplementary Table 4. The results of the Anderson–Darling (Ad-test) and Hartigan's Dip (Dip-test) tests investigating the normality and modality of the 5° latitude (Lat) and 10m (for shallow water and mesopelagic) and 100m depth intervals for deep water (Dep) plots. The plots include the number of occurrence records (NumRec), number of species (NumSpe), and ES50 (expected species richness) for the global dataset (Global) and for seven taxa, including Annelida, Arthropoda, Chordata, Cnidaria, Echinodermata, Mollusca, and Porifera.

	**Ad-test**	**p-value**	**Dip-test**	**p-value**	**Distribution**	**Modality**
Global-Lat-NumRec	3.1139	5.611e-08	0.0552	0.5436	Non-normal	Unimodal
Global-Lat-NumSpe	0.6432	0.08557	0.0589	0.4704	Non-normal	Unimodal
Global-Lat-ES50	1.3559	0.001364	0.0649	0.3040	Normal	Unimodal
Global-Dep-NumRec	39.720	< 2.2e-16	0.0257	0.9133	Non-normal	Unimodal
Global-Dep-NumSpe	23.238	< 2.2e-16	0.0360	0.4029	Normal	Unimodal
Global-Dep_ES50	4.0619	3.369e-10	0.0376	0.5876	Non-normal	Unimodal
Shallow-Lat-NumRec	4.2057	1.118e-10	0.0556	0.5306	Non-normal	Unimodal
Shallow-Lat-NumSpe	0.4271	0.2958	0.0431	0.9291	Normal	Unimodal
Shallow-Lat-ES50	1.0122	0.01002	0.0489	0.7939	Non-normal	Unimodal
Shallow-Dep-NumRec	3.7262	1.114e-09	0.0519	0.9566	Non-normal	Unimodal
Shallow-Dep-NumSpe	1.0644	0.006684	0.0526	0.9373	Non-normal	Unimodal
Shallow-Dep_ES50	0.4230	0.2911	0.0460	0.9902	Normal	Unimodal
Meso-Lat-NumRec	4.8675	2.638e-12	0.0579	0.4570	Non-normal	Unimodal
Meso-Lat-NumSpe	0.3027	0.5556	0.0601	0.4355	Non-normal	Unimodal

Meso-Lat-ES50	0.7050	0.05969	0.0535	0.6491	Non-normal	Unimodal
Meso-Dep-NumRec	3.7262	1.114e-09	0.0519	0.9566	Non-normal	Unimodal
Meso-Dep-NumSpe	0.1797	0.9085	0.0508	0.8016	Normal	Unimodal
Meso-Dep_ES50	0.3712	0.4012	0.0683	0.2915	Normal	Unimodal
Deep-Lat-NumRec	4.5160	1.925e-11	0.0301	0.9952	Non-normal	Unimodal
Deep-Lat-NumSpe	0.5509	0.1444	0.0442	0.9092	Normal	Unimodal
Deep-Lat-ES50	0.3359	0.4879	0.0424	0.9415	Normal	Unimodal
Deep-Dep-NumRec	23.9590	< 2.2e-16	0.0269	0.8918	Non-normal	Unimodal
Deep-Dep-NumSpe	15.2810	< 2.2e-16	0.0378	0.3622	Non-normal	Unimodal
Deep-Dep_ES50	3.8040	1.413e-09	0.0414	0.4656	Non-normal	Unimodal
Cnidaria-Lat-NumRec	2.5414	1.489e-06	0.0606	0.3745	Non-normal	Unimodal
Cnidaria -Lat-NumSpe	0.5740	0.1256	0.0601	0.4342	Normal	Unimodal
Cnidaria -Lat-ES50	0.8758	0.02211	0.0424	0.9407	Non-normal	Unimodal
Cnidaria -Dep-NumRec	34.8290	< 2.2e-16	0.0272	0.8526	Non-normal	Unimodal
Cnidaria -Dep-NumSpe	14.6890	< 2.2e-16	0.0402	0.3870	Non-normal	Unimodal
Cnidaria -Dep_ES50	1.48230	0.000716	0.0319	0.9631	Non-normal	Unimodal
Porifera-Lat-NumRec	4.9997	1.25e-12	0.0454	0.8531	Non-normal	Unimodal

Porifera -Lat-NumSpe	0.4586	0.2475	0.0436	0.9193	Normal	Unimodal
Porifera -Lat-ES50	0.3342	0.4925	0.0607	0.4163	Normal	Unimodal
Porifera -Dep-NumRec	34.8290	< 2.2e-16	0.0272	0.8526	Non-normal	Unimodal
Porifera -Dep-NumSpe	12.7040	< 2.2e-16	0.0338	0.8664	Non-normal	Unimodal
Porifera -Dep_ES50	1.1329	0.005257	0.0329	0.9723	Non-normal	Unimodal
Echinodermata-Lat-NumRec	4.2585	8.286e-11	0.0351	0.9910	Non-normal	Unimodal
Echinodermata -Lat-NumSpe	0.3818	0.3804	0.0690	0.2152	Normal	Unimodal
Echinodermata -Lat-ES50	1.6156	0.0003035	0.0440	0.9120	Non-normal	Unimodal
Echinodermata -Dep-NumRec	37.0450	< 2.2e-16	0.0246	0.9458	Non-normal	Unimodal
Echinodermata -Dep-NumSpe	13.5020	< 2.2e-16	0.0232	0.9909	Non-normal	Unimodal
Echinodermata -Dep_ES50	1.8742	7.62e-05	0.0470	0.4282	Non-normal	Unimodal
Annelida-Lat-NumRec	7.6991	2.2e-16	0.0366	0.9886	Non-normal	Unimodal
Annelida -Lat-NumSpe	0.5489	0.146	0.0460	0.8688	Normal	Unimodal
Annelida -Lat-ES50	39.2970	< 2.2e-16	0.0409	0.1979	Non-normal	Unimodal
Annelida -Dep-NumRec	18.5940	< 2.2e-16	0.0478	0.1133	Non-normal	Unimodal
Annelida -Dep-NumSpe	2.1733	1.214e-05	0.0419	0.9502	Non-normal	Unimodal
Annelida -Dep_ES50	4.6711	1.009e-11	0.0376	0.8207	Non-normal	Unimodal

Arthropoda-Lat-NumRec	7.6991	< 2.2e-16	0.0366	0.9886	Non-normal	Unimodal
Arthropoda -Lat-NumSpe	0.4144	0.3176	0.0690	0.2154	Normal	Unimodal
Arthropoda -Lat-ES50	40.2520	< 2.2e-16	0.0245	0.9485	Non-normal	Unimodal
Arthropoda -Dep-NumRec	19.6120	< 2.2e-16	0.0355	0.4884	Non-normal	Unimodal
Arthropoda -Dep-NumSpe	0.41693	0.3133	0.0545	0.6159	Normal	Unimodal
Arthropoda -Dep_ES50	7.3286	< 2.2e-16	0.0254	0.9931	Non-normal	Unimodal
Mollusca-Lat-NumRec	3.7512	1.48e-09	0.0434	0.9031	Non-normal	Unimodal
Mollusca -Lat-NumSpe	0.7986	0.03464	0.0463	0.8595	Non-normal	Unimodal
Mollusca -Lat-ES50	39.4600	< 2.2e-16	0.0272	0.8526	Non-normal	Unimodal
Mollusca -Dep-NumRec	19.5500	< 2.2e-16	0.0536	0.0760	Non-normal	Unimodal
Mollusca -Dep-NumSpe	0.8032	0.03372	0.0535	0.6506	Non-normal	Unimodal
Mollusca -Dep_ES50	7.9618	< 2.2e-16	0.0333	0.9294	Non-normal	Unimodal

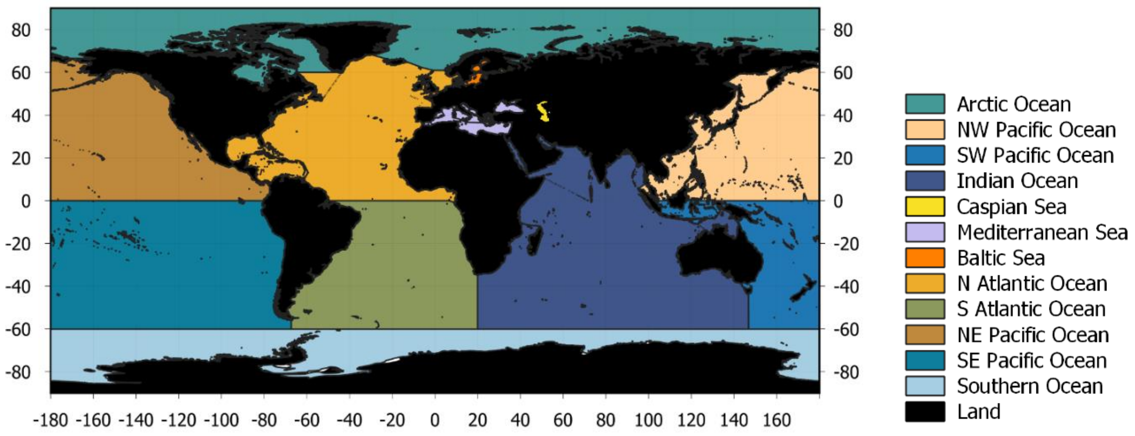

Supplementary Figure 1. The study area encompasses the world's oceans and enclosed seas, including nine oceanic regions, the Arctic Ocean, NW Pacific Ocean, SW Pacific Ocean, Indian Ocean, N Atlantic Ocean, S Atlantic Ocean, NE Pacific Ocean, SE Pacific Ocean, and the Southern Ocean, as well as three enclosed seas: the Caspian Sea, Mediterranean Sea, and the Baltic Sea, each represented by distinct colours.

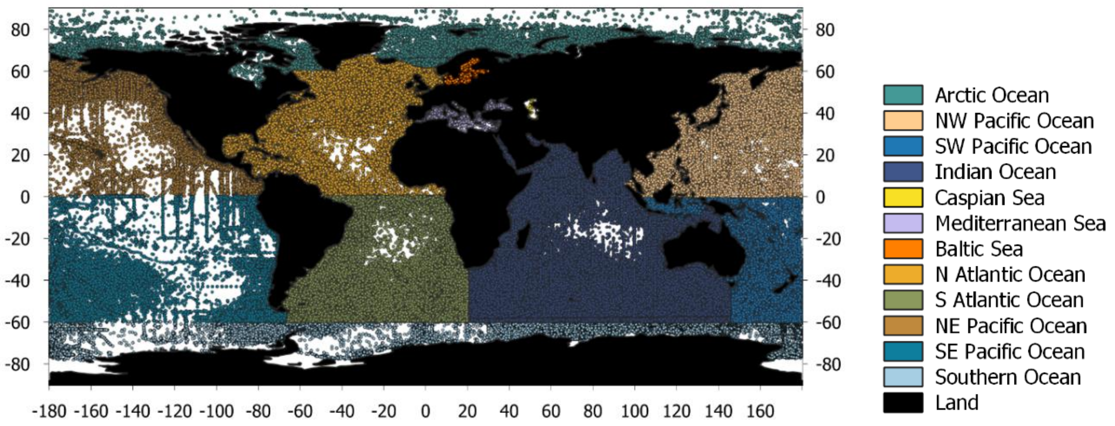

Supplementary Figure 2. The points show global occurrence data (n = 47,995,228 records), representing 184,141 accepted marine animal species after quality control. Colours correspond to the world's oceans and enclosed seas.

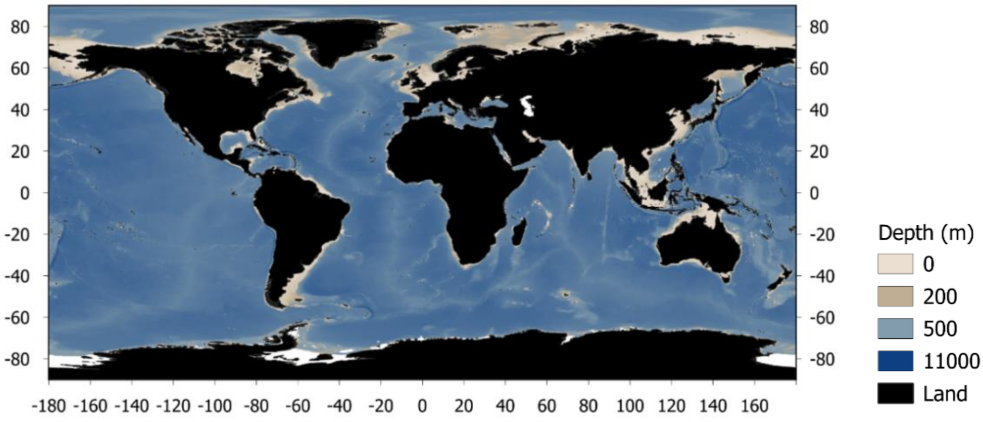

Supplementary Figure 3. Ocean depth was classified into three habitat zones: shallow (0–200 m), mesopelagic (200–500 m), and deep (>500 m), each represented by different colours. All occurrence records with available depth information were assigned to these three categories throughout the manuscript and in the presented maps.

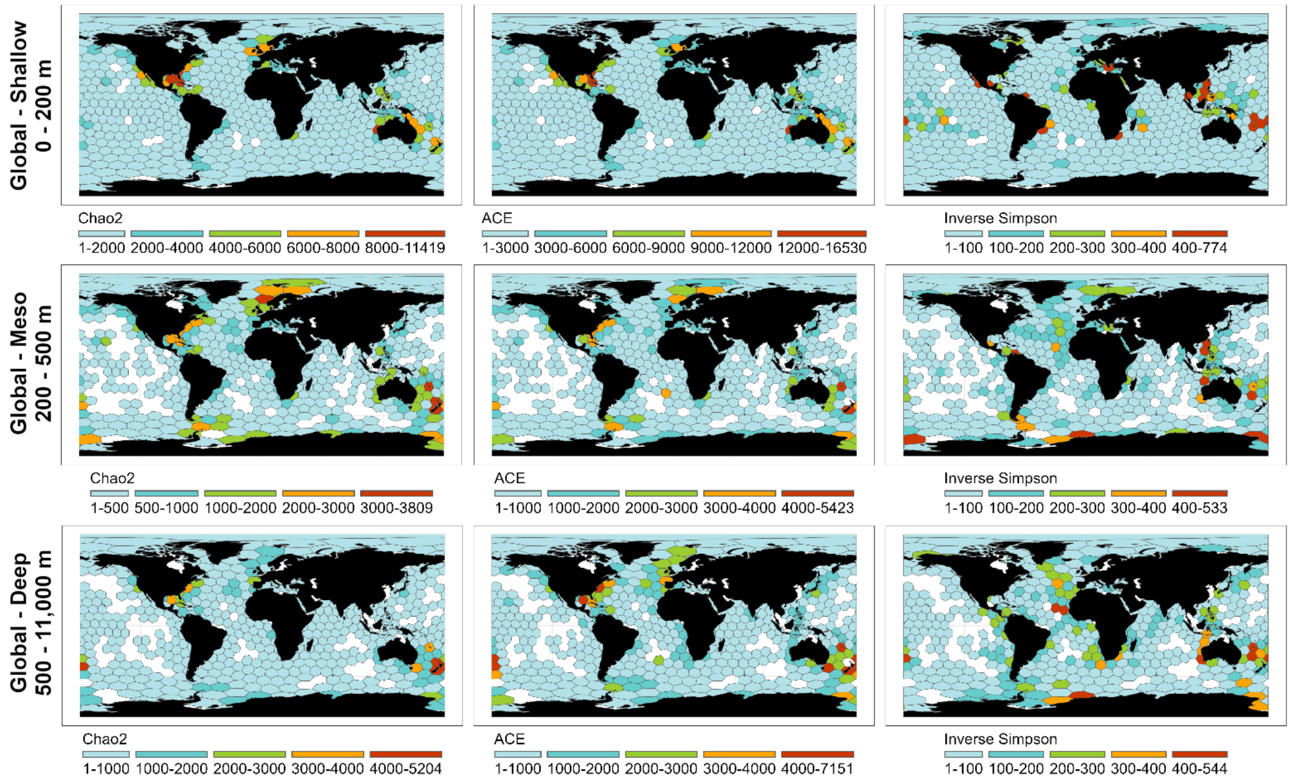

Supplementary Figure 4. Chao2 (based on presence-absence data), Abundance-based Coverage Estimator (ACE), and Inverse Simpson (species evenness) per hexagonal cells ranged from ca. 660,000 to 800,000 km2 for shallow (0-200 m), mesopelagic (>200-500 m), and deep (>500 m) habitats. The empty hexagons show areas with no information.

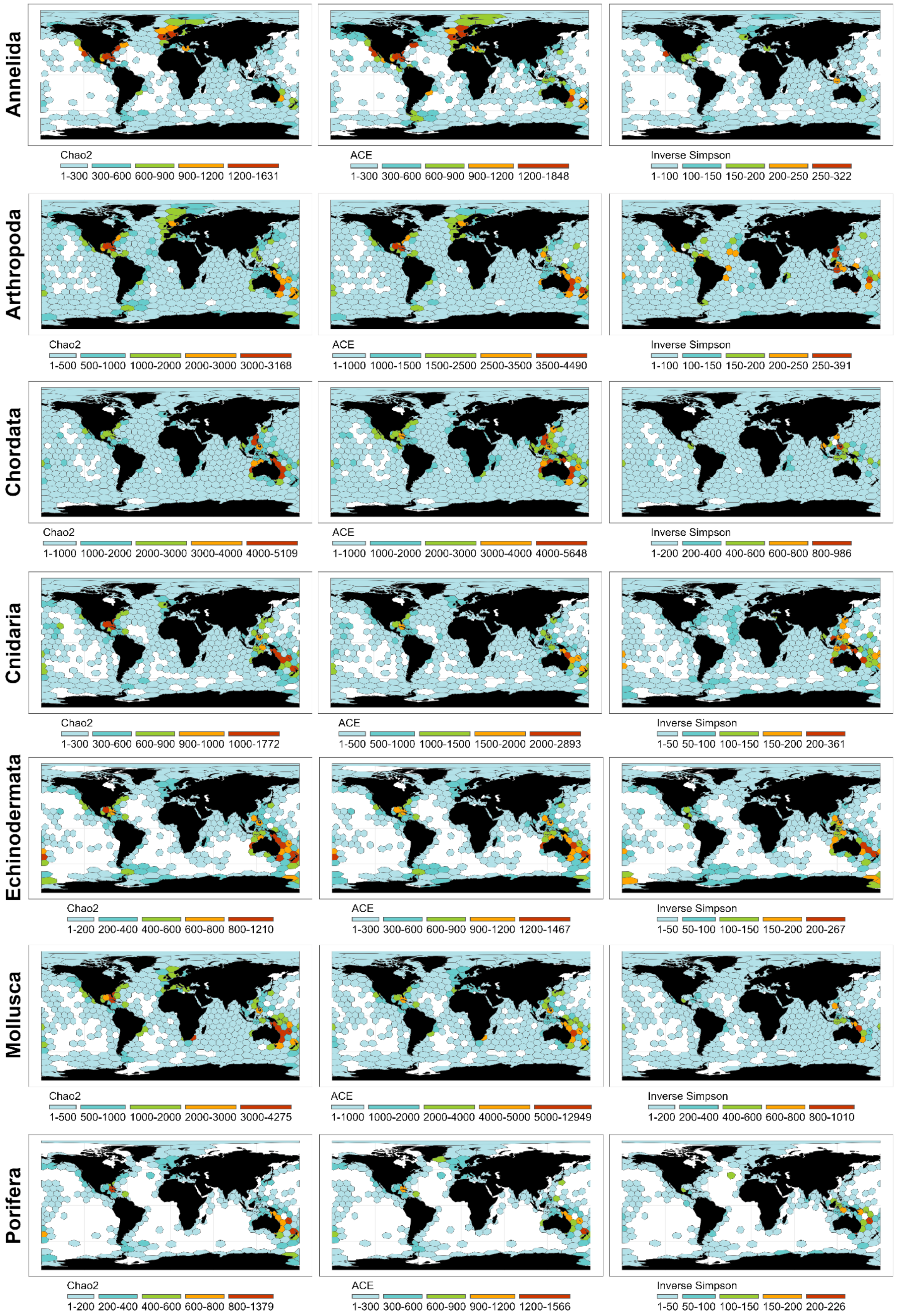

Supplementary Figure 5. Chao2 (based on presence-absence data), Abundance-based Coverage Estimator (ACE), and Inverse Simpson (species evenness) per hexagonal cells ranged from ca. 660,000 to 800,000 km2 for seven dominant taxa, including Annelida, Arthropoda, Chordata, Cnidaria, Echinodermata, Mollusca, and Porifera. The empty hexagons show areas with no information.

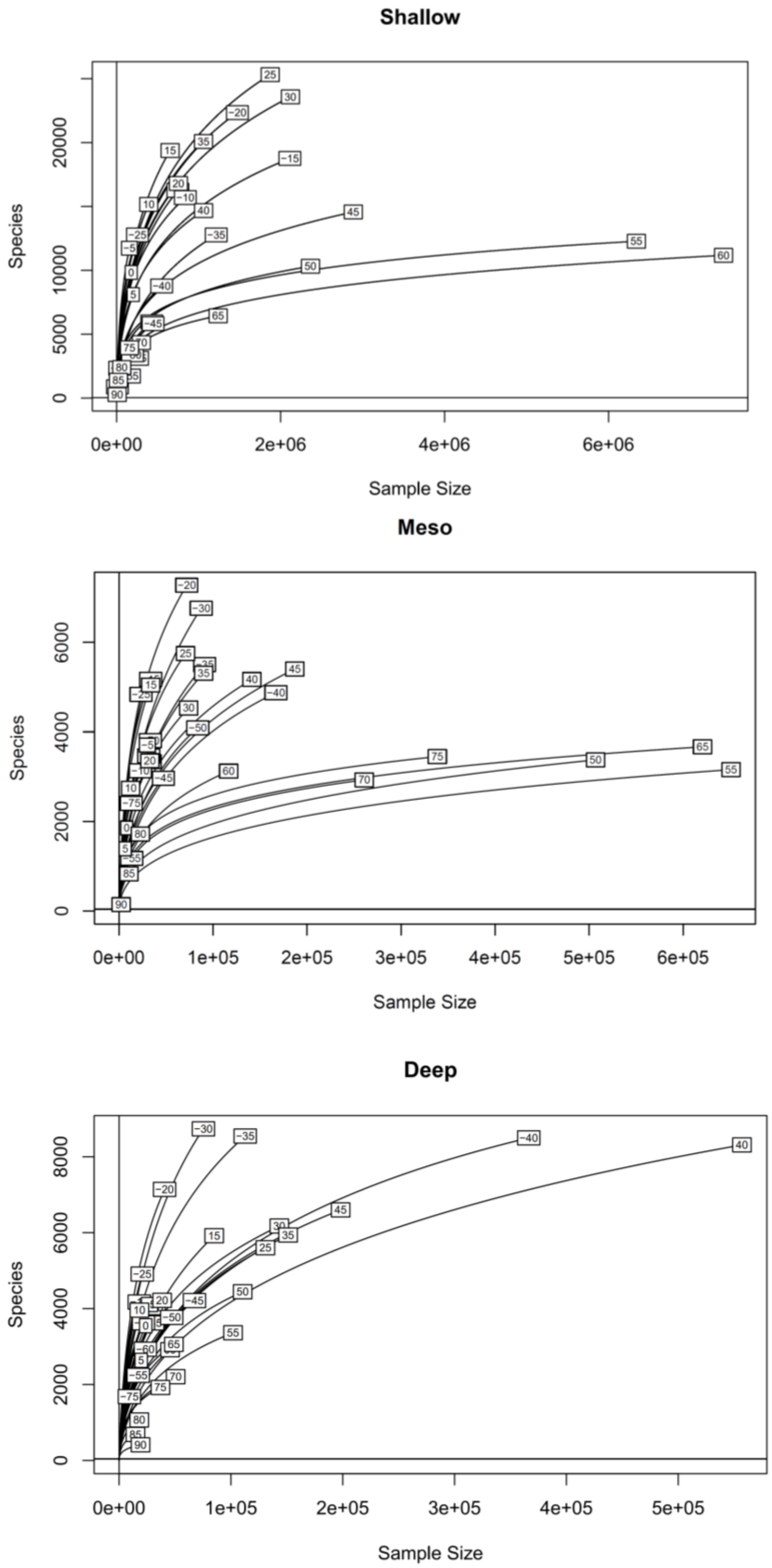

Supplementary Figure 6. Sample-based rarefaction curves showing the number of species as a function of sample size per 5° latitudinal bands (labelled -90-90) for shallow (0-200 m), mesopelagic (>200-500 m), and deep (>500 m) habitats. The southern latitudes expect many more species to be discovered with accelerating sampling efforts. In the shallow and mesopelagic depths, in latitudes from 55° to 65°, accelerating higher sampling efforts will not necessarily end up discovering new species, but it does in all other latitudes. In the deep sea, many species are waiting to be discovered in all latitudes.

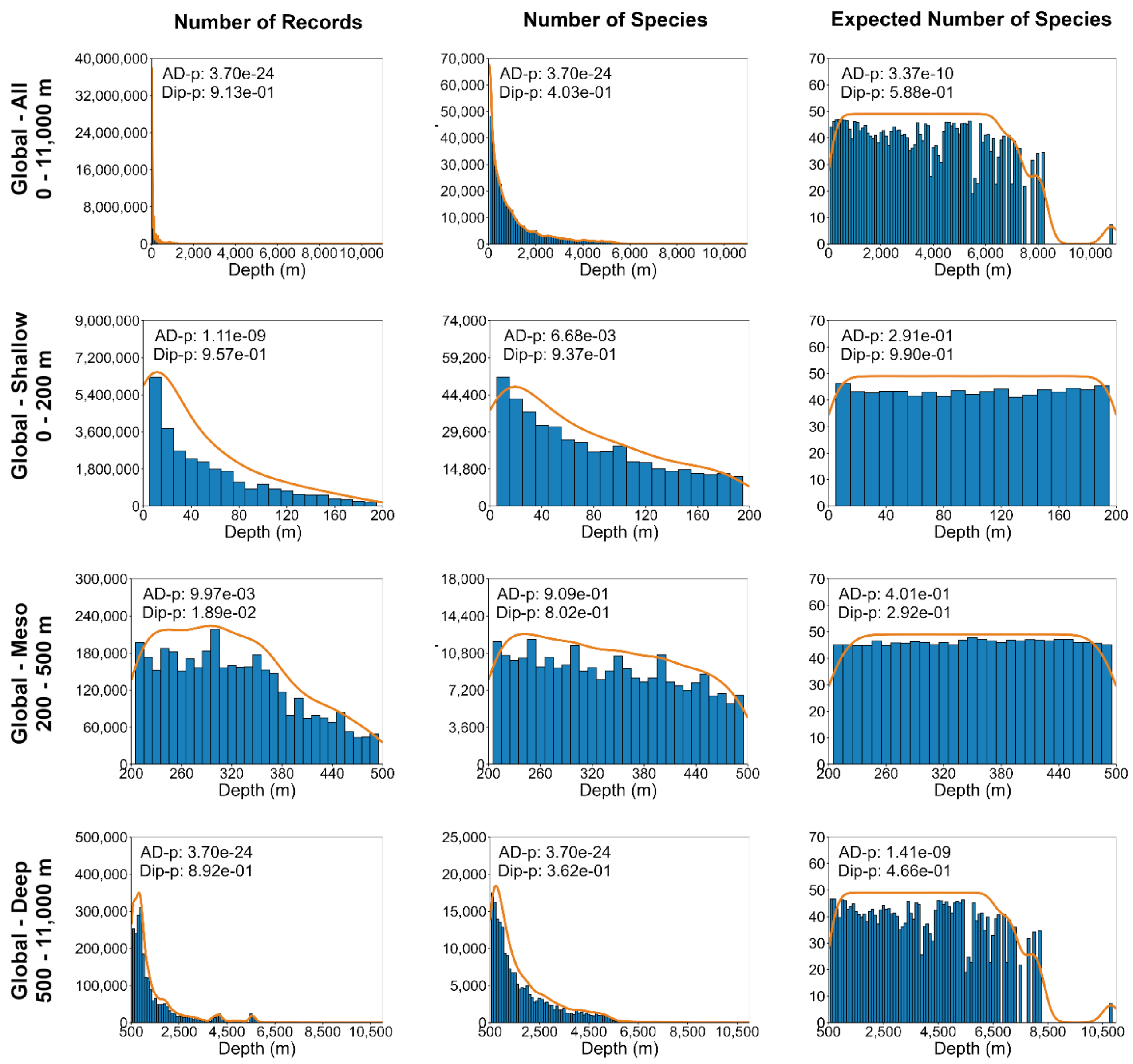

Supplementary Figure 7. Bathymetric species richness gradients for all marine species. Blue bars show the number of occurrence records, observed species, and expected species (ES50) across depth zones, global marine (0-11,000 m), shallow (0-200 m), mesopelagic (>200-500 m), and deep (>500 m) habitats. The orange line shows the kernel density estimation (KDE). Anderson–Darling (AD) and Hartigan's Dip (Dip) tests were used to assess normality and modality, respectively.

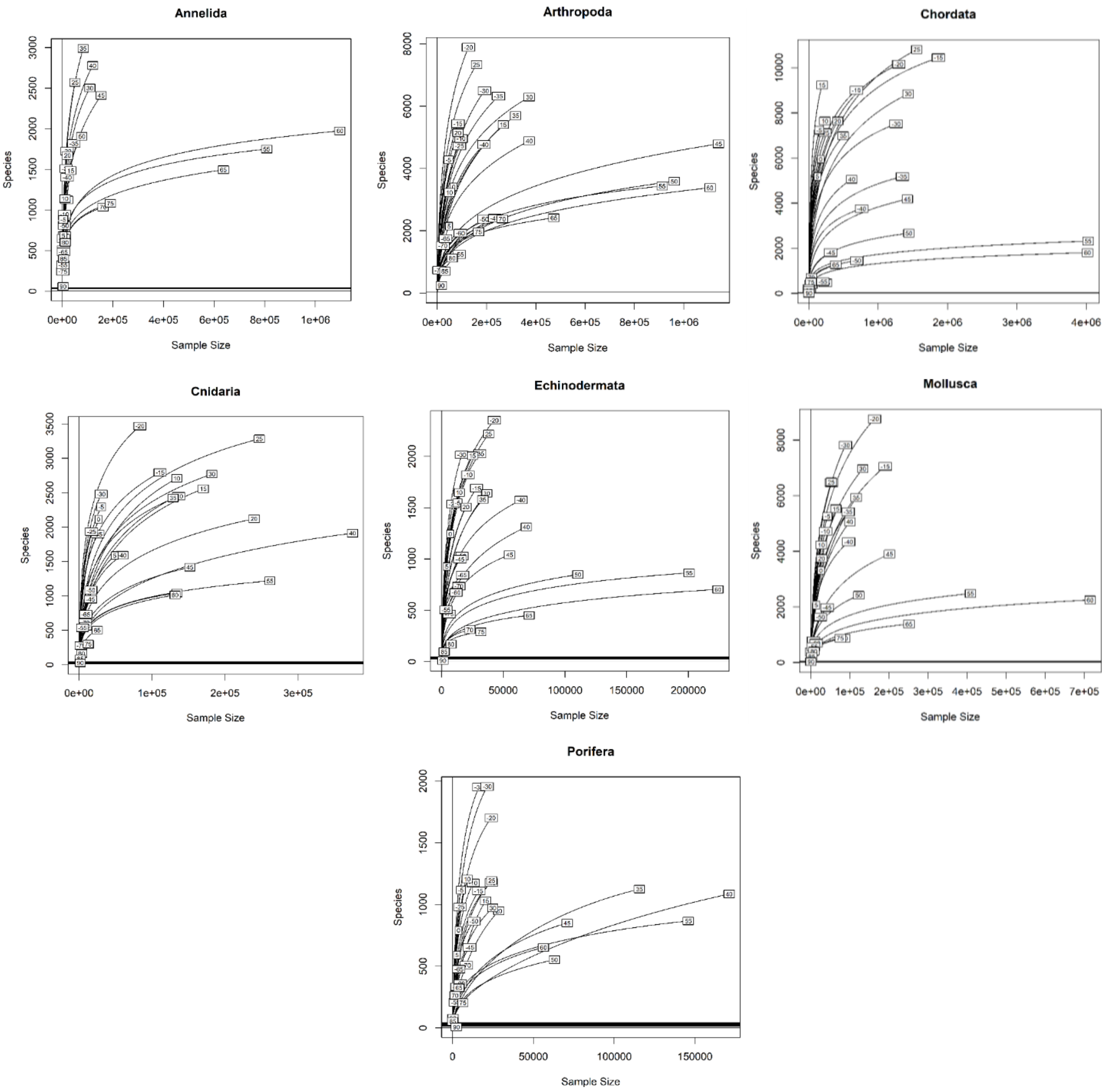

Supplementary Figure 8. Sample-based rarefaction curves showing the number of species as a function of sample size per 5° latitudinal bands (labelled -90-90) for seven taxa, including Annelida, Arthropoda, Chordata, Cnidaria, Echinodermata, Mollusca, and Porifera. The southern latitudes expect many more species to be discovered with accelerating sampling efforts. In all taxa, in latitudes from 40° to 60°, accelerating higher sampling efforts will not necessarily end up discovering new species, but it does in all other latitudes.

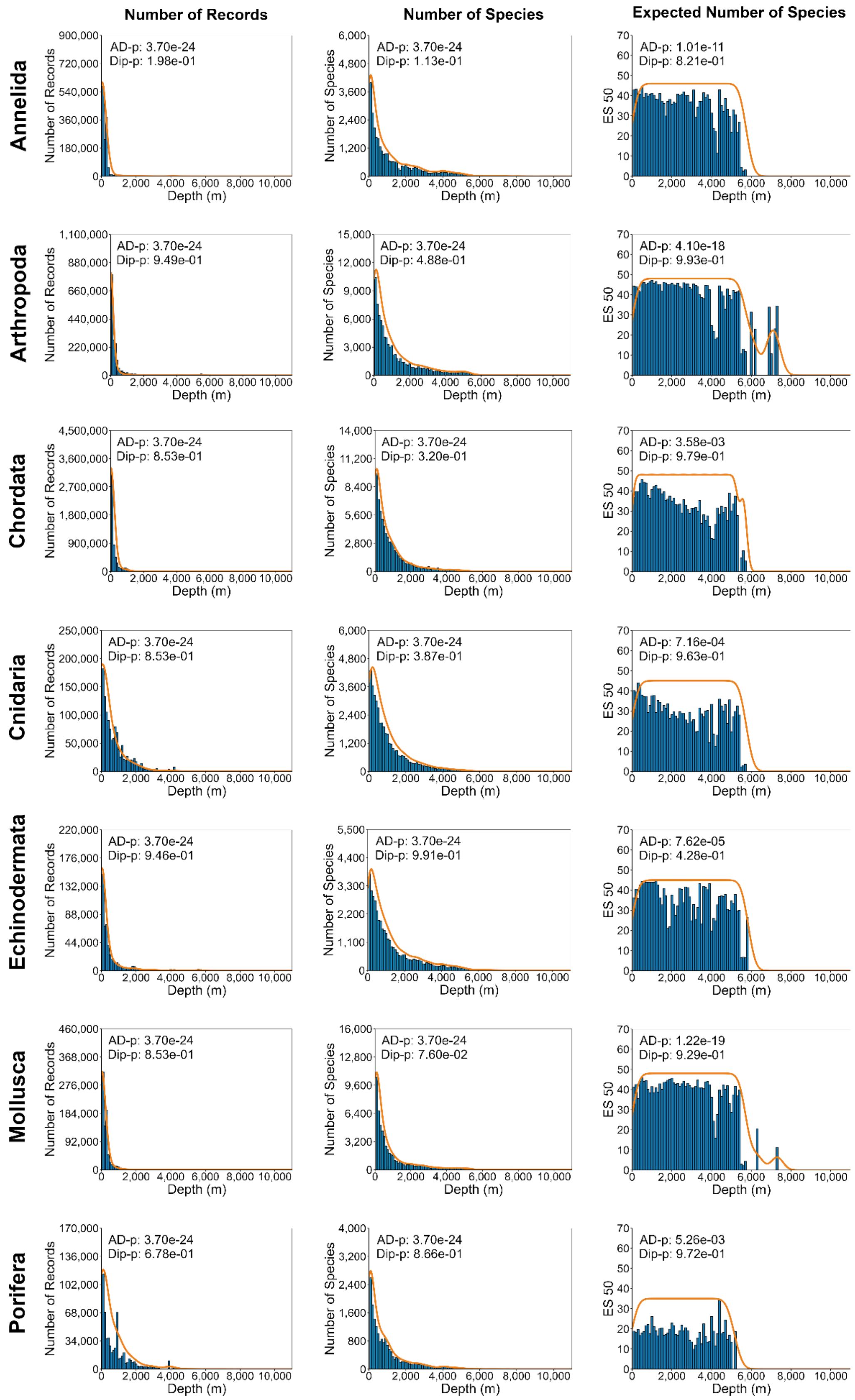

Supplementary Figure 9. Bathymetric species richness gradients for seven taxa, including Annelida, Arthropoda, Chordata, Cnidaria, Echinodermata, Mollusca, and Porifera. Blue bars show the number of occurrence records, observed species, and expected species (ES50) against 100 m depth intervals. The orange line shows the kernel density estimation (KDE). Anderson–Darling (AD) and Hartigan's Dip (Dip) tests were used to assess normality and modality, respectively.

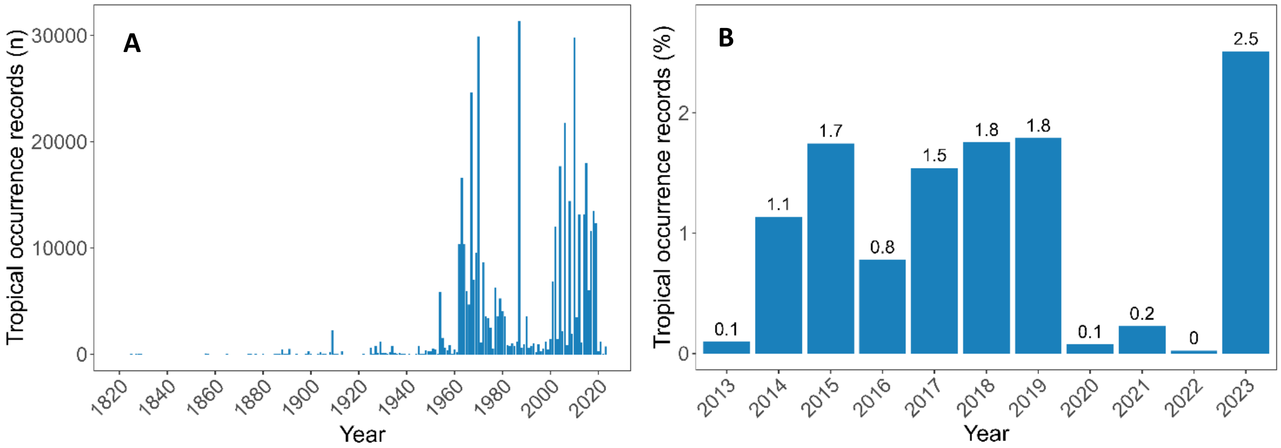

Supplementary Figure 10. A: Blue bars show the total number of marine animal occurrence records from central tropical latitudes (−5° to 5°) mobilised to the Ocean Biodiversity Information System per year. B: Blue bars show the percentage of marine animal occurrence records from central tropical latitudes (−5° to 5°) relative to the total number of records mobilised to the Ocean Biodiversity Information System between 2013 and 2023.

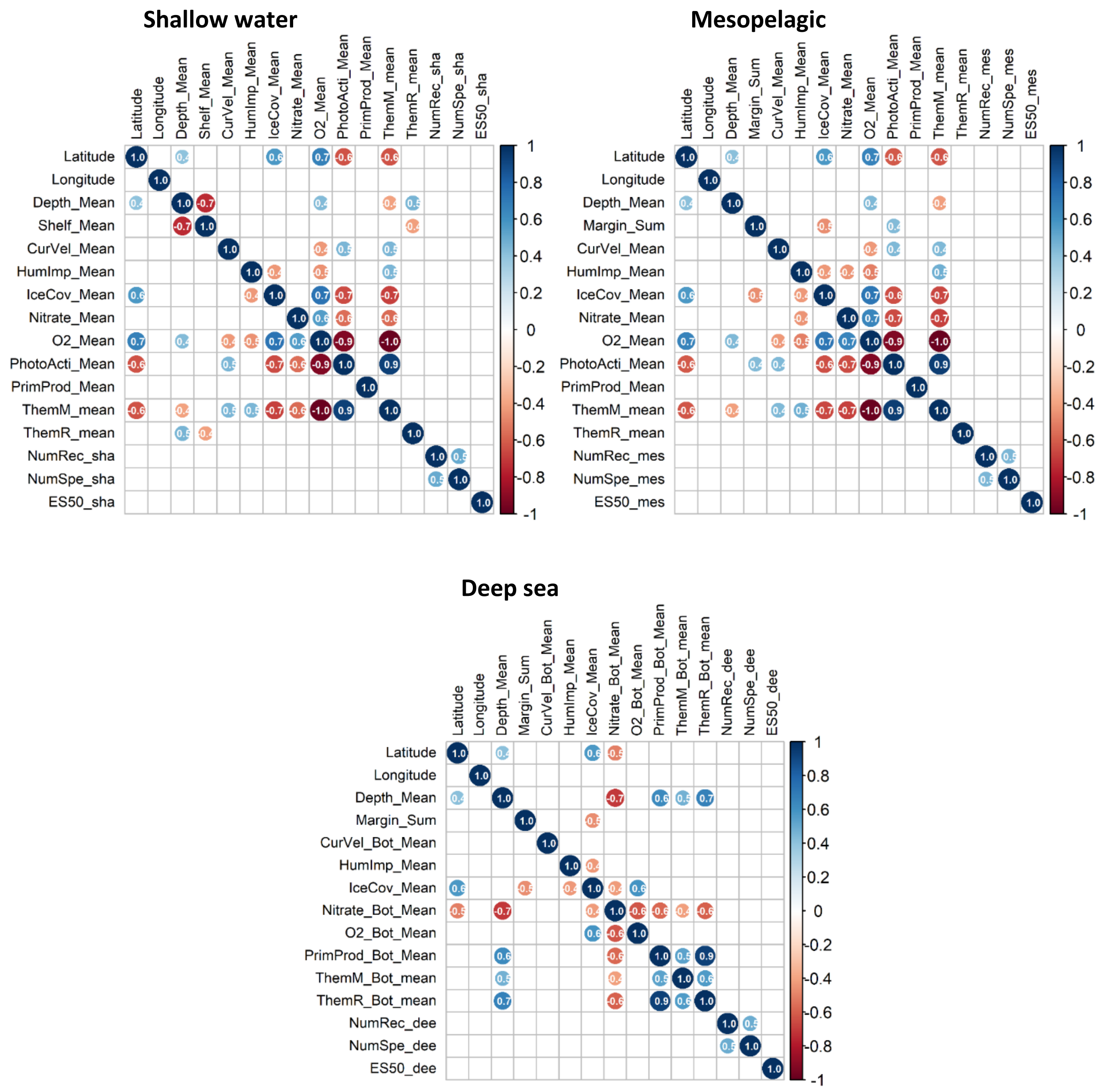

Supplementary Figure 11. Correlation matrix illustrating the relationships among all ecological variables (pelagic and benthic layers) extracted from BioOracle. Before modelling, collinearity among predictors was assessed using pairwise correlations. Dissolved oxygen (O_2) showed a strong correlation with mean temperature in both shallow-water and mesopelagic habitats, indicating potential multicollinearity. To reduce this issue and improve model interpretability, O_2 was excluded from the General Additive Model analyses for these layers. In contrast, this strong correlation was not observed in the deep-sea environment; therefore, O_2 was retained as a predictor in the deep-sea analyses. Correlation coefficients range from −1 (strong negative relationship) to 1 (strong positive relationship), with values represented by a colour gradient.

Supplementary Table 5. The GLM output results for the number of species per 5° latitudinal bands in shallow waters (0-200 m). AIC: Akaike Information Criterion, rel.LL: relative log-likelihood.

	Model	AIC	deltaAIC	rel.LL	weights	Cumulative.Weight
TemMea	TemMea	29138.9	0	1	1	1
Nitrate	Nitrate	44067.77	14928.88	0	0	1
Depth	Depth	56466.94	27328.05	0	0	1
IceCov	IceCov	57155.87	28016.97	0	0	1
HumImp	HumImp	59225.11	30086.22	0	0	1
ConShe	ConShe	75759.25	46620.36	0	0	1
PriPro	PriPro	95156.83	66017.94	0	0	1
Area	Area	107022.8	77883.89	0	0	1
CurVel	CurVel	107207.3	78068.4	0	0	1
NumRec	NumRec	107493.7	78354.8	0	0	1
Intercept	Intercept	113144.7	84005.81	0	0	1

Supplementary Table 6. The GLM output results for the expected species (ES50) per 5° latitudinal bands in shallow waters (0-200 m). AIC: Akaike Information Criterion, rel.LL: relative log-likelihood.

	Model	AIC	deltaAIC	rel.LL	weights	Cumulative.Weight
Nitrate	Nitrate	177.6473	0	1	0.617942	0.617942
TemMea	TemMea	180.3178	2.670493	0.263093	0.162576	0.780518
Area	Area	182.2458	4.598521	0.100333	0.062	0.842518
Intercept	Intercept	182.6947	5.04739	0.080163	0.049536	0.892054
HumImp	HumImp	183.612	5.964638	0.050675	0.031314	0.923368
PriPro	PriPro	184.7568	7.109483	0.028589	0.017666	0.941035
IceCov	IceCov	184.8564	7.209055	0.0272	0.016808	0.957843
NumRec	NumRec	184.9903	7.342943	0.025439	0.01572	0.973563
Depth	Depth	184.9923	7.345009	0.025413	0.015704	0.989266
CurVel	CurVel	186.8985	9.25114	0.009798	0.006055	0.995321
ConShe	ConShe	187.4139	9.766582	0.007572	0.004679	1

Supplementary Table 7. The GLM output results for the number of species per 5° latitudinal bands in mesopelagic habitats (>200-500 m). Akaike Information Criterion, rel.LL: relative log-likelihood.

	Model	AIC	deltaAIC	rel.LL	weights	Cumulative.Weight
HumImp	HumImp	16167.67	0	1	1	1
TemMea	TemMea	17187.78	1020.117	3.05E-222	3.05E-222	1
Depth	Depth	17353.33	1185.662	3.44E-258	3.44E-258	1
Nitrate	Nitrate	17400.97	1233.308	1.55E-268	1.55E-268	1
Area	Area	18908.72	2741.055	0	0	1
CurVel	CurVel	19128.79	2961.12	0	0	1
ConMar	ConMar	19349.79	3182.125	0	0	1
NumRec	NumRec	21104.27	4936.606	0	0	1
PriPro	PriPro	21105.08	4937.418	0	0	1
Intercept	Intercept	21108.5	4940.834	0	0	1

Supplementary Table 8. The GLM output results for the ES50 per 5° latitudinal bands in mesopelagic habitats (>200-500 m). Akaike Information Criterion, rel.LL: relative log-likelihood.

	Model	AIC	deltaAIC	rel.LL	weights	Cumulative.Weight
Nitrate	Nitrate	174.9318	0	1	0.220288	0.220288
TemMea	TemMea	175.2166	0.284773	0.867286	0.191052	0.41134
Intercept	Intercept	175.7642	0.832418	0.659542	0.145289	0.556629
CurVel	CurVel	176.7684	1.836571	0.399203	0.087939	0.644568
Area	Area	177.0926	2.160776	0.339464	0.07478	0.719348
NumRec	NumRec	177.1099	2.178045	0.336545	0.074137	0.793485
PriPro	PriPro	177.2444	2.312602	0.314648	0.069313	0.862798
HumIpm	HumIpm	178.074	3.142189	0.207818	0.04578	0.908578
Depth	Depth	178.0768	3.145005	0.207525	0.045715	0.954293
ConMar	ConMar	178.0772	3.145359	0.207488	0.045707	1

Supplementary Table 9. The GLM output results for the number of species per 5° latitudinal bands in deep-sea habitats (>500 m). Akaike Information Criterion, rel.LL: relative log-likelihood.

	Model	AIC	deltaAIC	rel.LL	weights	Cumulative.Weight
Nitrate	Nitrate	12335.95	0	1	1	1
Depth	Depth	13463.65	1127.699	1.33E-245	1.33E-245	1
O2	O2	13917.83	1581.877	0	0	1
HumImp	HumImp	15368.42	3032.467	0	0	1
PriPro	PriPro	17915.94	5579.988	0	0	1
TemMea	TemMea	19698.91	7362.963	0	0	1
CurVel	CurVel	19749.31	7413.361	0	0	1
Area	Area	20583.68	8247.731	0	0	1
ConMar	ConMar	22072.46	9736.506	0	0	1
NumRec	NumRec	22253.75	9917.797	0	0	1
Intercept	Intercept	32122.85	19786.9	0	0	1

Supplementary Table 10. The GLM output results for the ES50 per 5° latitudinal bands in deep-sea habitats (>500 m). Akaike Information Criterion, rel.LL: relative log-likelihood.

	Model	AIC	deltaAIC	rel.LL	weights	Cumulative.Weight
Intercept	Intercept	173.0017	0	1	0.214575	0.214575
NumRec	NumRec	174.096	1.094281	0.578602	0.124154	0.338729
Nitrate	Nitrate	174.5975	1.595752	0.450284	0.09662	0.435349
O2	O2	174.9917	1.989959	0.369731	0.079335	0.514684
Depth	Depth	175.2141	2.212416	0.330811	0.070984	0.585668
CurVel	CurVel	175.2231	2.221421	0.329325	0.070665	0.656333
TemMea	TemMea	175.2432	2.241509	0.326034	0.069959	0.726291
Area	Area	175.2765	2.274807	0.32065	0.068804	0.795095
ConMar	ConMar	175.2786	2.276835	0.320325	0.068734	0.863829
PriPro	PriPro	175.2808	2.279095	0.319964	0.068656	0.932485
HumImp	HumImp	175.3143	2.312623	0.314645	0.067515	1

Supplementary Note 1

Markdown_GLM_Shallow.R

hsaeedi

2025-06-07

```
library(rmarkdown) # for saving the code and the images, markdown

## Warning: package 'rmarkdown' was built under R version 4.3.3

library(readxl)

## Warning: package 'readxl' was built under R version 4.3.3

library(openxlsx)

## Warning: package 'openxlsx' was built under R version 4.3.3

library(tidyverse)

## Warning: package 'tidyverse' was built under R version 4.3.3

## Warning: package 'ggplot2' was built under R version 4.3.3

## Warning: package 'tibble' was built under R version 4.3.3

## Warning: package 'tidyr' was built under R version 4.3.3

## Warning: package 'readr' was built under R version 4.3.3

## Warning: package 'purrr' was built under R version 4.3.3

## Warning: package 'dplyr' was built under R version 4.3.3

## Warning: package 'stringr' was built under R version 4.3.3

## Warning: package 'forcats' was built under R version 4.3.3

## Warning: package 'lubridate' was built under R version 4.3.3

## ── Attaching core tidyverse packages ──────────────────────── tidyvers
e 2.0.0 ──
## ✔ dplyr     1.1.4     ✔ readr     2.1.5
## ✔ forcats   1.0.0     ✔ stringr   1.5.1
## ✔ ggplot2   3.5.1     ✔ tibble    3.2.1
## ✔ lubridate 1.9.3     ✔ tidyr     1.3.1
## ✔ purrr     1.0.2
## ── Conflicts ────────────────────────────────────────── tidyverse_conf
licts() ──
## ✖ dplyr::filter() masks stats::filter()
## ✖ dplyr::lag()    masks stats::lag()
```

```
## ℹ Use the conflicted package (<http://conflicted.r-lib.org/>) to for
ce all conflicts to become errors

library(sf)

## Warning: package 'sf' was built under R version 4.3.3

## Linking to GEOS 3.11.2, GDAL 3.8.2, PROJ 9.3.1; sf_use_s2() is TRUE

library(vegan)

## Warning: package 'vegan' was built under R version 4.3.3

## Loading required package: permute

## Warning: package 'permute' was built under R version 4.3.3

## Loading required package: lattice
## This is vegan 2.6-4

library(pvclust)

## Warning: package 'pvclust' was built under R version 4.3.3

library(dplyr)
library(ggplot2)
library(robis)

## Warning: package 'robis' was built under R version 4.3.3

library(obistools)
library(nortest) # for Anderson-Darling test
library(stringi) # for encoding UTF-8

## Warning: package 'stringi' was built under R version 4.3.3

library(corrplot)

## Warning: package 'corrplot' was built under R version 4.3.3

## corrplot 0.94 loaded

library(mgcv)

## Loading required package: nlme
##
## Attaching package: 'nlme'
##
## The following object is masked from 'package:dplyr':
##
##     collapse
##
## This is mgcv 1.9-0. For overview type 'help("mgcv-package")'.

library(ggeffects)

## Warning: package 'ggeffects' was built under R version 4.3.3
```

```r
library(DHARMa) #simulations package gam

## Warning: package 'DHARMa' was built under R version 4.3.3

## Warning in check_dep_version(): ABI version mismatch:
## lme4 was built with Matrix ABI version 1
## Current Matrix ABI version is 0
## Please re-install lme4 from source or restore original 'Matrix' packag
e

## This is DHARMa 0.4.6. For overview type '?DHARMa'. For recent changes,
type news(package = 'DHARMa')

library(knitr)

## Warning: package 'knitr' was built under R version 4.3.3

library(qpcR)

## Warning: package 'qpcR' was built under R version 4.3.3

## Loading required package: MASS
##
## Attaching package: 'MASS'
##
## The following object is masked from 'package:robis':
##
##     area
##
## The following object is masked from 'package:dplyr':
##
##     select
##
## Loading required package: minpack.lm

## Warning: package 'minpack.lm' was built under R version 4.3.3

## Loading required package: rgl

## Warning: package 'rgl' was built under R version 4.3.3

## Loading required package: robustbase

## Warning: package 'robustbase' was built under R version 4.3.3

## Loading required package: Matrix
##
## Attaching package: 'Matrix'
##
## The following objects are masked from 'package:tidyr':
##
##     expand, pack, unpack

# ===================================
#                 SHALLOW-NUMSPe-GLM
```

```
# ===================================

#Species Counts and Environment, 5 degree bands
Ecological_Data_Global <- read.csv("Ecological_Data_Global.csv", sep = ";
")
summary(Ecological_Data_Global)

##         id            left            top              right          bot
tom
##  Min.   : 1.00   Min.   :-180   Min.   :-85.00   Min.   :180   Min.
:-90.00
##  1st Qu.: 9.75   1st Qu.:-180   1st Qu.:-41.25   1st Qu.:180   1st Qu.
:-46.25
##  Median :18.50   Median :-180   Median :  2.50   Median :180   Median
: -2.50
##  Mean   :18.50   Mean   :-180   Mean   :  2.50   Mean   :180   Mean
: -2.50
##  3rd Qu.:27.25   3rd Qu.:-180   3rd Qu.: 46.25   3rd Qu.:180   3rd Qu.
: 41.25
##  Max.   :36.00   Max.   :-180   Max.   : 90.00   Max.   :180   Max.
: 85.00
##
##  Bathy_Max_Mean    Bathy_Mean_Mean   Bathy_Min_Mean    Chl_Mean_Mean
##  Min.   :-4278.5   Min.   :-4194.3   Min.   :-4117.6   Min.   :0.09031
##  1st Qu.:-4058.1   1st Qu.:-3970.8   1st Qu.:-3889.3   1st Qu.:0.13366
##  Median :-3874.8   Median :-3790.1   Median :-3702.5   Median :0.25032
##  Mean   :-3292.2   Mean   :-3225.1   Mean   :-3154.7   Mean   :0.26540
##  3rd Qu.:-2944.1   3rd Qu.:-2883.2   3rd Qu.:-2820.7   3rd Qu.:0.37041
##  Max.   : -761.9   Max.   : -741.7   Max.   : -721.3   Max.   :0.50726
##  NA's   :2         NA's   :2         NA's   :2         NA's   :2
##    Margin_Sum            Bathy_Max             Shelf_Sum          CurVel_
Mean
##  Min.   :-322305404   Min.   :-3.072e+09   Min.   :-9362904   Min.   :
0.02525
##  1st Qu.:-152911004   1st Qu.:-2.251e+09   1st Qu.:-1834335   1st Qu.:
0.04832
##  Median :-110339888   Median :-2.014e+09   Median :-1300462   Median :
0.06613
##  Mean   :-113889802   Mean   :-1.666e+09   Mean   :-1872021   Mean   :
0.08048
##  3rd Qu.: -70897881   3rd Qu.:-7.886e+08   3rd Qu.: -705476   3rd Qu.:
0.11028
##  Max.   :         0   Max.   : 0.000e+00   Max.   :       0   Max.   :
0.21723
##                                                                NA's   :
2
##  CurVel_Bot_Mean    Diff_Atte_Mean      Depth_Mean         HumImp_Sum
##  Min.   :0.002405   Min.   :0.02947   Min.   :-4204.0   Min.   :
0
##  1st Qu.:0.014127   1st Qu.:0.06059   1st Qu.:-3962.4   1st Qu.:  8151
342
```

```
##  Median :0.017665   Median :0.07062   Median :-3776.1   Median : 56012
445
##  Mean   :0.018497   Mean   :0.07477   Mean   :-3204.4   Mean   : 68495
091
##  3rd Qu.:0.020965   3rd Qu.:0.08153   3rd Qu.:-2826.0   3rd Qu.:103706
926
##  Max.   :0.045673   Max.   :0.12402   Max.   : -655.6   Max.   :195804
477
##  NA's   :2          NA's   :2         NA's   :2         NA's   :4
##   HumImp_Mean     Ice_Cover_Sum       IceCov_Mean        Ice_Tick_Mean
##  Min.   : 1.342   Min.   :     0.0   Min.   :0.0000000   Min.   :0.000
0000
##  1st Qu.: 8.030   1st Qu.:     0.0   1st Qu.:0.0000000   1st Qu.:0.000
0000
##  Median :10.361   Median :     0.5   Median :0.0000077   Median :0.000
0115
##  Mean   : 9.725   Mean   : 81161.0   Mean   :0.1808575   Mean   :0.323
9475
##  3rd Qu.:12.446   3rd Qu.: 44888.8   3rd Qu.:0.2563673   3rd Qu.:0.286
3370
##  Max.   :18.138   Max.   :696353.9   Max.   :0.9671582   Max.   :2.458
3182
##  NA's   :7                           NA's   :2           NA's   :2
##   MixLay_Mean     PhotoActi_Mean   PrimProd_Mean    Salinity_Mean
##  Min.   : 19.95   Min.   : 3.877   Min.   :0.3574   Min.   :30.31
##  1st Qu.: 30.85   1st Qu.:24.399   1st Qu.:1.3064   1st Qu.:33.11
##  Median : 40.91   Median :28.955   Median :1.5059   Median :34.13
##  Mean   : 45.60   Mean   :32.202   Mean   :1.5172   Mean   :33.96
##  3rd Qu.: 56.22   3rd Qu.:43.915   3rd Qu.:1.6973   3rd Qu.:35.00
##  Max.   :103.59   Max.   :48.163   Max.   :2.3878   Max.   :35.67
##  NA's   :2        NA's   :2        NA's   :2        NA's   :2
##  Silicate_Mean      ThemM_Mean      Them_Max_Mean     Them_Max_Max
##  Min.   : 1.556   Min.   :-1.721   Min.   :-1.524   Min.   :-0.8473
##  1st Qu.: 2.356   1st Qu.: 2.327   1st Qu.: 6.581   1st Qu.:14.8983
##  Median : 5.867   Median :13.104   Median :18.786   Median :27.7568
##  Mean   :16.058   Mean   :13.340   Mean   :16.923   Mean   :23.2399
##  3rd Qu.:13.556   3rd Qu.:25.013   3rd Qu.:27.983   3rd Qu.:32.6277
##  Max.   :83.166   Max.   :28.215   Max.   :29.775   Max.   :36.4851
##  NA's   :2        NA's   :2        NA's   :2        NA's   :2
##  Temp_Min_Mean       Temp_Min_Min      ThemR_Mean     Them_Range_Max
##  Min.   :-1.95401   Min.   :-2.000   Min.   : 0.297   Min.   : 0.7454
##  1st Qu.:-0.01615   1st Qu.:-2.000   1st Qu.: 4.105   1st Qu.: 9.4402
##  Median : 8.38265   Median :-1.030   Median : 5.556   Median :11.1749
##  Mean   :10.64040   Mean   : 4.492   Mean   : 6.284   Mean   :12.6015
##  3rd Qu.:21.91526   3rd Qu.:11.822   3rd Qu.: 8.375   3rd Qu.:16.9521
##  Max.   :26.38238   Max.   :19.556   Max.   :13.863   Max.   :24.1779
##  NA's   :2          NA's   :2        NA's   :2        NA's   :2
##     Area_Sum            O2_Mean       Nitrate_Mean     Nitrate_Bot_Mea
n
##  Min.   :1.020e+11   Min.   :203.1   Min.   : 0.1658   Min.   :10.42
##  1st Qu.:6.495e+11   1st Qu.:212.0   1st Qu.: 0.5914   1st Qu.:26.76
```

```
##  Median :9.760e+11   Median :268.6   Median : 4.1394   Median :31.54  
##  Mean   :9.857e+11   Mean   :273.6   Mean   : 7.9129   Mean   :27.32  
##  3rd Qu.:1.422e+12   3rd Qu.:325.9   3rd Qu.: 9.5803   3rd Qu.:32.03  
##  Max.   :2.050e+12   Max.   :371.1   Max.   :29.3414   Max.   :32.78  
##                      NA's   :2       NA's   :2         NA's   :2      
##   O2_Bot_Mean    PrimProd_Bot_Mean Salinity_Bot_Mean ThemM_Bot_Mean  
##  Min.   :167.8   Min.   :0.01356   Min.   :32.22     Min.   :-1.0599  
##  1st Qu.:184.0   1st Qu.:0.04705   1st Qu.:34.61     1st Qu.: 0.2374  
##  Median :199.6   Median :0.09406   Median :34.69     Median : 2.0996  
##  Mean   :219.7   Mean   :0.14575   Mean   :34.47     Mean   : 1.7394  
##  3rd Qu.:257.0   3rd Qu.:0.16129   3rd Qu.:34.74     3rd Qu.: 2.9030  
##  Max.   :314.2   Max.   :0.68720   Max.   :35.10     Max.   : 3.6338  
##  NA's   :2       NA's   :2         NA's   :2         NA's   :2       
##  ThemR_Bot_Mean        NumSpe          NumRec            NumPhy     
##  Min.   :0.04462   Min.   :  494   Min.   :  28649   Min.   :17.00  
##  1st Qu.:0.44255   1st Qu.: 6122   1st Qu.: 315808   1st Qu.:24.00  
##  Median :0.53982   Median :16326   Median : 840696   Median :25.50  
##  Mean   :0.86086   Mean   :16924   Mean   :1411624   Mean   :25.26  
##  3rd Qu.:0.97004   3rd Qu.:25489   3rd Qu.:1893681   3rd Qu.:27.75  
##  Max.   :3.53683   Max.   :38304   Max.   :7566285   Max.   :30.00  
##  NA's   :2         NA's   :2       NA's   :2         NA's   :2      
##       ES50        NumSpe_Sha      NumRec_Sha        NumPhy_Sha   
##  Min.   :23.68   Min.   :  271   Min.   :   7056   Min.   :13.00  
##  1st Qu.:39.81   1st Qu.: 4037   1st Qu.: 182153   1st Qu.:21.00  
##  Median :42.21   Median :10746   Median : 496946   Median :23.00  
##  Mean   :41.19   Mean   :10657   Mean   :1115969   Mean   :22.76  
##  3rd Qu.:45.60   3rd Qu.:15559   3rd Qu.:1232565   3rd Qu.:26.00  
##  Max.   :48.28   Max.   :25317   Max.   :7402048   Max.   :29.00  
##  NA's   :2       NA's   :2       NA's   :2         NA's   :2      
##     ES50_Sha       NumSpe_Mes     NumRec_Mes       NumPhy_Mes   
##  Min.   :18.00   Min.   : 141   Min.   :  2391   Min.   :10.00  
##  1st Qu.:35.25   1st Qu.:2937   1st Qu.: 22966   1st Qu.:17.00  
##  Median :40.50   Median :3455   Median : 40485   Median :18.50  
##  Mean   :38.53   Mean   :3689   Mean   :116802   Mean   :18.29  
##  3rd Qu.:44.50   3rd Qu.:5003   3rd Qu.:110266   3rd Qu.:20.00  
##  Max.   :47.00   Max.   :7275   Max.   :650912   Max.   :23.00  
##  NA's   :2       NA's   :2      NA's   :2        NA's   :2      
##     ES50_Mes       NumSpe_Dee     NumRec_Dee       NumPhy_Dee   
##  Min.   :29.00   Min.   : 411   Min.   :  9251   Min.   :15.00  
##  1st Qu.:39.00   1st Qu.:2920   1st Qu.: 21008   1st Qu.:18.00  
##  Median :42.00   Median :3992   Median : 39600   Median :19.50  
##  Mean   :42.21   Mean   :4270   Mean   : 80043   Mean   :20.12  
##  3rd Qu.:47.00   3rd Qu.:5841   3rd Qu.: 97697   3rd Qu.:22.00  
##  Max.   :49.00   Max.   :8735   Max.   :556993   Max.   :27.00  
##  NA's   :2       NA's   :2      NA's   :2        NA's   :2      
##     ES50_Dee    
##  Min.   :29.00  
##  1st Qu.:40.00  
##  Median :42.50  
##  Mean   :42.53  
##  3rd Qu.:45.00  
```

```
## Max.   :49.00
## NA's   :2

#First we’re going to load in our data and then trim the data frame down to just the columns we need.
analysis.cols <- c("Shelf_Sum", "CurVel_Mean", "Depth_Mean", "HumImp_Mean", "IceCov_Mean", "PrimProd_Mean", "Area_Sum", "Nitrate_Mean",
                   "ThemM_Mean", "NumSpe_Sha","NumRec_Sha", "NumPhy_Sha", "ES50_Sha")
Ecological_Data_Global <- Ecological_Data_Global[,analysis.cols]
Ecological_Data_Global  <- Ecological_Data_Global [complete.cases(Ecological_Data_Global ),]

# Calculate the correlation matrix
corr_matrix <- cor(Ecological_Data_Global)

# Create the correlation plot with black font for text
corrplot(corr_matrix, tl.col = "black")
```

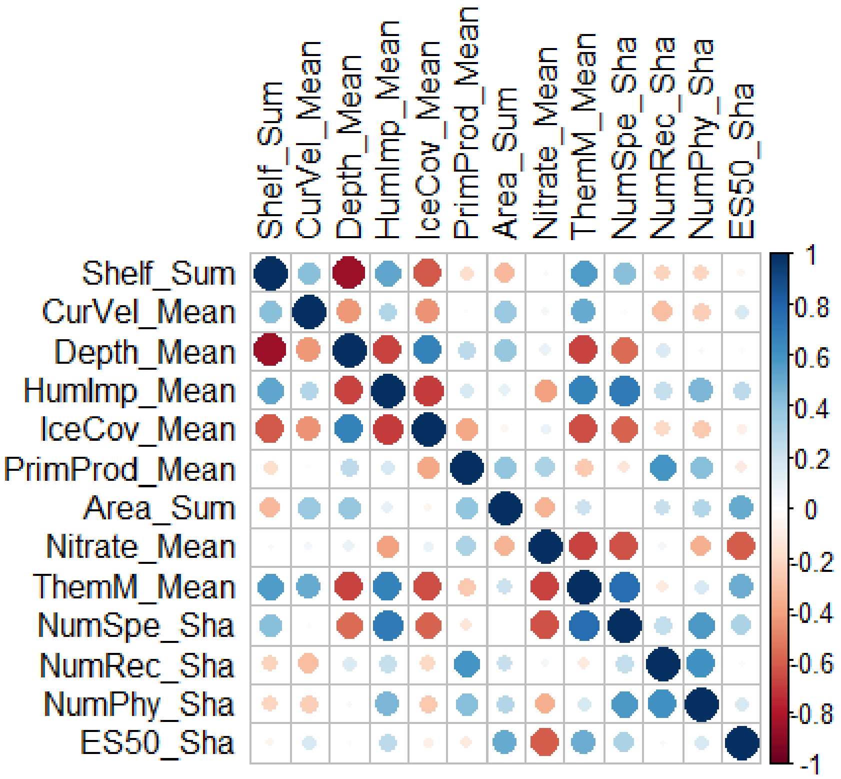


```
#GLMs for number of species, Global data, Shallow
shallow.numsp.intercept <- glm(NumSpe_Sha ~ 1, family = "poisson", data = Ecological_Data_Global)
summary(shallow.numsp.intercept)

##
## Call:
## glm(formula = NumSpe_Sha ~ 1, family = "poisson", data = Ecological_Data_Global)
##
## Coefficients:
```

```
##              Estimate Std. Error z value Pr(>|z|)
## (Intercept) 9.405113   0.001685    5583   <2e-16 ***
## ---
## Signif. codes:  0 '***' 0.001 '**' 0.01 '*' 0.05 '.' 0.1 ' ' 1
##
## (Dispersion parameter for poisson family taken to be 1)
##
##     Null deviance: 112823  on 28  degrees of freedom
## Residual deviance: 112823  on 28  degrees of freedom
## AIC: 113145
##
## Number of Fisher Scoring iterations: 5
```

```
shallow.numsp.numrec <- glm(NumSpe_Sha ~ NumRec_Sha, family = "poisson",
data = Ecological_Data_Global)
summary(shallow.numsp.numrec)
```

```
##
## Call:
## glm(formula = NumSpe_Sha ~ NumRec_Sha, family = "poisson", data = Ecol
ogical_Data_Global)
##
## Coefficients:
##               Estimate Std. Error z value Pr(>|z|)
## (Intercept) 9.310e+00  2.133e-03 4363.94   <2e-16 ***
## NumRec_Sha  6.783e-08  8.628e-10   78.61   <2e-16 ***
## ---
## Signif. codes:  0 '***' 0.001 '**' 0.01 '*' 0.05 '.' 0.1 ' ' 1
##
## (Dispersion parameter for poisson family taken to be 1)
##
##     Null deviance: 112823  on 28  degrees of freedom
## Residual deviance: 107169  on 27  degrees of freedom
## AIC: 107493
##
## Number of Fisher Scoring iterations: 5
```

```
shallow.numsp.shelfsum <- glm(NumSpe_Sha ~ NumRec_Sha + Shelf_Sum, family
= "poisson", data = Ecological_Data_Global)
summary(shallow.numsp.shelfsum)
```

```
##
## Call:
## glm(formula = NumSpe_Sha ~ NumRec_Sha + Shelf_Sum, family = "poisson",
##     data = Ecological_Data_Global)
##
## Coefficients:
##               Estimate Std. Error z value Pr(>|z|)
## (Intercept) 9.566e+00  2.607e-03  3668.9   <2e-16 ***
## NumRec_Sha  1.345e-07  1.012e-09   133.0   <2e-16 ***
## Shelf_Sum   1.771e-07  1.156e-09   153.2   <2e-16 ***
## ---
```

```
## Signif. codes:  0 '***' 0.001 '**' 0.01 '*' 0.05 '.' 0.1 ' ' 1
##
## (Dispersion parameter for poisson family taken to be 1)
##
##     Null deviance: 112823  on 28  degrees of freedom
## Residual deviance:  75432  on 26  degrees of freedom
## AIC: 75758
##
## Number of Fisher Scoring iterations: 4
```

```
shallow.numsp.current <- glm(NumSpe_Sha ~ NumRec_Sha + CurVel_Mean, famil
y = "poisson", data = Ecological_Data_Global)
summary(shallow.numsp.current)
```

```
##
## Call:
## glm(formula = NumSpe_Sha ~ NumRec_Sha + CurVel_Mean, family = "poisson
",
##     data = Ecological_Data_Global)
##
## Coefficients:
##              Estimate Std. Error z value Pr(>|z|)
## (Intercept) 9.246e+00  4.357e-03 2122.19   <2e-16 ***
## NumRec_Sha  7.305e-08  9.158e-10   79.78   <2e-16 ***
## CurVel_Mean 6.501e-01  3.808e-02   17.07   <2e-16 ***
## ---
## Signif. codes:  0 '***' 0.001 '**' 0.01 '*' 0.05 '.' 0.1 ' ' 1
##
## (Dispersion parameter for poisson family taken to be 1)
##
##     Null deviance: 112823  on 28  degrees of freedom
## Residual deviance: 106881  on 26  degrees of freedom
## AIC: 107206
##
## Number of Fisher Scoring iterations: 5
```

```
shallow.numsp.depth <- glm(NumSpe_Sha ~ NumRec_Sha + Depth_Mean, family =
"poisson", data = Ecological_Data_Global)
summary(shallow.numsp.depth)
```

```
##
## Call:
## glm(formula = NumSpe_Sha ~ NumRec_Sha + Depth_Mean, family = "poisson"
,
##     data = Ecological_Data_Global)
##
## Coefficients:
##               Estimate Std. Error z value Pr(>|z|)
## (Intercept)  7.573e+00  9.948e-03   761.2   <2e-16 ***
## NumRec_Sha   1.483e-07  1.074e-09   138.1   <2e-16 ***
## Depth_Mean  -4.658e-04  2.473e-06  -188.4   <2e-16 ***
## ---
```

```
## Signif. codes:  0 '***' 0.001 '**' 0.01 '*' 0.05 '.' 0.1 ' ' 1
##
## (Dispersion parameter for poisson family taken to be 1)
##
##     Null deviance: 112823  on 28  degrees of freedom
## Residual deviance:  56140  on 26  degrees of freedom
## AIC: 56466
##
## Number of Fisher Scoring iterations: 4
```

```
shallow.numsp.humimp <- glm(NumSpe_Sha ~ NumRec_Sha + HumImp_Mean, family
= "poisson", data = Ecological_Data_Global)
summary(shallow.numsp.humimp)
```

```
##
## Call:
## glm(formula = NumSpe_Sha ~ NumRec_Sha + HumImp_Mean, family = "poisson
",
##     data = Ecological_Data_Global)
##
## Coefficients:
##              Estimate Std. Error z value Pr(>|z|)
## (Intercept) 8.420e+00  5.036e-03 1672.13   <2e-16 ***
## NumRec_Sha  3.550e-08  9.586e-10   37.03   <2e-16 ***
## HumImp_Mean 8.819e-02  4.099e-04  215.14   <2e-16 ***
## ---
## Signif. codes:  0 '***' 0.001 '**' 0.01 '*' 0.05 '.' 0.1 ' ' 1
##
## (Dispersion parameter for poisson family taken to be 1)
##
##     Null deviance: 112823  on 28  degrees of freedom
## Residual deviance:  58898  on 26  degrees of freedom
## AIC: 59224
##
## Number of Fisher Scoring iterations: 5
```

```
shallow.numsp.icecov <- glm(NumSpe_Sha ~ NumRec_Sha + IceCov_Mean, family
= "poisson", data = Ecological_Data_Global)
summary(shallow.numsp.icecov)
```

```
##
## Call:
## glm(formula = NumSpe_Sha ~ NumRec_Sha + IceCov_Mean, family = "poisson
",
##     data = Ecological_Data_Global)
##
## Coefficients:
##               Estimate Std. Error z value Pr(>|z|)
## (Intercept)  9.490e+00  2.236e-03 4243.38   <2e-16 ***
## NumRec_Sha   4.583e-08  9.263e-10   49.48   <2e-16 ***
## IceCov_Mean -3.024e+00  1.874e-02 -161.42   <2e-16 ***
## ---
```

```
## Signif. codes:  0 '***' 0.001 '**' 0.01 '*' 0.05 '.' 0.1 ' ' 1
##
## (Dispersion parameter for poisson family taken to be 1)
##
##     Null deviance: 112823  on 28  degrees of freedom
## Residual deviance:  56829  on 26  degrees of freedom
## AIC: 57155
##
## Number of Fisher Scoring iterations: 4
```

```
shallow.numsp.primprod <- glm(NumSpe_Sha ~ NumRec_Sha + PrimProd_Mean, fa
mily = "poisson", data = Ecological_Data_Global)
summary(shallow.numsp.primprod)
```

```
##
## Call:
## glm(formula = NumSpe_Sha ~ NumRec_Sha + PrimProd_Mean, family = "poiss
on",
##     data = Ecological_Data_Global)
##
## Coefficients:
##                 Estimate Std. Error z value Pr(>|z|)
## (Intercept)    1.004e+01  6.718e-03  1495.0   <2e-16 ***
## NumRec_Sha     1.505e-07  1.150e-09   130.9   <2e-16 ***
## PrimProd_Mean -5.549e-01  4.969e-03  -111.7   <2e-16 ***
## ---
## Signif. codes:  0 '***' 0.001 '**' 0.01 '*' 0.05 '.' 0.1 ' ' 1
##
## (Dispersion parameter for poisson family taken to be 1)
##
##     Null deviance: 112823  on 28  degrees of freedom
## Residual deviance:  94830  on 26  degrees of freedom
## AIC: 95156
##
## Number of Fisher Scoring iterations: 5
```

```
shallow.numsp.themmean <- glm(NumSpe_Sha ~ NumRec_Sha + ThemM_Mean, famil
y = "poisson", data = Ecological_Data_Global)
summary(shallow.numsp.themmean)
```

```
##
## Call:
## glm(formula = NumSpe_Sha ~ NumRec_Sha + ThemM_Mean, family = "poisson"
,
##     data = Ecological_Data_Global)
##
## Coefficients:
##              Estimate Std. Error z value Pr(>|z|)
## (Intercept) 8.192e+00  5.494e-03  1491.1   <2e-16 ***
## NumRec_Sha  1.527e-07  1.028e-09   148.6   <2e-16 ***
## ThemM_Mean  5.480e-02  2.169e-04   252.6   <2e-16 ***
## ---
```

```
## Signif. codes:  0 '***' 0.001 '**' 0.01 '*' 0.05 '.' 0.1 ' ' 1
##
## (Dispersion parameter for poisson family taken to be 1)
##
##     Null deviance: 112823  on 28  degrees of freedom
## Residual deviance:  28812  on 26  degrees of freedom
## AIC: 29138
##
## Number of Fisher Scoring iterations: 4
```

```
shallow.numsp.area <- glm(NumSpe_Sha ~ NumRec_Sha + Area_Sum, family = "p
oisson", data = Ecological_Data_Global)
summary(shallow.numsp.area)
```

```
##
## Call:
## glm(formula = NumSpe_Sha ~ NumRec_Sha + Area_Sum, family = "poisson",
##     data = Ecological_Data_Global)
##
## Coefficients:
##               Estimate Std. Error z value Pr(>|z|)
## (Intercept)  9.406e+00  4.859e-03 1935.57   <2e-16 ***
## NumRec_Sha   7.394e-08  9.144e-10   80.87   <2e-16 ***
## Area_Sum    -8.826e-14  4.075e-15  -21.66   <2e-16 ***
## ---
## Signif. codes:  0 '***' 0.001 '**' 0.01 '*' 0.05 '.' 0.1 ' ' 1
##
## (Dispersion parameter for poisson family taken to be 1)
##
##     Null deviance: 112823  on 28  degrees of freedom
## Residual deviance: 106696  on 26  degrees of freedom
## AIC: 107022
##
## Number of Fisher Scoring iterations: 5
```

```
shallow.numsp.nitrate <- glm(NumSpe_Sha ~ NumRec_Sha + Nitrate_Mean, fami
ly = "poisson", data = Ecological_Data_Global)
summary(shallow.numsp.nitrate)
```

```
##
## Call:
## glm(formula = NumSpe_Sha ~ NumRec_Sha + Nitrate_Mean, family = "poisso
n",
##     data = Ecological_Data_Global)
##
## Coefficients:
##                Estimate Std. Error z value Pr(>|z|)
## (Intercept)   9.585e+00  2.328e-03  4117.3   <2e-16 ***
## NumRec_Sha    1.317e-07  1.017e-09   129.5   <2e-16 ***
## Nitrate_Mean -9.789e-02  4.744e-04  -206.3   <2e-16 ***
## ---
## Signif. codes:  0 '***' 0.001 '**' 0.01 '*' 0.05 '.' 0.1 ' ' 1
```

```
##
## (Dispersion parameter for poisson family taken to be 1)
##
##     Null deviance: 112823  on 28  degrees of freedom
## Residual deviance:  43741  on 26  degrees of freedom
## AIC: 44067
##
## Number of Fisher Scoring iterations: 5
```

```
#Model selection for number of species, Global
shallow.numsp.models <- list(Intercept = shallow.numsp.intercept,
                             NumRec = shallow.numsp.numrec,
                             ConShe = shallow.numsp.shelfsum,
                             CurVel = shallow.numsp.current,
                             Depth = shallow.numsp.depth,
                             HumImp = shallow.numsp.humimp,
                             IceCov = shallow.numsp.icecov,
                             PriPro = shallow.numsp.primprod,
                             TemMea = shallow.numsp.themmean,
                             Area = shallow.numsp.area,
                             Nitrate = shallow.numsp.nitrate)
shallow.numsp.aic.df <- data.frame(Model = names(shallow.numsp.models),
                                   AIC = sapply(shallow.numsp.models, fun
ction(x) AICc(x)),
                                   akaike.weights(sapply(shallow.numsp.mo
dels, function(x) AICc(x))))


shallow.numsp.aic.df <- shallow.numsp.aic.df[order(shallow.numsp.aic.df$A
IC),]
shallow.numsp.aic.df$Cumulative.Weight <- cumsum(shallow.numsp.aic.df$wei
ghts)

kable(shallow.numsp.aic.df, row.names = FALSE)
```

Model	AIC	deltaAIC	rel.LL	weights	Cumulative.Weight
TemMea	29138.90	0.00	1	1	1
Nitrate	44067.77	14928.88	0	0	1
Depth	56466.94	27328.05	0	0	1
IceCov	57155.87	28016.97	0	0	1
HumImp	59225.11	30086.22	0	0	1
ConShe	75759.25	46620.36	0	0	1
PriPro	95156.83	66017.94	0	0	1
Area	107022.79	77883.89	0	0	1
CurVel	107207.30	78068.40	0	0	1
NumRec	107493.69	78354.80	0	0	1
Intercept	113144.71	84005.81	0	0	1

```
#write.csv(shallow.numsp.aic.df, file = "global.5.degree.numsp.shallow.aic.csv")

#Plots for number of species, Shallow
ggplot(Ecological_Data_Global, aes(x = NumRec_Sha, y = predict(shallow.numsp.numrec, Ecological_Data_Global))) +
  geom_smooth(method = "glm", formula = y ~ x, color = "#1a80bb", fill = "#85bede") +  # Add a smooth dark blue line with light blue shadow
  geom_point(size = 3) +  # Add scatter plot points
  theme_bw() +  # Use the black and white theme
  scale_x_continuous(labels = scales::scientific) +
  labs(
    x = "Number of Records",  # Shorten the x-axis title
    y = "Predicted Value"  # Shorten the y-axis title
  ) +
  theme(
    panel.grid.minor = element_blank(),
    panel.grid.major = element_blank(),
    axis.text.x = element_text(size =20),  # Increase x-axis text size
    axis.text.y = element_text(size = 20),  # Increase y-axis text size
    axis.title.x = element_text(size = 22), # Increase x-axis title size
    axis.title.y = element_text(size = 22)  # Increase y-axis title size
  )
```

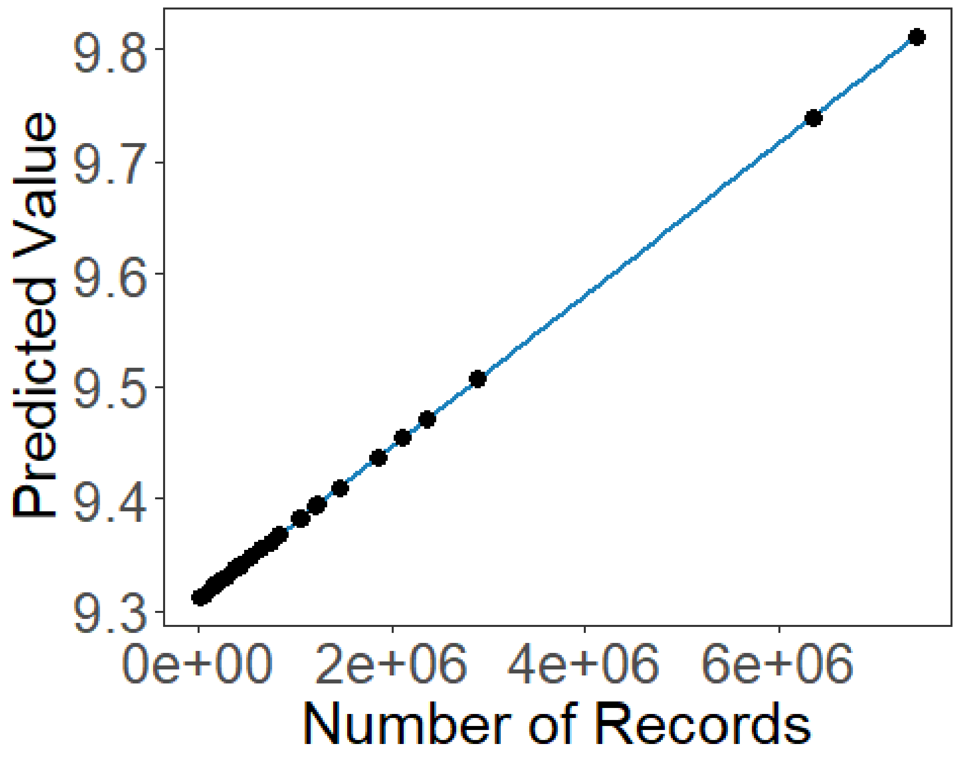


```
ggplot(Ecological_Data_Global, aes(x = Shelf_Sum, y = predict(shallow.numsp.shelfsum, Ecological_Data_Global))) +
  geom_smooth(method = "glm", formula = y ~ x, color = "#1a80bb", fill = "#85bede") +  # Add a smooth dark blue line with light blue shadow
  geom_point(size = 3) +  # Add scatter plot points
  theme_bw() +  # Use the black and white theme
```

```
  scale_x_continuous(labels = scales::scientific) +
  labs(
    x = "Continental Shelf (km2)",  # Shorten the x-axis title
    y = "Predicted Value"  # Shorten the y-axis title
  ) +
  theme(
    panel.grid.minor = element_blank(),
    panel.grid.major = element_blank(),
    axis.text.x = element_text(size =20),  # Increase x-axis text size
    axis.text.y = element_text(size = 20),  # Increase y-axis text size
    axis.title.x = element_text(size = 22), # Increase x-axis title size
    axis.title.y = element_text(size = 22)  # Increase y-axis title size
  )
```

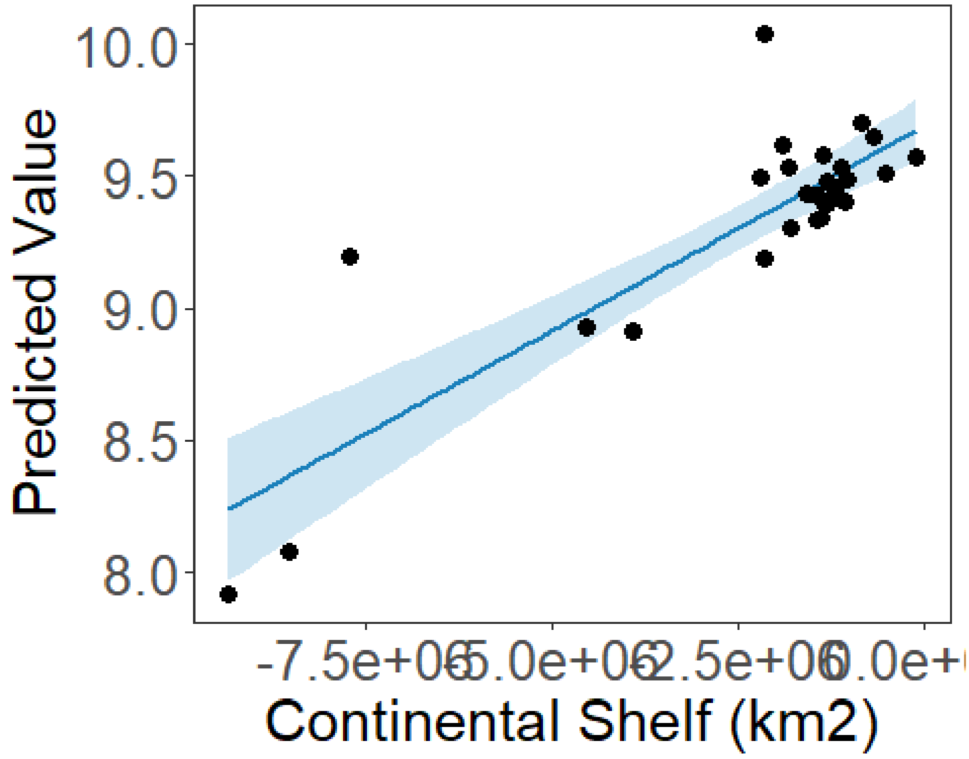


```
ggplot(Ecological_Data_Global, aes(x = CurVel_Mean, y = predict(shallow.n
umsp.current, Ecological_Data_Global))) +
  geom_smooth(method = "glm", formula = y ~ x, color = "#1a80bb", fill =
"#85bede") +  # Add a smooth dark blue line with light blue shadow
  geom_point(size = 3) +  # Add scatter plot points
  theme_bw() +  # Use the black and white theme
  labs(
    x = "Current Velocity (m.s-1)",  # Shorten the x-axis title
    y = "Predicted Value"  # Shorten the y-axis title
  ) +
  theme(
    panel.grid.minor = element_blank(),
    panel.grid.major = element_blank(),
    axis.text.x = element_text(size =20),  # Increase x-axis text size
    axis.text.y = element_text(size = 20),  # Increase y-axis text size
    axis.title.x = element_text(size = 22), # Increase x-axis title size
```

```
    axis.title.y = element_text(size = 22)  # Increase y-axis title size
  )
```

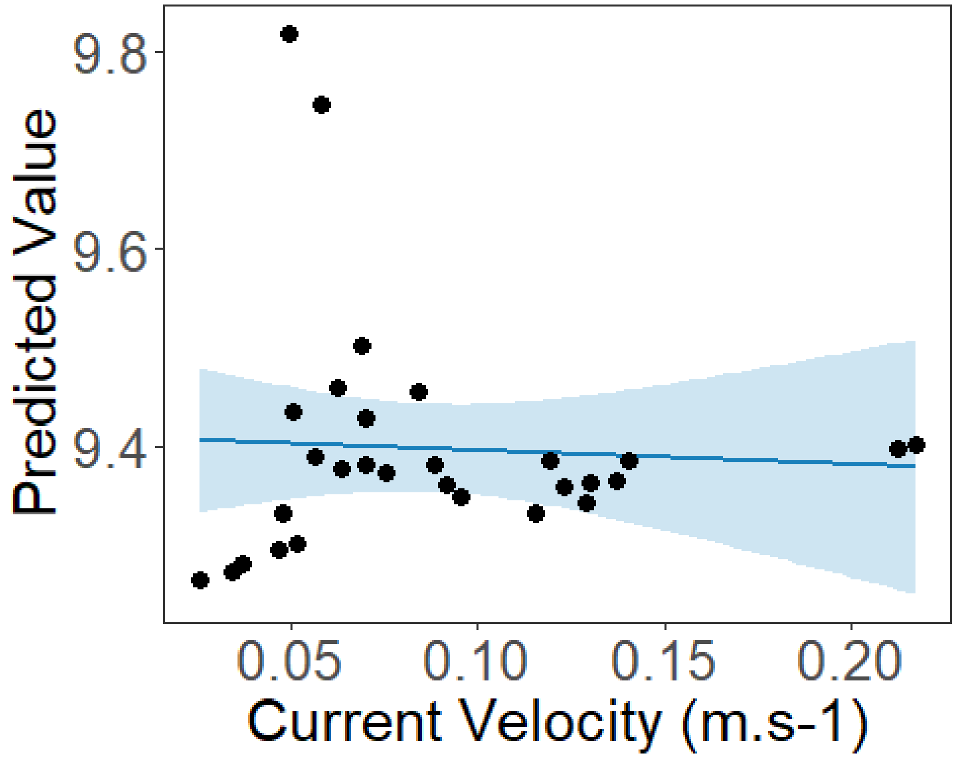


```
ggplot(Ecological_Data_Global, aes(x = Depth_Mean, y = predict(shallow.nu
msp.depth, Ecological_Data_Global))) +
  geom_smooth(method = "glm", formula = y ~ x, color = "#1a80bb", fill =
"#85bede") +  # Add a smooth dark blue line with light blue shadow
  geom_point(size = 3) +  # Add scatter plot points
  theme_bw() +  # Use the black and white theme
  scale_x_continuous(labels = scales::scientific) +
  labs(
    x = "Depth (m)",  # Shorten the x-axis title
    y = "Predicted Value"  # Shorten the y-axis title
  ) +
  theme(
    panel.grid.minor = element_blank(),
    panel.grid.major = element_blank(),
    axis.text.x = element_text(size =20),  # Increase x-axis text size
    axis.text.y = element_text(size = 20),  # Increase y-axis text size
    axis.title.x = element_text(size = 22), # Increase x-axis title size
    axis.title.y = element_text(size = 22)  # Increase y-axis title size
  )
```

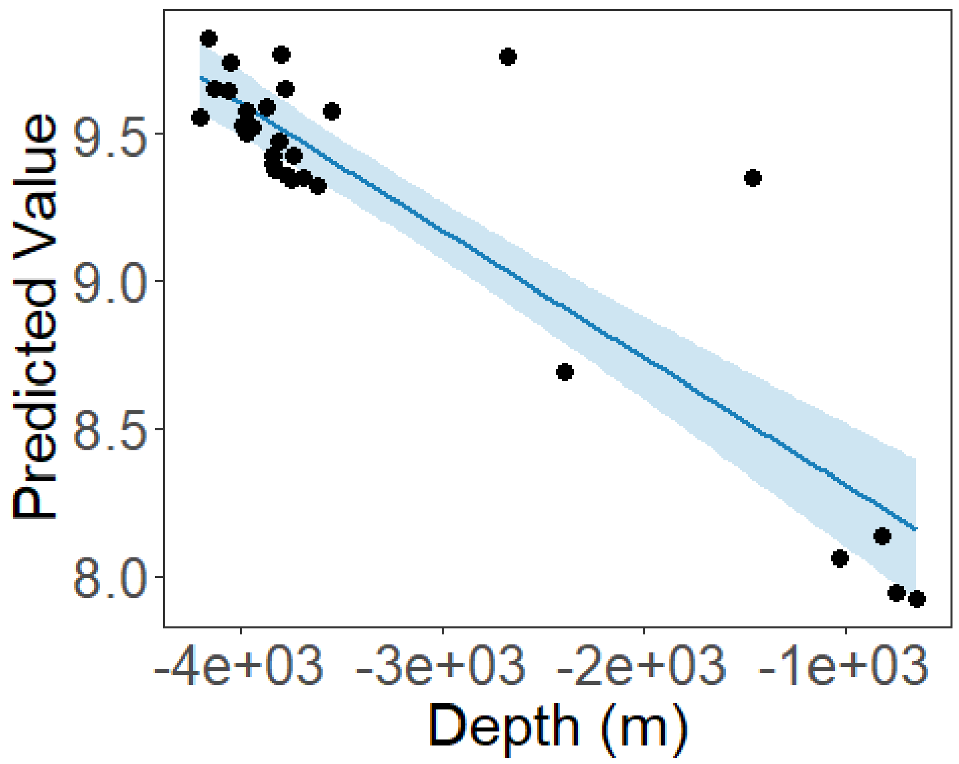


```r
ggplot(Ecological_Data_Global, aes(x = HumImp_Mean, y = predict(shallow.n
umsp.humimp, Ecological_Data_Global))) +
  geom_smooth(method = "glm", formula = y ~ x, color = "#1a80bb", fill =
"#85bede") +  # Add a smooth dark blue line with light blue shadow
  geom_point(size = 3) +  # Add scatter plot points
  theme_bw() +  # Use the black and white theme
  labs(
    x = "Human Impact",  # Shorten the x-axis title
    y = "Predicted Value"  # Shorten the y-axis title
  ) +
  theme(
    panel.grid.minor = element_blank(),
    panel.grid.major = element_blank(),
    axis.text.x = element_text(size =20),  # Increase x-axis text size
    axis.text.y = element_text(size = 20),  # Increase y-axis text size
    axis.title.x = element_text(size = 22), # Increase x-axis title size
    axis.title.y = element_text(size = 22)  # Increase y-axis title size
  )
```

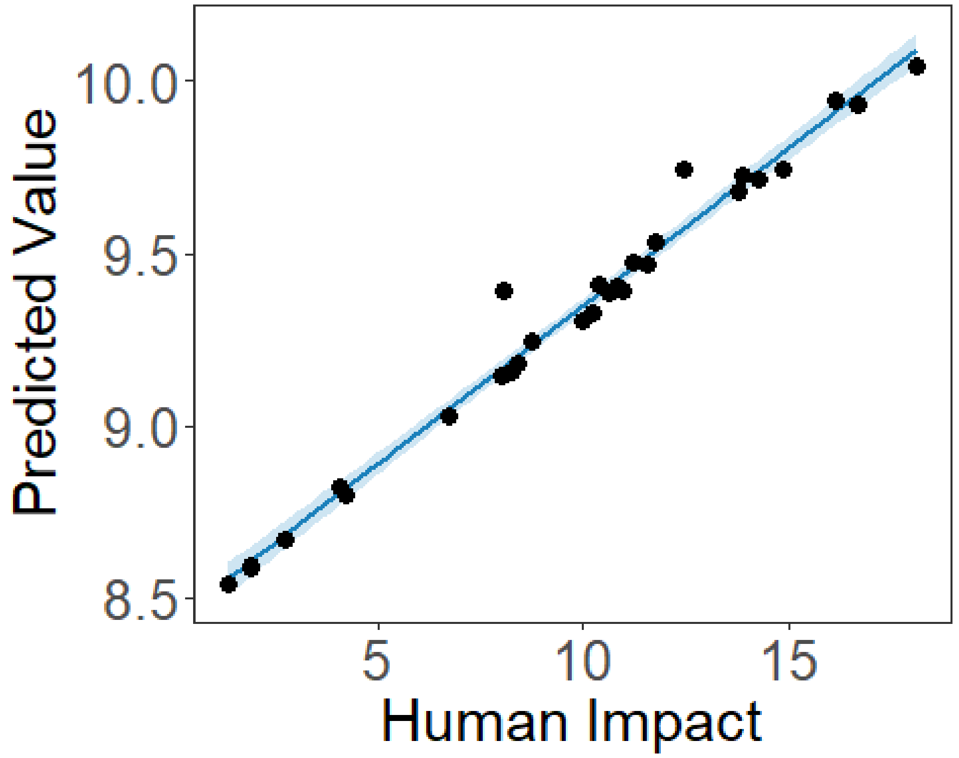


```r
ggplot(Ecological_Data_Global, aes(x = IceCov_Mean, y = predict(shallow.n
umsp.icecov, Ecological_Data_Global))) +
  geom_smooth(method = "glm", formula = y ~ x, color = "#1a80bb", fill =
"#85bede") +  # Add a smooth dark blue line with light blue shadow
  geom_point(size = 3) +  # Add scatter plot points
  theme_bw() +  # Use the black and white theme
  labs(
    x = "Ice Cover (fraction)",  # Shorten the x-axis title
    y = "Predicted Value"  # Shorten the y-axis title
  ) +
  theme(
    panel.grid.minor = element_blank(),
    panel.grid.major = element_blank(),
    axis.text.x = element_text(size =20),  # Increase x-axis text size
    axis.text.y = element_text(size = 20),  # Increase y-axis text size
    axis.title.x = element_text(size = 22), # Increase x-axis title size
    axis.title.y = element_text(size = 22)  # Increase y-axis title size
  )
```

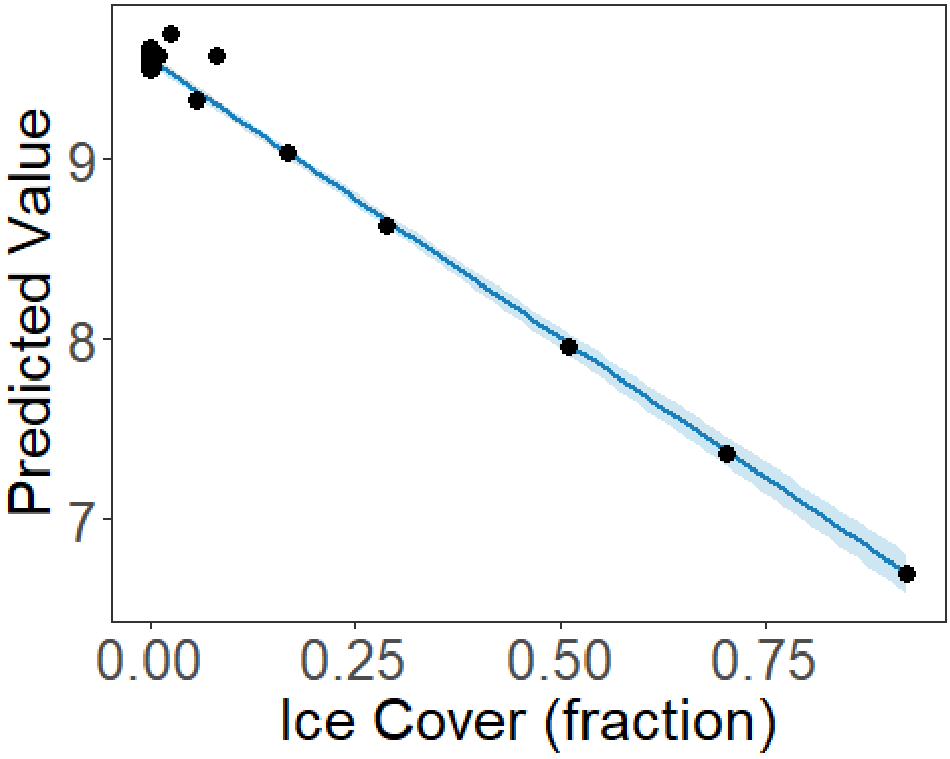


```
ggplot(Ecological_Data_Global, aes(x = PrimProd_Mean, y = predict(shallow
.numsp.primprod, Ecological_Data_Global))) +
  geom_smooth(method = "glm", formula = y ~ x, color = "#1a80bb", fill =
"#85bede") +  # Add a smooth dark blue line with light blue shadow
  geom_point(size = 3) +  # Add scatter plot points
  theme_bw() +  # Use the black and white theme
  labs(
    x = "Primary Productivity (mmol . m-3)",  # Shorten the x-axis title
    y = "Predicted Value"  # Shorten the y-axis title
  ) +
  theme(
    panel.grid.minor = element_blank(),
    panel.grid.major = element_blank(),
    axis.text.x = element_text(size =20),  # Increase x-axis text size
    axis.text.y = element_text(size = 20),  # Increase y-axis text size
    axis.title.x = element_text(size = 22), # Increase x-axis title size
    axis.title.y = element_text(size = 22)  # Increase y-axis title size
  )
```

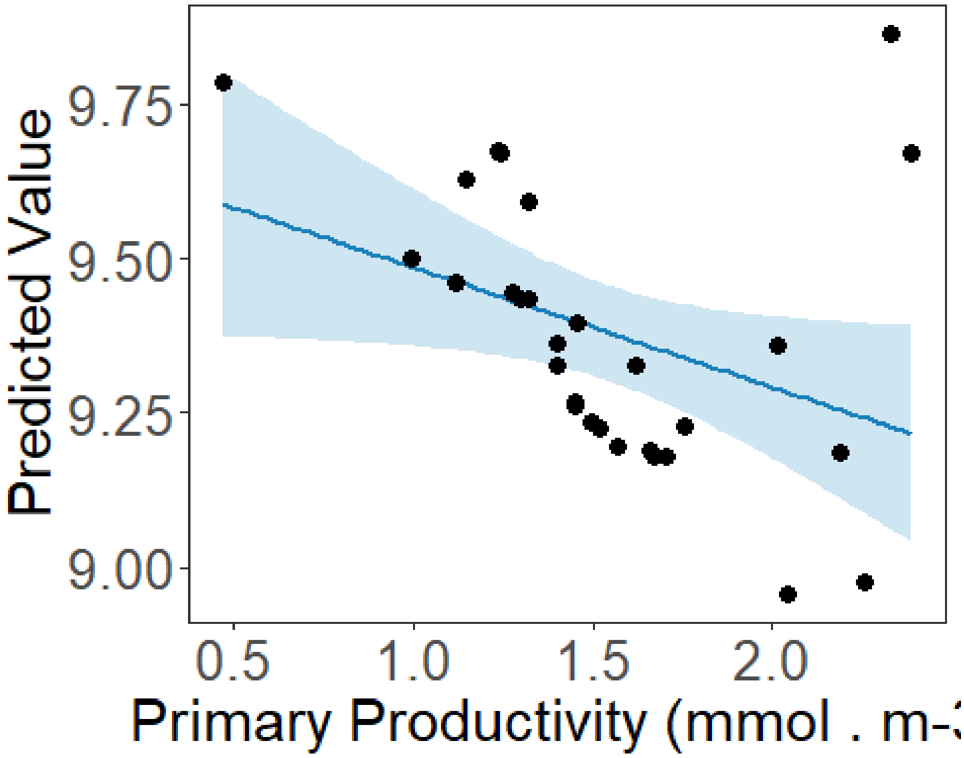


```
ggplot(Ecological_Data_Global, aes(x = ThemM_Mean, y = predict(shallow.nu
msp.themmean, Ecological_Data_Global))) +
  geom_smooth(method = "glm", formula = y ~ x, color = "#1a80bb", fill =
"#85bede") +  # Add a smooth dark blue line with light blue shadow
  geom_point(size = 3) +  # Add scatter plot points
  theme_bw() +  # Use the black and white theme
  labs(
    x = "Temperature Mean (ºC)",  # Shorten the x-axis title
    y = "Predicted Value"  # Shorten the y-axis title
  ) +
  theme(
    panel.grid.minor = element_blank(),
    panel.grid.major = element_blank(),
    axis.text.x = element_text(size =20),  # Increase x-axis text size
    axis.text.y = element_text(size = 20),  # Increase y-axis text size
    axis.title.x = element_text(size = 22), # Increase x-axis title size
    axis.title.y = element_text(size = 22)  # Increase y-axis title size
  )
```

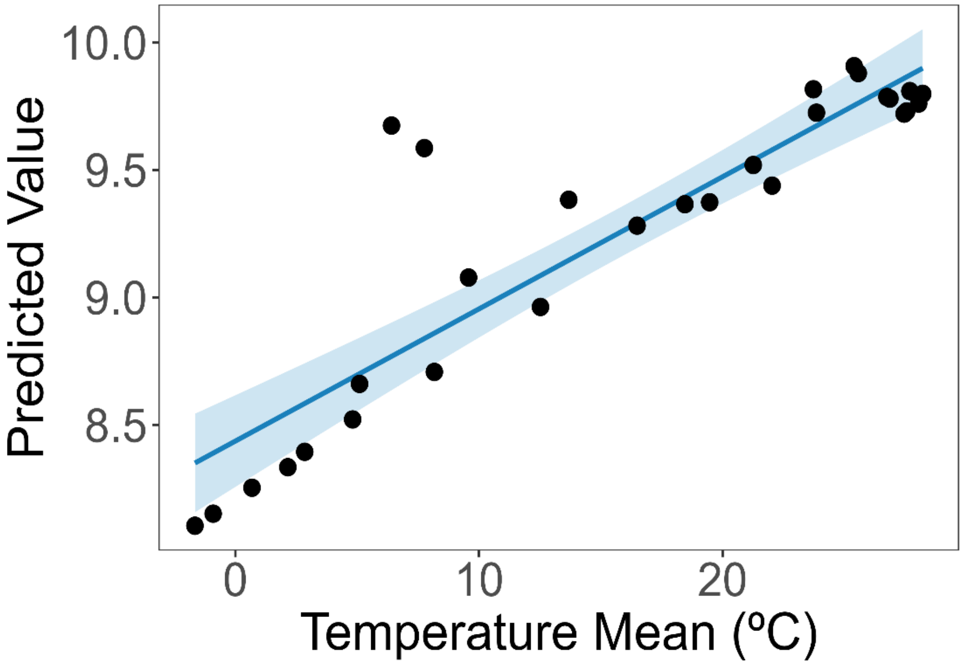


```
ggplot(Ecological_Data_Global, aes(x = Area_Sum, y = predict(shallow.nums
p.area, Ecological_Data_Global))) +
  geom_smooth(method = "glm", formula = y ~ x, color = "#1a80bb", fill =
"#85bede") +  # Add a smooth dark blue line with light blue shadow
  geom_point(size = 3) +  # Add scatter plot points
  theme_bw() +  # Use the black and white theme
  labs(
    x = "Area (km2)",  # Shorten the x-axis title
    y = "Predicted Value"  # Shorten the y-axis title
  ) +
  theme(
    panel.grid.minor = element_blank(),
    panel.grid.major = element_blank(),
    axis.text.x = element_text(size =20),  # Increase x-axis text size
    axis.text.y = element_text(size = 20),  # Increase y-axis text size
    axis.title.x = element_text(size = 22), # Increase x-axis title size
    axis.title.y = element_text(size = 22)  # Increase y-axis title size
  )
```

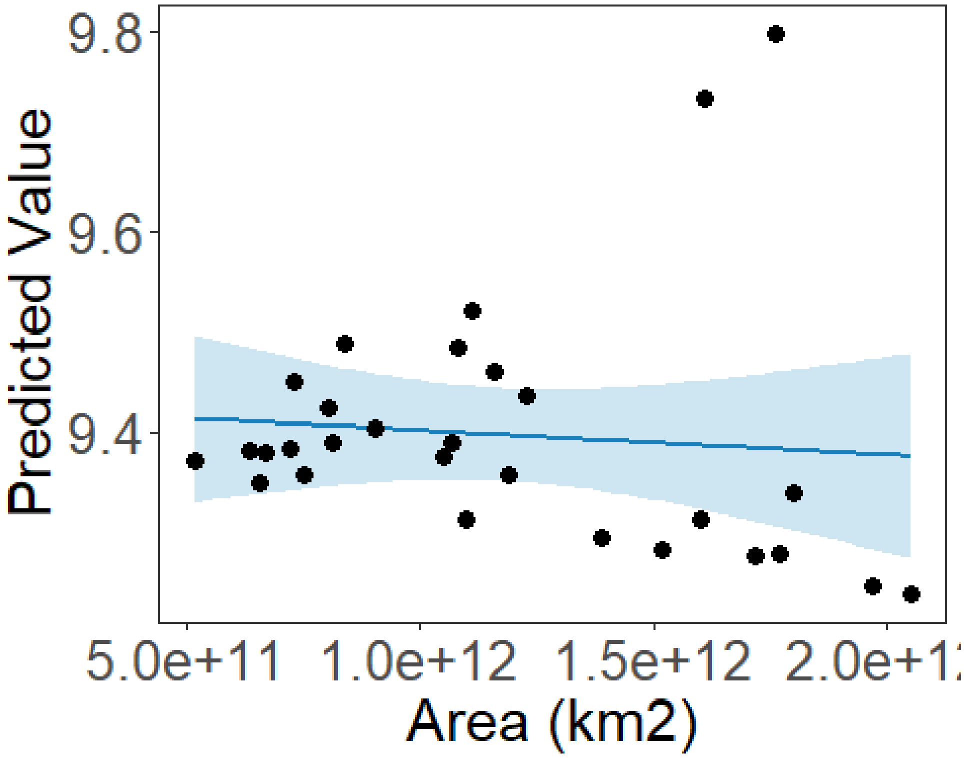


```
ggplot(Ecological_Data_Global, aes(x = Nitrate_Mean, y = predict(shallow.
numsp.nitrate, Ecological_Data_Global))) +
  geom_smooth(method = "glm", formula = y ~ x, color = "#1a80bb", fill =
"#85bede") +  # Add a smooth dark blue line with light blue shadow
  geom_point(size = 3) +  # Add scatter plot points
  theme_bw() +  # Use the black and white theme
  labs(
    x = "Nitrate (mmol . m-3)",  # Shorten the x-axis title
    y = "Predicted Value"  # Shorten the y-axis title
  ) +
  theme(
    panel.grid.minor = element_blank(),
    panel.grid.major = element_blank(),
    axis.text.x = element_text(size =20),  # Increase x-axis text size
    axis.text.y = element_text(size = 20),  # Increase y-axis text size
    axis.title.x = element_text(size = 22), # Increase x-axis title size
    axis.title.y = element_text(size = 22)  # Increase y-axis title size
  )
```

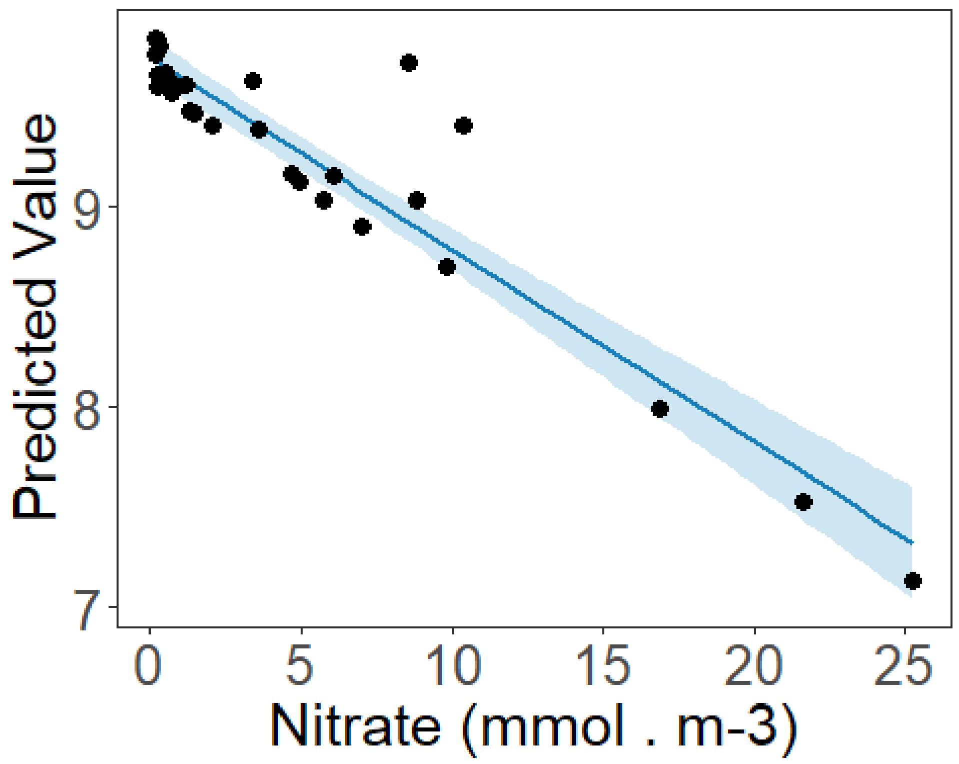


```
# ===================================
#                SHALLOW-ES50-GLM
# ===================================
shallow.es50.intercept <- glm(ES50_Sha ~ 1, family = "poisson", data = Ec
ological_Data_Global)
summary(shallow.es50.intercept)

##
## Call:
## glm(formula = ES50_Sha ~ 1, family = "poisson", data = Ecological_Data
_Global)
##
## Coefficients:
##             Estimate Std. Error z value Pr(>|z|)
## (Intercept)  3.69917    0.02921   126.6   <2e-16 ***
## ---
## Signif. codes:  0 '***' 0.001 '**' 0.01 '*' 0.05 '.' 0.1 ' ' 1
##
## (Dispersion parameter for poisson family taken to be 1)
##
##     Null deviance: 20.114  on 28  degrees of freedom
## Residual deviance: 20.114  on 28  degrees of freedom
## AIC: 182.55
##
## Number of Fisher Scoring iterations: 4

shallow.es50.themmean <- glm(ES50_Sha ~ ThemM_Mean, family = "poisson", d
ata = Ecological_Data_Global)
summary(shallow.es50.themmean)
```

```
##
## Call:
## glm(formula = ES50_Sha ~ ThemM_Mean, family = "poisson", data = Ecolog
ical_Data_Global)
##
## Coefficients:
##             Estimate Std. Error z value Pr(>|z|)
## (Intercept) 3.598392   0.055879  64.396   <2e-16 ***
## ThemM_Mean  0.006227   0.002886   2.158    0.031 *
## ---
## Signif. codes:  0 '***' 0.001 '**' 0.01 '*' 0.05 '.' 0.1 ' ' 1
##
## (Dispersion parameter for poisson family taken to be 1)
##
##     Null deviance: 20.114  on 28  degrees of freedom
## Residual deviance: 15.423  on 27  degrees of freedom
## AIC: 179.86
##
## Number of Fisher Scoring iterations: 4
```

```
shallow.es50.numrec <- glm(ES50_Sha ~ NumRec_Sha, family = "poisson", dat
a = Ecological_Data_Global)
summary(shallow.es50.numrec)
```

```
##
## Call:
## glm(formula = ES50_Sha ~ NumRec_Sha, family = "poisson", data = Ecolog
ical_Data_Global)
##
## Coefficients:
##               Estimate Std. Error z value Pr(>|z|)
## (Intercept)  3.702e+00  3.667e-02 100.957   <2e-16 ***
## NumRec_Sha  -2.302e-09  1.726e-08  -0.133    0.894
## ---
## Signif. codes:  0 '***' 0.001 '**' 0.01 '*' 0.05 '.' 0.1 ' ' 1
##
## (Dispersion parameter for poisson family taken to be 1)
##
##     Null deviance: 20.114  on 28  degrees of freedom
## Residual deviance: 20.096  on 27  degrees of freedom
## AIC: 184.53
##
## Number of Fisher Scoring iterations: 4
```

```
shallow.es50.shelfsum <- glm(ES50_Sha ~ NumRec_Sha + Shelf_Sum, family =
"poisson", data = Ecological_Data_Global)
summary(shallow.es50.shelfsum)
```

```
##
## Call:
## glm(formula = ES50_Sha ~ NumRec_Sha + Shelf_Sum, family = "poisson",
##     data = Ecological_Data_Global)
```

```
##
## Coefficients:
##                Estimate Std. Error z value Pr(>|z|)
## (Intercept)  3.695e+00  4.409e-02  83.807   <2e-16 ***
## NumRec_Sha  -3.361e-09  1.765e-08  -0.190    0.849
## Shelf_Sum   -3.527e-09  1.286e-08  -0.274    0.784
## ---
## Signif. codes:  0 '***' 0.001 '**' 0.01 '*' 0.05 '.' 0.1 ' ' 1
##
## (Dispersion parameter for poisson family taken to be 1)
##
##     Null deviance: 20.114  on 28  degrees of freedom
## Residual deviance: 20.021  on 26  degrees of freedom
## AIC: 186.45
##
## Number of Fisher Scoring iterations: 4
```

```
shallow.es50.current <- glm(ES50_Sha ~ NumRec_Sha + CurVel_Mean, family =
"poisson", data = Ecological_Data_Global)
summary(shallow.es50.current)
```

```
##
## Call:
## glm(formula = ES50_Sha ~ NumRec_Sha + CurVel_Mean, family = "poisson",
##     data = Ecological_Data_Global)
##
## Coefficients:
##               Estimate Std. Error z value Pr(>|z|)
## (Intercept) 3.653e+00  7.379e-02  49.508   <2e-16 ***
## NumRec_Sha  1.946e-09  1.811e-08   0.107    0.914
## CurVel_Mean 4.924e-01  6.390e-01   0.771    0.441
## ---
## Signif. codes:  0 '***' 0.001 '**' 0.01 '*' 0.05 '.' 0.1 ' ' 1
##
## (Dispersion parameter for poisson family taken to be 1)
##
##     Null deviance: 20.114  on 28  degrees of freedom
## Residual deviance: 19.505  on 26  degrees of freedom
## AIC: 185.94
##
## Number of Fisher Scoring iterations: 4
```

```
shallow.es50.depth <- glm(ES50_Sha ~ Depth_Mean, family = "poisson", data
= Ecological_Data_Global)
summary(shallow.es50.depth)
```

```
##
## Call:
## glm(formula = ES50_Sha ~ Depth_Mean, family = "poisson", data = Ecolog
ical_Data_Global)
##
## Coefficients:
```

```
##                  Estimate Std. Error z value Pr(>|z|)
## (Intercept)  3.689e+00  8.958e-02  41.174   <2e-16 ***
## Depth_Mean  -3.234e-06  2.578e-05  -0.125      0.9
## ---
## Signif. codes:  0 '***' 0.001 '**' 0.01 '*' 0.05 '.' 0.1 ' ' 1
##
## (Dispersion parameter for poisson family taken to be 1)
##
##     Null deviance: 20.114  on 28  degrees of freedom
## Residual deviance: 20.098  on 27  degrees of freedom
## AIC: 184.53
##
## Number of Fisher Scoring iterations: 4
```

```
shallow.es50.humimp <- glm(ES50_Sha ~ HumImp_Mean, family = "poisson", da
ta = Ecological_Data_Global)
summary(shallow.es50.humimp)
```

```
##
## Call:
## glm(formula = ES50_Sha ~ HumImp_Mean, family = "poisson", data = Ecolo
gical_Data_Global)
##
## Coefficients:
##                Estimate Std. Error z value Pr(>|z|)
## (Intercept) 3.623728   0.070765   51.21   <2e-16 ***
## HumImp_Mean 0.007696   0.006524    1.18    0.238
## ---
## Signif. codes:  0 '***' 0.001 '**' 0.01 '*' 0.05 '.' 0.1 ' ' 1
##
## (Dispersion parameter for poisson family taken to be 1)
##
##     Null deviance: 20.114  on 28  degrees of freedom
## Residual deviance: 18.717  on 27  degrees of freedom
## AIC: 183.15
##
## Number of Fisher Scoring iterations: 4
```

```
shallow.es50.icecov <- glm(ES50_Sha ~ IceCov_Mean, family = "poisson", da
ta = Ecological_Data_Global)
summary(shallow.es50.icecov)
```

```
##
## Call:
## glm(formula = ES50_Sha ~ IceCov_Mean, family = "poisson", data = Ecolo
gical_Data_Global)
##
## Coefficients:
##                Estimate Std. Error z value Pr(>|z|)
## (Intercept)  3.70399    0.03168 116.913   <2e-16 ***
## IceCov_Mean -0.05113    0.13191  -0.388    0.698
## ---
```

```
## Signif. codes:  0 '***' 0.001 '**' 0.01 '*' 0.05 '.' 0.1 ' ' 1
##
## (Dispersion parameter for poisson family taken to be 1)
##
##     Null deviance: 20.114  on 28  degrees of freedom
## Residual deviance: 19.962  on 27  degrees of freedom
## AIC: 184.39
##
## Number of Fisher Scoring iterations: 4
```

```
shallow.es50.primprod <- glm(ES50_Sha ~ PrimProd_Mean, family = "poisson"
, data = Ecological_Data_Global)
summary(shallow.es50.primprod)
```

```
##
## Call:
## glm(formula = ES50_Sha ~ PrimProd_Mean, family = "poisson", data = Eco
logical_Data_Global)
##
## Coefficients:
##               Estimate Std. Error z value Pr(>|z|)
## (Intercept)    3.75303    0.11116  33.763   <2e-16 ***
## PrimProd_Mean -0.03492    0.06968  -0.501    0.616
## ---
## Signif. codes:  0 '***' 0.001 '**' 0.01 '*' 0.05 '.' 0.1 ' ' 1
##
## (Dispersion parameter for poisson family taken to be 1)
##
##     Null deviance: 20.114  on 28  degrees of freedom
## Residual deviance: 19.862  on 27  degrees of freedom
## AIC: 184.3
##
## Number of Fisher Scoring iterations: 4
```

```
shallow.es50.area <- glm(ES50_Sha ~ NumRec_Sha + Area_Sum, family = "pois
son", data = Ecological_Data_Global)
summary(shallow.es50.area)
```

```
##
## Call:
## glm(formula = ES50_Sha ~ NumRec_Sha + Area_Sum, family = "poisson",
##     data = Ecological_Data_Global)
##
## Coefficients:
##               Estimate Std. Error z value Pr(>|z|)
## (Intercept)  3.530e+00  8.437e-02  41.838   <2e-16 ***
## NumRec_Sha  -1.070e-08  1.740e-08  -0.615   0.5386
## Area_Sum     1.533e-13  6.657e-14   2.303   0.0213 *
## ---
## Signif. codes:  0 '***' 0.001 '**' 0.01 '*' 0.05 '.' 0.1 ' ' 1
##
## (Dispersion parameter for poisson family taken to be 1)
```

```
##
##     Null deviance: 20.114  on 28  degrees of freedom
## Residual deviance: 14.853  on 26  degrees of freedom
## AIC: 181.29
##
## Number of Fisher Scoring iterations: 4
```

```
shallow.es50.nitrate <- glm(ES50_Sha ~ Nitrate_Mean, family = "poisson",
data = Ecological_Data_Global)
summary(shallow.es50.nitrate)
```

```
##
## Call:
## glm(formula = ES50_Sha ~ Nitrate_Mean, family = "poisson", data = Ecol
ogical_Data_Global)
##
## Coefficients:
##               Estimate Std. Error z value Pr(>|z|)
## (Intercept)   3.761509   0.036791 102.240  < 2e-16 ***
## Nitrate_Mean -0.012934   0.004887  -2.647  0.00813 **
## ---
## Signif. codes:  0 '***' 0.001 '**' 0.01 '*' 0.05 '.' 0.1 ' ' 1
##
## (Dispersion parameter for poisson family taken to be 1)
##
##     Null deviance: 20.114  on 28  degrees of freedom
## Residual deviance: 12.753  on 27  degrees of freedom
## AIC: 177.19
##
## Number of Fisher Scoring iterations: 4
```

```
#Model selection for number of species, Global
shallow.es50.models <- list(Intercept = shallow.es50.intercept,
                            NumRec = shallow.es50.numrec,
                            ConShe = shallow.es50.shelfsum,
                            CurVel = shallow.es50.current,
                            Depth = shallow.es50.depth,
                            HumImp = shallow.es50.humimp,
                            IceCov = shallow.es50.icecov,
                            PriPro = shallow.es50.primprod,
                            TemMea = shallow.es50.themmean,
                            Area = shallow.es50.area,
                            Nitrate = shallow.es50.nitrate)
shallow.es50.aic.df <- data.frame(Model = names(shallow.es50.models),
                                  AIC = sapply(shallow.es50.models, funct
ion(x) AICc(x)),
                                  akaike.weights(sapply(shallow.es50.mode
ls, function(x) AICc(x))))


shallow.es50.aic.df <- shallow.es50.aic.df[order(shallow.es50.aic.df$AIC)
,]
```

```r
shallow.es50.aic.df$Cumulative.Weight <- cumsum(shallow.es50.aic.df$weigh
ts)

kable(shallow.es50.aic.df, row.names = FALSE)
```

Model	AIC	deltaAIC	rel.LL	weights	Cumulative.Weight
Nitrate	177.6473	0.000000	1.0000000	0.6179418	0.6179418
TemMea	180.3178	2.670493	0.2630933	0.1625763	0.7805182
Area	182.2458	4.598521	0.1003330	0.0620000	0.8425181
Intercept	182.6947	5.047390	0.0801629	0.0495360	0.8920541
HumImp	183.6120	5.964638	0.0506752	0.0313143	0.9233685
PriPro	184.7568	7.109483	0.0285888	0.0176662	0.9410346
IceCov	184.8564	7.209055	0.0272003	0.0168082	0.9578428
NumRec	184.9903	7.342943	0.0254390	0.0157198	0.9735627
Depth	184.9923	7.345009	0.0254127	0.0157036	0.9892663
CurVel	186.8985	9.251140	0.0097981	0.0060546	0.9953209
ConShe	187.4139	9.766582	0.0075721	0.0046791	1.0000000

```r
#write.csv(shallow.es50.aic.df, file = "global.5.degree.es50.shallow.aic.
csv")

#Plots for ES50, shallow
ggplot(Ecological_Data_Global, aes(x = NumRec_Sha, y = ES50_Sha)) +
  geom_smooth(method = "glm", formula = y ~ x, color = "#1a80bb", fill =
"#85bede") +  # Add a smooth dark blue line with light blue shadow
  geom_point(size = 3) +  # Add scatter plot points
  theme_bw() +  # Use the black and white theme
  scale_x_continuous(labels = scales::scientific) +
  scale_y_continuous(limits=c(18, 52), breaks=seq(20, 50, by=5), expand =
c(0, 0)) +
  labs(
    x = "Number of Records",  # Shorten the x-axis title
    y = "ES50"  # Shorten the y-axis title
  ) +
  theme(
    panel.grid.minor = element_blank(),
    panel.grid.major = element_blank(),
    axis.text.x = element_text(size =20),  # Increase x-axis text size
    axis.text.y = element_text(size = 20),  # Increase y-axis text size
    axis.title.x = element_text(size = 22), # Increase x-axis title size
    axis.title.y = element_text(size = 22)  # Increase y-axis title size
  )
```

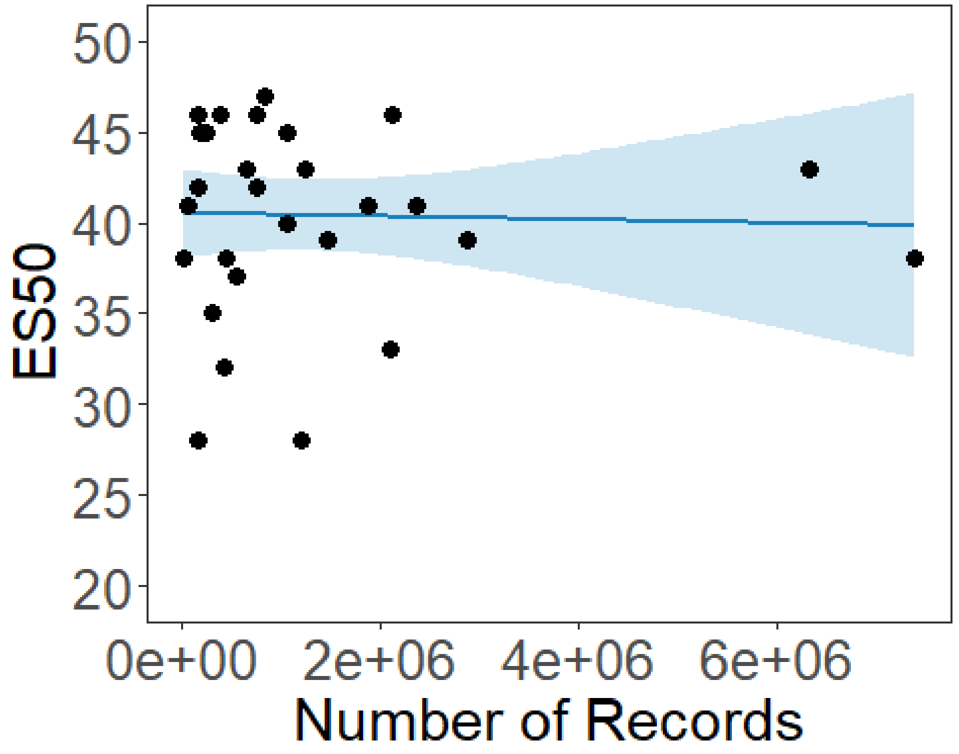


```
ggplot(Ecological_Data_Global, aes(x = Shelf_Sum, y = ES50_Sha)) +
  geom_smooth(method = "glm", formula = y ~ x, color = "#1a80bb", fill =
"#85bede") +  # Add a smooth dark blue line with light blue shadow
  geom_point(size = 3) +  # Add scatter plot points
  theme_bw() +  # Use the black and white theme
  scale_x_continuous(labels = scales::scientific) +
  scale_y_continuous(limits=c(18, 52), breaks=seq(20, 50, by=5), expand =
c(0, 0)) +
  labs(
    x = "Continental Shelf (km2)",  # Shorten the x-axis title
    y = "ES50"  # Shorten the y-axis title
  ) +
  theme(
    panel.grid.minor = element_blank(),
    panel.grid.major = element_blank(),
    axis.text.x = element_text(size =20),  # Increase x-axis text size
    axis.text.y = element_text(size = 20),  # Increase y-axis text size
    axis.title.x = element_text(size = 22), # Increase x-axis title size
    axis.title.y = element_text(size = 22)  # Increase y-axis title size
  )
```

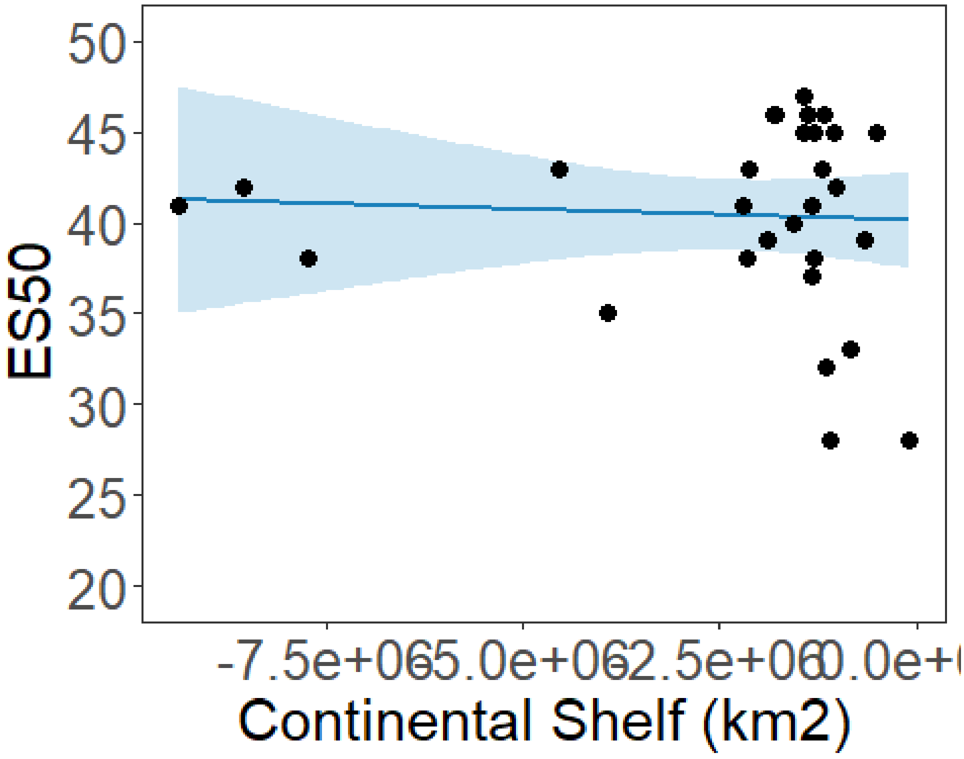


```
ggplot(Ecological_Data_Global, aes(x = CurVel_Mean, y = ES50_Sha)) +
  geom_smooth(method = "glm", formula = y ~ x, color = "#1a80bb", fill =
"#85bede") +  # Add a smooth dark blue line with light blue shadow
  geom_point(size = 3) +  # Add scatter plot points
  theme_bw() +  # Use the black and white theme
  scale_y_continuous(limits=c(18, 52), breaks=seq(20, 50, by=5), expand =
c(0, 0)) +
  labs(
    x = "Current Velocity (m.s-1)",  # Shorten the x-axis title
    y = "ES50"  # Shorten the y-axis title
  ) +
  theme(
    panel.grid.minor = element_blank(),
    panel.grid.major = element_blank(),
    axis.text.x = element_text(size =20),  # Increase x-axis text size
    axis.text.y = element_text(size = 20),  # Increase y-axis text size
    axis.title.x = element_text(size = 22), # Increase x-axis title size
    axis.title.y = element_text(size = 22)  # Increase y-axis title size
  )
```

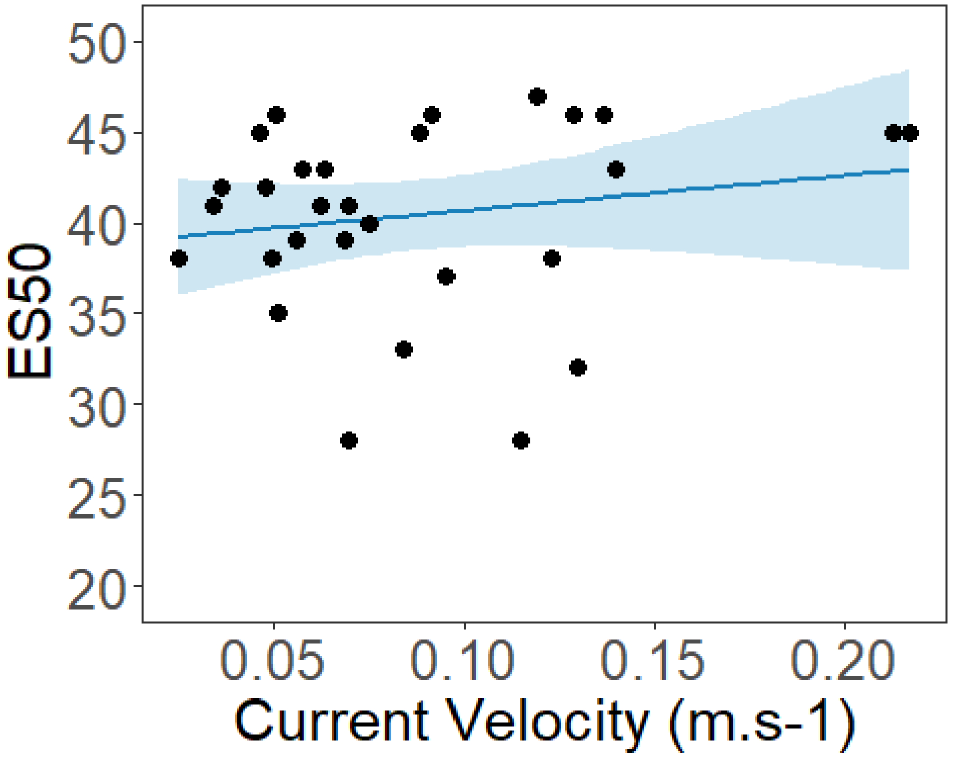


```
ggplot(Ecological_Data_Global, aes(x = Depth_Mean, y = ES50_Sha)) +
  geom_smooth(method = "glm", formula = y ~ x, color = "#1a80bb", fill =
"#85bede") +  # Add a smooth dark blue line with light blue shadow
  geom_point(size = 3) +  # Add scatter plot points
  theme_bw() +  # Use the black and white theme
  scale_x_continuous(labels = scales::scientific) +
  scale_y_continuous(limits=c(18, 52), breaks=seq(20, 50, by=5), expand =
c(0, 0)) +
  labs(
    x = "Depth (m)",  # Shorten the x-axis title
    y = "ES50"  # Shorten the y-axis title
  ) +
  theme(
    panel.grid.minor = element_blank(),
    panel.grid.major = element_blank(),
    axis.text.x = element_text(size =20),  # Increase x-axis text size
    axis.text.y = element_text(size = 20),  # Increase y-axis text size
    axis.title.x = element_text(size = 22), # Increase x-axis title size
    axis.title.y = element_text(size = 22)  # Increase y-axis title size
  )
```

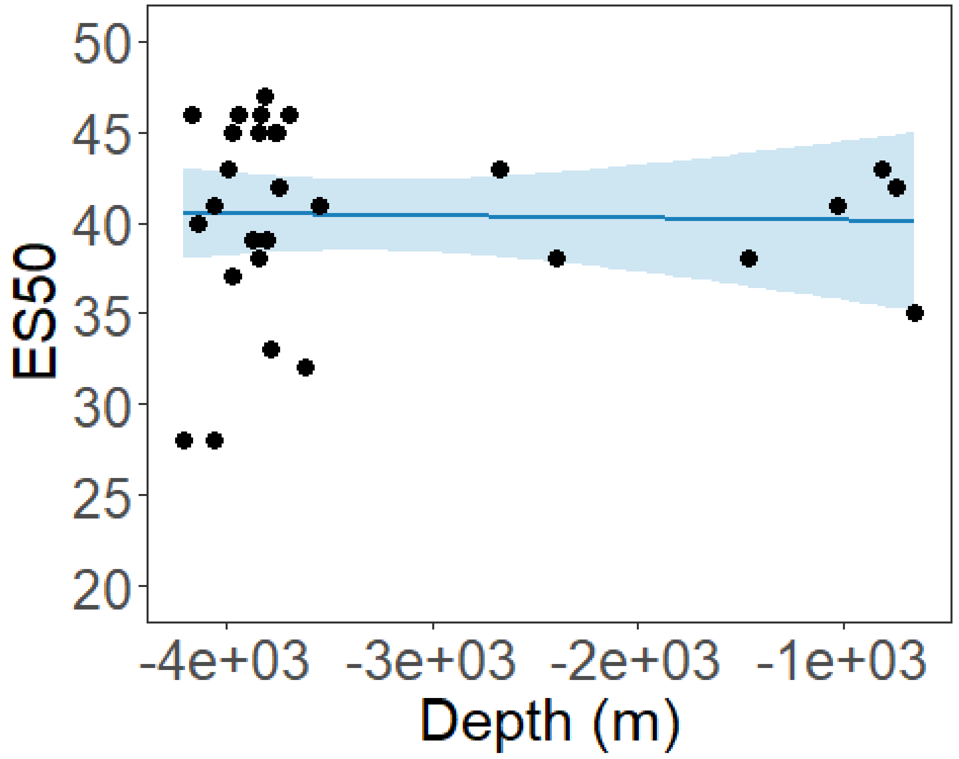


```
ggplot(Ecological_Data_Global, aes(x = HumImp_Mean, y = ES50_Sha)) +
  geom_smooth(method = "glm", formula = y ~ x, color = "#1a80bb", fill =
"#85bede") +  # Add a smooth dark blue line with light blue shadow
  geom_point(size = 3) +  # Add scatter plot points
  theme_bw() +  # Use the black and white theme
  scale_y_continuous(limits=c(18, 52), breaks=seq(20, 50, by=5), expand =
c(0, 0)) +
  labs(
    x = "Human Impact",  # Shorten the x-axis title
    y = "ES50"  # Shorten the y-axis title
  ) +
  theme(
    panel.grid.minor = element_blank(),
    panel.grid.major = element_blank(),
    axis.text.x = element_text(size =20),  # Increase x-axis text size
    axis.text.y = element_text(size = 20),  # Increase y-axis text size
    axis.title.x = element_text(size = 22), # Increase x-axis title size
    axis.title.y = element_text(size = 22)  # Increase y-axis title size
  )
```

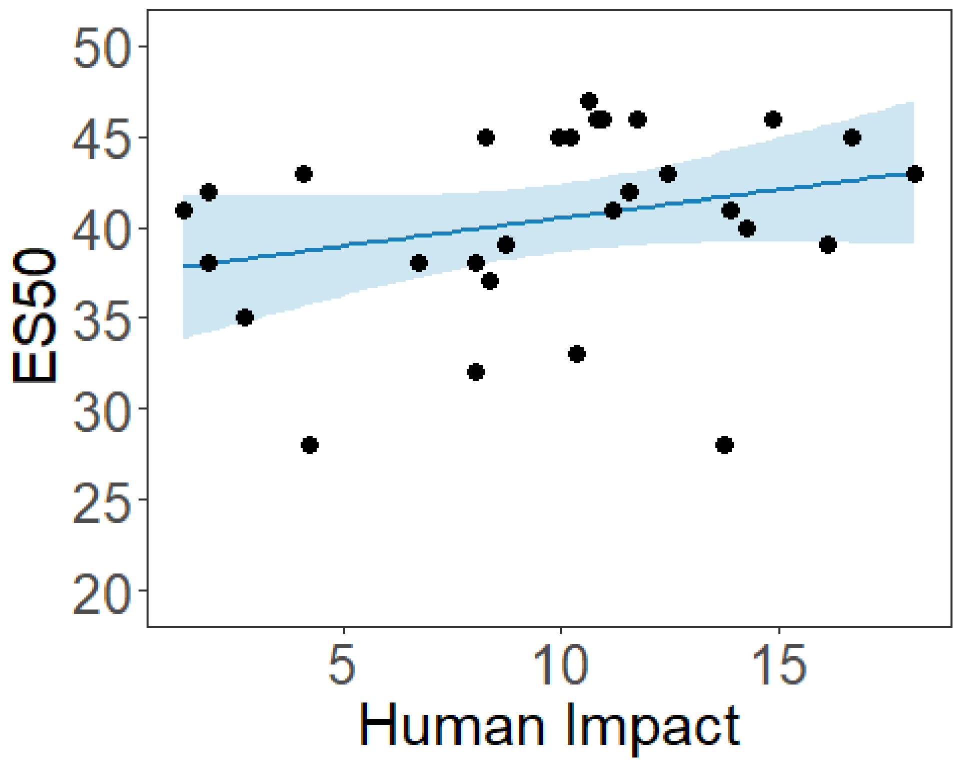


```r
ggplot(Ecological_Data_Global, aes(x = IceCov_Mean, y = ES50_Sha)) +
  geom_smooth(method = "glm", formula = y ~ x, color = "#1a80bb", fill =
"#85bede") +  # Add a smooth dark blue line with light blue shadow
  geom_point(size = 3) +  # Add scatter plot points
  theme_bw() +  # Use the black and white theme
  scale_y_continuous(limits=c(18, 52), breaks=seq(20, 50, by=5), expand =
c(0, 0)) +
  labs(
    x = "Ice Cover (fraction)",  # Shorten the x-axis title
    y = "ES50"  # Shorten the y-axis title
  ) +
  theme(
    panel.grid.minor = element_blank(),
    panel.grid.major = element_blank(),
    axis.text.x = element_text(size =20),  # Increase x-axis text size
    axis.text.y = element_text(size = 20),  # Increase y-axis text size
    axis.title.x = element_text(size = 22), # Increase x-axis title size
    axis.title.y = element_text(size = 22)  # Increase y-axis title size
  )
```

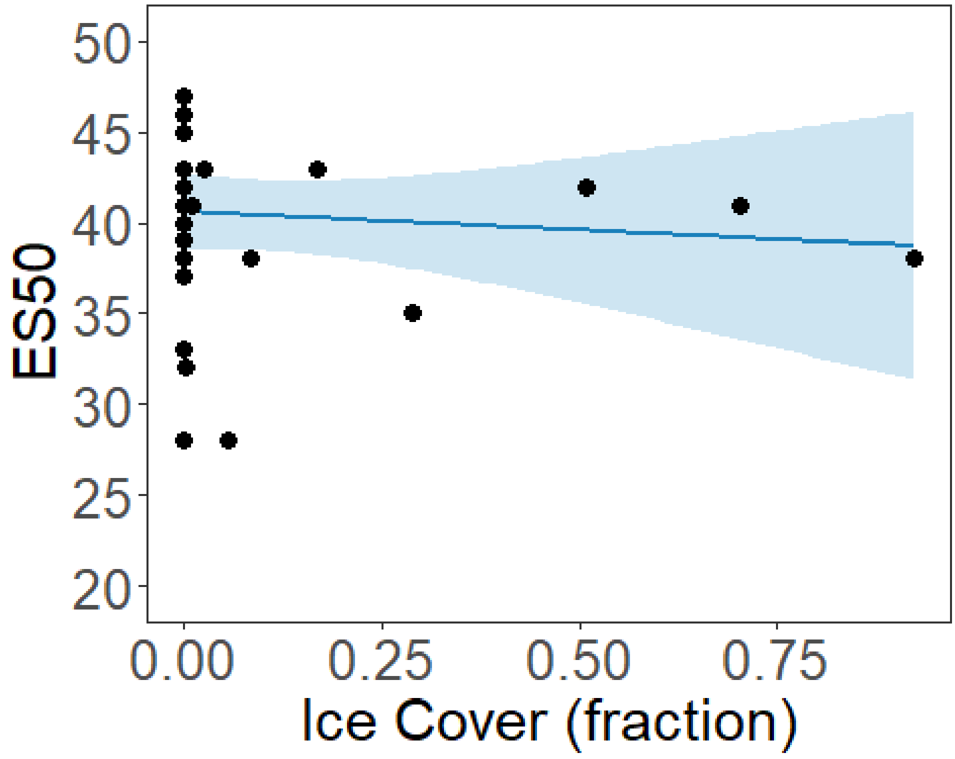


```
ggplot(Ecological_Data_Global, aes(x = PrimProd_Mean, y = ES50_Sha)) +
  geom_smooth(method = "glm", formula = y ~ x, color = "#1a80bb", fill =
"#85bede") +  # Add a smooth dark blue line with light blue shadow
  geom_point(size = 3) +  # Add scatter plot points
  theme_bw() +  # Use the black and white theme
  scale_y_continuous(limits=c(18, 52), breaks=seq(20, 50, by=5), expand =
c(0, 0)) +
  labs(
    x = "Primary Productivity (mmol . m-3)",  # Shorten the x-axis title
    y = "ES50"  # Shorten the y-axis title
  ) +
  theme(
    panel.grid.minor = element_blank(),
    panel.grid.major = element_blank(),
    axis.text.x = element_text(size =20),  # Increase x-axis text size
    axis.text.y = element_text(size = 20),  # Increase y-axis text size
    axis.title.x = element_text(size = 22), # Increase x-axis title size
    axis.title.y = element_text(size = 22)  # Increase y-axis title size
  )
```

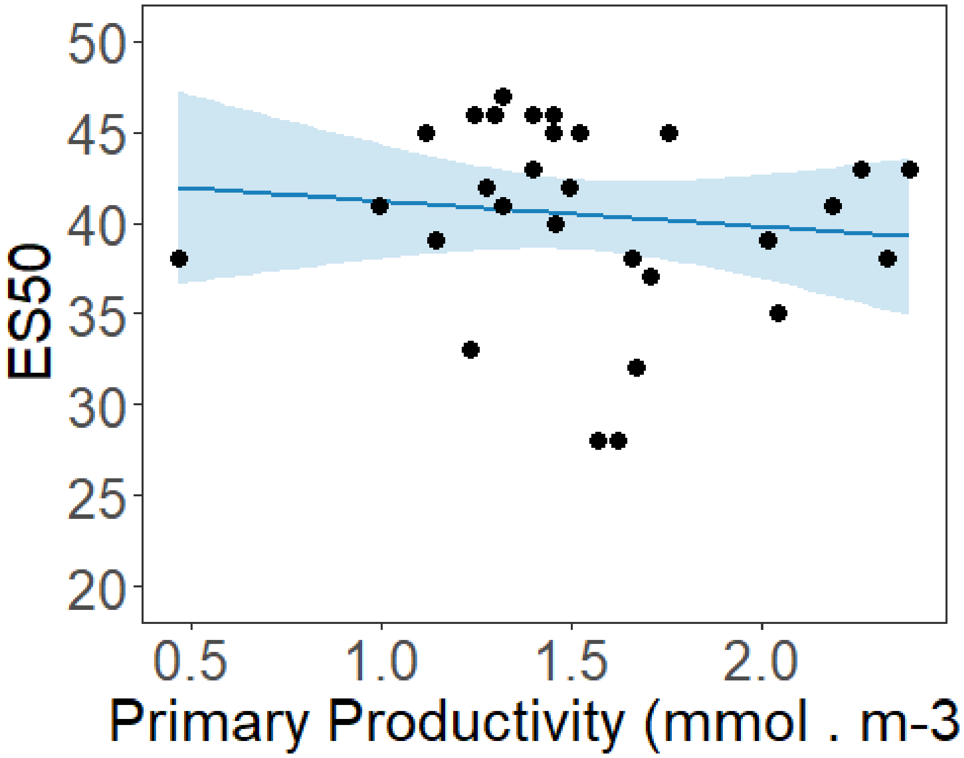


```
ggplot(Ecological_Data_Global, aes(x = ThemM_Mean, y = ES50_Sha)) +
  geom_smooth(method = "glm", formula = y ~ x, color = "#1a80bb", fill =
"#85bede") +  # Add a smooth dark blue line with light blue shadow
  geom_point(size = 3) +  # Add scatter plot points
  theme_bw() +  # Use the black and white theme
  scale_y_continuous(limits=c(18, 52), breaks=seq(20, 50, by=5), expand =
c(0, 0)) +
  labs(
    x = "Temperature Mean (ºC)",  # Shorten the x-axis title
    y = "ES50"  # Shorten the y-axis title
  ) +
  theme(
    panel.grid.minor = element_blank(),
    panel.grid.major = element_blank(),
    axis.text.x = element_text(size =20),  # Increase x-axis text size
    axis.text.y = element_text(size = 20),  # Increase y-axis text size
    axis.title.x = element_text(size = 22), # Increase x-axis title size
    axis.title.y = element_text(size = 22)  # Increase y-axis title size
  )
```

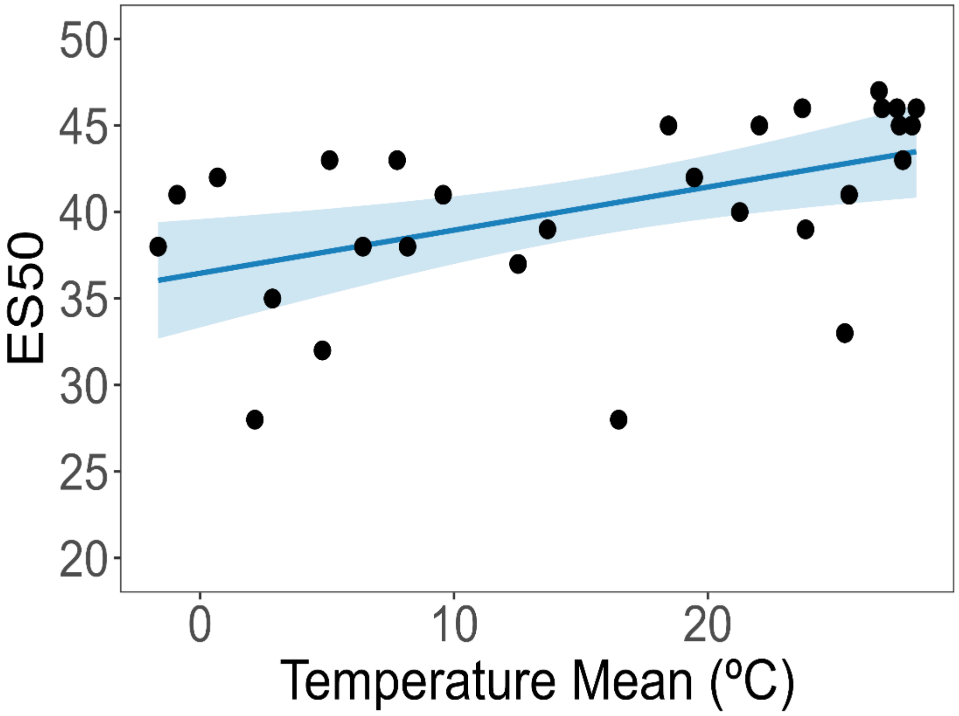


```
ggplot(Ecological_Data_Global, aes(x = Area_Sum, y = ES50_Sha)) +
  geom_smooth(method = "glm", formula = y ~ x, color = "#1a80bb", fill =
"#85bede") +  # Add a smooth dark blue line with light blue shadow
  geom_point(size = 3) +  # Add scatter plot points
  theme_bw() +  # Use the black and white theme
  scale_x_continuous(labels = scales::scientific) +
  scale_y_continuous(limits=c(18, 52), breaks=seq(20, 50, by=5), expand =
c(0, 0)) +
  labs(
    x = "Area (km2)",  # Shorten the x-axis title
    y = "ES50"  # Shorten the y-axis title
  ) +
  theme(
    panel.grid.minor = element_blank(),
    panel.grid.major = element_blank(),
    axis.text.x = element_text(size =20),  # Increase x-axis text size
    axis.text.y = element_text(size = 20),  # Increase y-axis text size
    axis.title.x = element_text(size = 22), # Increase x-axis title size
    axis.title.y = element_text(size = 22)  # Increase y-axis title size
  )
```

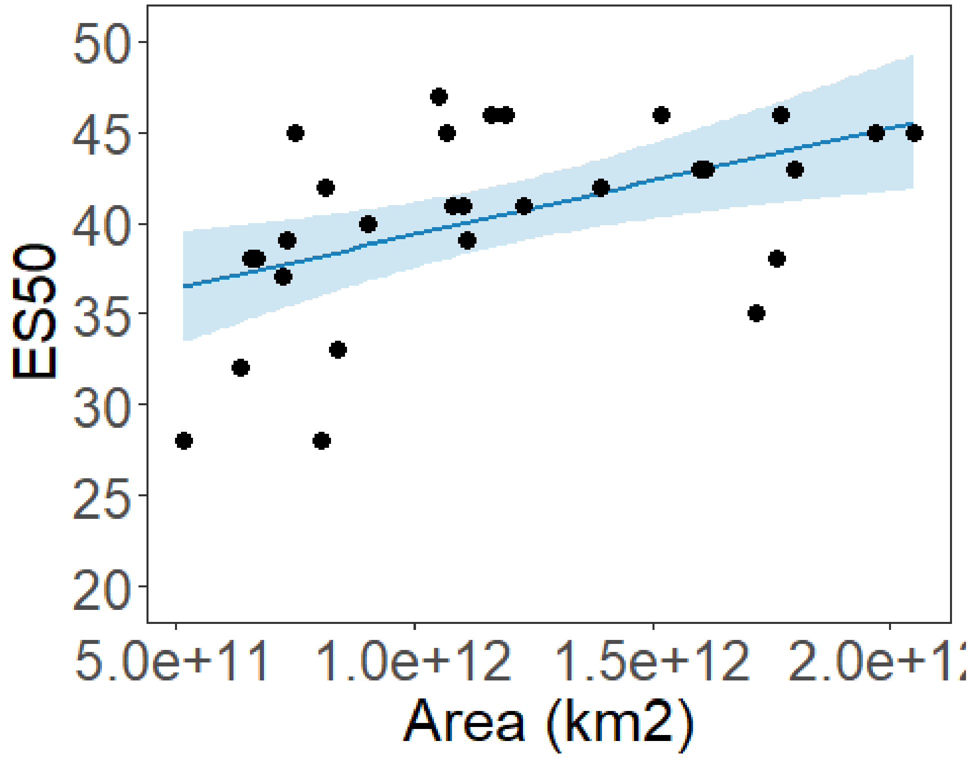


```r
ggplot(Ecological_Data_Global, aes(x = Nitrate_Mean, y = ES50_Sha)) +
  geom_smooth(method = "glm", formula = y ~ x, color = "#1a80bb", fill =
"#85bede") +  # Add a smooth dark blue line with light blue shadow
  geom_point(size = 3) +  # Add scatter plot points
  theme_bw() +  # Use the black and white theme
  scale_y_continuous(limits=c(18, 52), breaks=seq(20, 50, by=5), expand =
c(0, 0)) +
  labs(
    x = "Nitrate (mmol . m-3)",  # Shorten the x-axis title
    y = "ES50"  # Shorten the y-axis title
  ) +
  theme(
    panel.grid.minor = element_blank(),
    panel.grid.major = element_blank(),
    axis.text.x = element_text(size =20),  # Increase x-axis text size
    axis.text.y = element_text(size = 20),  # Increase y-axis text size
    axis.title.x = element_text(size = 22), # Increase x-axis title size
    axis.title.y = element_text(size = 22)  # Increase y-axis title size
  )
```

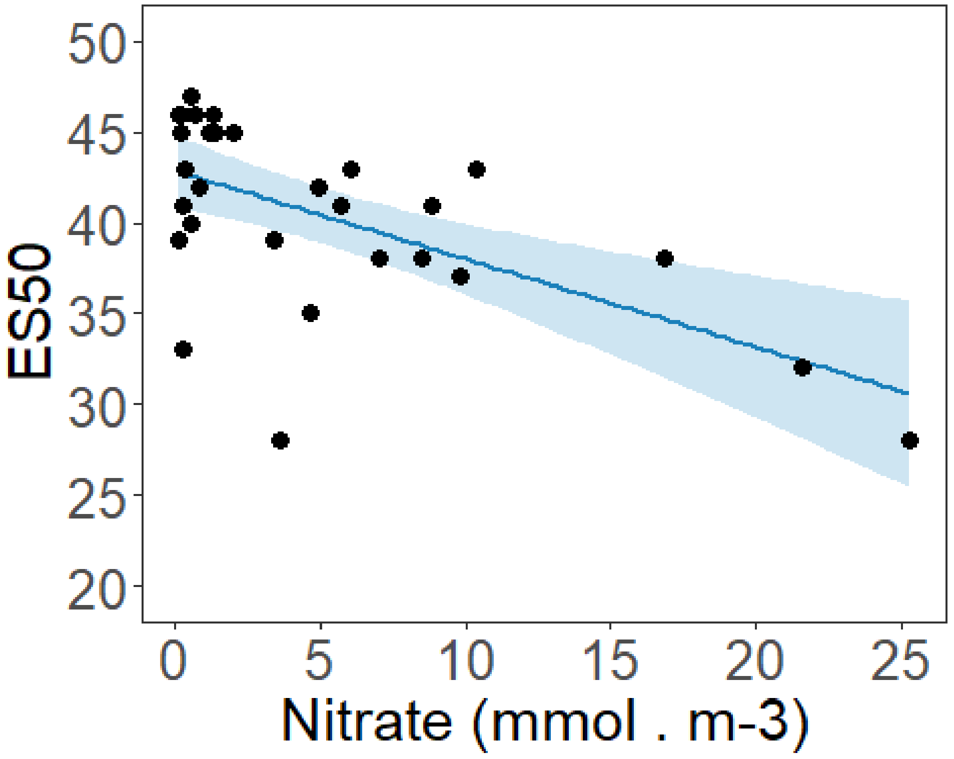

ES50
50
45
40
35
30
25
20
0
5
10
15
20
25
Nitrate (mmol . m-3)

Markdown_GLM_Meso.R

hsaeedi

2025-06-07

```
# ===================================
#                 MESO-NUMSPe-GLM
# ===================================
library(readxl)

## Warning: package 'readxl' was built under R version 4.3.3

library(openxlsx)

## Warning: package 'openxlsx' was built under R version 4.3.3

library(tidyverse)

## Warning: package 'tidyverse' was built under R version 4.3.3

## Warning: package 'ggplot2' was built under R version 4.3.3

## Warning: package 'tibble' was built under R version 4.3.3

## Warning: package 'tidyr' was built under R version 4.3.3

## Warning: package 'readr' was built under R version 4.3.3

## Warning: package 'purrr' was built under R version 4.3.3

## Warning: package 'dplyr' was built under R version 4.3.3

## Warning: package 'stringr' was built under R version 4.3.3

## Warning: package 'forcats' was built under R version 4.3.3

## Warning: package 'lubridate' was built under R version 4.3.3

## ── Attaching core tidyverse packages ──────────────────────── tidyvers
e 2.0.0 ──
## ✔ dplyr     1.1.4     ✔ readr     2.1.5
## ✔ forcats   1.0.0     ✔ stringr   1.5.1
## ✔ ggplot2   3.5.1     ✔ tibble    3.2.1
## ✔ lubridate 1.9.3     ✔ tidyr     1.3.1
## ✔ purrr     1.0.2
## ── Conflicts ────────────────────────────────────────── tidyverse_conf
licts() ──
## ✖ dplyr::filter() masks stats::filter()
## ✖ dplyr::lag()    masks stats::lag()
## ℹ Use the conflicted package (<http://conflicted.r-lib.org/>) to for
ce all conflicts to become errors

library(sf)
```

```r
## Warning: package 'sf' was built under R version 4.3.3

## Linking to GEOS 3.11.2, GDAL 3.8.2, PROJ 9.3.1; sf_use_s2() is TRUE

library(vegan)

## Warning: package 'vegan' was built under R version 4.3.3

## Loading required package: permute

## Warning: package 'permute' was built under R version 4.3.3

## Loading required package: lattice
## This is vegan 2.6-4

library(pvclust)

## Warning: package 'pvclust' was built under R version 4.3.3

library(dplyr)
library(ggplot2)
library(robis)

## Warning: package 'robis' was built under R version 4.3.3

library(obistools)
library(nortest) # for Anderson-Darling test
library(stringi) # for encoding UTF-8

## Warning: package 'stringi' was built under R version 4.3.3

library(corrplot)

## Warning: package 'corrplot' was built under R version 4.3.3

## corrplot 0.94 loaded

library(mgcv)

## Loading required package: nlme
##
## Attaching package: 'nlme'
##
## The following object is masked from 'package:dplyr':
##
##     collapse
##
## This is mgcv 1.9-0. For overview type 'help("mgcv-package")'.

library(ggeffects)

## Warning: package 'ggeffects' was built under R version 4.3.3

library(DHARMa) #simulations package gam-

## Warning: package 'DHARMa' was built under R version 4.3.3
```

```
## Warning in check_dep_version(): ABI version mismatch:
## lme4 was built with Matrix ABI version 1
## Current Matrix ABI version is 0
## Please re-install lme4 from source or restore original 'Matrix' packag
e

## This is DHARMa 0.4.6. For overview type '?DHARMa'. For recent changes,
type news(package = 'DHARMa')

library(knitr)

## Warning: package 'knitr' was built under R version 4.3.3

library(qpcR)

## Warning: package 'qpcR' was built under R version 4.3.3

## Loading required package: MASS
##
## Attaching package: 'MASS'
##
## The following object is masked from 'package:robis':
##
##     area
##
## The following object is masked from 'package:dplyr':
##
##     select
##
## Loading required package: minpack.lm

## Warning: package 'minpack.lm' was built under R version 4.3.3

## Loading required package: rgl

## Warning: package 'rgl' was built under R version 4.3.3

## Loading required package: robustbase

## Warning: package 'robustbase' was built under R version 4.3.3

## Loading required package: Matrix
##
## Attaching package: 'Matrix'
##
## The following objects are masked from 'package:tidyr':
##
##     expand, pack, unpack

#Species Counts and Environment, 5 degree bands
Ecological_Data_Global <- read.csv("Ecological_Data_Global.csv", sep = ";
")
summary(Ecological_Data_Global)
```

```
##       id             left           top              right         bot
tom
##  Min.   : 1.00   Min.   :-180   Min.   :-85.00   Min.   :180   Min.   
:-90.00  
##  1st Qu.: 9.75   1st Qu.:-180   1st Qu.:-41.25   1st Qu.:180   1st Qu.
:-46.25  
##  Median :18.50   Median :-180   Median :  2.50   Median :180   Median 
: -2.50  
##  Mean   :18.50   Mean   :-180   Mean   :  2.50   Mean   :180   Mean   
: -2.50  
##  3rd Qu.:27.25   3rd Qu.:-180   3rd Qu.: 46.25   3rd Qu.:180   3rd Qu.
: 41.25  
##  Max.   :36.00   Max.   :-180   Max.   : 90.00   Max.   :180   Max.   
: 85.00  
##                                                                        
##  Bathy_Max_Mean    Bathy_Mean_Mean   Bathy_Min_Mean    Chl_Mean_Mean   
##  Min.   :-4278.5   Min.   :-4194.3   Min.   :-4117.6   Min.   :0.09031  
##  1st Qu.:-4058.1   1st Qu.:-3970.8   1st Qu.:-3889.3   1st Qu.:0.13366  
##  Median :-3874.8   Median :-3790.1   Median :-3702.5   Median :0.25032  
##  Mean   :-3292.2   Mean   :-3225.1   Mean   :-3154.7   Mean   :0.26540  
##  3rd Qu.:-2944.1   3rd Qu.:-2883.2   3rd Qu.:-2820.7   3rd Qu.:0.37041  
##  Max.   : -761.9   Max.   : -741.7   Max.   : -721.3   Max.   :0.50726  
##  NA's   :2         NA's   :2         NA's   :2         NA's   :2        
##    Margin_Sum           Bathy_Max           Shelf_Sum         CurVel_
Mean    
##  Min.   :-322305404   Min.   :-3.072e+09   Min.   :-9362904   Min.   :
0.02525  
##  1st Qu.:-152911004   1st Qu.:-2.251e+09   1st Qu.:-1834335   1st Qu.:
0.04832  
##  Median :-110339888   Median :-2.014e+09   Median :-1300462   Median :
0.06613  
##  Mean   :-113889802   Mean   :-1.666e+09   Mean   :-1872021   Mean   :
0.08048  
##  3rd Qu.: -70897881   3rd Qu.:-7.886e+08   3rd Qu.: -705476   3rd Qu.:
0.11028  
##  Max.   :         0   Max.   : 0.000e+00   Max.   :       0   Max.   :
0.21723  
##                                                               NA's   :
2        
##  CurVel_Bot_Mean    Diff_Atte_Mean      Depth_Mean        HumImp_Sum   
##  Min.   :0.002405   Min.   :0.02947   Min.   :-4204.0   Min.   :      
0  
##  1st Qu.:0.014127   1st Qu.:0.06059   1st Qu.:-3962.4   1st Qu.:  8151
342  
##  Median :0.017665   Median :0.07062   Median :-3776.1   Median : 56012
445  
##  Mean   :0.018497   Mean   :0.07477   Mean   :-3204.4   Mean   : 68495
091  
##  3rd Qu.:0.020965   3rd Qu.:0.08153   3rd Qu.:-2826.0   3rd Qu.:103706
926  
##  Max.   :0.045673   Max.   :0.12402   Max.   : -655.6   Max.   :195804
```

```
477
##  NA's   :2          NA's   :2         NA's   :2         NA's   :4
##   HumImp_Mean     Ice_Cover_Sum      IceCov_Mean        Ice_Tick_Mean
##  Min.   : 1.342   Min.   :     0.0   Min.   :0.0000000   Min.   :0.000
0000
##  1st Qu.: 8.030   1st Qu.:     0.0   1st Qu.:0.0000000   1st Qu.:0.000
0000
##  Median :10.361   Median :     0.5   Median :0.0000077   Median :0.000
0115
##  Mean   : 9.725   Mean   : 81161.0   Mean   :0.1808575   Mean   :0.323
9475
##  3rd Qu.:12.446   3rd Qu.: 44888.8   3rd Qu.:0.2563673   3rd Qu.:0.286
3370
##  Max.   :18.138   Max.   :696353.9   Max.   :0.9671582   Max.   :2.458
3182
##  NA's   :7                           NA's   :2           NA's   :2
##   MixLay_Mean     PhotoActi_Mean   PrimProd_Mean    Salinity_Mean
##  Min.   : 19.95   Min.   : 3.877   Min.   :0.3574   Min.   :30.31
##  1st Qu.: 30.85   1st Qu.:24.399   1st Qu.:1.3064   1st Qu.:33.11
##  Median : 40.91   Median :28.955   Median :1.5059   Median :34.13
##  Mean   : 45.60   Mean   :32.202   Mean   :1.5172   Mean   :33.96
##  3rd Qu.: 56.22   3rd Qu.:43.915   3rd Qu.:1.6973   3rd Qu.:35.00
##  Max.   :103.59   Max.   :48.163   Max.   :2.3878   Max.   :35.67
##  NA's   :2        NA's   :2        NA's   :2        NA's   :2
##  Silicate_Mean      ThemM_Mean     Them_Max_Mean     Them_Max_Max
##  Min.   : 1.556   Min.   :-1.721   Min.   :-1.524   Min.   :-0.8473
##  1st Qu.: 2.356   1st Qu.: 2.327   1st Qu.: 6.581   1st Qu.:14.8983
##  Median : 5.867   Median :13.104   Median :18.786   Median :27.7568
##  Mean   :16.058   Mean   :13.340   Mean   :16.923   Mean   :23.2399
##  3rd Qu.:13.556   3rd Qu.:25.013   3rd Qu.:27.983   3rd Qu.:32.6277
##  Max.   :83.166   Max.   :28.215   Max.   :29.775   Max.   :36.4851
##  NA's   :2        NA's   :2        NA's   :2        NA's   :2
##  Temp_Min_Mean      Temp_Min_Min      ThemR_Mean      Them_Range_Max
##  Min.   :-1.95401   Min.   :-2.000   Min.   : 0.297   Min.   : 0.7454
##  1st Qu.:-0.01615   1st Qu.:-2.000   1st Qu.: 4.105   1st Qu.: 9.4402
##  Median : 8.38265   Median :-1.030   Median : 5.556   Median :11.1749
##  Mean   :10.64040   Mean   : 4.492   Mean   : 6.284   Mean   :12.6015
##  3rd Qu.:21.91526   3rd Qu.:11.822   3rd Qu.: 8.375   3rd Qu.:16.9521
##  Max.   :26.38238   Max.   :19.556   Max.   :13.863   Max.   :24.1779
##  NA's   :2          NA's   :2        NA's   :2        NA's   :2
##     Area_Sum            O2_Mean       Nitrate_Mean     Nitrate_Bot_Mea
n
##  Min.   :1.020e+11   Min.   :203.1   Min.   : 0.1658   Min.   :10.42
##  1st Qu.:6.495e+11   1st Qu.:212.0   1st Qu.: 0.5914   1st Qu.:26.76
##  Median :9.760e+11   Median :268.6   Median : 4.1394   Median :31.54
##  Mean   :9.857e+11   Mean   :273.6   Mean   : 7.9129   Mean   :27.32
##  3rd Qu.:1.422e+12   3rd Qu.:325.9   3rd Qu.: 9.5803   3rd Qu.:32.03
##  Max.   :2.050e+12   Max.   :371.1   Max.   :29.3414   Max.   :32.78
##                      NA's   :2       NA's   :2         NA's   :2
##   O2_Bot_Mean     PrimProd_Bot_Mean Salinity_Bot_Mean ThemM_Bot_Mean
##  Min.   :167.8   Min.   :0.01356   Min.   :32.22     Min.   :-1.0599
```

```
##   1st Qu.:184.0   1st Qu.:0.04705   1st Qu.:34.61     1st Qu.: 0.2374
##   Median :199.6   Median :0.09406   Median :34.69     Median : 2.0996
##   Mean   :219.7   Mean   :0.14575   Mean   :34.47     Mean   : 1.7394
##   3rd Qu.:257.0   3rd Qu.:0.16129   3rd Qu.:34.74     3rd Qu.: 2.9030
##   Max.   :314.2   Max.   :0.68720   Max.   :35.10     Max.   : 3.6338
##   NA's   :2       NA's   :2         NA's   :2         NA's   :2
##   ThemR_Bot_Mean        NumSpe          NumRec             NumPhy
##   Min.   :0.04462   Min.   :  494   Min.   :  28649   Min.   :17.00
##   1st Qu.:0.44255   1st Qu.: 6122   1st Qu.: 315808   1st Qu.:24.00
##   Median :0.53982   Median :16326   Median : 840696   Median :25.50
##   Mean   :0.86086   Mean   :16924   Mean   :1411624   Mean   :25.26
##   3rd Qu.:0.97004   3rd Qu.:25489   3rd Qu.:1893681   3rd Qu.:27.75
##   Max.   :3.53683   Max.   :38304   Max.   :7566285   Max.   :30.00
##   NA's   :2         NA's   :2       NA's   :2         NA's   :2
##        ES50         NumSpe_Sha      NumRec_Sha         NumPhy_Sha
##   Min.   :23.68   Min.   :  271   Min.   :   7056   Min.   :13.00
##   1st Qu.:39.81   1st Qu.: 4037   1st Qu.: 182153   1st Qu.:21.00
##   Median :42.21   Median :10746   Median : 496946   Median :23.00
##   Mean   :41.19   Mean   :10657   Mean   :1115969   Mean   :22.76
##   3rd Qu.:45.60   3rd Qu.:15559   3rd Qu.:1232565   3rd Qu.:26.00
##   Max.   :48.28   Max.   :25317   Max.   :7402048   Max.   :29.00
##   NA's   :2       NA's   :2       NA's   :2         NA's   :2
##      ES50_Sha       NumSpe_Mes      NumRec_Mes       NumPhy_Mes
##   Min.   :18.00   Min.   : 141   Min.   :  2391   Min.   :10.00
##   1st Qu.:35.25   1st Qu.:2937   1st Qu.: 22966   1st Qu.:17.00
##   Median :40.50   Median :3455   Median : 40485   Median :18.50
##   Mean   :38.53   Mean   :3689   Mean   :116802   Mean   :18.29
##   3rd Qu.:44.50   3rd Qu.:5003   3rd Qu.:110266   3rd Qu.:20.00
##   Max.   :47.00   Max.   :7275   Max.   :650912   Max.   :23.00
##   NA's   :2       NA's   :2      NA's   :2        NA's   :2
##      ES50_Mes       NumSpe_Dee      NumRec_Dee       NumPhy_Dee
##   Min.   :29.00   Min.   : 411   Min.   :  9251   Min.   :15.00
##   1st Qu.:39.00   1st Qu.:2920   1st Qu.: 21008   1st Qu.:18.00
##   Median :42.00   Median :3992   Median : 39600   Median :19.50
##   Mean   :42.21   Mean   :4270   Mean   : 80043   Mean   :20.12
##   3rd Qu.:47.00   3rd Qu.:5841   3rd Qu.: 97697   3rd Qu.:22.00
##   Max.   :49.00   Max.   :8735   Max.   :556993   Max.   :27.00
##   NA's   :2       NA's   :2      NA's   :2        NA's   :2
##      ES50_Dee
##   Min.   :29.00
##   1st Qu.:40.00
##   Median :42.50
##   Mean   :42.53
##   3rd Qu.:45.00
##   Max.   :49.00
##   NA's   :2

#First we're going to load in our data and then trim the data frame down
to just the columns we need.
analysis.cols <- c("Margin_Sum","CurVel_Mean", "Depth_Mean", "HumImp_Mean
",
```

```
                      "PrimProd_Mean", "ThemM_Mean", "Area_Sum", "Nitrate_Me
an", "NumSpe_Mes",
                      "NumRec_Mes", "NumPhy_Mes", "ES50_Mes")
Ecological_Data_Global <- Ecological_Data_Global[,analysis.cols]
Ecological_Data_Global  <- Ecological_Data_Global [complete.cases(Ecologi
cal_Data_Global ),]

# Calculate the correlation matrix
corr_matrix <- cor(Ecological_Data_Global)

# Create the correlation plot with black font for text
corrplot(corr_matrix, tl.col = "black")
```

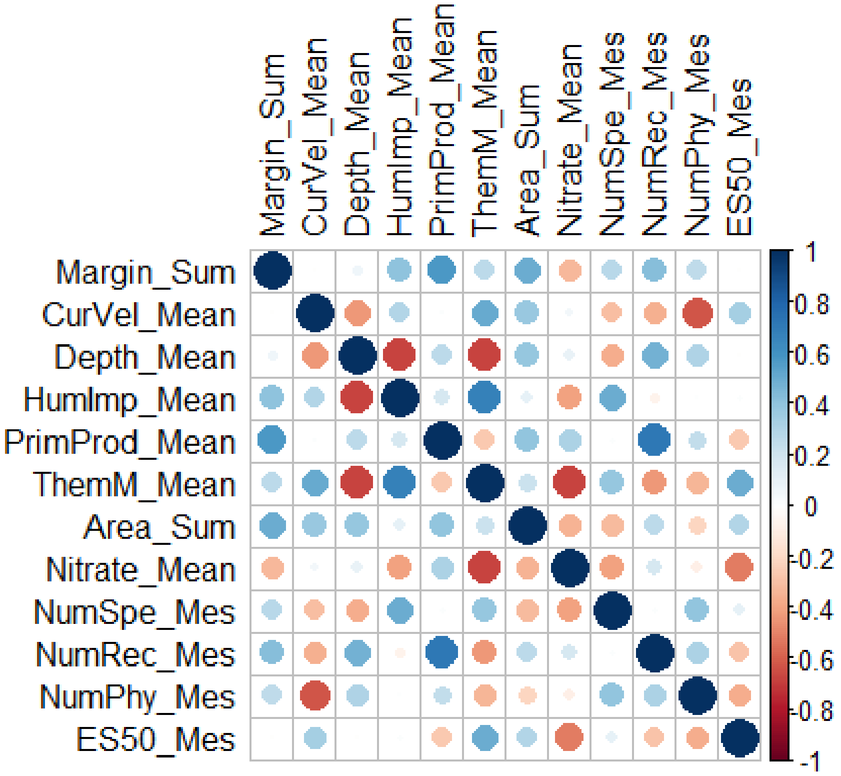


```
#GLMs for number of species, Global data, Meso
meso.numsp.intercept <- glm(NumSpe_Mes ~ 1, family = "poisson", data = Ec
ological_Data_Global)
summary(meso.numsp.intercept)

##
## Call:
## glm(formula = NumSpe_Mes ~ 1, family = "poisson", data = Ecological_Da
ta_Global)
##
## Coefficients:
##             Estimate Std. Error z value Pr(>|z|)
## (Intercept) 8.261455   0.002984    2768   <2e-16 ***
## ---
## Signif. codes:  0 '***' 0.001 '**' 0.01 '*' 0.05 '.' 0.1 ' ' 1
##
## (Dispersion parameter for poisson family taken to be 1)
```

```
##
##     Null deviance: 20817  on 28  degrees of freedom
## Residual deviance: 20817  on 28  degrees of freedom
## AIC: 21108
##
## Number of Fisher Scoring iterations: 4
```

```
meso.numsp.numrec <- glm(NumSpe_Mes ~ NumRec_Mes, family = "poisson", dat
a = Ecological_Data_Global)
summary(meso.numsp.numrec)
```

```
##
## Call:
## glm(formula = NumSpe_Mes ~ NumRec_Mes, family = "poisson", data = Ecol
ogical_Data_Global)
##
## Coefficients:
##              Estimate Std. Error  z value Pr(>|z|)
## (Intercept) 8.256e+00  3.753e-03 2199.773   <2e-16 ***
## NumRec_Mes  4.343e-08  1.694e-08    2.564   0.0104 *
## ---
## Signif. codes:  0 '***' 0.001 '**' 0.01 '*' 0.05 '.' 0.1 ' ' 1
##
## (Dispersion parameter for poisson family taken to be 1)
##
##     Null deviance: 20817  on 28  degrees of freedom
## Residual deviance: 20810  on 27  degrees of freedom
## AIC: 21104
##
## Number of Fisher Scoring iterations: 4
```

```
meso.numsp.marginsum <- glm(NumSpe_Mes ~ NumRec_Mes + Margin_Sum, family
= "poisson", data = Ecological_Data_Global)
summary(meso.numsp.marginsum)
```

```
##
## Call:
## glm(formula = NumSpe_Mes ~ NumRec_Mes + Margin_Sum, family = "poisson"
,
##     data = Ecological_Data_Global)
##
## Coefficients:
##               Estimate Std. Error z value Pr(>|z|)
## (Intercept)  8.612e+00  9.354e-03  920.72   <2e-16 ***
## NumRec_Mes  -2.944e-07  1.894e-08  -15.54   <2e-16 ***
## Margin_Sum   2.707e-09  6.681e-11   40.52   <2e-16 ***
## ---
## Signif. codes:  0 '***' 0.001 '**' 0.01 '*' 0.05 '.' 0.1 ' ' 1
##
## (Dispersion parameter for poisson family taken to be 1)
##
##     Null deviance: 20817  on 28  degrees of freedom
```

```
## Residual deviance: 19053  on 26  degrees of freedom
## AIC: 19349
##
## Number of Fisher Scoring iterations: 5
```

```
meso.numsp.current <- glm(NumSpe_Mes ~ NumRec_Mes + CurVel_Mean, family =
"poisson", data = Ecological_Data_Global)
summary(meso.numsp.current)
```

```
##
## Call:
## glm(formula = NumSpe_Mes ~ NumRec_Mes + CurVel_Mean, family = "poisson
",
##     data = Ecological_Data_Global)
##
## Coefficients:
##                Estimate Std. Error z value Pr(>|z|)
## (Intercept)  8.556e+00  7.624e-03 1122.31   <2e-16 ***
## NumRec_Mes  -2.265e-07  1.816e-08  -12.47   <2e-16 ***
## CurVel_Mean -3.118e+00  7.159e-02  -43.55   <2e-16 ***
## ---
## Signif. codes:  0 '***' 0.001 '**' 0.01 '*' 0.05 '.' 0.1 ' ' 1
##
## (Dispersion parameter for poisson family taken to be 1)
##
##     Null deviance: 20817  on 28  degrees of freedom
## Residual deviance: 18832  on 26  degrees of freedom
## AIC: 19128
##
## Number of Fisher Scoring iterations: 4
```

```
meso.numsp.depth <- glm(NumSpe_Mes ~ NumRec_Mes + Depth_Mean, family = "p
oisson", data = Ecological_Data_Global)
summary(meso.numsp.depth)
```

```
##
## Call:
## glm(formula = NumSpe_Mes ~ NumRec_Mes + Depth_Mean, family = "poisson"
,
##     data = Ecological_Data_Global)
##
## Coefficients:
##                Estimate Std. Error z value Pr(>|z|)
## (Intercept)  7.500e+00  1.370e-02  547.50   <2e-16 ***
## NumRec_Mes   6.244e-07  1.917e-08   32.56   <2e-16 ***
## Depth_Mean  -2.010e-04  3.457e-06  -58.16   <2e-16 ***
## ---
## Signif. codes:  0 '***' 0.001 '**' 0.01 '*' 0.05 '.' 0.1 ' ' 1
##
## (Dispersion parameter for poisson family taken to be 1)
##
##     Null deviance: 20817  on 28  degrees of freedom
```

```
## Residual deviance: 17057  on 26  degrees of freedom
## AIC: 17352
##
## Number of Fisher Scoring iterations: 4
```

```
meso.numsp.humimp <- glm(NumSpe_Mes ~ NumRec_Mes + HumImp_Mean, family =
"poisson", data = Ecological_Data_Global)
summary(meso.numsp.humimp)
```

```
##
## Call:
## glm(formula = NumSpe_Mes ~ NumRec_Mes + HumImp_Mean, family = "poisson
",
##     data = Ecological_Data_Global)
##
## Coefficients:
##              Estimate Std. Error z value Pr(>|z|)
## (Intercept) 7.762e+00  8.303e-03 934.840  < 2e-16 ***
## NumRec_Mes  1.031e-07  1.702e-08   6.058 1.38e-09 ***
## HumImp_Mean 4.766e-02  6.866e-04  69.416  < 2e-16 ***
## ---
## Signif. codes:  0 '***' 0.001 '**' 0.01 '*' 0.05 '.' 0.1 ' ' 1
##
## (Dispersion parameter for poisson family taken to be 1)
##
##     Null deviance: 20817  on 28  degrees of freedom
## Residual deviance: 15871  on 26  degrees of freedom
## AIC: 16167
##
## Number of Fisher Scoring iterations: 4
```

```
meso.numsp.primprod <- glm(NumSpe_Mes ~ NumRec_Mes + PrimProd_Mean, famil
y = "poisson", data = Ecological_Data_Global)
summary(meso.numsp.primprod)
```

```
##
## Call:
## glm(formula = NumSpe_Mes ~ NumRec_Mes + PrimProd_Mean, family = "poiss
on",
##     data = Ecological_Data_Global)
##
## Coefficients:
##                Estimate Std. Error z value Pr(>|z|)
## (Intercept)   8.238e+00  1.396e-02 590.261   <2e-16 ***
## NumRec_Mes    2.076e-08  2.432e-08   0.854    0.393
## PrimProd_Mean 1.324e-02  1.019e-02   1.298    0.194
## ---
## Signif. codes:  0 '***' 0.001 '**' 0.01 '*' 0.05 '.' 0.1 ' ' 1
##
## (Dispersion parameter for poisson family taken to be 1)
##
##     Null deviance: 20817  on 28  degrees of freedom
```

```
## Residual deviance: 20808  on 26  degrees of freedom
## AIC: 21104
##
## Number of Fisher Scoring iterations: 5
```

```
meso.numsp.themmean <- glm(NumSpe_Mes ~ NumRec_Mes + ThemM_Mean, family =
"poisson", data = Ecological_Data_Global)
summary(meso.numsp.themmean)
```

```
##
## Call:
## glm(formula = NumSpe_Mes ~ NumRec_Mes + ThemM_Mean, family = "poisson"
,
##     data = Ecological_Data_Global)
##
## Coefficients:
##              Estimate Std. Error z value Pr(>|z|)
## (Intercept) 7.814e+00  8.548e-03  914.12   <2e-16 ***
## NumRec_Mes  6.339e-07  1.954e-08   32.44   <2e-16 ***
## ThemM_Mean  2.171e-02  3.602e-04   60.28   <2e-16 ***
## ---
## Signif. codes:  0 '***' 0.001 '**' 0.01 '*' 0.05 '.' 0.1 ' ' 1
##
## (Dispersion parameter for poisson family taken to be 1)
##
##     Null deviance: 20817  on 28  degrees of freedom
## Residual deviance: 16891  on 26  degrees of freedom
## AIC: 17187
##
## Number of Fisher Scoring iterations: 4
```

```
meso.numsp.area <- glm(NumSpe_Mes ~ NumRec_Mes + Area_Sum, family = "pois
son", data = Ecological_Data_Global)
summary(meso.numsp.area)
```

```
##
## Call:
## glm(formula = NumSpe_Mes ~ NumRec_Mes + Area_Sum, family = "poisson",
##     data = Ecological_Data_Global)
##
## Coefficients:
##               Estimate Std. Error z value Pr(>|z|)
## (Intercept)  8.614e+00  8.419e-03 1023.23   <2e-16 ***
## NumRec_Mes   2.960e-07  1.813e-08   16.32   <2e-16 ***
## Area_Sum    -3.410e-13  7.415e-15  -45.99   <2e-16 ***
## ---
## Signif. codes:  0 '***' 0.001 '**' 0.01 '*' 0.05 '.' 0.1 ' ' 1
##
## (Dispersion parameter for poisson family taken to be 1)
##
##     Null deviance: 20817  on 28  degrees of freedom
## Residual deviance: 18612  on 26  degrees of freedom
```

```
## AIC: 18908
##
## Number of Fisher Scoring iterations: 4
```

```
meso.numsp.nitrate <- glm(NumSpe_Mes ~ NumRec_Mes + Nitrate_Mean, family
= "poisson", data = Ecological_Data_Global)
summary(meso.numsp.nitrate)
```

```
##
## Call:
## glm(formula = NumSpe_Mes ~ NumRec_Mes + Nitrate_Mean, family = "poisso
n",
##     data = Ecological_Data_Global)
##
## Coefficients:
##                Estimate Std. Error z value Pr(>|z|)
## (Intercept)   8.371e+00  4.106e-03 2038.91   <2e-16 ***
## NumRec_Mes    3.002e-07  1.778e-08   16.88   <2e-16 ***
## Nitrate_Mean -3.322e-02  5.906e-04  -56.25   <2e-16 ***
## ---
## Signif. codes:  0 '***' 0.001 '**' 0.01 '*' 0.05 '.' 0.1 ' ' 1
##
## (Dispersion parameter for poisson family taken to be 1)
##
##     Null deviance: 20817  on 28  degrees of freedom
## Residual deviance: 17104  on 26  degrees of freedom
## AIC: 17400
##
## Number of Fisher Scoring iterations: 4
```

```
#Model selection for number of species, Global
meso.numsp.models <- list(Intercept = meso.numsp.intercept,
                          NumRec = meso.numsp.numrec,
                          ConMar = meso.numsp.marginsum,
                          CurVel = meso.numsp.current,
                          Depth = meso.numsp.depth,
                          HumImp = meso.numsp.humimp,
                          PriPro = meso.numsp.primprod,
                          TemMea = meso.numsp.themmean,
                          Area = meso.numsp.area,
                          Nitrate = meso.numsp.nitrate)
meso.numsp.aic.df <- data.frame(Model = names(meso.numsp.models),
                                AIC = sapply(meso.numsp.models, function(
x) AICc(x)),
                                akaike.weights(sapply(meso.numsp.models,
function(x) AICc(x))))


meso.numsp.aic.df <- meso.numsp.aic.df[order(meso.numsp.aic.df$AIC),]
meso.numsp.aic.df$Cumulative.Weight <- cumsum(meso.numsp.aic.df$weights)

kable(meso.numsp.aic.df, row.names = FALSE)
```

Model	AIC	deltaAIC	rel.LL	weights	Cumulative.Weight
HumImp	16167.67	0.000	1	1	1
TemMea	17187.78	1020.117	0	0	1
Depth	17353.33	1185.662	0	0	1
Nitrate	17400.97	1233.308	0	0	1
Area	18908.72	2741.055	0	0	1
CurVel	19128.79	2961.120	0	0	1
ConMar	19349.79	3182.125	0	0	1
NumRec	21104.27	4936.606	0	0	1
PriPro	21105.08	4937.418	0	0	1
Intercept	21108.50	4940.834	0	0	1

```
#write.csv(meso.numsp.aic.df, file = "global.5.degree.numsp.meso.aic.csv"
)

#Plots for number of species, Global
ggplot(Ecological_Data_Global, aes(x = NumRec_Mes, y = predict(meso.numsp
.numrec, Ecological_Data_Global))) +
  geom_smooth(method = "glm", formula = y ~ x, color = "#1a80bb", fill =
"#85bede") +  # Add a smooth dark blue line with light blue shadow
  geom_point(size = 3) +  # Add scatter plot points
  theme_bw() +  # Use the black and white theme
  scale_x_continuous(labels = scales::scientific) +
  labs(
    x = "Number of Records",  # Shorten the x-axis title
    y = "Predicted Value"  # Shorten the y-axis title
  ) +
  theme(
    panel.grid.minor = element_blank(),
    panel.grid.major = element_blank(),
    axis.text.x = element_text(size =20),  # Increase x-axis text size
    axis.text.y = element_text(size = 20),  # Increase y-axis text size
    axis.title.x = element_text(size = 22), # Increase x-axis title size
    axis.title.y = element_text(size = 22)  # Increase y-axis title size
  )
```

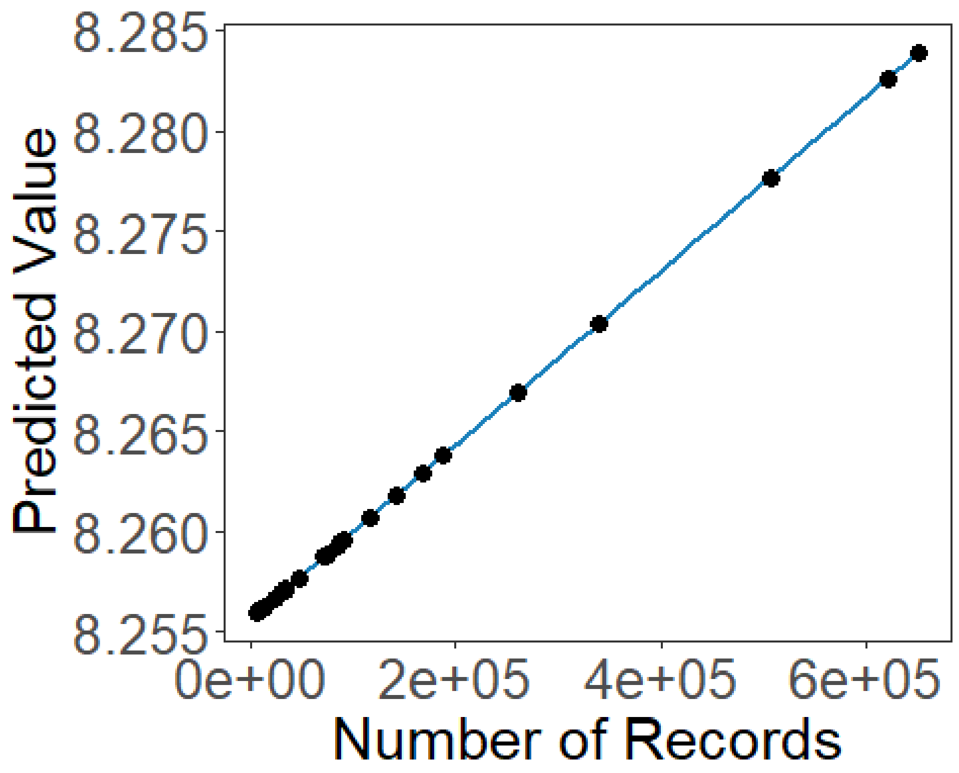


```
ggplot(Ecological_Data_Global, aes(x = Margin_Sum, y = predict(meso.numsp
.marginsum, Ecological_Data_Global))) +
  geom_smooth(method = "glm", formula = y ~ x, color = "#1a80bb", fill =
"#85bede") +  # Add a smooth dark blue line with light blue shadow
  geom_point(size = 3) +  # Add scatter plot points
  theme_bw() +  # Use the black and white theme
  scale_x_continuous(labels = scales::scientific) +
  labs(
    x = "Continental Margin (km2)",  # Shorten the x-axis title
    y = "Predicted Value"  # Shorten the y-axis title
  ) +
  theme(
    panel.grid.minor = element_blank(),
    panel.grid.major = element_blank(),
    axis.text.x = element_text(size =20),  # Increase x-axis text size
    axis.text.y = element_text(size = 20),  # Increase y-axis text size
    axis.title.x = element_text(size = 22), # Increase x-axis title size
    axis.title.y = element_text(size = 22)  # Increase y-axis title size
  )
```

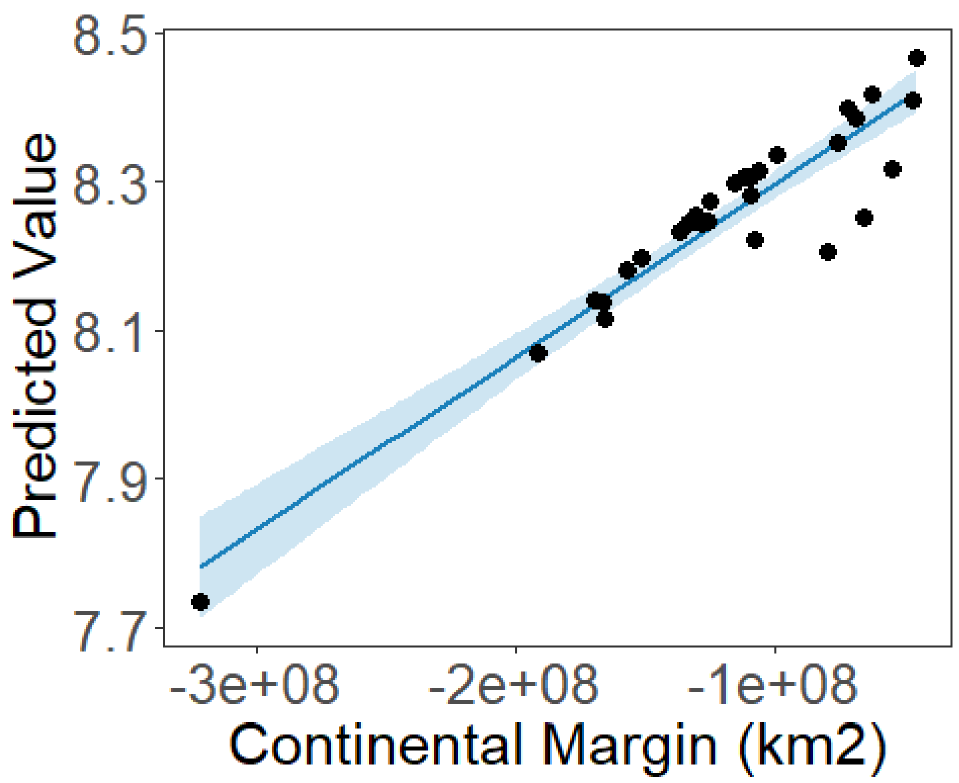


```
ggplot(Ecological_Data_Global, aes(x = CurVel_Mean, y = predict(meso.nums
p.marginsum, Ecological_Data_Global))) +
  geom_smooth(method = "glm", formula = y ~ x, color = "#1a80bb", fill =
"#85bede") +  # Add a smooth dark blue line with light blue shadow
  geom_point(size = 3) +  # Add scatter plot points
  theme_bw() +  # Use the black and white theme
  labs(
    x = "Current Velocity (m.s-1)",  # Shorten the x-axis title
    y = "Predicted Value"  # Shorten the y-axis title
  ) +
  theme(
    panel.grid.minor = element_blank(),
    panel.grid.major = element_blank(),
    axis.text.x = element_text(size =20),  # Increase x-axis text size
    axis.text.y = element_text(size = 20),  # Increase y-axis text size
    axis.title.x = element_text(size = 22), # Increase x-axis title size
    axis.title.y = element_text(size = 22)  # Increase y-axis title size
  )
```

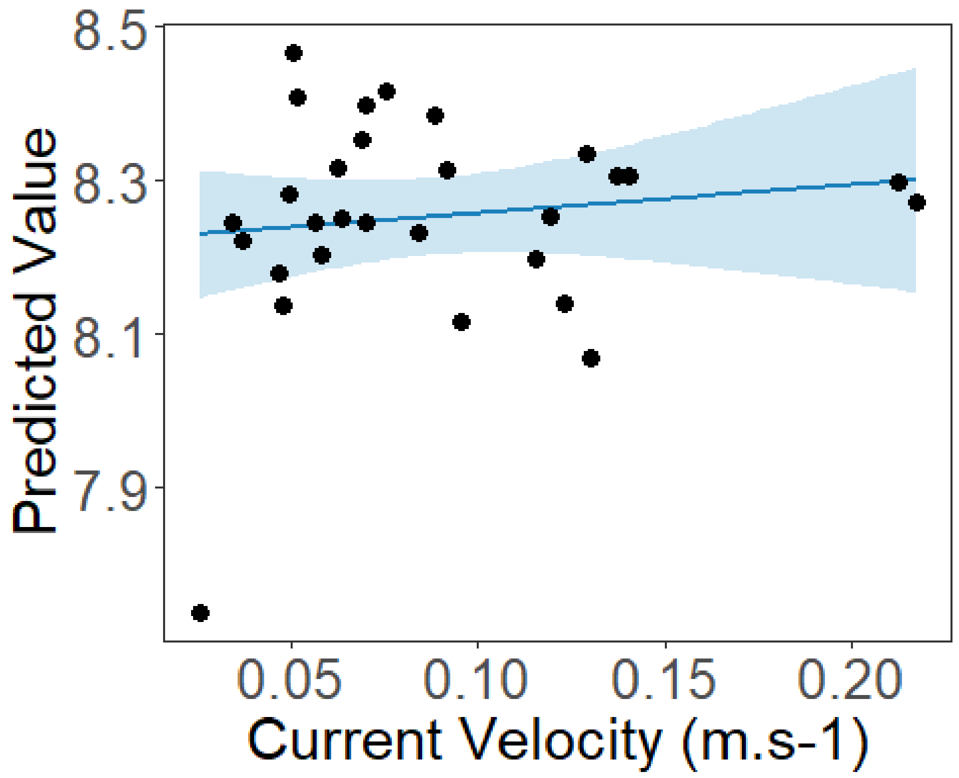


```r
ggplot(Ecological_Data_Global, aes(x = Depth_Mean, y = predict(meso.numsp
.depth, Ecological_Data_Global))) +
  geom_point(size = 3) +  # Add scatter plot points
  geom_smooth(method = "glm", formula = y ~ x, color = "#1a80bb", fill =
"#85bede") +  # Add a smooth dark blue line with light blue shadow
  theme_bw() +  # Use the black and white theme
  scale_x_continuous(labels = scales::scientific) +
  labs(
    x = "Depth (m)",  # Shorten the x-axis title
    y = "Predicted Value"  # Shorten the y-axis title
  ) +
  theme(
    panel.grid.minor = element_blank(),
    panel.grid.major = element_blank(),
    axis.text.x = element_text(size =20),  # Increase x-axis text size
    axis.text.y = element_text(size = 20),  # Increase y-axis text size
    axis.title.x = element_text(size = 22), # Increase x-axis title size
    axis.title.y = element_text(size = 22)  # Increase y-axis title size
  )
```

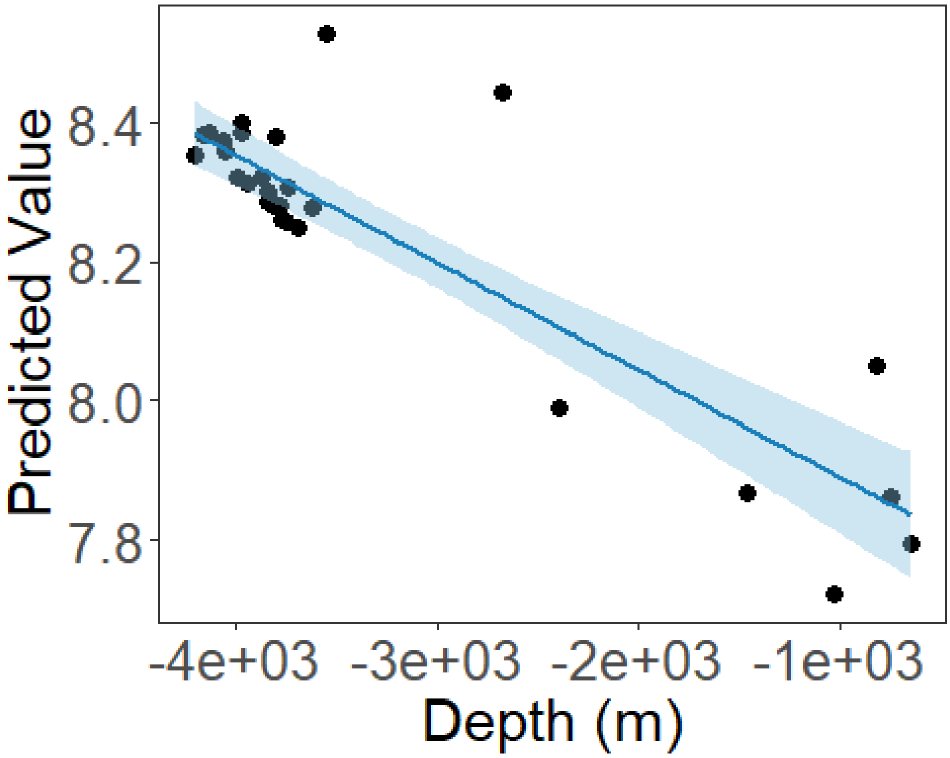


```r
ggplot(Ecological_Data_Global, aes(x = HumImp_Mean, y = predict(meso.nums
p.humimp, Ecological_Data_Global))) +
  geom_smooth(method = "glm", formula = y ~ x, color = "#1a80bb", fill =
"#85bede") +  # Add a smooth dark blue line with light blue shadow
  geom_point(size = 3) +  # Add scatter plot points
  theme_bw() +  # Use the black and white theme
  labs(
    x = "Human Impact",  # Shorten the x-axis title
    y = "Predicted Value"  # Shorten the y-axis title
  ) +
  theme(
    panel.grid.minor = element_blank(),
    panel.grid.major = element_blank(),
    axis.text.x = element_text(size =20),  # Increase x-axis text size
    axis.text.y = element_text(size = 20),  # Increase y-axis text size
    axis.title.x = element_text(size = 22), # Increase x-axis title size
    axis.title.y = element_text(size = 22)  # Increase y-axis title size
  )
```

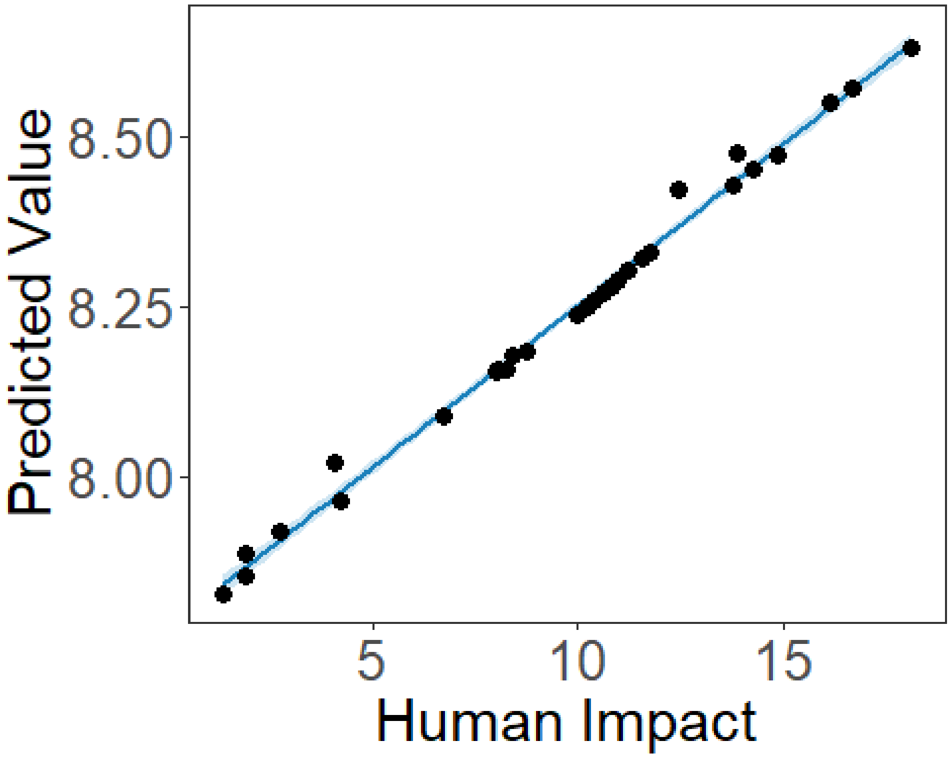


```
ggplot(Ecological_Data_Global, aes(x = PrimProd_Mean, y = predict(meso.nu
msp.primprod, Ecological_Data_Global))) +
  geom_smooth(method = "glm", formula = y ~ x, color = "#1a80bb", fill =
"#85bede") +  # Add a smooth dark blue line with light blue shadow
  geom_point(size = 3) +  # Add scatter plot points
  theme_bw() +  # Use the black and white theme
  labs(
    x = "Primary Productivity (mmol . m-3)",  # Shorten the x-axis title
    y = "Predicted Value"  # Shorten the y-axis title
  ) +
  theme(
    panel.grid.minor = element_blank(),
    panel.grid.major = element_blank(),
    axis.text.x = element_text(size =20),  # Increase x-axis text size
    axis.text.y = element_text(size = 20),  # Increase y-axis text size
    axis.title.x = element_text(size = 22), # Increase x-axis title size
    axis.title.y = element_text(size = 22)  # Increase y-axis title size
  )
```

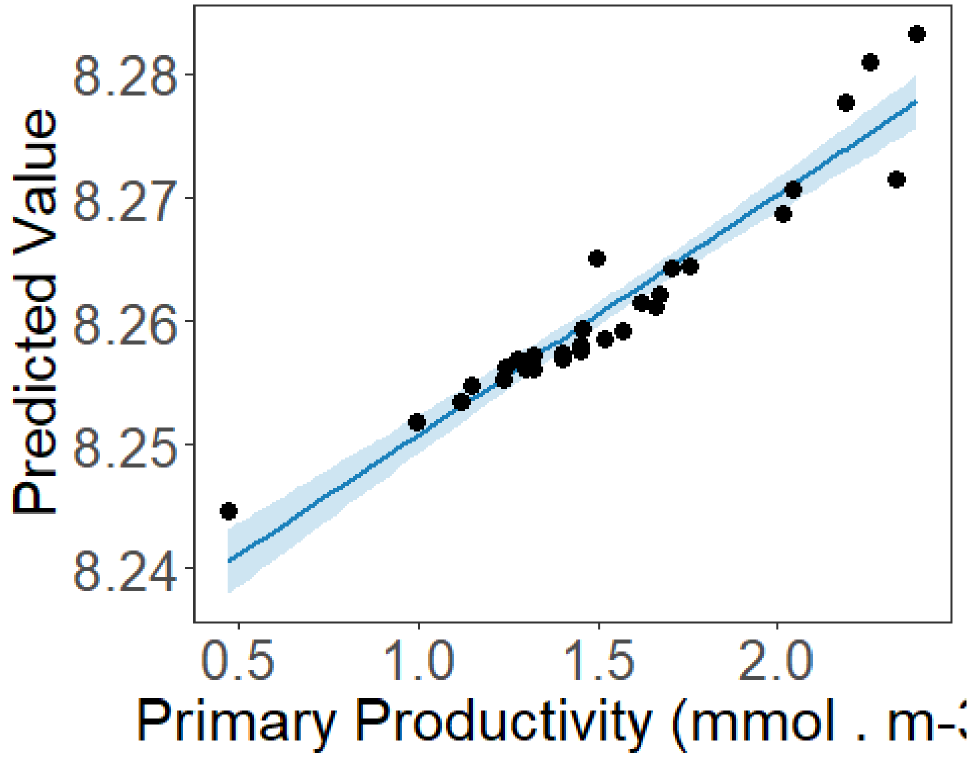


```
ggplot(Ecological_Data_Global, aes(x = ThemM_Mean, y = predict(meso.numsp
.themmean, Ecological_Data_Global))) +
  geom_smooth(method = "glm", formula = y ~ x, color = "#1a80bb", fill =
"#85bede") +  # Add a smooth dark blue line with light blue shadow
  geom_point(size = 3) +  # Add scatter plot points
  theme_bw() +  # Use the black and white theme
  labs(
    x = "Temperature Mean (ºC)",  # Shorten the x-axis title
    y = "Predicted Value"  # Shorten the y-axis title
  ) +
  theme(
    panel.grid.minor = element_blank(),
    panel.grid.major = element_blank(),
    axis.text.x = element_text(size =20),  # Increase x-axis text size
    axis.text.y = element_text(size = 20),  # Increase y-axis text size
    axis.title.x = element_text(size = 22), # Increase x-axis title size
    axis.title.y = element_text(size = 22)  # Increase y-axis title size
  )
```

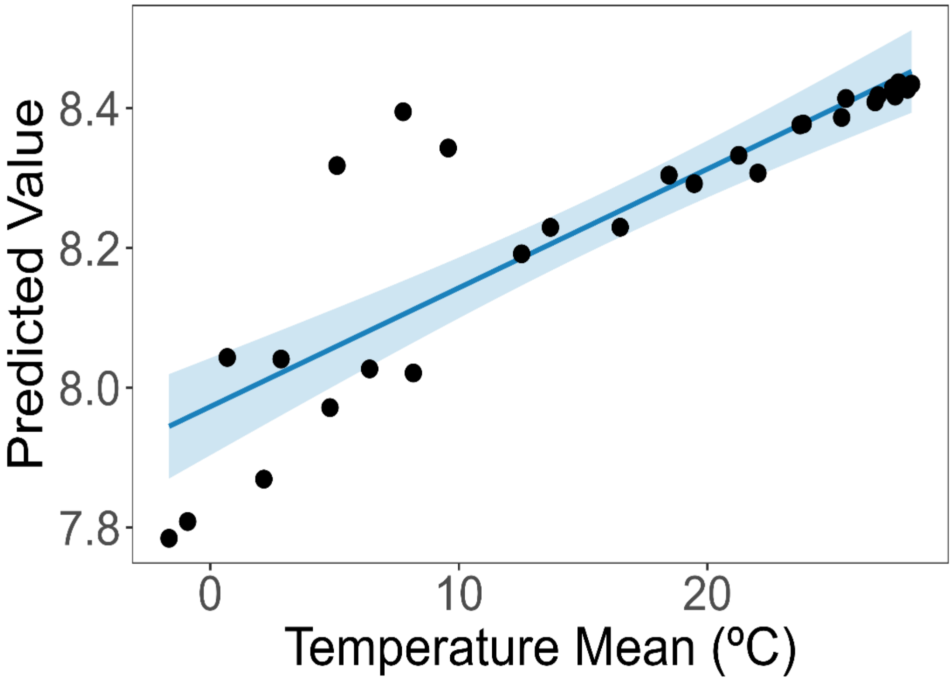


```r
ggplot(Ecological_Data_Global, aes(x = Area_Sum, y = predict(meso.numsp.a
rea, Ecological_Data_Global))) +
  geom_smooth(method = "glm", formula = y ~ x, color = "#1a80bb", fill =
"#85bede") +  # Add a smooth dark blue line with light blue shadow
  geom_point(size = 3) +  # Add scatter plot points
  theme_bw() +  # Use the black and white theme
  scale_x_continuous(labels = scales::scientific) +
  labs(
    x = "Area (km2)",  # Shorten the x-axis title
    y = "Predicted Value"  # Shorten the y-axis title
  ) +
  theme(
    panel.grid.minor = element_blank(),
    panel.grid.major = element_blank(),
    axis.text.x = element_text(size =20),  # Increase x-axis text size
    axis.text.y = element_text(size = 20),  # Increase y-axis text size
    axis.title.x = element_text(size = 22), # Increase x-axis title size
    axis.title.y = element_text(size = 22)  # Increase y-axis title size
  )
```

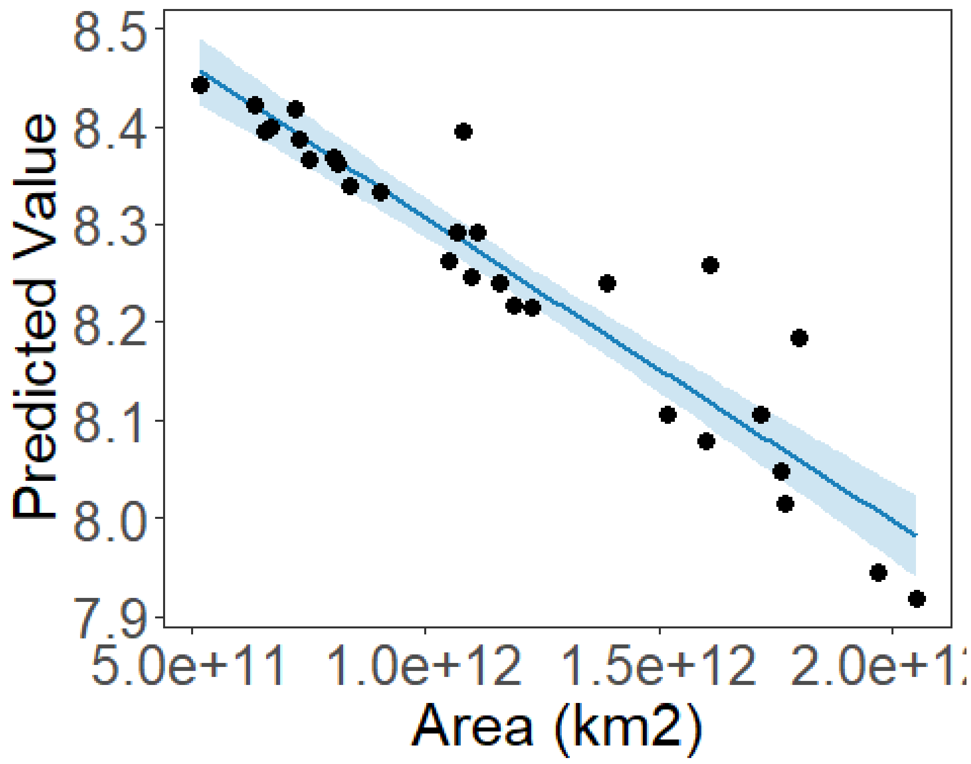


```
ggplot(Ecological_Data_Global, aes(x = Nitrate_Mean, y = predict(meso.num
sp.nitrate, Ecological_Data_Global))) +
  geom_smooth(method = "glm", formula = y ~ x, color = "#1a80bb", fill =
"#85bede") +  # Add a smooth dark blue line with light blue shadow
  geom_point(size = 3) +  # Add scatter plot points
  theme_bw() +  # Use the black and white theme
  labs(
    x = "Nitrate (mmol . m-3)",  # Shorten the x-axis title
    y = "Predicted Value"  # Shorten the y-axis title
  ) +
  theme(
    panel.grid.minor = element_blank(),
    panel.grid.major = element_blank(),
    axis.text.x = element_text(size =20),  # Increase x-axis text size
    axis.text.y = element_text(size = 20),  # Increase y-axis text size
    axis.title.x = element_text(size = 22), # Increase x-axis title size
    axis.title.y = element_text(size = 22)  # Increase y-axis title size
  )
```

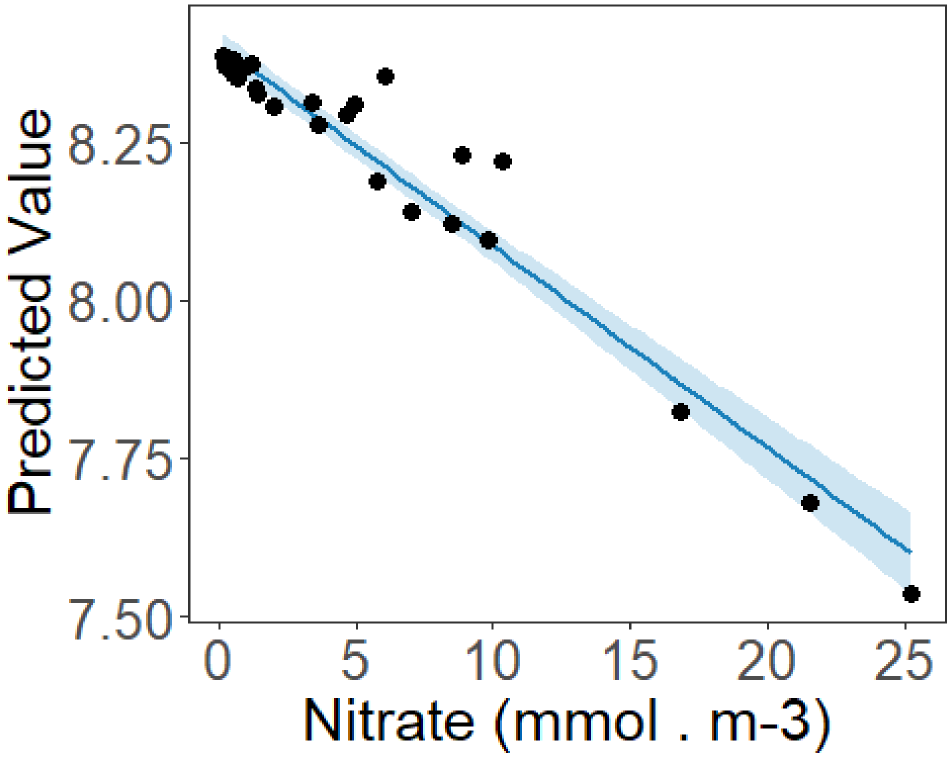


```
# ===================================
#                MESO-ES50-GLM
# ===================================

meso.es50.intercept <- glm(ES50_Mes ~ 1, family = "poisson", data = Ecolo
gical_Data_Global)
summary(meso.es50.intercept)

##
## Call:
## glm(formula = ES50_Mes ~ 1, family = "poisson", data = Ecological_Data
_Global)
##
## Coefficients:
##             Estimate Std. Error z value Pr(>|z|)
## (Intercept)  3.74585    0.02854   131.3   <2e-16 ***
## ---
## Signif. codes:  0 '***' 0.001 '**' 0.01 '*' 0.05 '.' 0.1 ' ' 1
##
## (Dispersion parameter for poisson family taken to be 1)
##
##     Null deviance: 11.713  on 28  degrees of freedom
## Residual deviance: 11.713  on 28  degrees of freedom
## AIC: 175.62
##
## Number of Fisher Scoring iterations: 4

meso.es50.numrec <- glm(ES50_Mes ~ NumRec_Mes, family = "poisson", data =
Ecological_Data_Global)
summary(meso.es50.numrec)
```

```
##
## Call:
## glm(formula = ES50_Mes ~ NumRec_Mes, family = "poisson", data = Ecolog
ical_Data_Global)
##
## Coefficients:
##                 Estimate Std. Error z value Pr(>|z|)
## (Intercept)  3.767e+00  3.573e-02 105.444   <2e-16 ***
## NumRec_Mes  -1.635e-07  1.677e-07  -0.975     0.33
## ---
## Signif. codes:  0 '***' 0.001 '**' 0.01 '*' 0.05 '.' 0.1 ' ' 1
##
## (Dispersion parameter for poisson family taken to be 1)
##
##     Null deviance: 11.713  on 28  degrees of freedom
## Residual deviance: 10.745  on 27  degrees of freedom
## AIC: 176.65
##
## Number of Fisher Scoring iterations: 4
```

```
meso.es50.marginsum <- glm(ES50_Mes ~ Margin_Sum, family = "poisson", dat
a = Ecological_Data_Global)
summary(meso.es50.marginsum)
```

```
##
## Call:
## glm(formula = ES50_Mes ~ Margin_Sum, family = "poisson", data = Ecolog
ical_Data_Global)
##
## Coefficients:
##                 Estimate Std. Error z value Pr(>|z|)
## (Intercept)  3.745e+00  6.795e-02  55.109   <2e-16 ***
## Margin_Sum  -1.105e-11  5.213e-10  -0.021    0.983
## ---
## Signif. codes:  0 '***' 0.001 '**' 0.01 '*' 0.05 '.' 0.1 ' ' 1
##
## (Dispersion parameter for poisson family taken to be 1)
##
##     Null deviance: 11.713  on 28  degrees of freedom
## Residual deviance: 11.713  on 27  degrees of freedom
## AIC: 177.62
##
## Number of Fisher Scoring iterations: 4
```

```
meso.es50.current <- glm(ES50_Mes ~ CurVel_Mean, family = "poisson", data
= Ecological_Data_Global)
summary(meso.es50.current)
```

```
##
## Call:
## glm(formula = ES50_Mes ~ CurVel_Mean, family = "poisson", data = Ecolo
gical_Data_Global)
```

```
##
## Coefficients:
##             Estimate Std. Error z value Pr(>|z|)
## (Intercept)  3.68562    0.05999  61.441   <2e-16 ***
## CurVel_Mean  0.67933    0.58998   1.151     0.25
## ---
## Signif. codes:  0 '***' 0.001 '**' 0.01 '*' 0.05 '.' 0.1 ' ' 1
##
## (Dispersion parameter for poisson family taken to be 1)
##
##     Null deviance: 11.713  on 28  degrees of freedom
## Residual deviance: 10.404  on 27  degrees of freedom
## AIC: 176.31
##
## Number of Fisher Scoring iterations: 4
```

```
meso.es50.depth <- glm(ES50_Mes ~ Depth_Mean, family = "poisson", data =
Ecological_Data_Global)
summary(meso.es50.depth)
```

```
##
## Call:
## glm(formula = ES50_Mes ~ Depth_Mean, family = "poisson", data = Ecolog
ical_Data_Global)
##
## Coefficients:
##               Estimate Std. Error z value Pr(>|z|)
## (Intercept)  3.744e+00  8.728e-02  42.892   <2e-16 ***
## Depth_Mean  -7.121e-07  2.513e-05  -0.028    0.977
## ---
## Signif. codes:  0 '***' 0.001 '**' 0.01 '*' 0.05 '.' 0.1 ' ' 1
##
## (Dispersion parameter for poisson family taken to be 1)
##
##     Null deviance: 11.713  on 28  degrees of freedom
## Residual deviance: 11.712  on 27  degrees of freedom
## AIC: 177.62
##
## Number of Fisher Scoring iterations: 4
```

```
meso.es50.humimp <- glm(ES50_Mes ~ HumImp_Mean, family = "poisson", data
= Ecological_Data_Global)
summary(meso.es50.humimp)
```

```
##
## Call:
## glm(formula = ES50_Mes ~ HumImp_Mean, family = "poisson", data = Ecolo
gical_Data_Global)
##
## Coefficients:
##              Estimate Std. Error z value Pr(>|z|)
## (Intercept) 3.7421328  0.0680306   55.01   <2e-16 ***
```

```
## HumImp_Mean 0.0003817  0.0063451    0.06    0.952
## ---
## Signif. codes:  0 '***' 0.001 '**' 0.01 '*' 0.05 '.' 0.1 ' ' 1
##
## (Dispersion parameter for poisson family taken to be 1)
##
##     Null deviance: 11.713  on 28  degrees of freedom
## Residual deviance: 11.710  on 27  degrees of freedom
## AIC: 177.61
##
## Number of Fisher Scoring iterations: 4
```

```
meso.es50.primprod <- glm(ES50_Mes ~ PrimProd_Mean, family = "poisson", d
ata = Ecological_Data_Global)
summary(meso.es50.primprod)
```

```
##
## Call:
## glm(formula = ES50_Mes ~ PrimProd_Mean, family = "poisson", data = Eco
logical_Data_Global)
##
## Coefficients:
##                Estimate Std. Error z value Pr(>|z|)
## (Intercept)     3.84155    0.10835  35.455   <2e-16 ***
## PrimProd_Mean -0.06215    0.06812  -0.912    0.362
## ---
## Signif. codes:  0 '***' 0.001 '**' 0.01 '*' 0.05 '.' 0.1 ' ' 1
##
## (Dispersion parameter for poisson family taken to be 1)
##
##     Null deviance: 11.713  on 28  degrees of freedom
## Residual deviance: 10.880  on 27  degrees of freedom
## AIC: 176.78
##
## Number of Fisher Scoring iterations: 4
```

```
meso.es50.themmean <- glm(ES50_Mes ~ ThemM_Mean, family = "poisson", data
= Ecological_Data_Global)
summary(meso.es50.themmean)
```

```
##
## Call:
## glm(formula = ES50_Mes ~ ThemM_Mean, family = "poisson", data = Ecolog
ical_Data_Global)
##
## Coefficients:
##             Estimate Std. Error z value Pr(>|z|)
## (Intercept) 3.669449   0.054115  67.808   <2e-16 ***
## ThemM_Mean  0.004743   0.002812   1.687   0.0916 .
## ---
## Signif. codes:  0 '***' 0.001 '**' 0.01 '*' 0.05 '.' 0.1 ' ' 1
##
```

```
## (Dispersion parameter for poisson family taken to be 1)
##
##     Null deviance: 11.7132  on 28  degrees of freedom
## Residual deviance:  8.8521  on 27  degrees of freedom
## AIC: 174.76
##
## Number of Fisher Scoring iterations: 3
```

```
meso.es50.area <- glm(ES50_Mes ~ Area_Sum, family = "poisson", data = Eco
logical_Data_Global)
summary(meso.es50.area)
```

```
##
## Call:
## glm(formula = ES50_Mes ~ Area_Sum, family = "poisson", data = Ecologic
al_Data_Global)
##
## Coefficients:
##              Estimate Std. Error z value Pr(>|z|)
## (Intercept) 3.670e+00  8.178e-02  44.877   <2e-16 ***
## Area_Sum    6.390e-14  6.427e-14   0.994     0.32
## ---
## Signif. codes:  0 '***' 0.001 '**' 0.01 '*' 0.05 '.' 0.1 ' ' 1
##
## (Dispersion parameter for poisson family taken to be 1)
##
##     Null deviance: 11.713  on 28  degrees of freedom
## Residual deviance: 10.728  on 27  degrees of freedom
## AIC: 176.63
##
## Number of Fisher Scoring iterations: 4
```

```
meso.es50.nitrate <- glm(ES50_Mes ~ Nitrate_Mean, family = "poisson", dat
a = Ecological_Data_Global)
summary(meso.es50.nitrate)
```

```
##
## Call:
## glm(formula = ES50_Mes ~ Nitrate_Mean, family = "poisson", data = Ecol
ogical_Data_Global)
##
## Coefficients:
##               Estimate Std. Error z value Pr(>|z|)
## (Intercept)   3.785694   0.036083 104.917   <2e-16 ***
## Nitrate_Mean -0.008112   0.004645  -1.747   0.0807 .
## ---
## Signif. codes:  0 '***' 0.001 '**' 0.01 '*' 0.05 '.' 0.1 ' ' 1
##
## (Dispersion parameter for poisson family taken to be 1)
##
##     Null deviance: 11.7132  on 28  degrees of freedom
## Residual deviance:  8.5673  on 27  degrees of freedom
```

```
## AIC: 174.47
##
## Number of Fisher Scoring iterations: 3

#Model selection for number of species, Global
meso.es50.models <- list(Intercept = meso.es50.intercept,
                         NumRec = meso.es50.numrec,
                         ConMar = meso.es50.marginsum,
                         CurVel = meso.es50.current,
                         Depth = meso.es50.depth,
                         HumIpm = meso.es50.humimp,
                         PriPro = meso.es50.primprod,
                         TemMea = meso.es50.themmean,
                         Area = meso.es50.area,
                         Nitrate = meso.es50.nitrate)
meso.es50.aic.df <- data.frame(Model = names(meso.es50.models),
                               AIC = sapply(meso.es50.models, function(x)
AICc(x)),
                               akaike.weights(sapply(meso.es50.models, fu
nction(x) AICc(x))))


meso.es50.aic.df <-meso.es50.aic.df[order(meso.es50.aic.df$AIC),]
meso.es50.aic.df$Cumulative.Weight <- cumsum(meso.es50.aic.df$weights)

kable(meso.es50.aic.df, row.names = FALSE)
```

Model	AIC	deltaAIC	rel.LL	weights	Cumulative.Weight
Nitrate	174.9318	0.0000000	1.0000000	0.2202876	0.2202876
TemMea	175.2166	0.2847725	0.8672862	0.1910524	0.4113400
Intercept	175.7642	0.8324181	0.6595424	0.1452890	0.5566290
CurVel	176.7684	1.8365708	0.3992029	0.0879395	0.6445685
Area	177.0926	2.1607756	0.3394639	0.0747797	0.7193482
NumRec	177.1099	2.1780447	0.3365454	0.0741368	0.7934849
PriPro	177.2444	2.3126015	0.3146480	0.0693131	0.8627980
HumIpm	178.0740	3.1421893	0.2078176	0.0457796	0.9085776
Depth	178.0768	3.1450050	0.2075252	0.0457152	0.9542929
ConMar	178.0772	3.1453591	0.2074885	0.0457071	1.0000000

```
#write.csv(meso.es50.aic.df, file = "global.5.degree.es50.meso.aic.csv")

#Plots for number of species, Global
ggplot(Ecological_Data_Global, aes(x = NumRec_Mes, y = ES50_Mes)) +
  geom_smooth(method = "glm", formula = y ~ x, color = "#1a80bb", fill =
"#85bede") +  # Add a smooth dark blue line with light blue shadow
  geom_point(size = 3) +  # Add scatter plot points
  theme_bw() +  # Use the black and white theme
  scale_x_continuous(labels = scales::scientific) +
  scale_y_continuous(limits=c(29, 52), breaks=seq(30, 50, by=5), expand =
```

```
c(0, 0)) +
  labs(
    x = "Number of Records",  # Shorten the x-axis title
    y = "ES50"  # Shorten the y-axis title
  ) +
  theme(
    panel.grid.minor = element_blank(),
    panel.grid.major = element_blank(),
    axis.text.x = element_text(size =20),  # Increase x-axis text size
    axis.text.y = element_text(size = 20),  # Increase y-axis text size
    axis.title.x = element_text(size = 22), # Increase x-axis title size
    axis.title.y = element_text(size = 22)  # Increase y-axis title size
  )
```

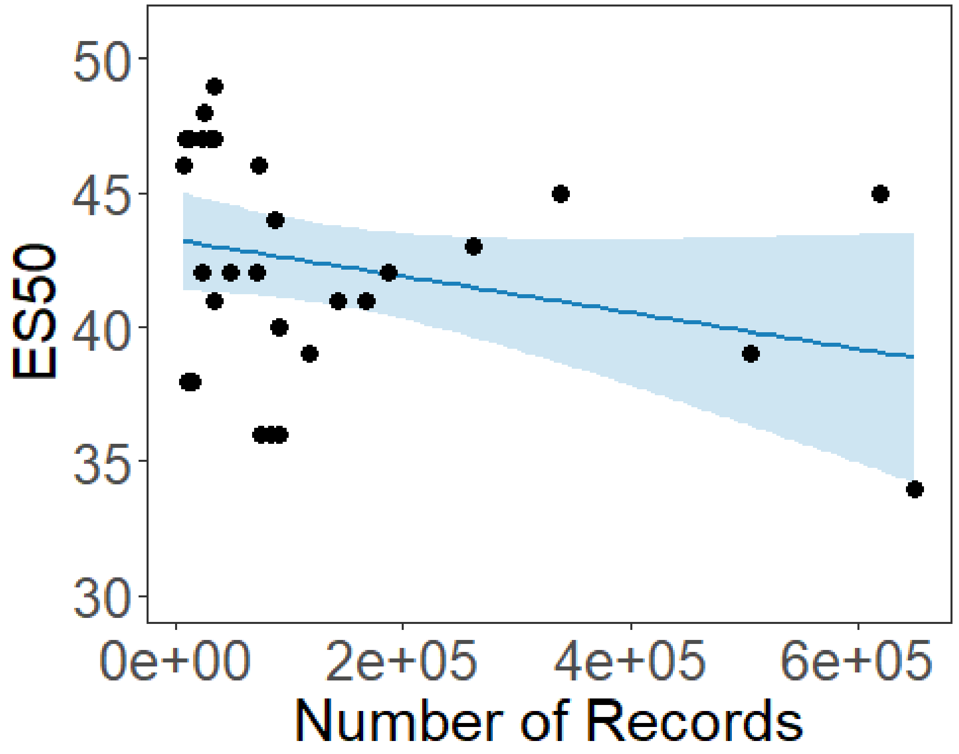


```
ggplot(Ecological_Data_Global, aes(x = Margin_Sum, y = ES50_Mes)) +
  geom_smooth(method = "glm", formula = y ~ x, color = "#1a80bb", fill =
"#85bede") +  # Add a smooth dark blue line with light blue shadow
  geom_point(size = 3) +  # Add scatter plot points
  theme_bw() +  # Use the black and white theme
  scale_x_continuous(labels = scales::scientific) +
  scale_y_continuous(limits=c(29, 52), breaks=seq(30, 50, by=5), expand =
c(0, 0)) +
  labs(
    x = "Continental Margin (km2)",  # Shorten the x-axis title
    y = "ES50"  # Shorten the y-axis title
  ) +
  theme(
    panel.grid.minor = element_blank(),
    panel.grid.major = element_blank(),
    axis.text.x = element_text(size =20),  # Increase x-axis text size
```

```
    axis.text.y = element_text(size = 20),  # Increase y-axis text size
    axis.title.x = element_text(size = 22), # Increase x-axis title size
    axis.title.y = element_text(size = 22)  # Increase y-axis title size
  )
```

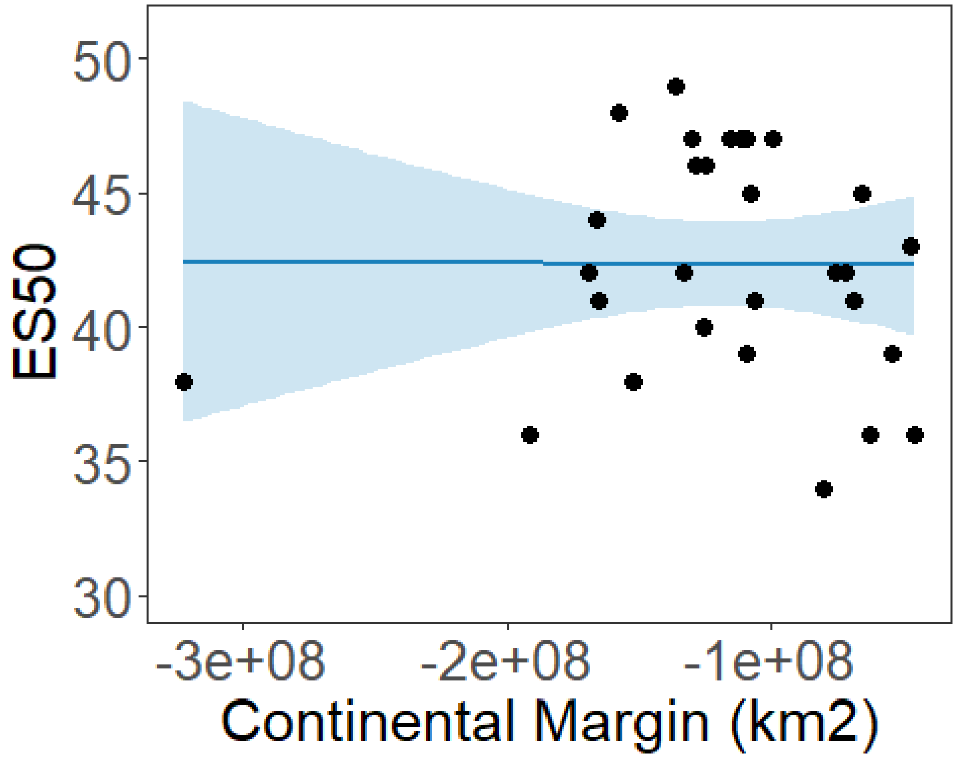


```
ggplot(Ecological_Data_Global, aes(x = CurVel_Mean, y = ES50_Mes)) +
  geom_smooth(method = "glm", formula = y ~ x, color = "#1a80bb", fill =
"#85bede") +  # Add a smooth dark blue line with light blue shadow
  geom_point(size = 3) +  # Add scatter plot points
  theme_bw() +  # Use the black and white theme
  scale_y_continuous(limits=c(29, 52), breaks=seq(30, 50, by=5), expand =
c(0, 0)) +
  labs(
    x = "Current Velocity (m.s-1)",  # Shorten the x-axis title
    y = "ES50"  # Shorten the y-axis title
  ) +
  theme(
    panel.grid.minor = element_blank(),
    panel.grid.major = element_blank(),
    axis.text.x = element_text(size =20),  # Increase x-axis text size
    axis.text.y = element_text(size = 20),  # Increase y-axis text size
    axis.title.x = element_text(size = 22), # Increase x-axis title size
    axis.title.y = element_text(size = 22)  # Increase y-axis title size
  )
```

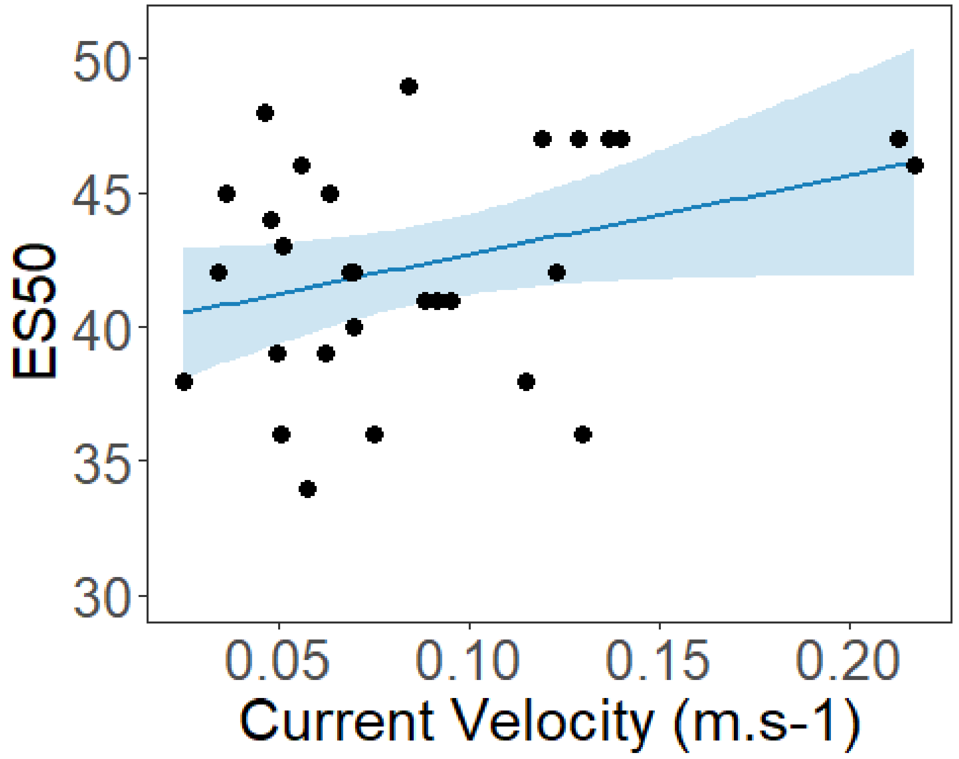


```
ggplot(Ecological_Data_Global, aes(x = Depth_Mean, y = ES50_Mes)) +
  geom_smooth(method = "glm", formula = y ~ x, color = "#1a80bb", fill =
"#85bede") +  # Add a smooth dark blue line with light blue shadow
  geom_point(size = 3) +  # Add scatter plot points
  theme_bw() +  # Use the black and white theme
  scale_x_continuous(labels = scales::scientific) +
  scale_y_continuous(limits=c(29, 52), breaks=seq(30, 50, by=5), expand =
c(0, 0)) +
  labs(
    x = "Depth (m)",  # Shorten the x-axis title
    y = "ES50"  # Shorten the y-axis title
  ) +
  theme(
    panel.grid.minor = element_blank(),
    panel.grid.major = element_blank(),
    axis.text.x = element_text(size =20),  # Increase x-axis text size
    axis.text.y = element_text(size = 20),  # Increase y-axis text size
    axis.title.x = element_text(size = 22), # Increase x-axis title size
    axis.title.y = element_text(size = 22)  # Increase y-axis title size
  )
```

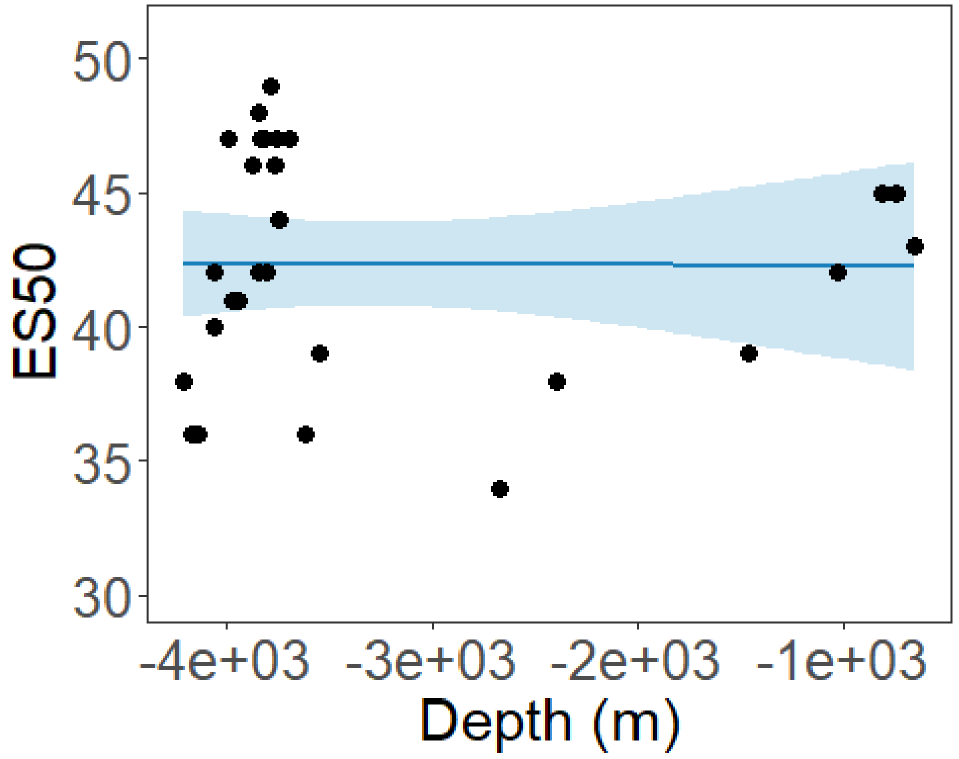


```
ggplot(Ecological_Data_Global, aes(x = HumImp_Mean, y = ES50_Mes)) +
  geom_smooth(method = "glm", formula = y ~ x, color = "#1a80bb", fill =
"#85bede") +  # Add a smooth dark blue line with light blue shadow
  geom_point(size = 3) +  # Add scatter plot points
  theme_bw() +  # Use the black and white theme
  scale_y_continuous(limits=c(29, 52), breaks=seq(30, 50, by=5), expand =
c(0, 0)) +
  labs(
    x = "Human Impact",  # Shorten the x-axis title
    y = "ES50"  # Shorten the y-axis title
  ) +
  theme(
    panel.grid.minor = element_blank(),
    panel.grid.major = element_blank(),
    axis.text.x = element_text(size =20),  # Increase x-axis text size
    axis.text.y = element_text(size = 20),  # Increase y-axis text size
    axis.title.x = element_text(size = 22), # Increase x-axis title size
    axis.title.y = element_text(size = 22)  # Increase y-axis title size
  )
```

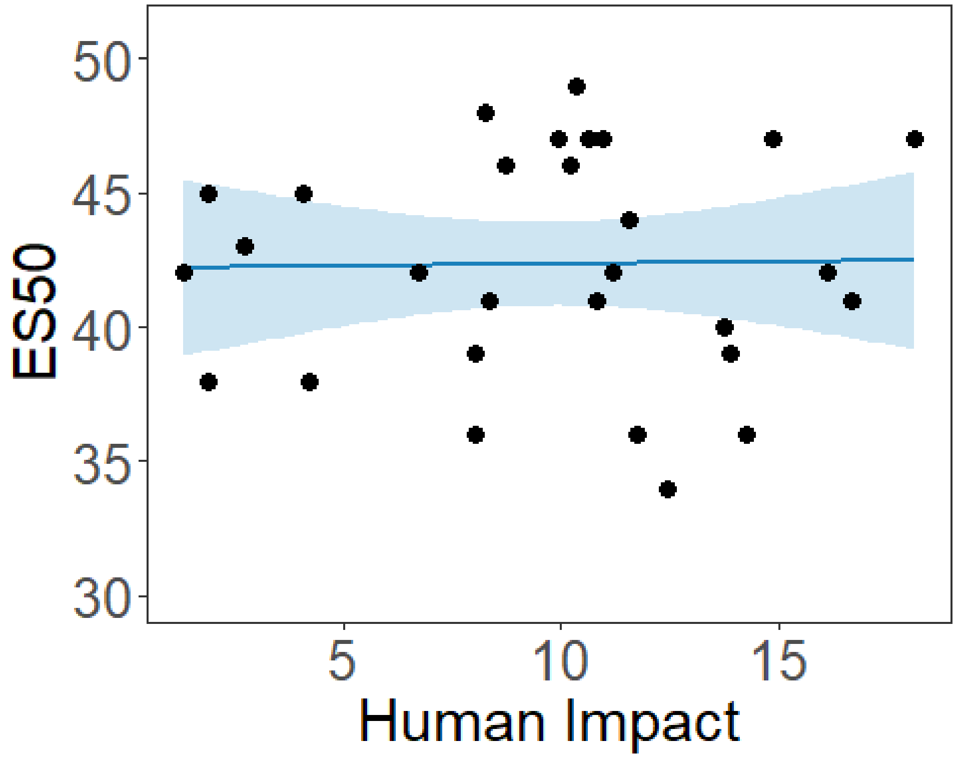


```r
ggplot(Ecological_Data_Global, aes(x = PrimProd_Mean, y = ES50_Mes)) +
  geom_smooth(method = "glm", formula = y ~ x, color = "#1a80bb", fill =
"#85bede") +  # Add a smooth dark blue line with light blue shadow
  geom_point(size = 3) +  # Add scatter plot points
  theme_bw() +  # Use the black and white theme
  scale_y_continuous(limits=c(29, 52), breaks=seq(30, 50, by=5), expand =
c(0, 0)) +
  labs(
    x = "Primary Productivity (mmol . m-3)",  # Shorten the x-axis title
    y = "ES50"  # Shorten the y-axis title
  ) +
  theme(
    panel.grid.minor = element_blank(),
    panel.grid.major = element_blank(),
    axis.text.x = element_text(size =20),  # Increase x-axis text size
    axis.text.y = element_text(size = 20),  # Increase y-axis text size
    axis.title.x = element_text(size = 22), # Increase x-axis title size
    axis.title.y = element_text(size = 22)  # Increase y-axis title size
  )
```

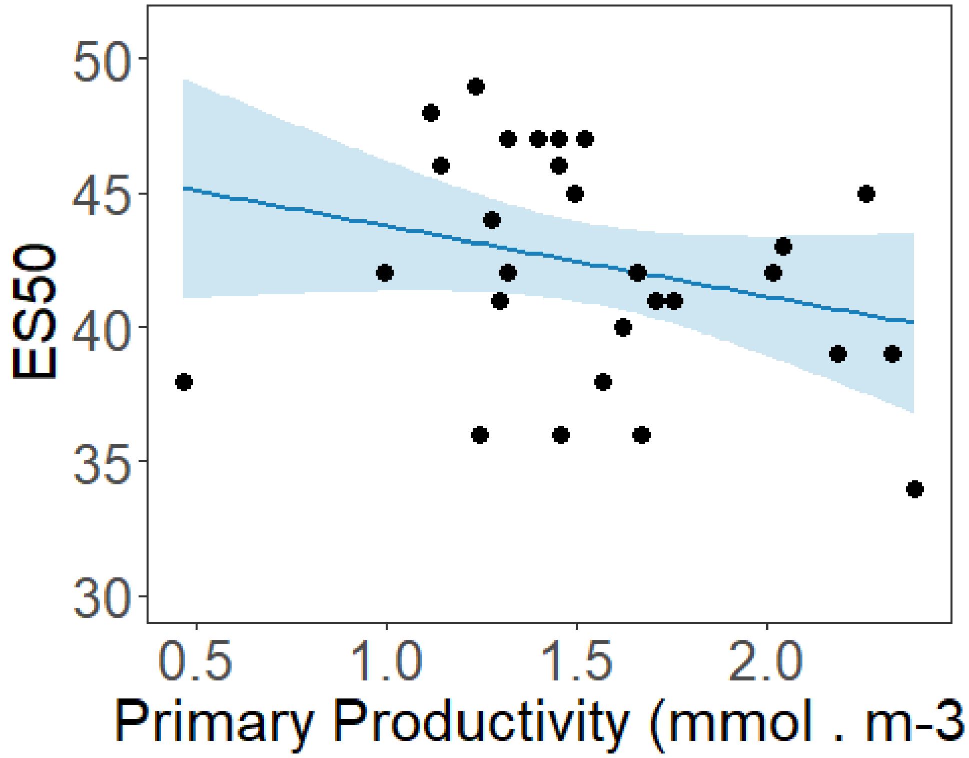


```
ggplot(Ecological_Data_Global, aes(x = ThemM_Mean, y = ES50_Mes)) +
  geom_smooth(method = "glm", formula = y ~ x, color = "#1a80bb", fill =
"#85bede") +  # Add a smooth dark blue line with light blue shadow
  geom_point(size = 3) +  # Add scatter plot points
  theme_bw() +  # Use the black and white theme
  scale_y_continuous(limits=c(29, 52), breaks=seq(30, 50, by=5), expand =
c(0, 0)) +
  labs(
    x = "Temperature Mean (ºC)",  # Shorten the x-axis title
    y = "ES50"  # Shorten the y-axis title
  ) +
  theme(
    panel.grid.minor = element_blank(),
    panel.grid.major = element_blank(),
    axis.text.x = element_text(size =20),  # Increase x-axis text size
    axis.text.y = element_text(size = 20),  # Increase y-axis text size
    axis.title.x = element_text(size = 22), # Increase x-axis title size
    axis.title.y = element_text(size = 22)  # Increase y-axis title size
  )
```

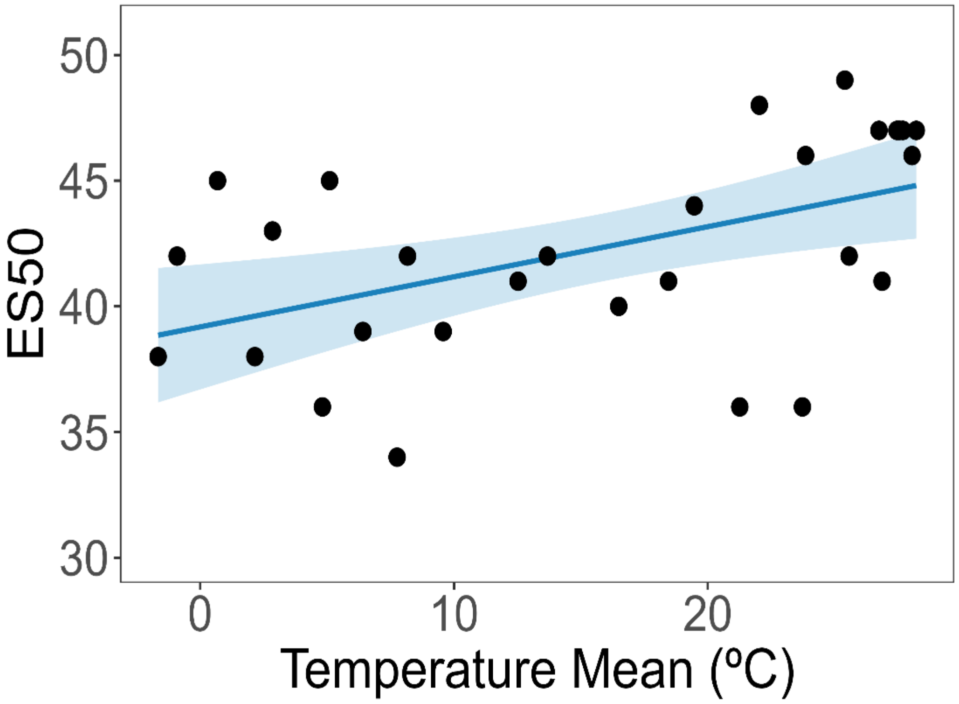


```
ggplot(Ecological_Data_Global, aes(x = Area_Sum, y = ES50_Mes)) +
  geom_smooth(method = "glm", formula = y ~ x, color = "#1a80bb", fill =
"#85bede") +  # Add a smooth dark blue line with light blue shadow
  geom_point(size = 3) +  # Add scatter plot points
  theme_bw() +  # Use the black and white theme
  scale_x_continuous(labels = scales::scientific) +
  scale_y_continuous(limits=c(29, 52), breaks=seq(30, 50, by=5), expand =
c(0, 0)) +
  labs(
    x = "Area (km2)",  # Shorten the x-axis title
    y = "ES50"  # Shorten the y-axis title
  ) +
  theme(
    panel.grid.minor = element_blank(),
    panel.grid.major = element_blank(),
    axis.text.x = element_text(size =20),  # Increase x-axis text size
    axis.text.y = element_text(size = 20),  # Increase y-axis text size
    axis.title.x = element_text(size = 22), # Increase x-axis title size
    axis.title.y = element_text(size = 22)  # Increase y-axis title size
  )
```

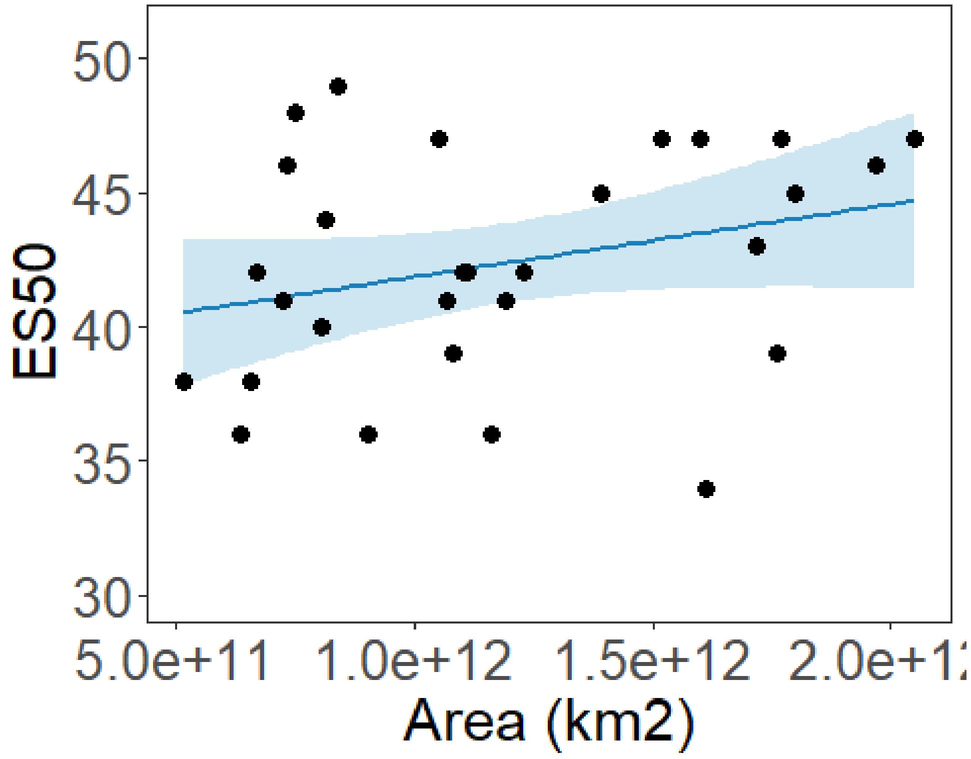


```
ggplot(Ecological_Data_Global, aes(x = Nitrate_Mean, y = ES50_Mes)) +
  geom_smooth(method = "glm", formula = y ~ x, color = "#1a80bb", fill =
"#85bede") +  # Add a smooth dark blue line with light blue shadow
  geom_point(size = 3) +  # Add scatter plot points
  theme_bw() +  # Use the black and white theme
  scale_y_continuous(limits=c(29, 52), breaks=seq(30, 50, by=5), expand =
c(0, 0)) +
  labs(
    x = "Nitrate (mmol . m-3)",  # Shorten the x-axis title
    y = "ES50"  # Shorten the y-axis title
  ) +
  theme(
    panel.grid.minor = element_blank(),
    panel.grid.major = element_blank(),
    axis.text.x = element_text(size =20),  # Increase x-axis text size
    axis.text.y = element_text(size = 20),  # Increase y-axis text size
    axis.title.x = element_text(size = 22), # Increase x-axis title size
    axis.title.y = element_text(size = 22)  # Increase y-axis title size
  )
```

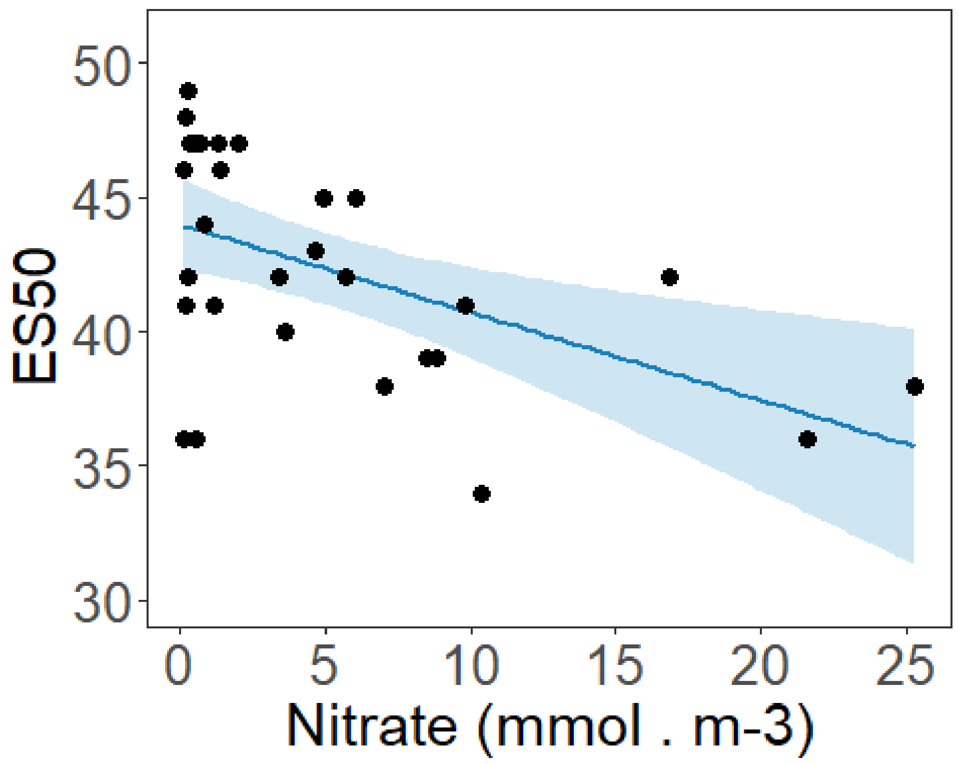

ES50
50
45
40
35
30
0
5
10
15
20
25
Nitrate (mmol . m-3)

Markdown_GLM_Deep.R

hsaeedi

2025-06-07

```
# ===================================
#                DEEP-NUMSPe-GLM
# ===================================
library(readxl)

## Warning: package 'readxl' was built under R version 4.3.3

library(openxlsx)

## Warning: package 'openxlsx' was built under R version 4.3.3

library(tidyverse)

## Warning: package 'tidyverse' was built under R version 4.3.3

## Warning: package 'ggplot2' was built under R version 4.3.3

## Warning: package 'tibble' was built under R version 4.3.3

## Warning: package 'tidyr' was built under R version 4.3.3

## Warning: package 'readr' was built under R version 4.3.3

## Warning: package 'purrr' was built under R version 4.3.3

## Warning: package 'dplyr' was built under R version 4.3.3

## Warning: package 'stringr' was built under R version 4.3.3

## Warning: package 'forcats' was built under R version 4.3.3

## Warning: package 'lubridate' was built under R version 4.3.3

## ── Attaching core tidyverse packages ──────────────────────── tidyvers
e 2.0.0 ──
## ✔ dplyr     1.1.4     ✔ readr     2.1.5
## ✔ forcats   1.0.0     ✔ stringr   1.5.1
## ✔ ggplot2   3.5.1     ✔ tibble    3.2.1
## ✔ lubridate 1.9.3     ✔ tidyr     1.3.1
## ✔ purrr     1.0.2
## ── Conflicts ────────────────────────────────────────── tidyverse_conf
licts() ──
## ✖ dplyr::filter() masks stats::filter()
## ✖ dplyr::lag()    masks stats::lag()
## ℹ Use the conflicted package (<http://conflicted.r-lib.org/>) to for
ce all conflicts to become errors

library(sf)
```

```
## Warning: package 'sf' was built under R version 4.3.3

## Linking to GEOS 3.11.2, GDAL 3.8.2, PROJ 9.3.1; sf_use_s2() is TRUE

library(vegan)

## Warning: package 'vegan' was built under R version 4.3.3

## Loading required package: permute

## Warning: package 'permute' was built under R version 4.3.3

## Loading required package: lattice
## This is vegan 2.6-4

library(pvclust)

## Warning: package 'pvclust' was built under R version 4.3.3

library(dplyr)
library(ggplot2)
library(robis)

## Warning: package 'robis' was built under R version 4.3.3

library(obistools)
library(nortest) # for Anderson-Darling test
library(stringi) # for encoding UTF-8

## Warning: package 'stringi' was built under R version 4.3.3

library(corrplot)

## Warning: package 'corrplot' was built under R version 4.3.3

## corrplot 0.94 loaded

library(mgcv)

## Loading required package: nlme
##
## Attaching package: 'nlme'
##
## The following object is masked from 'package:dplyr':
##
##     collapse
##
## This is mgcv 1.9-0. For overview type 'help("mgcv-package")'.

library(ggeffects)

## Warning: package 'ggeffects' was built under R version 4.3.3

library(DHARMa) #simulations package gam-

## Warning: package 'DHARMa' was built under R version 4.3.3
```

```
## Warning in check_dep_version(): ABI version mismatch:
## lme4 was built with Matrix ABI version 1
## Current Matrix ABI version is 0
## Please re-install lme4 from source or restore original 'Matrix' packag
e

## This is DHARMa 0.4.6. For overview type '?DHARMa'. For recent changes,
type news(package = 'DHARMa')

library(knitr)

## Warning: package 'knitr' was built under R version 4.3.3

library(qpcR)

## Warning: package 'qpcR' was built under R version 4.3.3

## Loading required package: MASS
##
## Attaching package: 'MASS'
##
## The following object is masked from 'package:robis':
##
##     area
##
## The following object is masked from 'package:dplyr':
##
##     select
##
## Loading required package: minpack.lm

## Warning: package 'minpack.lm' was built under R version 4.3.3

## Loading required package: rgl

## Warning: package 'rgl' was built under R version 4.3.3

## Loading required package: robustbase

## Warning: package 'robustbase' was built under R version 4.3.3

## Loading required package: Matrix
##
## Attaching package: 'Matrix'
##
## The following objects are masked from 'package:tidyr':
##
##     expand, pack, unpack

#Species Counts and Environment, 5 degree bands
Ecological_Data_Global <- read.csv("Ecological_Data_Global.csv", sep = ";
")
summary(Ecological_Data_Global)
```

```
##       id             left          top              right        bot
tom
##  Min.   : 1.00   Min.   :-180   Min.   :-85.00   Min.   :180   Min.   
:-90.00  
##  1st Qu.: 9.75   1st Qu.:-180   1st Qu.:-41.25   1st Qu.:180   1st Qu.
:-46.25  
##  Median :18.50   Median :-180   Median :  2.50   Median :180   Median 
: -2.50  
##  Mean   :18.50   Mean   :-180   Mean   :  2.50   Mean   :180   Mean   
: -2.50  
##  3rd Qu.:27.25   3rd Qu.:-180   3rd Qu.: 46.25   3rd Qu.:180   3rd Qu.
: 41.25  
##  Max.   :36.00   Max.   :-180   Max.   : 90.00   Max.   :180   Max.   
: 85.00  
##                                                                        
##  Bathy_Max_Mean    Bathy_Mean_Mean   Bathy_Min_Mean    Chl_Mean_Mean   
##  Min.   :-4278.5   Min.   :-4194.3   Min.   :-4117.6   Min.   :0.09031  
##  1st Qu.:-4058.1   1st Qu.:-3970.8   1st Qu.:-3889.3   1st Qu.:0.13366  
##  Median :-3874.8   Median :-3790.1   Median :-3702.5   Median :0.25032  
##  Mean   :-3292.2   Mean   :-3225.1   Mean   :-3154.7   Mean   :0.26540  
##  3rd Qu.:-2944.1   3rd Qu.:-2883.2   3rd Qu.:-2820.7   3rd Qu.:0.37041  
##  Max.   : -761.9   Max.   : -741.7   Max.   : -721.3   Max.   :0.50726  
##  NA's   :2         NA's   :2         NA's   :2         NA's   :2        
##    Margin_Sum           Bathy_Max           Shelf_Sum          CurVel_
Mean     
##  Min.   :-322305404   Min.   :-3.072e+09   Min.   :-9362904   Min.   :
0.02525  
##  1st Qu.:-152911004   1st Qu.:-2.251e+09   1st Qu.:-1834335   1st Qu.:
0.04832  
##  Median :-110339888   Median :-2.014e+09   Median :-1300462   Median :
0.06613  
##  Mean   :-113889802   Mean   :-1.666e+09   Mean   :-1872021   Mean   :
0.08048  
##  3rd Qu.: -70897881   3rd Qu.:-7.886e+08   3rd Qu.: -705476   3rd Qu.:
0.11028  
##  Max.   :         0   Max.   : 0.000e+00   Max.   :       0   Max.   :
0.21723  
##                                                                NA's   :
2        
##  CurVel_Bot_Mean    Diff_Atte_Mean      Depth_Mean        HumImp_Sum    
##  Min.   :0.002405   Min.   :0.02947   Min.   :-4204.0   Min.   :       
0  
##  1st Qu.:0.014127   1st Qu.:0.06059   1st Qu.:-3962.4   1st Qu.:  8151
342  
##  Median :0.017665   Median :0.07062   Median :-3776.1   Median : 56012
445  
##  Mean   :0.018497   Mean   :0.07477   Mean   :-3204.4   Mean   : 68495
091  
##  3rd Qu.:0.020965   3rd Qu.:0.08153   3rd Qu.:-2826.0   3rd Qu.:103706
926  
##  Max.   :0.045673   Max.   :0.12402   Max.   : -655.6   Max.   :195804
```

```
477
##  NA's   :2          NA's   :2          NA's   :2          NA's   :4
##   HumImp_Mean     Ice_Cover_Sum       IceCov_Mean        Ice_Tick_Mean
##  Min.   : 1.342   Min.   :     0.0   Min.   :0.0000000   Min.   :0.000
0000
##  1st Qu.: 8.030   1st Qu.:     0.0   1st Qu.:0.0000000   1st Qu.:0.000
0000
##  Median :10.361   Median :     0.5   Median :0.0000077   Median :0.000
0115
##  Mean   : 9.725   Mean   : 81161.0   Mean   :0.1808575   Mean   :0.323
9475
##  3rd Qu.:12.446   3rd Qu.: 44888.8   3rd Qu.:0.2563673   3rd Qu.:0.286
3370
##  Max.   :18.138   Max.   :696353.9   Max.   :0.9671582   Max.   :2.458
3182
##  NA's   :7                           NA's   :2           NA's   :2
##   MixLay_Mean     PhotoActi_Mean   PrimProd_Mean    Salinity_Mean
##  Min.   : 19.95   Min.   : 3.877   Min.   :0.3574   Min.   :30.31
##  1st Qu.: 30.85   1st Qu.:24.399   1st Qu.:1.3064   1st Qu.:33.11
##  Median : 40.91   Median :28.955   Median :1.5059   Median :34.13
##  Mean   : 45.60   Mean   :32.202   Mean   :1.5172   Mean   :33.96
##  3rd Qu.: 56.22   3rd Qu.:43.915   3rd Qu.:1.6973   3rd Qu.:35.00
##  Max.   :103.59   Max.   :48.163   Max.   :2.3878   Max.   :35.67
##  NA's   :2        NA's   :2        NA's   :2        NA's   :2
##  Silicate_Mean      ThemM_Mean     Them_Max_Mean     Them_Max_Max
##  Min.   : 1.556   Min.   :-1.721   Min.   :-1.524   Min.   :-0.8473
##  1st Qu.: 2.356   1st Qu.: 2.327   1st Qu.: 6.581   1st Qu.:14.8983
##  Median : 5.867   Median :13.104   Median :18.786   Median :27.7568
##  Mean   :16.058   Mean   :13.340   Mean   :16.923   Mean   :23.2399
##  3rd Qu.:13.556   3rd Qu.:25.013   3rd Qu.:27.983   3rd Qu.:32.6277
##  Max.   :83.166   Max.   :28.215   Max.   :29.775   Max.   :36.4851
##  NA's   :2        NA's   :2        NA's   :2        NA's   :2
##  Temp_Min_Mean      Temp_Min_Min      ThemR_Mean      Them_Range_Max
##  Min.   :-1.95401   Min.   :-2.000   Min.   : 0.297   Min.   : 0.7454
##  1st Qu.:-0.01615   1st Qu.:-2.000   1st Qu.: 4.105   1st Qu.: 9.4402
##  Median : 8.38265   Median :-1.030   Median : 5.556   Median :11.1749
##  Mean   :10.64040   Mean   : 4.492   Mean   : 6.284   Mean   :12.6015
##  3rd Qu.:21.91526   3rd Qu.:11.822   3rd Qu.: 8.375   3rd Qu.:16.9521
##  Max.   :26.38238   Max.   :19.556   Max.   :13.863   Max.   :24.1779
##  NA's   :2          NA's   :2        NA's   :2        NA's   :2
##     Area_Sum           O2_Mean        Nitrate_Mean     Nitrate_Bot_Mea
n
##  Min.   :1.020e+11   Min.   :203.1   Min.   : 0.1658   Min.   :10.42
##  1st Qu.:6.495e+11   1st Qu.:212.0   1st Qu.: 0.5914   1st Qu.:26.76
##  Median :9.760e+11   Median :268.6   Median : 4.1394   Median :31.54
##  Mean   :9.857e+11   Mean   :273.6   Mean   : 7.9129   Mean   :27.32
##  3rd Qu.:1.422e+12   3rd Qu.:325.9   3rd Qu.: 9.5803   3rd Qu.:32.03
##  Max.   :2.050e+12   Max.   :371.1   Max.   :29.3414   Max.   :32.78
##                      NA's   :2       NA's   :2         NA's   :2
##   O2_Bot_Mean    PrimProd_Bot_Mean Salinity_Bot_Mean ThemM_Bot_Mean
##  Min.   :167.8   Min.   :0.01356   Min.   :32.22     Min.   :-1.0599
```

```
##  1st Qu.:184.0   1st Qu.:0.04705   1st Qu.:34.61     1st Qu.: 0.2374
##  Median :199.6   Median :0.09406   Median :34.69     Median : 2.0996
##  Mean   :219.7   Mean   :0.14575   Mean   :34.47     Mean   : 1.7394
##  3rd Qu.:257.0   3rd Qu.:0.16129   3rd Qu.:34.74     3rd Qu.: 2.9030
##  Max.   :314.2   Max.   :0.68720   Max.   :35.10     Max.   : 3.6338
##  NA's   :2       NA's   :2         NA's   :2         NA's   :2
##  ThemR_Bot_Mean        NumSpe          NumRec            NumPhy
##  Min.   :0.04462   Min.   :  494   Min.   :  28649   Min.   :17.00
##  1st Qu.:0.44255   1st Qu.: 6122   1st Qu.: 315808   1st Qu.:24.00
##  Median :0.53982   Median :16326   Median : 840696   Median :25.50
##  Mean   :0.86086   Mean   :16924   Mean   :1411624   Mean   :25.26
##  3rd Qu.:0.97004   3rd Qu.:25489   3rd Qu.:1893681   3rd Qu.:27.75
##  Max.   :3.53683   Max.   :38304   Max.   :7566285   Max.   :30.00
##  NA's   :2         NA's   :2       NA's   :2         NA's   :2
##       ES50         NumSpe_Sha      NumRec_Sha        NumPhy_Sha
##  Min.   :23.68   Min.   :  271   Min.   :   7056   Min.   :13.00
##  1st Qu.:39.81   1st Qu.: 4037   1st Qu.: 182153   1st Qu.:21.00
##  Median :42.21   Median :10746   Median : 496946   Median :23.00
##  Mean   :41.19   Mean   :10657   Mean   :1115969   Mean   :22.76
##  3rd Qu.:45.60   3rd Qu.:15559   3rd Qu.:1232565   3rd Qu.:26.00
##  Max.   :48.28   Max.   :25317   Max.   :7402048   Max.   :29.00
##  NA's   :2       NA's   :2       NA's   :2         NA's   :2
##     ES50_Sha       NumSpe_Mes     NumRec_Mes       NumPhy_Mes
##  Min.   :18.00   Min.   : 141   Min.   :  2391   Min.   :10.00
##  1st Qu.:35.25   1st Qu.:2937   1st Qu.: 22966   1st Qu.:17.00
##  Median :40.50   Median :3455   Median : 40485   Median :18.50
##  Mean   :38.53   Mean   :3689   Mean   :116802   Mean   :18.29
##  3rd Qu.:44.50   3rd Qu.:5003   3rd Qu.:110266   3rd Qu.:20.00
##  Max.   :47.00   Max.   :7275   Max.   :650912   Max.   :23.00
##  NA's   :2       NA's   :2      NA's   :2        NA's   :2
##     ES50_Mes       NumSpe_Dee     NumRec_Dee       NumPhy_Dee
##  Min.   :29.00   Min.   : 411   Min.   :  9251   Min.   :15.00
##  1st Qu.:39.00   1st Qu.:2920   1st Qu.: 21008   1st Qu.:18.00
##  Median :42.00   Median :3992   Median : 39600   Median :19.50
##  Mean   :42.21   Mean   :4270   Mean   : 80043   Mean   :20.12
##  3rd Qu.:47.00   3rd Qu.:5841   3rd Qu.: 97697   3rd Qu.:22.00
##  Max.   :49.00   Max.   :8735   Max.   :556993   Max.   :27.00
##  NA's   :2       NA's   :2      NA's   :2        NA's   :2
##     ES50_Dee
##  Min.   :29.00
##  1st Qu.:40.00
##  Median :42.50
##  Mean   :42.53
##  3rd Qu.:45.00
##  Max.   :49.00
##  NA's   :2
```

```
#First we’re going to load in our data and then trim the data frame down to just the columns we need.
analysis.cols <- c("Margin_Sum", "Depth_Mean", "HumImp_Mean", "CurVel_Bot_Mean", "Nitrate_Bot_Mean",
```

```
                   "O2_Bot_Mean","Area_Sum","PrimProd_Bot_Mean", "ThemM_B
ot_Mean", "NumSpe_Dee",
                   "NumRec_Dee",    "NumPhy_Dee",   "ES50_Dee")
Ecological_Data_Global <- Ecological_Data_Global[,analysis.cols]
Ecological_Data_Global  <- Ecological_Data_Global [complete.cases(Ecologi
cal_Data_Global ),]

# Calculate the correlation matrix
corr_matrix <- cor(Ecological_Data_Global)

# Create the correlation plot with black font for text
corrplot(corr_matrix, tl.col = "black")
```

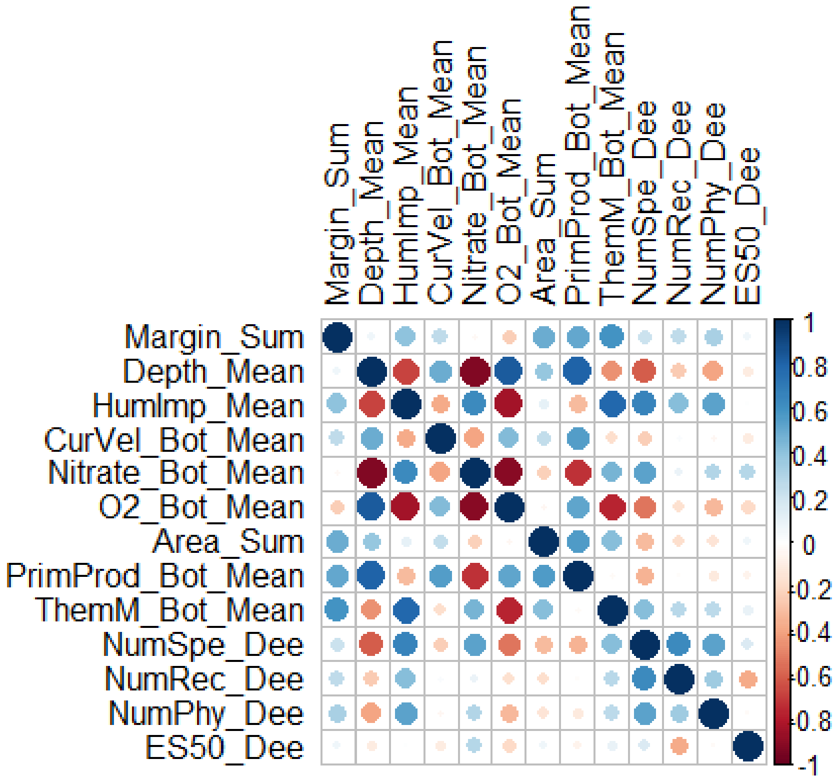


```
#GLMs for number of species, Global data, Deep
deep.numsp.intercept <- glm(NumSpe_Dee ~ 1, family = "poisson", data = Ec
ological_Data_Global)
summary(deep.numsp.intercept)

##
## Call:
## glm(formula = NumSpe_Dee ~ 1, family = "poisson", data = Ecological_Da
ta_Global)
##
## Coefficients:
##             Estimate Std. Error z value Pr(>|z|)
## (Intercept) 8.427193   0.002747    3068   <2e-16 ***
## ---
## Signif. codes:  0 '***' 0.001 '**' 0.01 '*' 0.05 '.' 0.1 ' ' 1
##
## (Dispersion parameter for poisson family taken to be 1)
```

```
##
##     Null deviance: 31827  on 28  degrees of freedom
## Residual deviance: 31827  on 28  degrees of freedom
## AIC: 32123
##
## Number of Fisher Scoring iterations: 4
```

```
deep.numsp.numrec <- glm(NumSpe_Dee ~ NumRec_Dee, family = "poisson", dat
a = Ecological_Data_Global)
summary(deep.numsp.numrec)
```

```
##
## Call:
## glm(formula = NumSpe_Dee ~ NumRec_Dee, family = "poisson", data = Ecol
ogical_Data_Global)
##
## Coefficients:
##              Estimate Std. Error z value Pr(>|z|)
## (Intercept) 8.222e+00  3.531e-03  2328.7   <2e-16 ***
## NumRec_Dee  1.937e-06  1.776e-08   109.1   <2e-16 ***
## ---
## Signif. codes:  0 '***' 0.001 '**' 0.01 '*' 0.05 '.' 0.1 ' ' 1
##
## (Dispersion parameter for poisson family taken to be 1)
##
##     Null deviance: 31827  on 28  degrees of freedom
## Residual deviance: 21956  on 27  degrees of freedom
## AIC: 22253
##
## Number of Fisher Scoring iterations: 4
```

```
deep.numsp.marginsum <- glm(NumSpe_Dee ~ NumRec_Dee + Margin_Sum, family
= "poisson", data = Ecological_Data_Global)
summary(deep.numsp.marginsum)
```

```
##
## Call:
## glm(formula = NumSpe_Dee ~ NumRec_Dee + Margin_Sum, family = "poisson"
,
##     data = Ecological_Data_Global)
##
## Coefficients:
##              Estimate Std. Error z value Pr(>|z|)
## (Intercept) 8.315e+00  7.661e-03 1085.27   <2e-16 ***
## NumRec_Dee  1.869e-06  1.851e-08  100.95   <2e-16 ***
## Margin_Sum  7.353e-10  5.476e-11   13.43   <2e-16 ***
## ---
## Signif. codes:  0 '***' 0.001 '**' 0.01 '*' 0.05 '.' 0.1 ' ' 1
##
## (Dispersion parameter for poisson family taken to be 1)
##
##     Null deviance: 31827  on 28  degrees of freedom
```

```
## Residual deviance: 21772  on 26  degrees of freedom
## AIC: 22071
##
## Number of Fisher Scoring iterations: 5
```

```r
deep.numsp.depth <- glm(NumSpe_Dee ~ NumRec_Dee + Depth_Mean, family = "p
oisson", data = Ecological_Data_Global)
summary(deep.numsp.depth)
```

```
##
## Call:
## glm(formula = NumSpe_Dee ~ NumRec_Dee + Depth_Mean, family = "poisson"
,
##     data = Ecological_Data_Global)
##
## Coefficients:
##               Estimate Std. Error z value Pr(>|z|)
## (Intercept)  7.267e+00  1.228e-02  591.88   <2e-16 ***
## NumRec_Dee   1.456e-06  1.869e-08   77.88   <2e-16 ***
## Depth_Mean  -2.905e-04  3.406e-06  -85.30   <2e-16 ***
## ---
## Signif. codes:  0 '***' 0.001 '**' 0.01 '*' 0.05 '.' 0.1 ' ' 1
##
## (Dispersion parameter for poisson family taken to be 1)
##
##     Null deviance: 31827  on 28  degrees of freedom
## Residual deviance: 13163  on 26  degrees of freedom
## AIC: 13463
##
## Number of Fisher Scoring iterations: 4
```

```r
deep.numsp.humimp <- glm(NumSpe_Dee ~ NumRec_Dee + HumImp_Mean, family =
"poisson", data = Ecological_Data_Global)
summary(deep.numsp.humimp)
```

```
##
## Call:
## glm(formula = NumSpe_Dee ~ NumRec_Dee + HumImp_Mean, family = "poisson
",
##     data = Ecological_Data_Global)
##
## Coefficients:
##              Estimate Std. Error z value Pr(>|z|)
## (Intercept) 7.696e+00  7.689e-03 1000.86   <2e-16 ***
## NumRec_Dee  1.125e-06  2.055e-08   54.77   <2e-16 ***
## HumImp_Mean 5.859e-02  7.165e-04   81.77   <2e-16 ***
## ---
## Signif. codes:  0 '***' 0.001 '**' 0.01 '*' 0.05 '.' 0.1 ' ' 1
##
## (Dispersion parameter for poisson family taken to be 1)
##
##     Null deviance: 31827  on 28  degrees of freedom
```

```
## Residual deviance: 15068  on 26  degrees of freedom
## AIC: 15367
##
## Number of Fisher Scoring iterations: 4
```

```
deep.numsp.curvel <- glm(NumSpe_Dee ~ NumRec_Dee + CurVel_Bot_Mean, famil
y = "poisson", data = Ecological_Data_Global)
summary(deep.numsp.curvel)
```

```
##
## Call:
## glm(formula = NumSpe_Dee ~ NumRec_Dee + CurVel_Bot_Mean, family = "poi
sson",
##     data = Ecological_Data_Global)
##
## Coefficients:
##                   Estimate Std. Error z value Pr(>|z|)
## (Intercept)      8.662e+00  9.604e-03  901.89   <2e-16 ***
## NumRec_Dee       2.005e-06  1.786e-08  112.30   <2e-16 ***
## CurVel_Bot_Mean -2.318e+01  4.861e-01  -47.69   <2e-16 ***
## ---
## Signif. codes:  0 '***' 0.001 '**' 0.01 '*' 0.05 '.' 0.1 ' ' 1
##
## (Dispersion parameter for poisson family taken to be 1)
##
##     Null deviance: 31827  on 28  degrees of freedom
## Residual deviance: 19449  on 26  degrees of freedom
## AIC: 19748
##
## Number of Fisher Scoring iterations: 4
```

```
deep.numsp.nitrate <- glm(NumSpe_Dee ~ NumRec_Dee + Nitrate_Bot_Mean, fam
ily = "poisson", data = Ecological_Data_Global)
summary(deep.numsp.nitrate)
```

```
##
## Call:
## glm(formula = NumSpe_Dee ~ NumRec_Dee + Nitrate_Bot_Mean, family = "po
isson",
##     data = Ecological_Data_Global)
##
## Coefficients:
##                   Estimate Std. Error z value Pr(>|z|)
## (Intercept)      6.914e+00  1.552e-02  445.60   <2e-16 ***
## NumRec_Dee       1.955e-06  1.817e-08  107.58   <2e-16 ***
## Nitrate_Bot_Mean 4.633e-02  5.145e-04   90.05   <2e-16 ***
## ---
## Signif. codes:  0 '***' 0.001 '**' 0.01 '*' 0.05 '.' 0.1 ' ' 1
##
## (Dispersion parameter for poisson family taken to be 1)
##
##     Null deviance: 31827  on 28  degrees of freedom
```

```
## Residual deviance: 12035  on 26  degrees of freedom
## AIC: 12335
##
## Number of Fisher Scoring iterations: 4
```

```
deep.numsp.oxygen <- glm(NumSpe_Dee ~ NumRec_Dee + O2_Bot_Mean, family =
"poisson", data = Ecological_Data_Global)
summary(deep.numsp.oxygen)
```

```
##
## Call:
## glm(formula = NumSpe_Dee ~ NumRec_Dee + O2_Bot_Mean, family = "poisson
",
##     data = Ecological_Data_Global)
##
## Coefficients:
##               Estimate Std. Error z value Pr(>|z|)
## (Intercept)  9.687e+00  1.730e-02  559.88   <2e-16 ***
## NumRec_Dee   1.748e-06  1.816e-08   96.30   <2e-16 ***
## O2_Bot_Mean -7.071e-03  8.440e-05  -83.78   <2e-16 ***
## ---
## Signif. codes:  0 '***' 0.001 '**' 0.01 '*' 0.05 '.' 0.1 ' ' 1
##
## (Dispersion parameter for poisson family taken to be 1)
##
##     Null deviance: 31827  on 28  degrees of freedom
## Residual deviance: 13617  on 26  degrees of freedom
## AIC: 13917
##
## Number of Fisher Scoring iterations: 4
```

```
deep.numsp.area <- glm(NumSpe_Dee ~ NumRec_Dee + Area_Sum, family = "pois
son", data = Ecological_Data_Global)
summary(deep.numsp.area)
```

```
##
## Call:
## glm(formula = NumSpe_Dee ~ NumRec_Dee + Area_Sum, family = "poisson",
##     data = Ecological_Data_Global)
##
## Coefficients:
##               Estimate Std. Error z value Pr(>|z|)
## (Intercept)  8.552e+00  8.659e-03  987.65   <2e-16 ***
## NumRec_Dee   1.804e-06  1.811e-08   99.61   <2e-16 ***
## Area_Sum    -2.753e-13  6.782e-15  -40.59   <2e-16 ***
## ---
## Signif. codes:  0 '***' 0.001 '**' 0.01 '*' 0.05 '.' 0.1 ' ' 1
##
## (Dispersion parameter for poisson family taken to be 1)
##
##     Null deviance: 31827  on 28  degrees of freedom
## Residual deviance: 20283  on 26  degrees of freedom
```

```
## AIC: 20583
##
## Number of Fisher Scoring iterations: 4
```

```
deep.numsp.primprod <- glm(NumSpe_Dee ~ NumRec_Dee + PrimProd_Bot_Mean, f
amily = "poisson", data = Ecological_Data_Global)
summary(deep.numsp.primprod)
```

```
##
## Call:
## glm(formula = NumSpe_Dee ~ NumRec_Dee + PrimProd_Bot_Mean, family = "p
oisson",
##     data = Ecological_Data_Global)
##
## Coefficients:
##                    Estimate Std. Error z value Pr(>|z|)
## (Intercept)        8.412e+00  4.468e-03 1882.84   <2e-16 ***
## NumRec_Dee         1.966e-06  1.786e-08  110.09   <2e-16 ***
## PrimProd_Bot_Mean -1.314e+00  2.132e-02  -61.65   <2e-16 ***
## ---
## Signif. codes:  0 '***' 0.001 '**' 0.01 '*' 0.05 '.' 0.1 ' ' 1
##
## (Dispersion parameter for poisson family taken to be 1)
##
##     Null deviance: 31827  on 28  degrees of freedom
## Residual deviance: 17615  on 26  degrees of freedom
## AIC: 17915
##
## Number of Fisher Scoring iterations: 4
```

```
deep.numsp.themmean <- glm(NumSpe_Dee ~ NumRec_Dee + ThemM_Bot_Mean, fami
ly = "poisson", data = Ecological_Data_Global)
summary(deep.numsp.themmean)
```

```
##
## Call:
## glm(formula = NumSpe_Dee ~ NumRec_Dee + ThemM_Bot_Mean, family = "pois
son",
##     data = Ecological_Data_Global)
##
## Coefficients:
##                Estimate Std. Error z value Pr(>|z|)
## (Intercept)    7.973e+00  6.334e-03 1258.88   <2e-16 ***
## NumRec_Dee     1.631e-06  1.880e-08   86.75   <2e-16 ***
## ThemM_Bot_Mean 1.257e-01  2.533e-03   49.64   <2e-16 ***
## ---
## Signif. codes:  0 '***' 0.001 '**' 0.01 '*' 0.05 '.' 0.1 ' ' 1
##
## (Dispersion parameter for poisson family taken to be 1)
##
##     Null deviance: 31827  on 28  degrees of freedom
## Residual deviance: 19398  on 26  degrees of freedom
```

```
## AIC: 19698
##
## Number of Fisher Scoring iterations: 5

#Model selection for number of species, Global
deep.numsp.models <- list(Intercept = deep.numsp.intercept,
                          NumRec = deep.numsp.numrec,
                          ConMar = deep.numsp.marginsum,
                          Depth = deep.numsp.depth,
                          HumImp = deep.numsp.humimp,
                          CurVel = deep.numsp.curvel,
                          Nitrate = deep.numsp.nitrate,
                          O2 = deep.numsp.oxygen,
                          Area = deep.numsp.area,
                          PriPro = deep.numsp.primprod,
                          TemMean = deep.numsp.themmean)
deep.numsp.aic.df <- data.frame(Model = names(deep.numsp.models),
                                AIC = sapply(deep.numsp.models, function(
x) AICc(x)),
                                akaike.weights(sapply(deep.numsp.models,
function(x) AICc(x))))


deep.numsp.aic.df <- deep.numsp.aic.df[order(deep.numsp.aic.df$AIC),]
deep.numsp.aic.df$Cumulative.Weight <- cumsum(deep.numsp.aic.df$weights)

kable(deep.numsp.aic.df, row.names = FALSE)
```

Model	AIC	deltaAIC	rel.LL	weights	Cumulative.Weight
Nitrate	12335.95	0.000	1	1	1
Depth	13463.65	1127.699	0	0	1
O2	13917.83	1581.877	0	0	1
HumImp	15368.42	3032.467	0	0	1
PriPro	17915.94	5579.988	0	0	1
TemMean	19698.91	7362.963	0	0	1
CurVel	19749.31	7413.361	0	0	1
Area	20583.68	8247.731	0	0	1
ConMar	22072.46	9736.506	0	0	1
NumRec	22253.75	9917.797	0	0	1
Intercept	32122.85	19786.896	0	0	1

```
#write.csv(deep.numsp.aic.df, file = "global.5.degree.numsp.deep.aic.csv"
)

#Plots for number of species, Deep
ggplot(Ecological_Data_Global, aes(x = NumRec_Dee, y = predict(deep.numsp
.numrec, Ecological_Data_Global))) +
  geom_smooth(method = "glm", formula = y ~ x, color = "#1a80bb", fill =
"#85bede") +  # Add a smooth dark blue line with light blue shadow
```

```
  geom_point(size = 3) +  # Add scatter plot points
  theme_bw() +  # Use the black and white theme
  scale_x_continuous(labels = scales::scientific) +
  labs(
    x = "Number of Records",  # Shorten the x-axis title
    y = "Predicted Value"  # Shorten the y-axis title
  ) +
  theme(
    panel.grid.minor = element_blank(),
    panel.grid.major = element_blank(),
    axis.text.x = element_text(size =20),  # Increase x-axis text size
    axis.text.y = element_text(size = 20),  # Increase y-axis text size
    axis.title.x = element_text(size = 22), # Increase x-axis title size
    axis.title.y = element_text(size = 22)  # Increase y-axis title size
  )
```

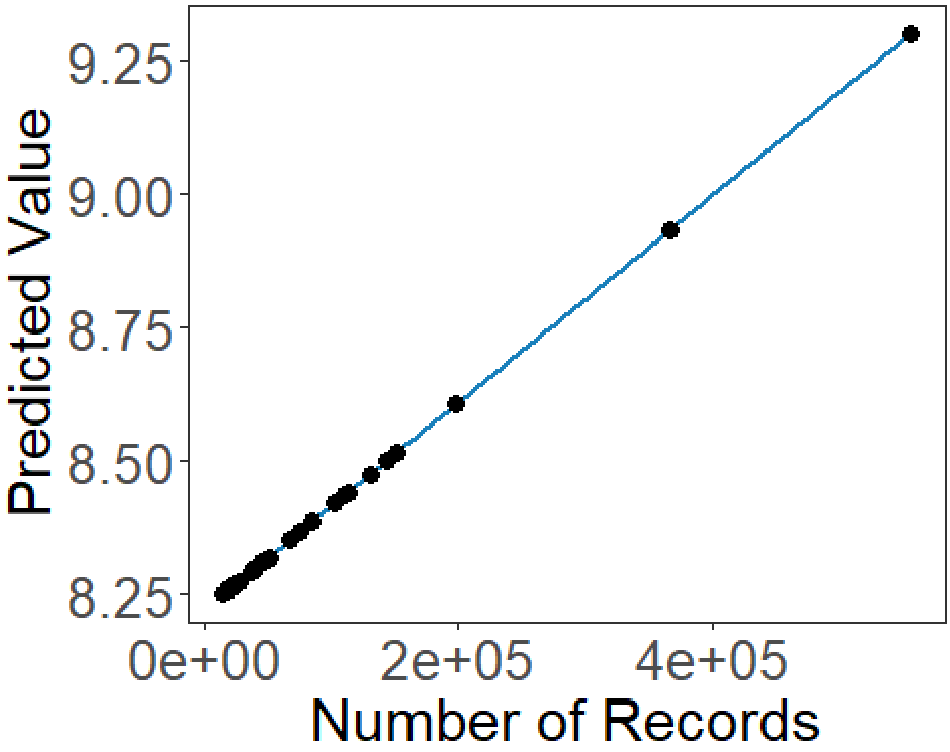


```
ggplot(Ecological_Data_Global, aes(x = Margin_Sum, y = predict(deep.numsp
.marginsum, Ecological_Data_Global))) +
  geom_smooth(method = "glm", formula = y ~ x, color = "#1a80bb", fill =
"#85bede") +  # Add a smooth dark blue line with light blue shadow
  geom_point(size = 3) +  # Add scatter plot points
  theme_bw() +  # Use the black and white theme
  scale_x_continuous(labels = scales::scientific) +
  labs(
    x = "Continental Margin (km2)",  # Shorten the x-axis title
    y = "Predicted Value"  # Shorten the y-axis title
  ) +
  theme(
    panel.grid.minor = element_blank(),
    panel.grid.major = element_blank(),
```

```
    axis.text.x = element_text(size =20),  # Increase x-axis text size
    axis.text.y = element_text(size = 20),  # Increase y-axis text size
    axis.title.x = element_text(size = 22), # Increase x-axis title size
    axis.title.y = element_text(size = 22)  # Increase y-axis title size
  )
```

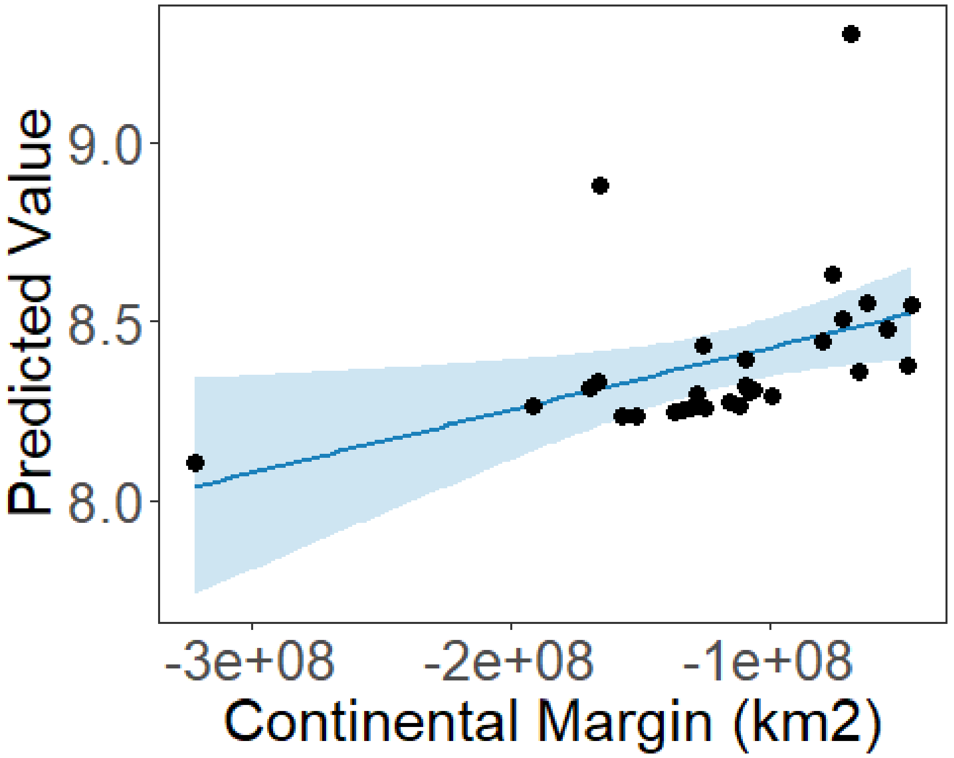


```
ggplot(Ecological_Data_Global, aes(x = Depth_Mean, y = predict(deep.numsp
.depth, Ecological_Data_Global))) +
  geom_smooth(method = "glm", formula = y ~ x, color = "#1a80bb", fill =
"#85bede") +  # Add a smooth dark blue line with light blue shadow
  geom_point(size = 3) +  # Add scatter plot points
  theme_bw() +  # Use the black and white theme
  scale_x_continuous(labels = scales::scientific) +
  labs(
    x = "Depth (m)",  # Shorten the x-axis title
    y = "Predicted Value"  # Shorten the y-axis title
  ) +
  theme(
    panel.grid.minor = element_blank(),
    panel.grid.major = element_blank(),
    axis.text.x = element_text(size =20),  # Increase x-axis text size
    axis.text.y = element_text(size = 20),  # Increase y-axis text size
    axis.title.x = element_text(size = 22), # Increase x-axis title size
    axis.title.y = element_text(size = 22)  # Increase y-axis title size
  )
```

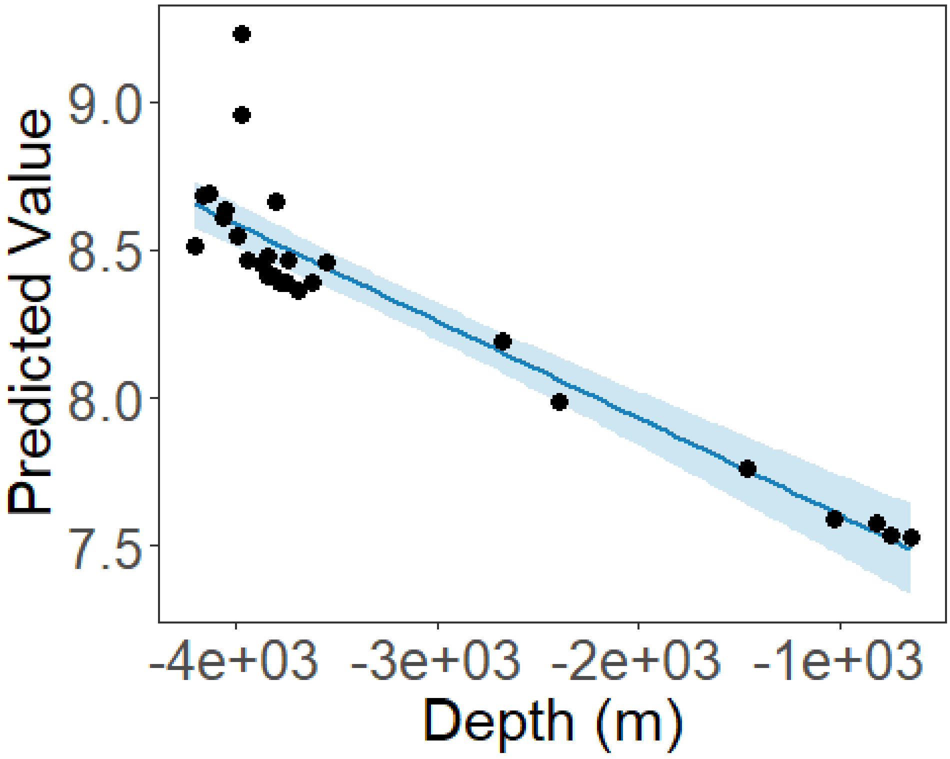


```
ggplot(Ecological_Data_Global, aes(x = HumImp_Mean, y = predict(deep.nums
p.humimp, Ecological_Data_Global))) +
  geom_smooth(method = "glm", formula = y ~ x, color = "#1a80bb", fill =
"#85bede") +  # Add a smooth dark blue line with light blue shadow
  geom_point(size = 3) +  # Add scatter plot points
  theme_bw() +  # Use the black and white theme
  labs(
    x = "Human Impact",  # Shorten the x-axis title
    y = "Predicted Value"  # Shorten the y-axis title
  ) +
  theme(
    panel.grid.minor = element_blank(),
    panel.grid.major = element_blank(),
    axis.text.x = element_text(size =20),  # Increase x-axis text size
    axis.text.y = element_text(size = 20),  # Increase y-axis text size
    axis.title.x = element_text(size = 22), # Increase x-axis title size
    axis.title.y = element_text(size = 22)  # Increase y-axis title size
  )
```

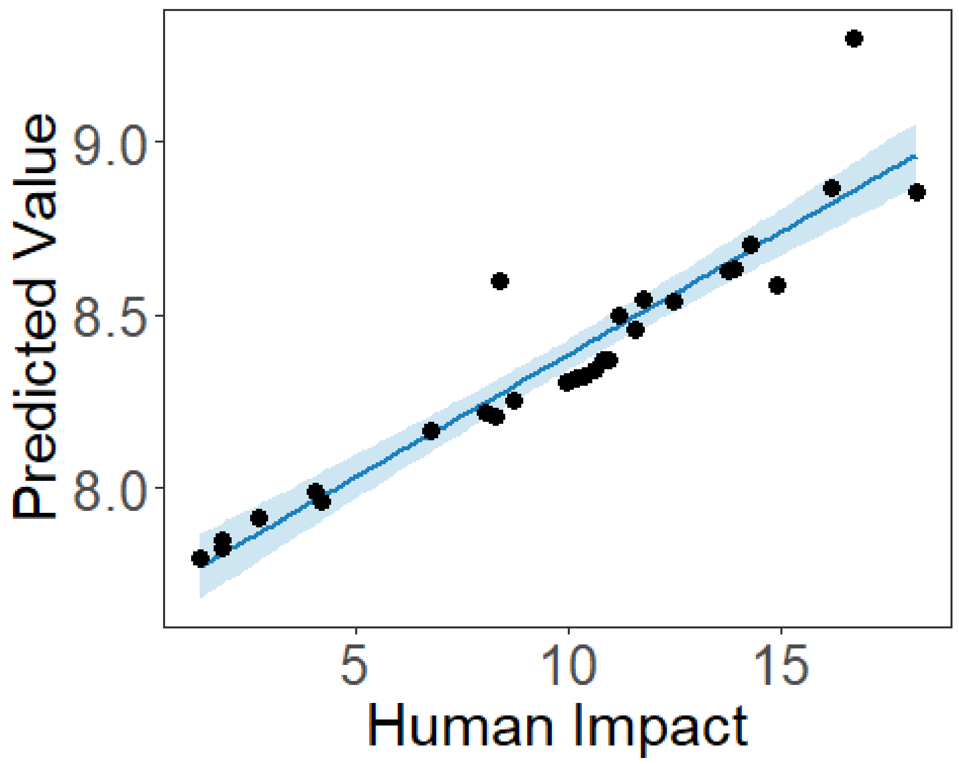


```r
ggplot(Ecological_Data_Global, aes(x = CurVel_Bot_Mean, y = predict(deep.
numsp.curvel, Ecological_Data_Global))) +
  geom_smooth(method = "glm", formula = y ~ x, color = "#1a80bb", fill =
"#85bede") +  # Add a smooth dark blue line with light blue shadow
  geom_point(size = 3) +  # Add scatter plot points
  theme_bw() +  # Use the black and white theme
  labs(
    x = "Current Velocity (m.s-1)",  # Shorten the x-axis title
    y = "Predicted Value"  # Shorten the y-axis title
  ) +
  theme(
    panel.grid.minor = element_blank(),
    panel.grid.major = element_blank(),
    axis.text.x = element_text(size =20),  # Increase x-axis text size
    axis.text.y = element_text(size = 20),  # Increase y-axis text size
    axis.title.x = element_text(size = 22), # Increase x-axis title size
    axis.title.y = element_text(size = 22)  # Increase y-axis title size
  )
```

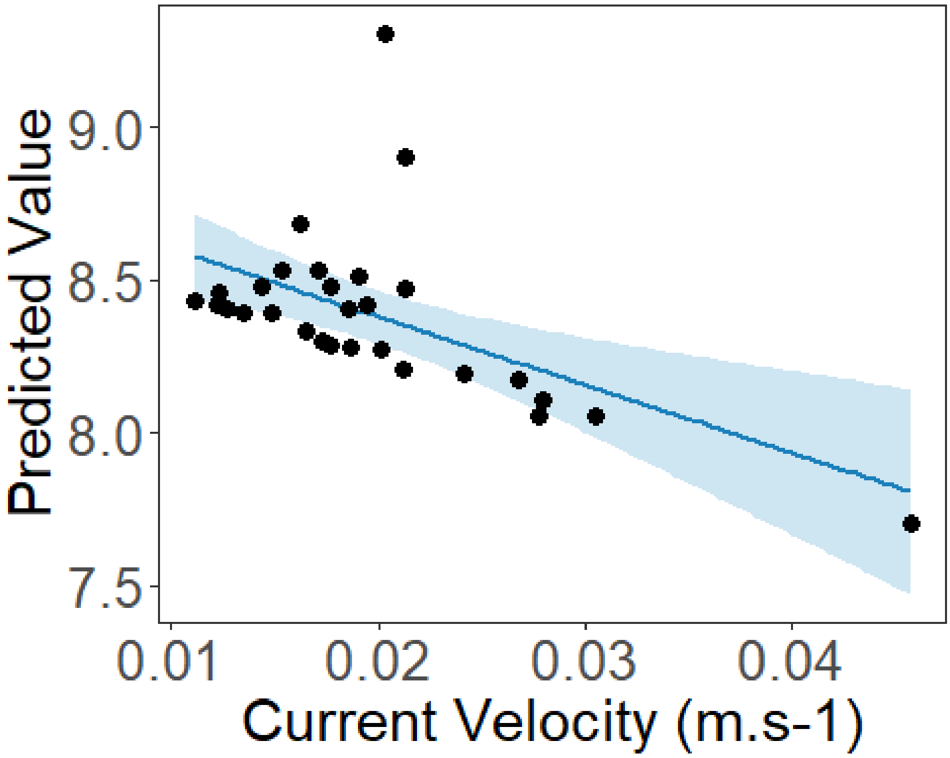


```r
ggplot(Ecological_Data_Global, aes(x = Nitrate_Bot_Mean, y = predict(deep
.numsp.nitrate, Ecological_Data_Global))) +
  geom_smooth(method = "glm", formula = y ~ x, color = "#1a80bb", fill =
"#85bede") +  # Add a smooth dark blue line with light blue shadow
  geom_point(size = 3) +  # Add scatter plot points
  theme_bw() +  # Use the black and white theme
  labs(
    x = "Nitrate (mmol . m-3)",  # Shorten the x-axis title
    y = "Predicted Value"  # Shorten the y-axis title
  ) +
  theme(
    panel.grid.minor = element_blank(),
    panel.grid.major = element_blank(),
    axis.text.x = element_text(size =20),  # Increase x-axis text size
    axis.text.y = element_text(size = 20),  # Increase y-axis text size
    axis.title.x = element_text(size = 22), # Increase x-axis title size
    axis.title.y = element_text(size = 22)  # Increase y-axis title size
  )
```

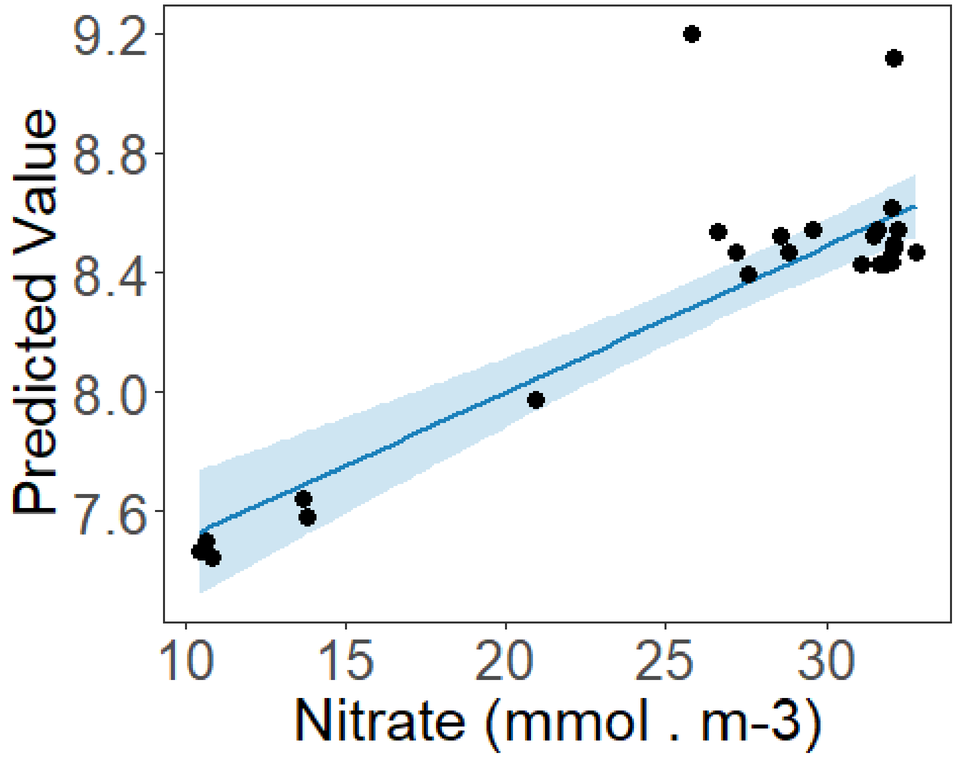


```r
ggplot(Ecological_Data_Global, aes(x = O2_Bot_Mean, y = predict(deep.nums
p.oxygen, Ecological_Data_Global))) +
  geom_smooth(method = "glm", formula = y ~ x, color = "#1a80bb", fill =
"#85bede") +  # Add a smooth dark blue line with light blue shadow
  geom_point(size = 3) +  # Add scatter plot points
  theme_bw() +  # Use the black and white theme
  labs(
    x = "O2 (mmol . m-3)",  # Shorten the x-axis title
    y = "Predicted Value"  # Shorten the y-axis title
  ) +
  theme(
    panel.grid.minor = element_blank(),
    panel.grid.major = element_blank(),
    axis.text.x = element_text(size =20),  # Increase x-axis text size
    axis.text.y = element_text(size = 20),  # Increase y-axis text size
    axis.title.x = element_text(size = 22), # Increase x-axis title size
    axis.title.y = element_text(size = 22)  # Increase y-axis title size
  )
```

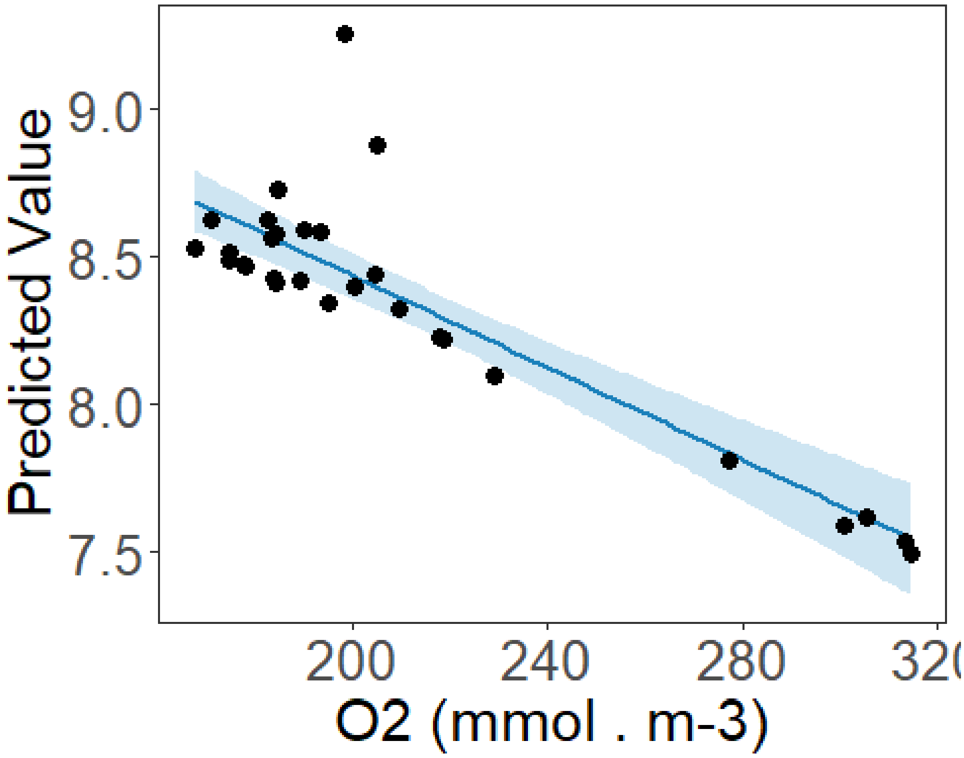


```r
ggplot(Ecological_Data_Global, aes(x = Area_Sum, y = predict(deep.numsp.a
rea, Ecological_Data_Global))) +
  geom_smooth(method = "glm", formula = y ~ x, color = "#1a80bb", fill =
"#85bede") +  # Add a smooth dark blue line with light blue shadow
  geom_point(size = 3) +  # Add scatter plot points
  theme_bw() +  # Use the black and white theme
  scale_x_continuous(labels = scales::scientific) +
  labs(
    x = "Area (km2)",  # Shorten the x-axis title
    y = "Predicted Value"  # Shorten the y-axis title
  ) +
  theme(
    panel.grid.minor = element_blank(),
    panel.grid.major = element_blank(),
    axis.text.x = element_text(size =20),  # Increase x-axis text size
    axis.text.y = element_text(size = 20),  # Increase y-axis text size
    axis.title.x = element_text(size = 22), # Increase x-axis title size
    axis.title.y = element_text(size = 22)  # Increase y-axis title size
  )
```

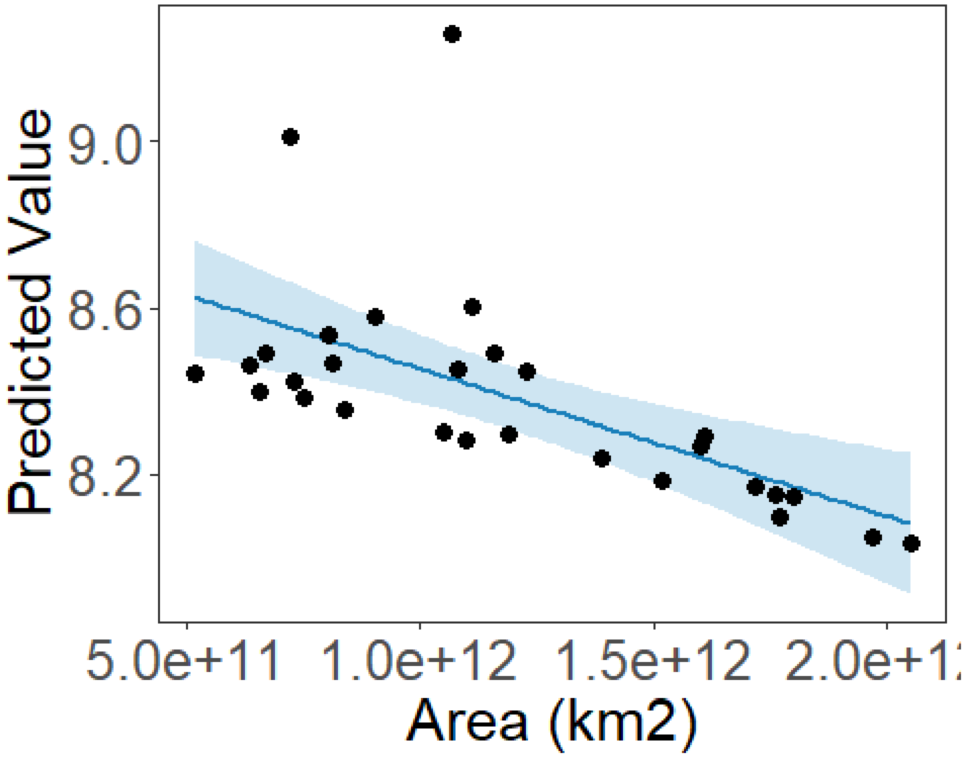


```
ggplot(Ecological_Data_Global, aes(x = PrimProd_Bot_Mean, y = predict(dee
p.numsp.primprod, Ecological_Data_Global))) +
  geom_smooth(method = "glm", formula = y ~ x, color = "#1a80bb", fill =
"#85bede") +  # Add a smooth dark blue line with light blue shadow
  geom_point(size = 3) +  # Add scatter plot points
  theme_bw() +  # Use the black and white theme
  labs(
    x = "Primary Productivity (mmol . m-3)",  # Shorten the x-axis title
    y = "Predicted Value"  # Shorten the y-axis title
  ) +
  theme(
    panel.grid.minor = element_blank(),
    panel.grid.major = element_blank(),
    axis.text.x = element_text(size =20),  # Increase x-axis text size
    axis.text.y = element_text(size = 20),  # Increase y-axis text size
    axis.title.x = element_text(size = 22), # Increase x-axis title size
    axis.title.y = element_text(size = 22)  # Increase y-axis title size
  )
```

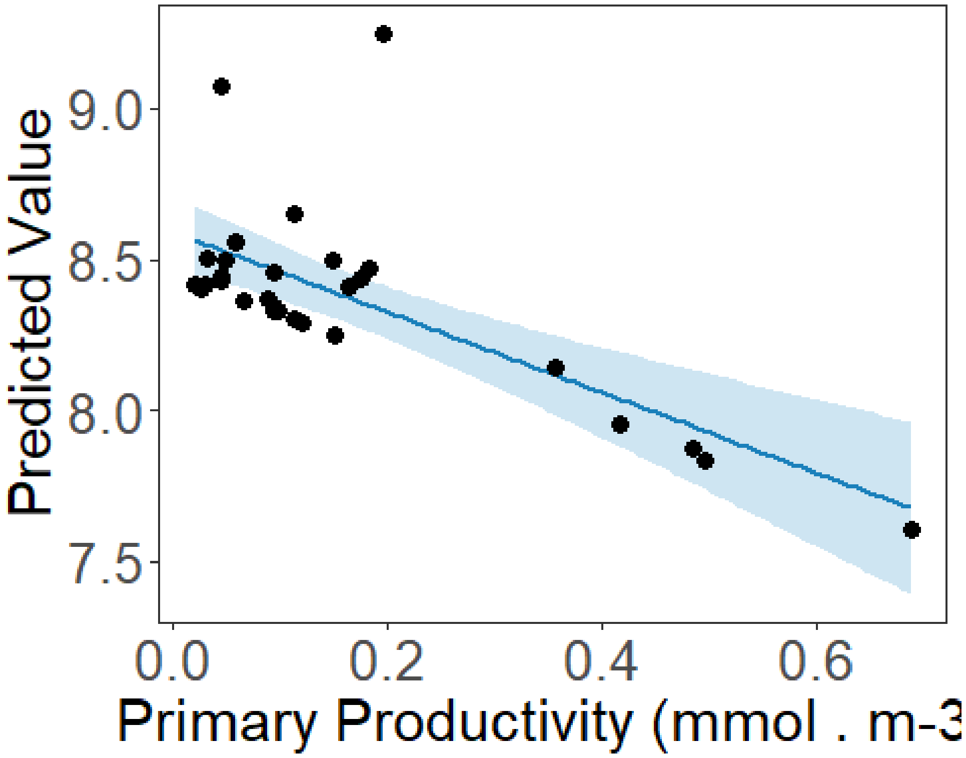


```
ggplot(Ecological_Data_Global, aes(x = ThemM_Bot_Mean, y = predict(deep.n
umsp.themmean, Ecological_Data_Global))) +
  geom_smooth(method = "glm", formula = y ~ x, color = "#1a80bb", fill =
"#85bede") +  # Add a smooth dark blue line with light blue shadow
  geom_point(size = 3) +  # Add scatter plot points
  theme_bw() +  # Use the black and white theme
  labs(
    x = "Temperature Mean (ºC)",  # Shorten the x-axis title
    y = "Predicted Value"  # Shorten the y-axis title
  ) +
  theme(
    panel.grid.minor = element_blank(),
    panel.grid.major = element_blank(),
    axis.text.x = element_text(size =20),  # Increase x-axis text size
    axis.text.y = element_text(size = 20),  # Increase y-axis text size
    axis.title.x = element_text(size = 22), # Increase x-axis title size
    axis.title.y = element_text(size = 22)  # Increase y-axis title size
  )
```

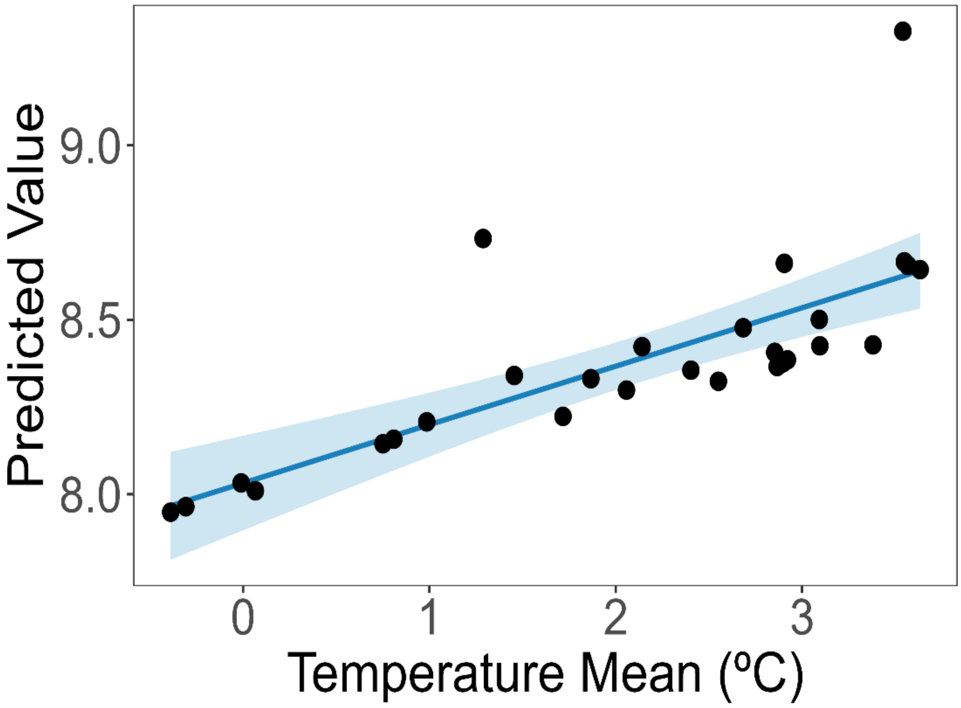


```
# ===================================
#                DEEP-ES50-GLM
# ===================================

deep.es50.intercept <- glm(ES50_Dee ~ 1, family = "poisson", data = Ecolo
gical_Data_Global)
summary(deep.es50.intercept)

##
## Call:
## glm(formula = ES50_Dee ~ 1, family = "poisson", data = Ecological_Data
_Global)
##
## Coefficients:
##             Estimate Std. Error z value Pr(>|z|)
## (Intercept)  3.75799    0.02836   132.5   <2e-16 ***
## ---
## Signif. codes:  0 '***' 0.001 '**' 0.01 '*' 0.05 '.' 0.1 ' ' 1
##
## (Dispersion parameter for poisson family taken to be 1)
##
##     Null deviance: 8.5605  on 28  degrees of freedom
## Residual deviance: 8.5605  on 28  degrees of freedom
## AIC: 172.85
##
## Number of Fisher Scoring iterations: 3

deep.es50.numrec <- glm(ES50_Dee ~ NumRec_Dee, family = "poisson", data =
Ecological_Data_Global)
summary(deep.es50.numrec)
```

```
##
## Call:
## glm(formula = ES50_Dee ~ NumRec_Dee, family = "poisson", data = Ecolog
ical_Data_Global)
##
## Coefficients:
##                Estimate Std. Error z value Pr(>|z|)
## (Intercept)  3.783e+00  3.613e-02 104.711   <2e-16 ***
## NumRec_Dee  -2.817e-07  2.590e-07  -1.088    0.277
## ---
## Signif. codes:  0 '***' 0.001 '**' 0.01 '*' 0.05 '.' 0.1 ' ' 1
##
## (Dispersion parameter for poisson family taken to be 1)
##
##     Null deviance: 8.5605  on 28  degrees of freedom
## Residual deviance: 7.3413  on 27  degrees of freedom
## AIC: 173.63
##
## Number of Fisher Scoring iterations: 3
```

```
deep.es50.marginsum <- glm(ES50_Dee ~ Margin_Sum, family = "poisson", dat
a = Ecological_Data_Global)
summary(deep.es50.marginsum)
```

```
##
## Call:
## glm(formula = ES50_Dee ~ Margin_Sum, family = "poisson", data = Ecolog
ical_Data_Global)
##
## Coefficients:
##               Estimate Std. Error z value Pr(>|z|)
## (Intercept) 3.770e+00  6.765e-02  55.723   <2e-16 ***
## Margin_Sum  9.939e-11  5.206e-10   0.191    0.849
## ---
## Signif. codes:  0 '***' 0.001 '**' 0.01 '*' 0.05 '.' 0.1 ' ' 1
##
## (Dispersion parameter for poisson family taken to be 1)
##
##     Null deviance: 8.5605  on 28  degrees of freedom
## Residual deviance: 8.5239  on 27  degrees of freedom
## AIC: 174.82
##
## Number of Fisher Scoring iterations: 3
```

```
deep.es50.depth <- glm(ES50_Dee ~ Depth_Mean, family = "poisson", data =
Ecological_Data_Global)
summary(deep.es50.depth)
```

```
##
## Call:
## glm(formula = ES50_Dee ~ Depth_Mean, family = "poisson", data = Ecolog
ical_Data_Global)
```

```
##
## Coefficients:
##               Estimate Std. Error z value Pr(>|z|)
## (Intercept)  3.732e+00  8.744e-02  42.679   <2e-16 ***
## Depth_Mean  -7.967e-06  2.513e-05  -0.317    0.751
## ---
## Signif. codes:  0 '***' 0.001 '**' 0.01 '*' 0.05 '.' 0.1 ' ' 1
##
## (Dispersion parameter for poisson family taken to be 1)
##
##     Null deviance: 8.5605  on 28  degrees of freedom
## Residual deviance: 8.4595  on 27  degrees of freedom
## AIC: 174.75
##
## Number of Fisher Scoring iterations: 3
```

```
deep.es50.humimp <- glm(ES50_Dee ~ HumImp_Mean, family = "poisson", data
= Ecological_Data_Global)
summary(deep.es50.humimp)
```

```
##
## Call:
## glm(formula = ES50_Dee ~ HumImp_Mean, family = "poisson", data = Ecolo
gical_Data_Global)
##
## Coefficients:
##              Estimate Std. Error z value Pr(>|z|)
## (Intercept) 3.7562883  0.0675880  55.576   <2e-16 ***
## HumImp_Mean 0.0001747  0.0063059   0.028    0.978
## ---
## Signif. codes:  0 '***' 0.001 '**' 0.01 '*' 0.05 '.' 0.1 ' ' 1
##
## (Dispersion parameter for poisson family taken to be 1)
##
##     Null deviance: 8.5605  on 28  degrees of freedom
## Residual deviance: 8.5597  on 27  degrees of freedom
## AIC: 174.85
##
## Number of Fisher Scoring iterations: 3
```

```
deep.es50.curvel <- glm(ES50_Dee ~ CurVel_Bot_Mean, family = "poisson", d
ata = Ecological_Data_Global)
summary(deep.es50.curvel)
```

```
##
## Call:
## glm(formula = ES50_Dee ~ CurVel_Bot_Mean, family = "poisson",
##     data = Ecological_Data_Global)
##
## Coefficients:
##                  Estimate Std. Error z value Pr(>|z|)
## (Intercept)       3.78257    0.08598  43.993   <2e-16 ***
```

```
## CurVel_Bot_Mean -1.24940    4.13082  -0.302    0.762
## ---
## Signif. codes:  0 '***' 0.001 '**' 0.01 '*' 0.05 '.' 0.1 ' ' 1
##
## (Dispersion parameter for poisson family taken to be 1)
##
##     Null deviance: 8.5605  on 28  degrees of freedom
## Residual deviance: 8.4685  on 27  degrees of freedom
## AIC: 174.76
##
## Number of Fisher Scoring iterations: 3
```

```
deep.es50.nitrate <- glm(ES50_Dee ~ Nitrate_Bot_Mean, family = "poisson",
data = Ecological_Data_Global)
summary(deep.es50.nitrate)
```

```
##
## Call:
## glm(formula = ES50_Dee ~ Nitrate_Bot_Mean, family = "poisson",
##     data = Ecological_Data_Global)
##
## Coefficients:
##                  Estimate Std. Error z value Pr(>|z|)
## (Intercept)      3.669200   0.109493  33.511   <2e-16 ***
## Nitrate_Bot_Mean 0.003267   0.003879   0.842      0.4
## ---
## Signif. codes:  0 '***' 0.001 '**' 0.01 '*' 0.05 '.' 0.1 ' ' 1
##
## (Dispersion parameter for poisson family taken to be 1)
##
##     Null deviance: 8.5605  on 28  degrees of freedom
## Residual deviance: 7.8428  on 27  degrees of freedom
## AIC: 174.14
##
## Number of Fisher Scoring iterations: 3
```

```
deep.es50.oxygen <- glm(ES50_Dee ~ O2_Bot_Mean, family = "poisson", data
= Ecological_Data_Global)
summary(deep.es50.oxygen)
```

```
##
## Call:
## glm(formula = ES50_Dee ~ O2_Bot_Mean, family = "poisson", data = Ecolo
gical_Data_Global)
##
## Coefficients:
##              Estimate Std. Error z value Pr(>|z|)
## (Intercept)  3.835006   0.138657  27.658   <2e-16 ***
## O2_Bot_Mean -0.000366   0.000646  -0.567    0.571
## ---
## Signif. codes:  0 '***' 0.001 '**' 0.01 '*' 0.05 '.' 0.1 ' ' 1
##
```

```
## (Dispersion parameter for poisson family taken to be 1)
##
##     Null deviance: 8.5605  on 28  degrees of freedom
## Residual deviance: 8.2370  on 27  degrees of freedom
## AIC: 174.53
##
## Number of Fisher Scoring iterations: 3
```

```
deep.es50.area <- glm(ES50_Dee ~ Area_Sum, family = "poisson", data = Eco
logical_Data_Global)
summary(deep.es50.area)
```

```
##
## Call:
## glm(formula = ES50_Dee ~ Area_Sum, family = "poisson", data = Ecologic
al_Data_Global)
##
## Coefficients:
##              Estimate Std. Error z value Pr(>|z|)
## (Intercept) 3.743e+00  8.098e-02  46.221   <2e-16 ***
## Area_Sum    1.261e-14  6.416e-14   0.196    0.844
## ---
## Signif. codes:  0 '***' 0.001 '**' 0.01 '*' 0.05 '.' 0.1 ' ' 1
##
## (Dispersion parameter for poisson family taken to be 1)
##
##     Null deviance: 8.5605  on 28  degrees of freedom
## Residual deviance: 8.5219  on 27  degrees of freedom
## AIC: 174.81
##
## Number of Fisher Scoring iterations: 3
```

```
deep.es50.primprod <- glm(ES50_Dee ~ PrimProd_Bot_Mean, family = "poisson
", data = Ecological_Data_Global)
summary(deep.es50.primprod)
```

```
##
## Call:
## glm(formula = ES50_Dee ~ PrimProd_Bot_Mean, family = "poisson",
##     data = Ecological_Data_Global)
##
## Coefficients:
##                   Estimate Std. Error z value Pr(>|z|)
## (Intercept)        3.76320    0.03995  94.196   <2e-16 ***
## PrimProd_Bot_Mean -0.03229    0.17464  -0.185    0.853
## ---
## Signif. codes:  0 '***' 0.001 '**' 0.01 '*' 0.05 '.' 0.1 ' ' 1
##
## (Dispersion parameter for poisson family taken to be 1)
##
##     Null deviance: 8.5605  on 28  degrees of freedom
## Residual deviance: 8.5262  on 27  degrees of freedom
```

```
## AIC: 174.82
##
## Number of Fisher Scoring iterations: 3
```

```
deep.es50.themmean <- glm(ES50_Dee ~ ThemM_Bot_Mean, family = "poisson",
data = Ecological_Data_Global)
summary(deep.es50.themmean)
```

```
##
## Call:
## glm(formula = ES50_Dee ~ ThemM_Bot_Mean, family = "poisson",
##     data = Ecological_Data_Global)
##
## Coefficients:
##                Estimate Std. Error z value Pr(>|z|)
## (Intercept)    3.744781   0.056971  65.731   <2e-16 ***
## ThemM_Bot_Mean 0.006239   0.023292   0.268    0.789
## ---
## Signif. codes:  0 '***' 0.001 '**' 0.01 '*' 0.05 '.' 0.1 ' ' 1
##
## (Dispersion parameter for poisson family taken to be 1)
##
##     Null deviance: 8.5605  on 28  degrees of freedom
## Residual deviance: 8.4886  on 27  degrees of freedom
## AIC: 174.78
##
## Number of Fisher Scoring iterations: 3
```

```
#Model selection for number of species, Global
deep.es50.models <- list(Intercept = deep.es50.intercept,
                         NumRec = deep.es50.numrec,
                         ConMar = deep.es50.marginsum,
                         Depth = deep.es50.depth,
                         HumImp = deep.es50.humimp,
                         CurVel = deep.es50.curvel,
                         Nitrate = deep.es50.nitrate,
                         O2 = deep.es50.oxygen,
                         Area = deep.es50.area,
                         PriPro = deep.es50.primprod,
                         TemMea = deep.es50.themmean)
deep.es50.aic.df <- data.frame(Model = names(deep.es50.models),
                               AIC = sapply(deep.es50.models, function(x)
AICc(x)),
                               akaike.weights(sapply(deep.es50.models, fu
nction(x) AICc(x))))


deep.es50.aic.df <- deep.es50.aic.df[order(deep.es50.aic.df$AIC),]
deep.es50.aic.df$Cumulative.Weight <- cumsum(deep.es50.aic.df$weights)

kable(deep.es50.aic.df, row.names = FALSE)
```

Model	AIC	deltaAIC	rel.LL	weights	Cumulative.Weight
Intercept	173.0017	0.000000	1.0000000	0.2145752	0.2145752
NumRec	174.0960	1.094281	0.5786019	0.1241536	0.3387288
Nitrate	174.5975	1.595752	0.4502843	0.0966198	0.4353486
O2	174.9917	1.989959	0.3697310	0.0793351	0.5146837
Depth	175.2141	2.212416	0.3308110	0.0709838	0.5856676
CurVel	175.2231	2.221421	0.3293248	0.0706649	0.6563325
TemMea	175.2432	2.241509	0.3260336	0.0699587	0.7262912
Area	175.2765	2.274807	0.3206505	0.0688036	0.7950949
ConMar	175.2786	2.276835	0.3203255	0.0687339	0.8638288
PriPro	175.2808	2.279095	0.3199637	0.0686563	0.9324851
HumImp	175.3143	2.312623	0.3146446	0.0675149	1.0000000

```
#write.csv(deep.es50.aic.df, file = "deep.5.degree.es50.deep.aic.csv")

#Plots for ES50, Deep
ggplot(Ecological_Data_Global, aes(x = NumRec_Dee, y = ES50_Dee)) +
  geom_smooth(method = "glm", formula = y ~ x, color = "#1a80bb", fill =
"#85bede") +  # Add a smooth dark blue line with light blue shadow
  geom_point(size = 3) +  # Add scatter plot points
  theme_bw() +  # Use the black and white theme
  scale_x_continuous(labels = scales::scientific) +
  scale_y_continuous(limits=c(29, 52), breaks=seq(30, 50, by=5), expand =
c(0, 0)) +
  labs(
    x = "Number of Records",  # Shorten the x-axis title
    y = "ES50"  # Shorten the y-axis title
  ) +
  theme(
    panel.grid.minor = element_blank(),
    panel.grid.major = element_blank(),
    axis.text.x = element_text(size =20),  # Increase x-axis text size
    axis.text.y = element_text(size = 20),  # Increase y-axis text size
    axis.title.x = element_text(size = 22), # Increase x-axis title size
    axis.title.y = element_text(size = 22)  # Increase y-axis title size
  )
```

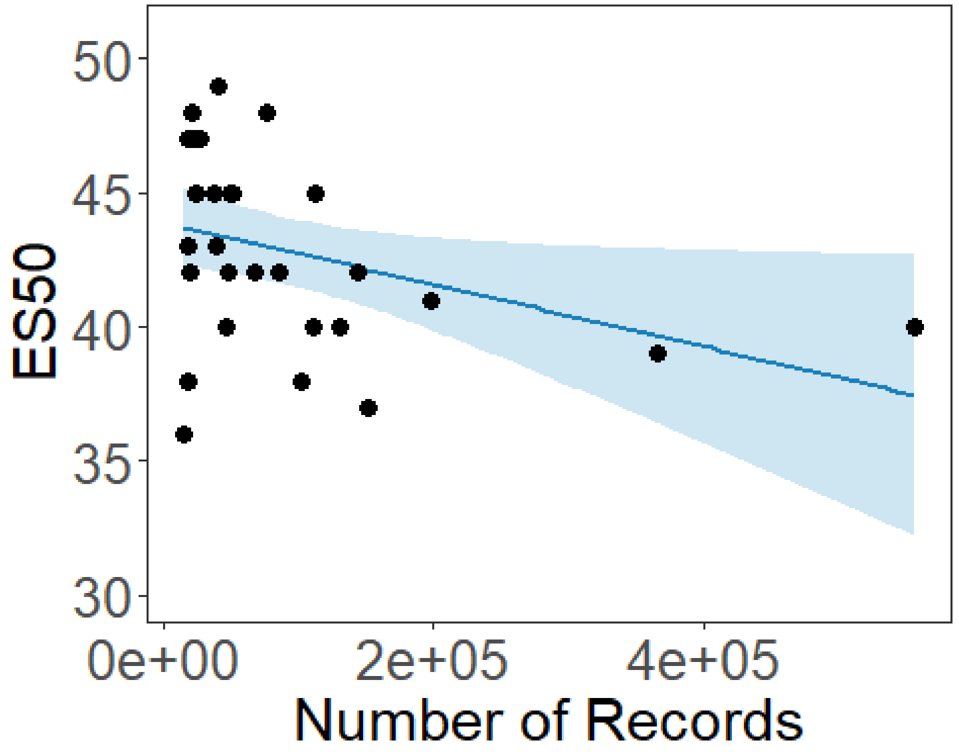


```
ggplot(Ecological_Data_Global, aes(x = Margin_Sum, y = ES50_Dee)) +
  geom_smooth(method = "glm", formula = y ~ x, color = "#1a80bb", fill =
"#85bede") +  # Add a smooth dark blue line with light blue shadow
  geom_point(size = 3) +  # Add scatter plot points
  theme_bw() +  # Use the black and white theme
  scale_x_continuous(labels = scales::scientific) +
  scale_y_continuous(limits=c(29, 52), breaks=seq(30, 50, by=5), expand =
c(0, 0)) +
  labs(
    x = "Continental Margin (km2)",  # Shorten the x-axis title
    y = "ES50"  # Shorten the y-axis title
  ) +
  theme(
    panel.grid.minor = element_blank(),
    panel.grid.major = element_blank(),
    axis.text.x = element_text(size =20),  # Increase x-axis text size
    axis.text.y = element_text(size = 20),  # Increase y-axis text size
    axis.title.x = element_text(size = 22), # Increase x-axis title size
    axis.title.y = element_text(size = 22)  # Increase y-axis title size
  )
```

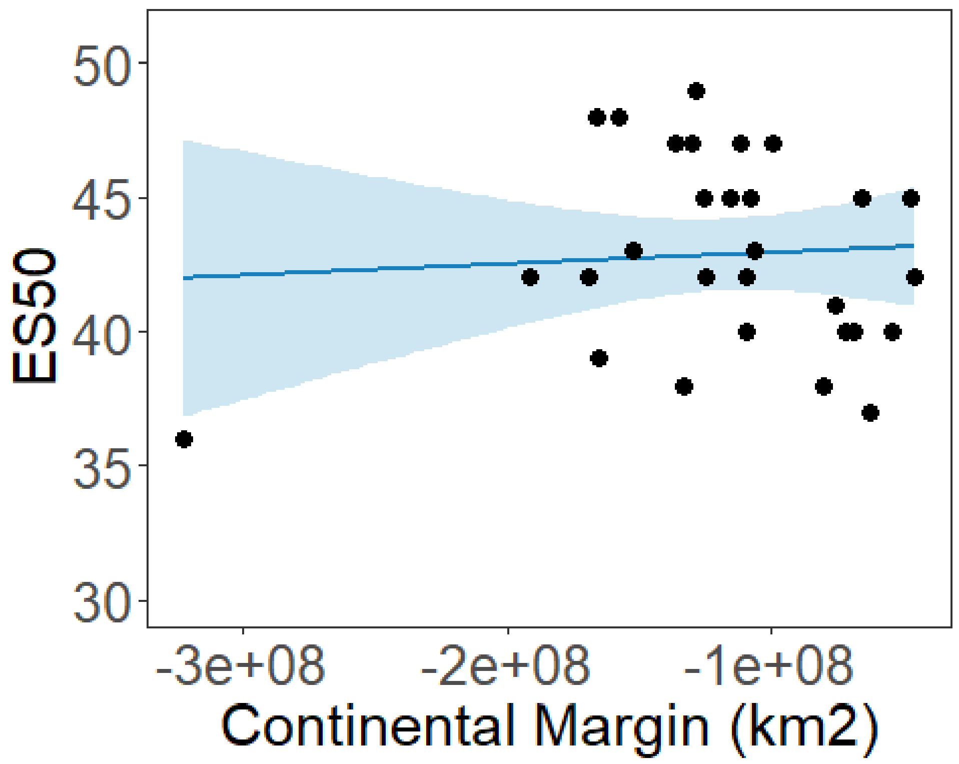


```r
ggplot(Ecological_Data_Global, aes(x = Depth_Mean, y = ES50_Dee)) +
  geom_smooth(method = "glm", formula = y ~ x, color = "#1a80bb", fill =
"#85bede") +  # Add a smooth dark blue line with light blue shadow
  geom_point(size = 3) +  # Add scatter plot points
  scale_x_continuous(labels = scales::scientific) +
  scale_y_continuous(limits=c(29, 52), breaks=seq(30, 50, by=5), expand =
c(0, 0)) +
  theme_bw() +  # Use the black and white theme
  labs(
    x = "Depth (m)",  # Shorten the x-axis title
    y = "ES50"  # Shorten the y-axis title
  ) +
  theme(
    panel.grid.minor = element_blank(),
    panel.grid.major = element_blank(),
    axis.text.x = element_text(size =20),  # Increase x-axis text size
    axis.text.y = element_text(size = 20),  # Increase y-axis text size
    axis.title.x = element_text(size = 22), # Increase x-axis title size
    axis.title.y = element_text(size = 22)  # Increase y-axis title size
  )
```

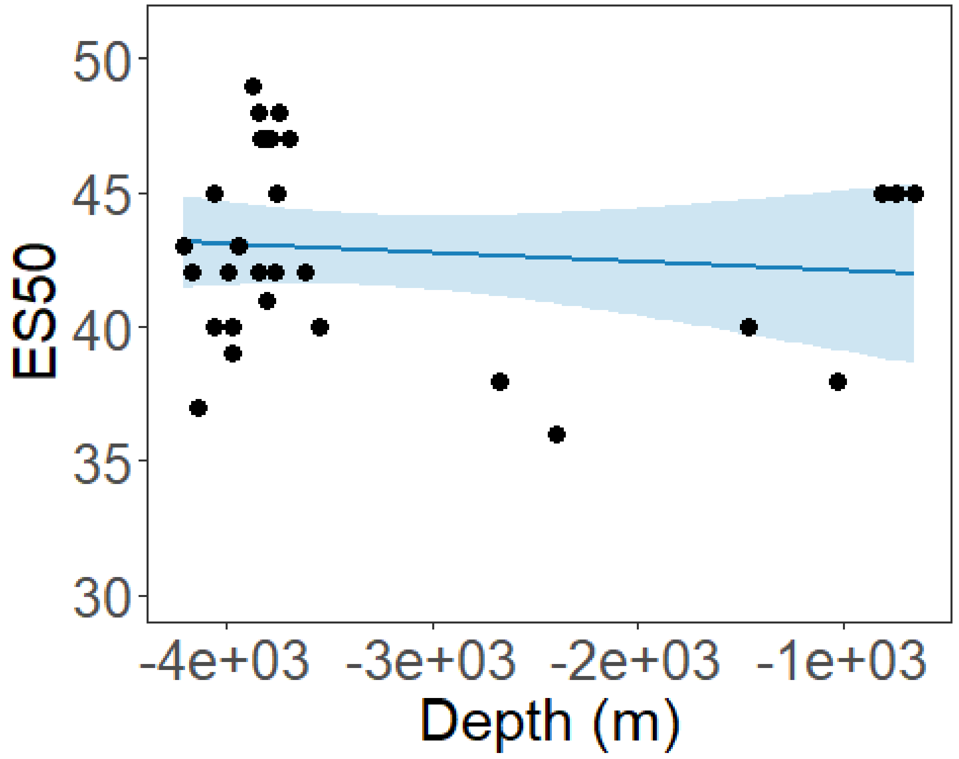


```
ggplot(Ecological_Data_Global, aes(x = HumImp_Mean, y = ES50_Dee)) +
  geom_smooth(method = "glm", formula = y ~ x, color = "#1a80bb", fill =
"#85bede") +  # Add a smooth dark blue line with light blue shadow
  geom_point(size = 3) +  # Add scatter plot points
  theme_bw() +  # Use the black and white theme
  scale_y_continuous(limits=c(29, 52), breaks=seq(30, 50, by=5), expand =
c(0, 0)) +
  labs(
    x = "Human Impact",  # Shorten the x-axis title
    y = "ES50"  # Shorten the y-axis title
  ) +
  theme(
    panel.grid.minor = element_blank(),
    panel.grid.major = element_blank(),
    axis.text.x = element_text(size =20),  # Increase x-axis text size
    axis.text.y = element_text(size = 20),  # Increase y-axis text size
    axis.title.x = element_text(size = 22), # Increase x-axis title size
    axis.title.y = element_text(size = 22)  # Increase y-axis title size
  )
```

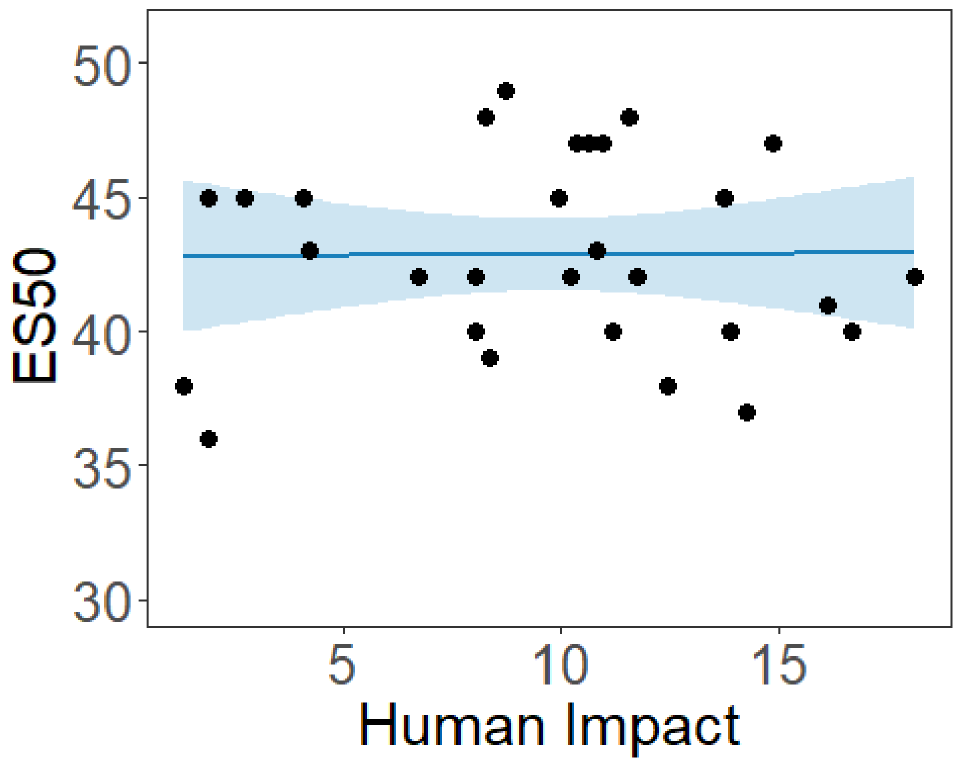


```
ggplot(Ecological_Data_Global, aes(x = CurVel_Bot_Mean, y = ES50_Dee)) +
  geom_smooth(method = "glm", formula = y ~ x, color = "#1a80bb", fill =
"#85bede") +  # Add a smooth dark blue line with light blue shadow
  geom_point(size = 3) +  # Add scatter plot points
  theme_bw() +  # Use the black and white theme
  scale_y_continuous(limits=c(29, 52), breaks=seq(30, 50, by=5), expand =
c(0, 0)) +
  labs(
    x = "Current Velocity (m.s-1)",  # Shorten the x-axis title
    y = "ES50"  # Shorten the y-axis title
  ) +
  theme(
    panel.grid.minor = element_blank(),
    panel.grid.major = element_blank(),
    axis.text.x = element_text(size =20),  # Increase x-axis text size
    axis.text.y = element_text(size = 20),  # Increase y-axis text size
    axis.title.x = element_text(size = 22), # Increase x-axis title size
    axis.title.y = element_text(size = 22)  # Increase y-axis title size
  )
```

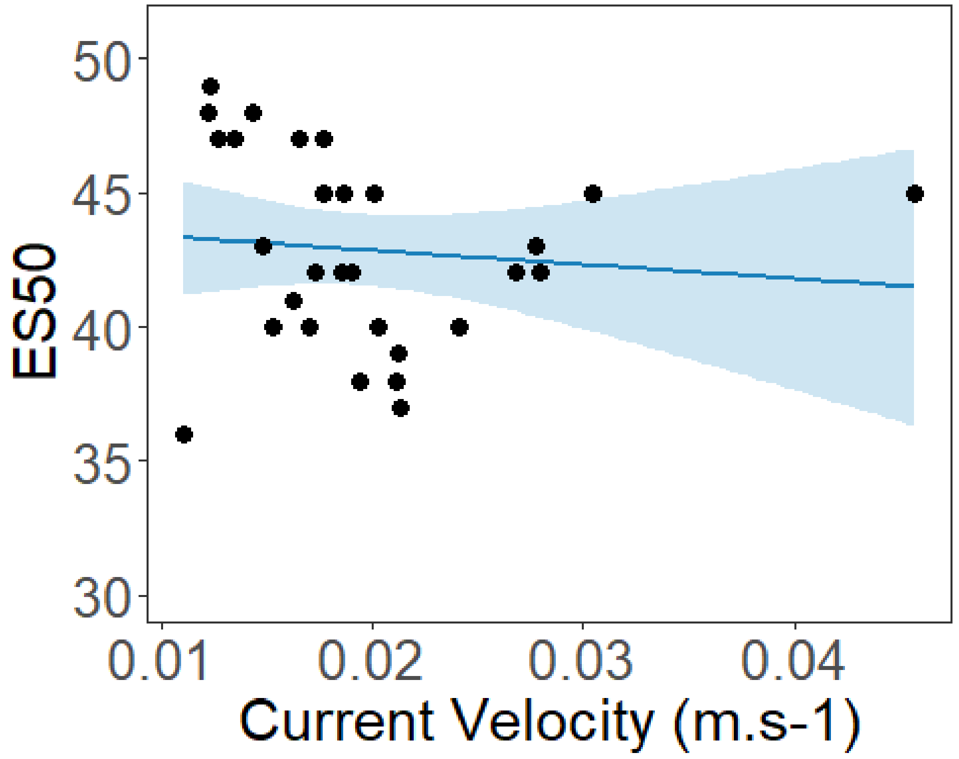


```r
ggplot(Ecological_Data_Global, aes(x = Nitrate_Bot_Mean, y = ES50_Dee)) +
  geom_smooth(method = "glm", formula = y ~ x, color = "#1a80bb", fill =
"#85bede") +  # Add a smooth dark blue line with light blue shadow
  geom_point(size = 3) +  # Add scatter plot points
  theme_bw() +  # Use the black and white theme
  scale_y_continuous(limits=c(29, 52), breaks=seq(30, 50, by=5), expand =
c(0, 0)) +
  labs(
    x = "Nitrate (mmol . m-3)",  # Shorten the x-axis title
    y = "ES50"  # Shorten the y-axis title
  ) +
  theme(
    panel.grid.minor = element_blank(),
    panel.grid.major = element_blank(),
    axis.text.x = element_text(size =20),  # Increase x-axis text size
    axis.text.y = element_text(size = 20),  # Increase y-axis text size
    axis.title.x = element_text(size = 22), # Increase x-axis title size
    axis.title.y = element_text(size = 22)  # Increase y-axis title size
  )
```

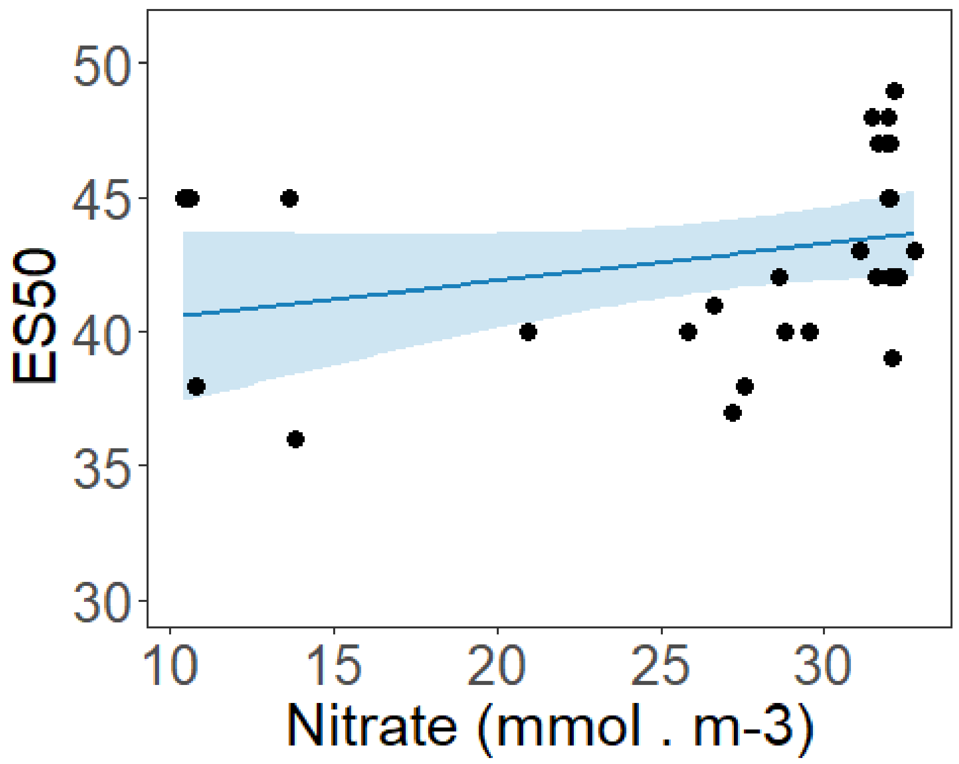


```
ggplot(Ecological_Data_Global, aes(x = O2_Bot_Mean, y = ES50_Dee)) +
  geom_smooth(method = "glm", formula = y ~ x, color = "#1a80bb", fill =
"#85bede") +  # Add a smooth dark blue line with light blue shadow
  geom_point(size = 3) +  # Add scatter plot points
  theme_bw() +  # Use the black and white theme
  scale_y_continuous(limits=c(29, 52), breaks=seq(30, 50, by=5), expand =
c(0, 0)) +
  labs(
    x = "O2 (mmol . m-3)",  # Shorten the x-axis title
    y = "ES50"  # Shorten the y-axis title
  ) +
  theme(
    panel.grid.minor = element_blank(),
    panel.grid.major = element_blank(),
    axis.text.x = element_text(size =20),  # Increase x-axis text size
    axis.text.y = element_text(size = 20),  # Increase y-axis text size
    axis.title.x = element_text(size = 22), # Increase x-axis title size
    axis.title.y = element_text(size = 22)  # Increase y-axis title size
  )
```

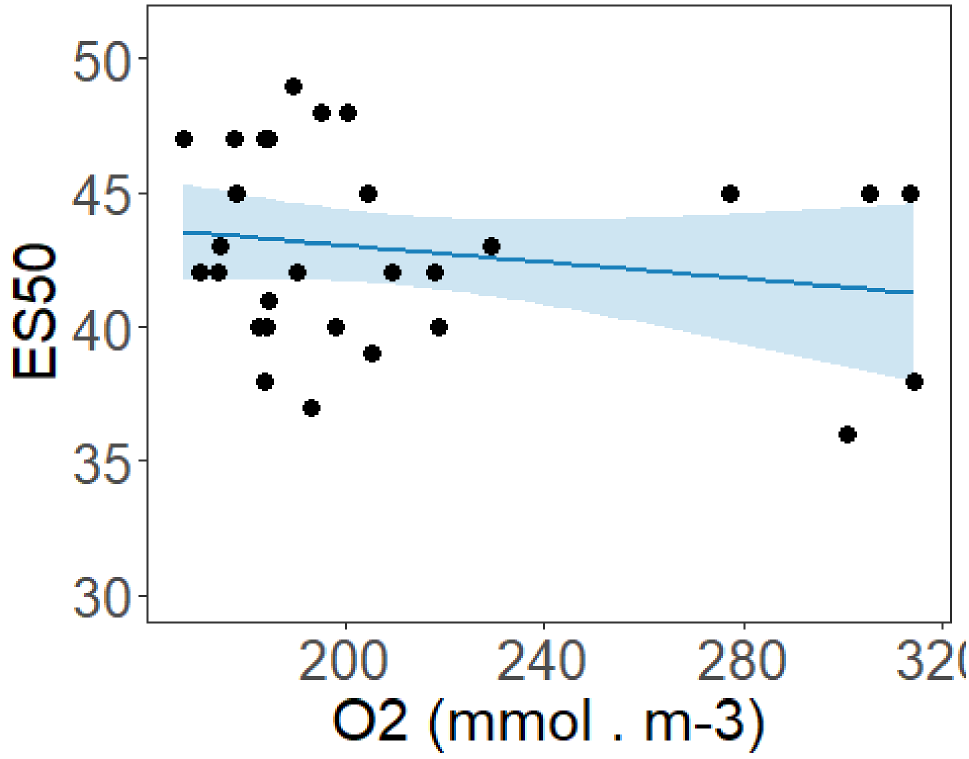


```
ggplot(Ecological_Data_Global, aes(x = Area_Sum, y = ES50_Dee)) +
  geom_smooth(method = "glm", formula = y ~ x, color = "#1a80bb", fill =
"#85bede") +  # Add a smooth dark blue line with light blue shadow
  geom_point(size = 3) +  # Add scatter plot points
  theme_bw() +  # Use the black and white theme
  scale_x_continuous(labels = scales::scientific) +
  scale_y_continuous(limits=c(29, 52), breaks=seq(30, 50, by=5), expand =
c(0, 0)) +
  labs(
    x = "Area (km2)",  # Shorten the x-axis title
    y = "ES50"  # Shorten the y-axis title
  ) +
  theme(
    panel.grid.minor = element_blank(),
    panel.grid.major = element_blank(),
    axis.text.x = element_text(size =20),  # Increase x-axis text size
    axis.text.y = element_text(size = 20),  # Increase y-axis text size
    axis.title.x = element_text(size = 22), # Increase x-axis title size
    axis.title.y = element_text(size = 22)  # Increase y-axis title size
  )
```

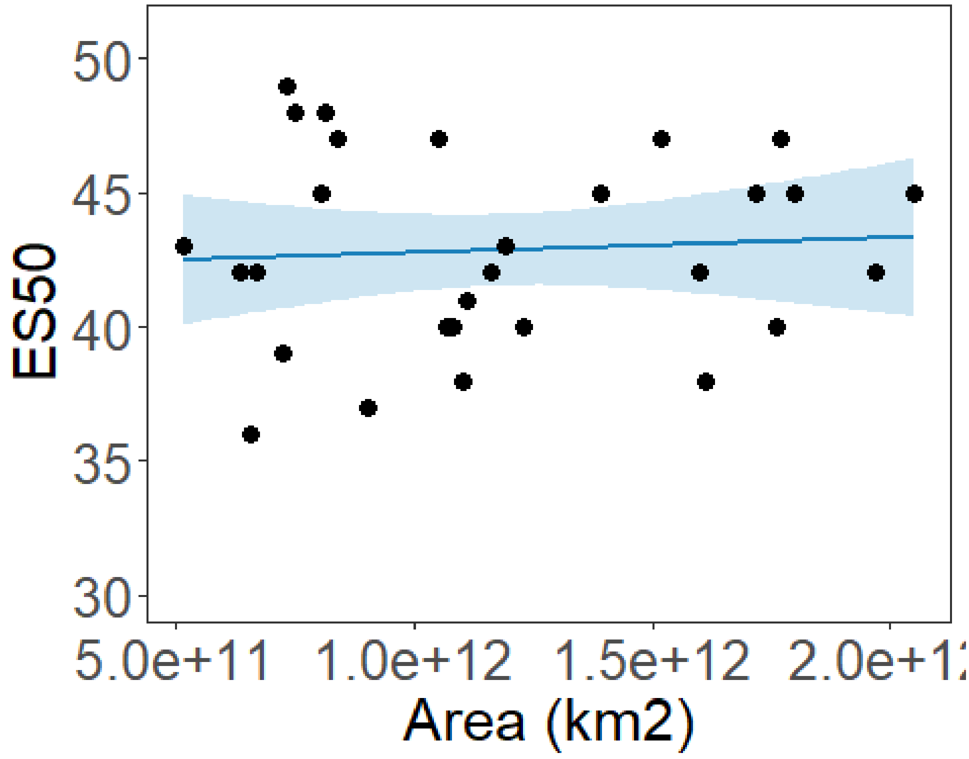


```
ggplot(Ecological_Data_Global, aes(x = PrimProd_Bot_Mean, y = ES50_Dee))
+
  geom_smooth(method = "glm", formula = y ~ x, color = "#1a80bb", fill =
"#85bede") +  # Add a smooth dark blue line with light blue shadow
  geom_point(size = 3) +  # Add scatter plot points
  theme_bw() +  # Use the black and white theme
  scale_y_continuous(limits=c(29, 52), breaks=seq(30, 50, by=5), expand =
c(0, 0)) +
  labs(
    x = "Primary Productivity (mmol . m-3)",  # Shorten the x-axis title
    y = "ES50"  # Shorten the y-axis title
  ) +
  theme(
    panel.grid.minor = element_blank(),
    panel.grid.major = element_blank(),
    axis.text.x = element_text(size =20),  # Increase x-axis text size
    axis.text.y = element_text(size = 20),  # Increase y-axis text size
    axis.title.x = element_text(size = 22), # Increase x-axis title size
    axis.title.y = element_text(size = 22)  # Increase y-axis title size
  )
```

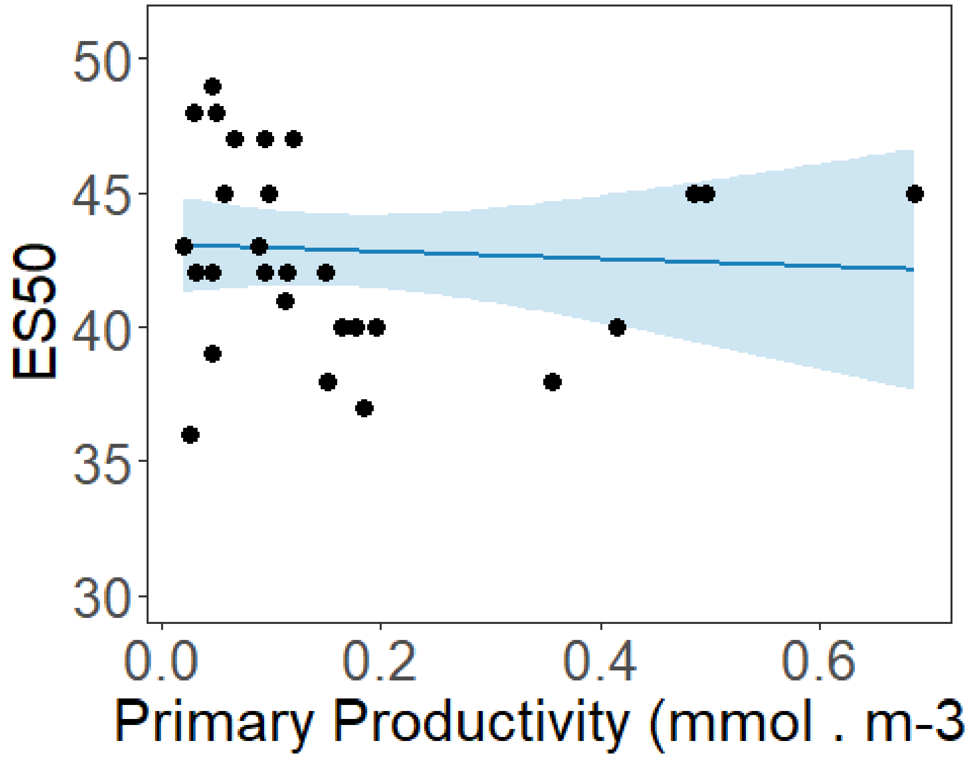


```
ggplot(Ecological_Data_Global, aes(x = ThemM_Bot_Mean, y = ES50_Dee)) +
  geom_smooth(method = "glm", formula = y ~ x, color = "#1a80bb", fill =
"#85bede") +  # Add a smooth dark blue line with light blue shadow
  geom_point(size = 3) +  # Add scatter plot points
  theme_bw() +  # Use the black and white theme
  scale_y_continuous(limits=c(29, 52), breaks=seq(30, 50, by=5), expand =
c(0, 0)) +
  labs(
    x = "Temperature Mean (ºC)",  # Shorten the x-axis title
    y = "ES50"  # Shorten the y-axis title
  ) +
  theme(
    panel.grid.minor = element_blank(),
    panel.grid.major = element_blank(),
    axis.text.x = element_text(size =20),  # Increase x-axis text size
    axis.text.y = element_text(size = 20),  # Increase y-axis text size
    axis.title.x = element_text(size = 22), # Increase x-axis title size
    axis.title.y = element_text(size = 22)  # Increase y-axis title size
  )
```

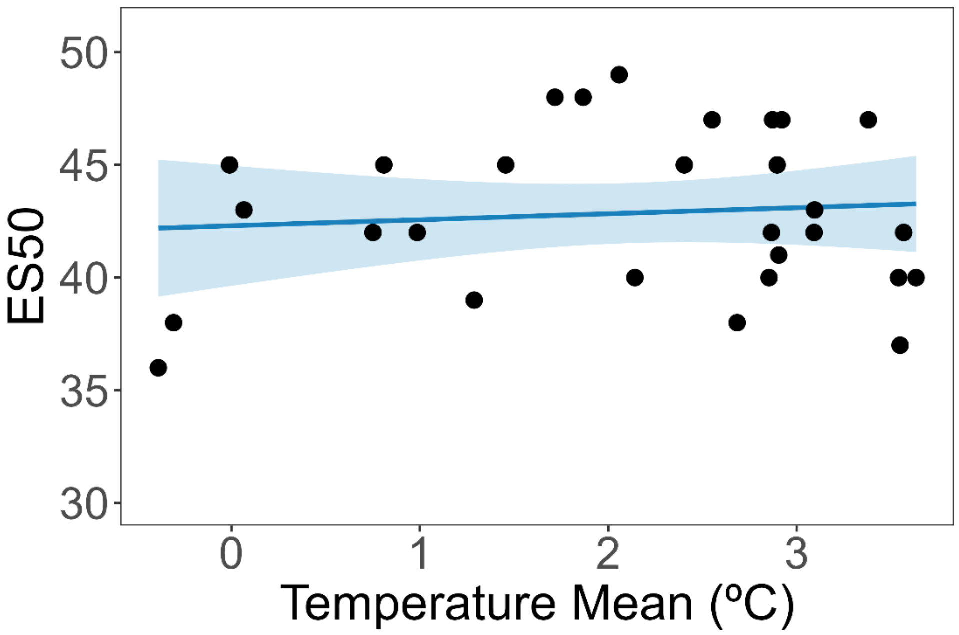

50
45
40
35
30
ES50
0
1
2
3
Temperature Mean (ºC)

Markdown_GAM_Shallow.R

hsaeedi

2025-06-08

```
# ===================================
#                 SHALLOW-NUMSPe-GAM
# ===================================
library(readxl)

## Warning: package 'readxl' was built under R version 4.3.3

library(openxlsx)

## Warning: package 'openxlsx' was built under R version 4.3.3

library(tidyverse)

## Warning: package 'tidyverse' was built under R version 4.3.3

## Warning: package 'ggplot2' was built under R version 4.3.3

## Warning: package 'tibble' was built under R version 4.3.3

## Warning: package 'tidyr' was built under R version 4.3.3

## Warning: package 'readr' was built under R version 4.3.3

## Warning: package 'purrr' was built under R version 4.3.3

## Warning: package 'dplyr' was built under R version 4.3.3

## Warning: package 'stringr' was built under R version 4.3.3

## Warning: package 'forcats' was built under R version 4.3.3

## Warning: package 'lubridate' was built under R version 4.3.3

## ── Attaching core tidyverse packages ──────────────────────── tidyvers
e 2.0.0 ──
## ✔ dplyr     1.1.4     ✔ readr     2.1.5
## ✔ forcats   1.0.0     ✔ stringr   1.5.1
## ✔ ggplot2   3.5.1     ✔ tibble    3.2.1
## ✔ lubridate 1.9.3     ✔ tidyr     1.3.1
## ✔ purrr     1.0.2
## ── Conflicts ────────────────────────────────────────── tidyverse_conf
licts() ──
## ✖ dplyr::filter() masks stats::filter()
## ✖ dplyr::lag()    masks stats::lag()
## ℹ Use the conflicted package (<http://conflicted.r-lib.org/>) to for
ce all conflicts to become errors

library(sf)
```

```
## Warning: package 'sf' was built under R version 4.3.3

## Linking to GEOS 3.11.2, GDAL 3.8.2, PROJ 9.3.1; sf_use_s2() is TRUE

library(vegan)

## Warning: package 'vegan' was built under R version 4.3.3

## Loading required package: permute

## Warning: package 'permute' was built under R version 4.3.3

## Loading required package: lattice
## This is vegan 2.6-4

library(pvclust)

## Warning: package 'pvclust' was built under R version 4.3.3

library(dplyr)
library(ggplot2)
library(robis)

## Warning: package 'robis' was built under R version 4.3.3

library(obistools)
library(nortest) # for Anderson-Darling test
library(stringi) # for encoding UTF-8

## Warning: package 'stringi' was built under R version 4.3.3

library(corrplot)

## Warning: package 'corrplot' was built under R version 4.3.3

## corrplot 0.94 loaded

library(mgcv)

## Loading required package: nlme
##
## Attaching package: 'nlme'
##
## The following object is masked from 'package:dplyr':
##
##     collapse
##
## This is mgcv 1.9-0. For overview type 'help("mgcv-package")'.

library(ggeffects)

## Warning: package 'ggeffects' was built under R version 4.3.3

library(DHARMa) #simulations package gam-

## Warning: package 'DHARMa' was built under R version 4.3.3
```

```
## Warning in check_dep_version(): ABI version mismatch:
## lme4 was built with Matrix ABI version 1
## Current Matrix ABI version is 0
## Please re-install lme4 from source or restore original 'Matrix' packag
e

## This is DHARMa 0.4.6. For overview type '?DHARMa'. For recent changes,
type news(package = 'DHARMa')

library(knitr)

## Warning: package 'knitr' was built under R version 4.3.3

library(qpcR)

## Warning: package 'qpcR' was built under R version 4.3.3

## Loading required package: MASS
##
## Attaching package: 'MASS'
##
## The following object is masked from 'package:robis':
##
##     area
##
## The following object is masked from 'package:dplyr':
##
##     select
##
## Loading required package: minpack.lm

## Warning: package 'minpack.lm' was built under R version 4.3.3

## Loading required package: rgl

## Warning: package 'rgl' was built under R version 4.3.3

## Loading required package: robustbase

## Warning: package 'robustbase' was built under R version 4.3.3

## Loading required package: Matrix
##
## Attaching package: 'Matrix'
##
## The following objects are masked from 'package:tidyr':
##
##     expand, pack, unpack

#Species Counts and Environment, hexagons
Ecological_Data_Global_Hex_sp <- read.csv("Ecological_Data_Global_Hex_sp.
csv", sep = ";")

summary(Ecological_Data_Global_Hex_sp)
```

```
##        X               ID            Latitude        Longitude
##  Min.   :  1.0   Min.   :  1.0   Min.   :-87.63   Min.   :-174.45982
##  1st Qu.:161.2   1st Qu.:161.2   1st Qu.:-29.98   1st Qu.: -86.99765
##  Median :321.5   Median :321.5   Median :  0.00   Median :   0.21022
##  Mean   :321.5   Mean   :321.5   Mean   :  0.00   Mean   :  -0.08773
##  3rd Qu.:481.8   3rd Qu.:481.8   3rd Qu.: 29.98   3rd Qu.:  87.29992
##  Max.   :642.0   Max.   :642.0   Max.   : 87.63   Max.   : 174.30184
##
##     Area_Sum        Depth_Mean       Depth_Median       Depth_Max
##  Min.   :661589   Min.   :-5660.6   Min.   :-5842.0   Min.   :-4502.0
##  1st Qu.:794383   1st Qu.:-4455.8   1st Qu.:-4680.0   1st Qu.: -672.0
##  Median :796851   Median :-3642.6   Median :-3818.0   Median :   42.0
##  Mean   :794623   Mean   :-3172.3   Mean   :-3273.1   Mean   : -267.8
##  3rd Qu.:799061   3rd Qu.:-2122.7   3rd Qu.:-2158.0   3rd Qu.:  383.0
##  Max.   :803949   Max.   :   35.5   Max.   :   14.5   Max.   : 2876.0
##                   NA's   :81        NA's   :81        NA's   :81
##     Chl_Mean         Chl_Median        Margin_Sum          Margin_Mean
##  Min.   :0.04837   Min.   :0.04746   Min.   :-195640765   Min.   :-288
3.58
##  1st Qu.:0.11386   1st Qu.:0.10239   1st Qu.:  -7955299   1st Qu.: -93
0.65
##  Median :0.22296   Median :0.19813   Median :  -2449082   Median : -42
8.88
##  Mean   :0.29174   Mean   :0.26079   Mean   :  -6386344   Mean   : -59
9.64
##  3rd Qu.:0.35835   3rd Qu.:0.33879   3rd Qu.:    -87662   3rd Qu.:  -6
2.06
##  Max.   :4.71631   Max.   :4.71268   Max.   :         0   Max.   :
0.00
##  NA's   :82        NA's   :82                             NA's   :81
##  Margin_Median      Shelf_Sum          Shelf_Mean        Shelf_Median
##  Min.   :-3148.0   Min.   :-2566468   Min.   :-55.6837   Min.   :-56.0
00
##  1st Qu.:    0.0   1st Qu.:  -66625   1st Qu.: -5.4761   1st Qu.:  0.0
00
##  Median :    0.0   Median :    -545   Median : -0.1858   Median :  0.0
00
##  Mean   : -325.7   Mean   : -104973   Mean   : -5.5298   Mean   : -2.2
34
##  3rd Qu.:    0.0   3rd Qu.:       0   3rd Qu.:  0.0000   3rd Qu.:  0.0
00
##  Max.   :    0.0   Max.   :       0   Max.   :  0.0000   Max.   :  0.0
00
##  NA's   :81                           NA's   :81         NA's   :81
##   CurVel_Mean       CurVel_Median     CurVel_Bot_Mean   CurVel_Bot_Medi
an
##  Min.   :0.00712   Min.   :0.00498   Min.   :0.00088   Min.   :0.00049
##  1st Qu.:0.04354   1st Qu.:0.03687   1st Qu.:0.00957   1st Qu.:0.00647
##  Median :0.06934   Median :0.05959   Median :0.01521   Median :0.01016
##  Mean   :0.09485   Mean   :0.08466   Mean   :0.01963   Mean   :0.01365
##  3rd Qu.:0.12765   3rd Qu.:0.11084   3rd Qu.:0.02423   3rd Qu.:0.01650
```

```
##  Max.   :0.51113   Max.   :0.46702   Max.   :0.16873   Max.   :0.16081
##  NA's   :82        NA's   :82        NA's   :82        NA's   :82
##  Diff_Atte_Mean    Diff_Atte_Median    HumImp_Sum        HumImp_Mean
##  Min.   :0.02839   Min.   :0.02586   Min.   :       0   Min.   : 0.000
##  1st Qu.:0.05826   1st Qu.:0.05759   1st Qu.:       0   1st Qu.: 5.361
##  Median :0.06980   Median :0.06815   Median :    5028   Median :12.000
##  Mean   :0.08042   Mean   :0.07798   Mean   : 3518207   Mean   :12.467
##  3rd Qu.:0.08694   3rd Qu.:0.08342   3rd Qu.: 4649304   3rd Qu.:18.055
##  Max.   :0.39725   Max.   :0.43915   Max.   :37425783   Max.   :38.554
##  NA's   :82        NA's   :82        NA's   :19         NA's   :297
##  HumImp_Median   Ice_Cover_Sum     IceCov_Mean       Ice_Cover_Median
##  Min.   : 0.00   Min.   :      0   Min.   :0.00000   Min.   :0.00000
##  1st Qu.: 4.00   1st Qu.:      0   1st Qu.:0.00000   1st Qu.:0.00000
##  Median :11.00   Median :      0   Median :0.00000   Median :0.00000
##  Mean   :11.43   Mean   :   4551   Mean   :0.07398   Mean   :0.07278
##  3rd Qu.:18.00   3rd Qu.:      0   3rd Qu.:0.00000   3rd Qu.:0.00000
##  Max.   :42.00   Max.   :603554   Max.   :0.96853   Max.   :0.96977
##  NA's   :297                      NA's   :82        NA's   :82
##  Ice_Tick_Mean    Ice_Tick_Median   MixLay_Mean      MixLay_Median
##  Min.   :0.0000   Min.   :0.0000   Min.   :  7.592   Min.   :  7.019
##  1st Qu.:0.0000   1st Qu.:0.0000   1st Qu.: 21.977   1st Qu.: 22.198
##  Median :0.0000   Median :0.0000   Median : 34.135   Median : 34.273
##  Mean   :0.1148   Mean   :0.1115   Mean   : 39.535   Mean   : 39.294
##  3rd Qu.:0.0000   3rd Qu.:0.0000   3rd Qu.: 47.630   3rd Qu.: 46.981
##  Max.   :3.1516   Max.   :3.5327   Max.   :177.253   Max.   :189.459
##  NA's   :82       NA's   :82       NA's   :82        NA's   :82
##   Nitrate_Mean      Nitrate_Median     Nitrate_Bot_Mean   Nitrate_Bot_
Median
##  Min.   : 0.00054   Min.   : 0.00045   Min.   : 0.04162   Min.   : 0.0
0
##  1st Qu.: 0.04998   1st Qu.: 0.01156   1st Qu.:22.14293   1st Qu.:22.6
4
##  Median : 1.14055   Median : 0.84186   Median :32.52189   Median :32.9
5
##  Mean   : 5.61826   Mean   : 5.53985   Mean   :27.47784   Mean   :28.0
7
##  3rd Qu.: 6.45838   3rd Qu.: 6.46006   3rd Qu.:34.88484   3rd Qu.:35.5
1
##  Max.   :33.91337   Max.   :31.22474   Max.   :39.54847   Max.   :40.1
0
##  NA's   :82         NA's   :82         NA's   :82         NA's   :82
##     O2_Mean         O2_Median      O2_Bot_Mean      O2_Bot_Median
##  Min.   :195.1   Min.   :195.2   Min.   :  3.051   Min.   :  0.4178
##  1st Qu.:206.2   1st Qu.:206.1   1st Qu.:164.353   1st Qu.:164.9396
##  Median :226.9   Median :226.0   Median :199.301   Median :202.8894
##  Mean   :250.8   Mean   :250.4   Mean   :200.601   Mean   :201.8704
##  3rd Qu.:294.9   3rd Qu.:292.7   3rd Qu.:234.898   3rd Qu.:237.5830
##  Max.   :390.2   Max.   :392.5   Max.   :389.954   Max.   :390.7931
##  NA's   :82      NA's   :82      NA's   :82        NA's   :82
##  PhotoActi_Mean   PhotoActi_Median PrimProd_Bot_Mean PrimProd_Bot_Medi
an
```

```
##  Min.   : 3.864   Min.   : 3.642   Min.   :0.01355   Min.   :0.01355  
##  1st Qu.:27.254   1st Qu.:27.229   1st Qu.:0.01355   1st Qu.:0.01355  
##  Median :39.860   Median :39.993   Median :0.01707   Median :0.01355  
##  Mean   :36.778   Mean   :36.811   Mean   :0.28762   Mean   :0.18279  
##  3rd Qu.:45.157   3rd Qu.:45.163   3rd Qu.:0.12868   3rd Qu.:0.01447  
##  Max.   :51.482   Max.   :52.344   Max.   :9.24785   Max.   :8.02454  
##  NA's   :82       NA's   :82       NA's   :82        NA's   :82       
##  PrimProd_Mean     PrimProd_median   Salinity_Bot_mean Salinity_Bot_me
dian
##  Min.   : 0.3348   Min.   : 0.3118   Min.   : 4.727    Min.   : 4.103  
##  1st Qu.: 1.2183   1st Qu.: 1.2000   1st Qu.:34.661    1st Qu.:34.680  
##  Median : 1.4959   Median : 1.4421   Median :34.696    Median :34.703  
##  Mean   : 1.7349   Mean   : 1.6125   Mean   :34.304    Mean   :34.400  
##  3rd Qu.: 1.7421   3rd Qu.: 1.6362   3rd Qu.:34.760    3rd Qu.:34.761  
##  Max.   :17.4345   Max.   :17.4302   Max.   :40.231    Max.   :40.600  
##  NA's   :82        NA's   :82        NA's   :82        NA's   :82      
##  Salinity_mean    Salinity_median  Silicate_mean      Silicate_median  
##  Min.   : 3.239   Min.   : 2.786   Min.   :  0.7463   Min.   :  0.7133  
##  1st Qu.:33.879   1st Qu.:33.888   1st Qu.:  1.6572   1st Qu.:  1.5791  
##  Median :34.534   Median :34.566   Median :  2.6372   Median :  2.4321  
##  Mean   :33.996   Mean   :34.123   Mean   : 11.1758   Mean   : 10.8693  
##  3rd Qu.:35.403   3rd Qu.:35.450   3rd Qu.: 10.9034   3rd Qu.:  8.9543  
##  Max.   :38.992   Max.   :39.180   Max.   :116.4004   Max.   :118.7041  
##  NA's   :82       NA's   :82       NA's   :82         NA's   :82        
##    ThemM_mean      ThemM_median     ThemM_Bot_mean    ThemM_Bot_median 
##  Min.   :-1.794   Min.   :-1.808   Min.   :-1.7009   Min.   :-1.7534  
##  1st Qu.: 9.333   1st Qu.: 9.049   1st Qu.: 0.8558   1st Qu.: 0.7385  
##  Median :21.327   Median :21.407   Median : 1.3503   Median : 1.1530  
##  Mean   :17.837   Mean   :17.863   Mean   : 3.3903   Mean   : 2.8933  
##  3rd Qu.:26.986   3rd Qu.:27.043   3rd Qu.: 3.1400   3rd Qu.: 1.9769  
##  Max.   :29.712   Max.   :29.737   Max.   :28.4978   Max.   :28.7753  
##  NA's   :82       NA's   :82       NA's   :82        NA's   :82       
##  Them_Max_mean    Them_Max_median   Them_Max_max      Them_Min_mean   
##  Min.   :-1.555   Min.   :-1.594   Min.   :-0.9619   Min.   :-1.981  
##  1st Qu.:14.674   1st Qu.:14.537   1st Qu.:19.2728   1st Qu.: 5.467  
##  Median :25.932   Median :25.868   Median :27.9112   Median :16.985  
##  Mean   :21.550   Mean   :21.548   Mean   :23.8339   Mean   :14.700  
##  3rd Qu.:29.399   3rd Qu.:29.409   3rd Qu.:30.3046   3rd Qu.:24.027  
##  Max.   :34.692   Max.   :34.669   Max.   :36.4851   Max.   :28.633  
##  NA's   :82       NA's   :82       NA's   :82        NA's   :82      
##  Them_Min_median   Them_Min_min       ThemR_mean       ThemR_median   
##  Min.   :-2.000   Min.   :-2.0000   Min.   : 0.226   Min.   : 0.1985  
##  1st Qu.: 5.339   1st Qu.: 0.6541   1st Qu.: 4.119   1st Qu.: 4.0608  
##  Median :17.097   Median :13.5745   Median : 6.166   Median : 6.0265  
##  Mean   :14.740   Mean   :12.1140   Mean   : 6.851   Mean   : 6.7578  
##  3rd Qu.:24.112   3rd Qu.:21.4512   3rd Qu.: 8.341   3rd Qu.: 8.3701  
##  Max.   :28.836   Max.   :27.4934   Max.   :25.406   Max.   :25.4389  
##  NA's   :82       NA's   :82        NA's   :82       NA's   :82       
##   ThemR_range       ThemR_Bot_mean      ThemR_Bot_median   ThemR_Bot_ra
nge
##  Min.   : 0.09749   Min.   : 0.01941   Min.   : 0.01558   Min.   : 0.0
```

```
5681
##  1st Qu.: 2.75496   1st Qu.: 0.10560   1st Qu.: 0.07944   1st Qu.: 1.1
2864
##  Median : 4.03592   Median : 0.35621   Median : 0.17431   Median : 5.3
0821
##  Mean   : 4.82962   Mean   : 1.34412   Mean   : 1.01748   Mean   : 6.8
4583
##  3rd Qu.: 6.17135   3rd Qu.: 1.13137   3rd Qu.: 0.51880   3rd Qu.:11.0
6246
##  Max.   :20.18199   Max.   :22.02747   Max.   :21.85832   Max.   :28.6
5922
##  NA's   :82         NA's   :82         NA's   :82         NA's   :82
##    NumRec_sha        NumSpe_sha          ES50_sha       Chao1_sha
##  Min.   :      2   Min.   :    2.00   Min.   : 3.40   Min.   :    5.0
##  1st Qu.:    657   1st Qu.:   88.25   1st Qu.:25.33   1st Qu.:  156.0
##  Median :   4034   Median :  278.00   Median :34.11   Median :  472.5
##  Mean   :  67514   Mean   :  939.73   Mean   :33.11   Mean   : 1414.0
##  3rd Qu.:  21699   3rd Qu.:  998.00   3rd Qu.:42.24   3rd Qu.: 1648.5
##  Max.   :6258830   Max.   :11419.00   Max.   :52.46   Max.   :13882.3
##  NA's   :80        NA's   :80         NA's   :102     NA's   :102
##     ACE_sha            Weighted_sha       NumRec_mes         NumSpe_mes
##  Min.   :    6.111   Min.   :  1.669   Min.   :     1.0   Min.   :   1
.00
##  1st Qu.:  147.383   1st Qu.: 14.734   1st Qu.:    71.8   1st Qu.:  32
.75
##  Median :  465.805   Median : 33.018   Median :   388.0   Median : 108
.50
##  Mean   : 1453.239   Mean   : 66.817   Mean   :  7464.8   Mean   : 325
.99
##  3rd Qu.: 1716.280   3rd Qu.: 68.502   3rd Qu.:  1907.0   3rd Qu.: 371
.75
##  Max.   :16530.276   Max.   :774.467   Max.   :809096.0   Max.   :3809
.00
##  NA's   :104         NA's   :102       NA's   :110        NA's   :110
##     ES50_mes        Chao1_mes         ACE_mes        Weighted_mes
##  Min.   : 8.561   Min.   :   9.0   Min.   :   9.0   Min.   :  3.076
##  1st Qu.:29.228   1st Qu.: 110.8   1st Qu.: 114.3   1st Qu.: 23.652
##  Median :36.387   Median : 302.3   Median : 305.9   Median : 48.663
##  Mean   :35.088   Mean   : 641.1   Mean   : 638.6   Mean   : 80.441
##  3rd Qu.:43.333   3rd Qu.: 789.7   3rd Qu.: 788.8   3rd Qu.:106.010
##  Max.   :50.000   Max.   :9453.0   Max.   :5423.8   Max.   :533.774
##  NA's   :217      NA's   :217      NA's   :219      NA's   :217
##    NumRec_dee         NumSpe_dee          ES50_dee        Chao1_dee
##  Min.   :     1.0   Min.   :   1.0   Min.   : 1.049   Min.   :   2.0
##  1st Qu.:    85.5   1st Qu.:  51.5   1st Qu.:32.574   1st Qu.: 189.5
##  Median :   509.0   Median : 161.0   Median :38.851   Median : 393.9
##  Mean   :  5125.1   Mean   : 382.0   Mean   :37.584   Mean   : 712.7
##  3rd Qu.:  2261.5   3rd Qu.: 435.0   3rd Qu.:44.250   3rd Qu.: 895.7
##  Max.   :512758.0   Max.   :5204.0   Max.   :50.000   Max.   :6899.6
##  NA's   :111        NA's   :111      NA's   :200      NA's   :200
##     ACE_dee          Weighted_dee
```

```
##  Min.   :  27.62   Min.   :  1.002
##  1st Qu.: 210.68   1st Qu.: 29.434
##  Median : 431.06   Median : 57.897
##  Mean   : 742.80   Mean   : 92.117
##  3rd Qu.: 931.61   3rd Qu.:123.713
##  Max.   :7151.71   Max.   :544.308
##  NA's   :203       NA's   :200

#Correlation analyses for variables
#select only numeric values
numeric_data <- Ecological_Data_Global_Hex_sp %>% select_if(is.numeric)

#select for all columns
correlation_matrix <- cor(numeric_data, use = "complete.obs", method = "s
pearman")

#First we're going to load in our data and then trim the data frame down
to just the columns we need.
analysis.cols <- c("Latitude", "Longitude", "Depth_Mean", "Shelf_Mean", "
CurVel_Mean", "HumImp_Mean", "IceCov_Mean",
                   "Nitrate_Mean", "PrimProd_Mean", "ThemM_mean", "ThemR_
mean", "NumRec_sha", "NumSpe_sha", "ES50_sha")
Ecological_Data_Global_Hex_sp <- Ecological_Data_Global_Hex_sp [,analysis
.cols]
Ecological_Data_Global_Hex_sp  <- Ecological_Data_Global_Hex_sp [complete
.cases(Ecological_Data_Global_Hex_sp),]

# Calculate the correlation matrix
corr_matrix <- cor(Ecological_Data_Global_Hex_sp)

# Create the correlation plot with black font for text
corrplot(corr_matrix, tl.col = "black")
```

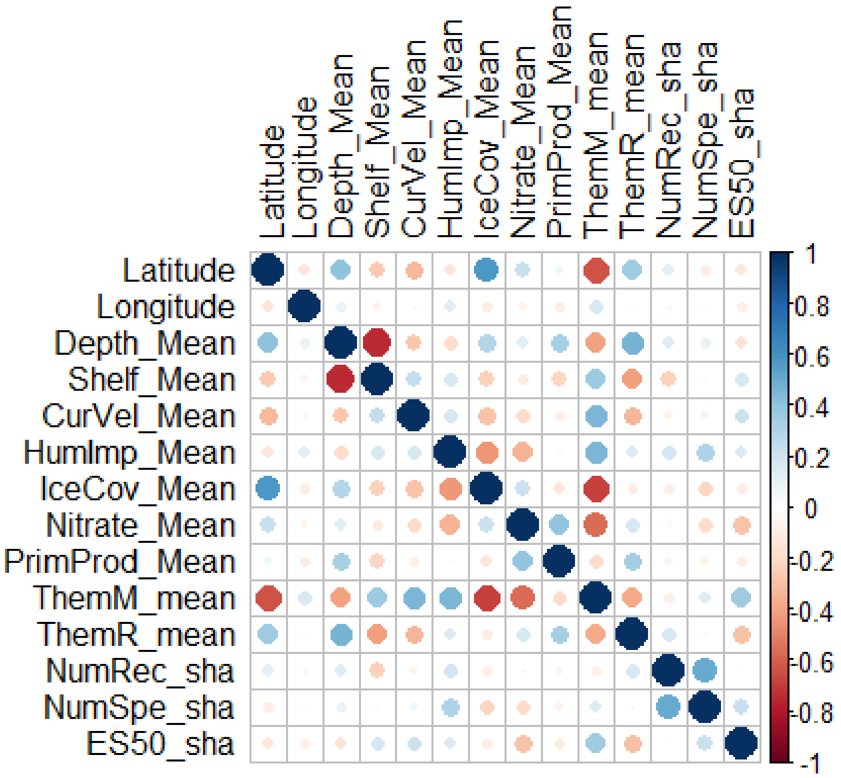


```r
#GAMs for number of species, shallow water
shallow.numsp.intercept <- gam(NumSpe_sha ~ 1, data = Ecological_Data_Glo
bal_Hex_sp, family = "nb", method = "REML", select = TRUE)
gam.check(shallow.numsp.intercept)
```

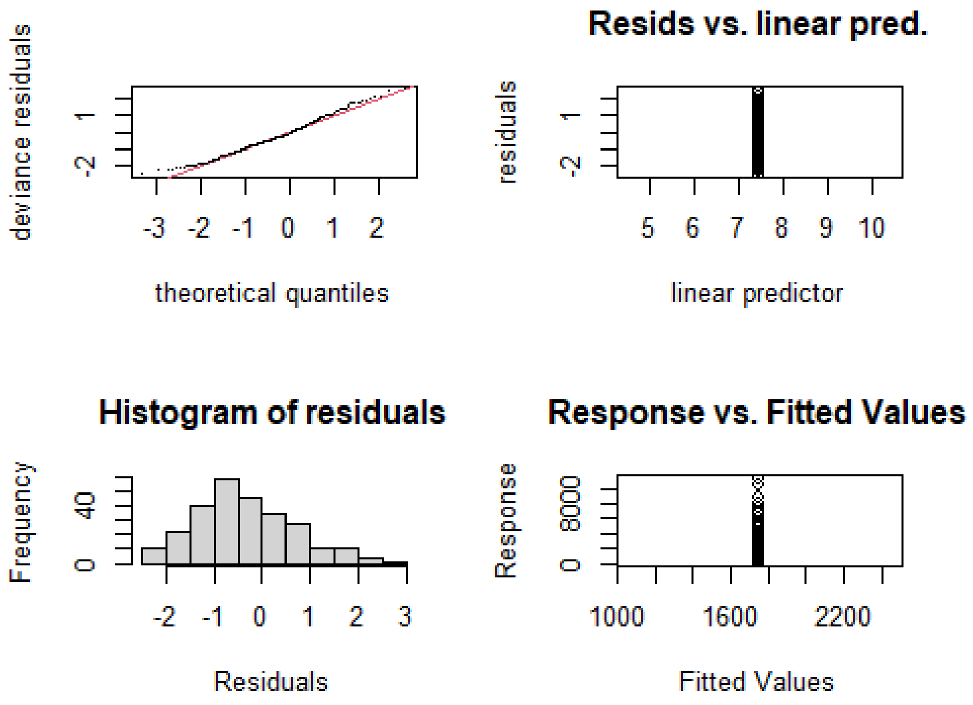


```
##
## Method: REML   Optimizer: outer newton
## full convergence after 3 iterations.
## Gradient range [1.138409e-08,1.138409e-08]
```

```
## (score 2236.673 & scale 1).
## Hessian positive definite, eigenvalue range [172.9693,172.9693].
## Model rank =  1 / 1

summary(shallow.numsp.intercept)

##
## Family: Negative Binomial(0.888)
## Link function: log
##
## Formula:
## NumSpe_sha ~ 1
##
## Parametric coefficients:
##             Estimate Std. Error z value Pr(>|z|)
## (Intercept)  7.46962    0.06531   114.4   <2e-16 ***
## ---
## Signif. codes:  0 '***' 0.001 '**' 0.01 '*' 0.05 '.' 0.1 ' ' 1
##
##
## R-sq.(adj) =      0   Deviance explained = 2.2e-08%
## -REML = 2236.7  Scale est. = 1         n = 264

shallow.numsp.latlon <- gam(NumSpe_sha ~ s(Latitude, Longitude, bs = "sos
") + s(NumRec_sha), data = Ecological_Data_Global_Hex_sp, family = "nb",
method = "REML", select = TRUE)
gam.check(shallow.numsp.latlon)
```

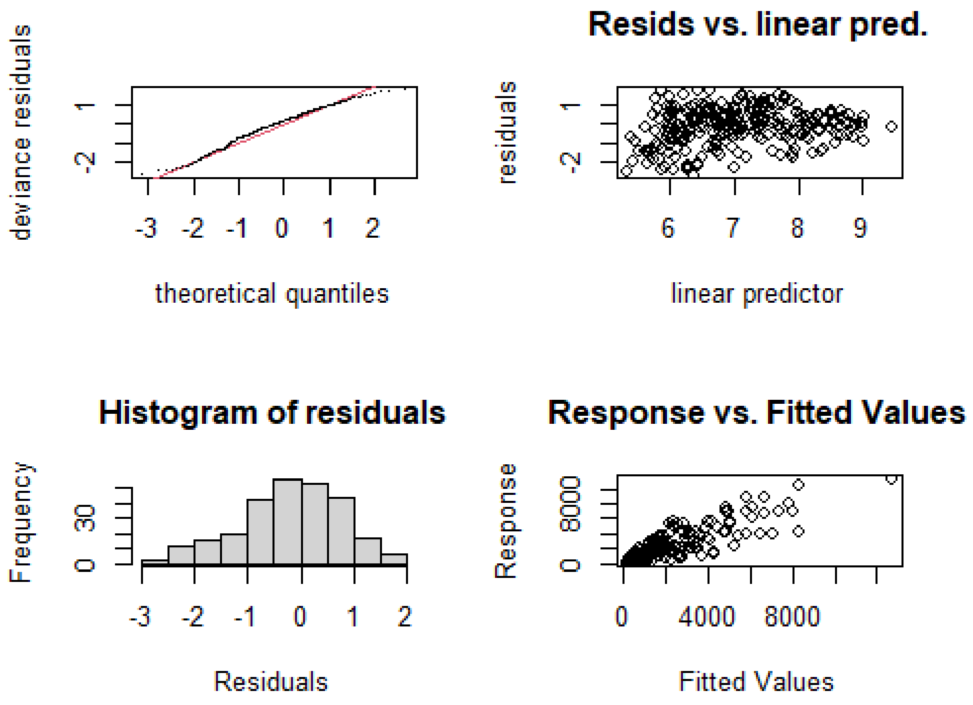


```
##
## Method: REML   Optimizer: outer newton
## full convergence after 8 iterations.
```

```
## Gradient range [-3.809351e-09,5.556966e-08]
## (score 2145.474 & scale 1).
## Hessian positive definite, eigenvalue range [0.474053,122.6063].
## Model rank =  59 / 59
##
## Basis dimension (k) checking results. Low p-value (k-index<1) may
## indicate that k is too low, especially if edf is close to k'.
##
##                             k'   edf k-index p-value
## s(Latitude,Longitude) 49.00 28.76    0.94    0.35
## s(NumRec_sha)          9.00  8.37    0.58  <2e-16 ***
## ---
## Signif. codes:  0 '***' 0.001 '**' 0.01 '*' 0.05 '.' 0.1 ' ' 1

summary(shallow.numsp.latlon)

##
## Family: Negative Binomial(2.24)
## Link function: log
##
## Formula:
## NumSpe_sha ~ s(Latitude, Longitude, bs = "sos") + s(NumRec_sha)
##
## Parametric coefficients:
##             Estimate Std. Error z value Pr(>|z|)
## (Intercept)  7.02156    0.04118   170.5   <2e-16 ***
## ---
## Signif. codes:  0 '***' 0.001 '**' 0.01 '*' 0.05 '.' 0.1 ' ' 1
##
## Approximate significance of smooth terms:
##                             edf Ref.df Chi.sq p-value
## s(Latitude,Longitude) 28.765     49  158.5  <2e-16 ***
## s(NumRec_sha)          8.373      9  161.6  <2e-16 ***
## ---
## Signif. codes:  0 '***' 0.001 '**' 0.01 '*' 0.05 '.' 0.1 ' ' 1
##
## R-sq.(adj) =  0.713   Deviance explained = 68.3%
## -REML = 2145.5  Scale est. = 1         n = 264

shallow.numsp.depth <- gam(NumSpe_sha ~ s(Latitude, Longitude, bs = "sos"
) + s(NumRec_sha) + s(Depth_Mean), data = Ecological_Data_Global_Hex_sp,
family = "nb", method = "REML", select = TRUE)
gam.check(shallow.numsp.depth)
```

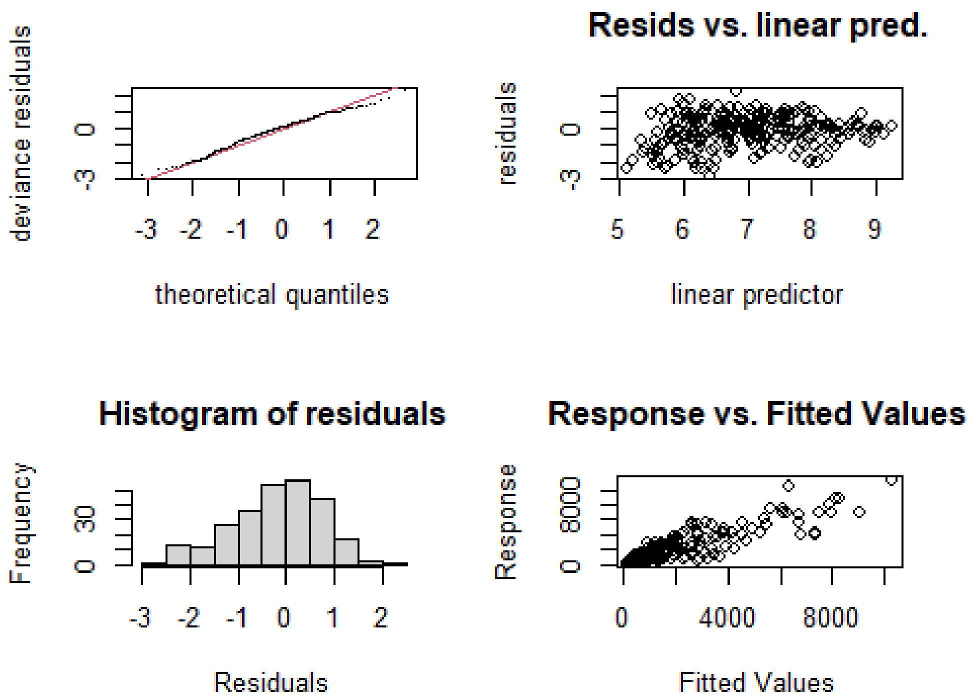


```
##
## Method: REML   Optimizer: outer newton
## full convergence after 8 iterations.
## Gradient range [-0.0002060021,0.0006577855]
## (score 2132.592 & scale 1).
## Hessian positive definite, eigenvalue range [0.0001121551,121.0075].
## Model rank =  68 / 68
##
## Basis dimension (k) checking results. Low p-value (k-index<1) may
## indicate that k is too low, especially if edf is close to k'.
##
##                             k'   edf k-index p-value
## s(Latitude,Longitude) 49.00 26.44    0.97    0.51
## s(NumRec_sha)          9.00  8.29    0.57  <2e-16 ***
## s(Depth_Mean)          9.00  2.87    0.93    0.26
## ---
## Signif. codes:  0 '***' 0.001 '**' 0.01 '*' 0.05 '.' 0.1 ' ' 1

summary(shallow.numsp.depth)

##
## Family: Negative Binomial(2.443)
## Link function: log
##
## Formula:
## NumSpe_sha ~ s(Latitude, Longitude, bs = "sos") + s(NumRec_sha) +
##     s(Depth_Mean)
##
## Parametric coefficients:
##             Estimate Std. Error z value Pr(>|z|)
```

```
## (Intercept)  7.00285    0.03944   177.6   <2e-16 ***
## ---
## Signif. codes:  0 '***' 0.001 '**' 0.01 '*' 0.05 '.' 0.1 ' ' 1
##
## Approximate significance of smooth terms:
##                             edf Ref.df Chi.sq p-value
## s(Latitude,Longitude) 26.439     49 132.36  <2e-16 ***
## s(NumRec_sha)          8.288      9 149.22  <2e-16 ***
## s(Depth_Mean)          2.871      9  37.11  <2e-16 ***
## ---
## Signif. codes:  0 '***' 0.001 '**' 0.01 '*' 0.05 '.' 0.1 ' ' 1
##
## R-sq.(adj) =  0.753   Deviance explained = 71.2%
## -REML = 2132.6  Scale est. = 1         n = 264
```

```r
shallow.numsp.shelf <- gam(NumSpe_sha ~ s(Latitude, Longitude, bs = "sos"
) + s(NumRec_sha) + s(Shelf_Mean), data = Ecological_Data_Global_Hex_sp,
family = "nb", method = "REML", select = TRUE)
gam.check(shallow.numsp.shelf)
```

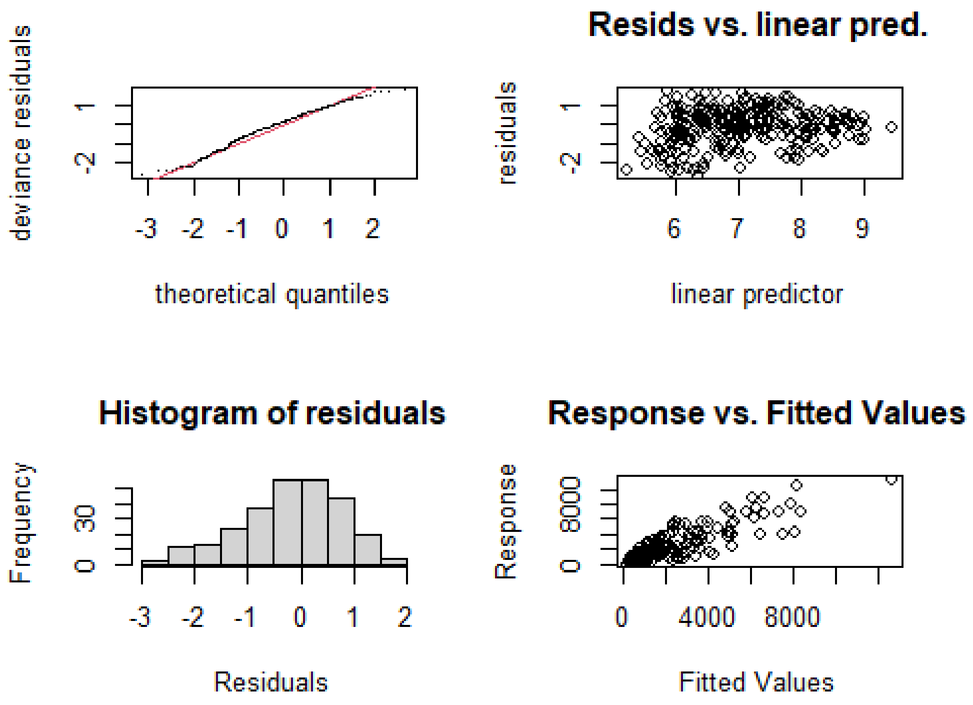


```
##
## Method: REML   Optimizer: outer newton
## full convergence after 13 iterations.
## Gradient range [-0.0006918394,0.0007296982]
## (score 2145.177 & scale 1).
## Hessian positive definite, eigenvalue range [0.0006395286,122.5643].
## Model rank =  68 / 68
##
## Basis dimension (k) checking results. Low p-value (k-index<1) may
## indicate that k is too low, especially if edf is close to k'.
```

```
##
##                              k'    edf k-index p-value
## s(Latitude,Longitude) 49.000 28.235    0.95    0.30
## s(NumRec_sha)          9.000  8.383    0.58  <2e-16 ***
## s(Shelf_Mean)          9.000  0.956    0.95    0.38
## ---
## Signif. codes:  0 '***' 0.001 '**' 0.01 '*' 0.05 '.' 0.1 ' ' 1
```

```
summary(shallow.numsp.shelf)
```

```
##
## Family: Negative Binomial(2.244)
## Link function: log
##
## Formula:
## NumSpe_sha ~ s(Latitude, Longitude, bs = "sos") + s(NumRec_sha) +
##     s(Shelf_Mean)
##
## Parametric coefficients:
##             Estimate Std. Error z value Pr(>|z|)
## (Intercept)  7.02084    0.04114   170.6   <2e-16 ***
## ---
## Signif. codes:  0 '***' 0.001 '**' 0.01 '*' 0.05 '.' 0.1 ' ' 1
##
## Approximate significance of smooth terms:
##                           edf Ref.df  Chi.sq p-value
## s(Latitude,Longitude) 28.2345     49 151.125  <2e-16 ***
## s(NumRec_sha)          8.3830      9 163.975  <2e-16 ***
## s(Shelf_Mean)          0.9563      9   2.014  0.0975 .
## ---
## Signif. codes:  0 '***' 0.001 '**' 0.01 '*' 0.05 '.' 0.1 ' ' 1
##
## R-sq.(adj) =  0.721   Deviance explained = 68.4%
## -REML = 2145.2  Scale est. = 1         n = 264
```

```
shallow.numsp.current <- gam(NumSpe_sha ~ s(Latitude, Longitude, bs = "so
s") + s(NumRec_sha) + s(CurVel_Mean), data = Ecological_Data_Global_Hex_s
p, family = "nb", method = "REML", select = TRUE)
gam.check(shallow.numsp.current)
```

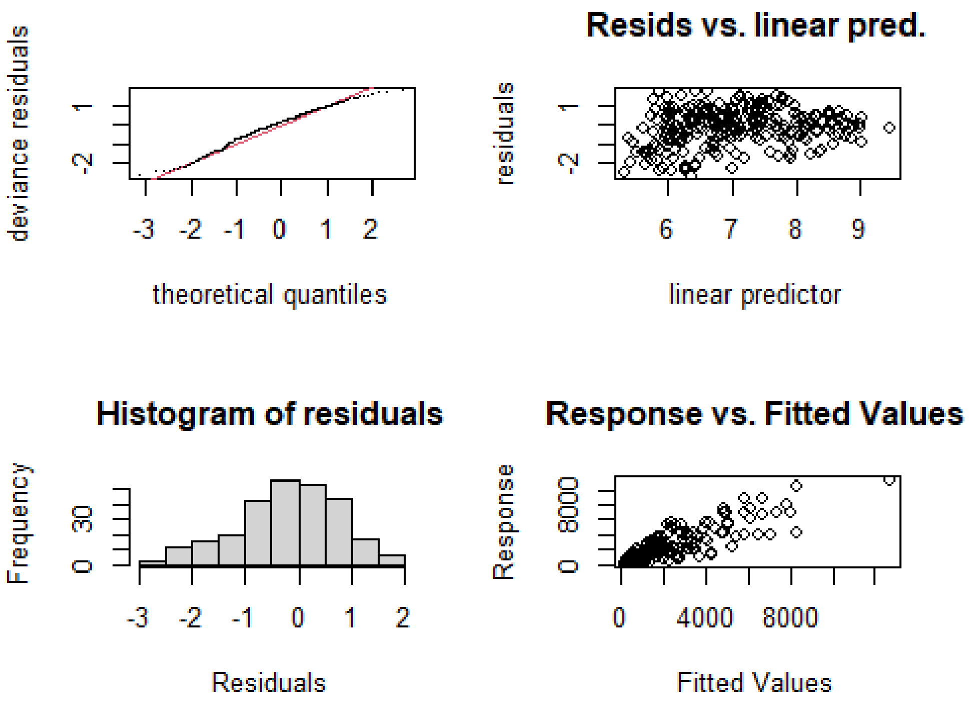


```
##
## Method: REML   Optimizer: outer newton
## full convergence after 11 iterations.
## Gradient range [-0.000464599,-4.843542e-06]
## (score 2145.475 & scale 1).
## Hessian positive definite, eigenvalue range [0.0003146368,122.6051].
## Model rank =  68 / 68
##
## Basis dimension (k) checking results. Low p-value (k-index<1) may
## indicate that k is too low, especially if edf is close to k'.
##
##                              k'      edf k-index p-value
## s(Latitude,Longitude) 49.00000 28.76400    0.94    0.38
## s(NumRec_sha)          9.00000  8.37280    0.58  <2e-16 ***
## s(CurVel_Mean)         9.00000  0.00388    0.90    0.17
## ---
## Signif. codes:  0 '***' 0.001 '**' 0.01 '*' 0.05 '.' 0.1 ' ' 1
```

```
summary(shallow.numsp.current)
```

```
##
## Family: Negative Binomial(2.24)
## Link function: log
##
## Formula:
## NumSpe_sha ~ s(Latitude, Longitude, bs = "sos") + s(NumRec_sha) +
##     s(CurVel_Mean)
##
## Parametric coefficients:
##             Estimate Std. Error z value Pr(>|z|)
```

```
## (Intercept)  7.02155    0.04118   170.5   <2e-16 ***
## ---
## Signif. codes:  0 '***' 0.001 '**' 0.01 '*' 0.05 '.' 0.1 ' ' 1
##
## Approximate significance of smooth terms:
##                               edf Ref.df  Chi.sq p-value
## s(Latitude,Longitude) 28.764000     49 158.383  <2e-16 ***
## s(NumRec_sha)          8.372799      9 161.559  <2e-16 ***
## s(CurVel_Mean)         0.003879      9   0.002    0.49
## ---
## Signif. codes:  0 '***' 0.001 '**' 0.01 '*' 0.05 '.' 0.1 ' ' 1
##
## R-sq.(adj) =  0.713   Deviance explained = 68.3%
## -REML = 2145.5  Scale est. = 1         n = 264
```

```
shallow.numsp.humimp <- gam(NumSpe_sha ~ s(Latitude, Longitude, bs = "sos
") + s(NumRec_sha) + s(HumImp_Mean), data = Ecological_Data_Global_Hex_sp
, family = "nb", method = "REML", select = TRUE)
gam.check(shallow.numsp.humimp)
```

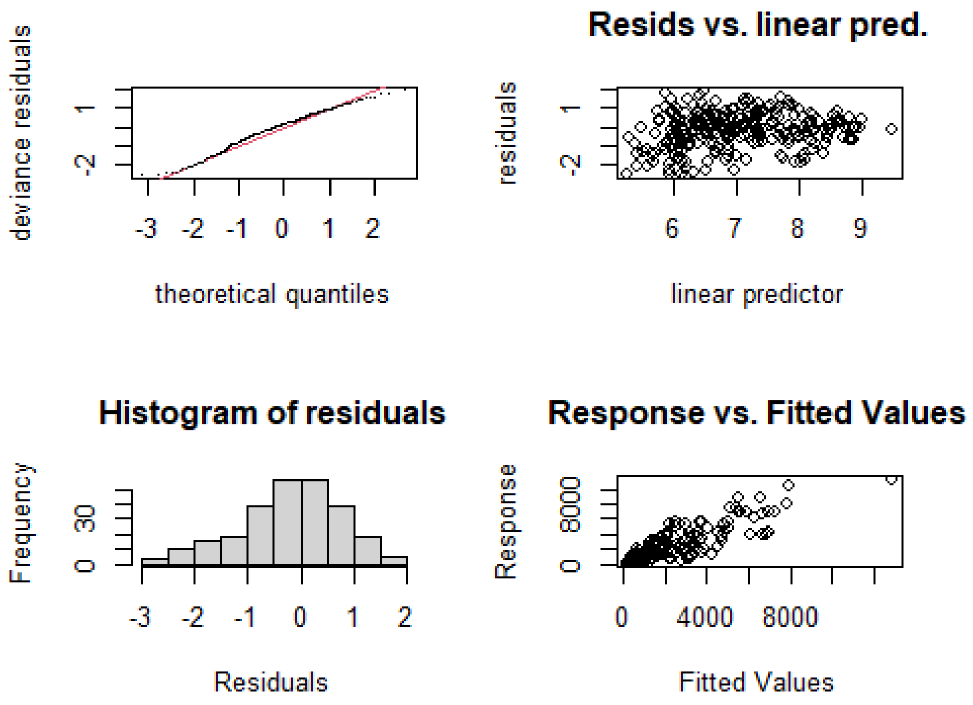


```
##
## Method: REML   Optimizer: outer newton
## full convergence after 10 iterations.
## Gradient range [-0.001053473,-6.70433e-05]
## (score 2143.516 & scale 1).
## Hessian positive definite, eigenvalue range [0.001956503,122.5904].
## Model rank =  68 / 68
##
## Basis dimension (k) checking results. Low p-value (k-index<1) may
## indicate that k is too low, especially if edf is close to k'.
```

```
##
##                              k'    edf k-index p-value
## s(Latitude,Longitude) 49.000 27.310    0.93    0.23
## s(NumRec_sha)          9.000  8.334    0.59  <2e-16 ***
## s(HumImp_Mean)         9.000  0.918    0.97    0.52
## ---
## Signif. codes:  0 '***' 0.001 '**' 0.01 '*' 0.05 '.' 0.1 ' ' 1
```

```
summary(shallow.numsp.humimp)
```

```
##
## Family: Negative Binomial(2.248)
## Link function: log
##
## Formula:
## NumSpe_sha ~ s(Latitude, Longitude, bs = "sos") + s(NumRec_sha) +
##     s(HumImp_Mean)
##
## Parametric coefficients:
##             Estimate Std. Error z value Pr(>|z|)
## (Intercept)   7.0212     0.0411   170.8   <2e-16 ***
## ---
## Signif. codes:  0 '***' 0.001 '**' 0.01 '*' 0.05 '.' 0.1 ' ' 1
##
## Approximate significance of smooth terms:
##                           edf Ref.df  Chi.sq p-value
## s(Latitude,Longitude) 27.310     49 130.881 < 2e-16 ***
## s(NumRec_sha)          8.334      9 140.221 < 2e-16 ***
## s(HumImp_Mean)         0.918      9   6.504 0.00226 **
## ---
## Signif. codes:  0 '***' 0.001 '**' 0.01 '*' 0.05 '.' 0.1 ' ' 1
##
## R-sq.(adj) =  0.726   Deviance explained = 68.4%
## -REML = 2143.5  Scale est. = 1         n = 264
```

```
shallow.numsp.icecover <- gam(NumSpe_sha ~ s(Latitude, Longitude, bs = "s
os") + s(NumRec_sha) + s(IceCov_Mean), data = Ecological_Data_Global_Hex_
sp, family = "nb", method = "REML", select = TRUE)
gam.check(shallow.numsp.icecover)
```

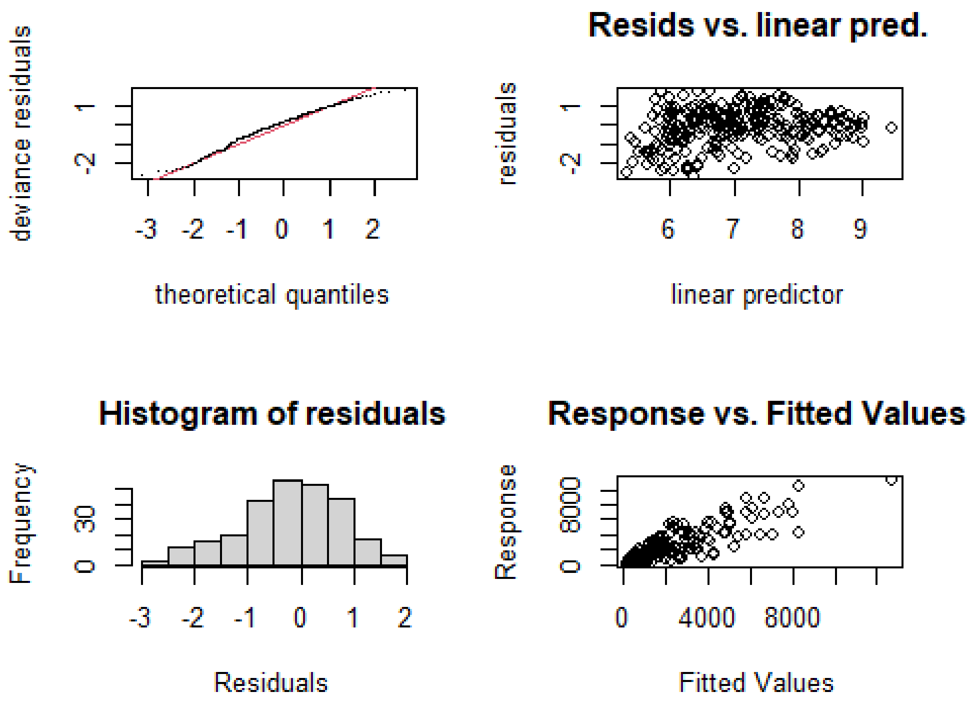


```
##
## Method: REML   Optimizer: outer newton
## full convergence after 9 iterations.
## Gradient range [-0.0009226175,0.0005466539]
## (score 2145.475 & scale 1).
## Hessian positive definite, eigenvalue range [1.272932e-05,122.6065].
## Model rank =  68 / 68
##
## Basis dimension (k) checking results. Low p-value (k-index<1) may
## indicate that k is too low, especially if edf is close to k'.
##
##                              k'      edf k-index p-value
## s(Latitude,Longitude) 49.00000 28.76377    0.94    0.41
## s(NumRec_sha)          9.00000  8.37280    0.58  <2e-16 ***
## s(IceCov_Mean)         9.00000  0.00194    0.96    0.44
## ---
## Signif. codes:  0 '***' 0.001 '**' 0.01 '*' 0.05 '.' 0.1 ' ' 1
```

```
summary(shallow.numsp.icecover)
```

```
##
## Family: Negative Binomial(2.24)
## Link function: log
##
## Formula:
## NumSpe_sha ~ s(Latitude, Longitude, bs = "sos") + s(NumRec_sha) +
##     s(IceCov_Mean)
##
## Parametric coefficients:
##             Estimate Std. Error z value Pr(>|z|)
```

```
## (Intercept)  7.02156    0.04118   170.5   <2e-16 ***
## ---
## Signif. codes:  0 '***' 0.001 '**' 0.01 '*' 0.05 '.' 0.1 ' ' 1
##
## Approximate significance of smooth terms:
##                               edf Ref.df Chi.sq p-value
## s(Latitude,Longitude) 28.763775     49   158.4  <2e-16 ***
## s(NumRec_sha)          8.372795      9   161.6  <2e-16 ***
## s(IceCov_Mean)         0.001937      9     0.0   0.961
## ---
## Signif. codes:  0 '***' 0.001 '**' 0.01 '*' 0.05 '.' 0.1 ' ' 1
##
## R-sq.(adj) =  0.713   Deviance explained = 68.3%
## -REML = 2145.5  Scale est. = 1         n = 264
```

```
shallow.numsp.nitrate <- gam(NumSpe_sha ~ s(Latitude, Longitude, bs = "so
s") + s(NumRec_sha) + s(Nitrate_Mean), data = Ecological_Data_Global_Hex_
sp, family = "nb", method = "REML", select = TRUE)
gam.check(shallow.numsp.nitrate)
```

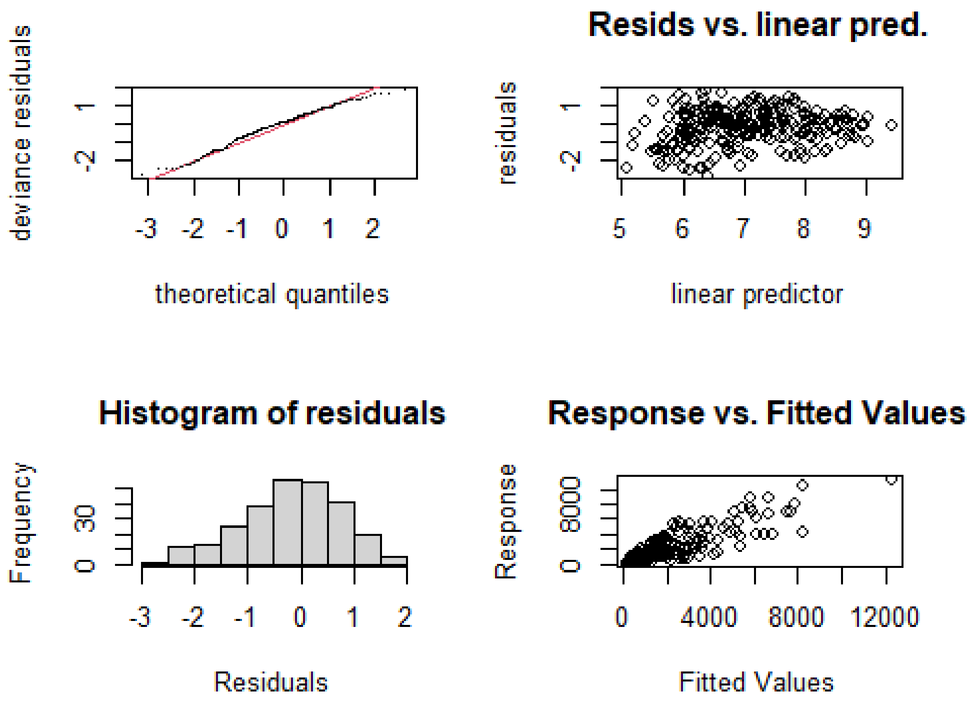


```
##
## Method: REML   Optimizer: outer newton
## full convergence after 10 iterations.
## Gradient range [-0.0005373999,0.000419698]
## (score 2143.491 & scale 1).
## Hessian positive definite, eigenvalue range [0.0005371309,120.8337].
## Model rank =  68 / 68
##
## Basis dimension (k) checking results. Low p-value (k-index<1) may
## indicate that k is too low, especially if edf is close to k'.
```

```
##
##                              k'    edf k-index p-value
## s(Latitude,Longitude) 49.000 29.227    0.96    0.47
## s(NumRec_sha)          9.000  8.344    0.60  <2e-16 ***
## s(Nitrate_Mean)        9.000  0.885    0.94    0.33
## ---
## Signif. codes:  0 '***' 0.001 '**' 0.01 '*' 0.05 '.' 0.1 ' ' 1
```

```
summary(shallow.numsp.nitrate)
```

```
##
## Family: Negative Binomial(2.298)
## Link function: log
##
## Formula:
## NumSpe_sha ~ s(Latitude, Longitude, bs = "sos") + s(NumRec_sha) +
##     s(Nitrate_Mean)
##
## Parametric coefficients:
##             Estimate Std. Error z value Pr(>|z|)
## (Intercept)  7.01482    0.04066   172.5   <2e-16 ***
## ---
## Signif. codes:  0 '***' 0.001 '**' 0.01 '*' 0.05 '.' 0.1 ' ' 1
##
## Approximate significance of smooth terms:
##                           edf Ref.df  Chi.sq p-value
## s(Latitude,Longitude) 29.2270     49 141.753  <2e-16 ***
## s(NumRec_sha)          8.3444      9 148.345  <2e-16 ***
## s(Nitrate_Mean)        0.8852      9   7.757  0.0012 **
## ---
## Signif. codes:  0 '***' 0.001 '**' 0.01 '*' 0.05 '.' 0.1 ' ' 1
##
## R-sq.(adj) =  0.723   Deviance explained = 69.3%
## -REML = 2143.5  Scale est. = 1         n = 264
```

```
shallow.numsp.PrimProd <- gam(NumSpe_sha ~ s(Latitude, Longitude, bs = "s
os") + s(NumRec_sha) + s(PrimProd_Mean), data = Ecological_Data_Global_He
x_sp, family = "nb", method = "REML", select = TRUE)
gam.check(shallow.numsp.PrimProd)
```

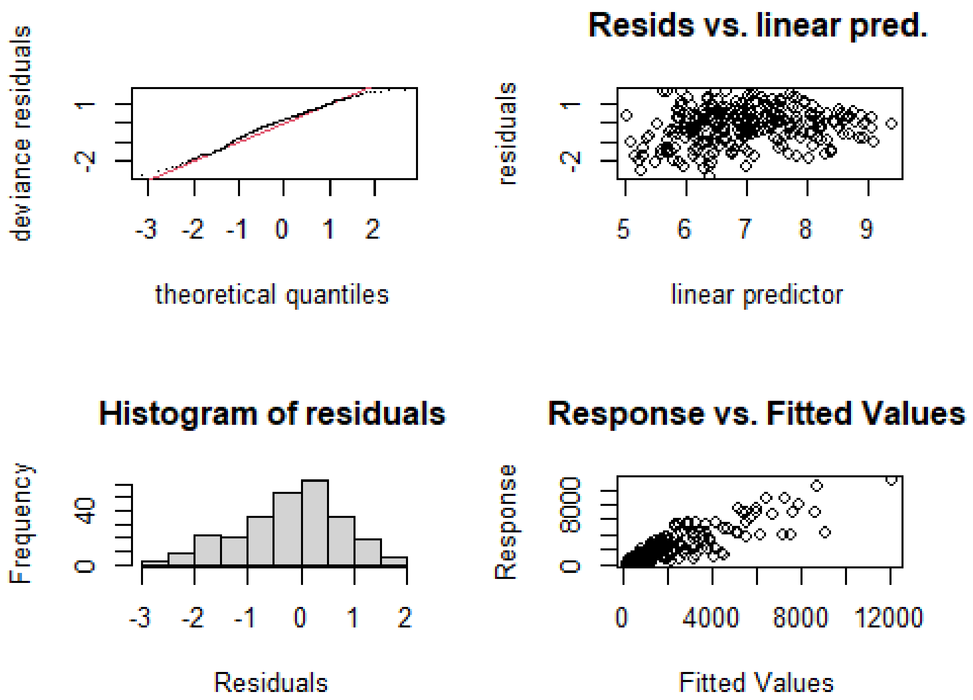


```
##
## Method: REML   Optimizer: outer newton
## full convergence after 8 iterations.
## Gradient range [-3.953658e-05,7.625657e-05]
## (score 2137.544 & scale 1).
## Hessian positive definite, eigenvalue range [3.374355e-05,122.122].
## Model rank =  68 / 68
##
## Basis dimension (k) checking results. Low p-value (k-index<1) may
## indicate that k is too low, especially if edf is close to k'.
##
##                             k'   edf k-index p-value
## s(Latitude,Longitude) 49.00 26.94    0.96    0.45
## s(NumRec_sha)          9.00  8.37    0.63  <2e-16 ***
## s(PrimProd_Mean)       9.00  2.21    0.91    0.17
## ---
## Signif. codes:  0 '***' 0.001 '**' 0.01 '*' 0.05 '.' 0.1 ' ' 1
```

```
summary(shallow.numsp.PrimProd)
```

```
##
## Family: Negative Binomial(2.361)
## Link function: log
##
## Formula:
## NumSpe_sha ~ s(Latitude, Longitude, bs = "sos") + s(NumRec_sha) +
##     s(PrimProd_Mean)
##
## Parametric coefficients:
##             Estimate Std. Error z value Pr(>|z|)
```

```
## (Intercept)  7.00994    0.04012   174.7   <2e-16 ***
## ---
## Signif. codes:  0 '***' 0.001 '**' 0.01 '*' 0.05 '.' 0.1 ' ' 1
##
## Approximate significance of smooth terms:
##                             edf Ref.df Chi.sq p-value
## s(Latitude,Longitude) 26.936     49 150.92 < 2e-16 ***
## s(NumRec_sha)          8.365      9 179.96 < 2e-16 ***
## s(PrimProd_Mean)       2.211      9  25.63 6.6e-07 ***
## ---
## Signif. codes:  0 '***' 0.001 '**' 0.01 '*' 0.05 '.' 0.1 ' ' 1
##
## R-sq.(adj) =  0.714   Deviance explained = 70.1%
## -REML = 2137.5  Scale est. = 1         n = 264
```

```
shallow.numsp.ThemM <- gam(NumSpe_sha ~ s(Latitude, Longitude, bs = "sos"
) + s(NumRec_sha) + s(ThemM_mean), data = Ecological_Data_Global_Hex_sp,
family = "nb", method = "REML", select = TRUE)
gam.check(shallow.numsp.ThemM)
```

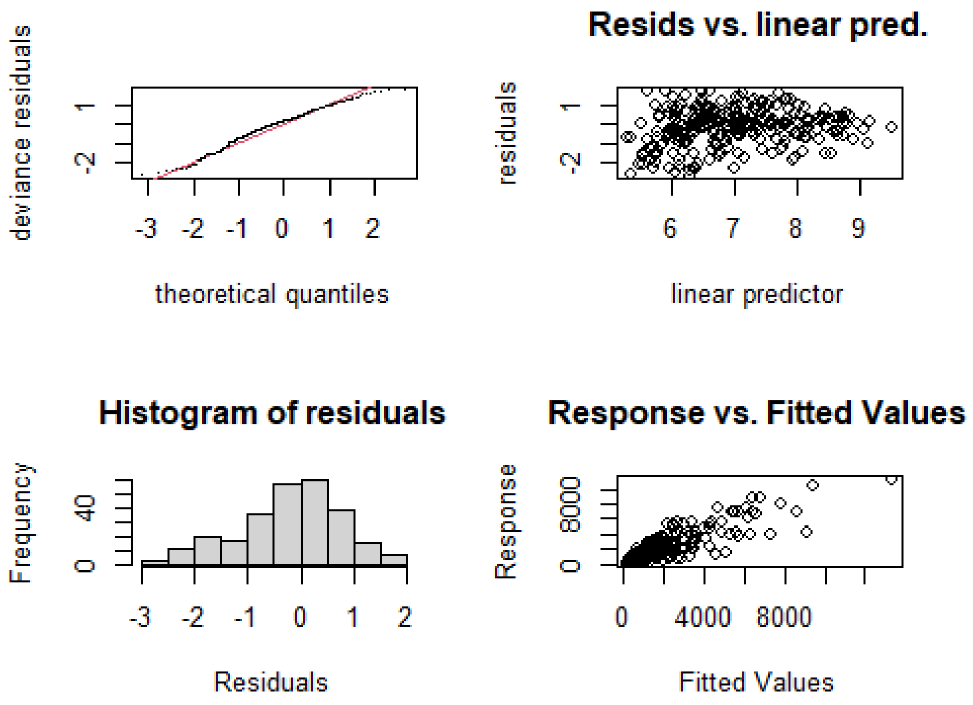


```
##
## Method: REML   Optimizer: outer newton
## full convergence after 7 iterations.
## Gradient range [-0.0001069884,0.0007878492]
## (score 2142.989 & scale 1).
## Hessian positive definite, eigenvalue range [8.895752e-05,121.531].
## Model rank =  68 / 68
##
## Basis dimension (k) checking results. Low p-value (k-index<1) may
## indicate that k is too low, especially if edf is close to k'.
```

```
##
##                             k'   edf k-index p-value
## s(Latitude,Longitude) 49.00 27.08    0.95    0.40
## s(NumRec_sha)          9.00  8.38    0.59  <2e-16 ***
## s(ThemM_mean)          9.00  2.26    0.89    0.11
## ---
## Signif. codes:  0 '***' 0.001 '**' 0.01 '*' 0.05 '.' 0.1 ' ' 1
```

```
summary(shallow.numsp.ThemM)
```

```
##
## Family: Negative Binomial(2.293)
## Link function: log
##
## Formula:
## NumSpe_sha ~ s(Latitude, Longitude, bs = "sos") + s(NumRec_sha) +
##     s(ThemM_mean)
##
## Parametric coefficients:
##             Estimate Std. Error z value Pr(>|z|)
## (Intercept)   7.0160     0.0407   172.4   <2e-16 ***
## ---
## Signif. codes:  0 '***' 0.001 '**' 0.01 '*' 0.05 '.' 0.1 ' ' 1
##
## Approximate significance of smooth terms:
##                          edf Ref.df  Chi.sq  p-value
## s(Latitude,Longitude) 27.079     49 119.327  < 2e-16 ***
## s(NumRec_sha)          8.384      9 161.735  < 2e-16 ***
## s(ThemM_mean)          2.259      9   9.774 1.99e-05 ***
## ---
## Signif. codes:  0 '***' 0.001 '**' 0.01 '*' 0.05 '.' 0.1 ' ' 1
##
## R-sq.(adj) =   0.72   Deviance explained = 69.2%
## -REML =   2143  Scale est. = 1         n = 264
```

```
shallow.numsp.ThemR <- gam(NumSpe_sha ~ s(Latitude, Longitude, bs = "sos"
) + s(NumRec_sha) + s(ThemR_mean), data = Ecological_Data_Global_Hex_sp,
family = "nb", method = "REML", select = TRUE)
gam.check(shallow.numsp.ThemR)
```

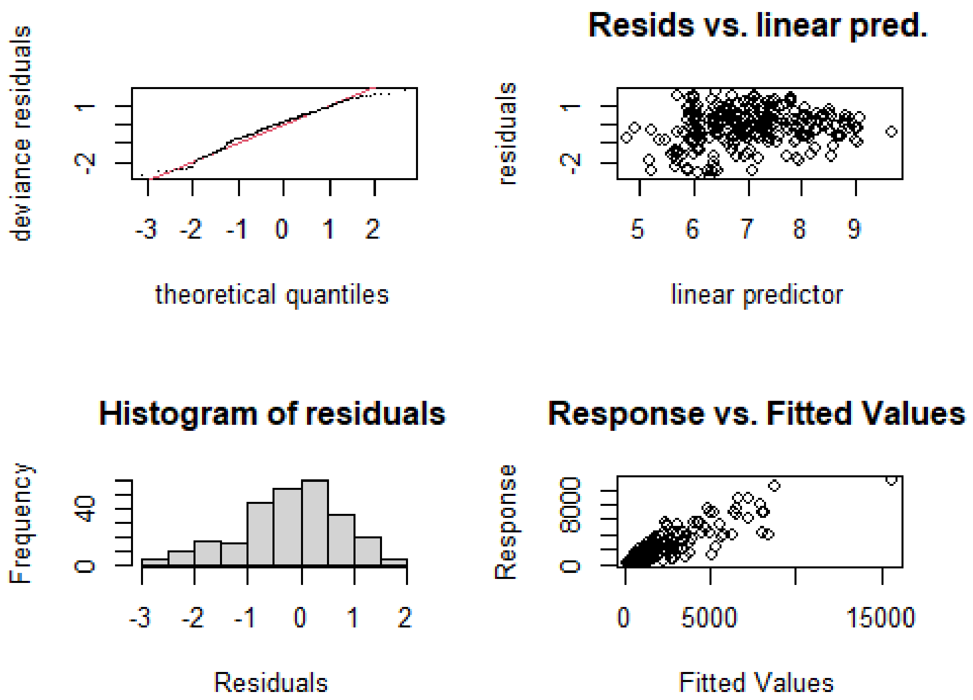


```
##
## Method: REML   Optimizer: outer newton
## full convergence after 8 iterations.
## Gradient range [-0.0001144282,0.0001551182]
## (score 2141.377 & scale 1).
## Hessian positive definite, eigenvalue range [0.0001145148,120.0083].
## Model rank =  68 / 68
##
## Basis dimension (k) checking results. Low p-value (k-index<1) may
## indicate that k is too low, especially if edf is close to k'.
##
##                            k'   edf k-index p-value
## s(Latitude,Longitude) 49.00 28.45    0.95    0.42
## s(NumRec_sha)          9.00  8.38    0.59  <2e-16 ***
## s(ThemR_mean)          9.00  3.00    0.92    0.21
## ---
## Signif. codes:  0 '***' 0.001 '**' 0.01 '*' 0.05 '.' 0.1 ' ' 1
```

```
summary(shallow.numsp.ThemR)
```

```
##
## Family: Negative Binomial(2.366)
## Link function: log
##
## Formula:
## NumSpe_sha ~ s(Latitude, Longitude, bs = "sos") + s(NumRec_sha) +
##     s(ThemR_mean)
##
## Parametric coefficients:
##             Estimate Std. Error z value Pr(>|z|)
```

```
## (Intercept)  7.00759    0.04007   174.9   <2e-16 ***
## ---
## Signif. codes:  0 '***' 0.001 '**' 0.01 '*' 0.05 '.' 0.1 ' ' 1
##
## Approximate significance of smooth terms:
##                             edf Ref.df Chi.sq  p-value
## s(Latitude,Longitude) 28.451     49  147.9  < 2e-16 ***
## s(NumRec_sha)          8.385      9  165.6  < 2e-16 ***
## s(ThemR_mean)          2.997      9   18.6 8.66e-06 ***
## ---
## Signif. codes:  0 '***' 0.001 '**' 0.01 '*' 0.05 '.' 0.1 ' ' 1
##
## R-sq.(adj) =  0.704   Deviance explained = 70.4%
## -REML = 2141.4  Scale est. = 1         n = 264
```

```
shallow.numsp.env <- gam(NumSpe_sha ~ s(Latitude, Longitude, bs = "sos")
+ s(NumRec_sha) + s(Depth_Mean) +  s(Shelf_Mean) + s(CurVel_Mean) + s(Hum
Imp_Mean) + s(IceCov_Mean) + s(Nitrate_Mean) + s(PrimProd_Mean) + s(ThemM
_mean) + s(ThemR_mean), data = Ecological_Data_Global_Hex_sp, family = "n
b", method = "REML", select = TRUE)
gam.check(shallow.numsp.env)
```

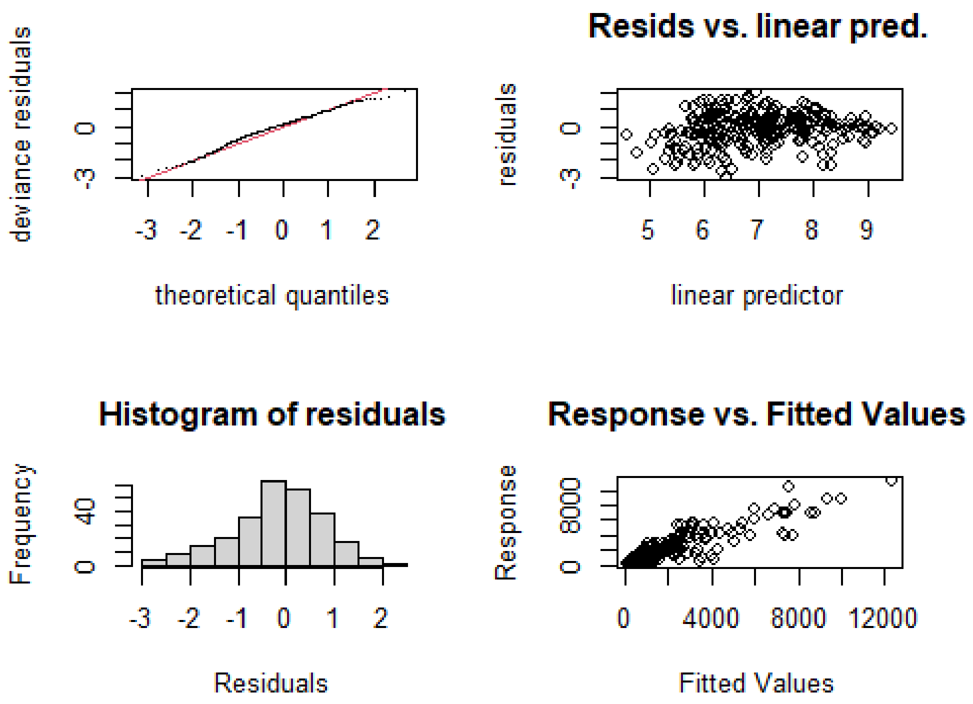


```
##
## Method: REML   Optimizer: outer newton
## full convergence after 16 iterations.
## Gradient range [-0.007756468,0.0007283321]
## (score 2119.93 & scale 1).
## Hessian positive definite, eigenvalue range [3.029655e-05,118.7714].
## Model rank =  140 / 140
##
```

```
## Basis dimension (k) checking results. Low p-value (k-index<1) may
## indicate that k is too low, especially if edf is close to k'.
##
##                              k'      edf k-index p-value
## s(Latitude,Longitude) 4.90e+01 2.14e+01    0.97   0.485
## s(NumRec_sha)         9.00e+00 8.19e+00    0.63  <2e-16 ***
## s(Depth_Mean)         9.00e+00 2.83e+00    0.96   0.410
## s(Shelf_Mean)         9.00e+00 2.98e-04    0.93   0.255
## s(CurVel_Mean)        9.00e+00 1.14e-03    0.90   0.150
## s(HumImp_Mean)        9.00e+00 1.06e+00    1.03   0.820
## s(IceCov_Mean)        9.00e+00 4.23e-01    1.06   0.910
## s(Nitrate_Mean)       9.00e+00 1.11e-03    0.94   0.335
## s(PrimProd_Mean)      9.00e+00 2.06e+00    0.97   0.465
## s(ThemM_mean)         9.00e+00 8.59e-04    0.88   0.065 .
## s(ThemR_mean)         9.00e+00 2.80e+00    0.97   0.510
## ---
## Signif. codes:  0 '***' 0.001 '**' 0.01 '*' 0.05 '.' 0.1 ' ' 1
```

```
summary(shallow.numsp.env)
```

```
##
## Family: Negative Binomial(2.668)
## Link function: log
##
## Formula:
## NumSpe_sha ~ s(Latitude, Longitude, bs = "sos") + s(NumRec_sha) +
##     s(Depth_Mean) + s(Shelf_Mean) + s(CurVel_Mean) + s(HumImp_Mean) +
##     s(IceCov_Mean) + s(Nitrate_Mean) + s(PrimProd_Mean) + s(ThemM_mean
) +
##     s(ThemR_mean)
##
## Parametric coefficients:
##             Estimate Std. Error z value Pr(>|z|)
## (Intercept)  6.98512    0.03775     185   <2e-16 ***
## ---
## Signif. codes:  0 '***' 0.001 '**' 0.01 '*' 0.05 '.' 0.1 ' ' 1
##
## Approximate significance of smooth terms:
##                             edf Ref.df  Chi.sq  p-value
## s(Latitude,Longitude) 2.136e+01     49  82.999  < 2e-16 ***
## s(NumRec_sha)         8.193e+00      9 135.019  < 2e-16 ***
## s(Depth_Mean)         2.832e+00      9  39.364  < 2e-16 ***
## s(Shelf_Mean)         2.976e-04      9   0.000 0.659807
## s(CurVel_Mean)        1.139e-03      9   0.001 0.440787
## s(HumImp_Mean)        1.064e+00      9   9.232 0.000403 ***
## s(IceCov_Mean)        4.234e-01      9   0.702 0.108859
## s(Nitrate_Mean)       1.105e-03      9   0.000 0.555829
## s(PrimProd_Mean)      2.056e+00      9  17.339 2.05e-05 ***
## s(ThemM_mean)         8.587e-04      9   0.000 0.299133
## s(ThemR_mean)         2.803e+00      9  16.201 2.71e-05 ***
## ---
## Signif. codes:  0 '***' 0.001 '**' 0.01 '*' 0.05 '.' 0.1 ' ' 1
```

```
##
## R-sq.(adj) =  0.764   Deviance explained = 73.9%
## -REML = 2119.9  Scale est. = 1         n = 264
```

```
shallow.numsp.models <- list(Intercept = shallow.numsp.intercept,
                             LatLon = shallow.numsp.latlon,
                             Depth = shallow.numsp.depth,
                             ConShe = shallow.numsp.shelf,
                             CurVel = shallow.numsp.current,
                             HumImp = shallow.numsp.humimp,
                             IceCov = shallow.numsp.icecover,
                             Nitrate = shallow.numsp.nitrate,
                             PriPro = shallow.numsp.PrimProd,
                             TemMea = shallow.numsp.ThemM,
                             TheRan = shallow.numsp.ThemR,
                             Environment = shallow.numsp.env)
shallow.numsp.aic.df <- data.frame(Model = names(shallow.numsp.models),
                                   AIC = sapply(shallow.numsp.models, fun
ction(x) x$aic),
                                   akaike.weights(sapply(shallow.numsp.mo
dels, function(x) x$aic)))

shallow.numsp.aic.df <- shallow.numsp.aic.df[order(shallow.numsp.aic.df$A
IC),]
shallow.numsp.aic.df$Cumulative.Weight <- cumsum(shallow.numsp.aic.df$wei
ghts)

kable(shallow.numsp.aic.df, row.names = FALSE)
```

Model	AIC	deltaAIC	rel.LL	weights	Cumulative.Weight
Environment	4161.680	0.00000	1.0e+00	0.9999962	0.9999962
Depth	4186.679	24.99856	3.7e-06	0.0000037	1.0000000
PriPro	4196.799	35.11853	0.0e+00	0.0000000	1.0000000
TheRan	4198.408	36.72790	0.0e+00	0.0000000	1.0000000
Nitrate	4205.699	44.01852	0.0e+00	0.0000000	1.0000000
TemMea	4205.727	44.04649	0.0e+00	0.0000000	1.0000000
HumImp	4210.406	48.72608	0.0e+00	0.0000000	1.0000000
ConShe	4212.051	50.37115	0.0e+00	0.0000000	1.0000000
LatLon	4212.093	50.41328	0.0e+00	0.0000000	1.0000000
IceCov	4212.096	50.41555	0.0e+00	0.0000000	1.0000000
CurVel	4212.097	50.41680	0.0e+00	0.0000000	1.0000000
Intercept	4471.726	310.04629	0.0e+00	0.0000000	1.0000000

```
#write.csv(shallow.numsp.aic.df, file = "shallow.numsp.aic.GAM.csv")

#Plots for number of species, shallow water
ggplot(Ecological_Data_Global_Hex_sp, aes(x = Depth_Mean, y = predict(sha
llow.numsp.depth, Ecological_Data_Global_Hex_sp))) +
  geom_smooth(method = "gam", formula = y ~ x, color = "#1a80bb", fill =
```

```
"#85bede") +  # Add a smooth dark blue line with light blue shadow
  geom_point(size = 3) +  # Add scatter plot points
  theme_bw() +  # Use the black and white theme
  labs(
    x = "Depth (m)",  # Shorten the x-axis title
    y = "Predicted Value"  # Shorten the y-axis title
  ) +
  theme(
    panel.grid.minor = element_blank(),
    panel.grid.major = element_blank(),
    axis.text.x = element_text(size =20),  # Increase x-axis text size
    axis.text.y = element_text(size = 20),  # Increase y-axis text size
    axis.title.x = element_text(size = 22), # Increase x-axis title size
    axis.title.y = element_text(size = 22)  # Increase y-axis title size
  )
```

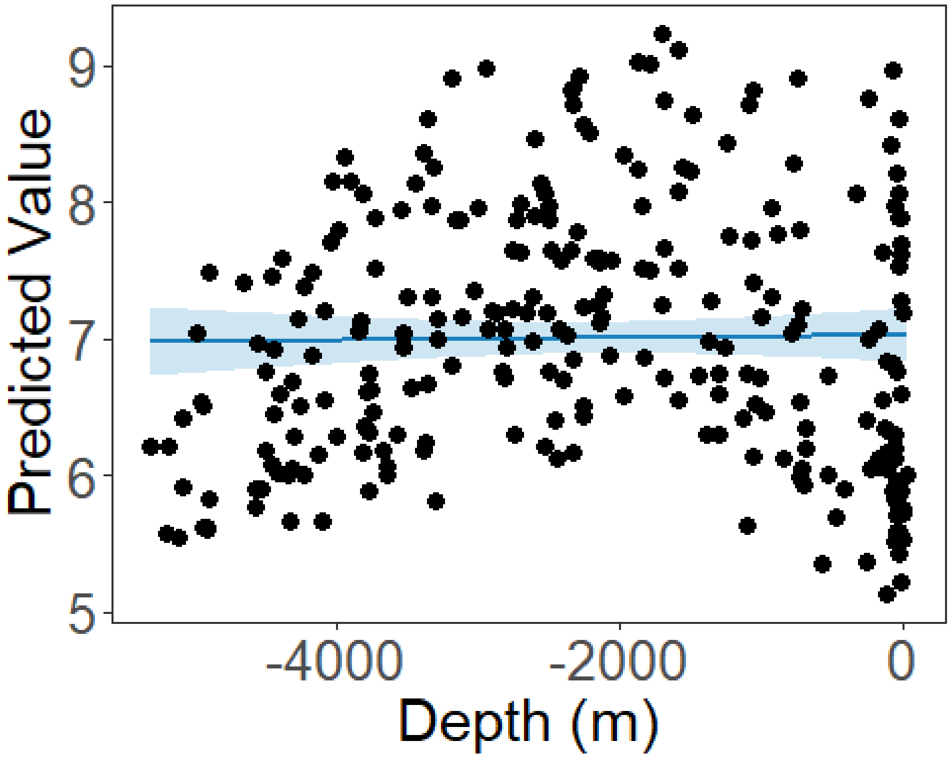


```
ggplot(Ecological_Data_Global_Hex_sp, aes(x = Shelf_Mean, y = predict(sha
llow.numsp.shelf, Ecological_Data_Global_Hex_sp))) +
  geom_smooth(method = "gam", formula = y ~ x, color = "#1a80bb", fill =
"#85bede") +  # Add a smooth dark blue line with light blue shadow
  geom_point(size = 3) +  # Add scatter plot points
  theme_bw() +  # Use the black and white theme
  labs(
    x = "Continental Shelf (km2)",  # Shorten the x-axis title
    y = "Predicted Value"  # Shorten the y-axis title
  ) +
  theme(
    panel.grid.minor = element_blank(),
    panel.grid.major = element_blank(),
    axis.text.x = element_text(size =20),  # Increase x-axis text size
```

```
    axis.text.y = element_text(size = 20),  # Increase y-axis text size
    axis.title.x = element_text(size = 22), # Increase x-axis title size
    axis.title.y = element_text(size = 22)  # Increase y-axis title size
  )
```

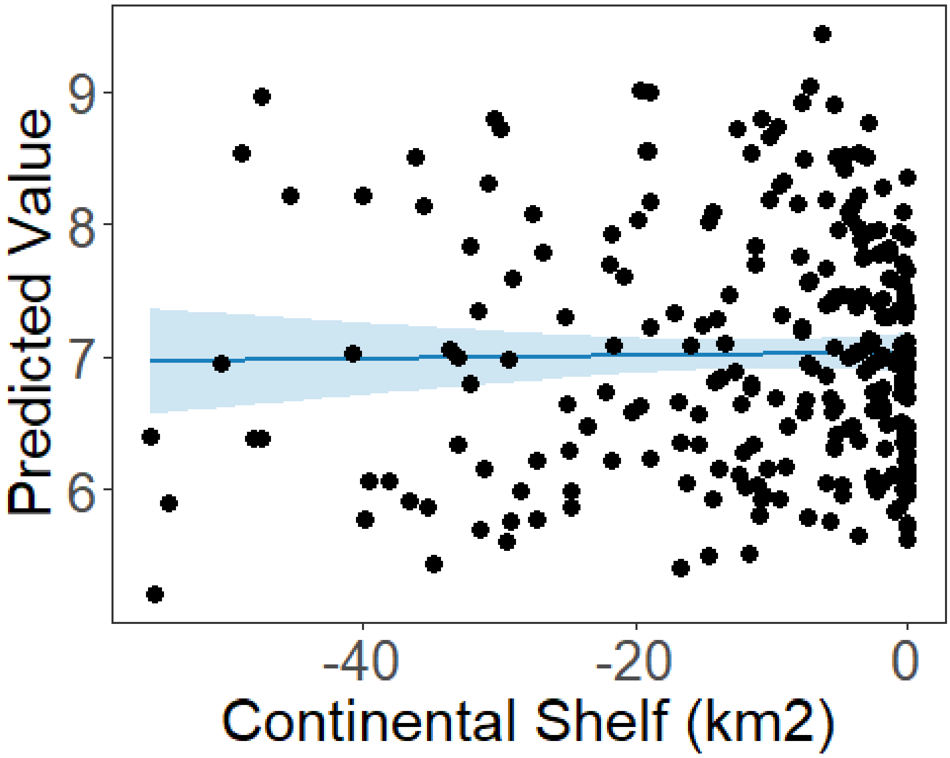


```
ggplot(Ecological_Data_Global_Hex_sp, aes(x = CurVel_Mean, y = predict(sh
allow.numsp.current, Ecological_Data_Global_Hex_sp))) +
  geom_smooth(method = "gam", formula = y ~ x, color = "#1a80bb", fill =
"#85bede") +  # Add a smooth dark blue line with light blue shadow
  geom_point(size = 3) +  # Add scatter plot points
  theme_bw() +  # Use the black and white theme
  labs(
    x = "Current Velocity (m.s-1)",  # Shorten the x-axis title
    y = "Predicted Value"  # Shorten the y-axis title
  ) +
  theme(
    panel.grid.minor = element_blank(),
    panel.grid.major = element_blank(),
    axis.text.x = element_text(size =20),  # Increase x-axis text size
    axis.text.y = element_text(size = 20),  # Increase y-axis text size
    axis.title.x = element_text(size = 22), # Increase x-axis title size
    axis.title.y = element_text(size = 22)  # Increase y-axis title size
  )
```

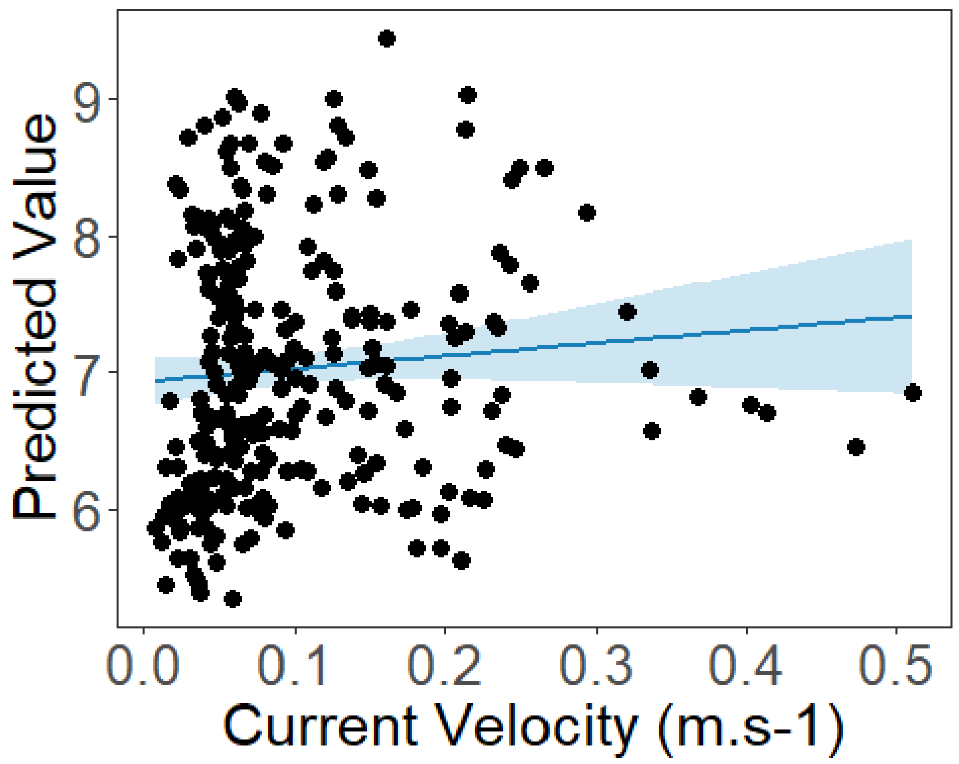


```
ggplot(Ecological_Data_Global_Hex_sp, aes(x = HumImp_Mean, y = predict(sh
allow.numsp.humimp, Ecological_Data_Global_Hex_sp))) +
  geom_smooth(method = "gam", formula = y ~ x, color = "#1a80bb", fill =
"#85bede") +  # Add a smooth dark blue line with light blue shadow
  geom_point(size = 3) +  # Add scatter plot points
  theme_bw() +  # Use the black and white theme
  labs(
    x = "Human Impact",  # Shorten the x-axis title
    y = "Predicted Value"  # Shorten the y-axis title
  ) +
  theme(
    panel.grid.minor = element_blank(),
    panel.grid.major = element_blank(),
    axis.text.x = element_text(size =20),  # Increase x-axis text size
    axis.text.y = element_text(size = 20),  # Increase y-axis text size
    axis.title.x = element_text(size = 22), # Increase x-axis title size
    axis.title.y = element_text(size = 22)  # Increase y-axis title size
  )
```

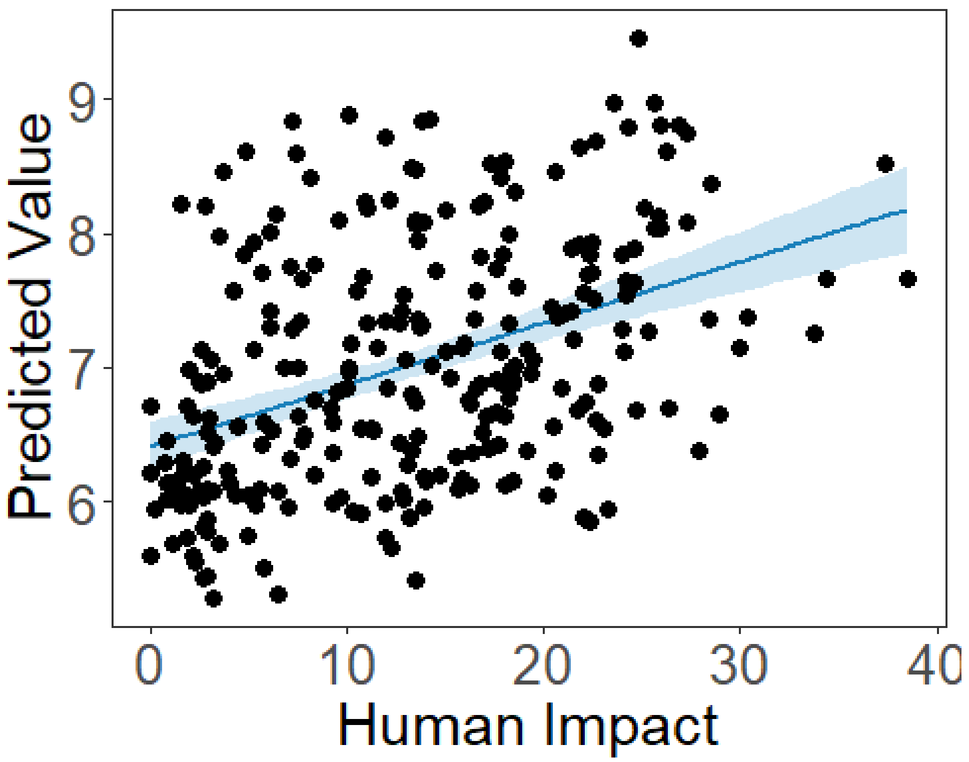


```r
ggplot(Ecological_Data_Global_Hex_sp, aes(x = IceCov_Mean, y = predict(sh
allow.numsp.icecover, Ecological_Data_Global_Hex_sp))) +
  geom_smooth(method = "gam", formula = y ~ x, color = "#1a80bb", fill =
"#85bede") +  # Add a smooth dark blue line with light blue shadow
  geom_point(size = 3) +  # Add scatter plot points
  theme_bw() +  # Use the black and white theme
  labs(
    x = "Ice Cover (fraction)",  # Shorten the x-axis title
    y = "Predicted Value"  # Shorten the y-axis title
  ) +
  theme(
    panel.grid.minor = element_blank(),
    panel.grid.major = element_blank(),
    axis.text.x = element_text(size =20),  # Increase x-axis text size
    axis.text.y = element_text(size = 20),  # Increase y-axis text size
    axis.title.x = element_text(size = 22), # Increase x-axis title size
    axis.title.y = element_text(size = 22)  # Increase y-axis title size
  )
```

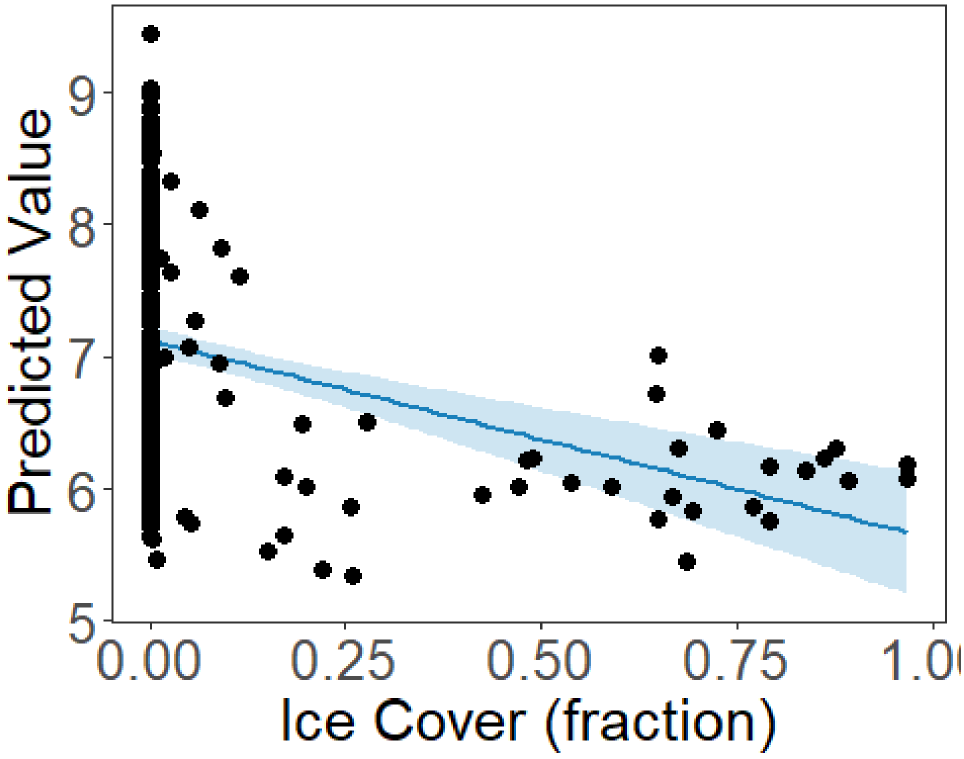


```
ggplot(Ecological_Data_Global_Hex_sp, aes(x = Nitrate_Mean, y = predict(s
hallow.numsp.nitrate, Ecological_Data_Global_Hex_sp))) +
  geom_smooth(method = "gam", formula = y ~ x, color = "#1a80bb", fill =
"#85bede") +  # Add a smooth dark blue line with light blue shadow
  geom_point(size = 3) +  # Add scatter plot points
  theme_bw() +  # Use the black and white theme
  labs(
    x = "Nitrate (mmol . m-3)",  # Shorten the x-axis title
    y = "Predicted Value"  # Shorten the y-axis title
  ) +
  theme(
    panel.grid.minor = element_blank(),
    panel.grid.major = element_blank(),
    axis.text.x = element_text(size =20),  # Increase x-axis text size
    axis.text.y = element_text(size = 20),  # Increase y-axis text size
    axis.title.x = element_text(size = 22), # Increase x-axis title size
    axis.title.y = element_text(size = 22)  # Increase y-axis title size
  )
```

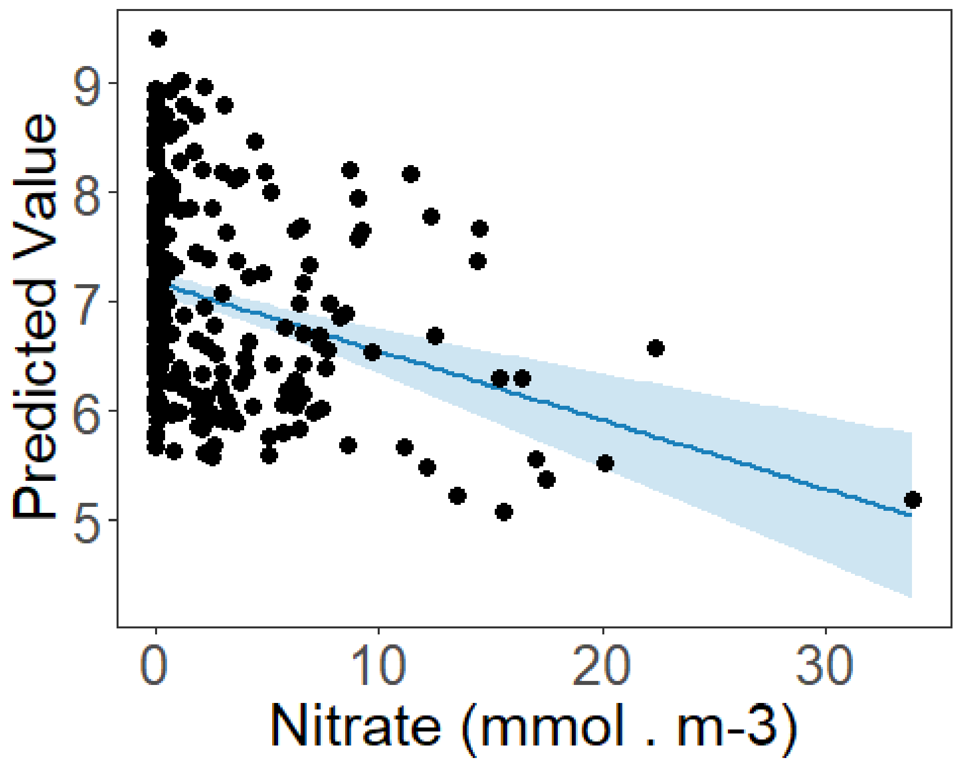


```
ggplot(Ecological_Data_Global_Hex_sp, aes(x = PrimProd_Mean, y = predict(
shallow.numsp.PrimProd, Ecological_Data_Global_Hex_sp))) +
  geom_smooth(method = "gam", formula = y ~ x, color = "#1a80bb", fill =
"#85bede") +  # Add a smooth dark blue line with light blue shadow
  geom_point(size = 3) +  # Add scatter plot points
  theme_bw() +  # Use the black and white theme
  labs(
    x = "Primary Productivity (mmol . m-3)",  # Shorten the x-axis title
    y = "Predicted Value"  # Shorten the y-axis title
  ) +
  theme(
    panel.grid.minor = element_blank(),
    panel.grid.major = element_blank(),
    axis.text.x = element_text(size =20),  # Increase x-axis text size
    axis.text.y = element_text(size = 20),  # Increase y-axis text size
    axis.title.x = element_text(size = 22), # Increase x-axis title size
    axis.title.y = element_text(size = 22)  # Increase y-axis title size
  )
```

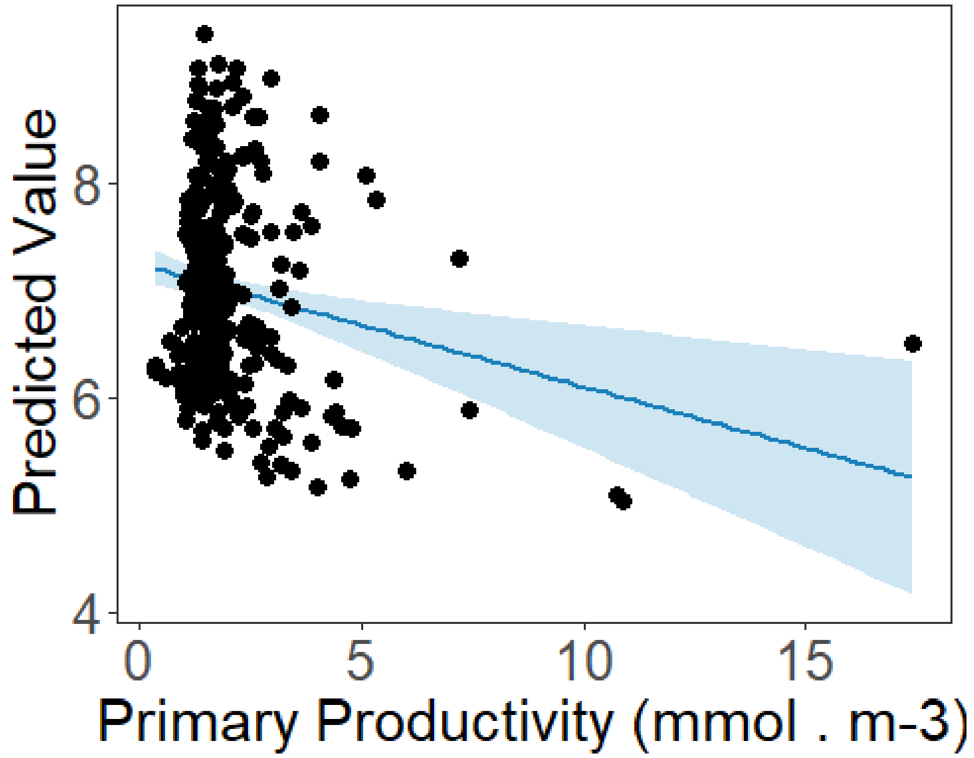


```
ggplot(Ecological_Data_Global_Hex_sp, aes(x = ThemM_mean, y = predict(sha
llow.numsp.ThemM, Ecological_Data_Global_Hex_sp))) +
  geom_smooth(method = "gam", formula = y ~ x, color = "#1a80bb", fill =
"#85bede") +  # Add a smooth dark blue line with light blue shadow
  geom_point(size = 3) +  # Add scatter plot points
  theme_bw() +  # Use the black and white theme
  labs(
    x = "Temperature Mean (ºC)",  # Shorten the x-axis title
    y = "Predicted Value"  # Shorten the y-axis title
  ) +
  theme(
    panel.grid.minor = element_blank(),
    panel.grid.major = element_blank(),
    axis.text.x = element_text(size =20),  # Increase x-axis text size
    axis.text.y = element_text(size = 20),  # Increase y-axis text size
    axis.title.x = element_text(size = 22), # Increase x-axis title size
    axis.title.y = element_text(size = 22)  # Increase y-axis title size
  )
```

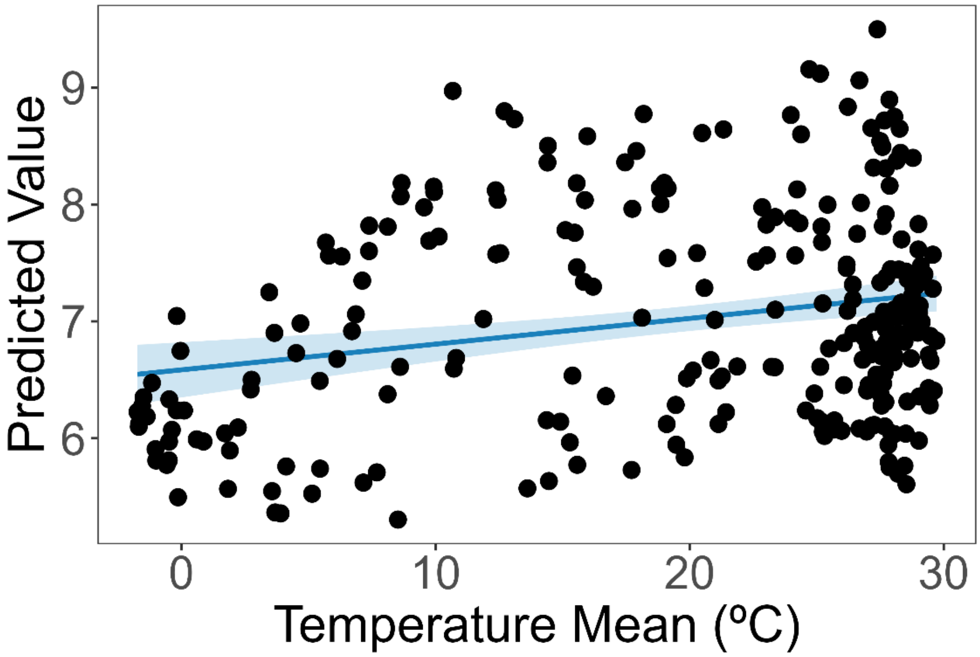


```
ggplot(Ecological_Data_Global_Hex_sp, aes(x = ThemR_mean, y = predict(sha
llow.numsp.ThemR, Ecological_Data_Global_Hex_sp))) +
  geom_smooth(method = "gam", formula = y ~ x, color = "#1a80bb", fill =
"#85bede") +  # Add a smooth dark blue line with light blue shadow
  geom_point(size = 3) +  # Add scatter plot points
  theme_bw() +  # Use the black and white theme
  labs(
    x = "Temperature Range (ºC)",  # Shorten the x-axis title
    y = "Predicted Value"  # Shorten the y-axis title
  ) +
  theme(
    panel.grid.minor = element_blank(),
    panel.grid.major = element_blank(),
    axis.text.x = element_text(size =20),  # Increase x-axis text size
    axis.text.y = element_text(size = 20),  # Increase y-axis text size
    axis.title.x = element_text(size = 22), # Increase x-axis title size
    axis.title.y = element_text(size = 22)  # Increase y-axis title size
  )
```

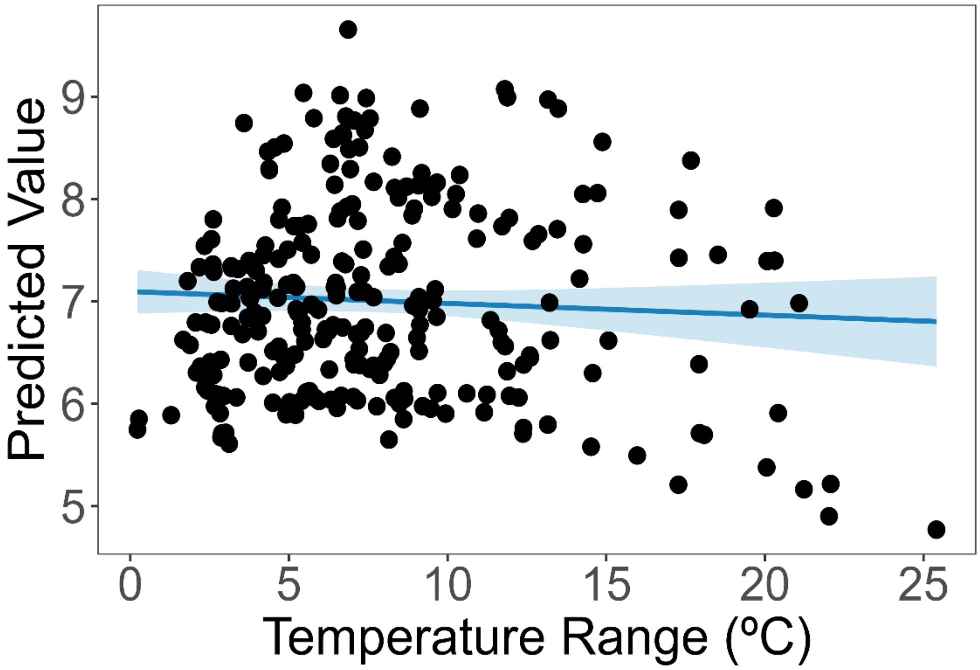


```
# ==================================
#                 SHALLOW-ES50-GAM
# ==================================

shallow.ES50.intercept <- gam(ES50_sha ~ 1, data = Ecological_Data_Global
_Hex_sp, family = "nb", method = "REML", select = TRUE)
gam.check(shallow.ES50.intercept)
```

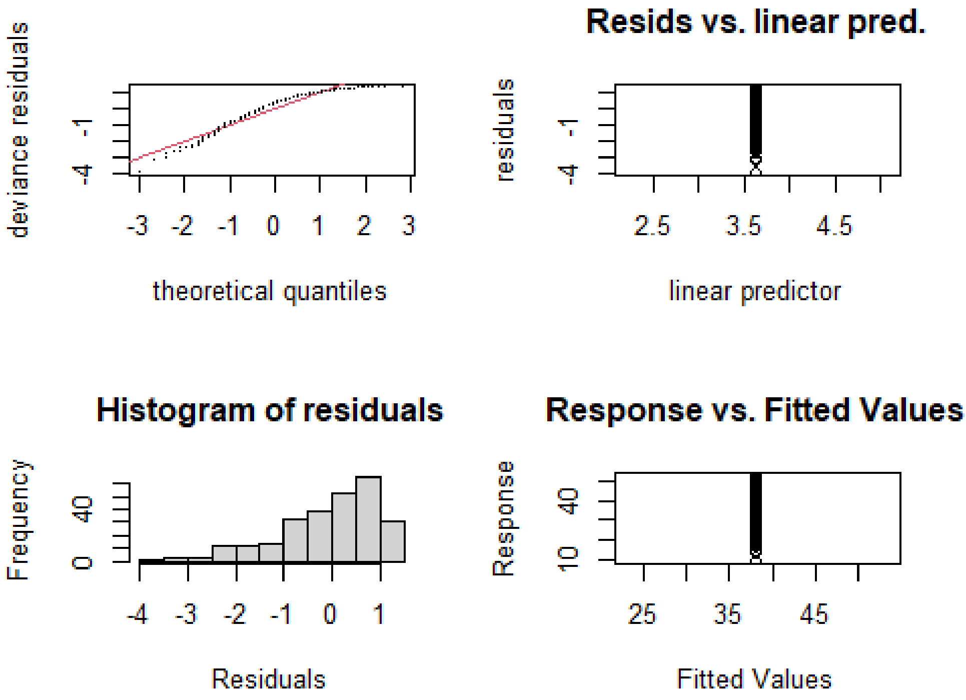


```
##
## Method: REML   Optimizer: outer newton
## full convergence after 5 iterations.
```

```
## Gradient range [-7.949184e-09,-7.949184e-09]
## (score 993.6973 & scale 1).
## Hessian positive definite, eigenvalue range [49.47001,49.47001].
## Model rank =  1 / 1
```

```
summary(shallow.ES50.intercept)
```

```
##
## Family: Negative Binomial(21.598)
## Link function: log
##
## Formula:
## ES50_sha ~ 1
##
## Parametric coefficients:
##             Estimate Std. Error z value Pr(>|z|)
## (Intercept)  3.64602    0.01656   220.2   <2e-16 ***
## ---
## Signif. codes:  0 '***' 0.001 '**' 0.01 '*' 0.05 '.' 0.1 ' ' 1
##
##
## R-sq.(adj) =      0   Deviance explained = 1.57e-09%
## -REML =   993.7  Scale est. = 1         n = 264
```

```
shallow.ES50.latlon <- gam(ES50_sha ~ s(Latitude, Longitude, bs = "sos"),
data = Ecological_Data_Global_Hex_sp, family = "nb", method = "REML", sel
ect = TRUE)
gam.check(shallow.ES50.latlon)
```

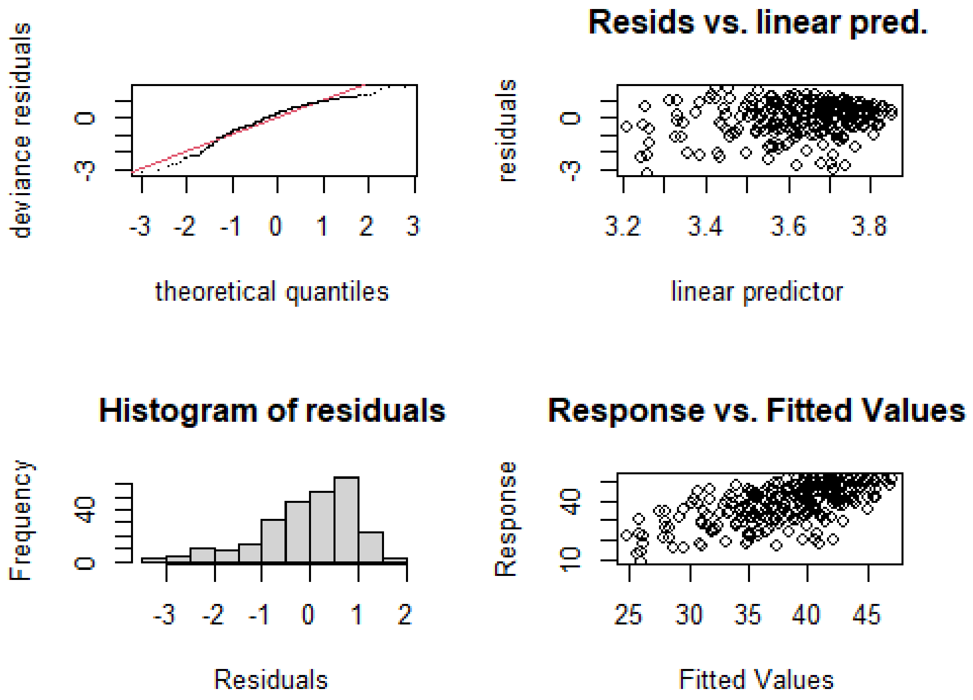


```
##
## Method: REML   Optimizer: outer newton
```

```
## full convergence after 7 iterations.
## Gradient range [-6.785323e-09,-2.605519e-09]
## (score 975.5277 & scale 1).
## Hessian positive definite, eigenvalue range [6.584817,21.3012].
## Model rank =  50 / 50
##
## Basis dimension (k) checking results. Low p-value (k-index<1) may
## indicate that k is too low, especially if edf is close to k'.
##
##                            k'  edf k-index p-value
## s(Latitude,Longitude) 49.0 26.5    0.97     0.3
```

```
summary(shallow.ES50.latlon)
```

```
##
## Family: Negative Binomial(44.052)
## Link function: log
##
## Formula:
## ES50_sha ~ s(Latitude, Longitude, bs = "sos")
##
## Parametric coefficients:
##             Estimate Std. Error z value Pr(>|z|)
## (Intercept)  3.63512    0.01366     266   <2e-16 ***
## ---
## Signif. codes:  0 '***' 0.001 '**' 0.01 '*' 0.05 '.' 0.1 ' ' 1
##
## Approximate significance of smooth terms:
##                         edf Ref.df Chi.sq p-value
## s(Latitude,Longitude) 26.52     49  118.3  <2e-16 ***
## ---
## Signif. codes:  0 '***' 0.001 '**' 0.01 '*' 0.05 '.' 0.1 ' ' 1
##
## R-sq.(adj) =  0.314   Deviance explained =   36%
## -REML = 975.53  Scale est. = 1         n = 264
```

```
shallow.ES50.depth <- gam(ES50_sha ~ s(Latitude, Longitude, bs = "sos") +
s(Depth_Mean), data = Ecological_Data_Global_Hex_sp, family = "nb", metho
d = "REML", select = TRUE)
gam.check(shallow.ES50.depth)
```

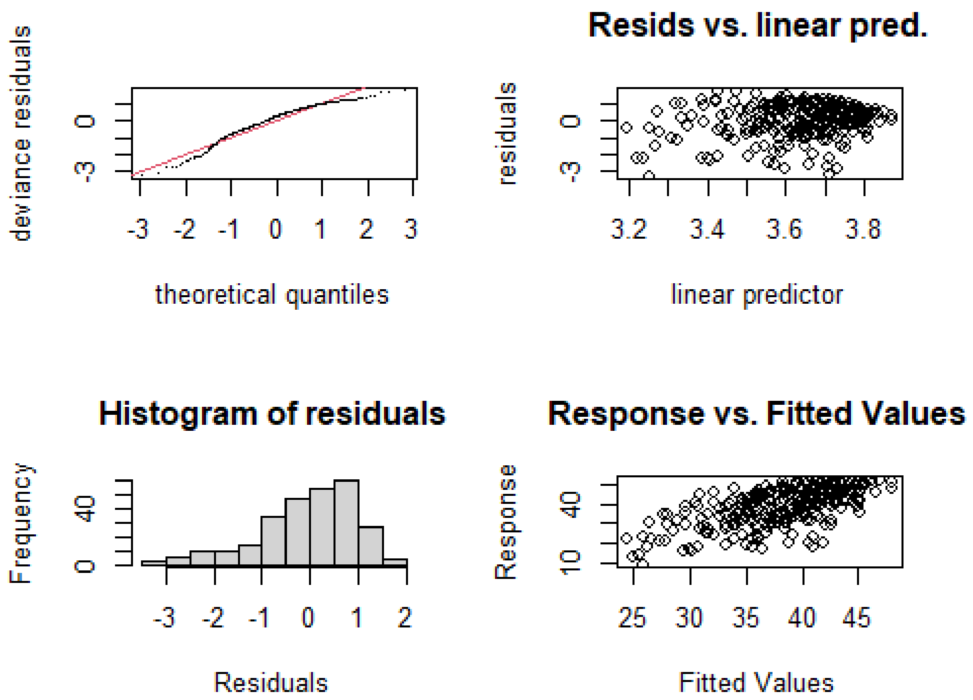


```
##
## Method: REML   Optimizer: outer newton
## full convergence after 9 iterations.
## Gradient range [-0.0003564383,0.0002138149]
## (score 975.0992 & scale 1).
## Hessian positive definite, eigenvalue range [0.0003577406,20.20401].
## Model rank =  59 / 59
##
## Basis dimension (k) checking results. Low p-value (k-index<1) may
## indicate that k is too low, especially if edf is close to k'.
##
##                           k'   edf k-index p-value
## s(Latitude,Longitude) 49.00 26.69    0.97    0.29
## s(Depth_Mean)          9.00  1.38    0.97    0.28
```

```
summary(shallow.ES50.depth)
```

```
##
## Family: Negative Binomial(45.717)
## Link function: log
##
## Formula:
## ES50_sha ~ s(Latitude, Longitude, bs = "sos") + s(Depth_Mean)
##
## Parametric coefficients:
##             Estimate Std. Error z value Pr(>|z|)
## (Intercept)  3.63480    0.01355   268.2   <2e-16 ***
## ---
## Signif. codes:  0 '***' 0.001 '**' 0.01 '*' 0.05 '.' 0.1 ' ' 1
##
```

```
## Approximate significance of smooth terms:
##                              edf Ref.df   Chi.sq p-value    
## s(Latitude,Longitude) 26.686     49 114.962  <2e-16 ***
## s(Depth_Mean)          1.376      9   3.116  0.0664 .  
## ---
## Signif. codes:  0 '***' 0.001 '**' 0.01 '*' 0.05 '.' 0.1 ' ' 1
## 
## R-sq.(adj) =  0.325   Deviance explained = 37.3%
## -REML =  975.1  Scale est. = 1         n = 264
```

```
shallow.ES50.shelf <- gam(ES50_sha ~ s(Latitude, Longitude, bs = "sos")
+ s(Shelf_Mean), data = Ecological_Data_Global_Hex_sp, family = "nb", met
hod = "REML", select = TRUE)
gam.check(shallow.ES50.shelf)
```

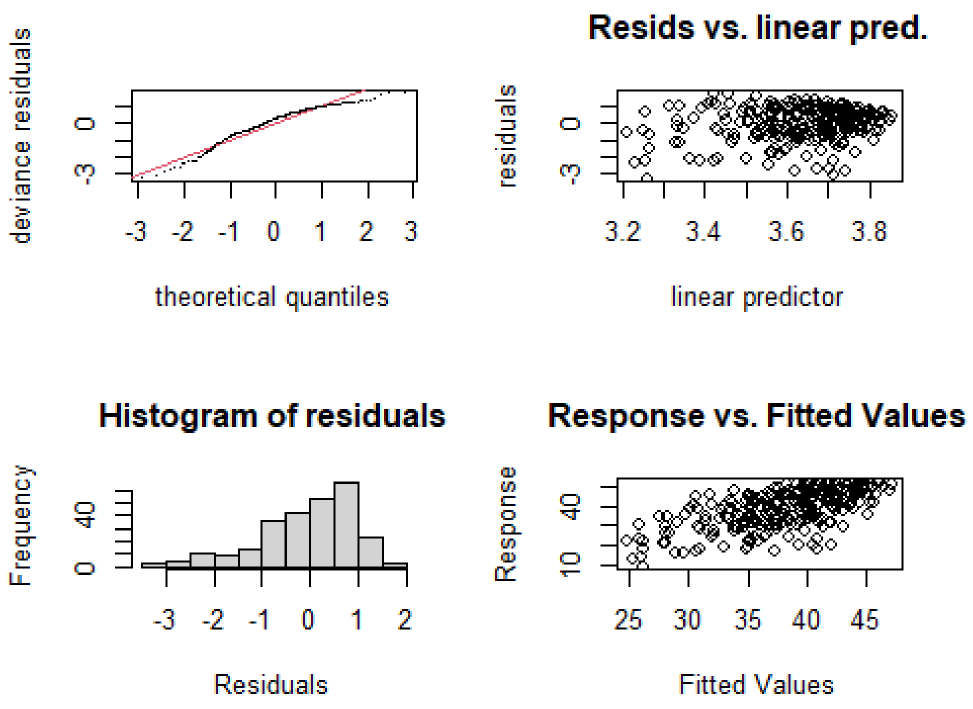


```
## 
## Method: REML   Optimizer: outer newton
## full convergence after 9 iterations.
## Gradient range [-0.0003502192,-0.0001137013]
## (score 975.5112 & scale 1).
## Hessian positive definite, eigenvalue range [0.0003500272,21.23683].
## Model rank =  59 / 59 
## 
## Basis dimension (k) checking results. Low p-value (k-index<1) may
## indicate that k is too low, especially if edf is close to k'.
## 
##                           k'    edf k-index p-value
## s(Latitude,Longitude) 49.000 26.478    0.97    0.27
## s(Shelf_Mean)          9.000  0.227    0.98    0.42
```

```
summary(shallow.ES50.shelf)

##
## Family: Negative Binomial(44.125)
## Link function: log
##
## Formula:
## ES50_sha ~ s(Latitude, Longitude, bs = "sos") + s(Shelf_Mean)
##
## Parametric coefficients:
##             Estimate Std. Error z value Pr(>|z|)
## (Intercept)  3.63511    0.01366   266.1   <2e-16 ***
## ---
## Signif. codes:  0 '***' 0.001 '**' 0.01 '*' 0.05 '.' 0.1 ' ' 1
##
## Approximate significance of smooth terms:
##                            edf Ref.df  Chi.sq p-value
## s(Latitude,Longitude) 26.4779     49 115.094  <2e-16 ***
## s(Shelf_Mean)          0.2274      9   0.288   0.237
## ---
## Signif. codes:  0 '***' 0.001 '**' 0.01 '*' 0.05 '.' 0.1 ' ' 1
##
## R-sq.(adj) =  0.315   Deviance explained = 36.1%
## -REML = 975.51  Scale est. = 1         n = 264

shallow.ES50.current <- gam(ES50_sha ~ s(Latitude, Longitude, bs = "sos")
+ s(CurVel_Mean), data = Ecological_Data_Global_Hex_sp, family = "nb", me
thod = "REML", select = TRUE)
gam.check(shallow.ES50.current)
```

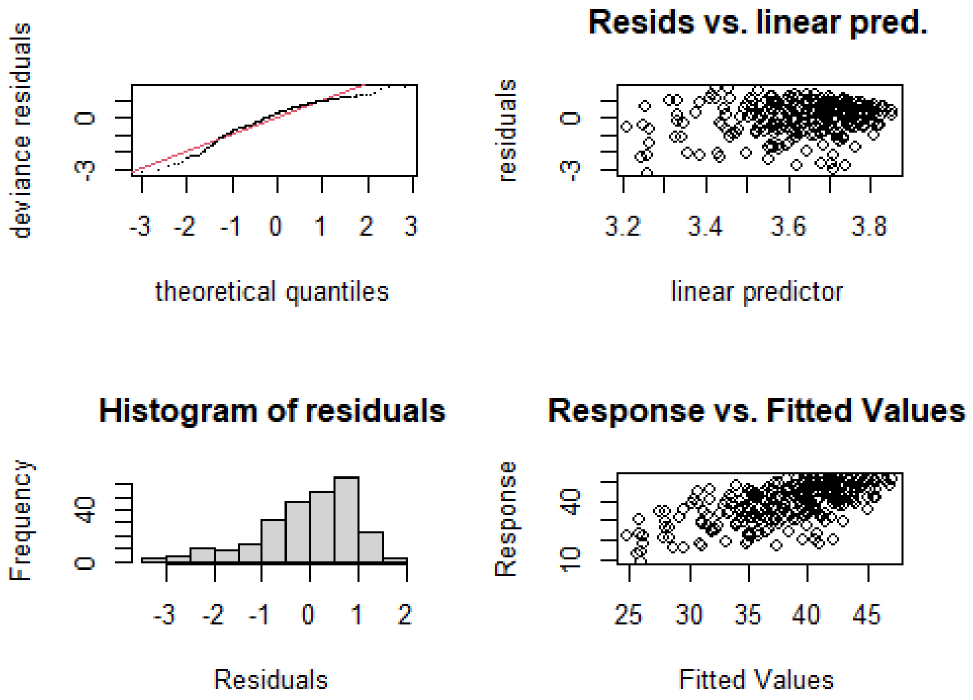

```
##
## Method: REML   Optimizer: outer newton
## full convergence after 10 iterations.
## Gradient range [-0.0001343351,0.0001043865]
## (score 975.5279 & scale 1).
## Hessian positive definite, eigenvalue range [8.22051e-05,21.30118].
## Model rank =  59 / 59
##
## Basis dimension (k) checking results. Low p-value (k-index<1) may
## indicate that k is too low, especially if edf is close to k'.
##
##                              k'      edf k-index p-value
## s(Latitude,Longitude) 4.90e+01 2.65e+01    0.97    0.27
## s(CurVel_Mean)        9.00e+00 6.63e-04    1.00    0.47

summary(shallow.ES50.current)

##
## Family: Negative Binomial(44.052)
## Link function: log
##
## Formula:
## ES50_sha ~ s(Latitude, Longitude, bs = "sos") + s(CurVel_Mean)
##
## Parametric coefficients:
##             Estimate Std. Error z value Pr(>|z|)
## (Intercept)  3.63512    0.01366     266   <2e-16 ***
## ---
## Signif. codes:  0 '***' 0.001 '**' 0.01 '*' 0.05 '.' 0.1 ' ' 1
##
## Approximate significance of smooth terms:
##                             edf Ref.df Chi.sq p-value
## s(Latitude,Longitude) 2.652e+01     49  118.3  <2e-16 ***
## s(CurVel_Mean)        6.633e-04      9    0.0   0.592
## ---
## Signif. codes:  0 '***' 0.001 '**' 0.01 '*' 0.05 '.' 0.1 ' ' 1
##
## R-sq.(adj) =  0.314   Deviance explained =   36%
## -REML = 975.53  Scale est. = 1         n = 264

shallow.ES50.humimp <- gam(ES50_sha ~ s(Latitude, Longitude, bs = "sos")
+ s(HumImp_Mean), data = Ecological_Data_Global_Hex_sp, family = "nb", me
thod = "REML", select = TRUE)
gam.check(shallow.ES50.humimp)
```

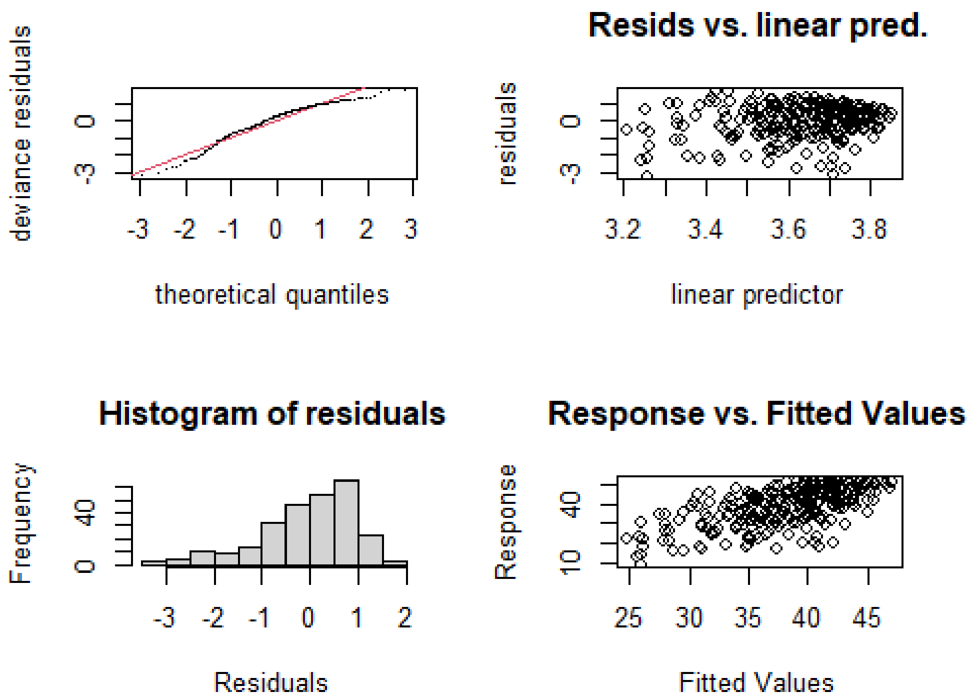


```
##
## Method: REML   Optimizer: outer newton
## full convergence after 9 iterations.
## Gradient range [-0.0004110335,-2.327894e-05]
## (score 975.5277 & scale 1).
## Hessian positive definite, eigenvalue range [0.0002576693,21.29664].
## Model rank =  59 / 59
##
## Basis dimension (k) checking results. Low p-value (k-index<1) may
## indicate that k is too low, especially if edf is close to k'.
##
##                             k'    edf k-index p-value
## s(Latitude,Longitude) 49.000 26.507    0.97    0.28
## s(HumImp_Mean)          9.000  0.032    1.10    0.98
```

```
summary(shallow.ES50.humimp)
```

```
##
## Family: Negative Binomial(44.054)
## Link function: log
##
## Formula:
## ES50_sha ~ s(Latitude, Longitude, bs = "sos") + s(HumImp_Mean)
##
## Parametric coefficients:
##             Estimate Std. Error z value Pr(>|z|)
## (Intercept)  3.63512    0.01366   266.1   <2e-16 ***
## ---
## Signif. codes:  0 '***' 0.001 '**' 0.01 '*' 0.05 '.' 0.1 ' ' 1
##
```

```
## Approximate significance of smooth terms:
##                               edf Ref.df  Chi.sq p-value
## s(Latitude,Longitude) 26.50723     49 117.693  <2e-16 ***
## s(HumImp_Mean)         0.03205      9   0.032   0.254
## ---
## Signif. codes:  0 '***' 0.001 '**' 0.01 '*' 0.05 '.' 0.1 ' ' 1
##
## R-sq.(adj) =  0.314   Deviance explained =   36%
## -REML = 975.53  Scale est. = 1         n = 264
```

```
shallow.ES50.icecover <- gam(ES50_sha ~ s(Latitude, Longitude, bs = "sos"
) + s(IceCov_Mean), data = Ecological_Data_Global_Hex_sp, family = "nb",
method = "REML", select = TRUE)
gam.check(shallow.ES50.icecover)
```

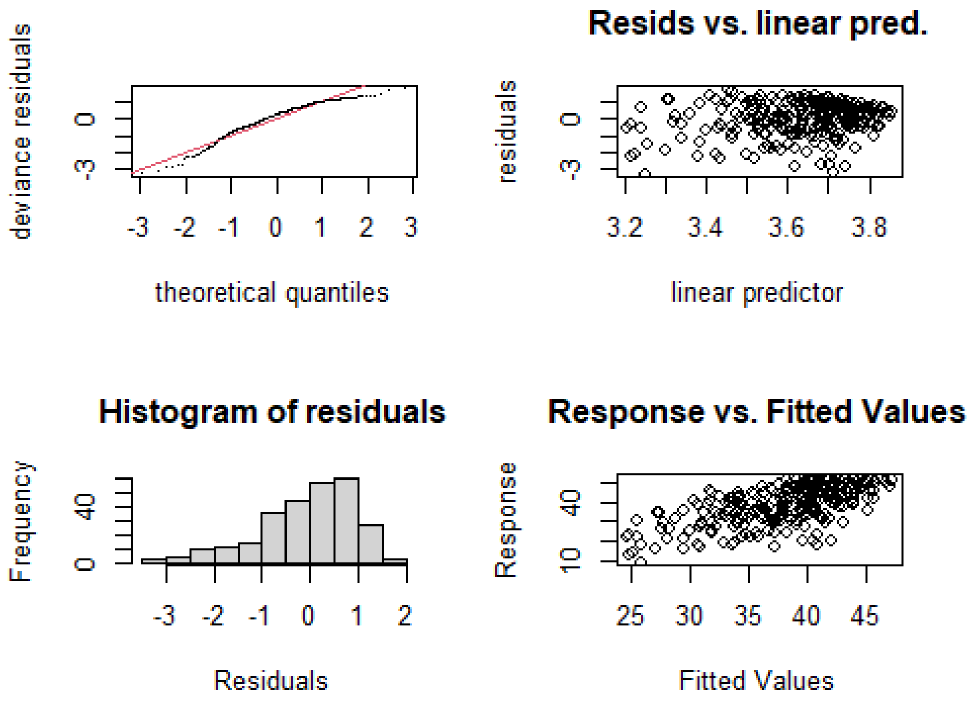


```
##
## Method: REML   Optimizer: outer newton
## full convergence after 8 iterations.
## Gradient range [-5.931489e-05,1.764955e-05]
## (score 974.3406 & scale 1).
## Hessian positive definite, eigenvalue range [5.930695e-05,19.85275].
## Model rank =  59 / 59
##
## Basis dimension (k) checking results. Low p-value (k-index<1) may
## indicate that k is too low, especially if edf is close to k'.
##
##                          k'   edf k-index p-value
## s(Latitude,Longitude) 49.00 26.74    0.97    0.25
## s(IceCov_Mean)         9.00  1.33    1.02    0.61
```

```
summary(shallow.ES50.icecover)

##
## Family: Negative Binomial(46.569)
## Link function: log
##
## Formula:
## ES50_sha ~ s(Latitude, Longitude, bs = "sos") + s(IceCov_Mean)
##
## Parametric coefficients:
##             Estimate Std. Error z value Pr(>|z|)
## (Intercept)   3.6346     0.0135   269.3   <2e-16 ***
## ---
## Signif. codes:  0 '***' 0.001 '**' 0.01 '*' 0.05 '.' 0.1 ' ' 1
##
## Approximate significance of smooth terms:
##                            edf Ref.df  Chi.sq p-value
## s(Latitude,Longitude) 26.740     49 122.483 < 2e-16 ***
## s(IceCov_Mean)         1.331      9   4.546 0.00631 **
## ---
## Signif. codes:  0 '***' 0.001 '**' 0.01 '*' 0.05 '.' 0.1 ' ' 1
##
## R-sq.(adj) =  0.329   Deviance explained = 37.8%
## -REML = 974.34  Scale est. = 1         n = 264

shallow.ES50.nitrate <- gam(ES50_sha ~ s(Latitude, Longitude, bs = "sos")
+ s(Nitrate_Mean), data = Ecological_Data_Global_Hex_sp, family = "nb", m
ethod = "REML", select = TRUE)
gam.check(shallow.ES50.nitrate)
```

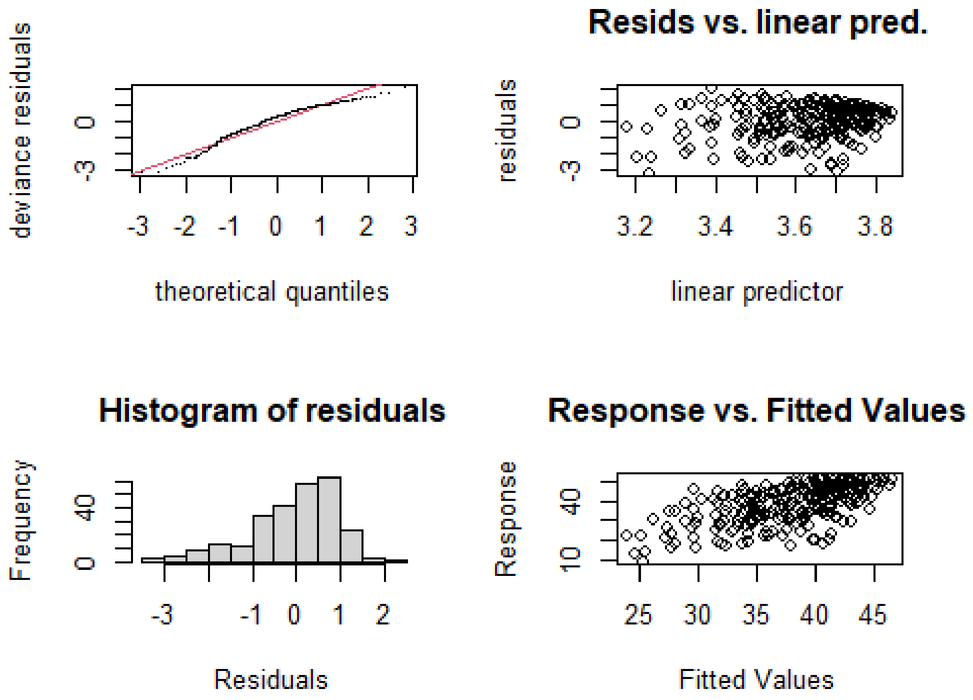

```
##
## Method: REML   Optimizer: outer newton
## full convergence after 7 iterations.
## Gradient range [-5.182447e-05,1.372934e-06]
## (score 974.5901 & scale 1).
## Hessian positive definite, eigenvalue range [5.181594e-05,21.75401].
## Model rank =  59 / 59
##
## Basis dimension (k) checking results. Low p-value (k-index<1) may
## indicate that k is too low, especially if edf is close to k'.
##
##                          k'   edf k-index p-value
## s(Latitude,Longitude) 49.00 23.88    0.97   0.280
## s(Nitrate_Mean)        9.00  1.64    0.92   0.085 .
## ---
## Signif. codes:  0 '***' 0.001 '**' 0.01 '*' 0.05 '.' 0.1 ' ' 1
```

```
summary(shallow.ES50.nitrate)
```

```
##
## Family: Negative Binomial(43.179)
## Link function: log
##
## Formula:
## ES50_sha ~ s(Latitude, Longitude, bs = "sos") + s(Nitrate_Mean)
##
## Parametric coefficients:
##             Estimate Std. Error z value Pr(>|z|)
## (Intercept)  3.63531    0.01373   264.8   <2e-16 ***
## ---
## Signif. codes:  0 '***' 0.001 '**' 0.01 '*' 0.05 '.' 0.1 ' ' 1
##
## Approximate significance of smooth terms:
##                          edf Ref.df Chi.sq p-value
## s(Latitude,Longitude) 23.882     49 70.805  <2e-16 ***
## s(Nitrate_Mean)        1.635      9  5.348  0.0166 *
## ---
## Signif. codes:  0 '***' 0.001 '**' 0.01 '*' 0.05 '.' 0.1 ' ' 1
##
## R-sq.(adj) =  0.309   Deviance explained = 35.2%
## -REML = 974.59  Scale est. = 1         n = 264
```

```
shallow.ES50.PrimProd <- gam(ES50_sha ~ s(Latitude, Longitude, bs = "sos"
) + s(PrimProd_Mean), data = Ecological_Data_Global_Hex_sp, family = "nb"
, method = "REML", select = TRUE)
gam.check(shallow.ES50.PrimProd)
```

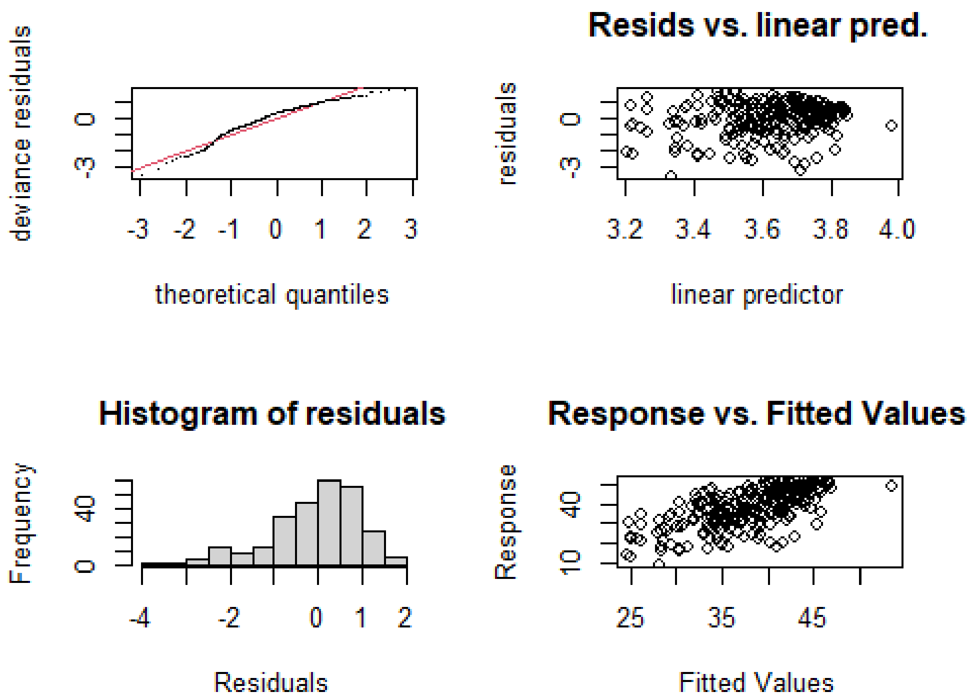


```
##
## Method: REML   Optimizer: outer newton
## full convergence after 13 iterations.
## Gradient range [-4.776461e-05,5.800716e-05]
## (score 969.6809 & scale 1).
## Hessian positive definite, eigenvalue range [0.1413407,18.05408].
## Model rank =  59 / 59
##
## Basis dimension (k) checking results. Low p-value (k-index<1) may
## indicate that k is too low, especially if edf is close to k'.
##
##                           k'  edf k-index p-value
## s(Latitude,Longitude) 49.0 23.8    0.96    0.22
## s(PrimProd_Mean)       9.0  3.3    0.98    0.32
```

```
summary(shallow.ES50.PrimProd)
```

```
##
## Family: Negative Binomial(50.207)
## Link function: log
##
## Formula:
## ES50_sha ~ s(Latitude, Longitude, bs = "sos") + s(PrimProd_Mean)
##
## Parametric coefficients:
##             Estimate Std. Error z value Pr(>|z|)
## (Intercept)  3.63404    0.01328   273.6   <2e-16 ***
## ---
## Signif. codes:  0 '***' 0.001 '**' 0.01 '*' 0.05 '.' 0.1 ' ' 1
##
```

```
## Approximate significance of smooth terms:
##                              edf Ref.df Chi.sq p-value
## s(Latitude,Longitude) 23.758     49  86.99 < 2e-16 ***
## s(PrimProd_Mean)       3.305      9  22.39 9.9e-06 ***
## ---
## Signif. codes:  0 '***' 0.001 '**' 0.01 '*' 0.05 '.' 0.1 ' ' 1
##
## R-sq.(adj) =  0.351   Deviance explained = 39.1%
## -REML = 969.68  Scale est. = 1         n = 264
```

```
shallow.ES50.ThemM <- gam(ES50_sha ~ s(Latitude, Longitude, bs = "sos") +
s(ThemM_mean), data = Ecological_Data_Global_Hex_sp, family = "nb", metho
d = "REML", select = TRUE)
gam.check(shallow.ES50.ThemM)
```

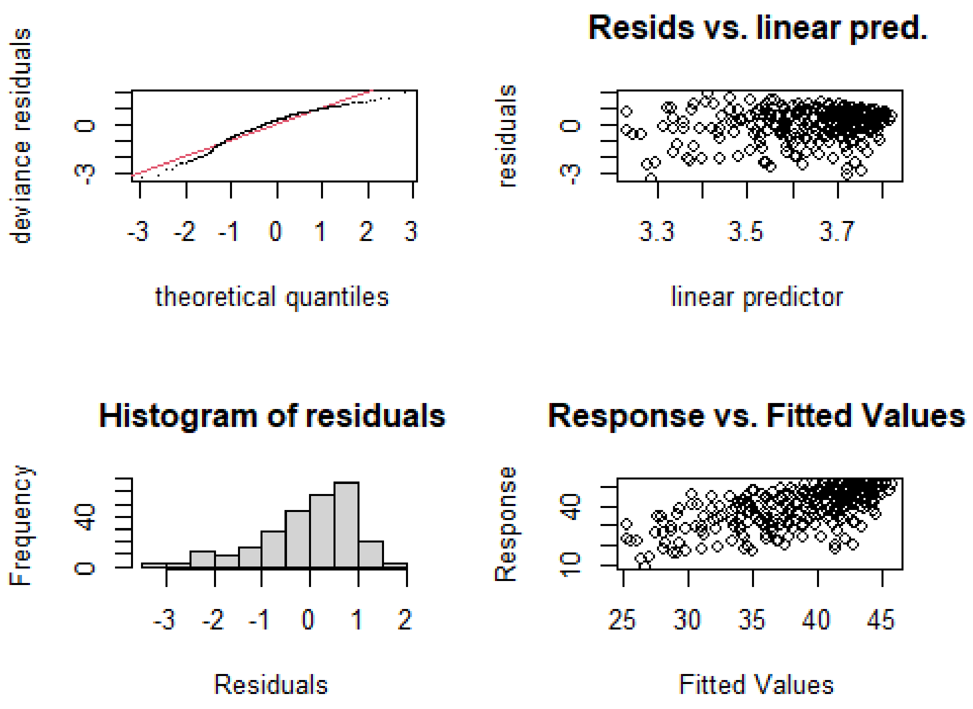


```
##
## Method: REML   Optimizer: outer newton
## full convergence after 6 iterations.
## Gradient range [-6.870579e-05,3.428804e-08]
## (score 970.2945 & scale 1).
## Hessian positive definite, eigenvalue range [3.19143e-05,22.05881].
## Model rank =  59 / 59
##
## Basis dimension (k) checking results. Low p-value (k-index<1) may
## indicate that k is too low, especially if edf is close to k'.
##
##                          k'   edf k-index p-value
## s(Latitude,Longitude) 49.00 19.34    0.94    0.14
## s(ThemM_mean)          9.00  2.31    1.00    0.44
```

```
summary(shallow.ES50.ThemM)

##
## Family: Negative Binomial(43.168)
## Link function: log
##
## Formula:
## ES50_sha ~ s(Latitude, Longitude, bs = "sos") + s(ThemM_mean)
##
## Parametric coefficients:
##             Estimate Std. Error z value Pr(>|z|)
## (Intercept)  3.63531    0.01373   264.8   <2e-16 ***
## ---
## Signif. codes:  0 '***' 0.001 '**' 0.01 '*' 0.05 '.' 0.1 ' ' 1
##
## Approximate significance of smooth terms:
##                          edf Ref.df Chi.sq  p-value
## s(Latitude,Longitude) 19.34     49  52.97 1.69e-06 ***
## s(ThemM_mean)          2.31      9  18.60  < 2e-16 ***
## ---
## Signif. codes:  0 '***' 0.001 '**' 0.01 '*' 0.05 '.' 0.1 ' ' 1
##
## R-sq.(adj) =  0.308   Deviance explained = 34.1%
## -REML = 970.29  Scale est. = 1         n = 264

shallow.ES50.ThemR <- gam(ES50_sha ~ s(Latitude, Longitude, bs = "sos") +
s(ThemR_mean), data = Ecological_Data_Global_Hex_sp, family = "nb", metho
d = "REML", select = TRUE)
gam.check(shallow.ES50.ThemR)
```

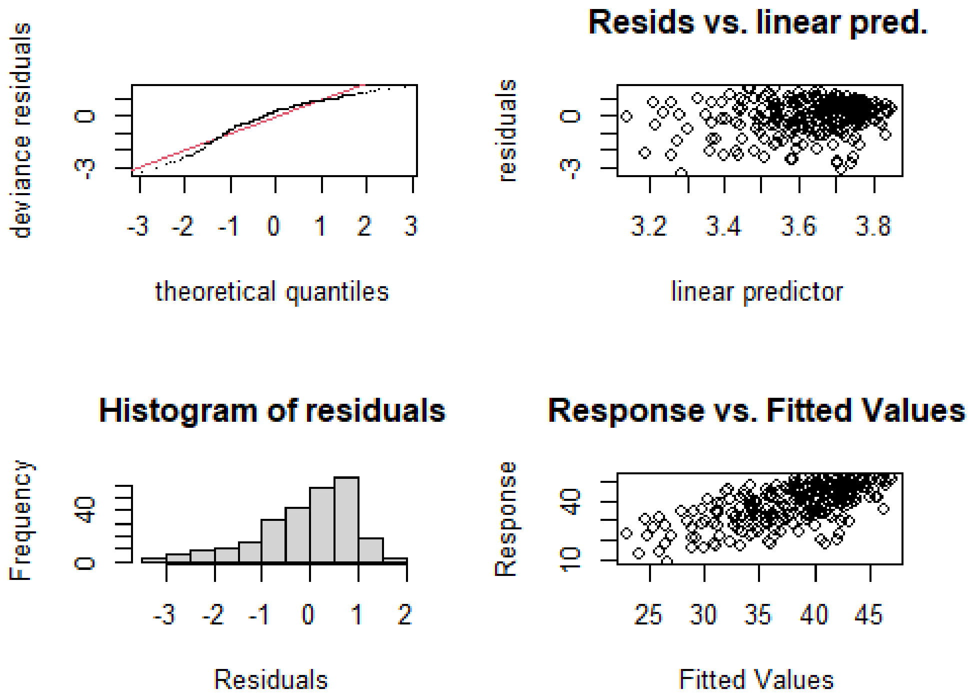

```
##
## Method: REML   Optimizer: outer newton
## full convergence after 6 iterations.
## Gradient range [-5.902724e-05,1.054204e-05]
## (score 972.6389 & scale 1).
## Hessian positive definite, eigenvalue range [3.845812e-06,19.54429].
## Model rank =  59 / 59
##
## Basis dimension (k) checking results. Low p-value (k-index<1) may
## indicate that k is too low, especially if edf is close to k'.
##
##                             k'   edf k-index p-value
## s(Latitude,Longitude) 49.00 25.16    0.98    0.28
## s(ThemR_mean)          9.00  2.09    0.91    0.04 *
## ---
## Signif. codes:  0 '***' 0.001 '**' 0.01 '*' 0.05 '.' 0.1 ' ' 1
```

```
summary(shallow.ES50.ThemR)
```

```
##
## Family: Negative Binomial(47.079)
## Link function: log
##
## Formula:
## ES50_sha ~ s(Latitude, Longitude, bs = "sos") + s(ThemR_mean)
##
## Parametric coefficients:
##             Estimate Std. Error z value Pr(>|z|)
## (Intercept)  3.63450    0.01347   269.9   <2e-16 ***
## ---
## Signif. codes:  0 '***' 0.001 '**' 0.01 '*' 0.05 '.' 0.1 ' ' 1
##
## Approximate significance of smooth terms:
##                          edf Ref.df Chi.sq  p-value
## s(Latitude,Longitude) 25.16     49  94.48  < 2e-16 ***
## s(ThemR_mean)          2.09      9  10.21 0.000394 ***
## ---
## Signif. codes:  0 '***' 0.001 '**' 0.01 '*' 0.05 '.' 0.1 ' ' 1
##
## R-sq.(adj) =  0.332   Deviance explained = 37.8%
## -REML = 972.64  Scale est. = 1         n = 264
```

```
shallow.ES50.env <- gam(ES50_sha ~ s(Latitude, Longitude, bs = "sos") + s
(Depth_Mean) + s(Shelf_Mean) + s(CurVel_Mean) + s(HumImp_Mean) + s(IceCov
_Mean) + s(Nitrate_Mean) + s(PrimProd_Mean) + s(ThemM_mean) + s(ThemR_mea
n), data = Ecological_Data_Global_Hex_sp, family = "nb", method = "REML",
select = TRUE)
gam.check(shallow.ES50.env)
```

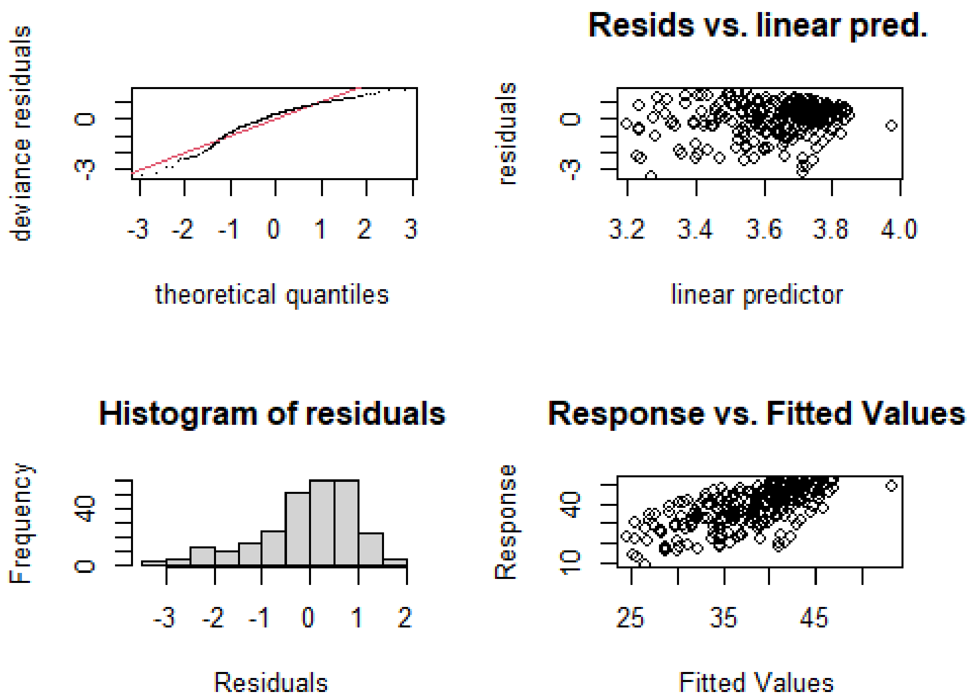


```
##
## Method: REML   Optimizer: outer newton
## full convergence after 11 iterations.
## Gradient range [-0.00127991,0.0006144376]
## (score 966.7847 & scale 1).
## Hessian positive definite, eigenvalue range [1.111933e-06,16.88596].
## Model rank =  131 / 131
##
## Basis dimension (k) checking results. Low p-value (k-index<1) may
## indicate that k is too low, especially if edf is close to k'.
##
##                            k'      edf k-index p-value
## s(Latitude,Longitude) 4.90e+01 1.99e+01    0.94    0.12
## s(Depth_Mean)         9.00e+00 1.70e+00    0.97    0.33
## s(Shelf_Mean)         9.00e+00 2.04e-04    0.98    0.42
## s(CurVel_Mean)        9.00e+00 2.06e-04    1.00    0.50
## s(HumImp_Mean)        9.00e+00 2.72e-04    1.11    0.98
## s(IceCov_Mean)        9.00e+00 1.36e+00    1.04    0.70
## s(Nitrate_Mean)       9.00e+00 1.83e-02    0.94    0.15
## s(PrimProd_Mean)      9.00e+00 2.12e+00    0.98    0.48
## s(ThemM_mean)         9.00e+00 2.00e+00    1.00    0.46
## s(ThemR_mean)         9.00e+00 2.47e-01    0.91    0.09 .
## ---
## Signif. codes:  0 '***' 0.001 '**' 0.01 '*' 0.05 '.' 0.1 ' ' 1
```

```
summary(shallow.ES50.env)
```

```
##
## Family: Negative Binomial(52.262)
## Link function: log
```

```
##
## Formula:
## ES50_sha ~ s(Latitude, Longitude, bs = "sos") + s(Depth_Mean) +
##     s(Shelf_Mean) + s(CurVel_Mean) + s(HumImp_Mean) + s(IceCov_Mean) +
##     s(Nitrate_Mean) + s(PrimProd_Mean) + s(ThemM_mean) + s(ThemR_mean)
##
## Parametric coefficients:
##             Estimate Std. Error z value Pr(>|z|)
## (Intercept)  3.63375    0.01317   275.9   <2e-16 ***
## ---
## Signif. codes:  0 '***' 0.001 '**' 0.01 '*' 0.05 '.' 0.1 ' ' 1
##
## Approximate significance of smooth terms:
##                             edf Ref.df Chi.sq  p-value
## s(Latitude,Longitude) 1.985e+01     49 53.430 1.28e-06 ***
## s(Depth_Mean)         1.700e+00      9  4.730  0.02990 *
## s(Shelf_Mean)         2.039e-04      9  0.000  0.55513
## s(CurVel_Mean)        2.057e-04      9  0.000  0.72521
## s(HumImp_Mean)        2.718e-04      9  0.000  0.41089
## s(IceCov_Mean)        1.356e+00      9  3.015  0.03872 *
## s(Nitrate_Mean)       1.829e-02      9  0.017  0.30021
## s(PrimProd_Mean)      2.117e+00      9 10.168  0.00198 **
## s(ThemM_mean)         1.996e+00      9 11.519 6.56e-07 ***
## s(ThemR_mean)         2.469e-01      9  0.270  0.21294
## ---
## Signif. codes:  0 '***' 0.001 '**' 0.01 '*' 0.05 '.' 0.1 ' ' 1
##
## R-sq.(adj) =  0.362   Deviance explained =   40%
## -REML = 966.78  Scale est. = 1         n = 264
```

```
shallow.ES50.models <- list(Intercept = shallow.ES50.intercept,
                            LatLon = shallow.ES50.latlon,
                            Depth = shallow.ES50.depth,
                            ConShe= shallow.ES50.shelf,
                            CurVel = shallow.ES50.current,
                            HumImp = shallow.ES50.humimp,
                            IceCov = shallow.ES50.icecover,
                            Nitrate = shallow.ES50.nitrate,
                            PriPro = shallow.ES50.PrimProd,
                            TemMea = shallow.ES50.ThemM,
                            TheRan = shallow.ES50.ThemR,
                            Environment = shallow.numsp.env)
shallow.ES50.aic.df <- data.frame(Model = names(shallow.ES50.models),
                                  AIC = sapply(shallow.ES50.models, funct
ion(x) x$aic),
                                  akaike.weights(sapply(shallow.ES50.mode
ls, function(x) x$aic)))

shallow.ES50.aic.df <- shallow.ES50.aic.df[order(shallow.ES50.aic.df$AIC)
,]
shallow.ES50.aic.df$Cumulative.Weight <- cumsum(shallow.ES50.aic.df$weigh
```

```
ts)

kable(shallow.ES50.aic.df, row.names = FALSE)
```

Model	AIC	deltaAIC	rel.LL	weights	Cumulative.Weight
PriPro	1899.768	0.000000	1.0000000	0.9358791	0.9358791
TheRan	1906.377	6.609097	0.0367158	0.0343615	0.9702406
IceCov	1908.057	8.288882	0.0158523	0.0148358	0.9850765
Depth	1910.279	10.510768	0.0052193	0.0048847	0.9899611
TemMea	1910.670	10.902093	0.0042918	0.0040166	0.9939778
ConShe	1912.843	13.075367	0.0014478	0.0013550	0.9953328
LatLon	1912.866	13.098603	0.0014311	0.0013394	0.9966721
CurVel	1912.867	13.099564	0.0014304	0.0013387	0.9980108
HumImp	1912.881	13.113020	0.0014208	0.0013297	0.9993405
Nitrate	1914.284	14.515671	0.0007046	0.0006595	1.0000000
Intercept	1983.031	83.263021	0.0000000	0.0000000	1.0000000
Environment	4161.680	2261.912309	0.0000000	0.0000000	1.0000000

```
#write.csv(shallow.ES50.aic.df, file = "shallow.ES50.aic.GAM.csv")

#Plots for ES50, shallow water
ggplot(Ecological_Data_Global_Hex_sp, aes(x = Depth_Mean, y = predict(sha
llow.ES50.depth, Ecological_Data_Global_Hex_sp))) +
  geom_smooth(method = "gam", formula = y ~ x, color = "#1a80bb", fill =
"#85bede") +  # Add a smooth dark blue line with light blue shadow
  geom_point(size = 3) +  # Add scatter plot points
  theme_bw() +  # Use the black and white theme
  labs(
    x = "Depth (m)",  # Shorten the x-axis title
    y = "Predicted Value"  # Shorten the y-axis title
  ) +
  theme(
    panel.grid.minor = element_blank(),
    panel.grid.major = element_blank(),
    axis.text.x = element_text(size =20),  # Increase x-axis text size
    axis.text.y = element_text(size = 20),  # Increase y-axis text size
    axis.title.x = element_text(size = 22), # Increase x-axis title size
    axis.title.y = element_text(size = 22)  # Increase y-axis title size
  )
```

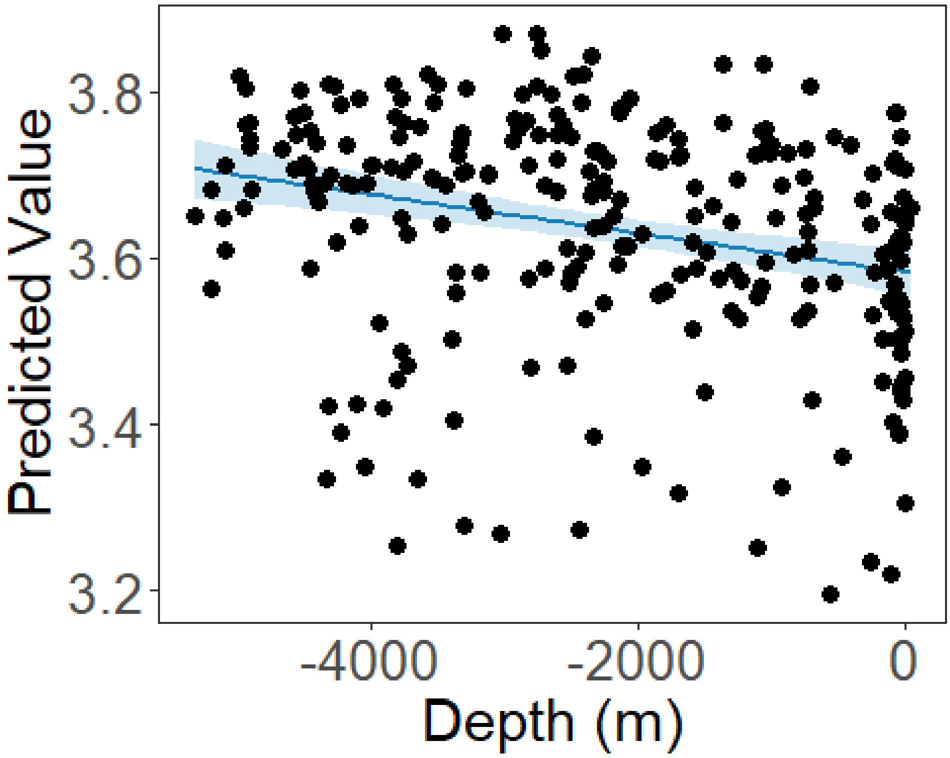


```r
ggplot(Ecological_Data_Global_Hex_sp, aes(x = Shelf_Mean, y = predict(sha
llow.ES50.shelf, Ecological_Data_Global_Hex_sp))) +
  geom_smooth(method = "gam", formula = y ~ x, color = "#1a80bb", fill =
"#85bede") +  # Add a smooth dark blue line with light blue shadow
  geom_point(size = 3) +  # Add scatter plot points
  theme_bw() +  # Use the black and white theme
  labs(
    x = "Continental Shelf (km2)",  # Shorten the x-axis title
    y = "Predicted Value"  # Shorten the y-axis title
  ) +
  theme(
    panel.grid.minor = element_blank(),
    panel.grid.major = element_blank(),
    axis.text.x = element_text(size =20),  # Increase x-axis text size
    axis.text.y = element_text(size = 20),  # Increase y-axis text size
    axis.title.x = element_text(size = 22), # Increase x-axis title size
    axis.title.y = element_text(size = 22)  # Increase y-axis title size
  )
```

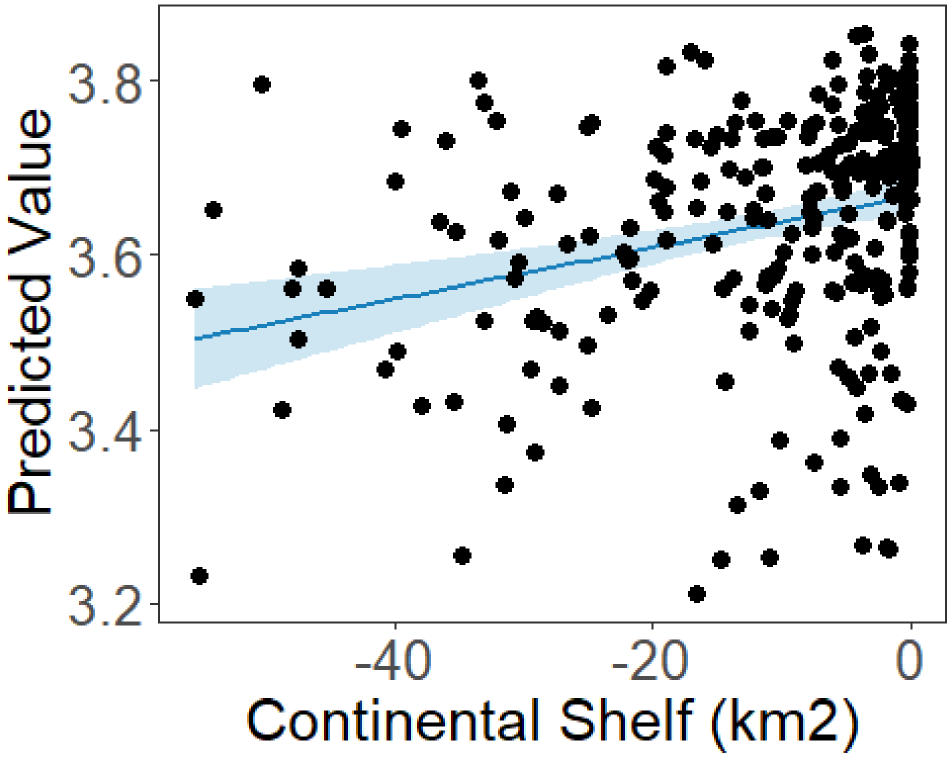


```
ggplot(Ecological_Data_Global_Hex_sp, aes(x = CurVel_Mean, y = predict(sh
allow.ES50.current, Ecological_Data_Global_Hex_sp))) +
  geom_smooth(method = "gam", formula = y ~ x, color = "#1a80bb", fill =
"#85bede") +  # Add a smooth dark blue line with light blue shadow
  geom_point(size = 3) +  # Add scatter plot points
  theme_bw() +  # Use the black and white theme
  labs(
    x = "Current Velocity (m.s-1)",  # Shorten the x-axis title
    y = "Predicted Value"  # Shorten the y-axis title
  ) +
  theme(
    panel.grid.minor = element_blank(),
    panel.grid.major = element_blank(),
    axis.text.x = element_text(size =20),  # Increase x-axis text size
    axis.text.y = element_text(size = 20),  # Increase y-axis text size
    axis.title.x = element_text(size = 22), # Increase x-axis title size
    axis.title.y = element_text(size = 22)  # Increase y-axis title size
  )
```

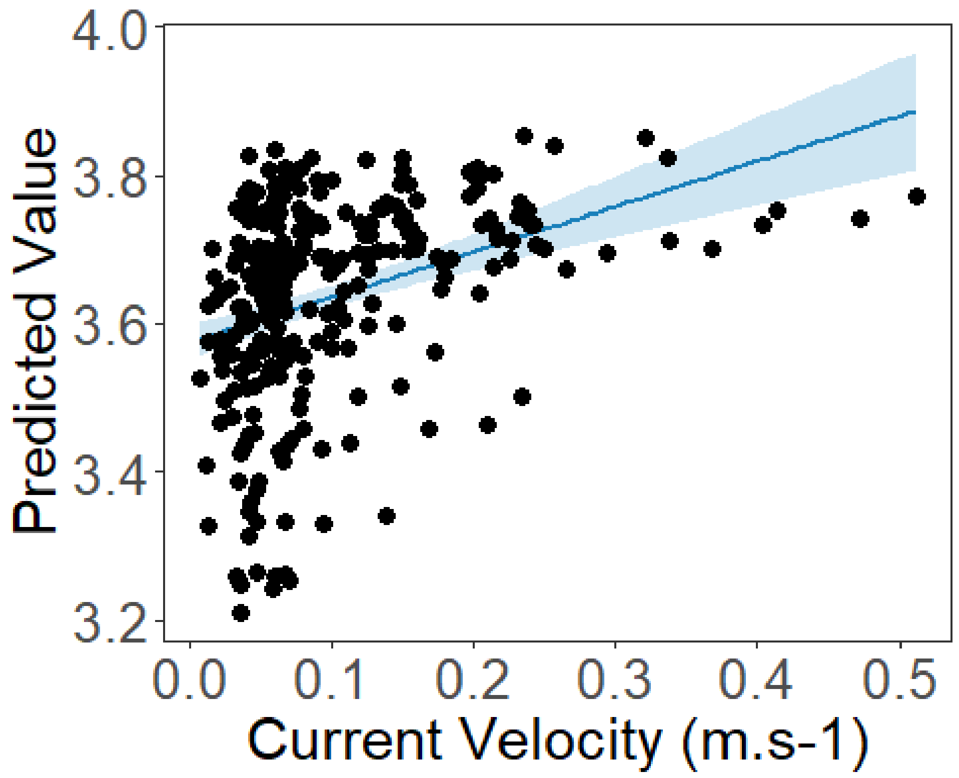


```
ggplot(Ecological_Data_Global_Hex_sp, aes(x = HumImp_Mean, y = predict(shallow.ES50.humimp, Ecological_Data_Global_Hex_sp))) +
  geom_smooth(method = "gam", formula = y ~ x, color = "#1a80bb", fill = "#85bede") +  # Add a smooth dark blue line with light blue shadow
  geom_point(size = 3) +  # Add scatter plot points
  theme_bw() +  # Use the black and white theme
  labs(
    x = "Human Impact",  # Shorten the x-axis title
    y = "Predicted Value"  # Shorten the y-axis title
  ) +
  theme(
    panel.grid.minor = element_blank(),
    panel.grid.major = element_blank(),
    axis.text.x = element_text(size =20),  # Increase x-axis text size
    axis.text.y = element_text(size = 20),  # Increase y-axis text size
    axis.title.x = element_text(size = 22), # Increase x-axis title size
    axis.title.y = element_text(size = 22)  # Increase y-axis title size
  )
```

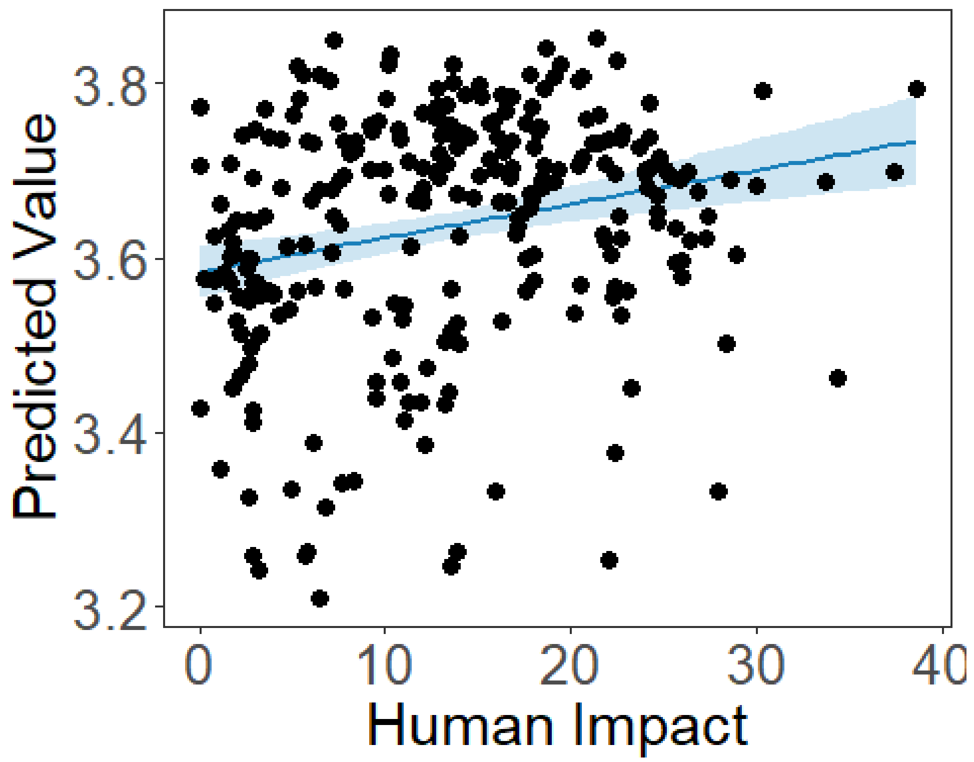


```
ggplot(Ecological_Data_Global_Hex_sp, aes(x = IceCov_Mean, y = predict(sh
allow.ES50.icecover, Ecological_Data_Global_Hex_sp))) +
  geom_smooth(method = "gam", formula = y ~ x, color = "#1a80bb", fill =
"#85bede") +  # Add a smooth dark blue line with light blue shadow
  geom_point(size = 3) +  # Add scatter plot points
  theme_bw() +  # Use the black and white theme
  labs(
    x = "Ice Cover (fraction)",  # Shorten the x-axis title
    y = "Predicted Value"  # Shorten the y-axis title
  ) +
  theme(
    panel.grid.minor = element_blank(),
    panel.grid.major = element_blank(),
    axis.text.x = element_text(size =20),  # Increase x-axis text size
    axis.text.y = element_text(size = 20),  # Increase y-axis text size
    axis.title.x = element_text(size = 22), # Increase x-axis title size
    axis.title.y = element_text(size = 22)  # Increase y-axis title size
  )
```

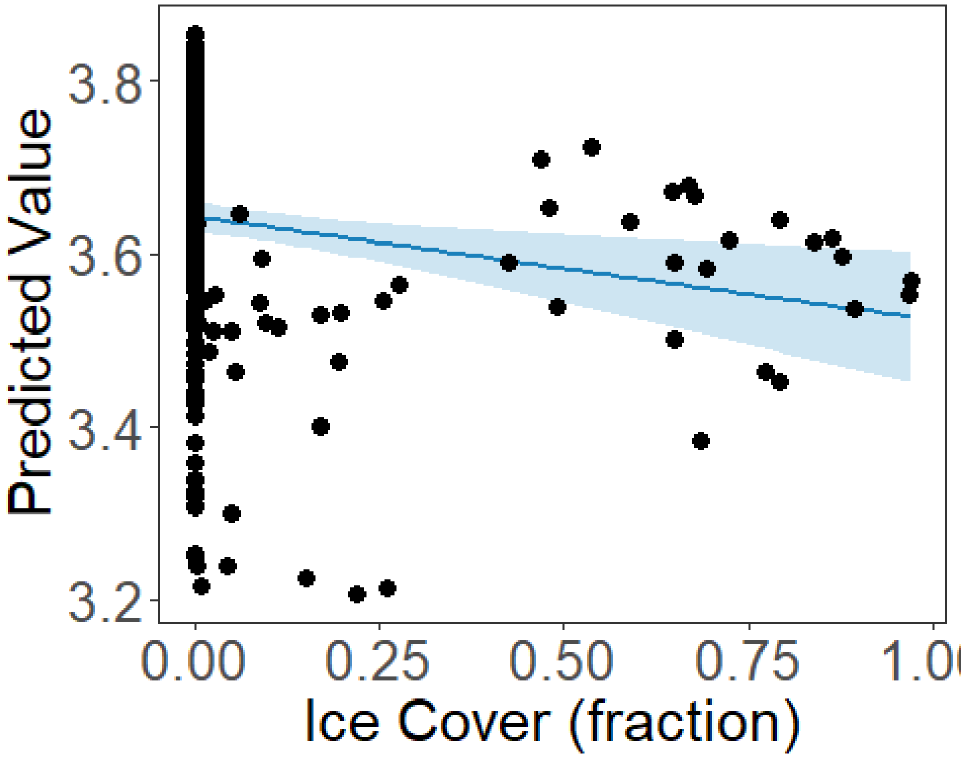


```
ggplot(Ecological_Data_Global_Hex_sp, aes(x = Nitrate_Mean, y = predict(s
hallow.ES50.nitrate, Ecological_Data_Global_Hex_sp))) +
  geom_smooth(method = "gam", formula = y ~ x, color = "#1a80bb", fill =
"#85bede") +  # Add a smooth dark blue line with light blue shadow
  geom_point(size = 3) +  # Add scatter plot points
  theme_bw() +  # Use the black and white theme
  labs(
    x = "Nitrate (mmol . m-3)",  # Shorten the x-axis title
    y = "Predicted Value"  # Shorten the y-axis title
  ) +
  theme(
    panel.grid.minor = element_blank(),
    panel.grid.major = element_blank(),
    axis.text.x = element_text(size =20),  # Increase x-axis text size
    axis.text.y = element_text(size = 20),  # Increase y-axis text size
    axis.title.x = element_text(size = 22), # Increase x-axis title size
    axis.title.y = element_text(size = 22)  # Increase y-axis title size
  )
```

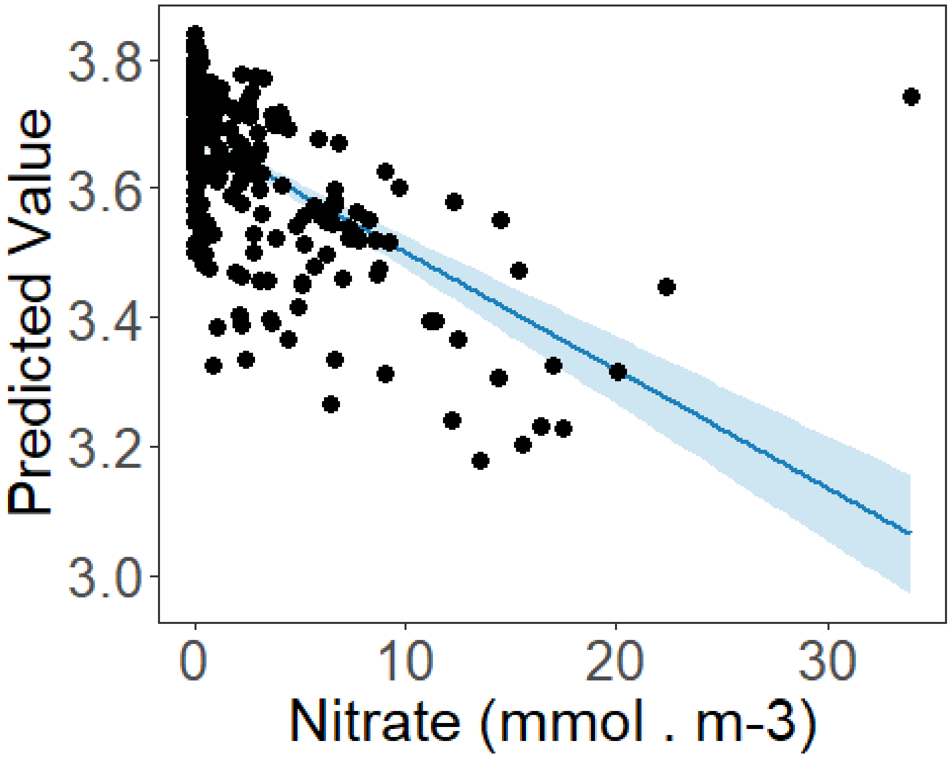


```
ggplot(Ecological_Data_Global_Hex_sp, aes(x = PrimProd_Mean, y = predict(
shallow.ES50.PrimProd, Ecological_Data_Global_Hex_sp))) +
  geom_smooth(method = "gam", formula = y ~ x, color = "#1a80bb", fill =
"#85bede") +  # Add a smooth dark blue line with light blue shadow
  geom_point(size = 3) +  # Add scatter plot points
  theme_bw() +  # Use the black and white theme
  labs(
    x = "Primary Productivity (mmol . m-3)",  # Shorten the x-axis title
    y = "Predicted Value"  # Shorten the y-axis title
  ) +
  theme(
    panel.grid.minor = element_blank(),
    panel.grid.major = element_blank(),
    axis.text.x = element_text(size =20),  # Increase x-axis text size
    axis.text.y = element_text(size = 20),  # Increase y-axis text size
    axis.title.x = element_text(size = 22), # Increase x-axis title size
    axis.title.y = element_text(size = 22)  # Increase y-axis title size
  )
```

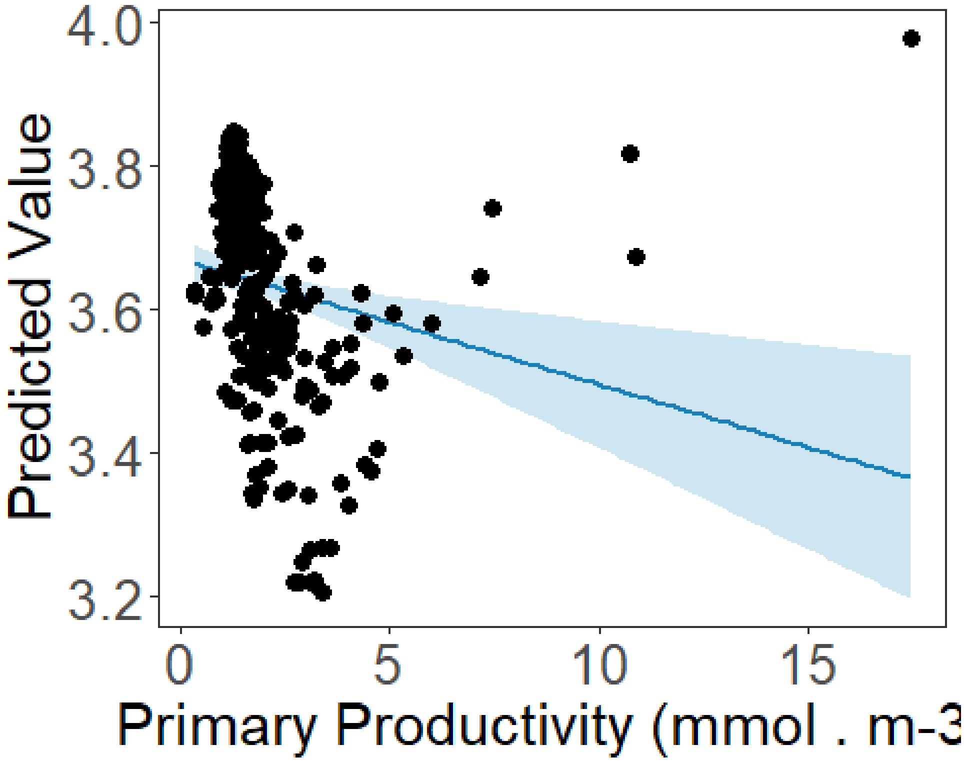


```
ggplot(Ecological_Data_Global_Hex_sp, aes(x = ThemM_mean, y = predict(sha
llow.ES50.ThemM, Ecological_Data_Global_Hex_sp))) +
  geom_smooth(method = "gam", formula = y ~ x, color = "#1a80bb", fill =
"#85bede") +  # Add a smooth dark blue line with light blue shadow
  geom_point(size = 3) +  # Add scatter plot points
  theme_bw() +  # Use the black and white theme
  labs(
    x = "Temperature Mean (ºC)",  # Shorten the x-axis title
    y = "Predicted Value"  # Shorten the y-axis title
  ) +
  theme(
    panel.grid.minor = element_blank(),
    panel.grid.major = element_blank(),
    axis.text.x = element_text(size =20),  # Increase x-axis text size
    axis.text.y = element_text(size = 20),  # Increase y-axis text size
    axis.title.x = element_text(size = 22), # Increase x-axis title size
    axis.title.y = element_text(size = 22)  # Increase y-axis title size
  )
```

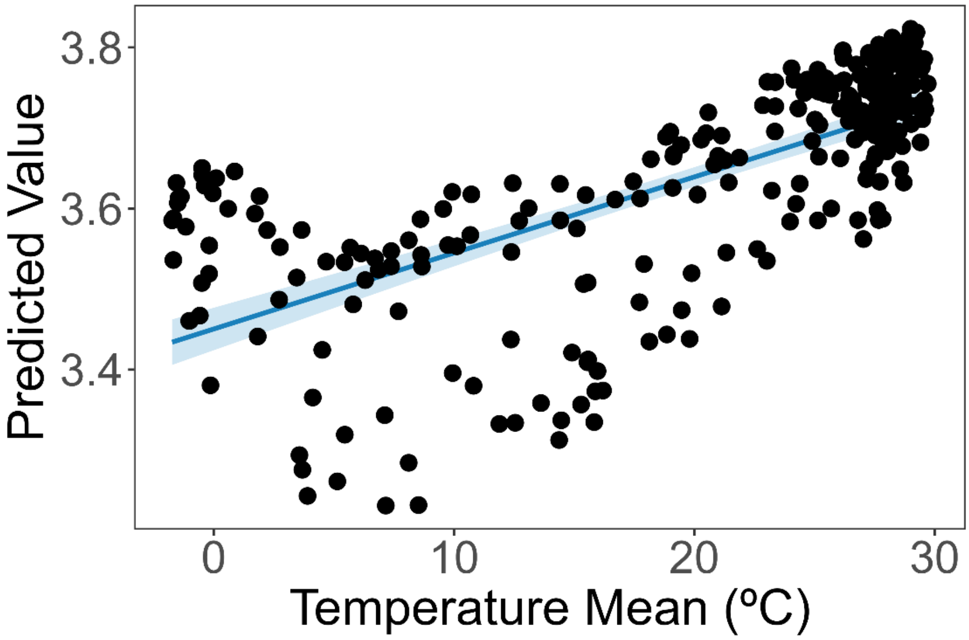


```
ggplot(Ecological_Data_Global_Hex_sp, aes(x = ThemR_mean, y = predict(sha
llow.ES50.ThemR, Ecological_Data_Global_Hex_sp))) +
  geom_smooth(method = "gam", formula = y ~ x, color = "#1a80bb", fill =
"#85bede") +  # Add a smooth dark blue line with light blue shadow
  geom_point(size = 3) +  # Add scatter plot points
  theme_bw() +  # Use the black and white theme
  labs(
    x = "Temperature Range (ºC)",  # Shorten the x-axis title
    y = "Predicted Value"  # Shorten the y-axis title
  ) +
  theme(
    panel.grid.minor = element_blank(),
    panel.grid.major = element_blank(),
    axis.text.x = element_text(size =20),  # Increase x-axis text size
    axis.text.y = element_text(size = 20),  # Increase y-axis text size
    axis.title.x = element_text(size = 22), # Increase x-axis title size
    axis.title.y = element_text(size = 22)  # Increase y-axis title size
  )
```

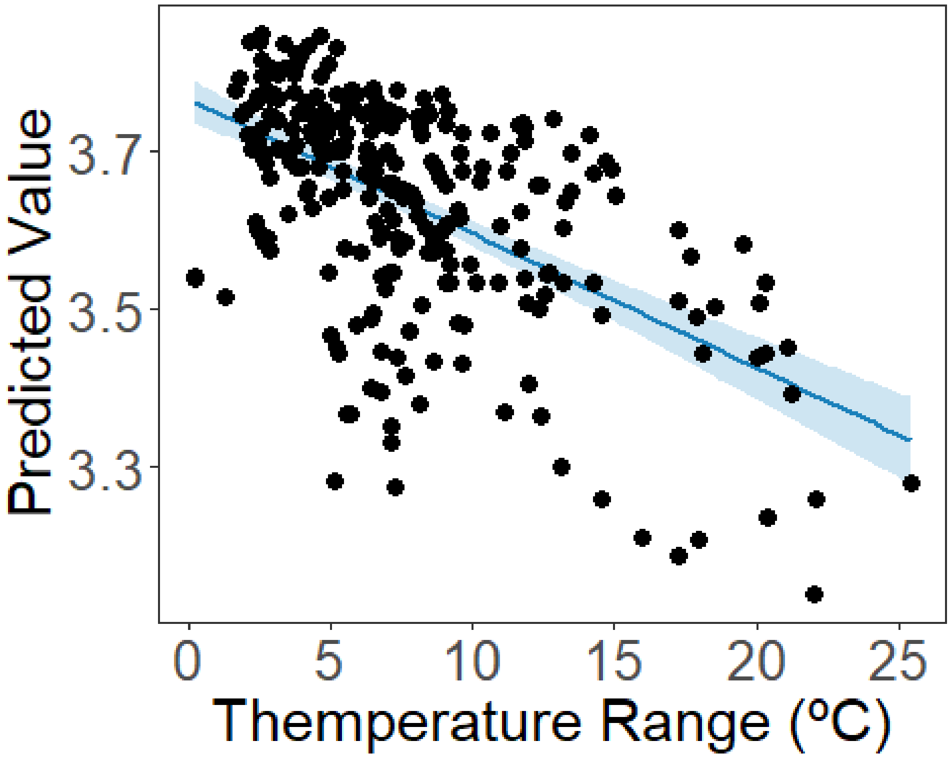

Predicted Value
3.7
3.5
3.3
0
5
10
15
20
25
Themperature Range (ºC)

Markdown_GAM_Meso.R

hsaeedi

2025-06-08

```
# ===================================
#                MESO-NUMSPe-GAM
# ===================================
library(readxl)

## Warning: package 'readxl' was built under R version 4.3.3

library(openxlsx)

## Warning: package 'openxlsx' was built under R version 4.3.3

library(tidyverse)

## Warning: package 'tidyverse' was built under R version 4.3.3

## Warning: package 'ggplot2' was built under R version 4.3.3

## Warning: package 'tibble' was built under R version 4.3.3

## Warning: package 'tidyr' was built under R version 4.3.3

## Warning: package 'readr' was built under R version 4.3.3

## Warning: package 'purrr' was built under R version 4.3.3

## Warning: package 'dplyr' was built under R version 4.3.3

## Warning: package 'stringr' was built under R version 4.3.3

## Warning: package 'forcats' was built under R version 4.3.3

## Warning: package 'lubridate' was built under R version 4.3.3

## ── Attaching core tidyverse packages ──────────────────────── tidyvers
e 2.0.0 ──
## ✔ dplyr     1.1.4     ✔ readr     2.1.5
## ✔ forcats   1.0.0     ✔ stringr   1.5.1
## ✔ ggplot2   3.5.1     ✔ tibble    3.2.1
## ✔ lubridate 1.9.3     ✔ tidyr     1.3.1
## ✔ purrr     1.0.2
## ── Conflicts ────────────────────────────────────────── tidyverse_conf
licts() ──
## ✖ dplyr::filter() masks stats::filter()
## ✖ dplyr::lag()    masks stats::lag()
## ℹ Use the conflicted package (<http://conflicted.r-lib.org/>) to for
ce all conflicts to become errors

library(sf)
```

```
## Warning: package 'sf' was built under R version 4.3.3

## Linking to GEOS 3.11.2, GDAL 3.8.2, PROJ 9.3.1; sf_use_s2() is TRUE

library(vegan)

## Warning: package 'vegan' was built under R version 4.3.3

## Loading required package: permute

## Warning: package 'permute' was built under R version 4.3.3

## Loading required package: lattice
## This is vegan 2.6-4

library(pvclust)

## Warning: package 'pvclust' was built under R version 4.3.3

library(dplyr)
library(ggplot2)
library(robis)

## Warning: package 'robis' was built under R version 4.3.3

library(obistools)
library(nortest) # for Anderson-Darling test
library(stringi) # for encoding UTF-8

## Warning: package 'stringi' was built under R version 4.3.3

library(corrplot)

## Warning: package 'corrplot' was built under R version 4.3.3

## corrplot 0.94 loaded

library(mgcv)

## Loading required package: nlme
##
## Attaching package: 'nlme'
##
## The following object is masked from 'package:dplyr':
##
##     collapse
##
## This is mgcv 1.9-0. For overview type 'help("mgcv-package")'.

library(ggeffects)

## Warning: package 'ggeffects' was built under R version 4.3.3

library(DHARMa) #simulations package gam-

## Warning: package 'DHARMa' was built under R version 4.3.3
```

```
## Warning in check_dep_version(): ABI version mismatch:
## lme4 was built with Matrix ABI version 1
## Current Matrix ABI version is 0
## Please re-install lme4 from source or restore original 'Matrix' packag
e

## This is DHARMa 0.4.6. For overview type '?DHARMa'. For recent changes,
type news(package = 'DHARMa')

library(knitr)

## Warning: package 'knitr' was built under R version 4.3.3

library(qpcR)

## Warning: package 'qpcR' was built under R version 4.3.3

## Loading required package: MASS
##
## Attaching package: 'MASS'
##
## The following object is masked from 'package:robis':
##
##     area
##
## The following object is masked from 'package:dplyr':
##
##     select
##
## Loading required package: minpack.lm

## Warning: package 'minpack.lm' was built under R version 4.3.3

## Loading required package: rgl

## Warning: package 'rgl' was built under R version 4.3.3

## Loading required package: robustbase

## Warning: package 'robustbase' was built under R version 4.3.3

## Loading required package: Matrix
##
## Attaching package: 'Matrix'
##
## The following objects are masked from 'package:tidyr':
##
##     expand, pack, unpack

#Species Counts and Environment, hexagons
Ecological_Data_Global_Hex_sp <- read.csv("Ecological_Data_Global_Hex_sp.
csv", sep = ";")

#select only numeric values
numeric_data <- Ecological_Data_Global_Hex_sp %>% select_if(is.numeric)
```

```r
#First we're going to load in our data and then trim the data frame down
to just the columns we need.
analysis.cols <- c("Latitude", "Longitude", "Depth_Mean", "Margin_Sum", "
CurVel_Mean", "HumImp_Mean",
                      "Nitrate_Mean", "PrimProd_Mean", "ThemM_mean", "ThemR_
mean", "NumRec_mes", "NumSpe_mes", "ES50_mes")
Ecological_Data_Global_Hex_sp <- Ecological_Data_Global_Hex_sp [,analysis
.cols]
Ecological_Data_Global_Hex_sp  <- Ecological_Data_Global_Hex_sp [complete
.cases(Ecological_Data_Global_Hex_sp),]

# Calculate the correlation matrix
corr_matrix <- cor(Ecological_Data_Global_Hex_sp)

# Create the correlation plot with black font for text
corrplot(corr_matrix, tl.col = "black")
```

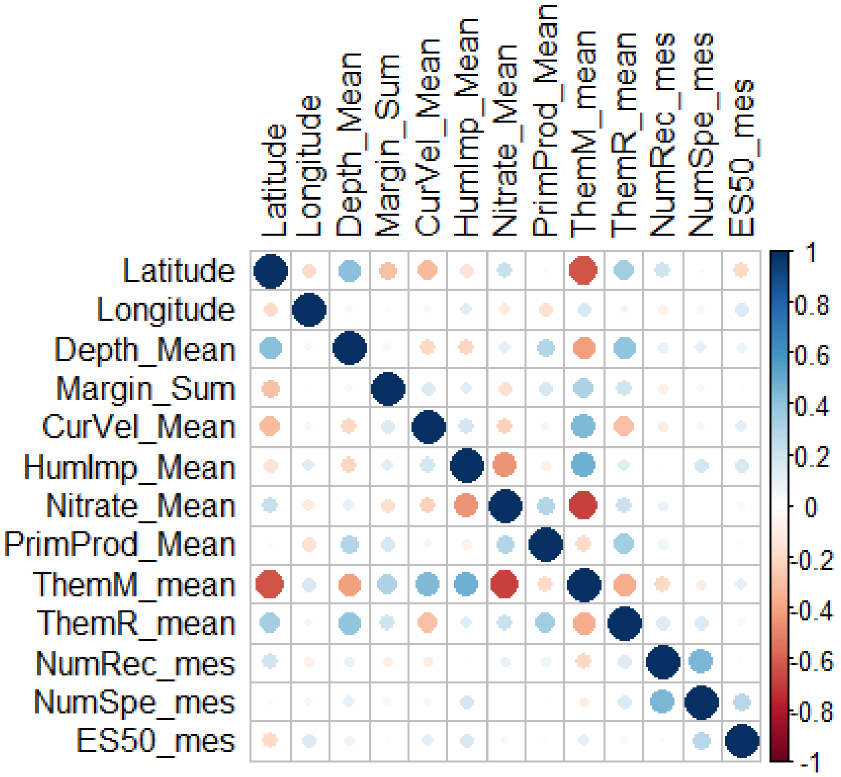


```r
#GAMs for number of species, shallow water
meso.numsp.intercept <- gam(NumSpe_mes ~ 1, data = Ecological_Data_Global
_Hex_sp, family = "nb", method = "REML", select = TRUE)
gam.check(meso.numsp.intercept)
```

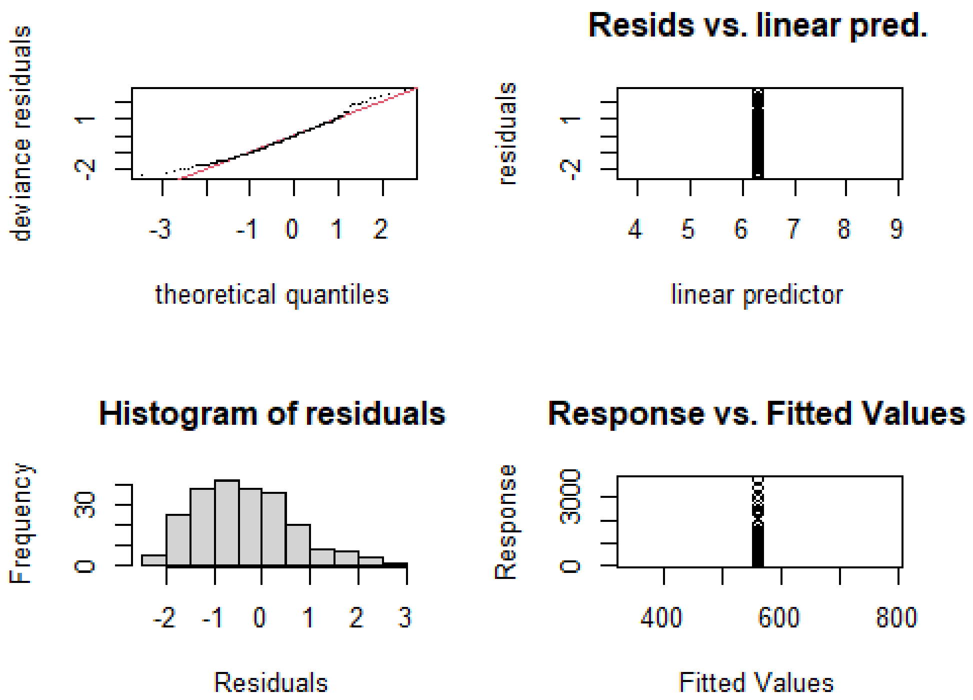


```
##
## Method: REML   Optimizer: outer newton
## full convergence after 2 iterations.
## Gradient range [0.0002938115,0.0002938115]
## (score 1651.498 & scale 1).
## Hessian positive definite, eigenvalue range [145.5484,145.5484].
## Model rank =  1 / 1
```

```
summary(meso.numsp.intercept)
```

```
##
## Family: Negative Binomial(0.928)
## Link function: log
##
## Formula:
## NumSpe_mes ~ 1
##
## Parametric coefficients:
##             Estimate Std. Error z value Pr(>|z|)
## (Intercept)  6.33301    0.06927   91.42   <2e-16 ***
## ---
## Signif. codes:  0 '***' 0.001 '**' 0.01 '*' 0.05 '.' 0.1 ' ' 1
##
##
## R-sq.(adj) =      0   Deviance explained = 2.19e-08%
## -REML = 1651.5  Scale est. = 1         n = 225
```

```
meso.numsp.latlon <- gam(NumSpe_mes ~ s(Latitude, Longitude, bs = "sos")
+ s(NumRec_mes), data = Ecological_Data_Global_Hex_sp, family = "nb", met
```

```r
hod = "REML", select = TRUE)
gam.check(meso.numsp.latlon)
```

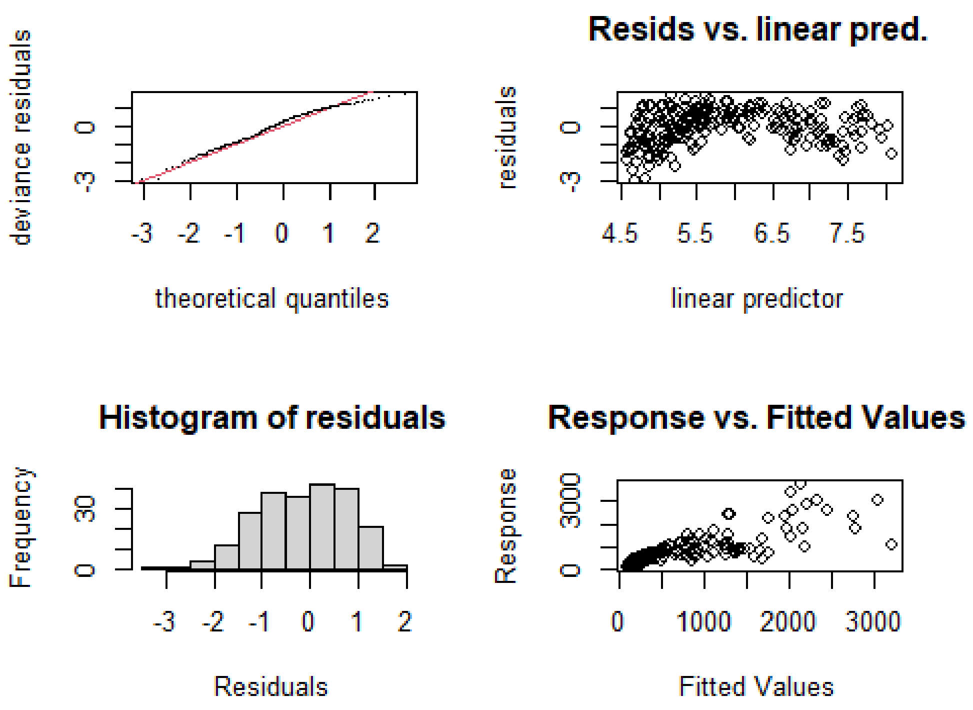


```
##
## Method: REML   Optimizer: outer newton
## full convergence after 9 iterations.
## Gradient range [-8.526406e-07,6.163126e-06]
## (score 1545.974 & scale 1).
## Hessian positive definite, eigenvalue range [0.4846765,94.5854].
## Model rank =  59 / 59
##
## Basis dimension (k) checking results. Low p-value (k-index<1) may
## indicate that k is too low, especially if edf is close to k'.
##
##                            k'   edf k-index p-value
## s(Latitude,Longitude) 49.00 24.79    0.88   0.045 *
## s(NumRec_mes)          9.00  8.54    0.51  <2e-16 ***
## ---
## Signif. codes:  0 '***' 0.001 '**' 0.01 '*' 0.05 '.' 0.1 ' ' 1
```

```r
summary(meso.numsp.latlon)
```

```
##
## Family: Negative Binomial(3.083)
## Link function: log
##
## Formula:
## NumSpe_mes ~ s(Latitude, Longitude, bs = "sos") + s(NumRec_mes)
##
## Parametric coefficients:
```

```
##              Estimate Std. Error z value Pr(>|z|)
## (Intercept)   5.8582     0.0382   153.3   <2e-16 ***
## ---
## Signif. codes:  0 '***' 0.001 '**' 0.01 '*' 0.05 '.' 0.1 ' ' 1
##
## Approximate significance of smooth terms:
##                             edf Ref.df Chi.sq p-value
## s(Latitude,Longitude) 24.791     49  97.36  <2e-16 ***
## s(NumRec_mes)          8.541      9 299.24  <2e-16 ***
## ---
## Signif. codes:  0 '***' 0.001 '**' 0.01 '*' 0.05 '.' 0.1 ' ' 1
##
## R-sq.(adj) =  0.623   Deviance explained = 76.3%
## -REML =   1546  Scale est. = 1         n = 225

meso.numsp.depth <- gam(NumSpe_mes ~ s(Latitude, Longitude, bs = "sos") +
s(NumRec_mes) + s(Depth_Mean), data = Ecological_Data_Global_Hex_sp, fami
ly = "nb", method = "REML", select = TRUE)
gam.check(meso.numsp.depth)
```

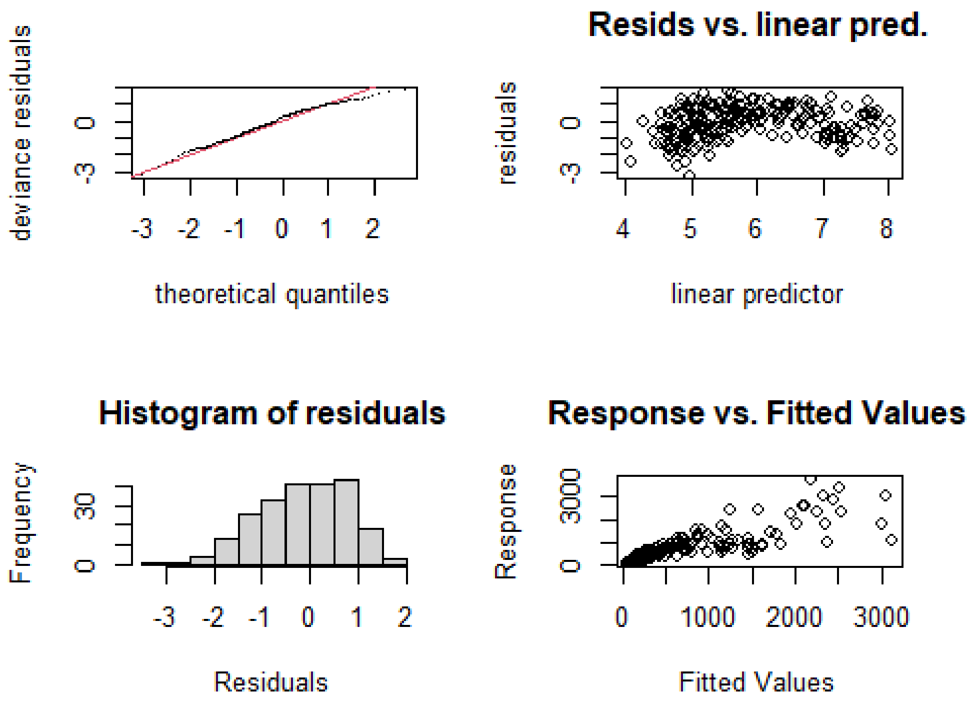


```
##
## Method: REML   Optimizer: outer newton
## full convergence after 9 iterations.
## Gradient range [-4.348986e-05,2.203905e-05]
## (score 1539.682 & scale 1).
## Hessian positive definite, eigenvalue range [4.348724e-05,92.47519].
## Model rank =  68 / 68
##
## Basis dimension (k) checking results. Low p-value (k-index<1) may
## indicate that k is too low, especially if edf is close to k'.
```

```
##
##                              k'   edf k-index p-value
## s(Latitude,Longitude) 49.00 24.44    0.89   0.075 .
## s(NumRec_mes)          9.00  8.51    0.51  <2e-16 ***
## s(Depth_Mean)          9.00  2.66    1.05   0.910
## ---
## Signif. codes:  0 '***' 0.001 '**' 0.01 '*' 0.05 '.' 0.1 ' ' 1
```

```
summary(meso.numsp.depth)
```

```
##
## Family: Negative Binomial(3.332)
## Link function: log
##
## Formula:
## NumSpe_mes ~ s(Latitude, Longitude, bs = "sos") + s(NumRec_mes) +
##     s(Depth_Mean)
##
## Parametric coefficients:
##             Estimate Std. Error z value Pr(>|z|)
## (Intercept)  5.84523    0.03678   158.9   <2e-16 ***
## ---
## Signif. codes:  0 '***' 0.001 '**' 0.01 '*' 0.05 '.' 0.1 ' ' 1
##
## Approximate significance of smooth terms:
##                          edf Ref.df Chi.sq  p-value
## s(Latitude,Longitude) 24.438     49  88.38  < 2e-16 ***
## s(NumRec_mes)          8.514      9 271.62  < 2e-16 ***
## s(Depth_Mean)          2.663      9  22.10 2.16e-06 ***
## ---
## Signif. codes:  0 '***' 0.001 '**' 0.01 '*' 0.05 '.' 0.1 ' ' 1
##
## R-sq.(adj) =  0.648   Deviance explained = 78.3%
## -REML = 1539.7  Scale est. = 1         n = 225
```

```
meso.numsp.margin <- gam(NumSpe_mes ~ s(Latitude, Longitude, bs = "sos")
+ s(NumRec_mes) + s(Margin_Sum), data = Ecological_Data_Global_Hex_sp, fa
mily = "nb", method = "REML", select = TRUE)
gam.check(meso.numsp.margin)
```

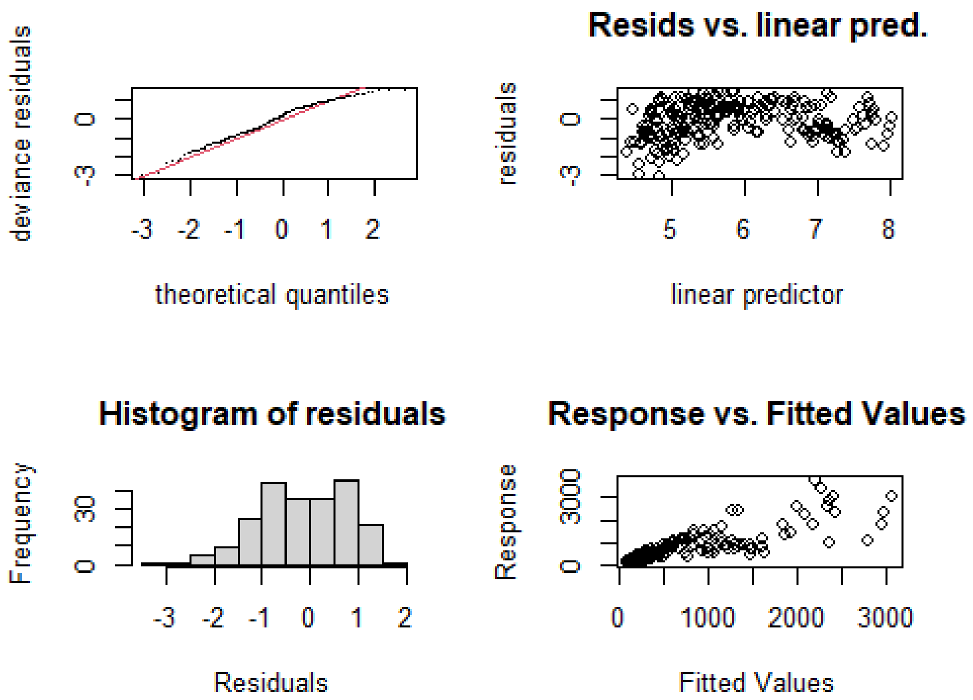


```
##
## Method: REML   Optimizer: outer newton
## full convergence after 10 iterations.
## Gradient range [-0.0001794098,0.001228159]
## (score 1543.935 & scale 1).
## Hessian positive definite, eigenvalue range [0.04227844,91.14811].
## Model rank =  68 / 68
##
## Basis dimension (k) checking results. Low p-value (k-index<1) may
## indicate that k is too low, especially if edf is close to k'.
##
##                            k'   edf k-index p-value
## s(Latitude,Longitude) 49.00 26.54    0.91    0.07 .
## s(NumRec_mes)          9.00  8.49    0.52  <2e-16 ***
## s(Margin_Sum)          9.00  2.25    1.06    0.92
## ---
## Signif. codes:  0 '***' 0.001 '**' 0.01 '*' 0.05 '.' 0.1 ' ' 1
```

```
summary(meso.numsp.margin)
```

```
##
## Family: Negative Binomial(3.273)
## Link function: log
##
## Formula:
## NumSpe_mes ~ s(Latitude, Longitude, bs = "sos") + s(NumRec_mes) +
##     s(Margin_Sum)
##
## Parametric coefficients:
##             Estimate Std. Error z value Pr(>|z|)
```

```
## (Intercept)   5.8467     0.0371   157.6   <2e-16 ***
## ---
## Signif. codes:  0 '***' 0.001 '**' 0.01 '*' 0.05 '.' 0.1 ' ' 1
##
## Approximate significance of smooth terms:
##                             edf Ref.df  Chi.sq p-value
## s(Latitude,Longitude) 26.543     49 109.375 < 2e-16 ***
## s(NumRec_mes)          8.491      9 273.182 < 2e-16 ***
## s(Margin_Sum)          2.253      9   9.792 0.00363 **
## ---
## Signif. codes:  0 '***' 0.001 '**' 0.01 '*' 0.05 '.' 0.1 ' ' 1
##
## R-sq.(adj) =  0.657   Deviance explained = 78.1%
## -REML = 1543.9  Scale est. = 1         n = 225
```

```
meso.numsp.current <- gam(NumSpe_mes ~ s(Latitude, Longitude, bs = "sos")
+ s(NumRec_mes) + s(CurVel_Mean), data = Ecological_Data_Global_Hex_sp, f
amily = "nb", method = "REML", select = TRUE)
gam.check(meso.numsp.current)
```

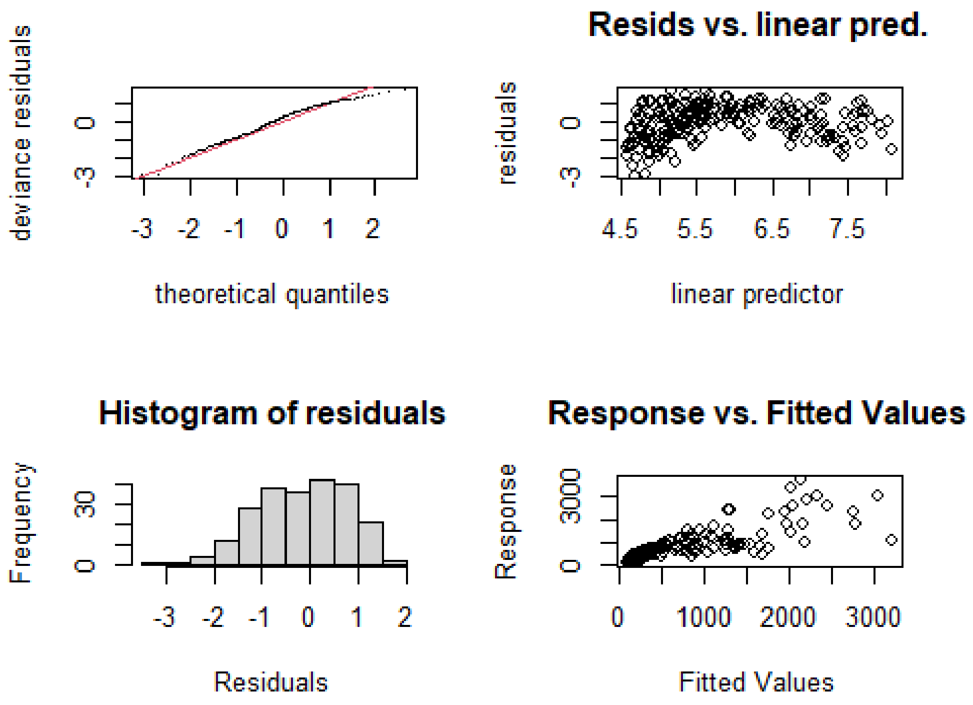


```
##
## Method: REML   Optimizer: outer newton
## full convergence after 9 iterations.
## Gradient range [-0.0009801619,-5.018585e-06]
## (score 1545.975 & scale 1).
## Hessian positive definite, eigenvalue range [4.590229e-05,94.58249].
## Model rank =  68 / 68
##
## Basis dimension (k) checking results. Low p-value (k-index<1) may
## indicate that k is too low, especially if edf is close to k'.
```

```
##
##                               k'      edf k-index p-value
## s(Latitude,Longitude) 49.00000 24.79026    0.88   0.045 *
## s(NumRec_mes)          9.00000  8.54085    0.51  <2e-16 ***
## s(CurVel_Mean)         9.00000  0.00453    0.93   0.245
## ---
## Signif. codes:  0 '***' 0.001 '**' 0.01 '*' 0.05 '.' 0.1 ' ' 1
```

```
summary(meso.numsp.current)
```

```
##
## Family: Negative Binomial(3.083)
## Link function: log
##
## Formula:
## NumSpe_mes ~ s(Latitude, Longitude, bs = "sos") + s(NumRec_mes) +
##     s(CurVel_Mean)
##
## Parametric coefficients:
##             Estimate Std. Error z value Pr(>|z|)
## (Intercept)   5.8582     0.0382   153.3   <2e-16 ***
## ---
## Signif. codes:  0 '***' 0.001 '**' 0.01 '*' 0.05 '.' 0.1 ' ' 1
##
## Approximate significance of smooth terms:
##                              edf Ref.df  Chi.sq p-value
## s(Latitude,Longitude) 24.790257     49  97.333  <2e-16 ***
## s(NumRec_mes)          8.540849      9 299.228  <2e-16 ***
## s(CurVel_Mean)         0.004532      9   0.003   0.385
## ---
## Signif. codes:  0 '***' 0.001 '**' 0.01 '*' 0.05 '.' 0.1 ' ' 1
##
## R-sq.(adj) =  0.623   Deviance explained = 76.3%
## -REML =   1546  Scale est. = 1         n = 225
```

```
meso.numsp.humimp <- gam(NumSpe_mes ~ s(Latitude, Longitude, bs = "sos")
+ s(NumRec_mes) + s(HumImp_Mean), data = Ecological_Data_Global_Hex_sp, f
amily = "nb", method = "REML", select = TRUE)
gam.check(meso.numsp.humimp)
```

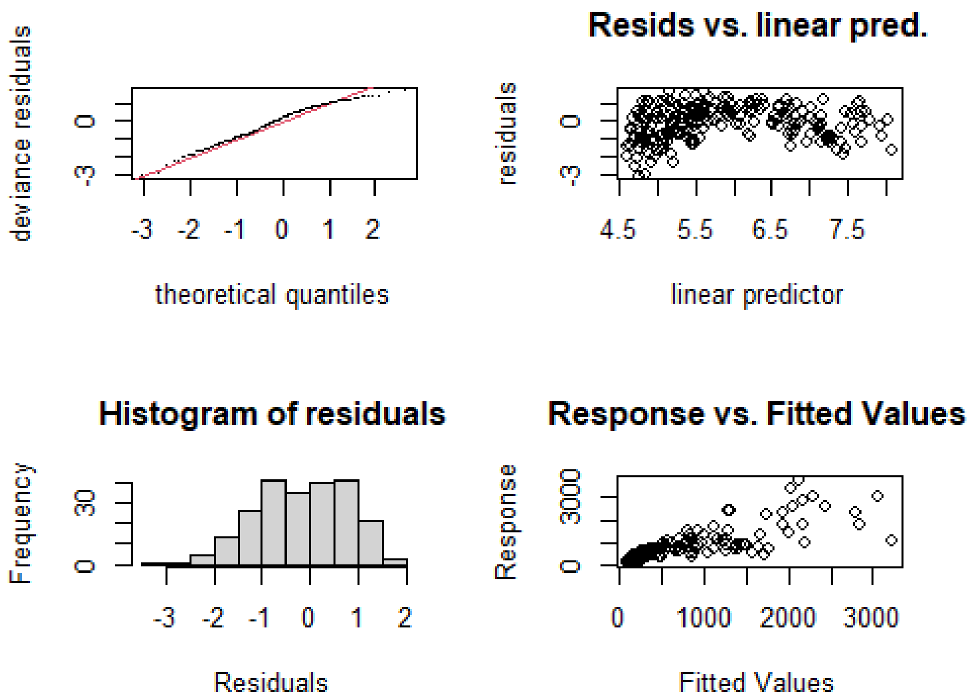


```
##
## Method: REML   Optimizer: outer newton
## full convergence after 9 iterations.
## Gradient range [-0.0002472833,0.0002701665]
## (score 1545.941 & scale 1).
## Hessian positive definite, eigenvalue range [0.000199429,95.08445].
## Model rank =  68 / 68
##
## Basis dimension (k) checking results. Low p-value (k-index<1) may
## indicate that k is too low, especially if edf is close to k'.
##
##                              k'    edf k-index p-value
## s(Latitude,Longitude) 49.000 23.920    0.87   0.025 *
## s(NumRec_mes)          9.000  8.537    0.51  <2e-16 ***
## s(HumImp_Mean)         9.000  0.336    1.02   0.780
## ---
## Signif. codes:  0 '***' 0.001 '**' 0.01 '*' 0.05 '.' 0.1 ' ' 1
```

```
summary(meso.numsp.humimp)
```

```
##
## Family: Negative Binomial(3.059)
## Link function: log
##
## Formula:
## NumSpe_mes ~ s(Latitude, Longitude, bs = "sos") + s(NumRec_mes) +
##     s(HumImp_Mean)
##
## Parametric coefficients:
##             Estimate Std. Error z value Pr(>|z|)
```

```
## (Intercept)  5.85980    0.03836   152.8   <2e-16 ***
## ---
## Signif. codes:  0 '***' 0.001 '**' 0.01 '*' 0.05 '.' 0.1 ' ' 1
##
## Approximate significance of smooth terms:
##                              edf Ref.df  Chi.sq p-value
## s(Latitude,Longitude) 23.9202     49  87.601  <2e-16 ***
## s(NumRec_mes)          8.5366      9 294.902  <2e-16 ***
## s(HumImp_Mean)         0.3361      9   0.493   0.168
## ---
## Signif. codes:  0 '***' 0.001 '**' 0.01 '*' 0.05 '.' 0.1 ' ' 1
##
## R-sq.(adj) =  0.622   Deviance explained =   76%
## -REML = 1545.9  Scale est. = 1         n = 225
```

```
meso.numsp.nitrate <- gam(NumSpe_mes ~ s(Latitude, Longitude, bs = "sos")
+ s(NumRec_mes) + s(Nitrate_Mean), data = Ecological_Data_Global_Hex_sp,
family = "nb", method = "REML", select = TRUE)
gam.check(meso.numsp.nitrate)
```

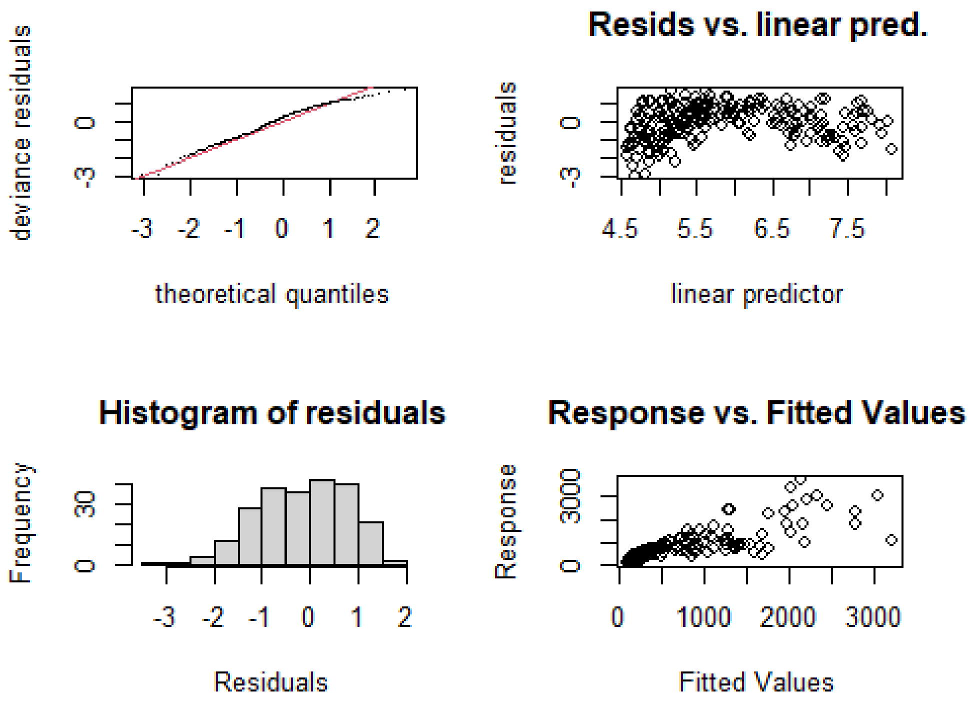


```
##
## Method: REML   Optimizer: outer newton
## full convergence after 10 iterations.
## Gradient range [-0.0004225906,0.0002361159]
## (score 1545.975 & scale 1).
## Hessian positive definite, eigenvalue range [2.401827e-05,94.58484].
## Model rank =  68 / 68
##
## Basis dimension (k) checking results. Low p-value (k-index<1) may
## indicate that k is too low, especially if edf is close to k'.
```

```
##
##                              k'      edf k-index p-value
## s(Latitude,Longitude) 49.00000 24.79064    0.88   0.035 *
## s(NumRec_mes)          9.00000  8.54085    0.51  <2e-16 ***
## s(Nitrate_Mean)        9.00000  0.00128    0.99   0.600
## ---
## Signif. codes:  0 '***' 0.001 '**' 0.01 '*' 0.05 '.' 0.1 ' ' 1
```

```
summary(meso.numsp.nitrate)
```

```
##
## Family: Negative Binomial(3.083)
## Link function: log
##
## Formula:
## NumSpe_mes ~ s(Latitude, Longitude, bs = "sos") + s(NumRec_mes) +
##     s(Nitrate_Mean)
##
## Parametric coefficients:
##             Estimate Std. Error z value Pr(>|z|)
## (Intercept)   5.8582     0.0382   153.3   <2e-16 ***
## ---
## Signif. codes:  0 '***' 0.001 '**' 0.01 '*' 0.05 '.' 0.1 ' ' 1
##
## Approximate significance of smooth terms:
##                             edf Ref.df Chi.sq p-value
## s(Latitude,Longitude) 24.790635     49  97.36  <2e-16 ***
## s(NumRec_mes)          8.540851      9 299.24  <2e-16 ***
## s(Nitrate_Mean)        0.001285      9   0.00   0.745
## ---
## Signif. codes:  0 '***' 0.001 '**' 0.01 '*' 0.05 '.' 0.1 ' ' 1
##
## R-sq.(adj) =  0.623   Deviance explained = 76.3%
## -REML =   1546  Scale est. = 1         n = 225
```

```
meso.numsp.PrimProd <- gam(NumSpe_mes ~ s(Latitude, Longitude, bs = "sos"
) + s(NumRec_mes) + s(PrimProd_Mean), data = Ecological_Data_Global_Hex_s
p, family = "nb", method = "REML", select = TRUE)
gam.check(meso.numsp.PrimProd)
```

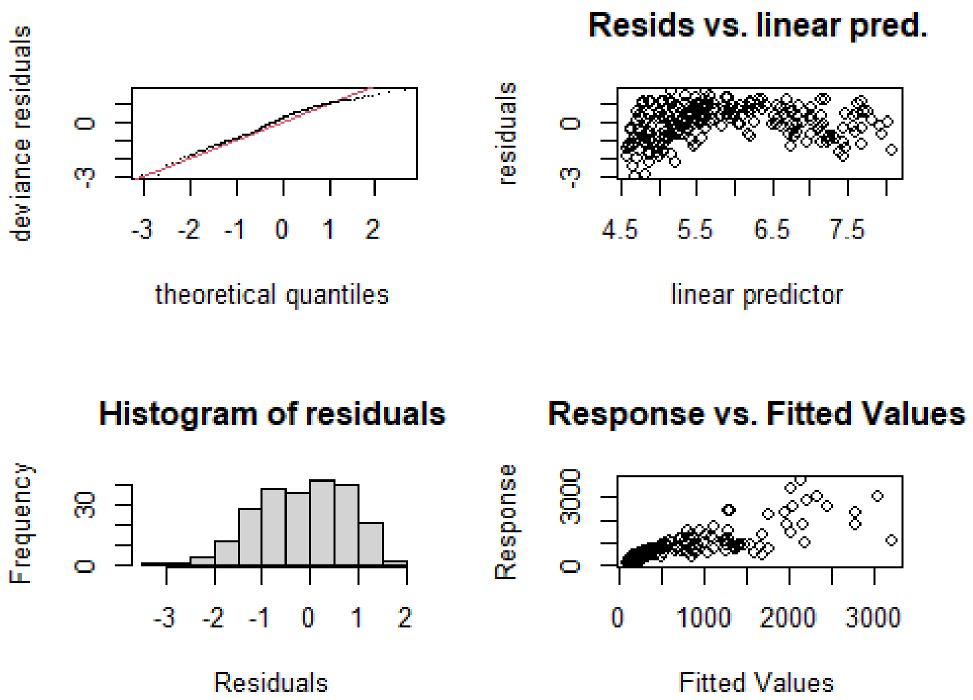


```
##
## Method: REML   Optimizer: outer newton
## full convergence after 10 iterations.
## Gradient range [-0.0003359434,0.0001563553]
## (score 1545.975 & scale 1).
## Hessian positive definite, eigenvalue range [0.000157952,94.58501].
## Model rank =  68 / 68
##
## Basis dimension (k) checking results. Low p-value (k-index<1) may
## indicate that k is too low, especially if edf is close to k'.
##
##                              k'      edf k-index p-value
## s(Latitude,Longitude) 49.00000 24.79065    0.88   0.020 *
## s(NumRec_mes)          9.00000  8.54085    0.51  <2e-16 ***
## s(PrimProd_Mean)       9.00000  0.00187    0.86   0.045 *
## ---
## Signif. codes:  0 '***' 0.001 '**' 0.01 '*' 0.05 '.' 0.1 ' ' 1
```

```
summary(meso.numsp.PrimProd)
```

```
##
## Family: Negative Binomial(3.083)
## Link function: log
##
## Formula:
## NumSpe_mes ~ s(Latitude, Longitude, bs = "sos") + s(NumRec_mes) +
##     s(PrimProd_Mean)
##
## Parametric coefficients:
##             Estimate Std. Error z value Pr(>|z|)
```

```
## (Intercept)   5.8582     0.0382   153.3   <2e-16 ***
## ---
## Signif. codes:  0 '***' 0.001 '**' 0.01 '*' 0.05 '.' 0.1 ' ' 1
##
## Approximate significance of smooth terms:
##                              edf Ref.df  Chi.sq p-value
## s(Latitude,Longitude) 24.790650     49  97.363  <2e-16 ***
## s(NumRec_mes)          8.540849      9 299.235  <2e-16 ***
## s(PrimProd_Mean)       0.001865      9   0.001   0.587
## ---
## Signif. codes:  0 '***' 0.001 '**' 0.01 '*' 0.05 '.' 0.1 ' ' 1
##
## R-sq.(adj) =  0.624   Deviance explained = 76.3%
## -REML =   1546  Scale est. = 1         n = 225
```

```
meso.numsp.ThemM <- gam(NumSpe_mes ~ s(Latitude, Longitude, bs = "sos") +
s(NumRec_mes) + s(ThemM_mean), data = Ecological_Data_Global_Hex_sp, fami
ly = "nb", method = "REML", select = TRUE)
gam.check(meso.numsp.ThemM)
```

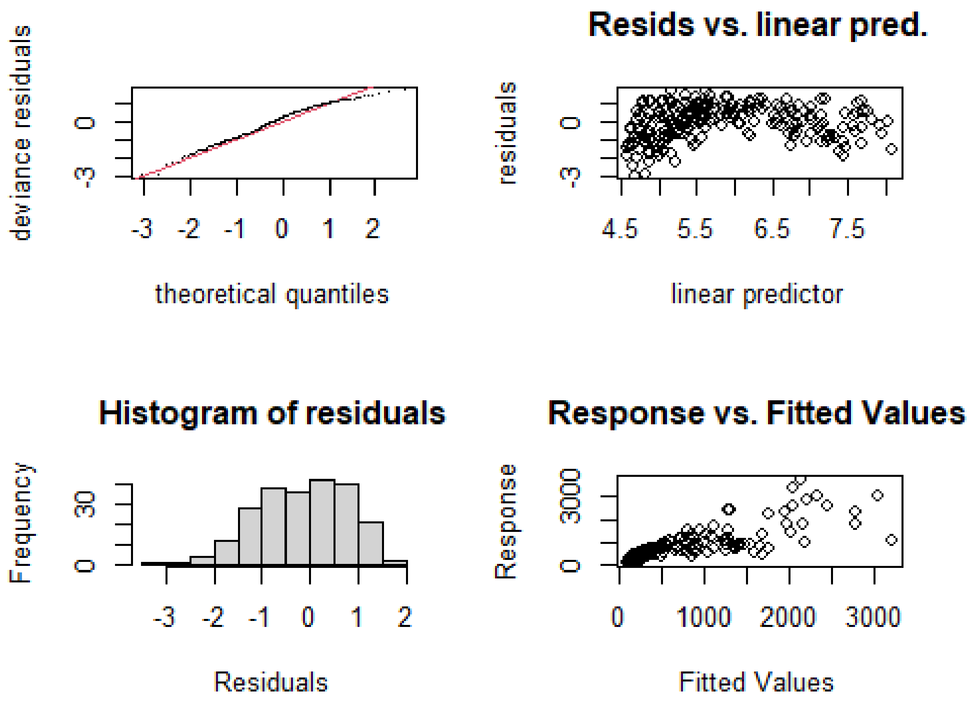


```
##
## Method: REML   Optimizer: outer newton
## full convergence after 10 iterations.
## Gradient range [-0.0008125786,0.0002713774]
## (score 1545.975 & scale 1).
## Hessian positive definite, eigenvalue range [1.218583e-05,94.58373].
## Model rank =  68 / 68
##
## Basis dimension (k) checking results. Low p-value (k-index<1) may
## indicate that k is too low, especially if edf is close to k'.
```

```
##
##                              k'      edf k-index p-value
## s(Latitude,Longitude) 49.00000 24.78872    0.88   0.025 *
## s(NumRec_mes)          9.00000  8.54085    0.51  <2e-16 ***
## s(ThemM_mean)          9.00000  0.00422    0.94   0.300
## ---
## Signif. codes:  0 '***' 0.001 '**' 0.01 '*' 0.05 '.' 0.1 ' ' 1
```

```
summary(meso.numsp.ThemM)
```

```
##
## Family: Negative Binomial(3.083)
## Link function: log
##
## Formula:
## NumSpe_mes ~ s(Latitude, Longitude, bs = "sos") + s(NumRec_mes) +
##     s(ThemM_mean)
##
## Parametric coefficients:
##             Estimate Std. Error z value Pr(>|z|)
## (Intercept)   5.8582     0.0382   153.3   <2e-16 ***
## ---
## Signif. codes:  0 '***' 0.001 '**' 0.01 '*' 0.05 '.' 0.1 ' ' 1
##
## Approximate significance of smooth terms:
##                             edf Ref.df  Chi.sq p-value
## s(Latitude,Longitude) 24.788720     49  97.241  <2e-16 ***
## s(NumRec_mes)          8.540849      9 299.190  <2e-16 ***
## s(ThemM_mean)          0.004221      9   0.003   0.281
## ---
## Signif. codes:  0 '***' 0.001 '**' 0.01 '*' 0.05 '.' 0.1 ' ' 1
##
## R-sq.(adj) =  0.624   Deviance explained = 76.3%
## -REML =   1546  Scale est. = 1         n = 225
```

```
meso.numsp.ThemR <- gam(NumSpe_mes ~ s(Latitude, Longitude, bs = "sos") +
s(NumRec_mes) + s(ThemR_mean), data = Ecological_Data_Global_Hex_sp, fami
ly = "nb", method = "REML", select = TRUE)
gam.check(meso.numsp.ThemR)
```

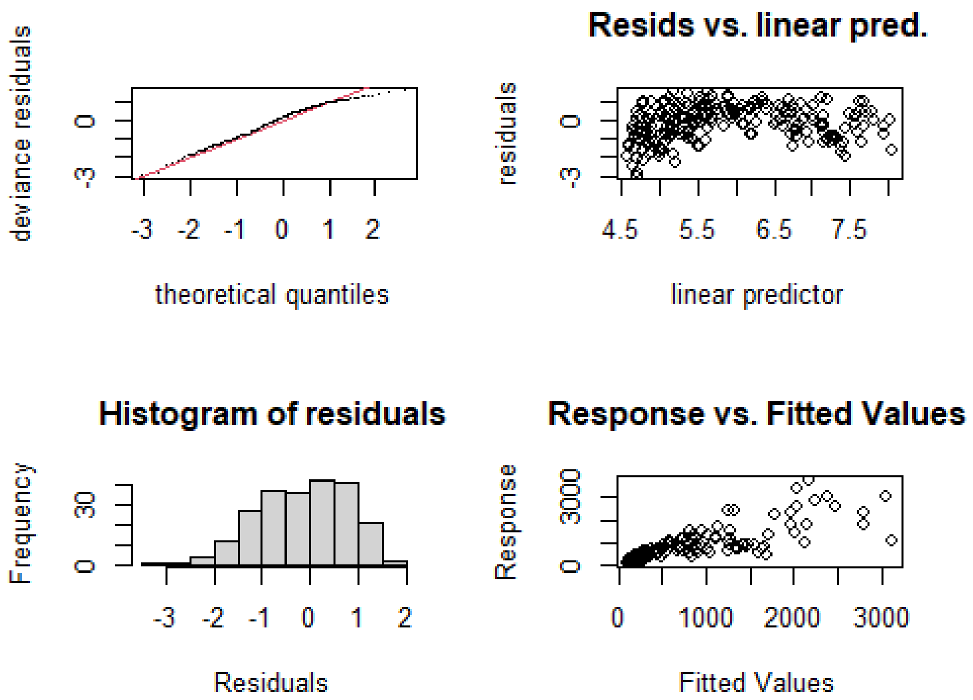


```
##
## Method: REML   Optimizer: outer newton
## full convergence after 8 iterations.
## Gradient range [-2.37141e-05,1.907653e-05]
## (score 1545.917 & scale 1).
## Hessian positive definite, eigenvalue range [2.37119e-05,93.73236].
## Model rank =  68 / 68
##
## Basis dimension (k) checking results. Low p-value (k-index<1) may
## indicate that k is too low, especially if edf is close to k'.
##
##                             k'    edf k-index p-value
## s(Latitude,Longitude) 49.000 24.763    0.88    0.03 *
## s(NumRec_mes)          9.000  8.536    0.51  <2e-16 ***
## s(ThemR_mean)          9.000  0.931    0.98    0.50
## ---
## Signif. codes:  0 '***' 0.001 '**' 0.01 '*' 0.05 '.' 0.1 ' ' 1
```

```
summary(meso.numsp.ThemR)
```

```
##
## Family: Negative Binomial(3.108)
## Link function: log
##
## Formula:
## NumSpe_mes ~ s(Latitude, Longitude, bs = "sos") + s(NumRec_mes) +
##     s(ThemR_mean)
##
## Parametric coefficients:
##             Estimate Std. Error z value Pr(>|z|)
```

```
## (Intercept)  5.85637    0.03806   153.9   <2e-16 ***
## ---
## Signif. codes:  0 '***' 0.001 '**' 0.01 '*' 0.05 '.' 0.1 ' ' 1
##
## Approximate significance of smooth terms:
##                              edf Ref.df  Chi.sq p-value
## s(Latitude,Longitude) 24.7632     49  97.028  <2e-16 ***
## s(NumRec_mes)          8.5358      9 293.423  <2e-16 ***
## s(ThemR_mean)          0.9308      9   1.321   0.127
## ---
## Signif. codes:  0 '***' 0.001 '**' 0.01 '*' 0.05 '.' 0.1 ' ' 1
##
## R-sq.(adj) =  0.634   Deviance explained = 76.5%
## -REML = 1545.9  Scale est. = 1         n = 225
```

```
meso.numsp.env <- gam(NumSpe_mes ~ s(Latitude, Longitude, bs = "sos") + s
(NumRec_mes) + s(Depth_Mean) +  s(Margin_Sum) + s(CurVel_Mean) + s(HumImp
_Mean) + s(Nitrate_Mean) + s(PrimProd_Mean) + s(ThemM_mean) + s(ThemR_mea
n), data = Ecological_Data_Global_Hex_sp, family = "nb", method = "REML",
select = TRUE)
gam.check(meso.numsp.env)
```

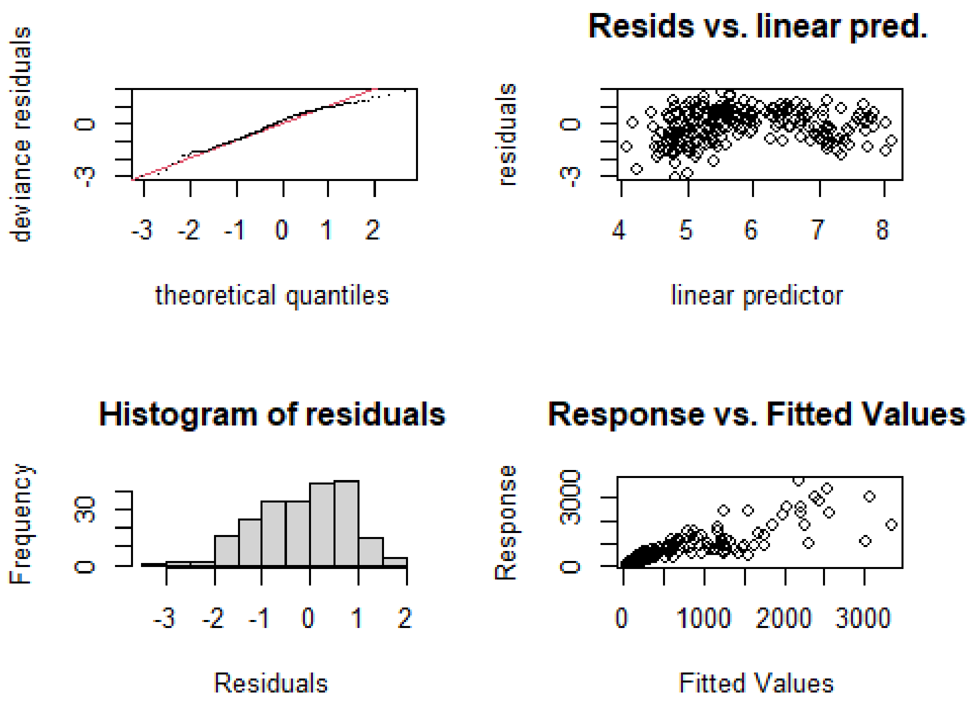


```
##
## Method: REML   Optimizer: outer newton
## full convergence after 12 iterations.
## Gradient range [-0.001286921,0.0003777977]
## (score 1538.698 & scale 1).
## Hessian positive definite, eigenvalue range [1.173781e-05,92.55533].
## Model rank =  131 / 131
##
```

```
## Basis dimension (k) checking results. Low p-value (k-index<1) may
## indicate that k is too low, especially if edf is close to k'.
##
##                                k'      edf k-index p-value
## s(Latitude,Longitude) 4.90e+01 2.01e+01    0.86   0.005 **
## s(NumRec_mes)         9.00e+00 8.50e+00    0.51  <2e-16 ***
## s(Depth_Mean)         9.00e+00 2.67e+00    1.06   0.890
## s(Margin_Sum)         9.00e+00 1.22e-02    1.05   0.850
## s(CurVel_Mean)        9.00e+00 1.55e+00    0.95   0.310
## s(HumImp_Mean)        9.00e+00 7.42e-01    1.01   0.695
## s(Nitrate_Mean)       9.00e+00 1.81e-04    0.98   0.515
## s(PrimProd_Mean)      9.00e+00 1.18e-04    0.91   0.125
## s(ThemM_mean)         9.00e+00 1.62e-04    0.98   0.565
## s(ThemR_mean)         9.00e+00 1.58e+00    1.01   0.725
## ---
## Signif. codes:  0 '***' 0.001 '**' 0.01 '*' 0.05 '.' 0.1 ' ' 1
```

```
summary(meso.numsp.env)
```

```
##
## Family: Negative Binomial(3.307)
## Link function: log
##
## Formula:
## NumSpe_mes ~ s(Latitude, Longitude, bs = "sos") + s(NumRec_mes) +
##     s(Depth_Mean) + s(Margin_Sum) + s(CurVel_Mean) + s(HumImp_Mean) +
##     s(Nitrate_Mean) + s(PrimProd_Mean) + s(ThemM_mean) + s(ThemR_mean)
##
## Parametric coefficients:
##             Estimate Std. Error z value Pr(>|z|)
## (Intercept)  5.84669    0.03691   158.4   <2e-16 ***
## ---
## Signif. codes:  0 '***' 0.001 '**' 0.01 '*' 0.05 '.' 0.1 ' ' 1
##
## Approximate significance of smooth terms:
##                             edf Ref.df  Chi.sq  p-value
## s(Latitude,Longitude) 2.007e+01     49  65.252  < 2e-16 ***
## s(NumRec_mes)         8.501e+00      9 256.747  < 2e-16 ***
## s(Depth_Mean)         2.670e+00      9  25.117 3.54e-07 ***
## s(Margin_Sum)         1.220e-02      9   0.010   0.4002
## s(CurVel_Mean)        1.552e+00      9   2.911   0.0970 .
## s(HumImp_Mean)        7.420e-01      9   2.870   0.0257 *
## s(Nitrate_Mean)       1.811e-04      9   0.000   0.9344
## s(PrimProd_Mean)      1.182e-04      9   0.000   0.6575
## s(ThemM_mean)         1.616e-04      9   0.000   0.3518
## s(ThemR_mean)         1.576e+00      9   3.716   0.0330 *
## ---
## Signif. codes:  0 '***' 0.001 '**' 0.01 '*' 0.05 '.' 0.1 ' ' 1
##
## R-sq.(adj) =  0.659   Deviance explained = 78.1%
## -REML = 1538.7  Scale est. = 1         n = 225
```

```r
meso.numsp.models <- list(Intercept = meso.numsp.intercept,
                          LatLon = meso.numsp.latlon,
                          Depth = meso.numsp.depth,
                          ConMAr = meso.numsp.margin,
                          CurVel = meso.numsp.current,
                          HumImp = meso.numsp.humimp,
                          Nitrate = meso.numsp.nitrate,
                          PriPro = meso.numsp.PrimProd,
                          TemMea = meso.numsp.ThemM,
                          TheRan = meso.numsp.ThemR,
                          Environment = meso.numsp.env)
meso.numsp.aic.df <- data.frame(Model = names(meso.numsp.models),
                                AIC = sapply(meso.numsp.models, function(
x) x$aic),
                                akaike.weights(sapply(meso.numsp.models,
function(x) x$aic)))

meso.numsp.aic.df <- meso.numsp.aic.df[order(meso.numsp.aic.df$AIC),]
meso.numsp.aic.df$Cumulative.Weight <- cumsum(meso.numsp.aic.df$weights)

kable(meso.numsp.aic.df, row.names = FALSE)
```

Model	AIC	deltaAIC	rel.LL	weights	Cumulative.Weight
Depth	3000.797	0.000000	1.0000000	0.6531844	0.6531844
Environment	3002.256	1.459019	0.4821455	0.3149299	0.9681143
ConMAr	3006.910	6.112356	0.0470672	0.0307436	0.9988579
TheRan	3016.443	15.645734	0.0004005	0.0002616	0.9991194
LatLon	3017.419	16.621996	0.0002458	0.0001606	0.9992800
TemMea	3017.420	16.622990	0.0002457	0.0001605	0.9994405
Nitrate	3017.421	16.623227	0.0002456	0.0001605	0.9996009
PriPro	3017.421	16.623968	0.0002456	0.0001604	0.9997613
CurVel	3017.422	16.624219	0.0002455	0.0001604	0.9999217
HumImp	3018.855	18.057572	0.0001199	0.0000783	1.0000000
Intercept	3301.494	300.697110	0.0000000	0.0000000	1.0000000

```r
#write.csv(meso.numsp.aic.df, file = "meso.numsp.aic.GAM.csv")

#Plots for number of species, Meso
ggplot(Ecological_Data_Global_Hex_sp, aes(x = Depth_Mean, y = predict(mes
o.numsp.depth, Ecological_Data_Global_Hex_sp))) +
  geom_smooth(method = "gam", formula = y ~ x, color = "#1a80bb", fill =
"#85bede") +  # Add a smooth dark blue line with light blue shadow
  geom_point(size = 3) +  # Add scatter plot points
  theme_bw() +  # Use the black and white theme
  labs(
    x = "Depth (m)",  # Shorten the x-axis title
    y = "Predicted Value"  # Shorten the y-axis title
  ) +
  theme(
```

```
    panel.grid.minor = element_blank(),
    panel.grid.major = element_blank(),
    axis.text.x = element_text(size =20),  # Increase x-axis text size
    axis.text.y = element_text(size = 20),  # Increase y-axis text size
    axis.title.x = element_text(size = 22), # Increase x-axis title size
    axis.title.y = element_text(size = 22)  # Increase y-axis title size
  )
```

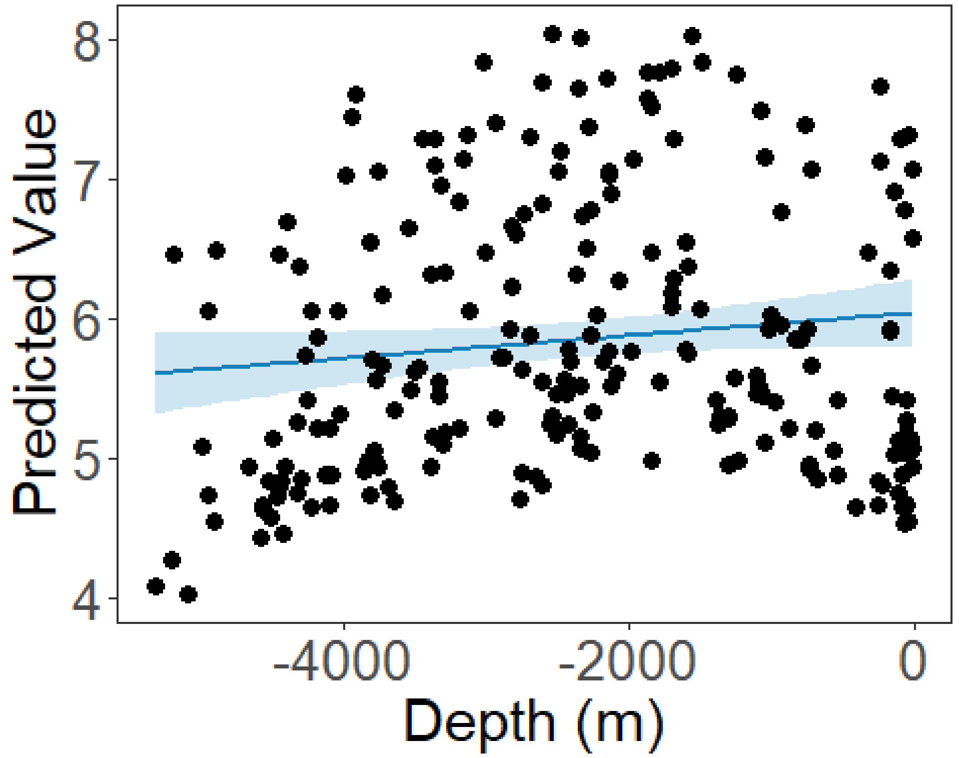


```
ggplot(Ecological_Data_Global_Hex_sp, aes(x = Margin_Sum, y = predict(mes
o.numsp.margin, Ecological_Data_Global_Hex_sp))) +
  geom_smooth(method = "gam", formula = y ~ x, color = "#1a80bb", fill =
"#85bede") +  # Add a smooth dark blue line with light blue shadow
  geom_point(size = 3) +  # Add scatter plot points
  theme_bw() +  # Use the black and white theme
  labs(
    x = "Continental Margin (km2)",  # Shorten the x-axis title
    y = "Predicted Value"  # Shorten the y-axis title
  ) +
  theme(
    panel.grid.minor = element_blank(),
    panel.grid.major = element_blank(),
    axis.text.x = element_text(size =20),  # Increase x-axis text size
    axis.text.y = element_text(size = 20),  # Increase y-axis text size
    axis.title.x = element_text(size = 22), # Increase x-axis title size
    axis.title.y = element_text(size = 22)  # Increase y-axis title size
  )
```

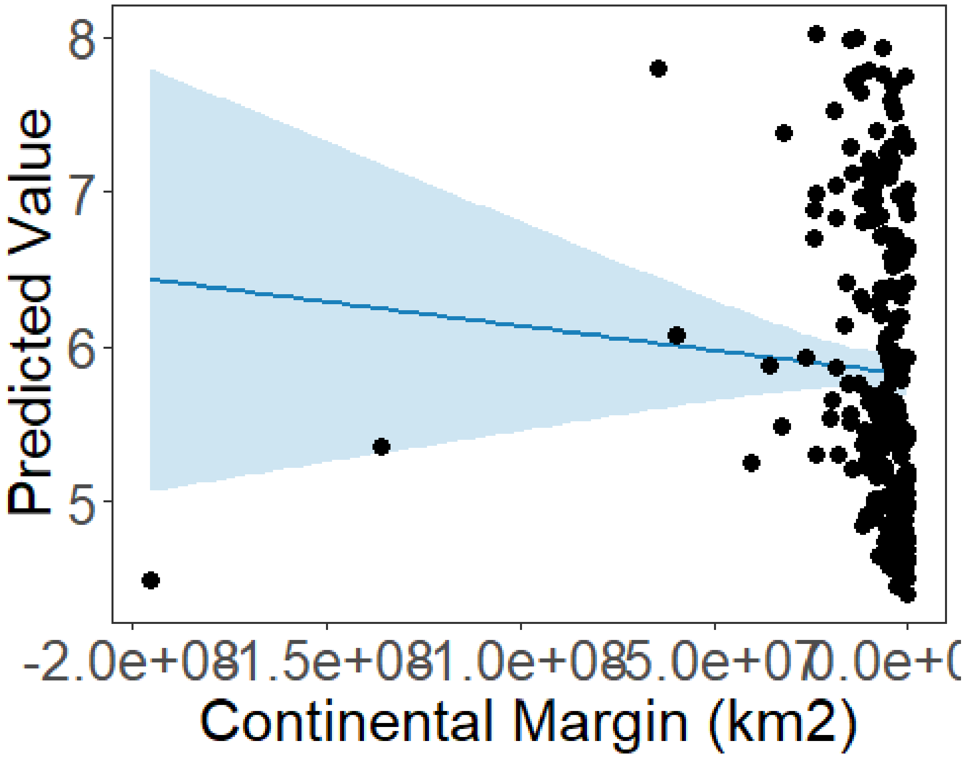


```
ggplot(Ecological_Data_Global_Hex_sp, aes(x = CurVel_Mean, y = predict(me
so.numsp.current, Ecological_Data_Global_Hex_sp))) +
  geom_smooth(method = "gam", formula = y ~ x, color = "#1a80bb", fill =
"#85bede") +  # Add a smooth dark blue line with light blue shadow
  geom_point(size = 3) +  # Add scatter plot points
  theme_bw() +  # Use the black and white theme
  labs(
    x = "Current Velocity (m.s-1)",  # Shorten the x-axis title
    y = "Predicted Value"  # Shorten the y-axis title
  ) +
  theme(
    panel.grid.minor = element_blank(),
    panel.grid.major = element_blank(),
    axis.text.x = element_text(size =20),  # Increase x-axis text size
    axis.text.y = element_text(size = 20),  # Increase y-axis text size
    axis.title.x = element_text(size = 22), # Increase x-axis title size
    axis.title.y = element_text(size = 22)  # Increase y-axis title size
  )
```

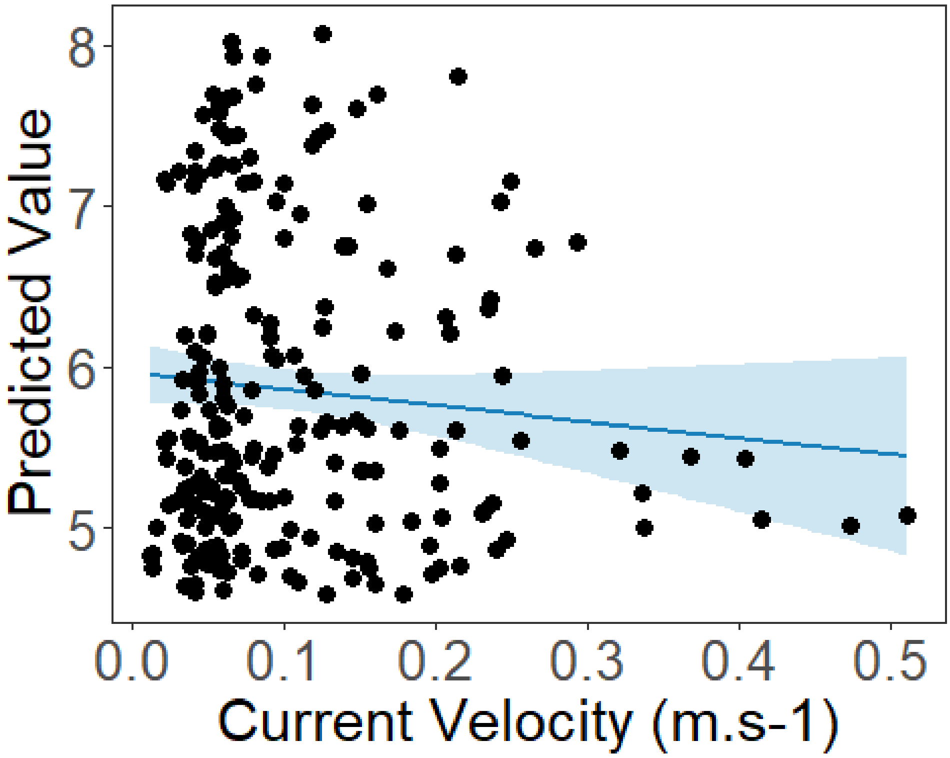


```
ggplot(Ecological_Data_Global_Hex_sp, aes(x = HumImp_Mean, y = predict(me
so.numsp.humimp, Ecological_Data_Global_Hex_sp))) +
  geom_smooth(method = "gam", formula = y ~ x, color = "#1a80bb", fill =
"#85bede") +  # Add a smooth dark blue line with light blue shadow
  geom_point(size = 3) +  # Add scatter plot points
  theme_bw() +  # Use the black and white theme
  labs(
    x = "Human Impact",  # Shorten the x-axis title
    y = "Predicted Value"  # Shorten the y-axis title
  ) +
  theme(
    panel.grid.minor = element_blank(),
    panel.grid.major = element_blank(),
    axis.text.x = element_text(size =20),  # Increase x-axis text size
    axis.text.y = element_text(size = 20),  # Increase y-axis text size
    axis.title.x = element_text(size = 22), # Increase x-axis title size
    axis.title.y = element_text(size = 22)  # Increase y-axis title size
  )
```

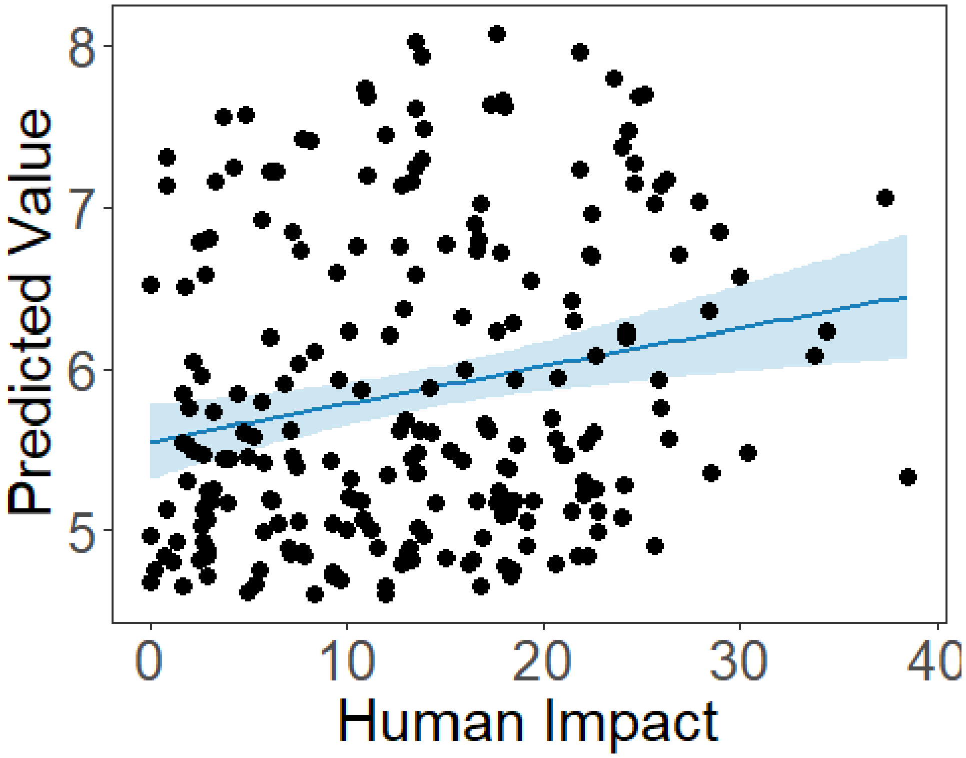


```
ggplot(Ecological_Data_Global_Hex_sp, aes(x = Nitrate_Mean, y = predict(m
eso.numsp.nitrate, Ecological_Data_Global_Hex_sp))) +
  geom_smooth(method = "gam", formula = y ~ x, color = "#1a80bb", fill =
"#85bede") +  # Add a smooth dark blue line with light blue shadow
  geom_point(size = 3) +  # Add scatter plot points
  theme_bw() +  # Use the black and white theme
  labs(
    x = "Nitrate (mmol . m-3)",  # Shorten the x-axis title
    y = "Predicted Value"  # Shorten the y-axis title
  ) +
  theme(
    panel.grid.minor = element_blank(),
    panel.grid.major = element_blank(),
    axis.text.x = element_text(size =20),  # Increase x-axis text size
    axis.text.y = element_text(size = 20),  # Increase y-axis text size
    axis.title.x = element_text(size = 22), # Increase x-axis title size
    axis.title.y = element_text(size = 22)  # Increase y-axis title size
  )
```

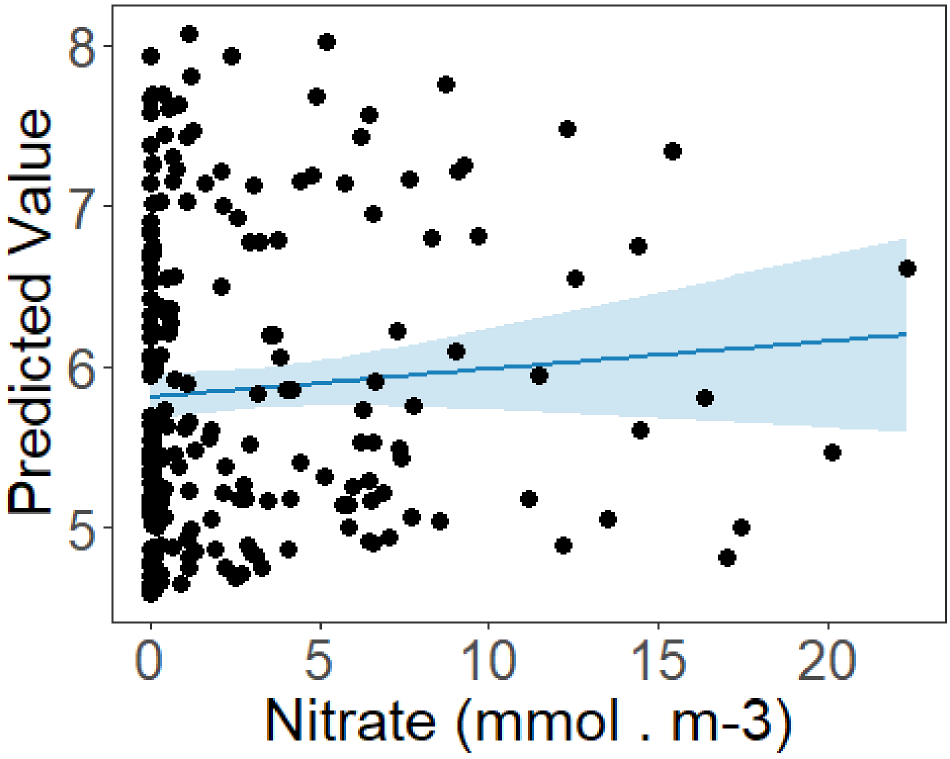


```
ggplot(Ecological_Data_Global_Hex_sp, aes(x = PrimProd_Mean, y = predict(
meso.numsp.PrimProd, Ecological_Data_Global_Hex_sp))) +
  geom_smooth(method = "gam", formula = y ~ x, color = "#1a80bb", fill =
"#85bede") +  # Add a smooth dark blue line with light blue shadow
  geom_point(size = 3) +  # Add scatter plot points
  theme_bw() +  # Use the black and white theme
  labs(
    x = "Primary Productivity (mmol . m-3)",  # Shorten the x-axis title
    y = "Predicted Value"  # Shorten the y-axis title
  ) +
  theme(
    panel.grid.minor = element_blank(),
    panel.grid.major = element_blank(),
    axis.text.x = element_text(size =20),  # Increase x-axis text size
    axis.text.y = element_text(size = 20),  # Increase y-axis text size
    axis.title.x = element_text(size = 22), # Increase x-axis title size
    axis.title.y = element_text(size = 22)  # Increase y-axis title size
  )
```

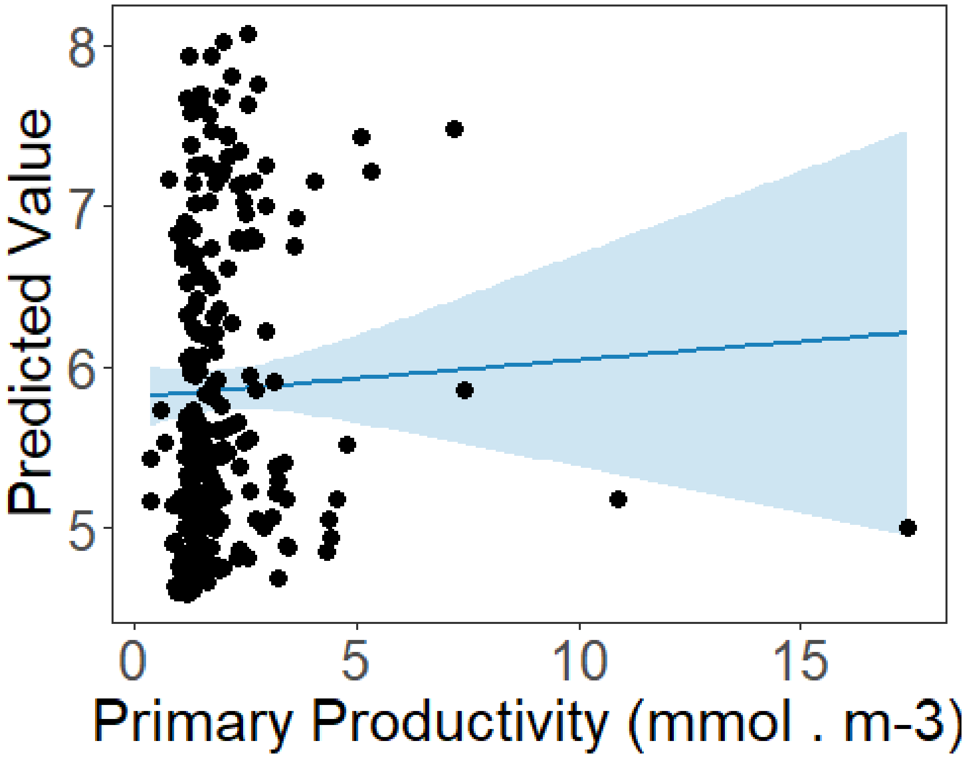


```
ggplot(Ecological_Data_Global_Hex_sp, aes(x = ThemM_mean, y = predict(meso.numsp.ThemM, Ecological_Data_Global_Hex_sp))) +
  geom_smooth(method = "gam", formula = y ~ x, color = "#1a80bb", fill = "#85bede") +  # Add a smooth dark blue line with light blue shadow
  geom_point(size = 3) +  # Add scatter plot points
  theme_bw() +  # Use the black and white theme
  labs(
    x = "Temperature Mean (ºC)",  # Shorten the x-axis title
    y = "Predicted Value"  # Shorten the y-axis title
  ) +
  theme(
    panel.grid.minor = element_blank(),
    panel.grid.major = element_blank(),
    axis.text.x = element_text(size =20),  # Increase x-axis text size
    axis.text.y = element_text(size = 20),  # Increase y-axis text size
    axis.title.x = element_text(size = 22), # Increase x-axis title size
    axis.title.y = element_text(size = 22)  # Increase y-axis title size
  )
```

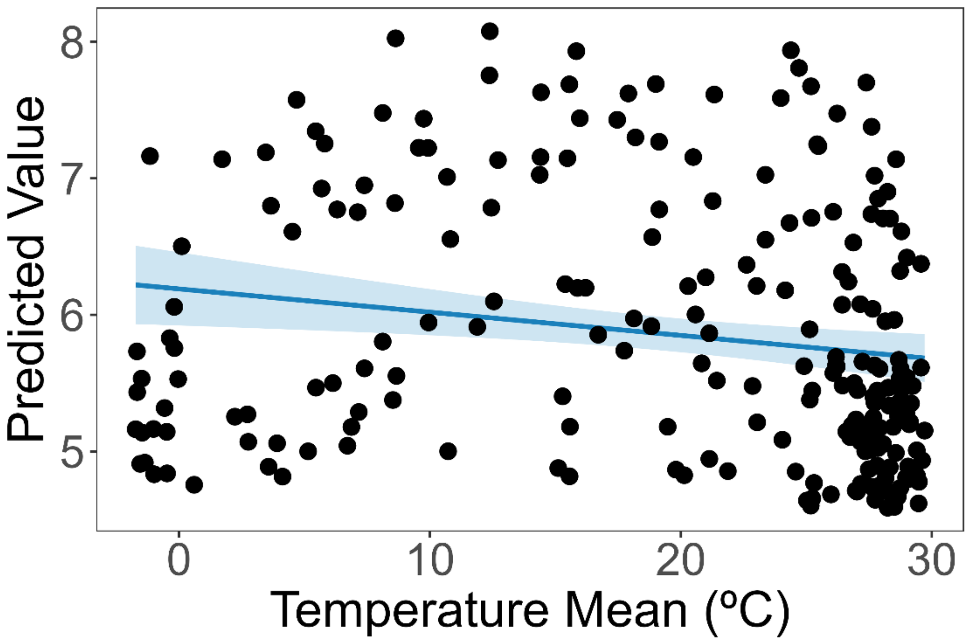


```
ggplot(Ecological_Data_Global_Hex_sp, aes(x = ThemR_mean, y = predict(meso.numsp.ThemR, Ecological_Data_Global_Hex_sp))) +
  geom_smooth(method = "gam", formula = y ~ x, color = "#1a80bb", fill = "#85bede") +  # Add a smooth dark blue line with light blue shadow
  geom_point(size = 3) +  # Add scatter plot points
  theme_bw() +  # Use the black and white theme
  labs(
    x = "Temperature Range (ºC)",  # Shorten the x-axis title
    y = "Predicted Value"  # Shorten the y-axis title
  ) +
  theme(
    panel.grid.minor = element_blank(),
    panel.grid.major = element_blank(),
    axis.text.x = element_text(size =20),  # Increase x-axis text size
    axis.text.y = element_text(size = 20),  # Increase y-axis text size
    axis.title.x = element_text(size = 22), # Increase x-axis title size
    axis.title.y = element_text(size = 22)  # Increase y-axis title size
  )
```

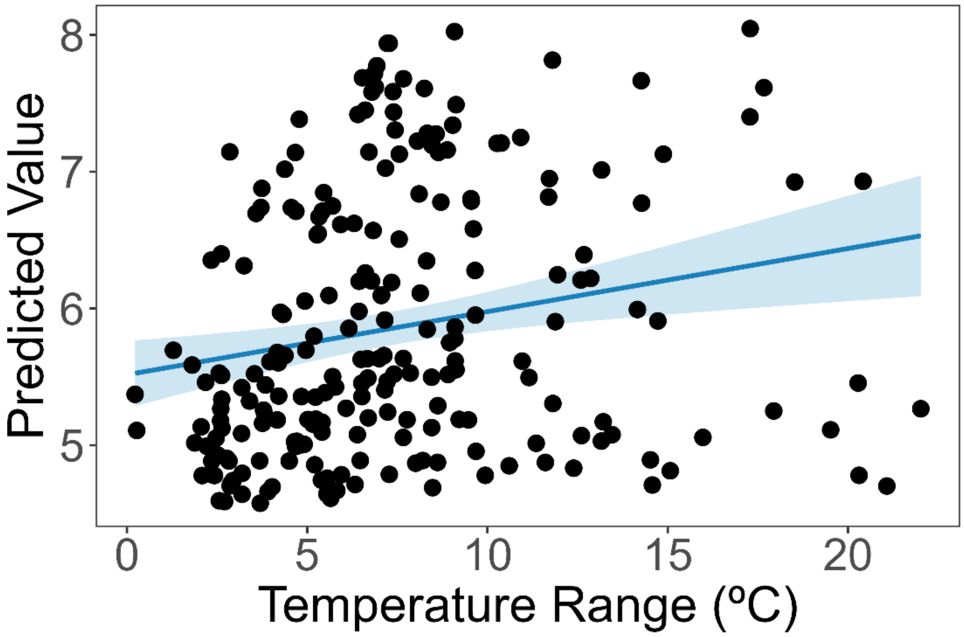


```
# ===================================
#                MESO-ES50-GAM
# ===================================

meso.ES50.intercept <- gam(ES50_mes ~ 1, data = Ecological_Data_Global_He
x_sp, family = "nb", method = "REML", select = TRUE)
gam.check(meso.ES50.intercept)
```

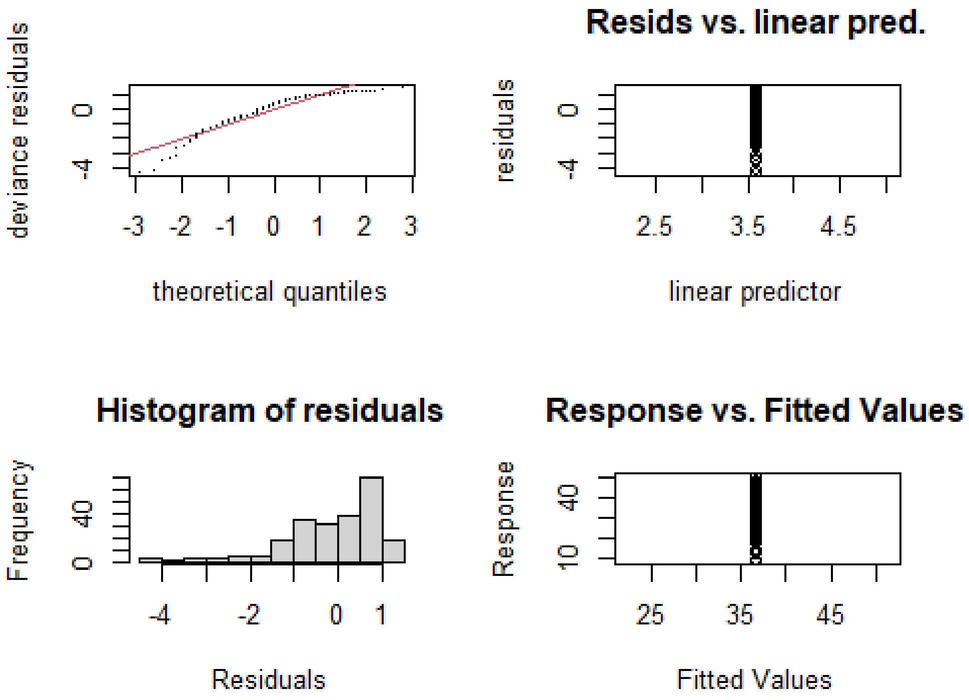


```
##
## Method: REML   Optimizer: outer newton
## full convergence after 4 iterations.
## Gradient range [-5.132154e-07,-5.132154e-07]
```

```
## (score 809.8765 & scale 1).
## Hessian positive definite, eigenvalue range [24.21747,24.21747].
## Model rank =  1 / 1
```

```
summary(meso.ES50.intercept)
```

```
##
## Family: Negative Binomial(37.397)
## Link function: log
##
## Formula:
## ES50_mes ~ 1
##
## Parametric coefficients:
##             Estimate Std. Error z value Pr(>|z|)
## (Intercept)  3.60665    0.01548   233.1   <2e-16 ***
## ---
## Signif. codes:  0 '***' 0.001 '**' 0.01 '*' 0.05 '.' 0.1 ' ' 1
##
##
## R-sq.(adj) =      0   Deviance explained = 5.44e-10%
## -REML = 809.88  Scale est. = 1         n = 225
```

```
meso.ES50.latlon <- gam(ES50_mes ~ s(Latitude, Longitude, bs = "sos"), da
ta = Ecological_Data_Global_Hex_sp, family = "nb", method = "REML", selec
t = TRUE)
gam.check(meso.ES50.latlon)
```

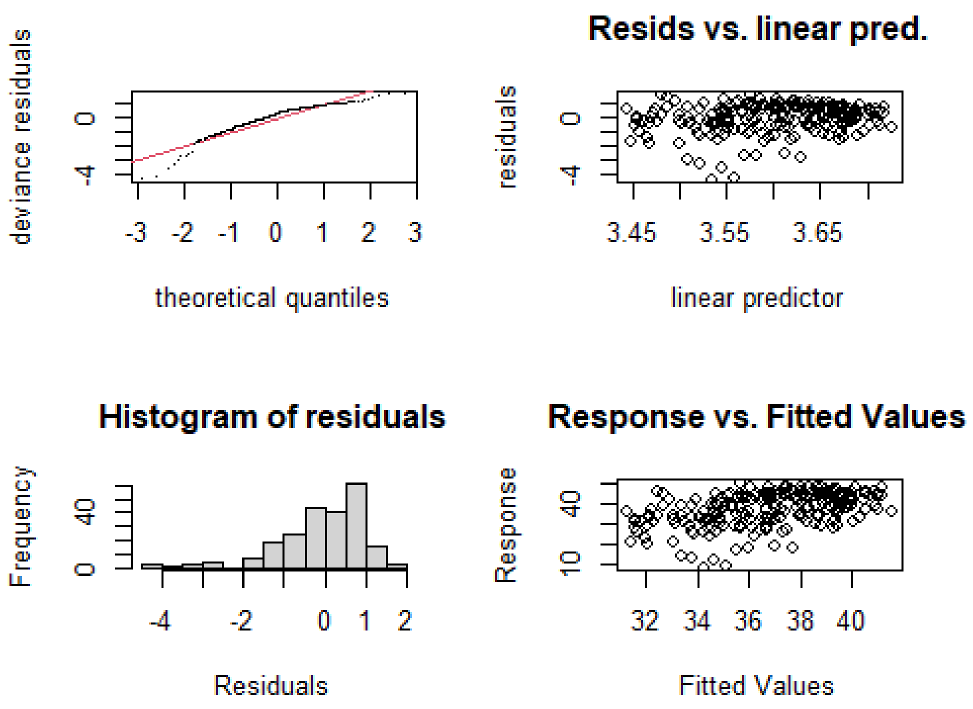


```
##
## Method: REML   Optimizer: outer newton
## full convergence after 4 iterations.
```

```
## Gradient range [-0.0008201761,-5.470664e-07]
## (score 804.902 & scale 1).
## Hessian positive definite, eigenvalue range [0.9861168,14.04282].
## Model rank =  50 / 50
##
## Basis dimension (k) checking results. Low p-value (k-index<1) may
## indicate that k is too low, especially if edf is close to k'.
##
##                           k'  edf k-index p-value
## s(Latitude,Longitude) 49.0 12.3    0.79  <2e-16 ***
## ---
## Signif. codes:  0 '***' 0.001 '**' 0.01 '*' 0.05 '.' 0.1 ' ' 1
```

```
summary(meso.ES50.latlon)
```

```
##
## Family: Negative Binomial(54.29)
## Link function: log
##
## Formula:
## ES50_mes ~ s(Latitude, Longitude, bs = "sos")
##
## Parametric coefficients:
##             Estimate Std. Error z value Pr(>|z|)
## (Intercept)  3.60325    0.01425   252.8   <2e-16 ***
## ---
## Signif. codes:  0 '***' 0.001 '**' 0.01 '*' 0.05 '.' 0.1 ' ' 1
##
## Approximate significance of smooth terms:
##                         edf Ref.df Chi.sq  p-value
## s(Latitude,Longitude) 12.31     49  35.84 2.65e-05 ***
## ---
## Signif. codes:  0 '***' 0.001 '**' 0.01 '*' 0.05 '.' 0.1 ' ' 1
##
## R-sq.(adj) =   0.15   Deviance explained = 17.1%
## -REML =  804.9  Scale est. = 1         n = 225
```

```
meso.ES50.depth <- gam(ES50_mes ~ s(Latitude, Longitude, bs = "sos") + s(
Depth_Mean), data = Ecological_Data_Global_Hex_sp, family = "nb", method
= "REML", select = TRUE)
gam.check(meso.ES50.depth)
```

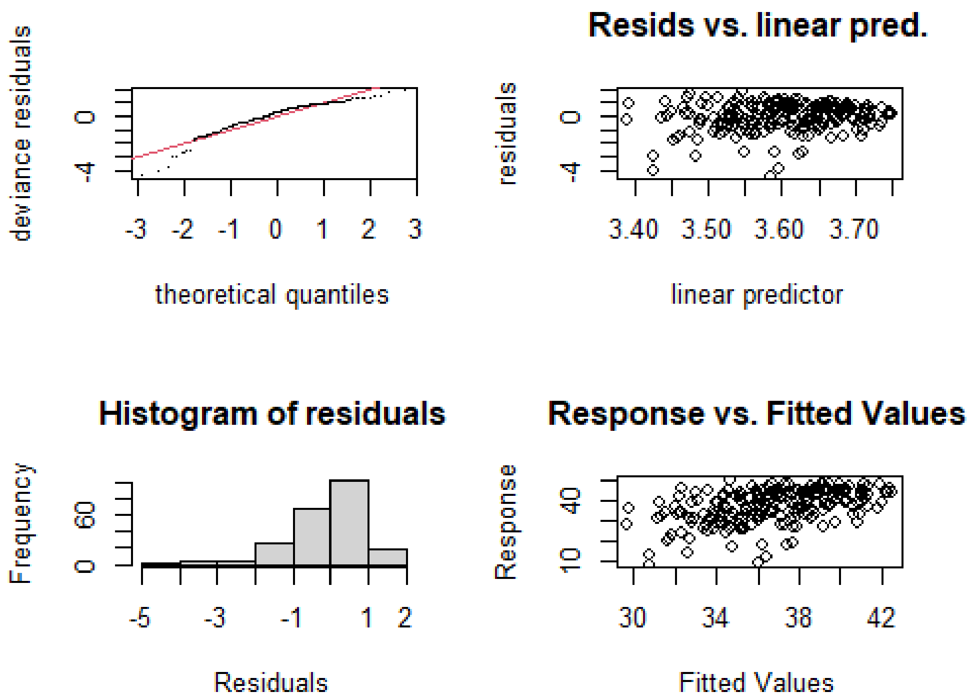


```
##
## Method: REML   Optimizer: outer newton
## full convergence after 6 iterations.
## Gradient range [-8.226758e-05,1.866699e-05]
## (score 802.2408 & scale 1).
## eigenvalue range [-1.865894e-05,12.85478].
## Model rank =  59 / 59
##
## Basis dimension (k) checking results. Low p-value (k-index<1) may
## indicate that k is too low, especially if edf is close to k'.
##
##                            k'   edf k-index p-value
## s(Latitude,Longitude) 49.0 10.3    0.79  <2e-16 ***
## s(Depth_Mean)          9.0  2.0    0.96    0.32
## ---
## Signif. codes:  0 '***' 0.001 '**' 0.01 '*' 0.05 '.' 0.1 ' ' 1

summary(meso.ES50.depth)

##
## Family: Negative Binomial(58.323)
## Link function: log
##
## Formula:
## ES50_mes ~ s(Latitude, Longitude, bs = "sos") + s(Depth_Mean)
##
## Parametric coefficients:
##             Estimate Std. Error z value Pr(>|z|)
## (Intercept)  3.60273    0.01406   256.3   <2e-16 ***
## ---
```

```
## Signif. codes:  0 '***' 0.001 '**' 0.01 '*' 0.05 '.' 0.1 ' ' 1
##
## Approximate significance of smooth terms:
##                               edf Ref.df Chi.sq  p-value
## s(Latitude,Longitude) 10.354     49 28.494 0.000171 ***
## s(Depth_Mean)          1.999      9  9.971 0.002186 **
## ---
## Signif. codes:  0 '***' 0.001 '**' 0.01 '*' 0.05 '.' 0.1 ' ' 1
##
## R-sq.(adj) =   0.17   Deviance explained =   19%
## -REML = 802.24  Scale est. = 1         n = 225
```

```
meso.ES50.margin <- gam(ES50_mes ~ s(Latitude, Longitude, bs = "sos") + s
(Margin_Sum), data = Ecological_Data_Global_Hex_sp, family = "nb", method
= "REML", select = TRUE)
gam.check(meso.ES50.margin)
```

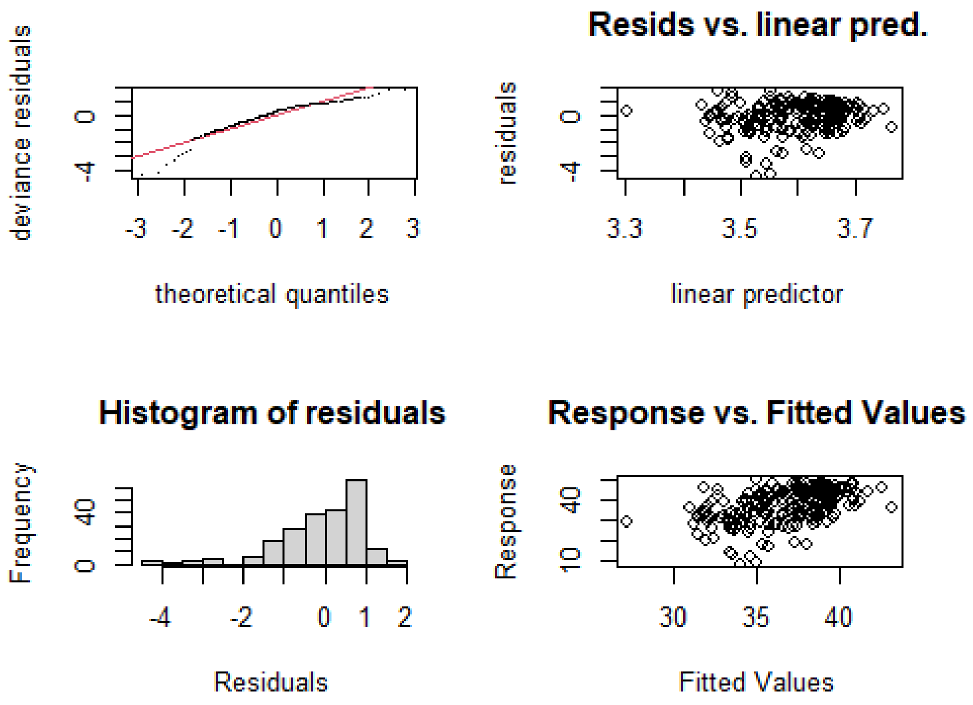


```
##
## Method: REML   Optimizer: outer newton
## full convergence after 6 iterations.
## Gradient range [-0.000131323,0.0001064507]
## (score 803.8878 & scale 1).
## Hessian positive definite, eigenvalue range [0.0001312673,12.99755].
## Model rank =  59 / 59
##
## Basis dimension (k) checking results. Low p-value (k-index<1) may
## indicate that k is too low, especially if edf is close to k'.
##
##                          k'   edf k-index p-value
## s(Latitude,Longitude) 49.00 11.84    0.79  <2e-16 ***
```

```
## s(Margin_Sum)          9.00  1.58    1.05    0.78
## ---
## Signif. codes:  0 '***' 0.001 '**' 0.01 '*' 0.05 '.' 0.1 ' ' 1
```

```
summary(meso.ES50.margin)
```

```
##
## Family: Negative Binomial(57.248)
## Link function: log
##
## Formula:
## ES50_mes ~ s(Latitude, Longitude, bs = "sos") + s(Margin_Sum)
##
## Parametric coefficients:
##             Estimate Std. Error z value Pr(>|z|)
## (Intercept)  3.60289    0.01411   255.4   <2e-16 ***
## ---
## Signif. codes:  0 '***' 0.001 '**' 0.01 '*' 0.05 '.' 0.1 ' ' 1
##
## Approximate significance of smooth terms:
##                            edf Ref.df Chi.sq  p-value
## s(Latitude,Longitude) 11.841     49 35.067 2.38e-05 ***
## s(Margin_Sum)          1.578      9  4.825   0.0343 *
## ---
## Signif. codes:  0 '***' 0.001 '**' 0.01 '*' 0.05 '.' 0.1 ' ' 1
##
## R-sq.(adj) =  0.166   Deviance explained = 18.8%
## -REML = 803.89  Scale est. = 1         n = 225
```

```
meso.ES50.current <- gam(ES50_mes ~ s(Latitude, Longitude, bs = "sos") +
s(CurVel_Mean), data = Ecological_Data_Global_Hex_sp, family = "nb", meth
od = "REML", select = TRUE)
gam.check(meso.ES50.current)
```

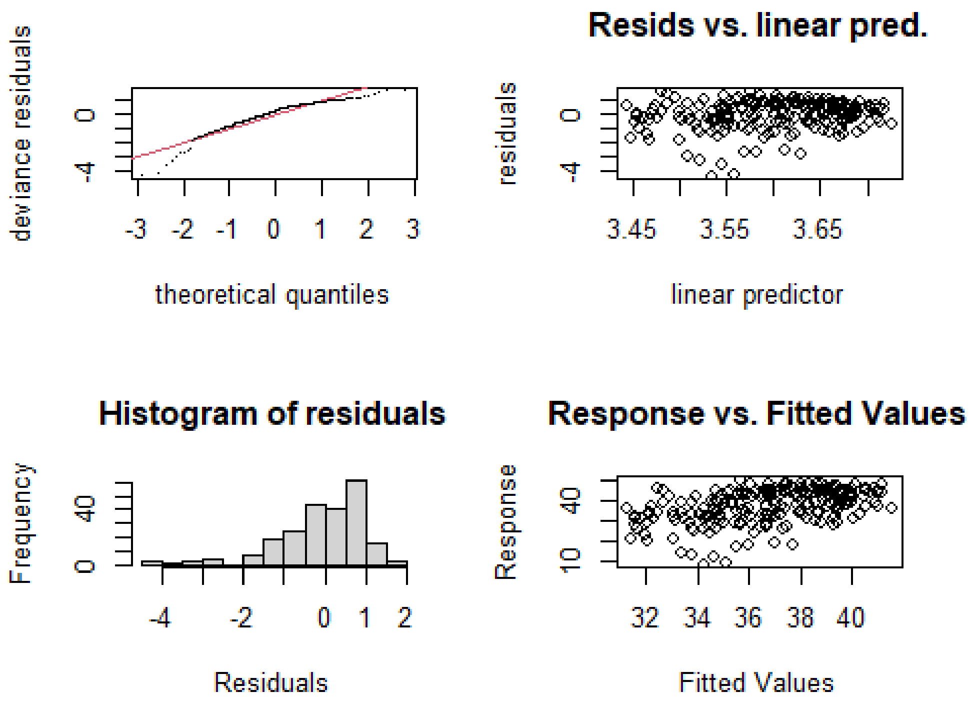


```
##
## Method: REML   Optimizer: outer newton
## full convergence after 8 iterations.
## Gradient range [-0.0002187214,8.057292e-05]
## (score 804.9023 & scale 1).
## Hessian positive definite, eigenvalue range [1.046262e-05,14.04124].
## Model rank =  59 / 59
##
## Basis dimension (k) checking results. Low p-value (k-index<1) may
## indicate that k is too low, especially if edf is close to k'.
##
##                               k'      edf k-index p-value
## s(Latitude,Longitude) 49.00000 12.31479    0.79  <2e-16 ***
## s(CurVel_Mean)         9.00000  0.00057    0.89   0.045 *
## ---
## Signif. codes:  0 '***' 0.001 '**' 0.01 '*' 0.05 '.' 0.1 ' ' 1
```

```
summary(meso.ES50.current)
```

```
##
## Family: Negative Binomial(54.294)
## Link function: log
##
## Formula:
## ES50_mes ~ s(Latitude, Longitude, bs = "sos") + s(CurVel_Mean)
##
## Parametric coefficients:
##             Estimate Std. Error z value Pr(>|z|)
## (Intercept)  3.60325    0.01425   252.8   <2e-16 ***
## ---
```

```
## Signif. codes:  0 '***' 0.001 '**' 0.01 '*' 0.05 '.' 0.1 ' ' 1
##
## Approximate significance of smooth terms:
##                              edf Ref.df Chi.sq  p-value
## s(Latitude,Longitude) 1.231e+01     49  35.84 2.65e-05 ***
## s(CurVel_Mean)        5.695e-04      9   0.00    0.788
## ---
## Signif. codes:  0 '***' 0.001 '**' 0.01 '*' 0.05 '.' 0.1 ' ' 1
##
## R-sq.(adj) =   0.15   Deviance explained = 17.1%
## -REML =  804.9  Scale est. = 1         n = 225
```

```
meso.ES50.humimp <- gam(ES50_mes ~ s(Latitude, Longitude, bs = "sos") + s
(HumImp_Mean), data = Ecological_Data_Global_Hex_sp, family = "nb", metho
d = "REML", select = TRUE)
gam.check(meso.ES50.humimp)
```

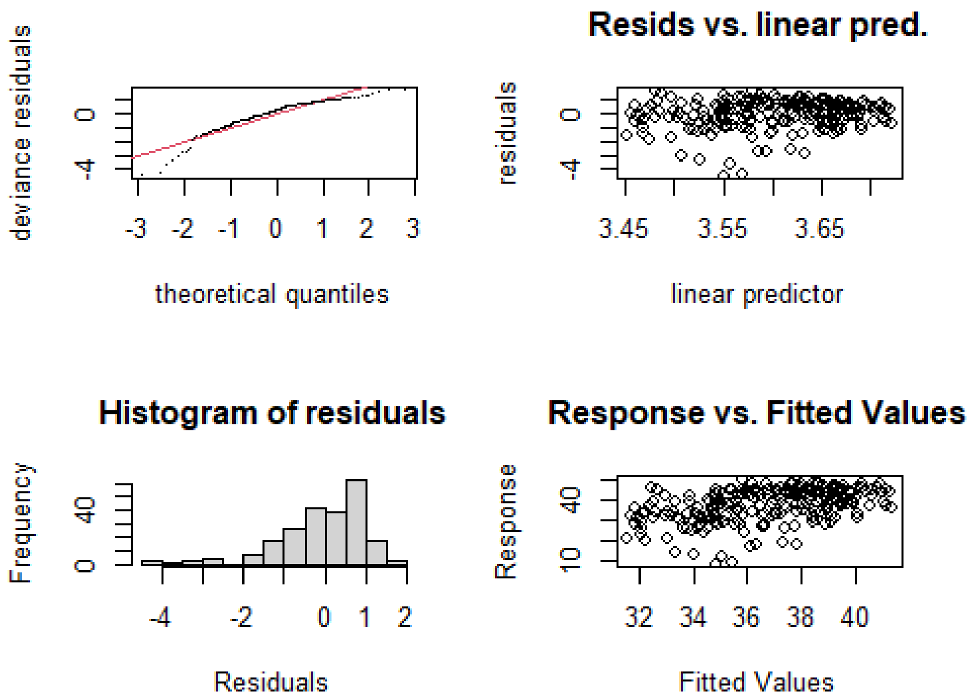


```
##
## Method: REML   Optimizer: outer newton
## full convergence after 9 iterations.
## Gradient range [-0.000256611,2.577551e-05]
## (score 804.8565 & scale 1).
## Hessian positive definite, eigenvalue range [0.0002565636,14.37238].
## Model rank =  59 / 59
##
## Basis dimension (k) checking results. Low p-value (k-index<1) may
## indicate that k is too low, especially if edf is close to k'.
##
##                           k'    edf k-index p-value
## s(Latitude,Longitude) 49.000 11.425    0.78  <2e-16 ***
```

```
## s(HumImp_Mean)         9.000  0.359    1.00    0.42
## ---
## Signif. codes:  0 '***' 0.001 '**' 0.01 '*' 0.05 '.' 0.1 ' ' 1
```

```
summary(meso.ES50.humimp)
```

```
##
## Family: Negative Binomial(53.425)
## Link function: log
##
## Formula:
## ES50_mes ~ s(Latitude, Longitude, bs = "sos") + s(HumImp_Mean)
##
## Parametric coefficients:
##             Estimate Std. Error z value Pr(>|z|)
## (Intercept)   3.6034     0.0143     252   <2e-16 ***
## ---
## Signif. codes:  0 '***' 0.001 '**' 0.01 '*' 0.05 '.' 0.1 ' ' 1
##
## Approximate significance of smooth terms:
##                            edf Ref.df Chi.sq p-value
## s(Latitude,Longitude) 11.425     49 31.529 9.3e-05 ***
## s(HumImp_Mean)         0.359      9  0.556   0.168
## ---
## Signif. codes:  0 '***' 0.001 '**' 0.01 '*' 0.05 '.' 0.1 ' ' 1
##
## R-sq.(adj) =  0.144   Deviance explained = 16.4%
## -REML = 804.86  Scale est. = 1         n = 225
```

```
meso.ES50.nitrate <- gam(ES50_mes ~ s(Latitude, Longitude, bs = "sos") +
s(Nitrate_Mean), data = Ecological_Data_Global_Hex_sp, family = "nb", met
hod = "REML", select = TRUE)
gam.check(meso.ES50.nitrate)
```

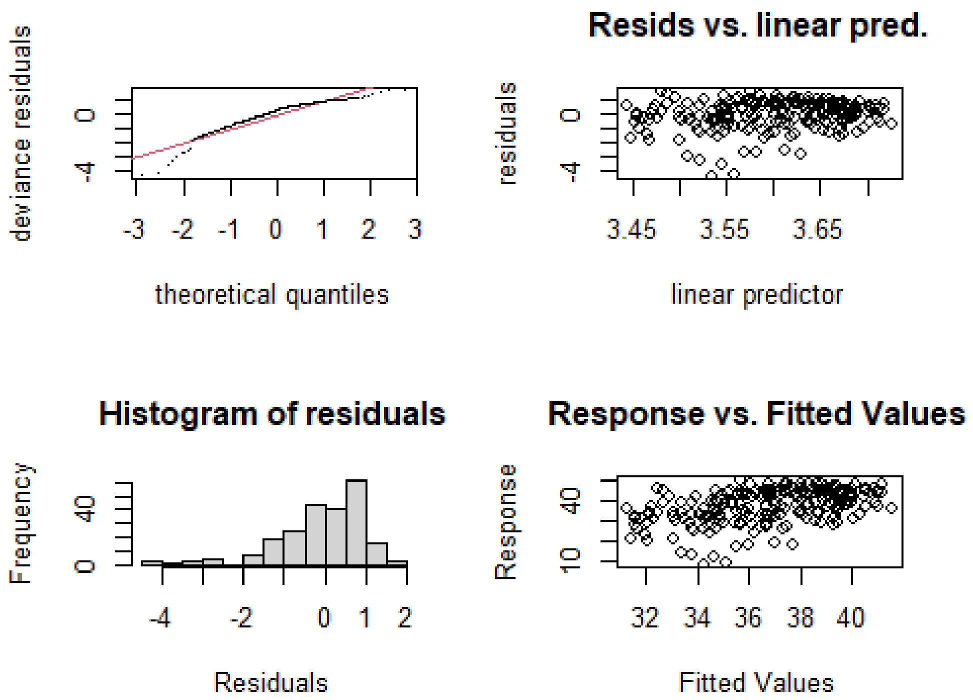


```
##
## Method: REML   Optimizer: outer newton
## full convergence after 8 iterations.
## Gradient range [-0.0002601955,7.129034e-05]
## (score 804.9023 & scale 1).
## Hessian positive definite, eigenvalue range [9.185918e-06,14.04124].
## Model rank =  59 / 59
##
## Basis dimension (k) checking results. Low p-value (k-index<1) may
## indicate that k is too low, especially if edf is close to k'.
##
##                               k'      edf k-index p-value
## s(Latitude,Longitude) 4.90e+01 1.23e+01    0.79  <2e-16 ***
## s(Nitrate_Mean)       9.00e+00 6.42e-04    1.06     0.8
## ---
## Signif. codes:  0 '***' 0.001 '**' 0.01 '*' 0.05 '.' 0.1 ' ' 1
```

```
summary(meso.ES50.nitrate)
```

```
##
## Family: Negative Binomial(54.294)
## Link function: log
##
## Formula:
## ES50_mes ~ s(Latitude, Longitude, bs = "sos") + s(Nitrate_Mean)
##
## Parametric coefficients:
##             Estimate Std. Error z value Pr(>|z|)
## (Intercept)  3.60325    0.01425   252.8   <2e-16 ***
## ---
```

```
## Signif. codes:  0 '***' 0.001 '**' 0.01 '*' 0.05 '.' 0.1 ' ' 1
##
## Approximate significance of smooth terms:
##                               edf Ref.df Chi.sq  p-value
## s(Latitude,Longitude) 1.231e+01     49  35.84 2.65e-05 ***
## s(Nitrate_Mean)       6.421e-04      9   0.00    0.822
## ---
## Signif. codes:  0 '***' 0.001 '**' 0.01 '*' 0.05 '.' 0.1 ' ' 1
##
## R-sq.(adj) =   0.15   Deviance explained = 17.1%
## -REML =  804.9  Scale est. = 1         n = 225
```

```
meso.ES50.PrimProd <- gam(ES50_mes ~ s(Latitude, Longitude, bs = "sos") +
s(PrimProd_Mean), data = Ecological_Data_Global_Hex_sp, family = "nb", me
thod = "REML", select = TRUE)
gam.check(meso.ES50.PrimProd)
```

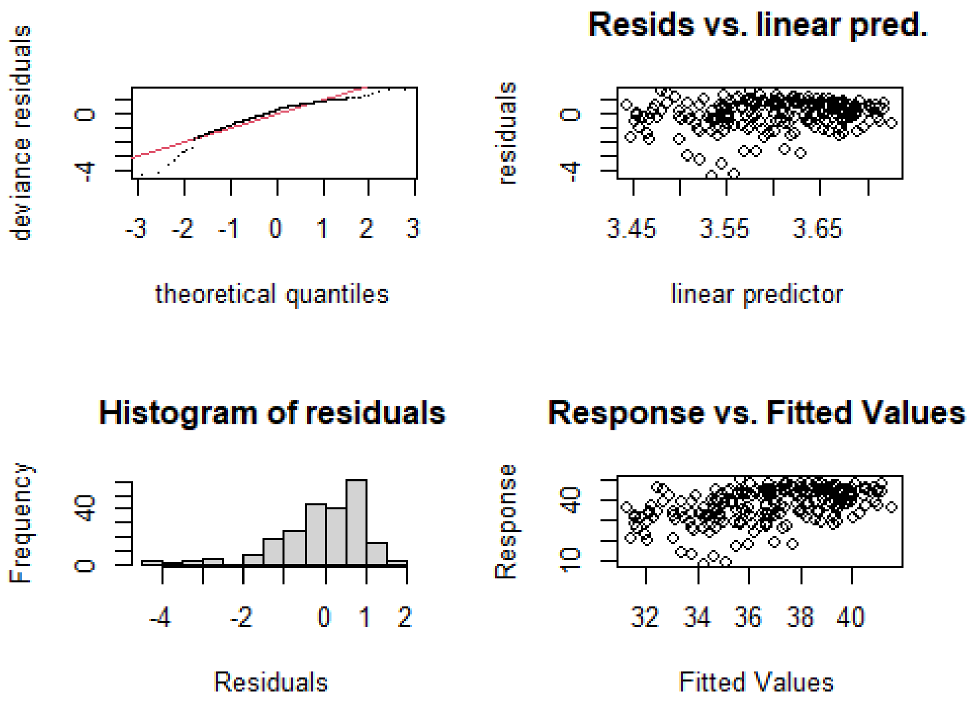


```
##
## Method: REML   Optimizer: outer newton
## full convergence after 8 iterations.
## Gradient range [-0.0002319808,4.298289e-05]
## (score 804.9023 & scale 1).
## Hessian positive definite, eigenvalue range [1.199057e-05,14.0412].
## Model rank =  59 / 59
##
## Basis dimension (k) checking results. Low p-value (k-index<1) may
## indicate that k is too low, especially if edf is close to k'.
##
##                             k'      edf k-index p-value
## s(Latitude,Longitude) 4.90e+01 1.23e+01    0.79  <2e-16 ***
```

```
## s(PrimProd_Mean)      9.00e+00 7.85e-04    0.89   0.035 *
## ---
## Signif. codes:  0 '***' 0.001 '**' 0.01 '*' 0.05 '.' 0.1 ' ' 1
```

```
summary(meso.ES50.PrimProd)
```

```
##
## Family: Negative Binomial(54.294)
## Link function: log
##
## Formula:
## ES50_mes ~ s(Latitude, Longitude, bs = "sos") + s(PrimProd_Mean)
##
## Parametric coefficients:
##             Estimate Std. Error z value Pr(>|z|)
## (Intercept)  3.60325    0.01425   252.8   <2e-16 ***
## ---
## Signif. codes:  0 '***' 0.001 '**' 0.01 '*' 0.05 '.' 0.1 ' ' 1
##
## Approximate significance of smooth terms:
##                             edf Ref.df Chi.sq  p-value
## s(Latitude,Longitude) 1.231e+01     49  35.84 2.65e-05 ***
## s(PrimProd_Mean)      7.848e-04      9   0.00     0.66
## ---
## Signif. codes:  0 '***' 0.001 '**' 0.01 '*' 0.05 '.' 0.1 ' ' 1
##
## R-sq.(adj) =   0.15   Deviance explained = 17.1%
## -REML =  804.9  Scale est. = 1         n = 225
```

```
meso.ES50.ThemM <- gam(ES50_mes ~ s(Latitude, Longitude, bs = "sos") + s(
ThemM_mean), data = Ecological_Data_Global_Hex_sp, family = "nb", method
= "REML", select = TRUE)
gam.check(meso.ES50.ThemM)
```

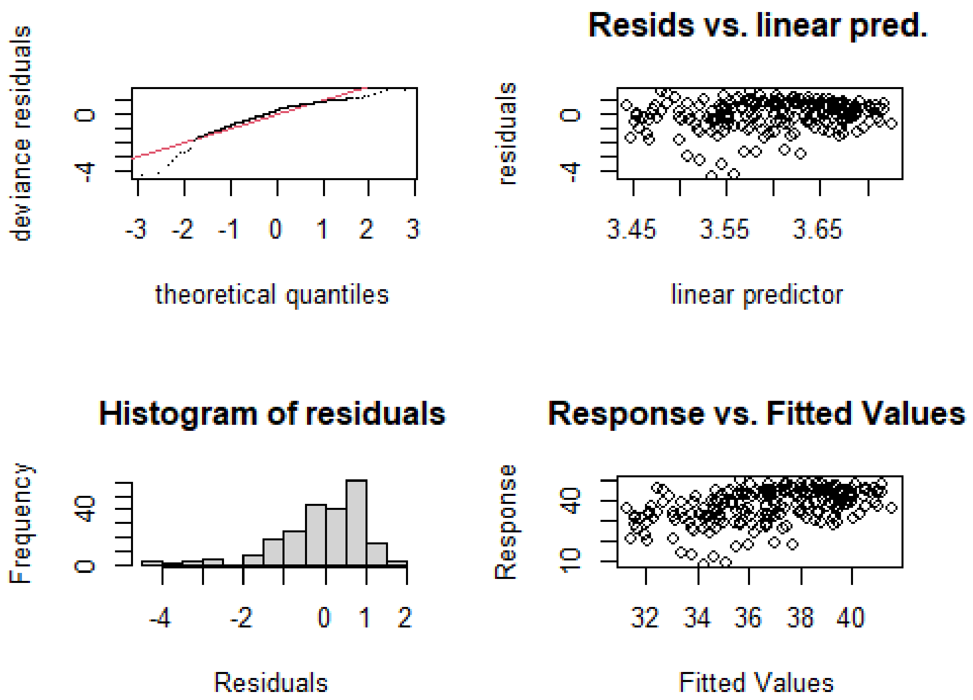


```
##
## Method: REML   Optimizer: outer newton
## full convergence after 8 iterations.
## Gradient range [-0.0001870482,0.0001042793]
## (score 804.9022 & scale 1).
## Hessian positive definite, eigenvalue range [4.66661e-06,14.04123].
## Model rank =  59 / 59
##
## Basis dimension (k) checking results. Low p-value (k-index<1) may
## indicate that k is too low, especially if edf is close to k'.
##
##                              k'      edf k-index p-value
## s(Latitude,Longitude) 4.90e+01 1.23e+01    0.79  <2e-16 ***
## s(ThemM_mean)         9.00e+00 5.56e-04    1.04    0.69
## ---
## Signif. codes:  0 '***' 0.001 '**' 0.01 '*' 0.05 '.' 0.1 ' ' 1
```

```
summary(meso.ES50.ThemM)
```

```
##
## Family: Negative Binomial(54.294)
## Link function: log
##
## Formula:
## ES50_mes ~ s(Latitude, Longitude, bs = "sos") + s(ThemM_mean)
##
## Parametric coefficients:
##             Estimate Std. Error z value Pr(>|z|)
## (Intercept)  3.60325    0.01425   252.8   <2e-16 ***
## ---
```

```
## Signif. codes:  0 '***' 0.001 '**' 0.01 '*' 0.05 '.' 0.1 ' ' 1
##
## Approximate significance of smooth terms:
##                                edf Ref.df Chi.sq  p-value
## s(Latitude,Longitude) 1.231e+01     49  35.84 2.65e-05 ***
## s(ThemM_mean)         5.562e-04      9   0.00    0.578
## ---
## Signif. codes:  0 '***' 0.001 '**' 0.01 '*' 0.05 '.' 0.1 ' ' 1
##
## R-sq.(adj) =   0.15   Deviance explained = 17.1%
## -REML =  804.9  Scale est. = 1         n = 225
```

```
meso.ES50.ThemR <- gam(ES50_mes ~ s(Latitude, Longitude, bs = "sos") + s(
ThemR_mean), data = Ecological_Data_Global_Hex_sp, family = "nb", method
= "REML", select = TRUE)
gam.check(meso.ES50.ThemR)
```

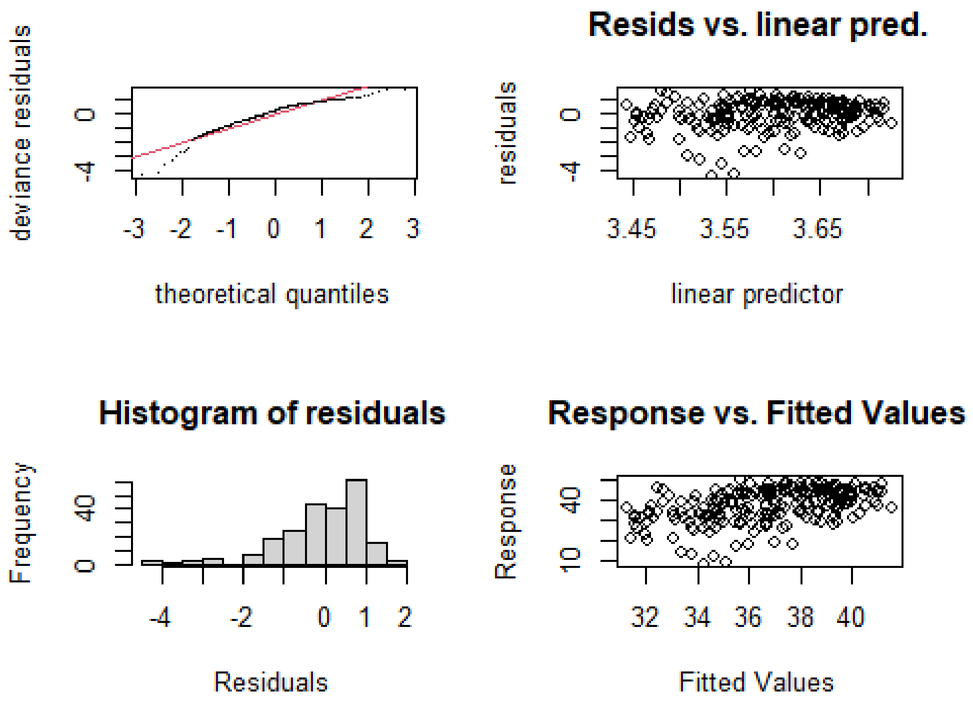


```
##
## Method: REML   Optimizer: outer newton
## full convergence after 8 iterations.
## Gradient range [-0.0002190474,4.506027e-05]
## (score 804.9023 & scale 1).
## Hessian positive definite, eigenvalue range [5.234245e-06,14.04119].
## Model rank =  59 / 59
##
## Basis dimension (k) checking results. Low p-value (k-index<1) may
## indicate that k is too low, especially if edf is close to k'.
##
##                             k'      edf k-index p-value
## s(Latitude,Longitude) 49.00000 12.31467    0.79  <2e-16 ***
```

```
## s(ThemR_mean)          9.00000  0.00108    1.04    0.68
## ---
## Signif. codes:  0 '***' 0.001 '**' 0.01 '*' 0.05 '.' 0.1 ' ' 1
```

```
summary(meso.ES50.ThemR)
```

```
##
## Family: Negative Binomial(54.294)
## Link function: log
##
## Formula:
## ES50_mes ~ s(Latitude, Longitude, bs = "sos") + s(ThemR_mean)
##
## Parametric coefficients:
##             Estimate Std. Error z value Pr(>|z|)
## (Intercept)  3.60325    0.01425   252.8   <2e-16 ***
## ---
## Signif. codes:  0 '***' 0.001 '**' 0.01 '*' 0.05 '.' 0.1 ' ' 1
##
## Approximate significance of smooth terms:
##                              edf Ref.df Chi.sq  p-value
## s(Latitude,Longitude) 12.314674     49 35.843 2.65e-05 ***
## s(ThemR_mean)          0.001084      9  0.001    0.483
## ---
## Signif. codes:  0 '***' 0.001 '**' 0.01 '*' 0.05 '.' 0.1 ' ' 1
##
## R-sq.(adj) =   0.15   Deviance explained = 17.1%
## -REML =  804.9  Scale est. = 1         n = 225
```

```
meso.ES50.env <- gam(ES50_mes ~ s(Latitude, Longitude, bs = "sos") + s(De
pth_Mean) +  s(Margin_Sum) + s(CurVel_Mean) + s(HumImp_Mean) + s(Nitrate_
Mean) + s(PrimProd_Mean) + s(ThemM_mean) + s(ThemR_mean), data = Ecologic
al_Data_Global_Hex_sp, family = "nb", method = "REML", select = TRUE)
gam.check(meso.ES50.env)
```

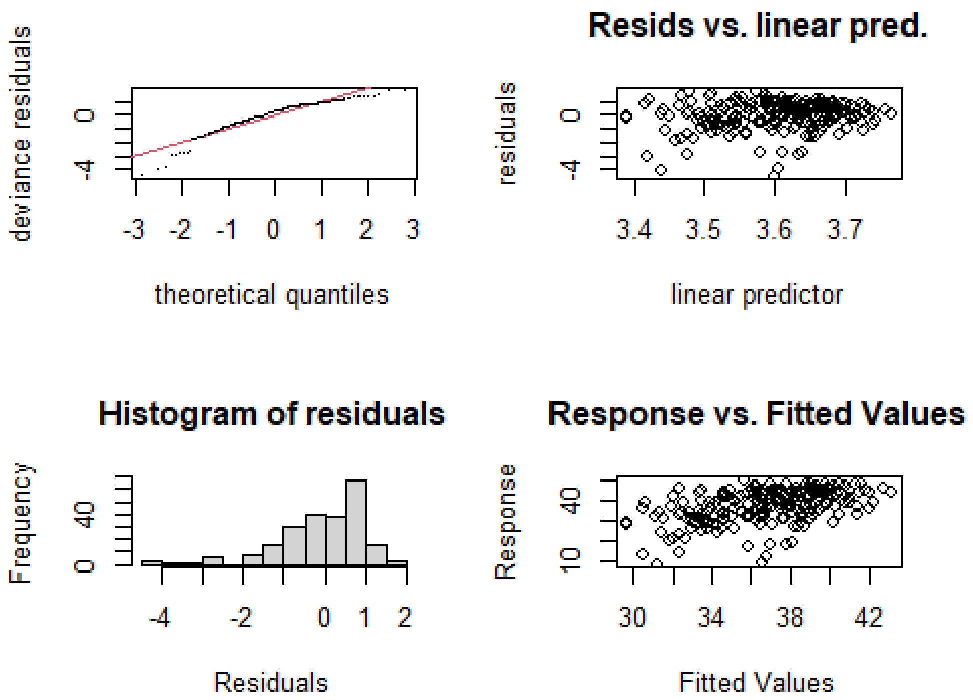


```
##
## Method: REML   Optimizer: outer newton
## full convergence after 11 iterations.
## Gradient range [-0.0001212376,3.183136e-05]
## (score 801.8442 & scale 1).
## Hessian positive definite, eigenvalue range [1.467606e-06,12.77644].
## Model rank =  122 / 122
##
## Basis dimension (k) checking results. Low p-value (k-index<1) may
## indicate that k is too low, especially if edf is close to k'.
##
##                             k'      edf k-index p-value
## s(Latitude,Longitude) 4.90e+01 9.01e+00    0.79  <2e-16 ***
## s(Depth_Mean)         9.00e+00 1.94e+00    0.96    0.22
## s(Margin_Sum)         9.00e+00 9.07e-01    1.04    0.73
## s(CurVel_Mean)        9.00e+00 2.19e-04    0.88    0.05 *
## s(HumImp_Mean)        9.00e+00 6.15e-01    0.97    0.30
## s(Nitrate_Mean)       9.00e+00 2.42e-04    1.05    0.80
## s(PrimProd_Mean)      9.00e+00 4.45e-04    0.93    0.18
## s(ThemM_mean)         9.00e+00 1.51e-04    1.04    0.65
## s(ThemR_mean)         9.00e+00 5.11e-04    1.04    0.71
## ---
## Signif. codes:  0 '***' 0.001 '**' 0.01 '*' 0.05 '.' 0.1 ' ' 1
```

```
summary(meso.ES50.env)
```

```
##
## Family: Negative Binomial(58.593)
## Link function: log
##
```

```
## Formula:
## ES50_mes ~ s(Latitude, Longitude, bs = "sos") + s(Depth_Mean) +
##     s(Margin_Sum) + s(CurVel_Mean) + s(HumImp_Mean) + s(Nitrate_Mean)
+
##     s(PrimProd_Mean) + s(ThemM_mean) + s(ThemR_mean)
##
## Parametric coefficients:
##             Estimate Std. Error z value Pr(>|z|)
## (Intercept)  3.60269    0.01404   256.5   <2e-16 ***
## ---
## Signif. codes:  0 '***' 0.001 '**' 0.01 '*' 0.05 '.' 0.1 ' ' 1
##
## Approximate significance of smooth terms:
##                              edf Ref.df Chi.sq  p-value
## s(Latitude,Longitude) 9.0109310     49 24.171 0.000397 ***
## s(Depth_Mean)         1.9369475      9  9.076 0.002334 **
## s(Margin_Sum)         0.9066358      9  1.421 0.169826
## s(CurVel_Mean)        0.0002187      9  0.000 0.568339
## s(HumImp_Mean)        0.6147096      9  1.591 0.078370 .
## s(Nitrate_Mean)       0.0002419      9  0.000 0.748987
## s(PrimProd_Mean)      0.0004445      9  0.000 0.450532
## s(ThemM_mean)         0.0001511      9  0.000 0.611362
## s(ThemR_mean)         0.0005110      9  0.000 0.362541
## ---
## Signif. codes:  0 '***' 0.001 '**' 0.01 '*' 0.05 '.' 0.1 ' ' 1
##
## R-sq.(adj) =  0.171   Deviance explained = 19.1%
## -REML = 801.84  Scale est. = 1         n = 225

meso.ES50.models <- list(Intercept = meso.ES50.intercept,
                         LatLon = meso.ES50.latlon,
                         Depth = meso.ES50.depth,
                         ConMar = meso.ES50.margin,
                         CurVel = meso.ES50.current,
                         HumImp = meso.ES50.humimp,
                         Nitrate = meso.ES50.nitrate,
                         PriPro = meso.ES50.PrimProd,
                         TemMea = meso.ES50.ThemM,
                         TheRan = meso.ES50.ThemR,
                         Environment = meso.ES50.env)
meso.ES50.aic.df <- data.frame(Model = names(meso.ES50.models),
                               AIC = sapply(meso.ES50.models, function(x)
x$aic),
                               akaike.weights(sapply(meso.ES50.models, fu
nction(x) x$aic)))

meso.ES50.aic.df <- meso.ES50.aic.df[order(meso.ES50.aic.df$AIC),]
meso.ES50.aic.df$Cumulative.Weight <- cumsum(meso.ES50.aic.df$weights)

kable(meso.ES50.aic.df, row.names = FALSE)
```

Model	AIC	deltaAIC	rel.LL	weights	Cumulative.Weight
Environment	1588.753	0.0000000	1.0000000	0.4074133	0.4074133
Depth	1589.030	0.2769343	0.8706918	0.3547315	0.7621448
ConMar	1591.767	3.0140548	0.2215676	0.0902696	0.8524144
TemMea	1594.581	5.8279296	0.0542602	0.0221063	0.8745208
TheRan	1594.582	5.8282608	0.0542512	0.0221027	0.8966234
Nitrate	1594.582	5.8284382	0.0542464	0.0221007	0.9187241
PriPro	1594.582	5.8284528	0.0542460	0.0221005	0.9408246
CurVel	1594.582	5.8284948	0.0542448	0.0221001	0.9629247
LatLon	1594.582	5.8286216	0.0542414	0.0220987	0.9850234
HumImp	1595.360	6.6067721	0.0367585	0.0149759	0.9999993
Intercept	1615.254	26.5005357	0.0000018	0.0000007	1.0000000

```
#write.csv(meso.ES50.aic.df, file = "meso.ES50.aic.GAM.csv")

#Plots for Es50, Meso
ggplot(Ecological_Data_Global_Hex_sp, aes(x = Depth_Mean, y = predict(mes
o.ES50.depth, Ecological_Data_Global_Hex_sp))) +
  geom_smooth(method = "gam", formula = y ~ x, color = "#1a80bb", fill =
"#85bede") +  # Add a smooth dark blue line with light blue shadow
  geom_point(size = 3) +  # Add scatter plot points
  theme_bw() +  # Use the black and white theme
  labs(
    x = "Depth (m)",  # Shorten the x-axis title
    y = "Predicted Value"  # Shorten the y-axis title
  ) +
  theme(
    panel.grid.minor = element_blank(),
    panel.grid.major = element_blank(),
    axis.text.x = element_text(size =20),  # Increase x-axis text size
    axis.text.y = element_text(size = 20),  # Increase y-axis text size
    axis.title.x = element_text(size = 22), # Increase x-axis title size
    axis.title.y = element_text(size = 22)  # Increase y-axis title size
  )
```

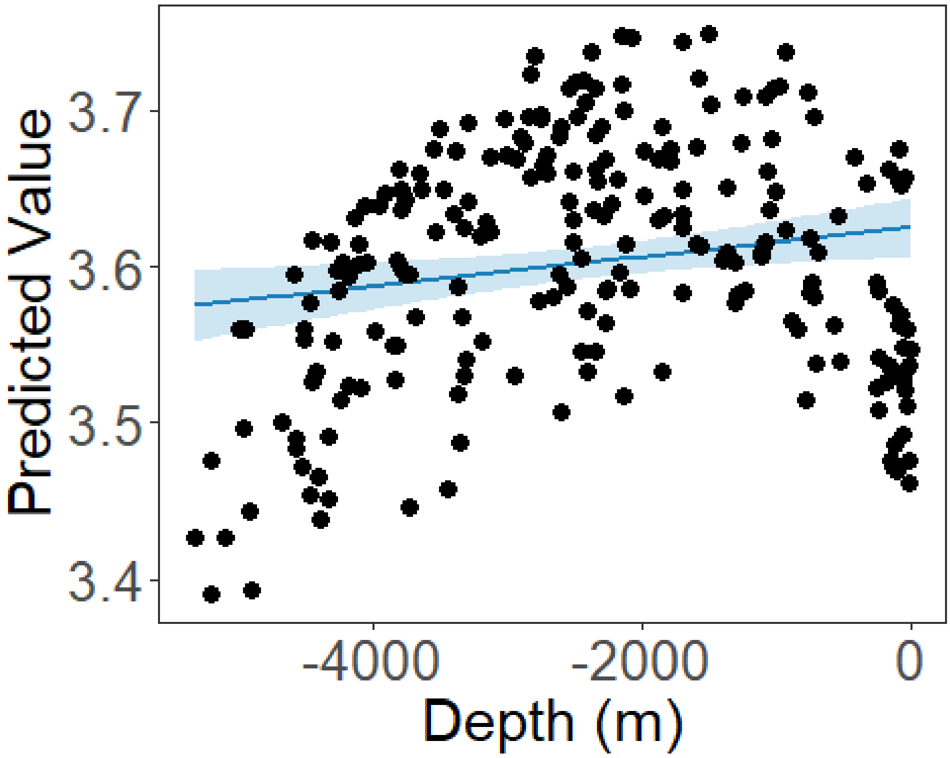


```r
ggplot(Ecological_Data_Global_Hex_sp, aes(x = Margin_Sum, y = predict(meso.ES50.margin, Ecological_Data_Global_Hex_sp))) +
  geom_smooth(method = "gam", formula = y ~ x, color = "#1a80bb", fill = "#85bede") +  # Add a smooth dark blue line with light blue shadow
  geom_point(size = 3) +  # Add scatter plot points
  theme_bw() +  # Use the black and white theme
  labs(
    x = "Continental Margin (km2)",  # Shorten the x-axis title
    y = "Predicted Value"  # Shorten the y-axis title
  ) +
  theme(
    panel.grid.minor = element_blank(),
    panel.grid.major = element_blank(),
    axis.text.x = element_text(size =20),  # Increase x-axis text size
    axis.text.y = element_text(size = 20),  # Increase y-axis text size
    axis.title.x = element_text(size = 22), # Increase x-axis title size
    axis.title.y = element_text(size = 22)  # Increase y-axis title size
  )
```

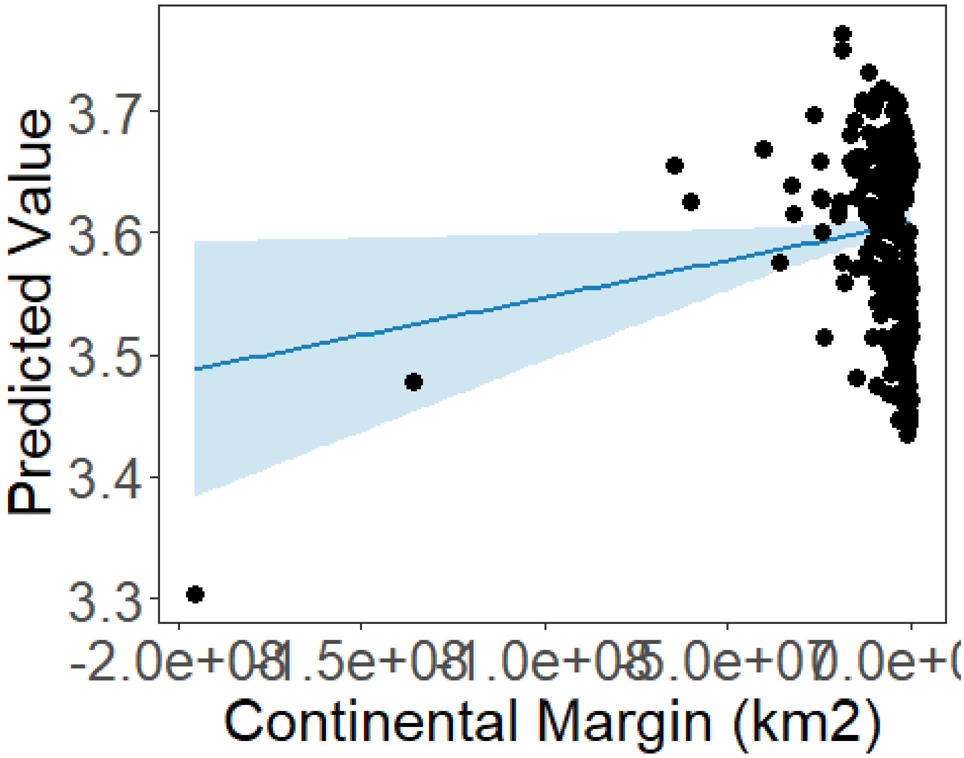


```
ggplot(Ecological_Data_Global_Hex_sp, aes(x = CurVel_Mean, y = predict(me
so.ES50.current, Ecological_Data_Global_Hex_sp))) +
  geom_smooth(method = "gam", formula = y ~ x, color = "#1a80bb", fill =
"#85bede") +  # Add a smooth dark blue line with light blue shadow
  geom_point(size = 3) +  # Add scatter plot points
  theme_bw() +  # Use the black and white theme
  labs(
    x = "Current Velocity (m.s-1)",  # Shorten the x-axis title
    y = "Predicted Value"  # Shorten the y-axis title
  ) +
  theme(
    panel.grid.minor = element_blank(),
    panel.grid.major = element_blank(),
    axis.text.x = element_text(size =20),  # Increase x-axis text size
    axis.text.y = element_text(size = 20),  # Increase y-axis text size
    axis.title.x = element_text(size = 22), # Increase x-axis title size
    axis.title.y = element_text(size = 22)  # Increase y-axis title size
  )
```

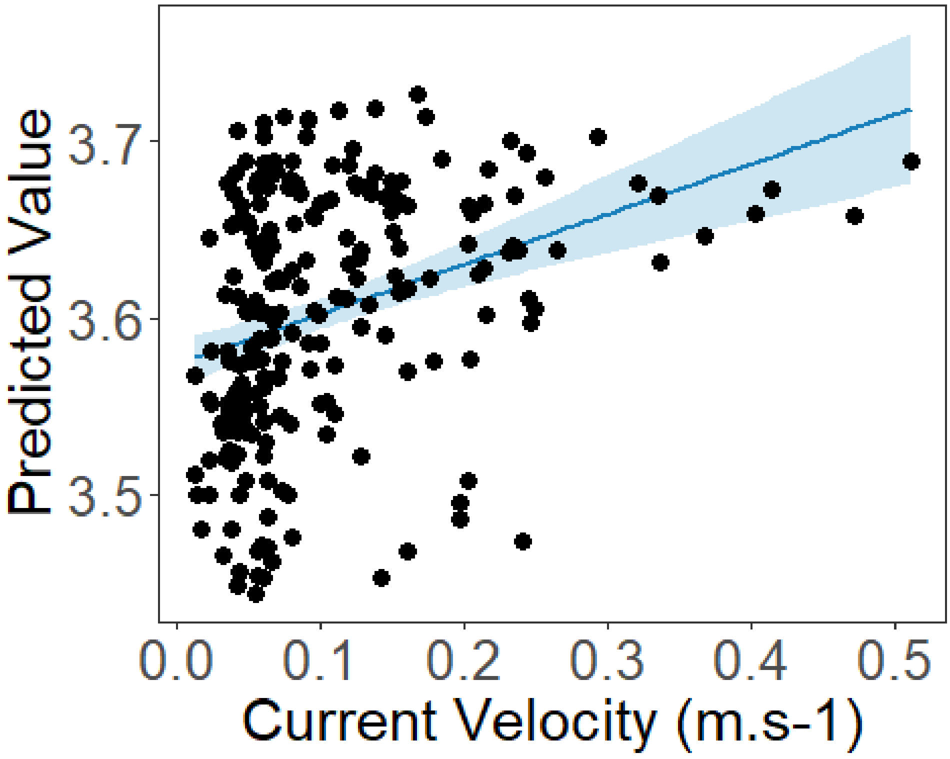


```
ggplot(Ecological_Data_Global_Hex_sp, aes(x = HumImp_Mean, y = predict(me
so.ES50.humimp, Ecological_Data_Global_Hex_sp))) +
  geom_smooth(method = "gam", formula = y ~ x, color = "#1a80bb", fill =
"#85bede") +  # Add a smooth dark blue line with light blue shadow
  geom_point(size = 3) +  # Add scatter plot points
  theme_bw() +  # Use the black and white theme
  labs(
    x = "Human Impact",  # Shorten the x-axis title
    y = "Predicted Value"  # Shorten the y-axis title
  ) +
  theme(
    panel.grid.minor = element_blank(),
    panel.grid.major = element_blank(),
    axis.text.x = element_text(size =20),  # Increase x-axis text size
    axis.text.y = element_text(size = 20),  # Increase y-axis text size
    axis.title.x = element_text(size = 22), # Increase x-axis title size
    axis.title.y = element_text(size = 22)  # Increase y-axis title size
  )
```

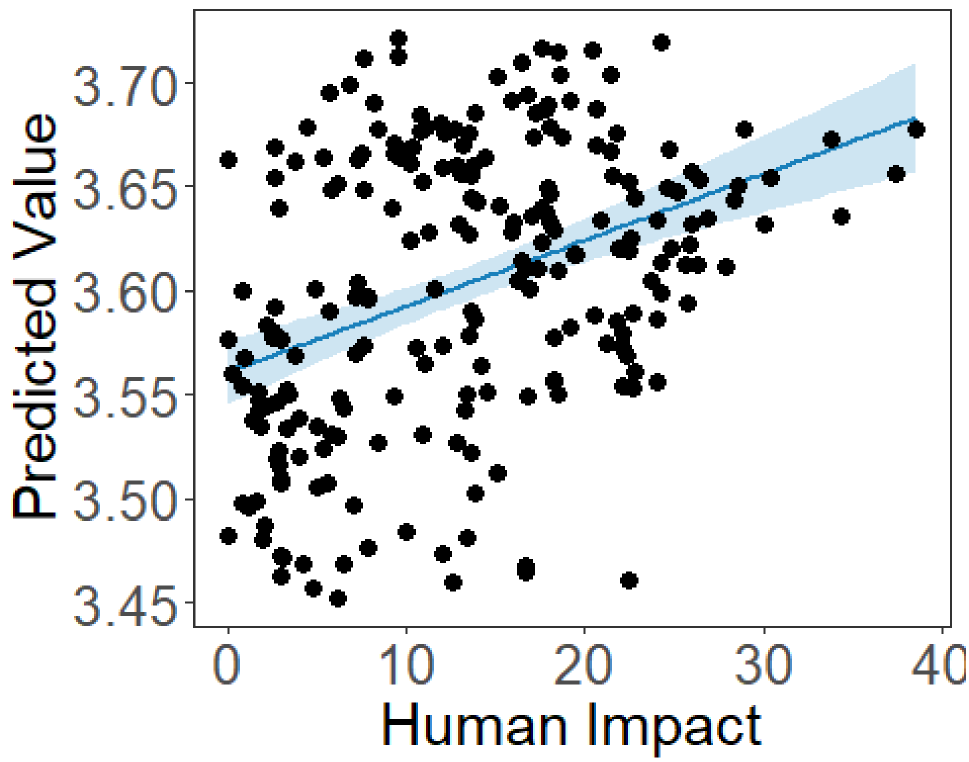


```
ggplot(Ecological_Data_Global_Hex_sp, aes(x = Nitrate_Mean, y = predict(m
eso.ES50.nitrate, Ecological_Data_Global_Hex_sp))) +
  geom_smooth(method = "gam", formula = y ~ x, color = "#1a80bb", fill =
"#85bede") +  # Add a smooth dark blue line with light blue shadow
  geom_point(size = 3) +  # Add scatter plot points
  theme_bw() +  # Use the black and white theme
  labs(
    x = "Nitrate (mmol . m-3)",  # Shorten the x-axis title
    y = "Predicted Value"  # Shorten the y-axis title
  ) +
  theme(
    panel.grid.minor = element_blank(),
    panel.grid.major = element_blank(),
    axis.text.x = element_text(size =20),  # Increase x-axis text size
    axis.text.y = element_text(size = 20),  # Increase y-axis text size
    axis.title.x = element_text(size = 22), # Increase x-axis title size
    axis.title.y = element_text(size = 22)  # Increase y-axis title size
  )
```

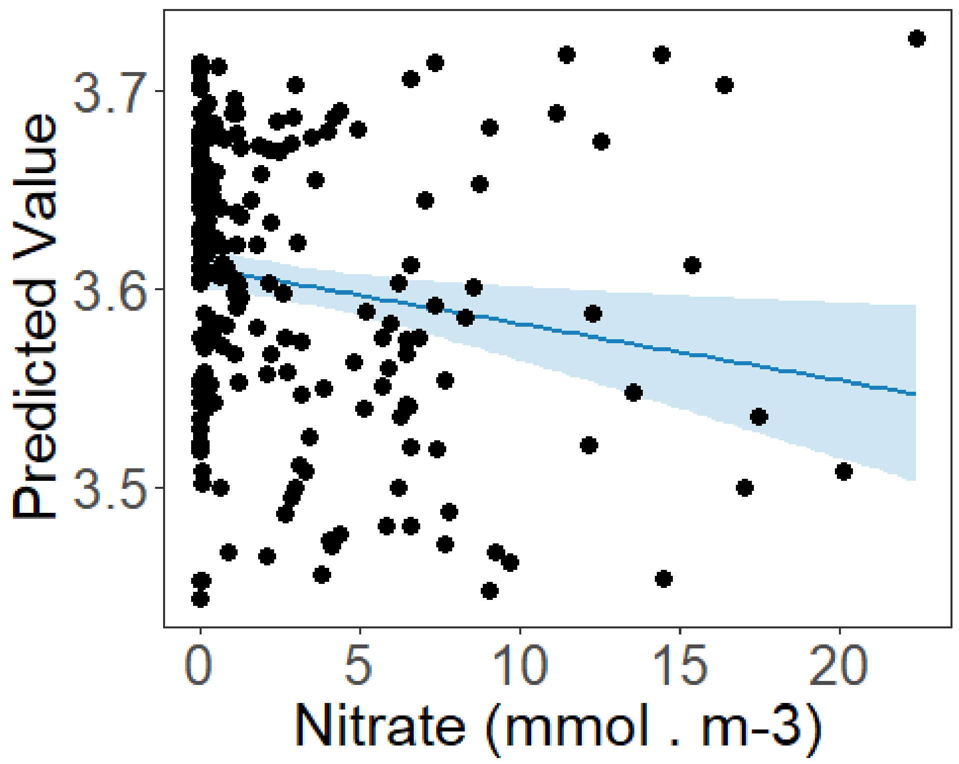


```
ggplot(Ecological_Data_Global_Hex_sp, aes(x = PrimProd_Mean, y = predict(
meso.ES50.PrimProd, Ecological_Data_Global_Hex_sp))) +
  geom_smooth(method = "gam", formula = y ~ x, color = "#1a80bb", fill =
"#85bede") +  # Add a smooth dark blue line with light blue shadow
  geom_point(size = 3) +  # Add scatter plot points
  theme_bw() +  # Use the black and white theme
  labs(
    x = "Primary Productivity (mmol . m-3)",  # Shorten the x-axis title
    y = "Predicted Value"  # Shorten the y-axis title
  ) +
  theme(
    panel.grid.minor = element_blank(),
    panel.grid.major = element_blank(),
    axis.text.x = element_text(size =20),  # Increase x-axis text size
    axis.text.y = element_text(size = 20),  # Increase y-axis text size
    axis.title.x = element_text(size = 22), # Increase x-axis title size
    axis.title.y = element_text(size = 22)  # Increase y-axis title size
  )
```

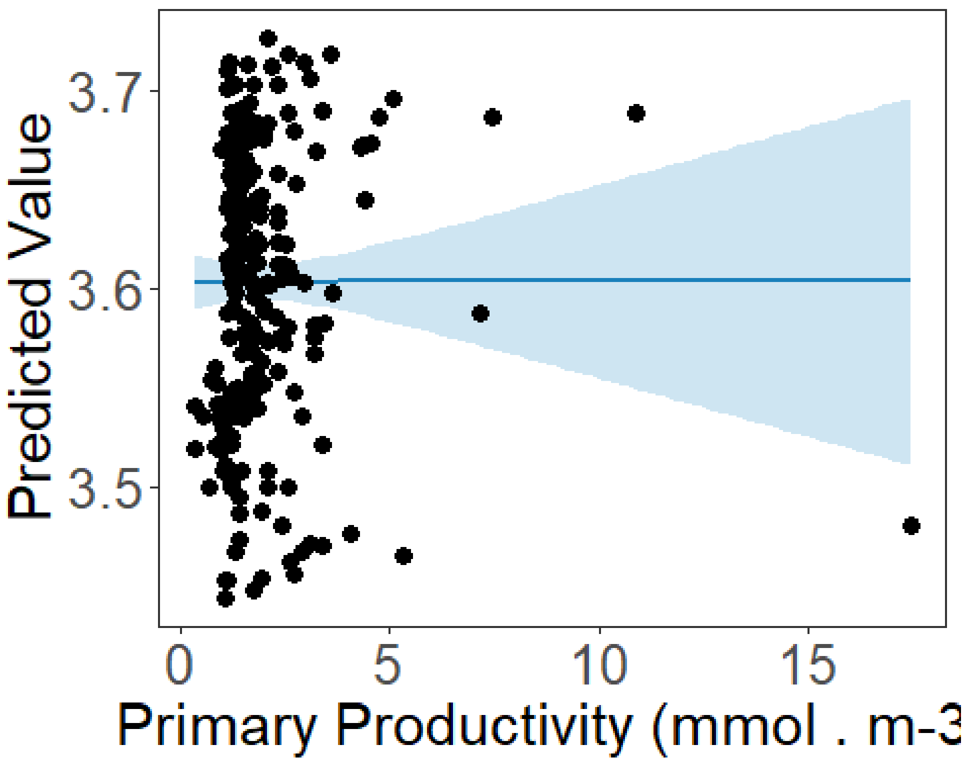


```
ggplot(Ecological_Data_Global_Hex_sp, aes(x = ThemM_mean, y = predict(meso.ES50.ThemM, Ecological_Data_Global_Hex_sp))) +
  geom_smooth(method = "gam", formula = y ~ x, color = "#1a80bb", fill = "#85bede") +  # Add a smooth dark blue line with light blue shadow
  geom_point(size = 3) +  # Add scatter plot points
  theme_bw() +  # Use the black and white theme
  labs(
    x = "Temperature Mean (ºC)",  # Shorten the x-axis title
    y = "Predicted Value"  # Shorten the y-axis title
  ) +
  theme(
    panel.grid.minor = element_blank(),
    panel.grid.major = element_blank(),
    axis.text.x = element_text(size =20),  # Increase x-axis text size
    axis.text.y = element_text(size = 20),  # Increase y-axis text size
    axis.title.x = element_text(size = 22), # Increase x-axis title size
    axis.title.y = element_text(size = 22)  # Increase y-axis title size
  )
```

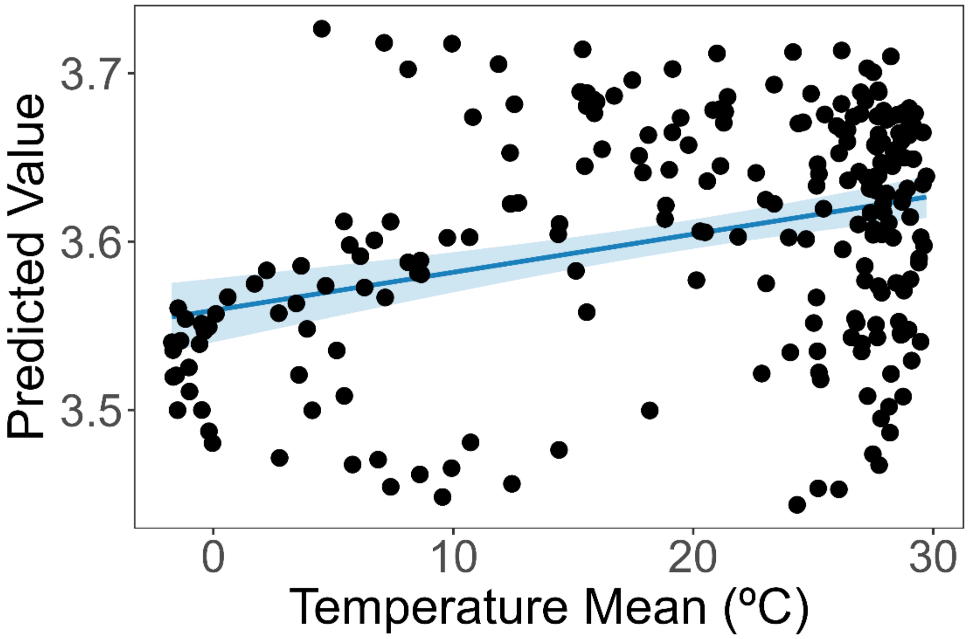


```
ggplot(Ecological_Data_Global_Hex_sp, aes(x = ThemR_mean, y = predict(mes
o.ES50.ThemR, Ecological_Data_Global_Hex_sp))) +
  geom_smooth(method = "gam", formula = y ~ x, color = "#1a80bb", fill =
"#85bede") +  # Add a smooth dark blue line with light blue shadow
  geom_point(size = 3) +  # Add scatter plot points
  theme_bw() +  # Use the black and white theme
  labs(
    x = "Temperature Range (ºC)",  # Shorten the x-axis title
    y = "Predicted Value"  # Shorten the y-axis title
  ) +
  theme(
    panel.grid.minor = element_blank(),
    panel.grid.major = element_blank(),
    axis.text.x = element_text(size =20),  # Increase x-axis text size
    axis.text.y = element_text(size = 20),  # Increase y-axis text size
    axis.title.x = element_text(size = 22), # Increase x-axis title size
    axis.title.y = element_text(size = 22)  # Increase y-axis title size
  )
```

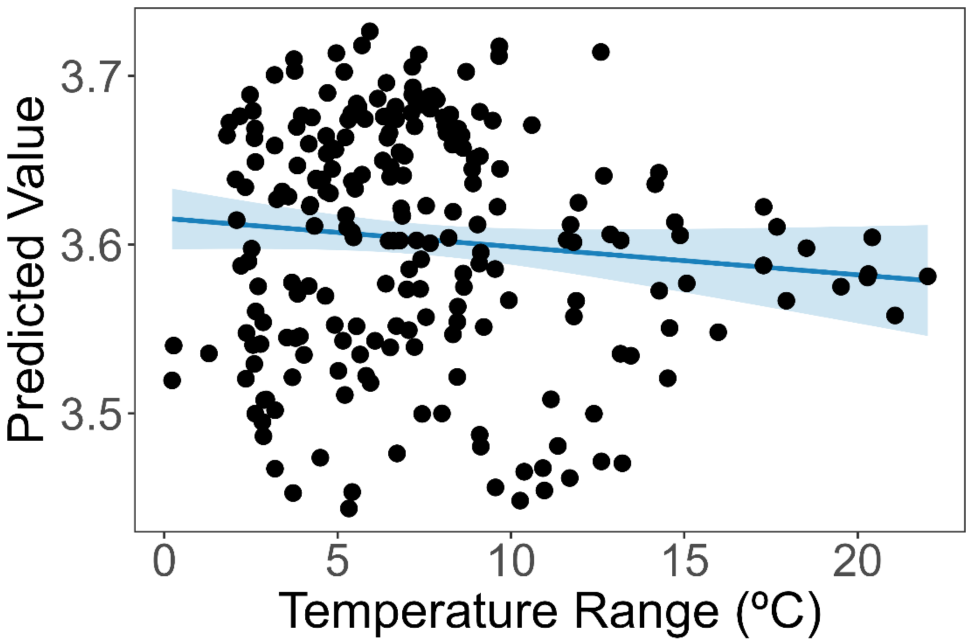

Predicted Value
3.7
3.6
3.5
0
5
10
15
20
Temperature Range (ºC)

Markdown_GAM_Deep.R

hsaeedi

2025-06-08

```r
# ====================================
#                 DEEP-NUMSPe-GAM
# ====================================
library(readxl)
```

```
## Warning: package 'readxl' was built under R version 4.3.3
```

```r
library(openxlsx)
```

```
## Warning: package 'openxlsx' was built under R version 4.3.3
```

```r
library(tidyverse)
```

```
## Warning: package 'tidyverse' was built under R version 4.3.3
```

```
## Warning: package 'ggplot2' was built under R version 4.3.3
```

```
## Warning: package 'tibble' was built under R version 4.3.3
```

```
## Warning: package 'tidyr' was built under R version 4.3.3
```

```
## Warning: package 'readr' was built under R version 4.3.3
```

```
## Warning: package 'purrr' was built under R version 4.3.3
```

```
## Warning: package 'dplyr' was built under R version 4.3.3
```

```
## Warning: package 'stringr' was built under R version 4.3.3
```

```
## Warning: package 'forcats' was built under R version 4.3.3
```

```
## Warning: package 'lubridate' was built under R version 4.3.3
```

```
## ── Attaching core tidyverse packages ──────────────────────── tidyvers
e 2.0.0 ──
## ✔ dplyr     1.1.4     ✔ readr     2.1.5
## ✔ forcats   1.0.0     ✔ stringr   1.5.1
## ✔ ggplot2   3.5.1     ✔ tibble    3.2.1
## ✔ lubridate 1.9.3     ✔ tidyr     1.3.1
## ✔ purrr     1.0.2
## ── Conflicts ────────────────────────────────────────── tidyverse_conf
licts() ──
## ✖ dplyr::filter() masks stats::filter()
## ✖ dplyr::lag()    masks stats::lag()
## ℹ Use the conflicted package (<http://conflicted.r-lib.org/>) to for
ce all conflicts to become errors
```

```r
library(sf)
```

```
## Warning: package 'sf' was built under R version 4.3.3

## Linking to GEOS 3.11.2, GDAL 3.8.2, PROJ 9.3.1; sf_use_s2() is TRUE

library(vegan)

## Warning: package 'vegan' was built under R version 4.3.3

## Loading required package: permute

## Warning: package 'permute' was built under R version 4.3.3

## Loading required package: lattice
## This is vegan 2.6-4

library(pvclust)

## Warning: package 'pvclust' was built under R version 4.3.3

library(dplyr)
library(ggplot2)
library(robis)

## Warning: package 'robis' was built under R version 4.3.3

library(obistools)
library(nortest) # for Anderson-Darling test
library(stringi) # for encoding UTF-8

## Warning: package 'stringi' was built under R version 4.3.3

library(corrplot)

## Warning: package 'corrplot' was built under R version 4.3.3

## corrplot 0.94 loaded

library(mgcv)

## Loading required package: nlme
##
## Attaching package: 'nlme'
##
## The following object is masked from 'package:dplyr':
##
##     collapse
##
## This is mgcv 1.9-0. For overview type 'help("mgcv-package")'.

library(ggeffects)

## Warning: package 'ggeffects' was built under R version 4.3.3

library(DHARMa) #simulations package gam-

## Warning: package 'DHARMa' was built under R version 4.3.3
```

```
## Warning in check_dep_version(): ABI version mismatch:
## lme4 was built with Matrix ABI version 1
## Current Matrix ABI version is 0
## Please re-install lme4 from source or restore original 'Matrix' packag
e

## This is DHARMa 0.4.6. For overview type '?DHARMa'. For recent changes,
type news(package = 'DHARMa')

library(knitr)

## Warning: package 'knitr' was built under R version 4.3.3

library(qpcR)

## Warning: package 'qpcR' was built under R version 4.3.3

## Loading required package: MASS
##
## Attaching package: 'MASS'
##
## The following object is masked from 'package:robis':
##
##     area
##
## The following object is masked from 'package:dplyr':
##
##     select
##
## Loading required package: minpack.lm

## Warning: package 'minpack.lm' was built under R version 4.3.3

## Loading required package: rgl

## Warning: package 'rgl' was built under R version 4.3.3

## Loading required package: robustbase

## Warning: package 'robustbase' was built under R version 4.3.3

## Loading required package: Matrix
##
## Attaching package: 'Matrix'
##
## The following objects are masked from 'package:tidyr':
##
##     expand, pack, unpack

#Species Counts and Environment, hexagons
Ecological_Data_Global_Hex_sp <- read.csv("Ecological_Data_Global_Hex_sp.
csv", sep = ";")

summary(Ecological_Data_Global_Hex_sp)
```

```
##        X               ID           Latitude        Longitude
##  Min.   :  1.0   Min.   :  1.0   Min.   :-87.63   Min.   :-174.45982
##  1st Qu.:161.2   1st Qu.:161.2   1st Qu.:-29.98   1st Qu.: -86.99765
##  Median :321.5   Median :321.5   Median :  0.00   Median :   0.21022
##  Mean   :321.5   Mean   :321.5   Mean   :  0.00   Mean   :  -0.08773
##  3rd Qu.:481.8   3rd Qu.:481.8   3rd Qu.: 29.98   3rd Qu.:  87.29992
##  Max.   :642.0   Max.   :642.0   Max.   : 87.63   Max.   : 174.30184
##
##     Area_Sum        Depth_Mean        Depth_Median        Depth_Max
##  Min.   :661589   Min.   :-5660.6   Min.   :-5842.0   Min.   :-4502.0
##  1st Qu.:794383   1st Qu.:-4455.8   1st Qu.:-4680.0   1st Qu.: -672.0
##  Median :796851   Median :-3642.6   Median :-3818.0   Median :   42.0
##  Mean   :794623   Mean   :-3172.3   Mean   :-3273.1   Mean   : -267.8
##  3rd Qu.:799061   3rd Qu.:-2122.7   3rd Qu.:-2158.0   3rd Qu.:  383.0
##  Max.   :803949   Max.   :   35.5   Max.   :   14.5   Max.   : 2876.0
##                   NA's   :81        NA's   :81        NA's   :81
##     Chl_Mean         Chl_Median        Margin_Sum          Margin_Mean
##  Min.   :0.04837   Min.   :0.04746   Min.   :-195640765   Min.   :-288
3.58
##  1st Qu.:0.11386   1st Qu.:0.10239   1st Qu.:  -7955299   1st Qu.: -93
0.65
##  Median :0.22296   Median :0.19813   Median :  -2449082   Median : -42
8.88
##  Mean   :0.29174   Mean   :0.26079   Mean   :  -6386344   Mean   : -59
9.64
##  3rd Qu.:0.35835   3rd Qu.:0.33879   3rd Qu.:    -87662   3rd Qu.:  -6
2.06
##  Max.   :4.71631   Max.   :4.71268   Max.   :         0   Max.   :
0.00
##  NA's   :82        NA's   :82                             NA's   :81
##  Margin_Median       Shelf_Sum         Shelf_Mean        Shelf_Median
##  Min.   :-3148.0   Min.   :-2566468   Min.   :-55.6837   Min.   :-56.0
00
##  1st Qu.:    0.0   1st Qu.:  -66625   1st Qu.: -5.4761   1st Qu.:  0.0
00
##  Median :    0.0   Median :    -545   Median : -0.1858   Median :  0.0
00
##  Mean   : -325.7   Mean   : -104973   Mean   : -5.5298   Mean   : -2.2
34
##  3rd Qu.:    0.0   3rd Qu.:       0   3rd Qu.:  0.0000   3rd Qu.:  0.0
00
##  Max.   :    0.0   Max.   :       0   Max.   :  0.0000   Max.   :  0.0
00
##  NA's   :81                           NA's   :81         NA's   :81
##   CurVel_Mean      CurVel_Median     CurVel_Bot_Mean   CurVel_Bot_Medi
an
##  Min.   :0.00712   Min.   :0.00498   Min.   :0.00088   Min.   :0.00049
##  1st Qu.:0.04354   1st Qu.:0.03687   1st Qu.:0.00957   1st Qu.:0.00647
##  Median :0.06934   Median :0.05959   Median :0.01521   Median :0.01016
##  Mean   :0.09485   Mean   :0.08466   Mean   :0.01963   Mean   :0.01365
##  3rd Qu.:0.12765   3rd Qu.:0.11084   3rd Qu.:0.02423   3rd Qu.:0.01650
```

```
##  Max.   :0.51113   Max.   :0.46702   Max.   :0.16873   Max.   :0.16081
##  NA's   :82        NA's   :82        NA's   :82        NA's   :82
##  Diff_Atte_Mean    Diff_Atte_Median    HumImp_Sum         HumImp_Mean
##  Min.   :0.02839   Min.   :0.02586   Min.   :       0   Min.   : 0.000
##  1st Qu.:0.05826   1st Qu.:0.05759   1st Qu.:       0   1st Qu.: 5.361
##  Median :0.06980   Median :0.06815   Median :    5028   Median :12.000
##  Mean   :0.08042   Mean   :0.07798   Mean   : 3518207   Mean   :12.467
##  3rd Qu.:0.08694   3rd Qu.:0.08342   3rd Qu.: 4649304   3rd Qu.:18.055
##  Max.   :0.39725   Max.   :0.43915   Max.   :37425783   Max.   :38.554
##  NA's   :82        NA's   :82        NA's   :19         NA's   :297
##  HumImp_Median   Ice_Cover_Sum     IceCov_Mean      Ice_Cover_Median
##  Min.   : 0.00   Min.   :     0   Min.   :0.00000   Min.   :0.00000
##  1st Qu.: 4.00   1st Qu.:     0   1st Qu.:0.00000   1st Qu.:0.00000
##  Median :11.00   Median :     0   Median :0.00000   Median :0.00000
##  Mean   :11.43   Mean   :  4551   Mean   :0.07398   Mean   :0.07278
##  3rd Qu.:18.00   3rd Qu.:     0   3rd Qu.:0.00000   3rd Qu.:0.00000
##  Max.   :42.00   Max.   :603554   Max.   :0.96853   Max.   :0.96977
##  NA's   :297                      NA's   :82        NA's   :82
##  Ice_Tick_Mean    Ice_Tick_Median   MixLay_Mean      MixLay_Median
##  Min.   :0.0000   Min.   :0.0000   Min.   :  7.592   Min.   :  7.019
##  1st Qu.:0.0000   1st Qu.:0.0000   1st Qu.: 21.977   1st Qu.: 22.198
##  Median :0.0000   Median :0.0000   Median : 34.135   Median : 34.273
##  Mean   :0.1148   Mean   :0.1115   Mean   : 39.535   Mean   : 39.294
##  3rd Qu.:0.0000   3rd Qu.:0.0000   3rd Qu.: 47.630   3rd Qu.: 46.981
##  Max.   :3.1516   Max.   :3.5327   Max.   :177.253   Max.   :189.459
##  NA's   :82       NA's   :82       NA's   :82        NA's   :82
##   Nitrate_Mean      Nitrate_Median     Nitrate_Bot_Mean   Nitrate_Bot_
Median
##  Min.   : 0.00054   Min.   : 0.00045   Min.   : 0.04162   Min.   : 0.0
0
##  1st Qu.: 0.04998   1st Qu.: 0.01156   1st Qu.:22.14293   1st Qu.:22.6
4
##  Median : 1.14055   Median : 0.84186   Median :32.52189   Median :32.9
5
##  Mean   : 5.61826   Mean   : 5.53985   Mean   :27.47784   Mean   :28.0
7
##  3rd Qu.: 6.45838   3rd Qu.: 6.46006   3rd Qu.:34.88484   3rd Qu.:35.5
1
##  Max.   :33.91337   Max.   :31.22474   Max.   :39.54847   Max.   :40.1
0
##  NA's   :82         NA's   :82         NA's   :82         NA's   :82
##     O2_Mean        O2_Median       O2_Bot_Mean      O2_Bot_Median
##  Min.   :195.1   Min.   :195.2   Min.   :  3.051   Min.   :  0.4178
##  1st Qu.:206.2   1st Qu.:206.1   1st Qu.:164.353   1st Qu.:164.9396
##  Median :226.9   Median :226.0   Median :199.301   Median :202.8894
##  Mean   :250.8   Mean   :250.4   Mean   :200.601   Mean   :201.8704
##  3rd Qu.:294.9   3rd Qu.:292.7   3rd Qu.:234.898   3rd Qu.:237.5830
##  Max.   :390.2   Max.   :392.5   Max.   :389.954   Max.   :390.7931
##  NA's   :82      NA's   :82      NA's   :82        NA's   :82
##  PhotoActi_Mean   PhotoActi_Median PrimProd_Bot_Mean PrimProd_Bot_Medi
an
```

```
##  Min.   : 3.864   Min.   : 3.642   Min.   :0.01355   Min.   :0.01355  
##  1st Qu.:27.254   1st Qu.:27.229   1st Qu.:0.01355   1st Qu.:0.01355  
##  Median :39.860   Median :39.993   Median :0.01707   Median :0.01355  
##  Mean   :36.778   Mean   :36.811   Mean   :0.28762   Mean   :0.18279  
##  3rd Qu.:45.157   3rd Qu.:45.163   3rd Qu.:0.12868   3rd Qu.:0.01447  
##  Max.   :51.482   Max.   :52.344   Max.   :9.24785   Max.   :8.02454  
##  NA's   :82       NA's   :82       NA's   :82       NA's   :82       
##  PrimProd_Mean     PrimProd_median   Salinity_Bot_mean Salinity_Bot_me
dian
##  Min.   : 0.3348   Min.   : 0.3118   Min.   : 4.727    Min.   : 4.103  
##  1st Qu.: 1.2183   1st Qu.: 1.2000   1st Qu.:34.661    1st Qu.:34.680  
##  Median : 1.4959   Median : 1.4421   Median :34.696    Median :34.703  
##  Mean   : 1.7349   Mean   : 1.6125   Mean   :34.304    Mean   :34.400  
##  3rd Qu.: 1.7421   3rd Qu.: 1.6362   3rd Qu.:34.760    3rd Qu.:34.761  
##  Max.   :17.4345   Max.   :17.4302   Max.   :40.231    Max.   :40.600  
##  NA's   :82        NA's   :82        NA's   :82        NA's   :82      
##  Salinity_mean    Salinity_median  Silicate_mean      Silicate_median  
##  Min.   : 3.239   Min.   : 2.786   Min.   :  0.7463   Min.   :  0.7133
##  1st Qu.:33.879   1st Qu.:33.888   1st Qu.:  1.6572   1st Qu.:  1.5791
##  Median :34.534   Median :34.566   Median :  2.6372   Median :  2.4321
##  Mean   :33.996   Mean   :34.123   Mean   : 11.1758   Mean   : 10.8693
##  3rd Qu.:35.403   3rd Qu.:35.450   3rd Qu.: 10.9034   3rd Qu.:  8.9543
##  Max.   :38.992   Max.   :39.180   Max.   :116.4004   Max.   :118.7041
##  NA's   :82       NA's   :82       NA's   :82         NA's   :82      
##    ThemM_mean      ThemM_median     ThemM_Bot_mean    ThemM_Bot_median 
##  Min.   :-1.794   Min.   :-1.808   Min.   :-1.7009   Min.   :-1.7534  
##  1st Qu.: 9.333   1st Qu.: 9.049   1st Qu.: 0.8558   1st Qu.: 0.7385  
##  Median :21.327   Median :21.407   Median : 1.3503   Median : 1.1530  
##  Mean   :17.837   Mean   :17.863   Mean   : 3.3903   Mean   : 2.8933  
##  3rd Qu.:26.986   3rd Qu.:27.043   3rd Qu.: 3.1400   3rd Qu.: 1.9769  
##  Max.   :29.712   Max.   :29.737   Max.   :28.4978   Max.   :28.7753  
##  NA's   :82       NA's   :82       NA's   :82        NA's   :82       
##  Them_Max_mean    Them_Max_median   Them_Max_max      Them_Min_mean   
##  Min.   :-1.555   Min.   :-1.594   Min.   :-0.9619   Min.   :-1.981  
##  1st Qu.:14.674   1st Qu.:14.537   1st Qu.:19.2728   1st Qu.: 5.467  
##  Median :25.932   Median :25.868   Median :27.9112   Median :16.985  
##  Mean   :21.550   Mean   :21.548   Mean   :23.8339   Mean   :14.700  
##  3rd Qu.:29.399   3rd Qu.:29.409   3rd Qu.:30.3046   3rd Qu.:24.027  
##  Max.   :34.692   Max.   :34.669   Max.   :36.4851   Max.   :28.633  
##  NA's   :82       NA's   :82       NA's   :82        NA's   :82      
##  Them_Min_median   Them_Min_min       ThemR_mean       ThemR_median    
##  Min.   :-2.000   Min.   :-2.0000   Min.   : 0.226   Min.   : 0.1985  
##  1st Qu.: 5.339   1st Qu.: 0.6541   1st Qu.: 4.119   1st Qu.: 4.0608  
##  Median :17.097   Median :13.5745   Median : 6.166   Median : 6.0265  
##  Mean   :14.740   Mean   :12.1140   Mean   : 6.851   Mean   : 6.7578  
##  3rd Qu.:24.112   3rd Qu.:21.4512   3rd Qu.: 8.341   3rd Qu.: 8.3701  
##  Max.   :28.836   Max.   :27.4934   Max.   :25.406   Max.   :25.4389  
##  NA's   :82       NA's   :82        NA's   :82       NA's   :82       
##   ThemR_range       ThemR_Bot_mean      ThemR_Bot_median   ThemR_Bot_ra
nge
##  Min.   : 0.09749   Min.   : 0.01941   Min.   : 0.01558   Min.   : 0.0
```

```
5681
##  1st Qu.: 2.75496   1st Qu.: 0.10560   1st Qu.: 0.07944   1st Qu.: 1.1
2864
##  Median : 4.03592   Median : 0.35621   Median : 0.17431   Median : 5.3
0821
##  Mean   : 4.82962   Mean   : 1.34412   Mean   : 1.01748   Mean   : 6.8
4583
##  3rd Qu.: 6.17135   3rd Qu.: 1.13137   3rd Qu.: 0.51880   3rd Qu.:11.0
6246
##  Max.   :20.18199   Max.   :22.02747   Max.   :21.85832   Max.   :28.6
5922
##  NA's   :82         NA's   :82         NA's   :82         NA's   :82
##    NumRec_sha        NumSpe_sha          ES50_sha       Chao1_sha
##  Min.   :      2   Min.   :    2.00   Min.   : 3.40   Min.   :    5.0
##  1st Qu.:    657   1st Qu.:   88.25   1st Qu.:25.33   1st Qu.:  156.0
##  Median :   4034   Median :  278.00   Median :34.11   Median :  472.5
##  Mean   :  67514   Mean   :  939.73   Mean   :33.11   Mean   : 1414.0
##  3rd Qu.:  21699   3rd Qu.:  998.00   3rd Qu.:42.24   3rd Qu.: 1648.5
##  Max.   :6258830   Max.   :11419.00   Max.   :52.46   Max.   :13882.3
##  NA's   :80        NA's   :80         NA's   :102     NA's   :102
##     ACE_sha           Weighted_sha       NumRec_mes          NumSpe_mes
##  Min.   :    6.111   Min.   :  1.669   Min.   :     1.0   Min.   :   1
.00
##  1st Qu.:  147.383   1st Qu.: 14.734   1st Qu.:    71.8   1st Qu.:  32
.75
##  Median :  465.805   Median : 33.018   Median :   388.0   Median : 108
.50
##  Mean   : 1453.239   Mean   : 66.817   Mean   :  7464.8   Mean   : 325
.99
##  3rd Qu.: 1716.280   3rd Qu.: 68.502   3rd Qu.:  1907.0   3rd Qu.: 371
.75
##  Max.   :16530.276   Max.   :774.467   Max.   :809096.0   Max.   :3809
.00
##  NA's   :104         NA's   :102       NA's   :110        NA's   :110
##     ES50_mes       Chao1_mes         ACE_mes        Weighted_mes
##  Min.   : 8.561   Min.   :   9.0   Min.   :   9.0   Min.   :  3.076
##  1st Qu.:29.228   1st Qu.: 110.8   1st Qu.: 114.3   1st Qu.: 23.652
##  Median :36.387   Median : 302.3   Median : 305.9   Median : 48.663
##  Mean   :35.088   Mean   : 641.1   Mean   : 638.6   Mean   : 80.441
##  3rd Qu.:43.333   3rd Qu.: 789.7   3rd Qu.: 788.8   3rd Qu.:106.010
##  Max.   :50.000   Max.   :9453.0   Max.   :5423.8   Max.   :533.774
##  NA's   :217      NA's   :217      NA's   :219      NA's   :217
##    NumRec_dee         NumSpe_dee         ES50_dee       Chao1_dee
##  Min.   :     1.0   Min.   :   1.0   Min.   : 1.049   Min.   :   2.0
##  1st Qu.:    85.5   1st Qu.:  51.5   1st Qu.:32.574   1st Qu.: 189.5
##  Median :   509.0   Median : 161.0   Median :38.851   Median : 393.9
##  Mean   :  5125.1   Mean   : 382.0   Mean   :37.584   Mean   : 712.7
##  3rd Qu.:  2261.5   3rd Qu.: 435.0   3rd Qu.:44.250   3rd Qu.: 895.7
##  Max.   :512758.0   Max.   :5204.0   Max.   :50.000   Max.   :6899.6
##  NA's   :111        NA's   :111      NA's   :200      NA's   :200
##     ACE_dee          Weighted_dee
```

```
##  Min.   :  27.62   Min.   :  1.002  
##  1st Qu.: 210.68   1st Qu.: 29.434  
##  Median : 431.06   Median : 57.897  
##  Mean   : 742.80   Mean   : 92.117  
##  3rd Qu.: 931.61   3rd Qu.:123.713  
##  Max.   :7151.71   Max.   :544.308  
##  NA's   :203       NA's   :200

#Correlation analyses for variables
#select only numeric values
numeric_data <- Ecological_Data_Global_Hex_sp %>% select_if(is.numeric)

#select for all columns
correlation_matrix <- cor(numeric_data, use = "complete.obs", method = "s
pearman")

#First we're going to load in our data and then trim the data frame down
to just the columns we need.
analysis.cols <- c("Latitude", "Longitude", "Depth_Mean", "Margin_Sum", "
CurVel_Bot_Mean", "HumImp_Mean",
                   "Nitrate_Bot_Mean", "O2_Bot_Mean", "PrimProd_Bot_Mean"
, "ThemM_Bot_mean", "NumRec_dee",
                   "NumSpe_dee", "ES50_dee")
Ecological_Data_Global_Hex_sp <- Ecological_Data_Global_Hex_sp [,analysis
.cols]
Ecological_Data_Global_Hex_sp  <- Ecological_Data_Global_Hex_sp [complete
.cases(Ecological_Data_Global_Hex_sp),]

# Calculate the correlation matrix
corr_matrix <- cor(Ecological_Data_Global_Hex_sp)

# Create the correlation plot with black font for text
corrplot(corr_matrix, tl.col = "black")
```

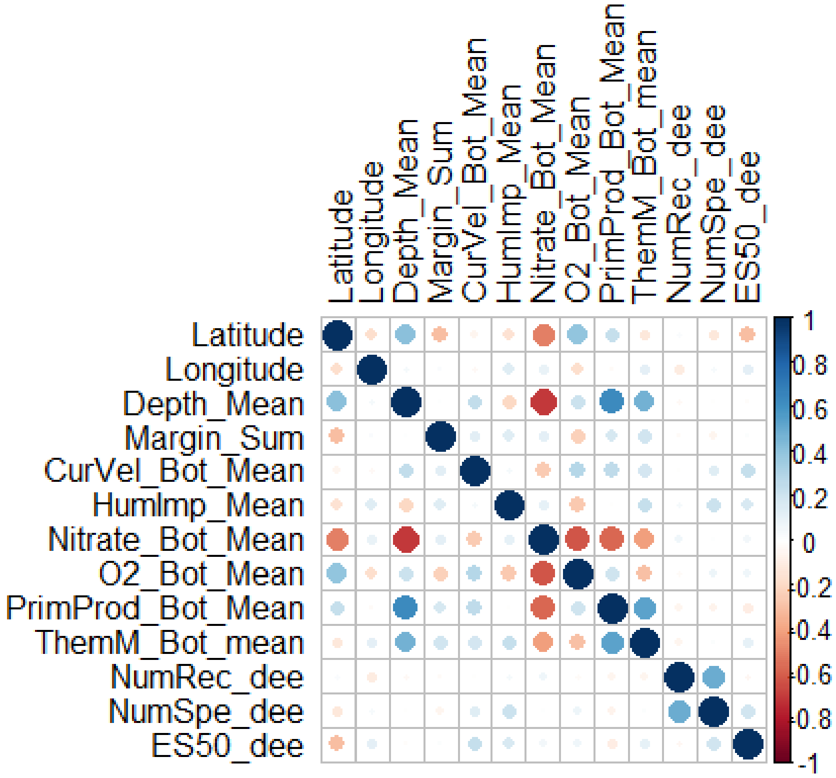


```r
#GAMs for number of species, deep
deep.numsp.intercept <- gam(NumSpe_dee ~ 1, data = Ecological_Data_Global
_Hex_sp, family = "nb", method = "REML", select = TRUE)
gam.check(deep.numsp.intercept)
```

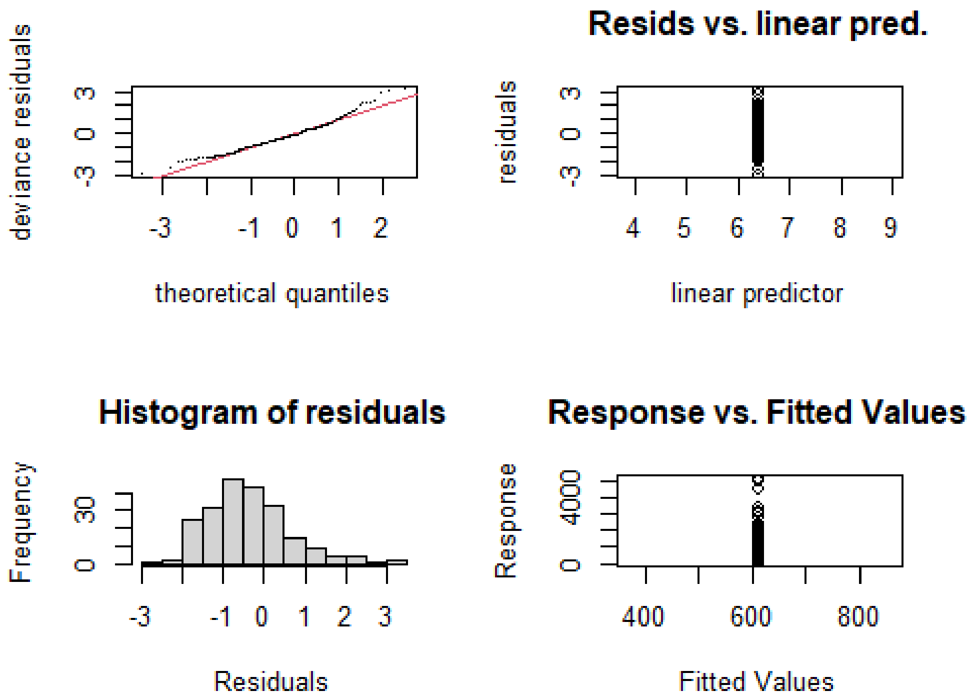


```
##
## Method: REML   Optimizer: outer newton
## full convergence after 2 iterations.
## Gradient range [0.0002950784,0.0002950784]
```

```
## (score 1611.525 & scale 1).
## Hessian positive definite, eigenvalue range [140.2144,140.2144].
## Model rank =  1 / 1

summary(deep.numsp.intercept)

##
## Family: Negative Binomial(0.927)
## Link function: log
##
## Formula:
## NumSpe_dee ~ 1
##
## Parametric coefficients:
##             Estimate Std. Error z value Pr(>|z|)
## (Intercept)  6.41931    0.07056   90.97   <2e-16 ***
## ---
## Signif. codes:  0 '***' 0.001 '**' 0.01 '*' 0.05 '.' 0.1 ' ' 1
##
##
## R-sq.(adj) =      0   Deviance explained = 2.32e-08%
## -REML = 1611.5  Scale est. = 1         n = 217

deep.numsp.latlon <- gam(NumSpe_dee ~ s(Latitude, Longitude, bs = "sos")
+ s(NumRec_dee), data = Ecological_Data_Global_Hex_sp, family = "nb", met
hod = "REML", select = TRUE)
gam.check(deep.numsp.latlon)
```

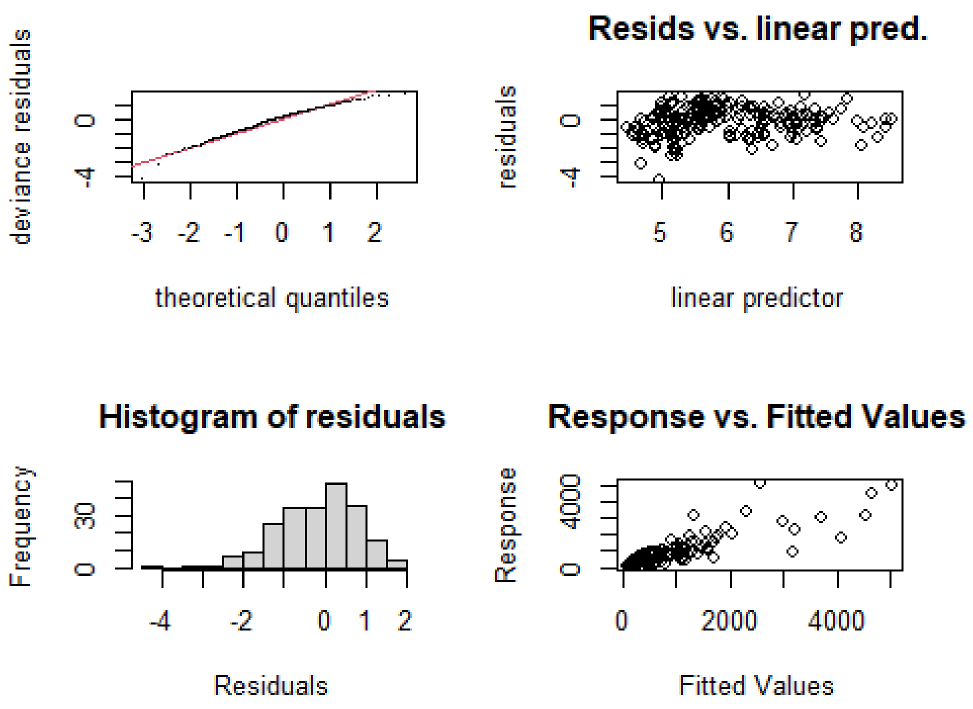


```
##
## Method: REML   Optimizer: outer newton
## full convergence after 10 iterations.
```

```
## Gradient range [-2.211786e-11,6.067618e-09]
## (score 1497.718 & scale 1).
## Hessian positive definite, eigenvalue range [0.4866557,96.77615].
## Model rank =  59 / 59
##
## Basis dimension (k) checking results. Low p-value (k-index<1) may
## indicate that k is too low, especially if edf is close to k'.
##
##                             k'   edf k-index p-value
## s(Latitude,Longitude) 49.00 14.81    0.82  <2e-16 ***
## s(NumRec_dee)          9.00  8.56    0.55  <2e-16 ***
## ---
## Signif. codes:  0 '***' 0.001 '**' 0.01 '*' 0.05 '.' 0.1 ' ' 1
```

```
summary(deep.numsp.latlon)
```

```
##
## Family: Negative Binomial(3.123)
## Link function: log
##
## Formula:
## NumSpe_dee ~ s(Latitude, Longitude, bs = "sos") + s(NumRec_dee)
##
## Parametric coefficients:
##             Estimate Std. Error z value Pr(>|z|)
## (Intercept)  5.95012    0.03863     154   <2e-16 ***
## ---
## Signif. codes:  0 '***' 0.001 '**' 0.01 '*' 0.05 '.' 0.1 ' ' 1
##
## Approximate significance of smooth terms:
##                          edf Ref.df Chi.sq p-value
## s(Latitude,Longitude) 14.810     49  75.01  <2e-16 ***
## s(NumRec_dee)          8.562      9 358.00  <2e-16 ***
## ---
## Signif. codes:  0 '***' 0.001 '**' 0.01 '*' 0.05 '.' 0.1 ' ' 1
##
## R-sq.(adj) =  0.707   Deviance explained = 75.4%
## -REML = 1497.7  Scale est. = 1         n = 217
```

```
deep.numsp.depth <- gam(NumSpe_dee ~ s(Latitude, Longitude, bs = "sos") +
s(NumRec_dee) + s(Depth_Mean), data = Ecological_Data_Global_Hex_sp, fami
ly = "nb", method = "REML", select = TRUE)
gam.check(deep.numsp.depth)
```

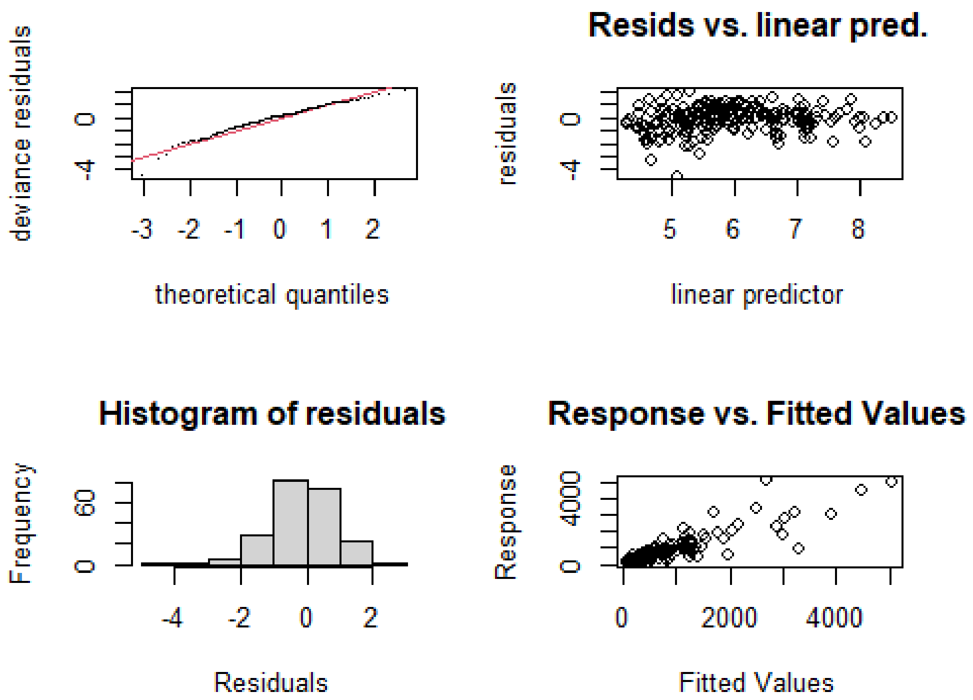


```
##
## Method: REML   Optimizer: outer newton
## full convergence after 9 iterations.
## Gradient range [-8.176507e-05,5.000045e-05]
## (score 1484.391 & scale 1).
## Hessian positive definite, eigenvalue range [8.175036e-05,95.12781].
## Model rank =  68 / 68
##
## Basis dimension (k) checking results. Low p-value (k-index<1) may
## indicate that k is too low, especially if edf is close to k'.
##
##                              k'    edf k-index p-value
## s(Latitude,Longitude) 49.00 12.39    0.84  <2e-16 ***
## s(NumRec_dee)          9.00  8.46    0.63  <2e-16 ***
## s(Depth_Mean)          9.00  3.00    1.12    0.99
## ---
## Signif. codes:  0 '***' 0.001 '**' 0.01 '*' 0.05 '.' 0.1 ' ' 1
```

```
summary(deep.numsp.depth)
```

```
##
## Family: Negative Binomial(3.527)
## Link function: log
##
## Formula:
## NumSpe_dee ~ s(Latitude, Longitude, bs = "sos") + s(NumRec_dee) +
##     s(Depth_Mean)
##
## Parametric coefficients:
##             Estimate Std. Error z value Pr(>|z|)
```

```
## (Intercept)  5.93216    0.03639     163   <2e-16 ***
## ---
## Signif. codes:  0 '***' 0.001 '**' 0.01 '*' 0.05 '.' 0.1 ' ' 1
##
## Approximate significance of smooth terms:
##                             edf Ref.df Chi.sq p-value
## s(Latitude,Longitude) 12.387     49  45.99  <2e-16 ***
## s(NumRec_dee)          8.463      9 313.79  <2e-16 ***
## s(Depth_Mean)          2.999      9  41.68  <2e-16 ***
## ---
## Signif. codes:  0 '***' 0.001 '**' 0.01 '*' 0.05 '.' 0.1 ' ' 1
##
## R-sq.(adj) =  0.758   Deviance explained = 78.3%
## -REML = 1484.4  Scale est. = 1         n = 217
```

```
deep.numsp.margin <- gam(NumSpe_dee ~ s(Latitude, Longitude, bs = "sos")
+ s(NumRec_dee) + s(Margin_Sum), data = Ecological_Data_Global_Hex_sp, fa
mily = "nb", method = "REML", select = TRUE)
gam.check(deep.numsp.margin)
```

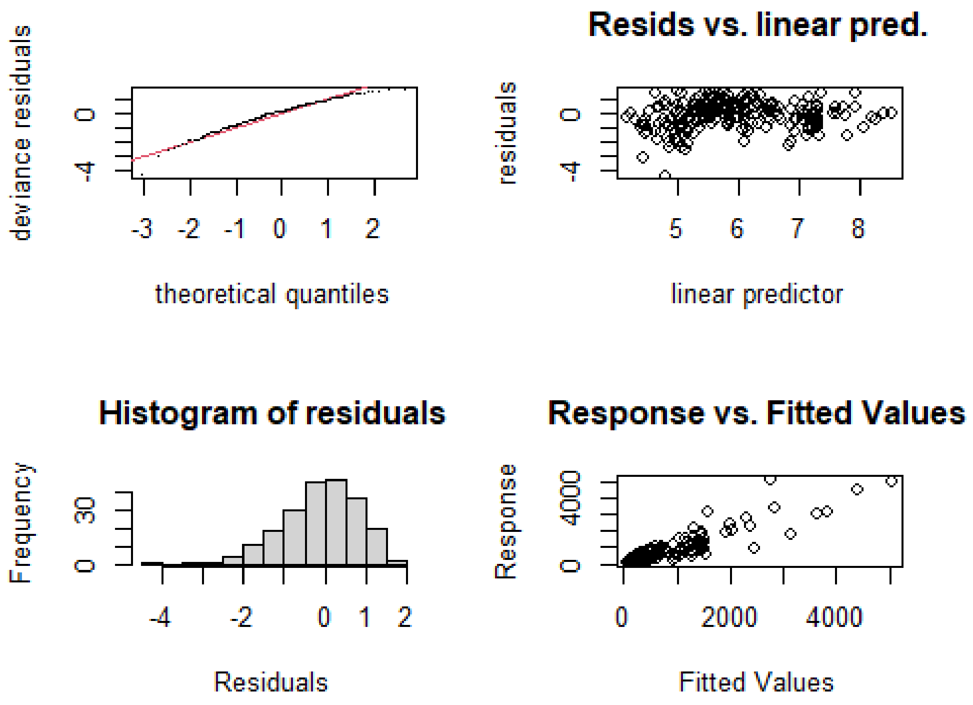


```
##
## Method: REML   Optimizer: outer newton
## full convergence after 10 iterations.
## Gradient range [-3.243863e-08,1.754679e-06]
## (score 1487.548 & scale 1).
## Hessian positive definite, eigenvalue range [0.2307296,91.02984].
## Model rank =  68 / 68
##
## Basis dimension (k) checking results. Low p-value (k-index<1) may
## indicate that k is too low, especially if edf is close to k'.
```

```
##
##                              k'    edf k-index p-value
## s(Latitude,Longitude) 49.00 16.72    0.85   0.005 **
## s(NumRec_dee)          9.00  8.55    0.58  <2e-16 ***
## s(Margin_Sum)          9.00  3.26    0.99   0.550
## ---
## Signif. codes:  0 '***' 0.001 '**' 0.01 '*' 0.05 '.' 0.1 ' ' 1
```

```
summary(deep.numsp.margin)
```

```
##
## Family: Negative Binomial(3.643)
## Link function: log
##
## Formula:
## NumSpe_dee ~ s(Latitude, Longitude, bs = "sos") + s(NumRec_dee) +
##     s(Margin_Sum)
##
## Parametric coefficients:
##             Estimate Std. Error z value Pr(>|z|)
## (Intercept)  5.92507    0.03582   165.4   <2e-16 ***
## ---
## Signif. codes:  0 '***' 0.001 '**' 0.01 '*' 0.05 '.' 0.1 ' ' 1
##
## Approximate significance of smooth terms:
##                          edf Ref.df Chi.sq p-value
## s(Latitude,Longitude) 16.722     49  99.41  <2e-16 ***
## s(NumRec_dee)          8.554      9 327.26  <2e-16 ***
## s(Margin_Sum)          3.262      9  32.31  <2e-16 ***
## ---
## Signif. codes:  0 '***' 0.001 '**' 0.01 '*' 0.05 '.' 0.1 ' ' 1
##
## R-sq.(adj) =   0.79   Deviance explained = 79.5%
## -REML = 1487.5  Scale est. = 1         n = 217
```

```
deep.numsp.current <- gam(NumSpe_dee ~ s(Latitude, Longitude, bs = "sos")
+ s(NumRec_dee) + s(CurVel_Bot_Mean), data = Ecological_Data_Global_Hex_s
p, family = "nb", method = "REML", select = TRUE)
gam.check(deep.numsp.current)
```

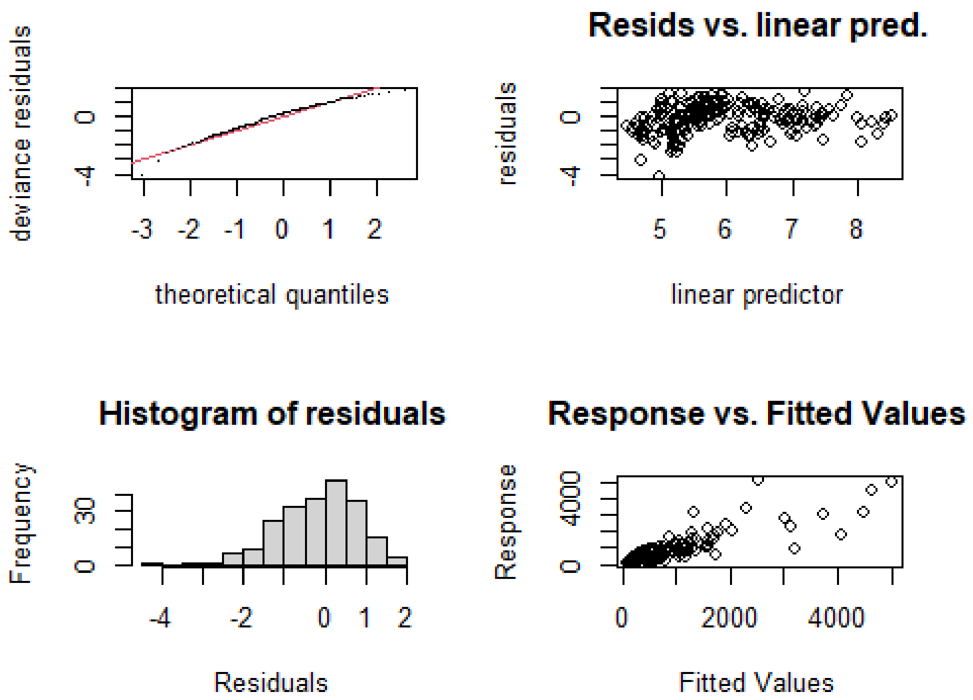


```
##
## Method: REML   Optimizer: outer newton
## full convergence after 14 iterations.
## Gradient range [-0.001549631,0.0007180756]
## (score 1497.671 & scale 1).
## Hessian positive definite, eigenvalue range [0.0007499878,96.89412].
## Model rank =  68 / 68
##
## Basis dimension (k) checking results. Low p-value (k-index<1) may
## indicate that k is too low, especially if edf is close to k'.
##
##                             k'    edf k-index p-value
## s(Latitude,Longitude) 49.000 14.455    0.82  <2e-16 ***
## s(NumRec_dee)          9.000  8.559    0.55  <2e-16 ***
## s(CurVel_Bot_Mean)     9.000  0.399    0.97    0.46
## ---
## Signif. codes:  0 '***' 0.001 '**' 0.01 '*' 0.05 '.' 0.1 ' ' 1
```

```
summary(deep.numsp.current)
```

```
##
## Family: Negative Binomial(3.12)
## Link function: log
##
## Formula:
## NumSpe_dee ~ s(Latitude, Longitude, bs = "sos") + s(NumRec_dee) +
##     s(CurVel_Bot_Mean)
##
## Parametric coefficients:
##             Estimate Std. Error z value Pr(>|z|)
```

```
## (Intercept)  5.95023    0.03865     154   <2e-16 ***
## ---
## Signif. codes:  0 '***' 0.001 '**' 0.01 '*' 0.05 '.' 0.1 ' ' 1
##
## Approximate significance of smooth terms:
##                             edf Ref.df  Chi.sq p-value
## s(Latitude,Longitude) 14.4547     49  70.903  <2e-16 ***
## s(NumRec_dee)          8.5594      9 360.173  <2e-16 ***
## s(CurVel_Bot_Mean)     0.3985      9   0.565   0.199
## ---
## Signif. codes:  0 '***' 0.001 '**' 0.01 '*' 0.05 '.' 0.1 ' ' 1
##
## R-sq.(adj) =  0.706   Deviance explained = 75.4%
## -REML = 1497.7  Scale est. = 1         n = 217
```

```
deep.numsp.humimp <- gam(NumSpe_dee ~ s(Latitude, Longitude, bs = "sos")
+ s(NumRec_dee) + s(HumImp_Mean), data = Ecological_Data_Global_Hex_sp, f
amily = "nb", method = "REML", select = TRUE)
gam.check(deep.numsp.humimp)
```

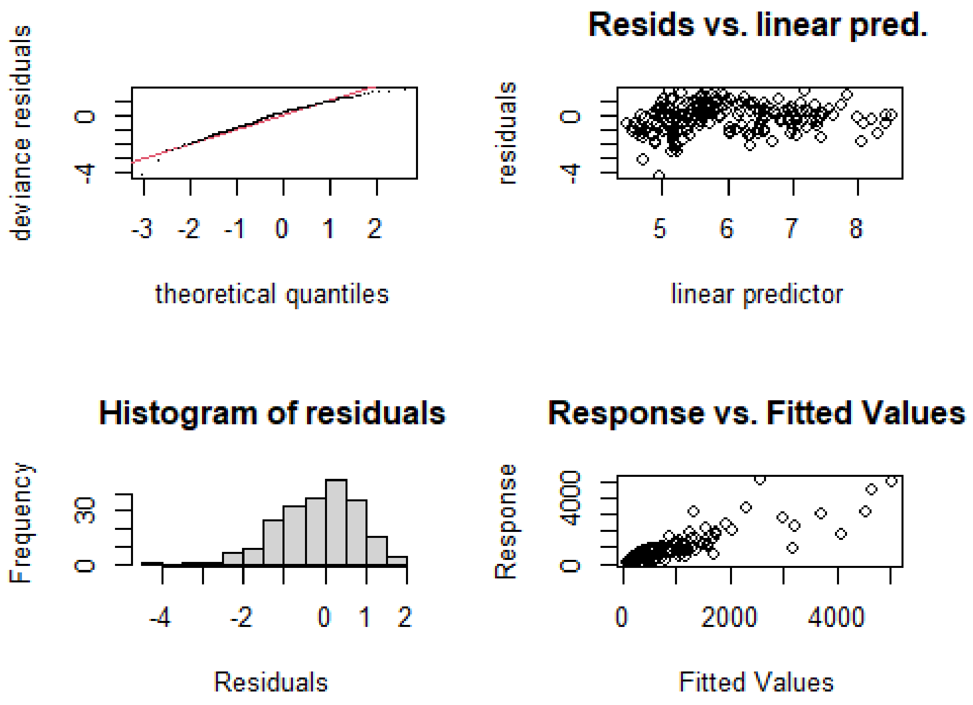


```
##
## Method: REML   Optimizer: outer newton
## full convergence after 10 iterations.
## Gradient range [-0.001588364,0.0003804437]
## (score 1497.715 & scale 1).
## Hessian positive definite, eigenvalue range [0.0004141268,96.83117].
## Model rank =  68 / 68
##
## Basis dimension (k) checking results. Low p-value (k-index<1) may
## indicate that k is too low, especially if edf is close to k'.
```

```
##
##                              k'    edf k-index p-value
## s(Latitude,Longitude) 49.000 14.658    0.82  <2e-16 ***
## s(NumRec_dee)          9.000  8.561    0.55  <2e-16 ***
## s(HumImp_Mean)         9.000  0.155    1.06    0.94
## ---
## Signif. codes:  0 '***' 0.001 '**' 0.01 '*' 0.05 '.' 0.1 ' ' 1
```

```
summary(deep.numsp.humimp)
```

```
##
## Family: Negative Binomial(3.121)
## Link function: log
##
## Formula:
## NumSpe_dee ~ s(Latitude, Longitude, bs = "sos") + s(NumRec_dee) +
##     s(HumImp_Mean)
##
## Parametric coefficients:
##             Estimate Std. Error z value Pr(>|z|)
## (Intercept)  5.95023    0.03864     154   <2e-16 ***
## ---
## Signif. codes:  0 '***' 0.001 '**' 0.01 '*' 0.05 '.' 0.1 ' ' 1
##
## Approximate significance of smooth terms:
##                           edf Ref.df  Chi.sq p-value
## s(Latitude,Longitude) 14.6583     49  71.961  <2e-16 ***
## s(NumRec_dee)          8.5607      9 354.457  <2e-16 ***
## s(HumImp_Mean)         0.1546      9   0.165   0.255
## ---
## Signif. codes:  0 '***' 0.001 '**' 0.01 '*' 0.05 '.' 0.1 ' ' 1
##
## R-sq.(adj) =  0.707   Deviance explained = 75.4%
## -REML = 1497.7  Scale est. = 1         n = 217
```

```
deep.numsp.nitrate <- gam(NumSpe_dee ~ s(Latitude, Longitude, bs = "sos")
+ s(NumRec_dee) + s(Nitrate_Bot_Mean), data = Ecological_Data_Global_Hex_
sp, family = "nb", method = "REML", select = TRUE)
gam.check(deep.numsp.nitrate)
```

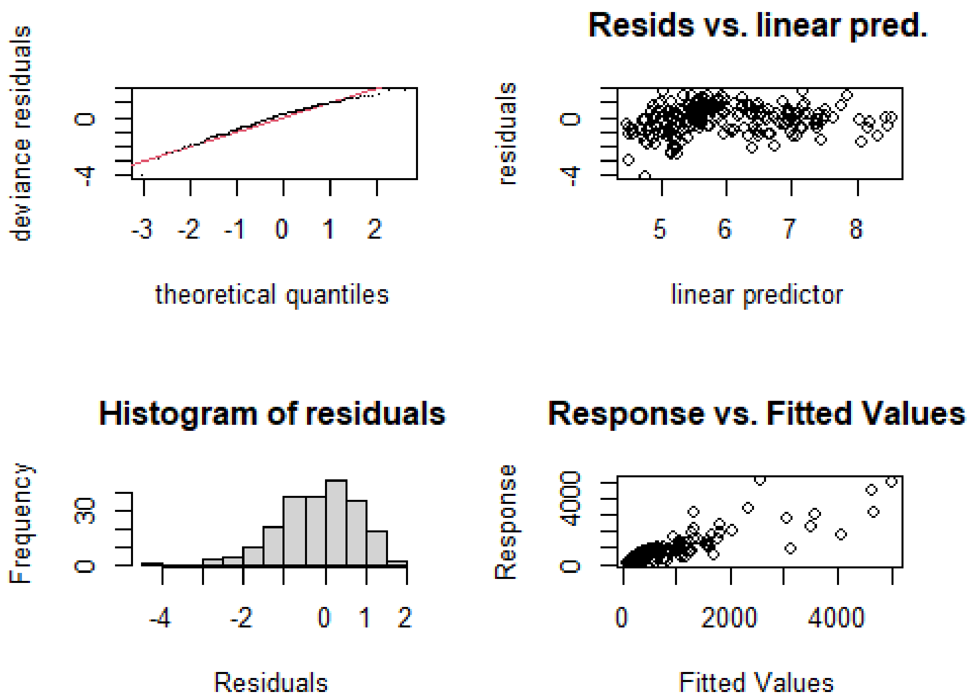


```
##
## Method: REML   Optimizer: outer newton
## full convergence after 10 iterations.
## Gradient range [-0.0009922308,0.0002871979]
## (score 1497.007 & scale 1).
## Hessian positive definite, eigenvalue range [0.009678219,97.09976].
## Model rank =  68 / 68
##
## Basis dimension (k) checking results. Low p-value (k-index<1) may
## indicate that k is too low, especially if edf is close to k'.
##
##                            k'   edf k-index p-value
## s(Latitude,Longitude) 49.00 13.22    0.83   0.005 **
## s(NumRec_dee)          9.00  8.56    0.54  <2e-16 ***
## s(Nitrate_Bot_Mean)    9.00  1.67    0.81   0.005 **
## ---
## Signif. codes:  0 '***' 0.001 '**' 0.01 '*' 0.05 '.' 0.1 ' ' 1

summary(deep.numsp.nitrate)

##
## Family: Negative Binomial(3.141)
## Link function: log
##
## Formula:
## NumSpe_dee ~ s(Latitude, Longitude, bs = "sos") + s(NumRec_dee) +
##     s(Nitrate_Bot_Mean)
##
## Parametric coefficients:
##             Estimate Std. Error z value Pr(>|z|)
```

```
## (Intercept)  5.94923    0.03852   154.4   <2e-16 ***
## ---
## Signif. codes:  0 '***' 0.001 '**' 0.01 '*' 0.05 '.' 0.1 ' ' 1
##
## Approximate significance of smooth terms:
##                              edf Ref.df   Chi.sq p-value
## s(Latitude,Longitude) 13.219      49   51.307  <2e-16 ***
## s(NumRec_dee)           8.555       9  348.951  <2e-16 ***
## s(Nitrate_Bot_Mean)     1.669       9    4.873  0.0218 *
## ---
## Signif. codes:  0 '***' 0.001 '**' 0.01 '*' 0.05 '.' 0.1 ' ' 1
##
## R-sq.(adj) =  0.698   Deviance explained = 75.5%
## -REML =   1497  Scale est. = 1         n = 217
```

```
deep.numsp.o2 <- gam(NumSpe_dee ~ s(Latitude, Longitude, bs = "sos") + s(
NumRec_dee) + s(O2_Bot_Mean), data = Ecological_Data_Global_Hex_sp, famil
y = "nb", method = "REML", select = TRUE)
gam.check(deep.numsp.o2)
```

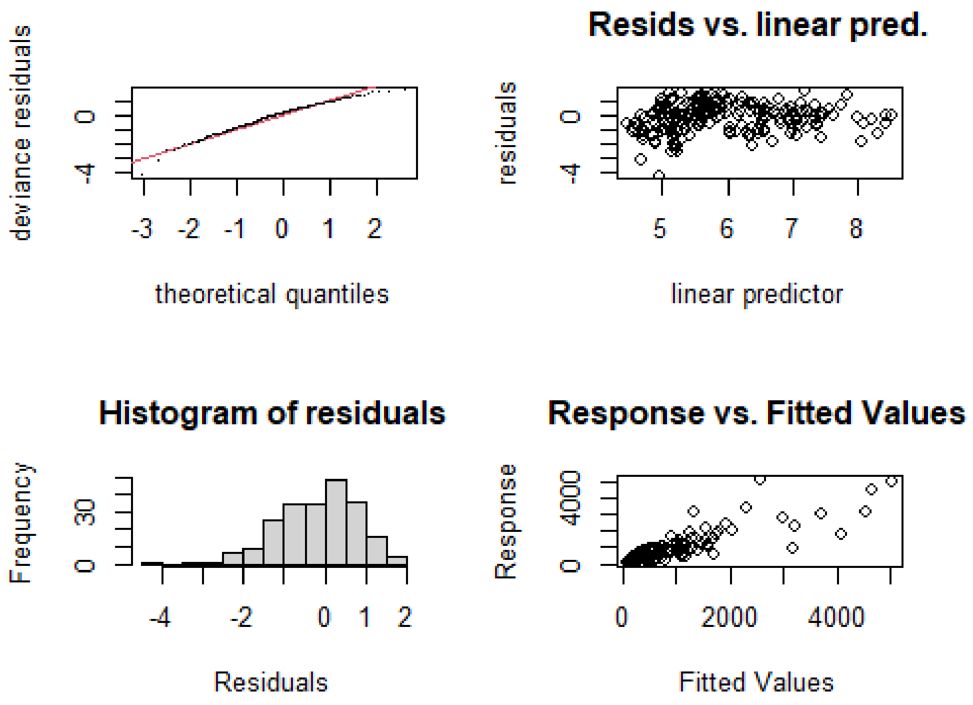


```
##
## Method: REML   Optimizer: outer newton
## full convergence after 11 iterations.
## Gradient range [-0.0003422706,0.0002318808]
## (score 1497.719 & scale 1).
## Hessian positive definite, eigenvalue range [1.994744e-05,96.77624].
## Model rank =  68 / 68
##
## Basis dimension (k) checking results. Low p-value (k-index<1) may
## indicate that k is too low, especially if edf is close to k'.
```

```
##
##                              k'      edf k-index p-value
## s(Latitude,Longitude) 4.90e+01 1.48e+01    0.82  <2e-16 ***
## s(NumRec_dee)         9.00e+00 8.56e+00    0.55  <2e-16 ***
## s(O2_Bot_Mean)        9.00e+00 9.54e-04    0.93    0.21
## ---
## Signif. codes:  0 '***' 0.001 '**' 0.01 '*' 0.05 '.' 0.1 ' ' 1
```

```
summary(deep.numsp.o2)
```

```
##
## Family: Negative Binomial(3.123)
## Link function: log
##
## Formula:
## NumSpe_dee ~ s(Latitude, Longitude, bs = "sos") + s(NumRec_dee) +
##     s(O2_Bot_Mean)
##
## Parametric coefficients:
##             Estimate Std. Error z value Pr(>|z|)
## (Intercept)  5.95012    0.03863     154   <2e-16 ***
## ---
## Signif. codes:  0 '***' 0.001 '**' 0.01 '*' 0.05 '.' 0.1 ' ' 1
##
## Approximate significance of smooth terms:
##                             edf Ref.df Chi.sq p-value
## s(Latitude,Longitude) 1.481e+01     49     75  <2e-16 ***
## s(NumRec_dee)         8.562e+00      9    358  <2e-16 ***
## s(O2_Bot_Mean)        9.544e-04      9      0   0.849
## ---
## Signif. codes:  0 '***' 0.001 '**' 0.01 '*' 0.05 '.' 0.1 ' ' 1
##
## R-sq.(adj) =  0.707   Deviance explained = 75.4%
## -REML = 1497.7  Scale est. = 1         n = 217
```

```
deep.numsp.PrimProd <- gam(NumSpe_dee ~ s(Latitude, Longitude, bs = "sos"
) + s(NumRec_dee) + s(PrimProd_Bot_Mean), data = Ecological_Data_Global_H
ex_sp, family = "nb", method = "REML", select = TRUE)
gam.check(deep.numsp.PrimProd)
```

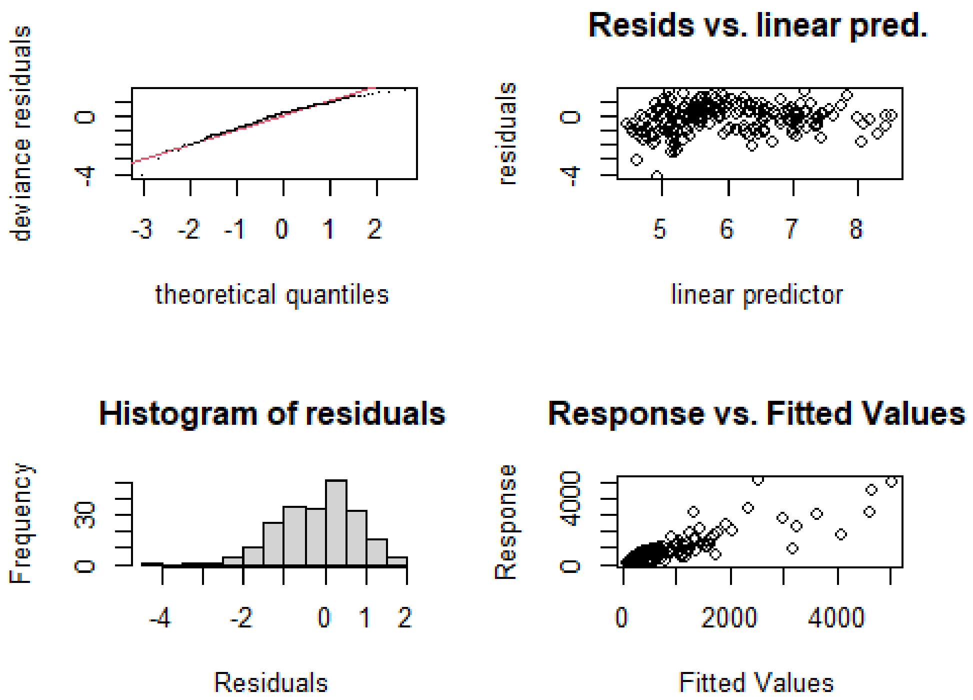


```
##
## Method: REML   Optimizer: outer newton
## full convergence after 14 iterations.
## Gradient range [-0.0003869799,0.0001542842]
## (score 1497.604 & scale 1).
## Hessian positive definite, eigenvalue range [0.000386717,95.95101].
## Model rank =  68 / 68
##
## Basis dimension (k) checking results. Low p-value (k-index<1) may
## indicate that k is too low, especially if edf is close to k'.
##
##                             k'    edf k-index p-value
## s(Latitude,Longitude) 49.000 15.126    0.82   0.005 **
## s(NumRec_dee)          9.000  8.556    0.55  <2e-16 ***
## s(PrimProd_Bot_Mean)   9.000  0.473    0.96   0.410
## ---
## Signif. codes:  0 '***' 0.001 '**' 0.01 '*' 0.05 '.' 0.1 ' ' 1
```

```
summary(deep.numsp.PrimProd)
```

```
##
## Family: Negative Binomial(3.145)
## Link function: log
##
## Formula:
## NumSpe_dee ~ s(Latitude, Longitude, bs = "sos") + s(NumRec_dee) +
##     s(PrimProd_Bot_Mean)
##
## Parametric coefficients:
##             Estimate Std. Error z value Pr(>|z|)
```

```
## (Intercept)   5.9485     0.0385   154.5   <2e-16 ***
## ---
## Signif. codes:  0 '***' 0.001 '**' 0.01 '*' 0.05 '.' 0.1 ' ' 1
##
## Approximate significance of smooth terms:
##                             edf Ref.df  Chi.sq p-value
## s(Latitude,Longitude) 15.1258     49  75.429  <2e-16 ***
## s(NumRec_dee)          8.5564      9 340.896  <2e-16 ***
## s(PrimProd_Bot_Mean)   0.4727      9   0.879    0.15
## ---
## Signif. codes:  0 '***' 0.001 '**' 0.01 '*' 0.05 '.' 0.1 ' ' 1
##
## R-sq.(adj) =  0.703   Deviance explained = 75.6%
## -REML = 1497.6  Scale est. = 1         n = 217
```

```
deep.numsp.ThemM <- gam(NumSpe_dee ~ s(Latitude, Longitude, bs = "sos") +
s(NumRec_dee) + s(ThemM_Bot_mean), data = Ecological_Data_Global_Hex_sp,
family = "nb", method = "REML", select = TRUE)
gam.check(deep.numsp.ThemM)
```

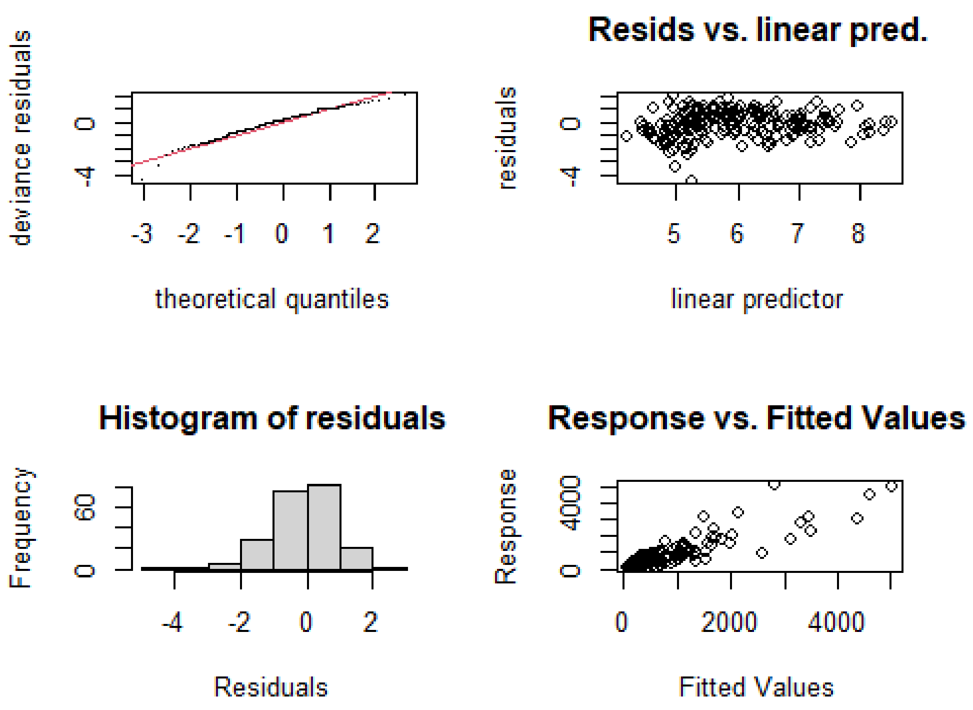


```
##
## Method: REML   Optimizer: outer newton
## full convergence after 8 iterations.
## Gradient range [-0.0001205723,1.251459e-06]
## (score 1492.454 & scale 1).
## Hessian positive definite, eigenvalue range [0.0001205409,97.33177].
## Model rank =  68 / 68
##
## Basis dimension (k) checking results. Low p-value (k-index<1) may
## indicate that k is too low, especially if edf is close to k'.
```

```
##
##                             k'   edf k-index p-value
## s(Latitude,Longitude) 49.00  9.65    0.81  <2e-16 ***
## s(NumRec_dee)          9.00  8.52    0.59  <2e-16 ***
## s(ThemM_Bot_mean)      9.00  3.77    0.92    0.23
## ---
## Signif. codes:  0 '***' 0.001 '**' 0.01 '*' 0.05 '.' 0.1 ' ' 1
```

```
summary(deep.numsp.ThemM)
```

```
##
## Family: Negative Binomial(3.256)
## Link function: log
##
## Formula:
## NumSpe_dee ~ s(Latitude, Longitude, bs = "sos") + s(NumRec_dee) +
##     s(ThemM_Bot_mean)
##
## Parametric coefficients:
##             Estimate Std. Error z value Pr(>|z|)
## (Intercept)  5.94483    0.03785   157.1   <2e-16 ***
## ---
## Signif. codes:  0 '***' 0.001 '**' 0.01 '*' 0.05 '.' 0.1 ' ' 1
##
## Approximate significance of smooth terms:
##                         edf Ref.df Chi.sq  p-value
## s(Latitude,Longitude) 9.650     49  23.57 0.000957 ***
## s(NumRec_dee)         8.516      9 351.10  < 2e-16 ***
## s(ThemM_Bot_mean)     3.775      9  26.73 2.58e-06 ***
## ---
## Signif. codes:  0 '***' 0.001 '**' 0.01 '*' 0.05 '.' 0.1 ' ' 1
##
## R-sq.(adj) =  0.764   Deviance explained = 76.2%
## -REML = 1492.5  Scale est. = 1         n = 217
```

```
deep.numsp.env <- gam(NumSpe_dee ~ s(Latitude, Longitude, bs = "sos") + s
(NumRec_dee) + s(Depth_Mean) +  s(Margin_Sum) + s(CurVel_Bot_Mean) + s(Hu
mImp_Mean) + s(Nitrate_Bot_Mean) + s(O2_Bot_Mean) + s(PrimProd_Bot_Mean)
+ s(ThemM_Bot_mean), data = Ecological_Data_Global_Hex_sp, family = "nb",
method = "REML", select = TRUE)
gam.check(deep.numsp.env)
```

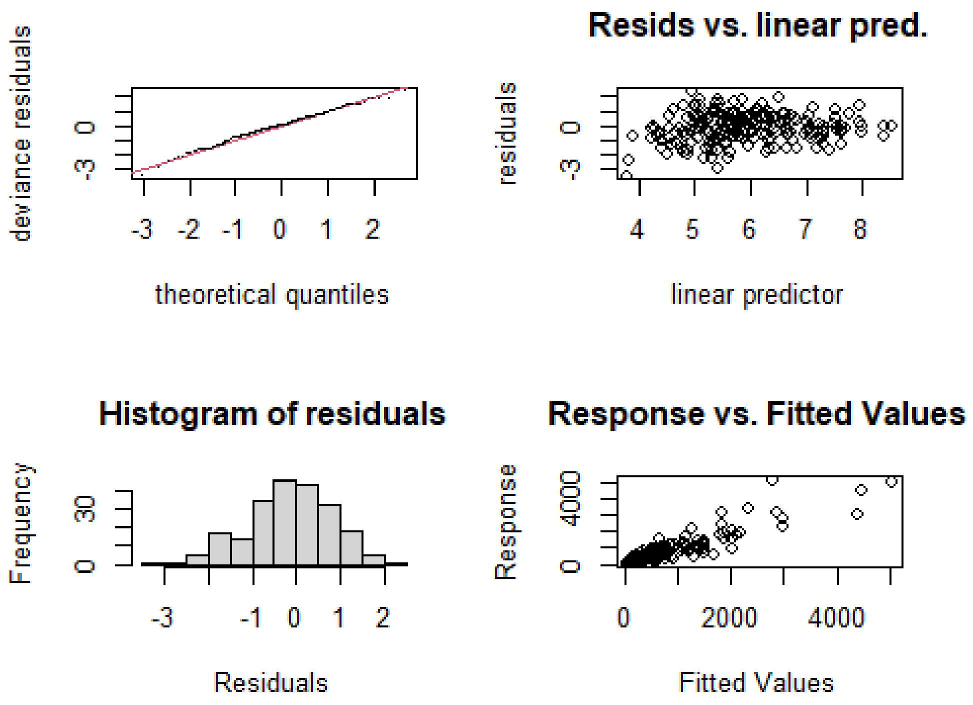


```
##
## Method: REML   Optimizer: outer newton
## full convergence after 12 iterations.
## Gradient range [-0.002010993,0.0003209862]
## (score 1477.82 & scale 1).
## Hessian positive definite, eigenvalue range [8.609159e-06,98.17527].
## Model rank =  131 / 131
##
## Basis dimension (k) checking results. Low p-value (k-index<1) may
## indicate that k is too low, especially if edf is close to k'.
##
##                              k'      edf k-index p-value
## s(Latitude,Longitude) 4.90e+01 3.55e-04    0.81  <2e-16 ***
## s(NumRec_dee)         9.00e+00 8.46e+00    0.67  <2e-16 ***
## s(Depth_Mean)         9.00e+00 2.97e+00    1.10    0.97
## s(Margin_Sum)         9.00e+00 1.47e+00    1.00    0.64
## s(CurVel_Bot_Mean)    9.00e+00 5.16e-05    1.07    0.92
## s(HumImp_Mean)        9.00e+00 9.58e-03    0.99    0.62
## s(Nitrate_Bot_Mean)   9.00e+00 1.14e+00    0.91    0.15
## s(O2_Bot_Mean)        9.00e+00 9.73e-01    0.91    0.17
## s(PrimProd_Bot_Mean)  9.00e+00 2.60e-04    1.06    0.86
## s(ThemM_Bot_mean)     9.00e+00 4.63e+00    0.98    0.54
## ---
## Signif. codes:  0 '***' 0.001 '**' 0.01 '*' 0.05 '.' 0.1 ' ' 1
```

```
summary(deep.numsp.env)
```

```
##
## Family: Negative Binomial(3.77)
## Link function: log
```

```
##
## Formula:
## NumSpe_dee ~ s(Latitude, Longitude, bs = "sos") + s(NumRec_dee) +
##     s(Depth_Mean) + s(Margin_Sum) + s(CurVel_Bot_Mean) + s(HumImp_Mean
) +
##     s(Nitrate_Bot_Mean) + s(O2_Bot_Mean) + s(PrimProd_Bot_Mean) +
##     s(ThemM_Bot_mean)
##
## Parametric coefficients:
##             Estimate Std. Error z value Pr(>|z|)
## (Intercept)  5.92563    0.03523   168.2   <2e-16 ***
## ---
## Signif. codes:  0 '***' 0.001 '**' 0.01 '*' 0.05 '.' 0.1 ' ' 1
##
## Approximate significance of smooth terms:
##                              edf Ref.df  Chi.sq  p-value
## s(Latitude,Longitude) 3.546e-04     49   0.000   0.4860
## s(NumRec_dee)         8.459e+00      9 418.122  < 2e-16 ***
## s(Depth_Mean)         2.969e+00      9  18.736 3.66e-05 ***
## s(Margin_Sum)         1.472e+00      9   3.905   0.0536 .
## s(CurVel_Bot_Mean)    5.163e-05      9   0.000   0.9906
## s(HumImp_Mean)        9.581e-03      9   0.010   0.3124
## s(Nitrate_Bot_Mean)   1.136e+00      9  28.142  < 2e-16 ***
## s(O2_Bot_Mean)        9.731e-01      9  36.184  < 2e-16 ***
## s(PrimProd_Bot_Mean)  2.601e-04      9   0.000   0.7056
## s(ThemM_Bot_mean)     4.629e+00      9  60.414  < 2e-16 ***
## ---
## Signif. codes:  0 '***' 0.001 '**' 0.01 '*' 0.05 '.' 0.1 ' ' 1
##
## R-sq.(adj) =  0.809   Deviance explained = 79.4%
## -REML = 1477.8  Scale est. = 1         n = 217
```

```
deep.numsp.models <- list(Intercept = deep.numsp.intercept,
                          LatLon = deep.numsp.latlon,
                          Depth = deep.numsp.depth,
                          ConMar = deep.numsp.margin,
                          CurVel = deep.numsp.current,
                          HumImp = deep.numsp.humimp,
                          Nitrate = deep.numsp.nitrate,
                          O2 = deep.numsp.o2,
                          PriPro = deep.numsp.PrimProd,
                          ThemMea = deep.numsp.ThemM,
                          Environment = deep.numsp.env)
deep.numsp.aic.df <- data.frame(Model = names(deep.numsp.models),
                                AIC = sapply(deep.numsp.models, function(
x) x$aic),
                                akaike.weights(sapply(deep.numsp.models,
function(x) x$aic)))

deep.numsp.aic.df <- deep.numsp.aic.df[order(deep.numsp.aic.df$AIC),]
deep.numsp.aic.df$Cumulative.Weight <- cumsum(deep.numsp.aic.df$weights)
```

```r
kable(deep.numsp.aic.df, row.names = FALSE)
```

Model	AIC	deltaAIC	rel.LL	weights	Cumulative.Weight
Environment	2889.474	0.00000	1.0000000	0.9997838	0.9997838
ConMar	2906.836	17.36188	0.0001698	0.0001698	0.9999535
Depth	2909.428	19.95419	0.0000465	0.0000464	1.0000000
ThemMea	2925.856	36.38213	0.0000000	0.0000000	1.0000000
Nitrate	2935.646	46.17152	0.0000000	0.0000000	1.0000000
PriPro	2936.027	46.55252	0.0000000	0.0000000	1.0000000
LatLon	2936.839	47.36502	0.0000000	0.0000000	1.0000000
O2	2936.841	47.36634	0.0000000	0.0000000	1.0000000
HumImp	2937.004	47.53013	0.0000000	0.0000000	1.0000000
CurVel	2937.096	47.62192	0.0000000	0.0000000	1.0000000
Intercept	3221.586	332.11204	0.0000000	0.0000000	1.0000000

```r
#write.csv(deep.numsp.aic.df, file = "deep.numsp.aic.GAM.csv")

#Plots for number of species, deep water
ggplot(Ecological_Data_Global_Hex_sp, aes(x = Depth_Mean, y = predict(dee
p.numsp.depth, Ecological_Data_Global_Hex_sp))) +
  geom_smooth(method = "gam", formula = y ~ x, color = "#1a80bb", fill =
"#85bede") +  # Add a smooth dark blue line with light blue shadow
  geom_point(size = 3) +  # Add scatter plot points
  theme_bw() +  # Use the black and white theme
  labs(
    x = "Depth (m)",  # Shorten the x-axis title
    y = "Predicted Value"  # Shorten the y-axis title
  ) +
  theme(
    panel.grid.minor = element_blank(),
    panel.grid.major = element_blank(),
    axis.text.x = element_text(size =20),  # Increase x-axis text size
    axis.text.y = element_text(size = 20),  # Increase y-axis text size
    axis.title.x = element_text(size = 22), # Increase x-axis title size
    axis.title.y = element_text(size = 22)  # Increase y-axis title size
  )
```

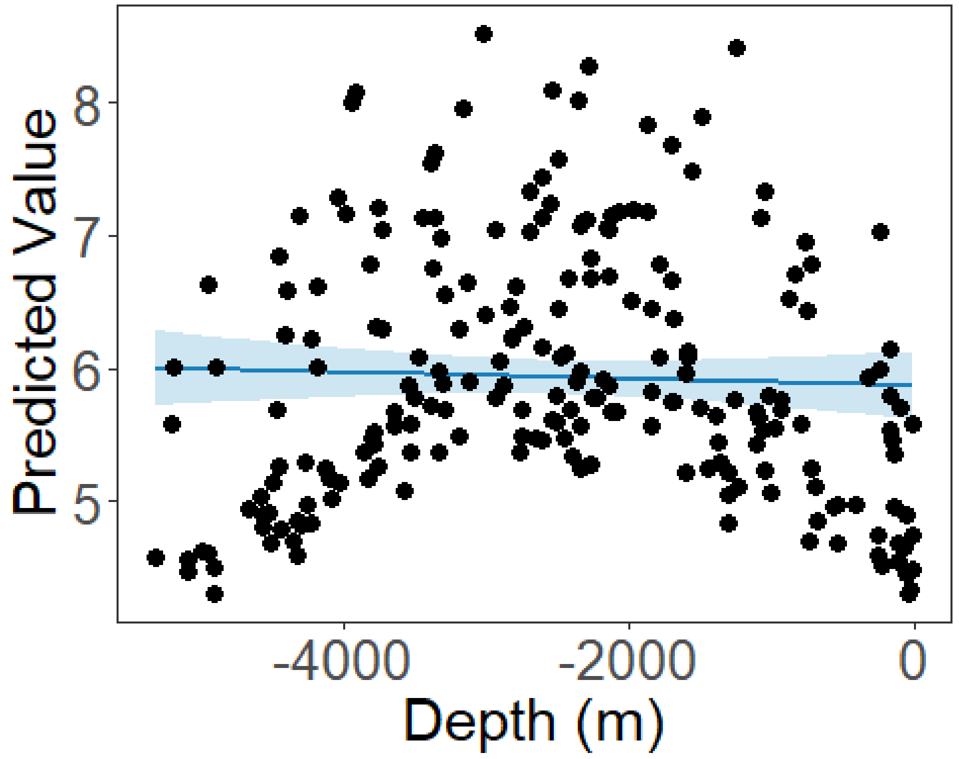


```r
ggplot(Ecological_Data_Global_Hex_sp, aes(x = Margin_Sum, y = predict(dee
p.numsp.margin, Ecological_Data_Global_Hex_sp))) +
  geom_smooth(method = "gam", formula = y ~ x, color = "#1a80bb", fill =
"#85bede") +  # Add a smooth dark blue line with light blue shadow
  geom_point(size = 3) +  # Add scatter plot points
  theme_bw() +  # Use the black and white theme
  labs(
    x = "Continental Margin (km2)",  # Shorten the x-axis title
    y = "Predicted Value"  # Shorten the y-axis title
  ) +
  theme(
    panel.grid.minor = element_blank(),
    panel.grid.major = element_blank(),
    axis.text.x = element_text(size =20),  # Increase x-axis text size
    axis.text.y = element_text(size = 20),  # Increase y-axis text size
    axis.title.x = element_text(size = 22), # Increase x-axis title size
    axis.title.y = element_text(size = 22)  # Increase y-axis title size
  )
```

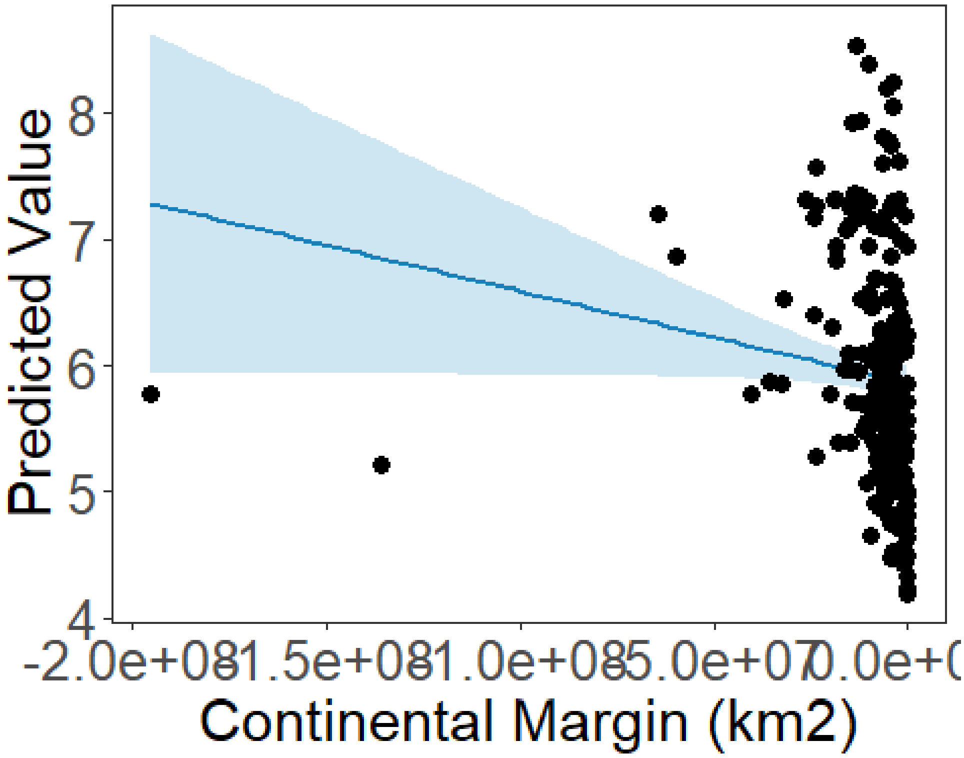


```
ggplot(Ecological_Data_Global_Hex_sp, aes(x = CurVel_Bot_Mean, y = predic
t(deep.numsp.current, Ecological_Data_Global_Hex_sp))) +
  geom_smooth(method = "gam", formula = y ~ x, color = "#1a80bb", fill =
"#85bede") +  # Add a smooth dark blue line with light blue shadow
  geom_point(size = 3) +  # Add scatter plot points
  theme_bw() +  # Use the black and white theme
  labs(
    x = "Current Velocity (m.s-1)",  # Shorten the x-axis title
    y = "Predicted Value"  # Shorten the y-axis title
  ) +
  theme(
    panel.grid.minor = element_blank(),
    panel.grid.major = element_blank(),
    axis.text.x = element_text(size =20),  # Increase x-axis text size
    axis.text.y = element_text(size = 20),  # Increase y-axis text size
    axis.title.x = element_text(size = 22), # Increase x-axis title size
    axis.title.y = element_text(size = 22)  # Increase y-axis title size
  )
```

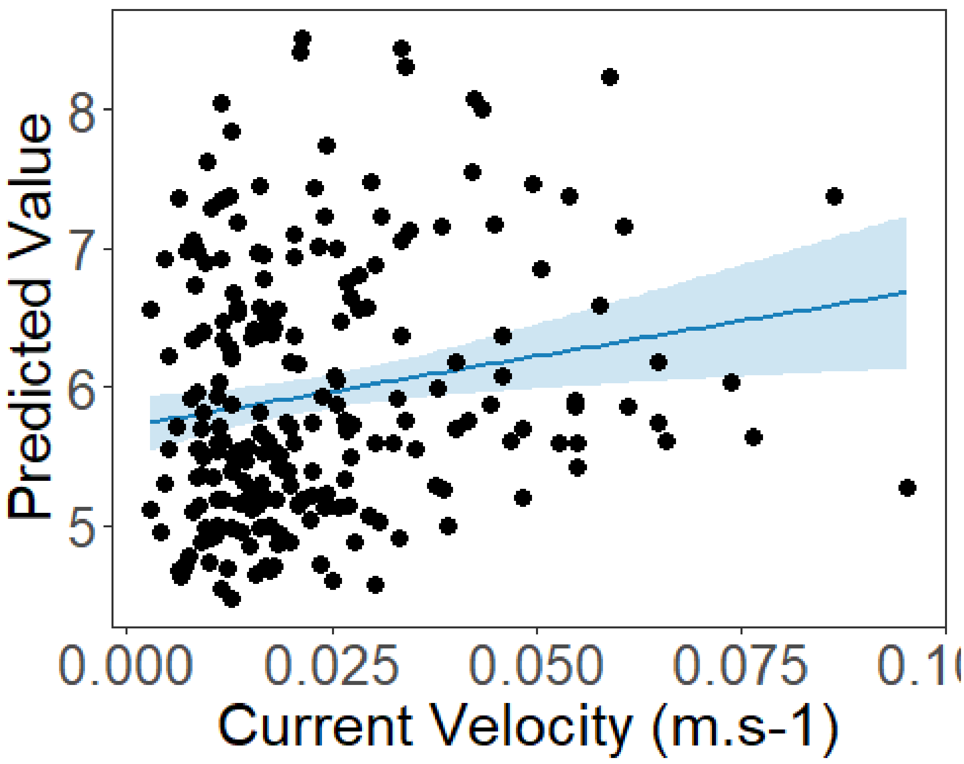


```
ggplot(Ecological_Data_Global_Hex_sp, aes(x = HumImp_Mean, y = predict(de
ep.numsp.humimp, Ecological_Data_Global_Hex_sp))) +
  geom_smooth(method = "gam", formula = y ~ x, color = "#1a80bb", fill =
"#85bede") +  # Add a smooth dark blue line with light blue shadow
  geom_point(size = 3) +  # Add scatter plot points
  theme_bw() +  # Use the black and white theme
  labs(
    x = "Human Impact",  # Shorten the x-axis title
    y = "Predicted Value"  # Shorten the y-axis title
  ) +
  theme(
    panel.grid.minor = element_blank(),
    panel.grid.major = element_blank(),
    axis.text.x = element_text(size =20),  # Increase x-axis text size
    axis.text.y = element_text(size = 20),  # Increase y-axis text size
    axis.title.x = element_text(size = 22), # Increase x-axis title size
    axis.title.y = element_text(size = 22)  # Increase y-axis title size
  )
```

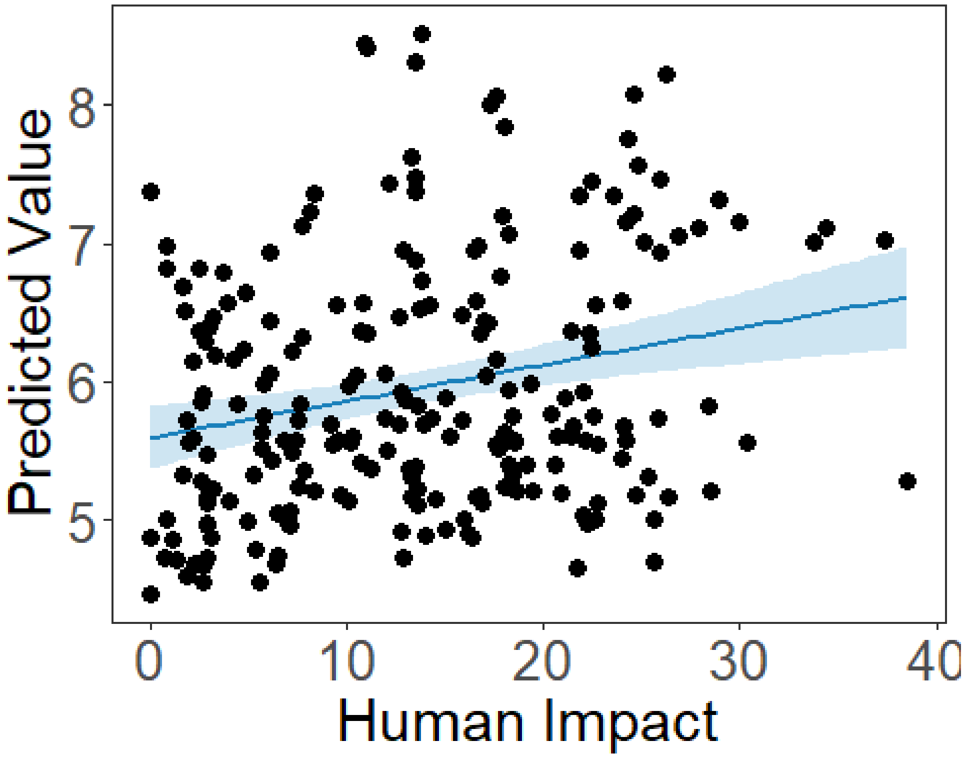


```
ggplot(Ecological_Data_Global_Hex_sp, aes(x = Nitrate_Bot_Mean, y = predi
ct(deep.numsp.nitrate, Ecological_Data_Global_Hex_sp))) +
  geom_smooth(method = "gam", formula = y ~ x, color = "#1a80bb", fill =
"#85bede") +  # Add a smooth dark blue line with light blue shadow
  geom_point(size = 3) +  # Add scatter plot points
  theme_bw() +  # Use the black and white theme
  labs(
    x = "Nitrate (mmol . m-3)",  # Shorten the x-axis title
    y = "Predicted Value"  # Shorten the y-axis title
  ) +
  theme(
    panel.grid.minor = element_blank(),
    panel.grid.major = element_blank(),
    axis.text.x = element_text(size =20),  # Increase x-axis text size
    axis.text.y = element_text(size = 20),  # Increase y-axis text size
    axis.title.x = element_text(size = 22), # Increase x-axis title size
    axis.title.y = element_text(size = 22)  # Increase y-axis title size
  )
```

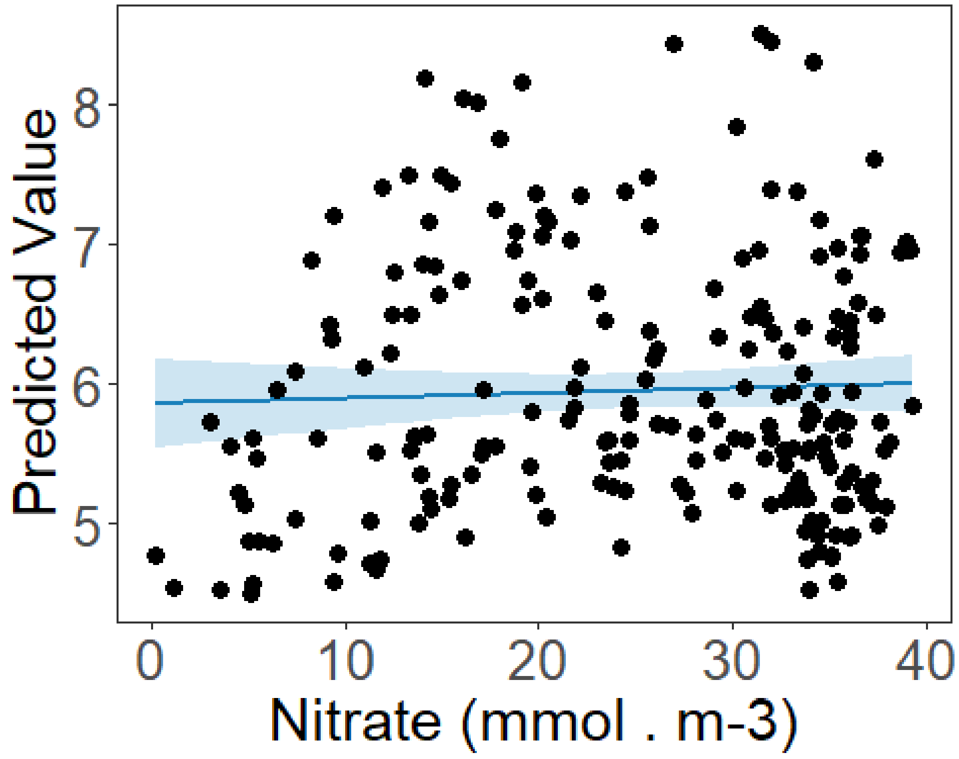


```
ggplot(Ecological_Data_Global_Hex_sp, aes(x = O2_Bot_Mean, y = predict(de
ep.numsp.o2, Ecological_Data_Global_Hex_sp))) +
  geom_smooth(method = "gam", formula = y ~ x, color = "#1a80bb", fill =
"#85bede") +  # Add a smooth dark blue line with light blue shadow
  geom_point(size = 3) +  # Add scatter plot points
  theme_bw() +  # Use the black and white theme
  labs(
    x = "O2 (mmol . m-3)",  # Shorten the x-axis title
    y = "Predicted Value"  # Shorten the y-axis title
  ) +
  theme(
    panel.grid.minor = element_blank(),
    panel.grid.major = element_blank(),
    axis.text.x = element_text(size =20),  # Increase x-axis text size
    axis.text.y = element_text(size = 20),  # Increase y-axis text size
    axis.title.x = element_text(size = 22), # Increase x-axis title size
    axis.title.y = element_text(size = 22)  # Increase y-axis title size
  )
```

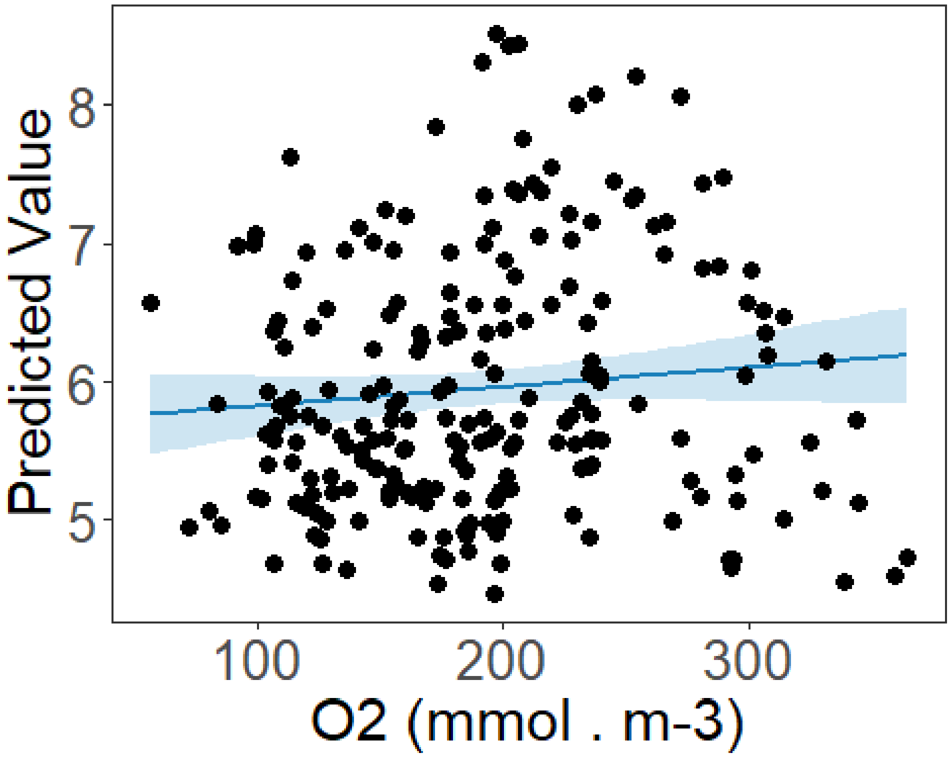


```r
ggplot(Ecological_Data_Global_Hex_sp, aes(x = PrimProd_Bot_Mean, y = pred
ict(deep.numsp.PrimProd, Ecological_Data_Global_Hex_sp))) +
  geom_smooth(method = "gam", formula = y ~ x, color = "#1a80bb", fill =
"#85bede") +  # Add a smooth dark blue line with light blue shadow
  geom_point(size = 3) +  # Add scatter plot points
  theme_bw() +  # Use the black and white theme
  labs(
    x = "Primary Productivity (mmol . m-3)",  # Shorten the x-axis title
    y = "Predicted Value"  # Shorten the y-axis title
  ) +
  theme(
    panel.grid.minor = element_blank(),
    panel.grid.major = element_blank(),
    axis.text.x = element_text(size =20),  # Increase x-axis text size
    axis.text.y = element_text(size = 20),  # Increase y-axis text size
    axis.title.x = element_text(size = 22), # Increase x-axis title size
    axis.title.y = element_text(size = 22)  # Increase y-axis title size
  )
```

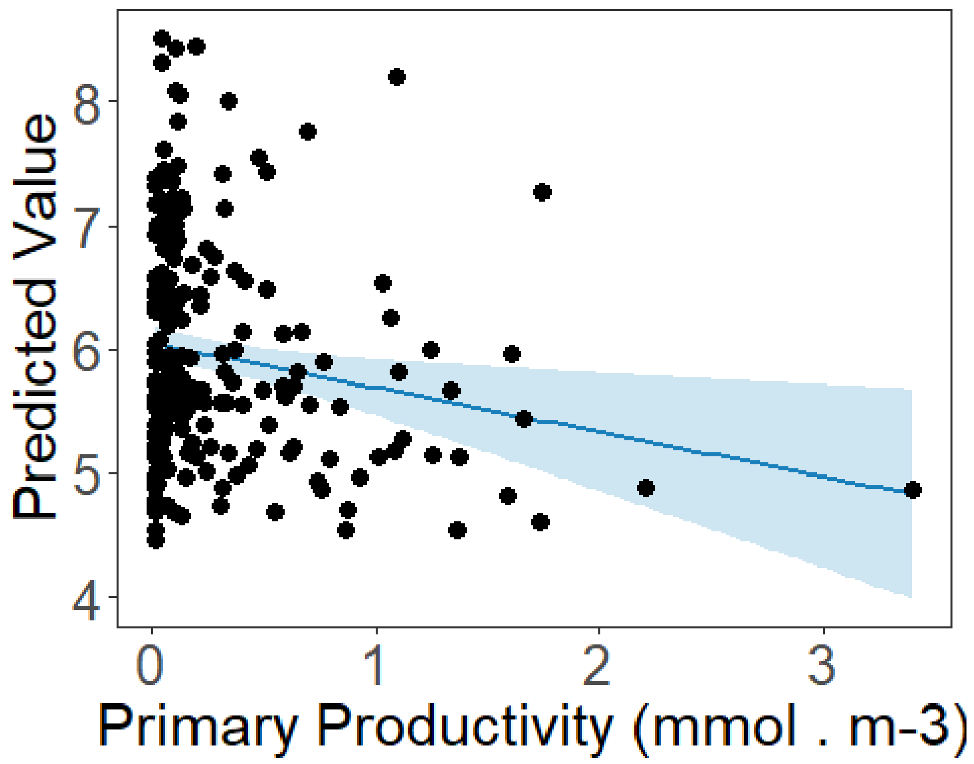


```
ggplot(Ecological_Data_Global_Hex_sp, aes(x = ThemM_Bot_mean, y = predict
(deep.numsp.ThemM, Ecological_Data_Global_Hex_sp))) +
  geom_smooth(method = "gam", formula = y ~ x, color = "#1a80bb", fill =
"#85bede") +  # Add a smooth dark blue line with light blue shadow
  geom_point(size = 3) +  # Add scatter plot points
  theme_bw() +  # Use the black and white theme
  labs(
    x = "Temperature Mean (ºC)",  # Shorten the x-axis title
    y = "Predicted Value"  # Shorten the y-axis title
  ) +
  theme(
    panel.grid.minor = element_blank(),
    panel.grid.major = element_blank(),
    axis.text.x = element_text(size =20),  # Increase x-axis text size
    axis.text.y = element_text(size = 20),  # Increase y-axis text size
    axis.title.x = element_text(size = 22), # Increase x-axis title size
    axis.title.y = element_text(size = 22)  # Increase y-axis title size
  )
```

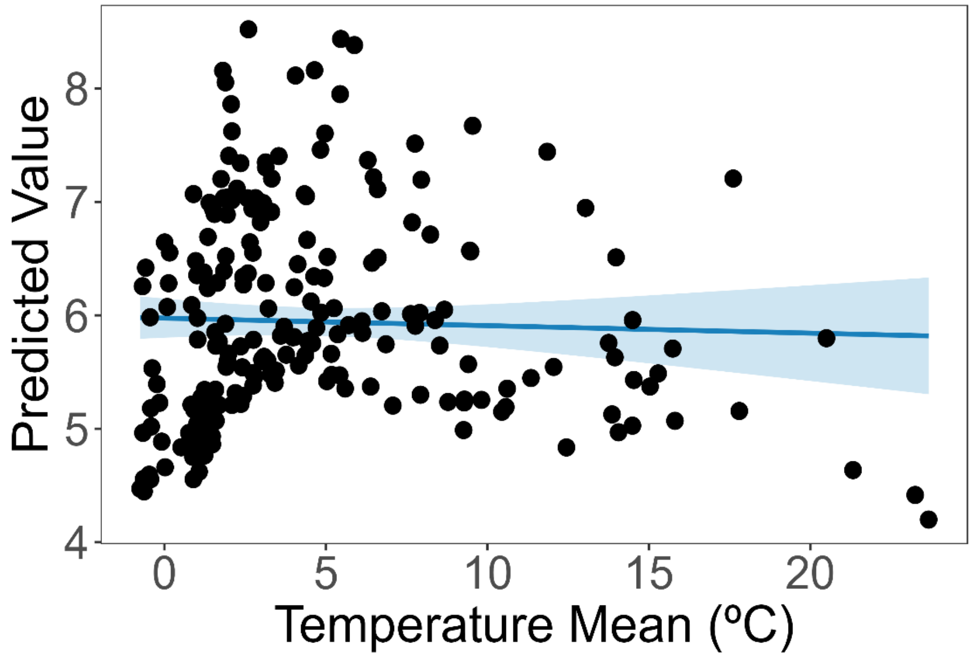


```
# ===================================
#                  DEEP-ES50-GAM
# ===================================

#GAMs for ES50, deep water
deep.ES50.intercept <- gam(ES50_dee ~ 1, data = Ecological_Data_Global_He
x_sp, family = "nb", method = "REML", select = TRUE)
gam.check(deep.ES50.intercept)
```

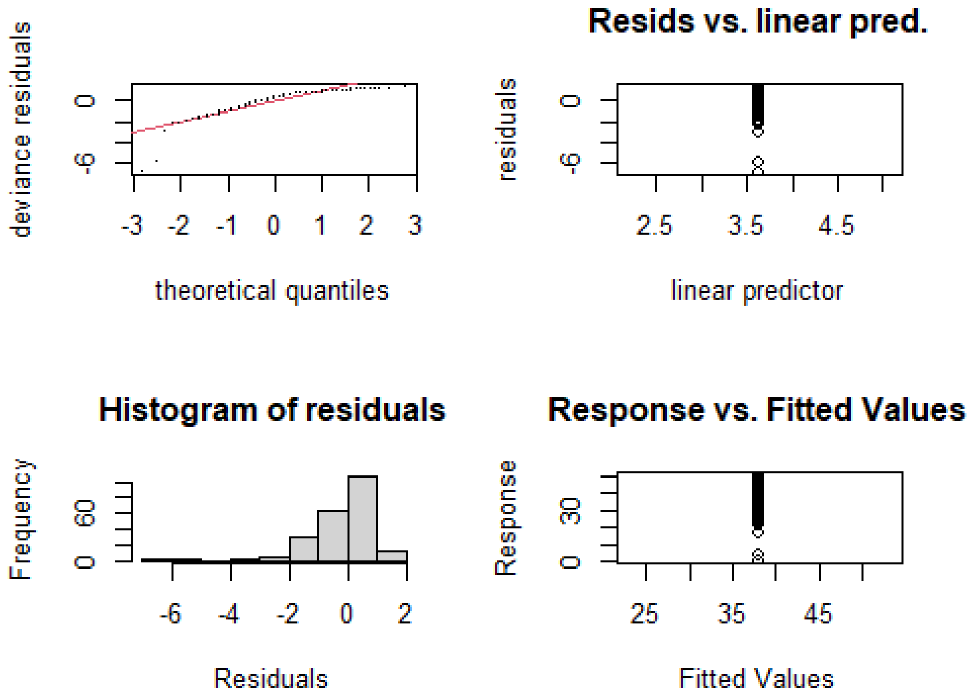


```
##
## Method: REML   Optimizer: outer newton
```

```
## full convergence after 5 iterations.
## Gradient range [-1.871906e-05,-1.871906e-05]
## (score 784.3152 & scale 1).
## Hessian positive definite, eigenvalue range [18.27899,18.27899].
## Model rank =  1 / 1
```

```
summary(deep.ES50.intercept)
```

```
##
## Family: Negative Binomial(45.988)
## Link function: log
##
## Formula:
## ES50_dee ~ 1
##
## Parametric coefficients:
##             Estimate Std. Error z value Pr(>|z|)
## (Intercept)  3.64115    0.01487   244.9   <2e-16 ***
## ---
## Signif. codes:  0 '***' 0.001 '**' 0.01 '*' 0.05 '.' 0.1 ' ' 1
##
##
## R-sq.(adj) =      0   Deviance explained = 5.36e-07%
## -REML = 784.32  Scale est. = 1         n = 217
```

```
deep.ES50.latlon <- gam(ES50_dee ~ s(Latitude, Longitude, bs = "sos"), da
ta = Ecological_Data_Global_Hex_sp, family = "nb", method = "REML", selec
t = TRUE)
gam.check(deep.ES50.latlon)
```

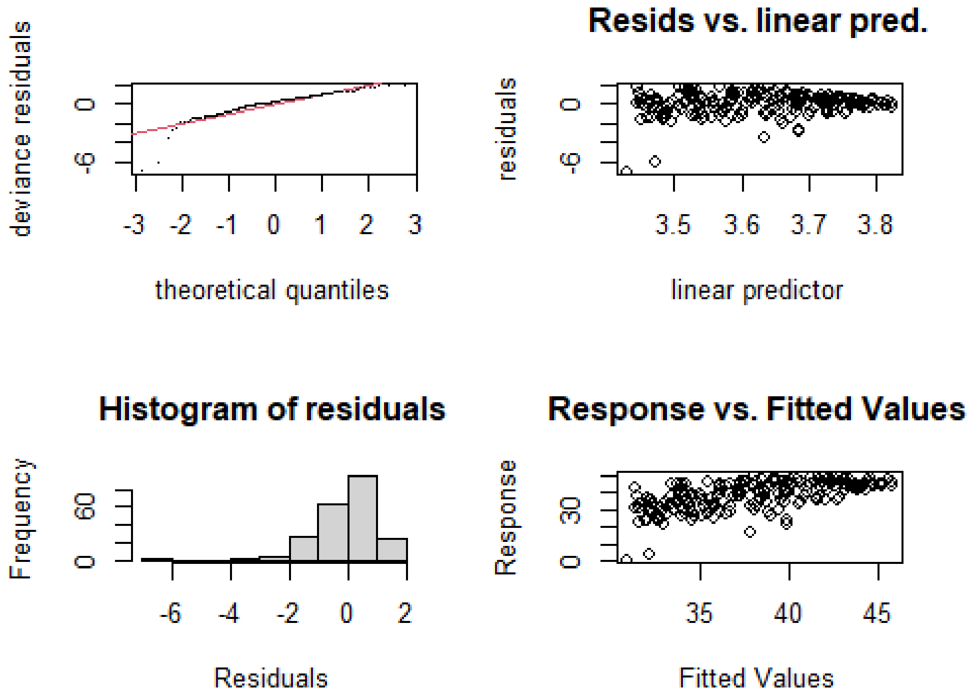

```
##
## Method: REML   Optimizer: outer newton
## full convergence after 6 iterations.
## Gradient range [-9.277844e-05,-8.118616e-06]
## (score 762.1247 & scale 1).
## Hessian positive definite, eigenvalue range [1.537282,3.846934].
## Model rank =  50 / 50
##
## Basis dimension (k) checking results. Low p-value (k-index<1) may
## indicate that k is too low, especially if edf is close to k'.
##
##                           k'   edf k-index p-value
## s(Latitude,Longitude) 49.0 15.7    0.93    0.14
```

```
summary(deep.ES50.latlon)
```

```
##
## Family: Negative Binomial(207.589)
## Link function: log
##
## Formula:
## ES50_dee ~ s(Latitude, Longitude, bs = "sos")
##
## Parametric coefficients:
##             Estimate Std. Error z value Pr(>|z|)
## (Intercept)  3.63516    0.01201   302.6   <2e-16 ***
## ---
## Signif. codes:  0 '***' 0.001 '**' 0.01 '*' 0.05 '.' 0.1 ' ' 1
##
## Approximate significance of smooth terms:
##                          edf Ref.df Chi.sq p-value
## s(Latitude,Longitude) 15.7     49  93.96  <2e-16 ***
## ---
## Signif. codes:  0 '***' 0.001 '**' 0.01 '*' 0.05 '.' 0.1 ' ' 1
##
## R-sq.(adj) =   0.31   Deviance explained = 30.3%
## -REML = 762.12  Scale est. = 1         n = 217
```

```
deep.ES50.depth <- gam(ES50_dee ~ s(Latitude, Longitude, bs = "sos") + s(
Depth_Mean), data = Ecological_Data_Global_Hex_sp, family = "nb", method
= "REML", select = TRUE)
gam.check(deep.ES50.depth)
```

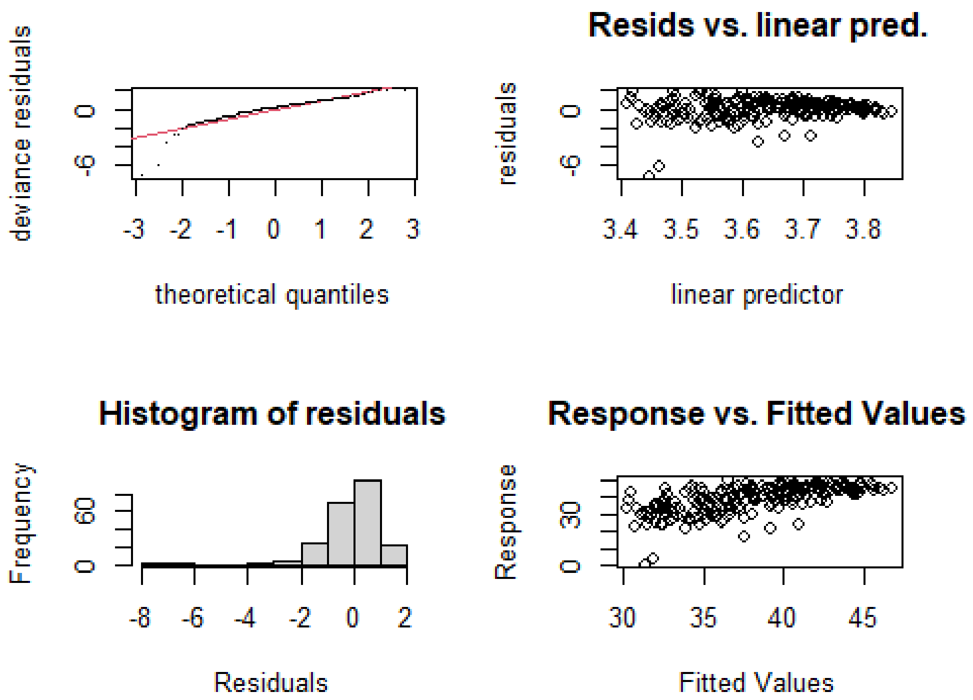


```
##
## Method: REML   Optimizer: outer newton
## full convergence after 7 iterations.
## Gradient range [-0.0004850962,-4.336401e-06]
## (score 760.2513 & scale 1).
## Hessian positive definite, eigenvalue range [7.015978e-05,3.558102].
## Model rank =  59 / 59
##
## Basis dimension (k) checking results. Low p-value (k-index<1) may
## indicate that k is too low, especially if edf is close to k'.
##
##                            k'   edf k-index p-value
## s(Latitude,Longitude) 49.00 15.15    0.94    0.18
## s(Depth_Mean)          9.00  1.96    1.13    0.98
```

```
summary(deep.ES50.depth)
```

```
##
## Family: Negative Binomial(287.813)
## Link function: log
##
## Formula:
## ES50_dee ~ s(Latitude, Longitude, bs = "sos") + s(Depth_Mean)
##
## Parametric coefficients:
##             Estimate Std. Error z value Pr(>|z|)
## (Intercept)  3.63480    0.01176   309.2   <2e-16 ***
## ---
## Signif. codes:  0 '***' 0.001 '**' 0.01 '*' 0.05 '.' 0.1 ' ' 1
##
```

```
## Approximate significance of smooth terms:
##                                edf Ref.df Chi.sq p-value
## s(Latitude,Longitude) 15.154     49 80.628  <2e-16 ***
## s(Depth_Mean)          1.963      9  7.977  0.0063 **
## ---
## Signif. codes:  0 '***' 0.001 '**' 0.01 '*' 0.05 '.' 0.1 ' ' 1
##
## R-sq.(adj) =  0.333   Deviance explained = 32.6%
## -REML = 760.25  Scale est. = 1         n = 217
```

```
deep.ES50.margin <- gam(ES50_dee ~ s(Latitude, Longitude, bs = "sos") + s
(Margin_Sum), data = Ecological_Data_Global_Hex_sp, family = "nb", method
= "REML", select = TRUE)
gam.check(deep.ES50.margin)
```

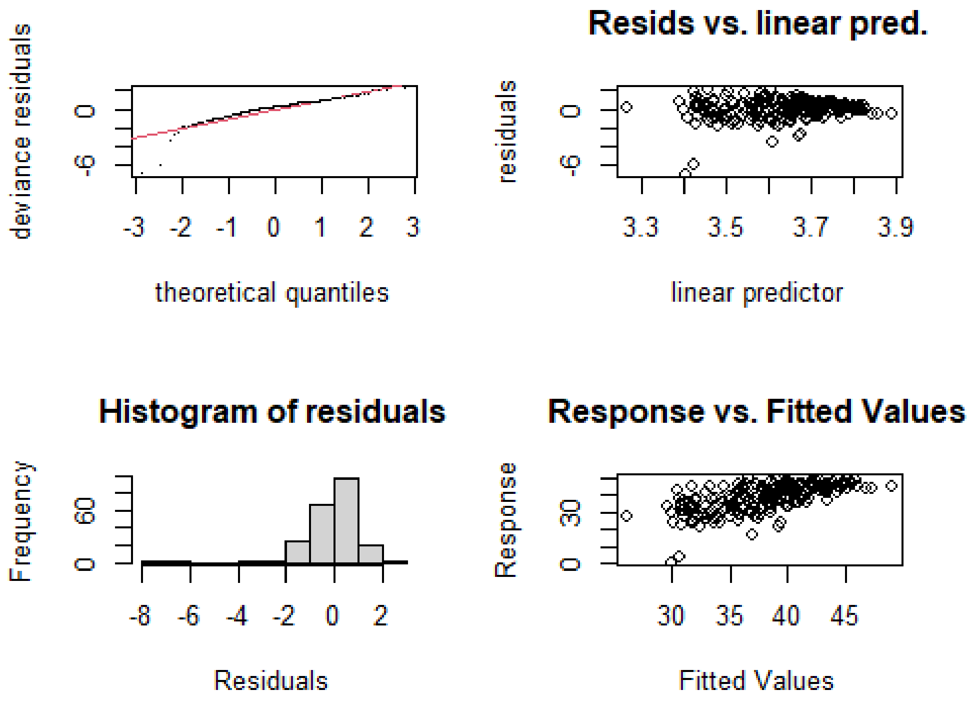


```
##
## Method: REML   Optimizer: outer newton
## full convergence after 8 iterations.
## Gradient range [-0.001019451,1.211173e-06]
## (score 756.8283 & scale 1).
## Hessian positive definite, eigenvalue range [3.269952e-05,4.141522].
## Model rank =  59 / 59
##
## Basis dimension (k) checking results. Low p-value (k-index<1) may
## indicate that k is too low, especially if edf is close to k'.
##
##                          k'   edf k-index p-value
## s(Latitude,Longitude) 49.00 16.67    0.95     0.2
## s(Margin_Sum)          9.00  2.15    0.96     0.3
```

```
summary(deep.ES50.margin)

## 
## Family: Negative Binomial(626.773) 
## Link function: log 
## 
## Formula:
## ES50_dee ~ s(Latitude, Longitude, bs = "sos") + s(Margin_Sum)
## 
## Parametric coefficients:
##             Estimate Std. Error z value Pr(>|z|)    
## (Intercept)  3.63416    0.01139   319.1   <2e-16 ***
## ---
## Signif. codes:  0 '***' 0.001 '**' 0.01 '*' 0.05 '.' 0.1 ' ' 1
## 
## Approximate significance of smooth terms:
##                           edf Ref.df Chi.sq  p-value    
## s(Latitude,Longitude) 16.675     49 111.83  < 2e-16 ***
## s(Margin_Sum)          2.145      9  16.41 7.37e-05 ***
## ---
## Signif. codes:  0 '***' 0.001 '**' 0.01 '*' 0.05 '.' 0.1 ' ' 1
## 
## R-sq.(adj) =  0.363   Deviance explained = 35.9%
## -REML = 756.83  Scale est. = 1         n = 217

deep.ES50.current <- gam(ES50_dee ~ s(Latitude, Longitude, bs = "sos") +
s(CurVel_Bot_Mean), data = Ecological_Data_Global_Hex_sp, family = "nb",
method = "REML", select = TRUE)
gam.check(deep.ES50.current)
```

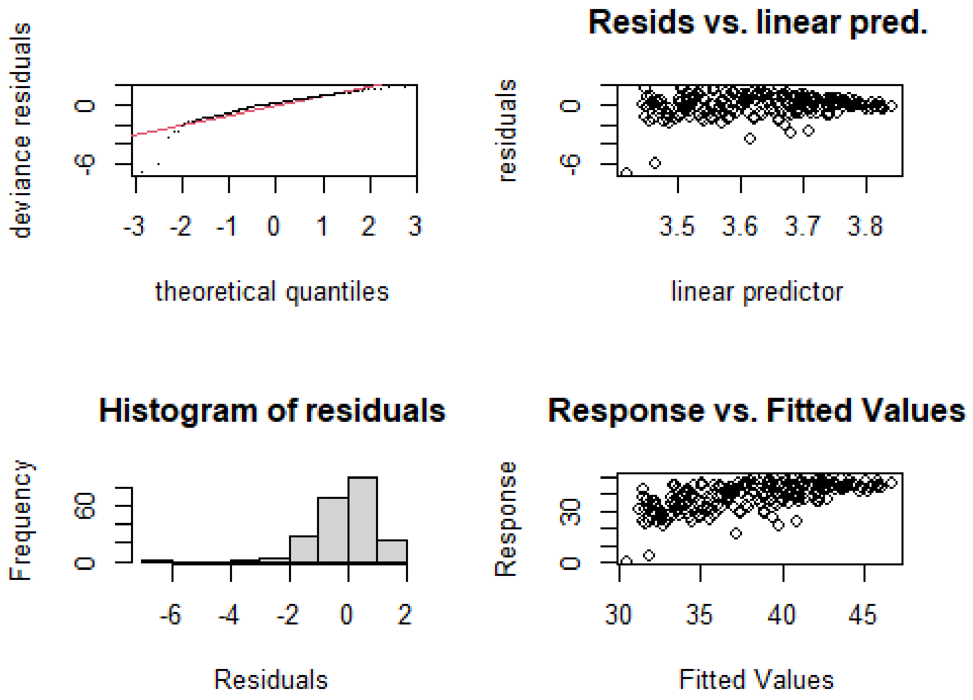

```
##
## Method: REML   Optimizer: outer newton
## full convergence after 7 iterations.
## Gradient range [-5.761453e-05,-3.383794e-09]
## (score 761.6376 & scale 1).
## Hessian positive definite, eigenvalue range [5.762204e-05,3.535314].
## Model rank =  59 / 59
##
## Basis dimension (k) checking results. Low p-value (k-index<1) may
## indicate that k is too low, especially if edf is close to k'.
##
##                              k'   edf k-index p-value
## s(Latitude,Longitude) 49.00 14.91    0.92    0.14
## s(CurVel_Bot_Mean)     9.00  1.09    1.00    0.52
```

```
summary(deep.ES50.current)
```

```
##
## Family: Negative Binomial(211.581)
## Link function: log
##
## Formula:
## ES50_dee ~ s(Latitude, Longitude, bs = "sos") + s(CurVel_Bot_Mean)
##
## Parametric coefficients:
##             Estimate Std. Error z value Pr(>|z|)
## (Intercept)    3.635      0.012     303   <2e-16 ***
## ---
## Signif. codes:  0 '***' 0.001 '**' 0.01 '*' 0.05 '.' 0.1 ' ' 1
##
## Approximate significance of smooth terms:
##                         edf Ref.df Chi.sq p-value
## s(Latitude,Longitude) 14.91     49 81.296  <2e-16 ***
## s(CurVel_Bot_Mean)     1.09      9  2.649  0.0647 .
## ---
## Signif. codes:  0 '***' 0.001 '**' 0.01 '*' 0.05 '.' 0.1 ' ' 1
##
## R-sq.(adj) =  0.311   Deviance explained = 30.7%
## -REML = 761.64  Scale est. = 1         n = 217
```

```
deep.ES50.humimp <- gam(ES50_dee ~ s(Latitude, Longitude, bs = "sos") + s
(HumImp_Mean), data = Ecological_Data_Global_Hex_sp, family = "nb", metho
d = "REML", select = TRUE)
gam.check(deep.ES50.humimp)
```

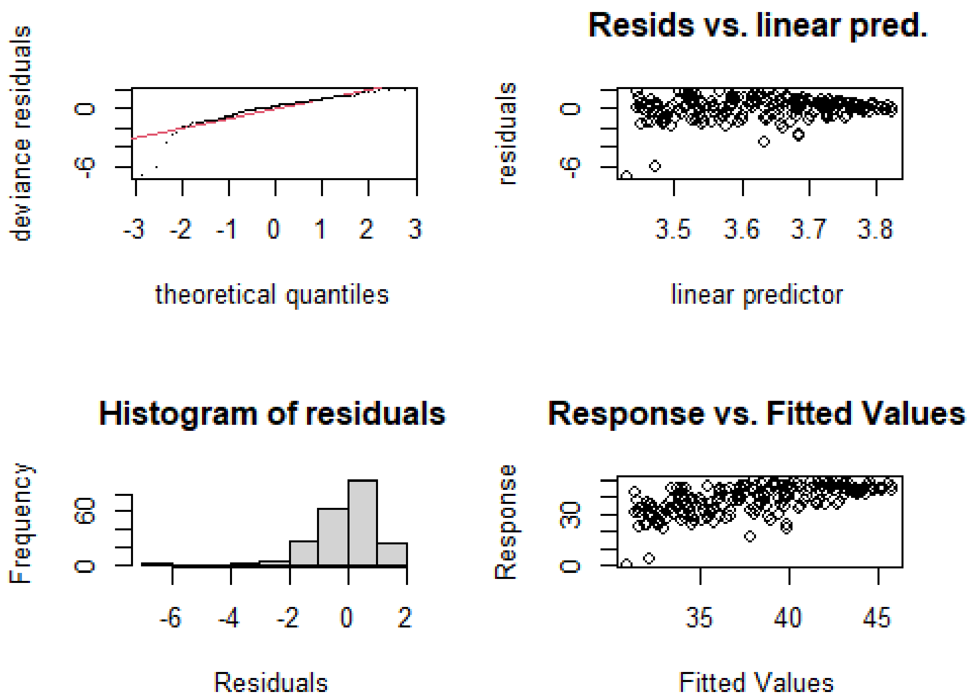


```
##
## Method: REML   Optimizer: outer newton
## full convergence after 7 iterations.
## Gradient range [-0.0003799083,1.528986e-05]
## (score 762.1251 & scale 1).
## Hessian positive definite, eigenvalue range [5.28627e-05,3.846792].
## Model rank =  59 / 59
##
## Basis dimension (k) checking results. Low p-value (k-index<1) may
## indicate that k is too low, especially if edf is close to k'.
##
##                               k'      edf k-index p-value
## s(Latitude,Longitude) 49.00000 15.70209    0.93    0.11
## s(HumImp_Mean)         9.00000  0.00112    1.10    0.96
```

```
summary(deep.ES50.humimp)
```

```
##
## Family: Negative Binomial(207.598)
## Link function: log
##
## Formula:
## ES50_dee ~ s(Latitude, Longitude, bs = "sos") + s(HumImp_Mean)
##
## Parametric coefficients:
##             Estimate Std. Error z value Pr(>|z|)
## (Intercept)  3.63516    0.01201   302.6   <2e-16 ***
## ---
## Signif. codes:  0 '***' 0.001 '**' 0.01 '*' 0.05 '.' 0.1 ' ' 1
##
```

```
## Approximate significance of smooth terms:
##                                edf Ref.df Chi.sq p-value
## s(Latitude,Longitude) 15.702093     49  93.94  <2e-16 ***
## s(HumImp_Mean)         0.001117      9   0.00   0.747
## ---
## Signif. codes:  0 '***' 0.001 '**' 0.01 '*' 0.05 '.' 0.1 ' ' 1
##
## R-sq.(adj) =   0.31   Deviance explained = 30.3%
## -REML = 762.13  Scale est. = 1         n = 217
```

```
deep.ES50.nitrate <- gam(ES50_dee ~ s(Latitude, Longitude, bs = "sos") +
s(Nitrate_Bot_Mean), data = Ecological_Data_Global_Hex_sp, family = "nb",
method = "REML", select = TRUE)
gam.check(deep.ES50.nitrate)
```

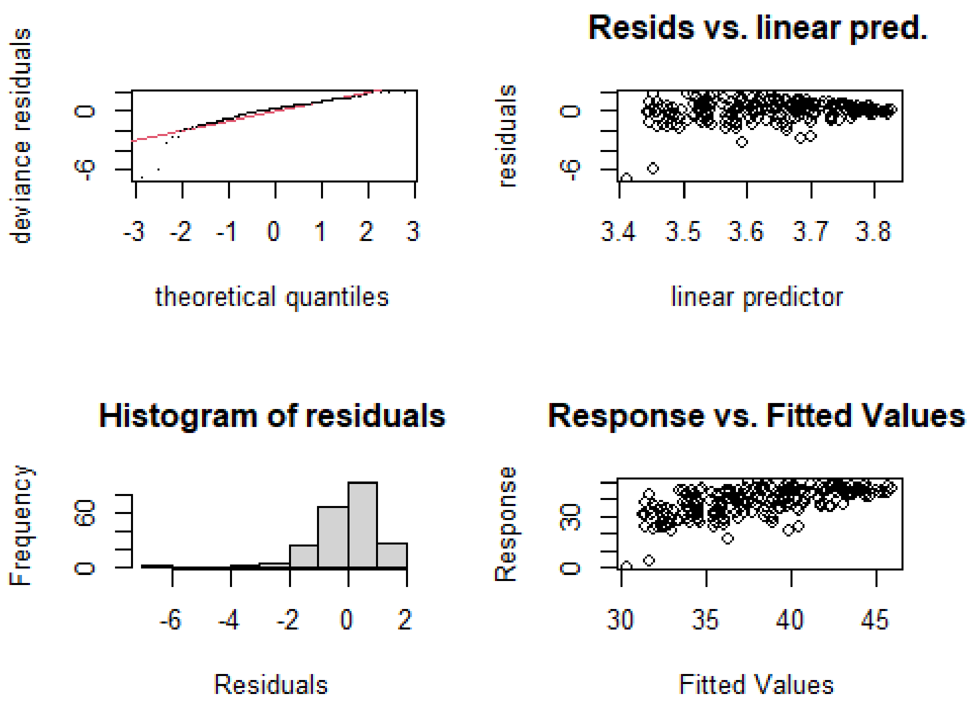


```
##
## Method: REML   Optimizer: outer newton
## full convergence after 7 iterations.
## Gradient range [-4.144649e-05,7.325799e-05]
## (score 762.0816 & scale 1).
## Hessian positive definite, eigenvalue range [3.480201e-05,3.474659].
## Model rank =  59 / 59
##
## Basis dimension (k) checking results. Low p-value (k-index<1) may
## indicate that k is too low, especially if edf is close to k'.
##
##                          k'   edf k-index p-value
## s(Latitude,Longitude) 49.00 14.56    0.93    0.14
## s(Nitrate_Bot_Mean)    9.00  1.27    0.77  <2e-16 ***
```

```
## ---
## Signif. codes:  0 '***' 0.001 '**' 0.01 '*' 0.05 '.' 0.1 ' ' 1
```

```r
summary(deep.ES50.nitrate)
```

```
##
## Family: Negative Binomial(203.834)
## Link function: log
##
## Formula:
## ES50_dee ~ s(Latitude, Longitude, bs = "sos") + s(Nitrate_Bot_Mean)
##
## Parametric coefficients:
##             Estimate Std. Error z value Pr(>|z|)
## (Intercept)  3.63518    0.01203   302.2   <2e-16 ***
## ---
## Signif. codes:  0 '***' 0.001 '**' 0.01 '*' 0.05 '.' 0.1 ' ' 1
##
## Approximate significance of smooth terms:
##                          edf Ref.df Chi.sq p-value
## s(Latitude,Longitude) 14.56     49 72.742  <2e-16 ***
## s(Nitrate_Bot_Mean)    1.27      9  2.167   0.121
## ---
## Signif. codes:  0 '***' 0.001 '**' 0.01 '*' 0.05 '.' 0.1 ' ' 1
##
## R-sq.(adj) =  0.308   Deviance explained = 30.3%
## -REML = 762.08  Scale est. = 1         n = 217
```

```r
deep.ES50.o2 <- gam(ES50_dee ~ s(Latitude, Longitude, bs = "sos") + s(O2_
Bot_Mean), data = Ecological_Data_Global_Hex_sp, family = "nb", method =
"REML", select = TRUE)
gam.check(deep.ES50.o2)
```

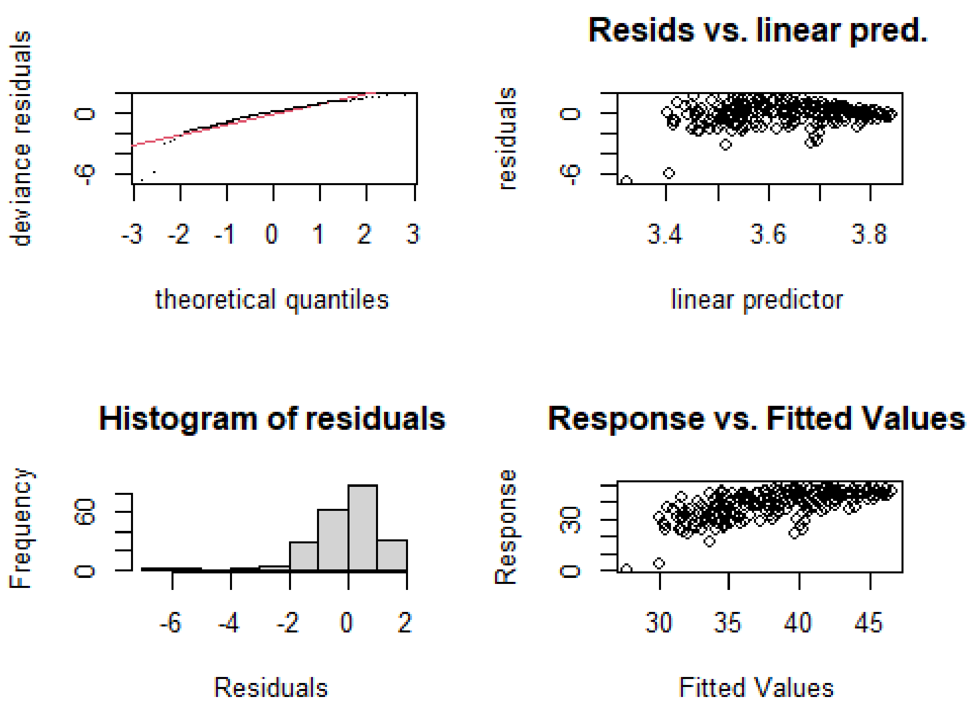


```
##
## Method: REML   Optimizer: outer newton
## full convergence after 9 iterations.
## Gradient range [-2.159673e-05,7.45825e-06]
## (score 759.6078 & scale 1).
## Hessian positive definite, eigenvalue range [2.159559e-05,3.778813].
## Model rank =  59 / 59
##
## Basis dimension (k) checking results. Low p-value (k-index<1) may
## indicate that k is too low, especially if edf is close to k'.
##
##                             k'    edf k-index p-value
## s(Latitude,Longitude) 49.000 16.941    0.93    0.10
## s(O2_Bot_Mean)          9.000  0.881    0.98    0.38

summary(deep.ES50.o2)

##
## Family: Negative Binomial(302.571)
## Link function: log
##
## Formula:
## ES50_dee ~ s(Latitude, Longitude, bs = "sos") + s(O2_Bot_Mean)
##
## Parametric coefficients:
##             Estimate Std. Error z value Pr(>|z|)
## (Intercept)  3.63457    0.01173     310   <2e-16 ***
## ---
## Signif. codes:  0 '***' 0.001 '**' 0.01 '*' 0.05 '.' 0.1 ' ' 1
##
```

```
## Approximate significance of smooth terms:
##                             edf Ref.df  Chi.sq  p-value
## s(Latitude,Longitude) 16.941     49 108.210  < 2e-16 ***
## s(O2_Bot_Mean)         0.881      9   7.383 0.000902 ***
## ---
## Signif. codes:  0 '***' 0.001 '**' 0.01 '*' 0.05 '.' 0.1 ' ' 1
##
## R-sq.(adj) =  0.334   Deviance explained = 33.8%
## -REML = 759.61  Scale est. = 1         n = 217
```

```
deep.ES50.PrimProd <- gam(ES50_dee ~ s(Latitude, Longitude, bs = "sos") +
s(PrimProd_Bot_Mean), data = Ecological_Data_Global_Hex_sp, family = "nb"
, method = "REML", select = TRUE)
gam.check(deep.ES50.PrimProd)
```

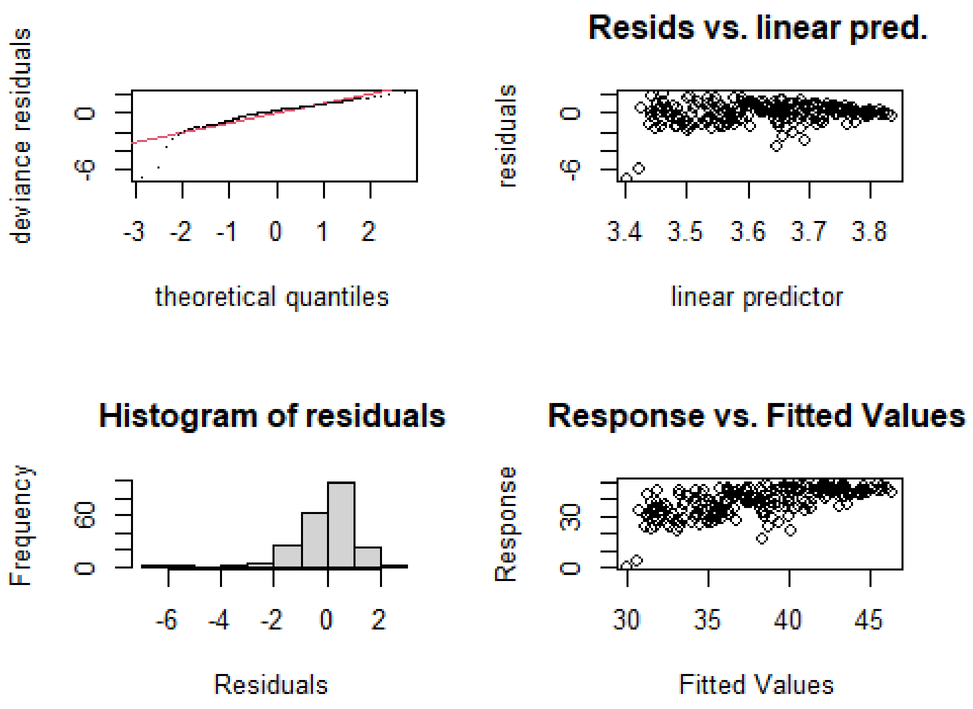


```
##
## Method: REML   Optimizer: outer newton
## full convergence after 7 iterations.
## Gradient range [-0.0001203768,-9.356184e-07]
## (score 761.1567 & scale 1).
## Hessian positive definite, eigenvalue range [5.317279e-05,3.834724].
## Model rank =  59 / 59
##
## Basis dimension (k) checking results. Low p-value (k-index<1) may
## indicate that k is too low, especially if edf is close to k'.
##
##                          k'   edf k-index p-value
## s(Latitude,Longitude) 49.00 16.06    0.93    0.13
## s(PrimProd_Bot_Mean)   9.00  1.32    1.08    0.87
```

```r
summary(deep.ES50.PrimProd)

##
## Family: Negative Binomial(256)
## Link function: log
##
## Formula:
## ES50_dee ~ s(Latitude, Longitude, bs = "sos") + s(PrimProd_Bot_Mean)
##
## Parametric coefficients:
##             Estimate Std. Error z value Pr(>|z|)
## (Intercept)  3.63491    0.01184     307   <2e-16 ***
## ---
## Signif. codes:  0 '***' 0.001 '**' 0.01 '*' 0.05 '.' 0.1 ' ' 1
##
## Approximate significance of smooth terms:
##                            edf Ref.df Chi.sq p-value
## s(Latitude,Longitude) 16.057     49 95.399  <2e-16 ***
## s(PrimProd_Bot_Mean)   1.319      9  4.193  0.0306 *
## ---
## Signif. codes:  0 '***' 0.001 '**' 0.01 '*' 0.05 '.' 0.1 ' ' 1
##
## R-sq.(adj) =  0.325   Deviance explained = 32.2%
## -REML = 761.16  Scale est. = 1         n = 217

deep.ES50.ThemM <- gam(ES50_dee ~ s(Latitude, Longitude, bs = "sos") + s(
ThemM_Bot_mean), data = Ecological_Data_Global_Hex_sp, family = "nb", met
hod = "REML", select = TRUE)
gam.check(deep.ES50.ThemM)
```

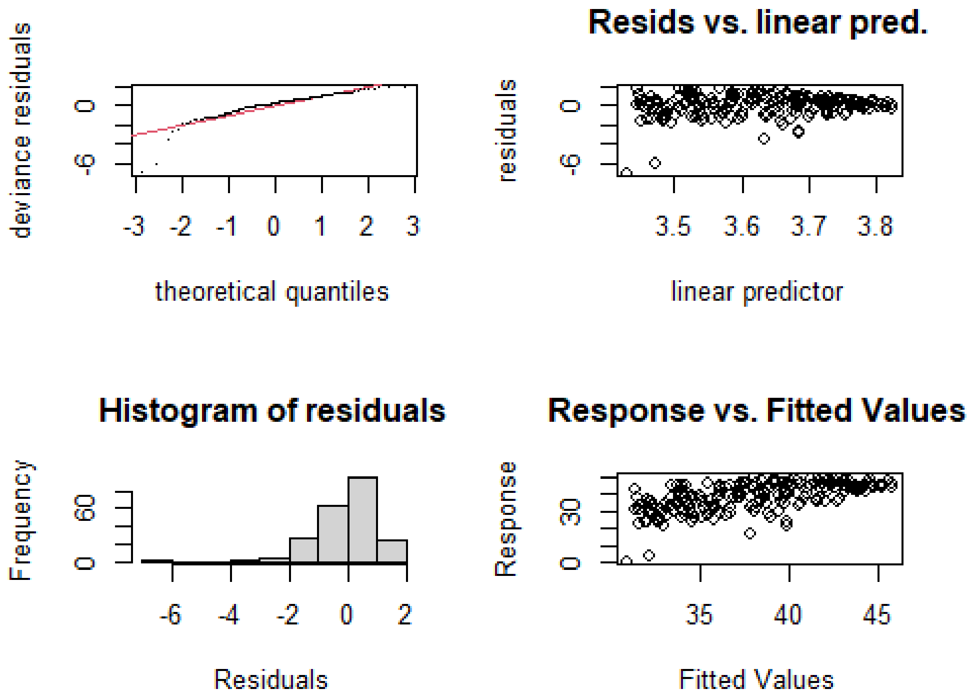

```
##
## Method: REML   Optimizer: outer newton
## full convergence after 7 iterations.
## Gradient range [-0.001254934,-3.643982e-05]
## (score 762.1248 & scale 1).
## Hessian positive definite, eigenvalue range [3.643715e-05,3.847166].
## Model rank =  59 / 59
##
## Basis dimension (k) checking results. Low p-value (k-index<1) may
## indicate that k is too low, especially if edf is close to k'.
##
##                              k'      edf k-index p-value
## s(Latitude,Longitude) 4.90e+01 1.57e+01    0.93   0.140
## s(ThemM_Bot_mean)     9.00e+00 3.18e-04    0.90   0.095 .
## ---
## Signif. codes:  0 '***' 0.001 '**' 0.01 '*' 0.05 '.' 0.1 ' ' 1
```

```
summary(deep.ES50.ThemM)
```

```
##
## Family: Negative Binomial(207.438)
## Link function: log
##
## Formula:
## ES50_dee ~ s(Latitude, Longitude, bs = "sos") + s(ThemM_Bot_mean)
##
## Parametric coefficients:
##             Estimate Std. Error z value Pr(>|z|)
## (Intercept)  3.63516    0.01201   302.6   <2e-16 ***
## ---
## Signif. codes:  0 '***' 0.001 '**' 0.01 '*' 0.05 '.' 0.1 ' ' 1
##
## Approximate significance of smooth terms:
##                              edf Ref.df Chi.sq p-value
## s(Latitude,Longitude) 1.570e+01     49  93.94  <2e-16 ***
## s(ThemM_Bot_mean)     3.183e-04      9   0.00    0.88
## ---
## Signif. codes:  0 '***' 0.001 '**' 0.01 '*' 0.05 '.' 0.1 ' ' 1
##
## R-sq.(adj) =   0.31   Deviance explained = 30.3%
## -REML = 762.12  Scale est. = 1         n = 217
```

```
deep.ES50.env <- gam(ES50_dee ~ s(Latitude, Longitude, bs = "sos") + s(De
pth_Mean) +  s(Margin_Sum) + s(CurVel_Bot_Mean) + s(HumImp_Mean) + s(Nitr
ate_Bot_Mean) + s(O2_Bot_Mean) + s(PrimProd_Bot_Mean) + s(ThemM_Bot_mean)
, data = Ecological_Data_Global_Hex_sp, family = "nb", method = "REML", s
elect = TRUE)
gam.check(deep.ES50.env)
```

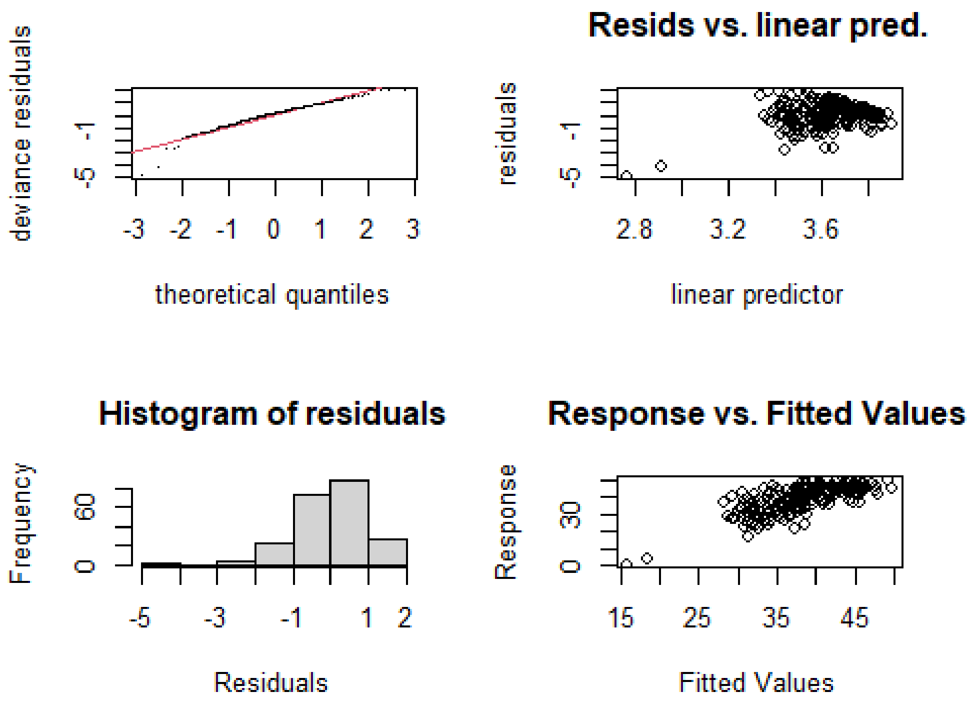


```
##
## Method: REML   Optimizer: outer newton
## full convergence after 16 iterations.
## Gradient range [-4.538378e-05,5.675004e-05]
## (score 742.0537 & scale 1).
## eigenvalue range [-5.671211e-05,3.037764].
## Model rank =  122 / 122
##
## Basis dimension (k) checking results. Low p-value (k-index<1) may
## indicate that k is too low, especially if edf is close to k'.
##
##                              k'      edf k-index p-value
## s(Latitude,Longitude) 4.90e+01 1.40e+01    0.99    0.45
## s(Depth_Mean)         9.00e+00 9.19e-01    1.11    0.93
## s(Margin_Sum)         9.00e+00 1.37e+00    0.95    0.22
## s(CurVel_Bot_Mean)    9.00e+00 1.17e-04    1.02    0.57
## s(HumImp_Mean)        9.00e+00 1.12e-04    1.09    0.90
## s(Nitrate_Bot_Mean)   9.00e+00 9.44e-01    0.89    0.05 *
## s(O2_Bot_Mean)        9.00e+00 2.18e+00    1.00    0.49
## s(PrimProd_Bot_Mean)  9.00e+00 2.33e+00    1.16    0.99
## s(ThemM_Bot_mean)     9.00e+00 3.52e+00    0.98    0.37
## ---
## Signif. codes:  0 '***' 0.001 '**' 0.01 '*' 0.05 '.' 0.1 ' ' 1
```

```
summary(deep.ES50.env)
```

```
##
## Family: Negative Binomial(10585790.5)
## Link function: log
##
```

```
## Formula:
## ES50_dee ~ s(Latitude, Longitude, bs = "sos") + s(Depth_Mean) +
##     s(Margin_Sum) + s(CurVel_Bot_Mean) + s(HumImp_Mean) + s(Nitrate_Bo
t_Mean) +
##     s(O2_Bot_Mean) + s(PrimProd_Bot_Mean) + s(ThemM_Bot_mean)
##
## Parametric coefficients:
##             Estimate Std. Error z value Pr(>|z|)
## (Intercept)   3.6310     0.0111   327.2   <2e-16 ***
## ---
## Signif. codes:  0 '***' 0.001 '**' 0.01 '*' 0.05 '.' 0.1 ' ' 1
##
## Approximate significance of smooth terms:
##                             edf Ref.df Chi.sq  p-value
## s(Latitude,Longitude) 1.405e+01     49 50.980  < 2e-16 ***
## s(Depth_Mean)         9.186e-01      9 11.233 5.02e-05 ***
## s(Margin_Sum)         1.373e+00      9  3.235   0.0706 .
## s(CurVel_Bot_Mean)    1.169e-04      9  0.000   0.7362
## s(HumImp_Mean)        1.121e-04      9  0.000   0.8164
## s(Nitrate_Bot_Mean)   9.436e-01      9 16.583 1.75e-06 ***
## s(O2_Bot_Mean)        2.179e+00      9 39.421  < 2e-16 ***
## s(PrimProd_Bot_Mean)  2.328e+00      9 20.061 5.76e-06 ***
## s(ThemM_Bot_mean)     3.524e+00      9 24.491 9.19e-07 ***
## ---
## Signif. codes:  0 '***' 0.001 '**' 0.01 '*' 0.05 '.' 0.1 ' ' 1
##
## R-sq.(adj) =  0.467   Deviance explained =   50%
## -REML = 742.05  Scale est. = 1         n = 217
```

```
deep.ES50.models <- list(Intercept = deep.ES50.intercept,
                         LatLon = deep.ES50.latlon,
                         Depth = deep.ES50.depth,
                         ConMAr = deep.ES50.margin,
                         CUrVel = deep.ES50.current,
                         HumImp = deep.ES50.humimp,
                         Nitrate = deep.ES50.nitrate,
                         O2 = deep.ES50.o2,
                         PriPro = deep.ES50.PrimProd,
                         TemMea = deep.ES50.ThemM,
                         Environment = deep.ES50.env)
deep.ES50.aic.df <- data.frame(Model = names(deep.ES50.models),
                               AIC = sapply(deep.ES50.models, function(x)
x$aic),
                               akaike.weights(sapply(deep.ES50.models, fu
nction(x) x$aic)))

deep.ES50.aic.df <- deep.ES50.aic.df[order(deep.ES50.aic.df$AIC),]
deep.ES50.aic.df$Cumulative.Weight <- cumsum(deep.ES50.aic.df$weights)

kable(deep.ES50.aic.df, row.names = FALSE)
```

Model	AIC	deltaAIC	rel.LL	weights	Cumulative.Weight
Environment	1443.312	0.00000	1	1	1
ConMAr	1488.893	45.58086	0	0	1
O2	1495.501	52.18915	0	0	1
Depth	1498.585	55.27363	0	0	1
PriPro	1500.548	57.23622	0	0	1
CUrVel	1503.476	60.16389	0	0	1
LatLon	1504.125	60.81352	0	0	1
HumImp	1504.127	60.81492	0	0	1
TemMea	1504.128	60.81611	0	0	1
Nitrate	1504.415	61.10336	0	0	1
Intercept	1564.051	120.73924	0	0	1

```
#write.csv(deep.ES50.aic.df, file = "deep.ES50.aic.GAM.csv")

#Plots for ES50, deep
ggplot(Ecological_Data_Global_Hex_sp, aes(x = Depth_Mean, y = predict(dee
p.ES50.depth, Ecological_Data_Global_Hex_sp))) +
  geom_smooth(method = "gam", formula = y ~ x, color = "#1a80bb", fill =
"#85bede") +  # Add a smooth dark blue line with light blue shadow
  geom_point(size = 3) +  # Add scatter plot points
  theme_bw() +  # Use the black and white theme
  labs(
    x = "Depth (m)",  # Shorten the x-axis title
    y = "Predicted Value"  # Shorten the y-axis title
  ) +
  theme(
    panel.grid.minor = element_blank(),
    panel.grid.major = element_blank(),
    axis.text.x = element_text(size =20),  # Increase x-axis text size
    axis.text.y = element_text(size = 20),  # Increase y-axis text size
    axis.title.x = element_text(size = 22), # Increase x-axis title size
    axis.title.y = element_text(size = 22)  # Increase y-axis title size
  )
```

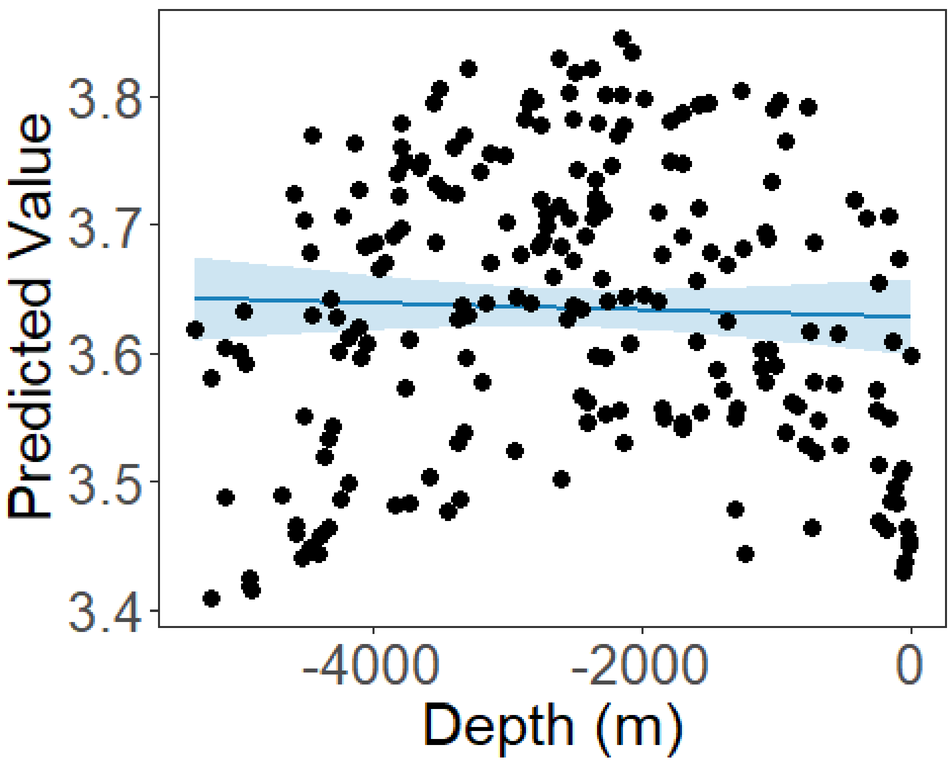


```
ggplot(Ecological_Data_Global_Hex_sp, aes(x = Margin_Sum, y = predict(dee
p.ES50.margin, Ecological_Data_Global_Hex_sp))) +
  geom_smooth(method = "gam", formula = y ~ x, color = "#1a80bb", fill =
"#85bede") +  # Add a smooth dark blue line with light blue shadow
  geom_point(size = 3) +  # Add scatter plot points
  theme_bw() +  # Use the black and white theme
  labs(
    x = "Continental Margin (km2)",  # Shorten the x-axis title
    y = "Predicted Value"  # Shorten the y-axis title
  ) +
  theme(
    panel.grid.minor = element_blank(),
    panel.grid.major = element_blank(),
    axis.text.x = element_text(size =20),  # Increase x-axis text size
    axis.text.y = element_text(size = 20),  # Increase y-axis text size
    axis.title.x = element_text(size = 22), # Increase x-axis title size
    axis.title.y = element_text(size = 22)  # Increase y-axis title size
  )
```

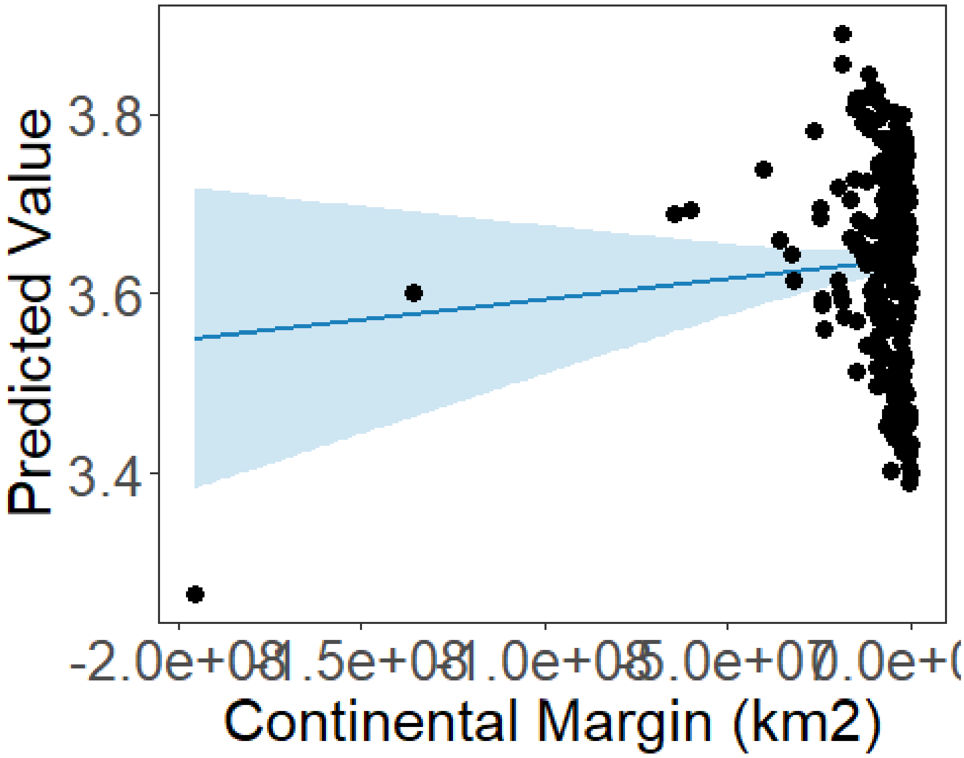


```r
ggplot(Ecological_Data_Global_Hex_sp, aes(x = CurVel_Bot_Mean, y = predic
t(deep.ES50.current, Ecological_Data_Global_Hex_sp))) +
  geom_smooth(method = "gam", formula = y ~ x, color = "#1a80bb", fill =
"#85bede") +  # Add a smooth dark blue line with light blue shadow
  geom_point(size = 3) +  # Add scatter plot points
  theme_bw() +  # Use the black and white theme
  labs(
    x = "Current Velocity (m.s-1)",  # Shorten the x-axis title
    y = "Predicted Value"  # Shorten the y-axis title
  ) +
  theme(
    panel.grid.minor = element_blank(),
    panel.grid.major = element_blank(),
    axis.text.x = element_text(size =20),  # Increase x-axis text size
    axis.text.y = element_text(size = 20),  # Increase y-axis text size
    axis.title.x = element_text(size = 22), # Increase x-axis title size
    axis.title.y = element_text(size = 22)  # Increase y-axis title size
  )
```

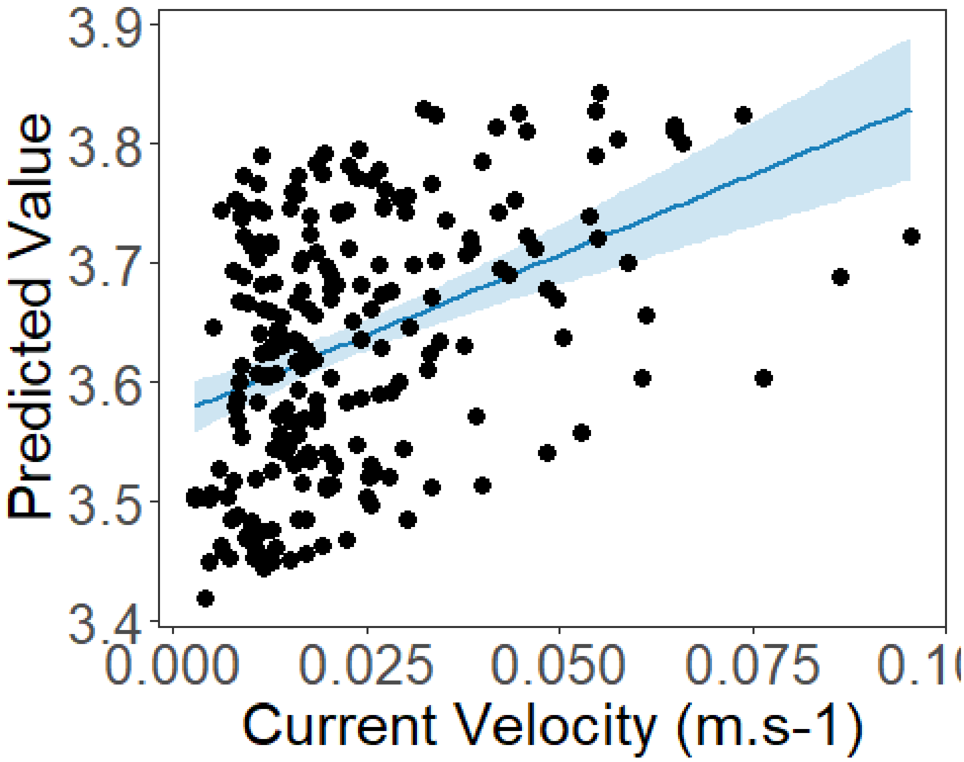


```
ggplot(Ecological_Data_Global_Hex_sp, aes(x = HumImp_Mean, y = predict(de
ep.ES50.humimp, Ecological_Data_Global_Hex_sp))) +
  geom_smooth(method = "gam", formula = y ~ x, color = "#1a80bb", fill =
"#85bede") +  # Add a smooth dark blue line with light blue shadow
  geom_point(size = 3) +  # Add scatter plot points
  theme_bw() +  # Use the black and white theme
  labs(
    x = "Human Impact",  # Shorten the x-axis title
    y = "Predicted Value"  # Shorten the y-axis title
  ) +
  theme(
    panel.grid.minor = element_blank(),
    panel.grid.major = element_blank(),
    axis.text.x = element_text(size =20),  # Increase x-axis text size
    axis.text.y = element_text(size = 20),  # Increase y-axis text size
    axis.title.x = element_text(size = 22), # Increase x-axis title size
    axis.title.y = element_text(size = 22)  # Increase y-axis title size
  )
```

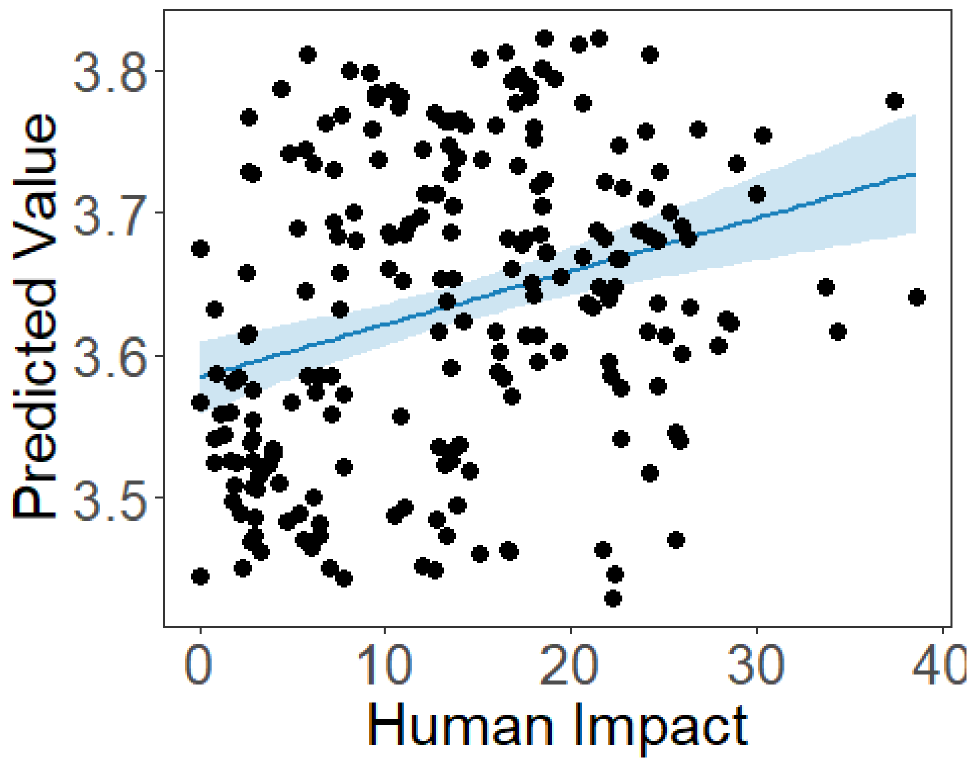


```r
ggplot(Ecological_Data_Global_Hex_sp, aes(x = Nitrate_Bot_Mean, y = predi
ct(deep.ES50.nitrate, Ecological_Data_Global_Hex_sp))) +
  geom_smooth(method = "gam", formula = y ~ x, color = "#1a80bb", fill =
"#85bede") +  # Add a smooth dark blue line with light blue shadow
  geom_point(size = 3) +  # Add scatter plot points
  theme_bw() +  # Use the black and white theme
  labs(
    x = "Nitrate (mmol . m-3)",  # Shorten the x-axis title
    y = "Predicted Value"  # Shorten the y-axis title
  ) +
  theme(
    panel.grid.minor = element_blank(),
    panel.grid.major = element_blank(),
    axis.text.x = element_text(size =20),  # Increase x-axis text size
    axis.text.y = element_text(size = 20),  # Increase y-axis text size
    axis.title.x = element_text(size = 22), # Increase x-axis title size
    axis.title.y = element_text(size = 22)  # Increase y-axis title size
  )
```

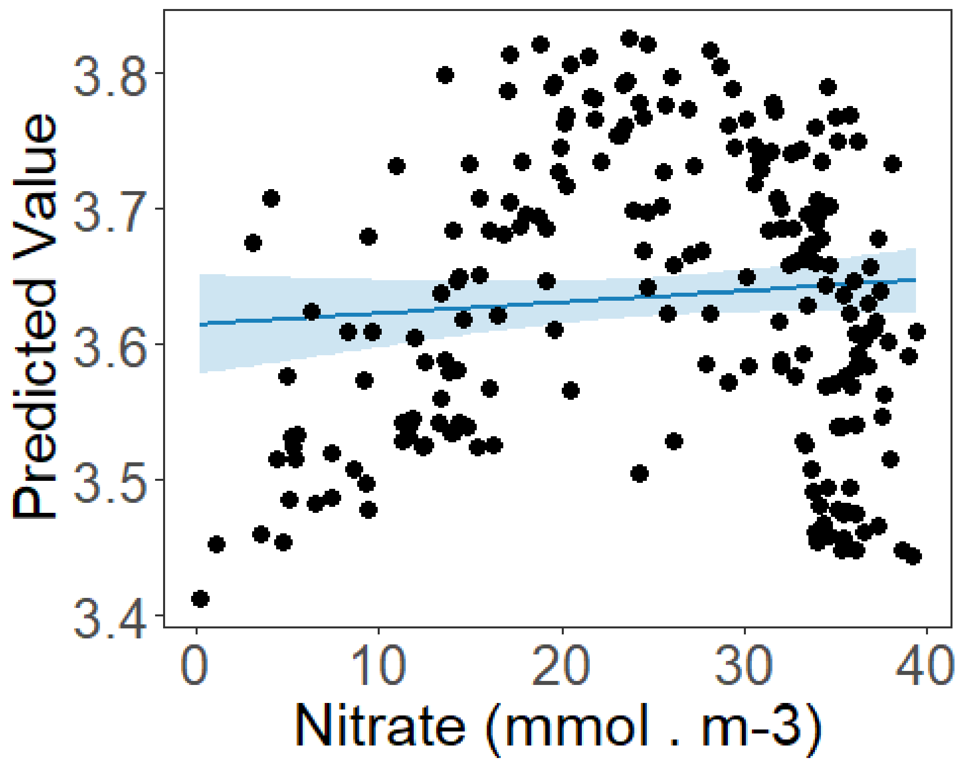


```
ggplot(Ecological_Data_Global_Hex_sp, aes(x = O2_Bot_Mean, y = predict(de
ep.ES50.o2, Ecological_Data_Global_Hex_sp))) +
  geom_smooth(method = "gam", formula = y ~ x, color = "#1a80bb", fill =
"#85bede") +  # Add a smooth dark blue line with light blue shadow
  geom_point(size = 3) +  # Add scatter plot points
  theme_bw() +  # Use the black and white theme
  labs(
    x = "O2 (mmol . m-3)",  # Shorten the x-axis title
    y = "Predicted Value"  # Shorten the y-axis title
  ) +
  theme(
    panel.grid.minor = element_blank(),
    panel.grid.major = element_blank(),
    axis.text.x = element_text(size =20),  # Increase x-axis text size
    axis.text.y = element_text(size = 20),  # Increase y-axis text size
    axis.title.x = element_text(size = 22), # Increase x-axis title size
    axis.title.y = element_text(size = 22)  # Increase y-axis title size
  )
```

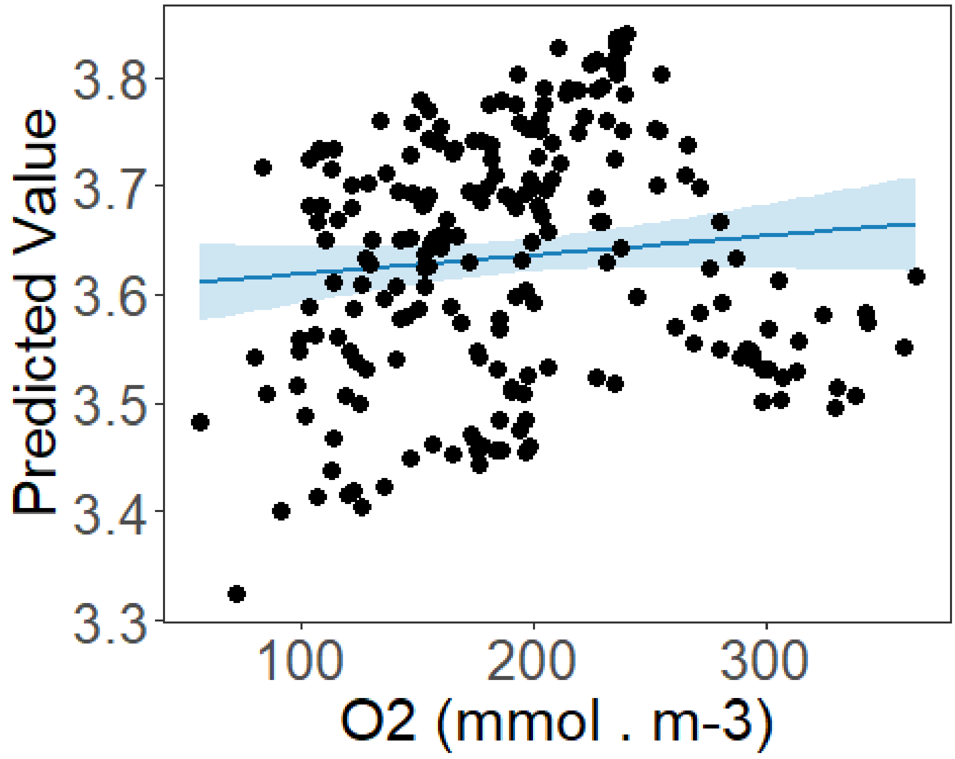


```r
ggplot(Ecological_Data_Global_Hex_sp, aes(x = PrimProd_Bot_Mean, y = predict(deep.ES50.PrimProd, Ecological_Data_Global_Hex_sp))) +
  geom_smooth(method = "gam", formula = y ~ x, color = "#1a80bb", fill = "#85bede") +  # Add a smooth dark blue line with light blue shadow
  geom_point(size = 3) +  # Add scatter plot points
  theme_bw() +  # Use the black and white theme
  labs(
    x = "Primary Productivity (mmol . m-3)",  # Shorten the x-axis title
    y = "Predicted Value"  # Shorten the y-axis title
  ) +
  theme(
    panel.grid.minor = element_blank(),
    panel.grid.major = element_blank(),
    axis.text.x = element_text(size =20),  # Increase x-axis text size
    axis.text.y = element_text(size = 20),  # Increase y-axis text size
    axis.title.x = element_text(size = 22), # Increase x-axis title size
    axis.title.y = element_text(size = 22)  # Increase y-axis title size
  )
```

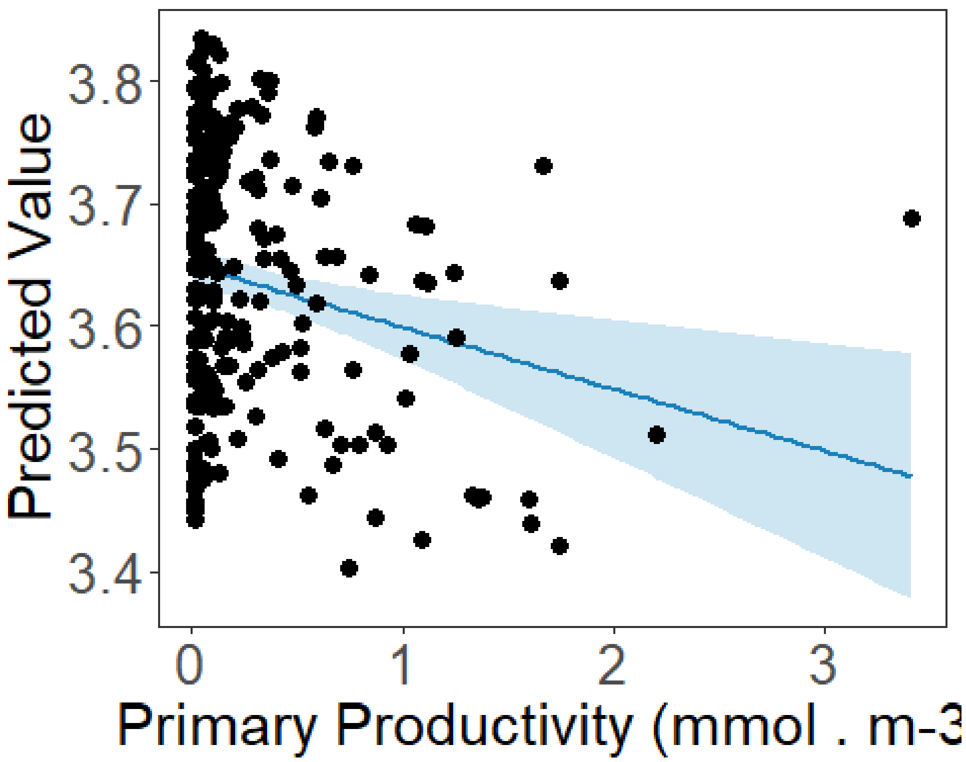


```
ggplot(Ecological_Data_Global_Hex_sp, aes(x = ThemM_Bot_mean, y = predict
(deep.ES50.ThemM, Ecological_Data_Global_Hex_sp))) +
  geom_smooth(method = "gam", formula = y ~ x, color = "#1a80bb", fill =
"#85bede") +  # Add a smooth dark blue line with light blue shadow
  geom_point(size = 3) +  # Add scatter plot points
  theme_bw() +  # Use the black and white theme
  labs(
    x = "Temperature Mean (ºC)",  # Shorten the x-axis title
    y = "Predicted Value"  # Shorten the y-axis title
  ) +
  theme(
    panel.grid.minor = element_blank(),
    panel.grid.major = element_blank(),
    axis.text.x = element_text(size =20),  # Increase x-axis text size
    axis.text.y = element_text(size = 20),  # Increase y-axis text size
    axis.title.x = element_text(size = 22), # Increase x-axis title size
    axis.title.y = element_text(size = 22)  # Increase y-axis title size
  )
```

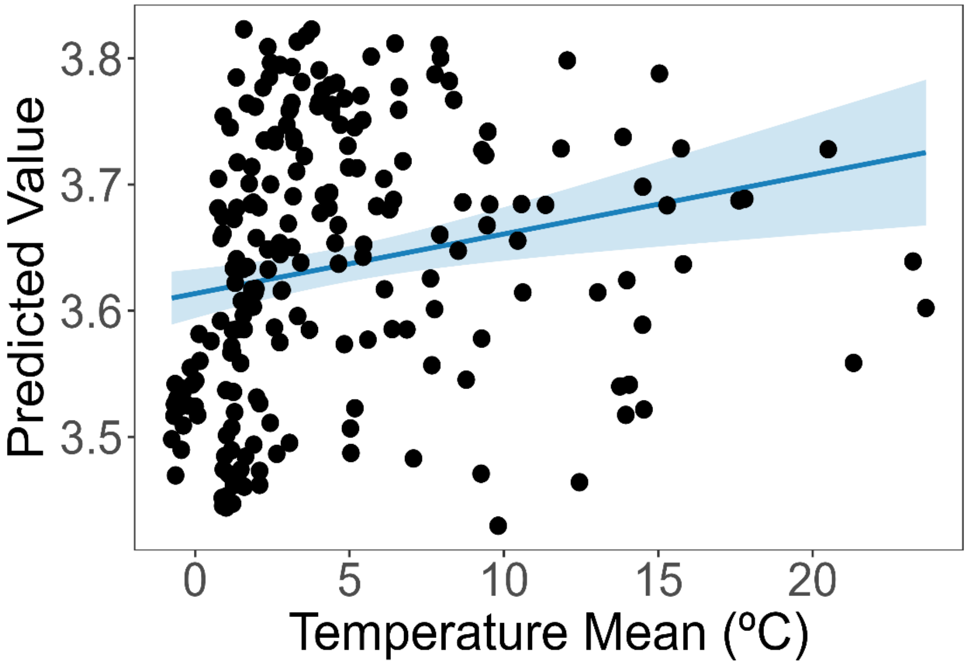
Predicted Value
3.8
3.7
3.6
3.5
0
5
10
15
20
Temperature Mean (ºC)
