# Supplementary material for: Gaps and drivers of global marine animal biodiversity from the surface to abyss
Source: Nat Commun. 2026 May 27;17:4553. doi: 10.1038/s41467-026-73613-z (PMC13216614; doi:10.1038/s41467-026-73613-z)
Supplement: Supplementary file 3 — Description of Additional Supplementary Files [file 41467_2026_73613_MOESM3_ESM.pdf]

File Name: Supplementary Data 1

Description: Supplementary Data 1 includes all dataset IDs and their corresponding citations for the occurrence data extracted from the Ocean Biodiversity Information System (OBIS).
