# Supplementary material for: Gaps and drivers of global marine animal biodiversity from the surface to abyss
Source: Nat Commun. 2026 May 27;17:4553. doi: 10.1038/s41467-026-73613-z (PMC13216614; doi:10.1038/s41467-026-73613-z)
Supplement: Supplementary file 5 — Reporting Summary [file 41467_2026_73613_MOESM5_ESM.pdf]

nature portfolio

Corresponding author(s): Hanieh Saeedi

Last updated by author(s): 17/04/2026

Reporting Summary

Nature Portfolio wishes to improve the reproducibility of the work that we publish. This form provides structure for consistency and transparency in reporting. For further information on Nature Portfolio policies, see our Editorial Policies and the Editorial Policy Checklist.

Statistics

For all statistical analyses, confirm that the following items are present in the figure legend, table legend, main text, or Methods section.

n/a	Confirmed	
☐	☒	The exact sample size (n) for each experimental group/condition, given as a discrete number and unit of measurement
☐	☒	A statement on whether measurements were taken from distinct samples or whether the same sample was measured repeatedly
☐	☒	The statistical test(s) used AND whether they are one- or two-sided<br>*Only common tests should be described solely by name; describe more complex techniques in the Methods section.*
☐	☒	A description of all covariates tested
☐	☒	A description of any assumptions or corrections, such as tests of normality and adjustment for multiple comparisons
☐	☒	A full description of the statistical parameters including central tendency (e.g. means) or other basic estimates (e.g. regression coefficient) AND variation (e.g. standard deviation) or associated estimates of uncertainty (e.g. confidence intervals)
☐	☒	For null hypothesis testing, the test statistic (e.g. F, t, r) with confidence intervals, effect sizes, degrees of freedom and P value noted<br>*Give P values as exact values whenever suitable.*
☒	☐	For Bayesian analysis, information on the choice of priors and Markov chain Monte Carlo settings
☐	☒	For hierarchical and complex designs, identification of the appropriate level for tests and full reporting of outcomes
☒	☐	Estimates of effect sizes (e.g. Cohen's d, Pearson's r), indicating how they were calculated

Our web collection on statistics for biologists contains articles on many of the points above.

Software and code

Policy information about availability of computer code

Data collection	R 4.3.2 and E packages rOBIS and rGBIF were used for data collection.
Data analysis	R 4.3.2 and QGIS 3.34.12 was used for data analysis and visualisation.

For manuscripts utilizing custom algorithms or software that are central to the research but not yet described in published literature, software must be made available to editors and reviewers. We strongly encourage code deposition in a community repository (e.g. GitHub). See the Nature Portfolio guidelines for submitting code & software for further information.

Data

Policy information about availability of data

All manuscripts must include a data availability statement. This statement should provide the following information, where applicable:

- Accession codes, unique identifiers, or web links for publicly available datasets
- A description of any restrictions on data availability
- For clinical datasets or third party data, please ensure that the statement adheres to our policy

All data are available Online through OBIS and GBIF (all the citations and dois are in the Supplementary Files 1 and 2), and all the R codes are already available in the Supplementary File 1 and R files will be Publicly available on GitHub upon acceptance of the manuscript.

Research involving human participants, their data, or biological material

Policy information about studies with human participants or human data. See also policy information about sex, gender (identity/presentation), and sexual orientation and race, ethnicity and racism.

Reporting on sex and gender	*Use the terms sex (biological attribute) and gender (shaped by social and cultural circumstances) carefully in order to avoid confusing both terms. Indicate if findings apply to only one sex or gender; describe whether sex and gender were considered in study design; whether sex and/or gender was determined based on self-reporting or assigned and methods used. Provide in the source data disaggregated sex and gender data, where this information has been collected, and if consent has been obtained for sharing of individual-level data; provide overall numbers in this Reporting Summary. Please state if this information has not been collected. Report sex- and gender-based analyses where performed, justify reasons for lack of sex- and gender-based analysis.*
Reporting on race, ethnicity, or other socially relevant groupings	*Please specify the socially constructed or socially relevant categorization variable(s) used in your manuscript and explain why they were used. Please note that such variables should not be used as proxies for other socially constructed/relevant variables (for example, race or ethnicity should not be used as a proxy for socioeconomic status). Provide clear definitions of the relevant terms used, how they were provided (by the participants/respondents, the researchers, or third parties), and the method(s) used to classify people into the different categories (e.g. self-report, census or administrative data, social media data, etc.) Please provide details about how you controlled for confounding variables in your analyses.*
Population characteristics	*Describe the covariate-relevant population characteristics of the human research participants (e.g. age, genotypic information, past and current diagnosis and treatment categories). If you filled out the behavioural & social sciences study design questions and have nothing to add here, write "See above."*
Recruitment	*Describe how participants were recruited. Outline any potential self-selection bias or other biases that may be present and how these are likely to impact results.*
Ethics oversight	*Identify the organization(s) that approved the study protocol.*

Note that full information on the approval of the study protocol must also be provided in the manuscript.

Field-specific reporting

Please select the one below that is the best fit for your research. If you are not sure, read the appropriate sections before making your selection.

☐ Life sciences ☐ Behavioural & social sciences ☒ Ecological, evolutionary & environmental sciences

For a reference copy of the document with all sections, see nature.com/documents/nr-reporting-summary-flat.pdf

Ecological, evolutionary & environmental sciences study design

All studies must disclose on these points even when the disclosure is negative.

Study description	The Ocean Biodiversity Information System (OBIS) and Global Biodiversity Information Facility (GBIF) currently host over 150 million marine occurrence records. With this global opportunity, it is time to synthesise the current knowledge of marine biodiversity and identify data gaps. This study provides a more robust description of global marine animal knowledge gaps and associated biodiversity patterns while remaining strictly descriptive rather than causal. In this study, a quality-controlled global dataset of 47,995,228 occurrence records from 184,141 marine species (Animalia) was compiled to map biodiversity patterns and model their geoecological drivers across depths and taxa. The 184,141 species included here represent approximately 87% of the existing accepted species in the World Register of Marine Species (WoRMS) and 91% of the OBIS databases. Generalised Linear Models (GLMs) and Generalised Additive Models (GAMs) were applied to assess how geoecological predictors and human impact influence species counts and ES50, accounting for sampling effort and spatial autocorrelation. This study reveals critical knowledge gaps that should be addressed to guide future sampling strategies, targeted data mobilisation, and the design of effective, science-based conservation and management policies, particularly in support of the goals of the UN Ocean Decade.
Research sample	Only accepted marine and brackish water species (Animalia) were retained for analysis. A final quality-controlled dataset of 47,995,228 occurrence records (citations to the dataset are in Supplementary Table 1 and Supplementary Data 1 belonging to 184,141 accepted marine species was compiled for the biodiversity analyses (Supplementary Figure 2). Non-animal kingdoms, including Bacteria, Chromista, Plantae, Protozoa, Fungi, or Viruses, were outside this study's scope. This scope limitation reflects current data availability and the already large scale of the analysis. Readers should note that some biodiversity patterns, particularly those of microbial and non-animal taxa, are not captured here.
Sampling strategy	No field sampling or expedition was done in this study. Only the open-access data were collected, quality controlled. and finally utilised in statistical analyses and map generations.
Data collection	Well-Known Text (WKT) was then generated for each generated ocean polygon to extract the occurrence records of Animalia from OBIS and GBIF using different packages such as "robis" and "obistools" and rgbif.
Timing and spatial scale	The study area covers all the oceans and enclosed seas worldwide from -90°S to 90°N, and -180°W to 180°E, from 0 m to 11,000 m. Base polygons of the global oceans and seas were initially extracted from the Marine Regions (www.marineregions.org) 35. The

extracted ocean polygons did not cover the whole ocean area. Thus, they were modified to ensure complete and accurate coverage of all ocean and enclosed sea boundaries for extracting species occurrence data. Modifications in QGIS 3.36.2 included adjusting boundaries, merging or splitting polygons where necessary, and correcting gaps or overlaps to fully represent the study area from -90°S to 90°N and -180°W to 180°E (Supplementary Figure 1).

Data exclusions

To clean and quality-control the merged dataset following OBIS Manual and all duplicate records and those meeting the following criteria were removed using the R package obistools: absent data, fossil records, doubtful or missing coordinates, occurrences mapped on land, or if the reported depth exceeded the maximum bathymetry for that location, with a depth margin threshold set at 50 m, and those with coordinate uncertainty exceeding 100 km. Species names were taxon-matched against the World Register of Marine Species (WoRMS) (www.marinespecies.org), reconciling synonyms and misspellings.

Reproducibility

Since all the data are open access and all the R codes are shared openly on GitHub https://github.com/haniehsaeedi/Gaps-and-drivers-of-global-marine-animal-biodiversity-from-surface-to-Abyss/tree/main and Zenodo https://zenodo.org/records/19629992, all the data analyses and generating the results and graphs will follow the FAIR principles and will be reproducible.

Randomization

To standardise sampling effort and reduce biases, animal species occurrences were aggregated into equal-sized hexagonal grids (ca. 800,000 km^2), 5-degree latitudinal bands, and equal depth intervals. Using hexagonal grids as the unit of analysis minimises edge effects, smooths over local variability, and accounts for uneven sampling across regions and depths. The chosen spatial resolutions of ca. 800,000 km^2 hexagonal grids balance global coverage, sampling completeness, and model reliability (reducing the number of grids with zero occurrences), while also improving map visualisation and colour distinction. However, this coarse scale may obscure finer local patterns, such as coastal reefs or seamount hotspots, and this limitation should be acknowledged. Additionally, 5-degree latitudinal bands were used because this resolution is widely used in global marine biodiversity research and offers a practical balance between ecological relevance and statistical robustness, providing adequate sample size per band while minimising spatial bias from uneven sampling across latitudes. The rarefaction ES50 method was used to estimate species richness, where ES50 represents the expected number of species in a random sample of 50 individuals in both hexagonal cells and 5-degree latitudinal bands. The extrapolation is performed using individual-based rarefaction: a rarefaction curve is generated from the observed species individuals, and the expected number of species for a standardised sample of 50 individuals is calculated. For each species, the probability of being represented at least once in a random subsample of 50 individuals is computed, and these probabilities are summed across all species. This approach provides a more accurate estimate of species richness while controlling for uneven sampling effort.

Blinding

Blinding was not applicable because all data were publicly available Online. But data were classified into different animal taxa and depth categories based on the existing standards.

Did the study involve field work? ☐ Yes ☒ No

Reporting for specific materials, systems and methods

We require information from authors about some types of materials, experimental systems and methods used in many studies. Here, indicate whether each material, system or method listed is relevant to your study. If you are not sure if a list item applies to your research, read the appropriate section before selecting a response.

Materials & experimental systems

n/a	Involved in the study	
☒	☐	Antibodies
☒	☐	Eukaryotic cell lines
☒	☐	Palaeontology and archaeology
☒	☐	Animals and other organisms
☒	☐	Clinical data
☒	☐	Dual use research of concern
☒	☐	Plants

Methods

n/a	Involved in the study	
☒	☐	ChIP-seq
☒	☐	Flow cytometry
☒	☐	MRI-based neuroimaging

Plants

Seed stocks

NA

Novel plant genotypes

NA

Authentication

NA
